# Simple accessible clemastine fumarate analogues as effective antileishmanials†

**DOI:** 10.1039/d4md01004c

**Published:** 2025-01-31

**Authors:** Rebecca L. Charlton, Douglas O. Escrivani, Christopher Brown, Niranjan Thota, Victor S. Agostino, Exequiel O. J. Porta, Timur Avkiran, Andrew T. Merritt, Paul W. Denny, Bartira Rossi-Bergmann, Patrick G. Steel

**Affiliations:** a Department of Chemistry, Durham University Lower Mountjoy, South Rd Durham DH1 3LE UK p.g.steel@durham.ac.uk; b Institute of Biophysics, Carlos Chagas Filho, Universidade Federal do Rio de Janeiro 21941-902 Rio de Janeiro – RJ Brazil; c LifeArc Accelerator Building, Open Innovation Campus Stevenage SG1 2FX UK; d Department of Biosciences, Durham University Lower Mountjoy, South Rd Durham DH1 3LE UK

## Abstract

Current therapeutic options for leishmaniasis are severely limited, highlighting an urgent need to develop more effective and less toxic drugs to combat a major global public health challenge. Clemastine fumarate displays good levels of antileishmanial efficacy, but further optimisation is challenged by its difficult synthesis. Here, we demonstrate that simple N-linked analogues are easier to access, can exhibit higher *selectivity* and show comparable efficacy in a mouse model of *Leishmania amazonensis* infection.

## Abstract 2

N-linked analogues of clemastine fumarate afford simpler, more accessible, more selective antileishmanials.

## Graphical abstract

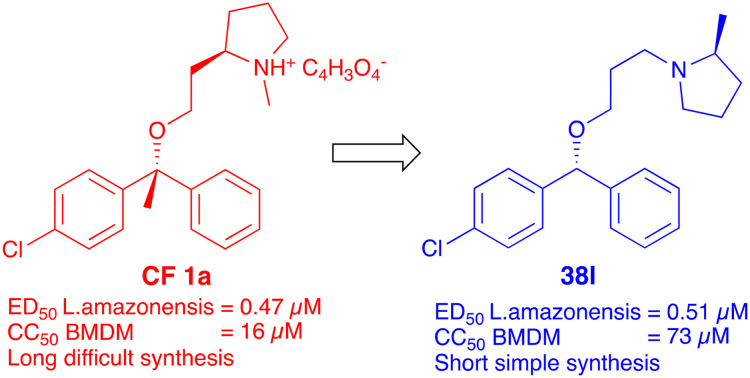

## Introduction

The leishmaniases are a set of vector borne neglected tropical diseases caused by intracellular protozoans of the genus *Leishmania* that afflict many of the world's poorest people.^1^ The disease is endemic in 98 countries, with an estimated 1.3 million new cases emerging each year leading to more than 30 000 deaths per annum.^2–4^ Globally, it has been estimated that approximately 1.5 billion people can be considered at risk with the impact, in terms of both health and economic viability, best illustrated by the loss of 3.32 million disability-adjusted life years (DALYs), accounting for 13% of all NTD-related DALYs lost.^4–7^ Moreover, the spread and severity of leishmaniasis is exacerbated by its status as an important co-infection of AIDS patients and the overlap in prevalence of HIV and *Leishmania* spp.^4^ As such, it is one of the key contributors to the global burden of NTDs, significantly and adversely impacting the socio-economic status of many low- and middle-income countries.^3^

The current treatment of *Leishmania* infections is difficult with multiple manifestations caused by 21 of the 35 known *Leishmania* species reported to infect mammals. There is currently no approved vaccine against *Leishmania* infection,^7–9^ so control of the disease relies on a limited number of drug treatments that are toxic, require intensive professional health services and are not effective across all manifestations and causative species.^10^ For example, the antimonials (Glucantime or Pentostam) and amphotericin B which are current first-line therapies require up to 20–30 daily intramuscular or intravenous injections to complete a course of treatment. In addition, these not only produce severe adverse reactions, but the unpleasant nature of the administration leads to poor patient compliance which increases the risk of the development of resistance. Currently, the only orally viable solution is miltefosine, a failed (but repurposed) antineoplastic agent, which has significant toxicity and resistance issues.^11^

**Fig. 1:**
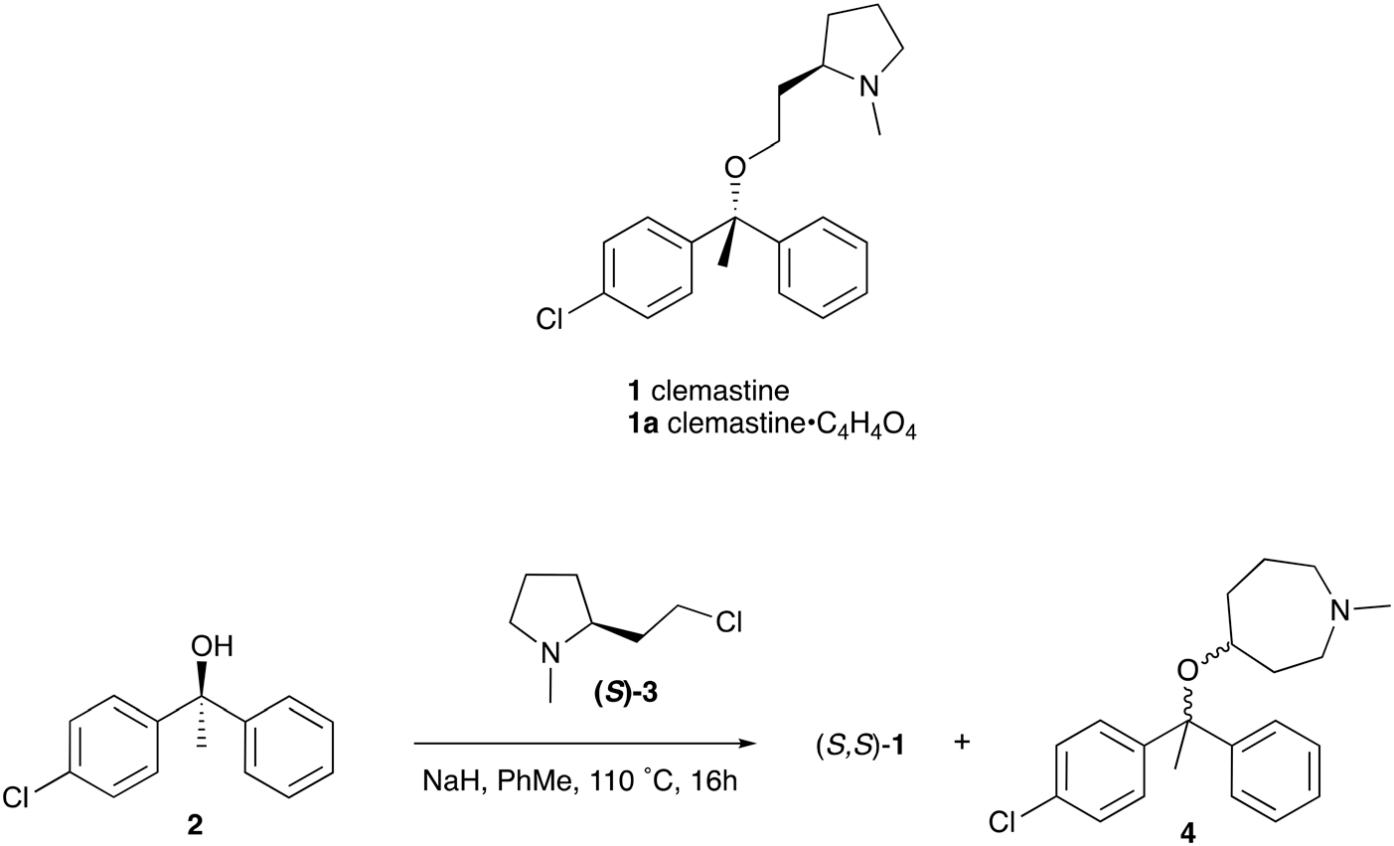
Structures of clemastine (1) and CF (1a) and previously reported synthesis of (*S*,*S*)-clemastine 1 by Clayden *et al*.^14^

Reflecting this situation, the discovery and development of new therapeutic solutions is vital. Towards this goal, in recent work, we have identified clemastine fumarate (CF) 1a as a potential new anti-leishmanial agent that functions as an inhibitor of the essential leishmanial sphingolipid biosynthetic enzyme, *inositol phosphorylceramide synthase* (IPCS). Excitingly, this activity could be translated into *in vivo* mouse models of infection.^12^ Whilst attractive as a repurposed drug,^13^ CF exhibits multiple activities. Moreover, although an over-the-counter drug, assembling the CF skeleton is surprisingly challenging due to the requirement to form a chiral tertiary ether linkage. Only two stereoselective syntheses have been reported and, in these, the ether bond is achieved by the alkylation of an enantioenriched tertiary alcohol 2 with a proline derived alkyl halide (*S*)-3.^14,15^ However, this reaction is non-trivial, occurring in low yields, and was accompanied by competing ring expansion to afford the azepane 4 (Fig. 1). As such we were interested in exploring alternative linkages that could enable more rapid SAR and, in this report, summarise our efforts in this regard and describe the formation of simpler, more accessible analogues that provide equivalent inhibition of *Leishmania* survival *in vitro* and *in vivo*.

## Results and discussion

**Scheme 1:**
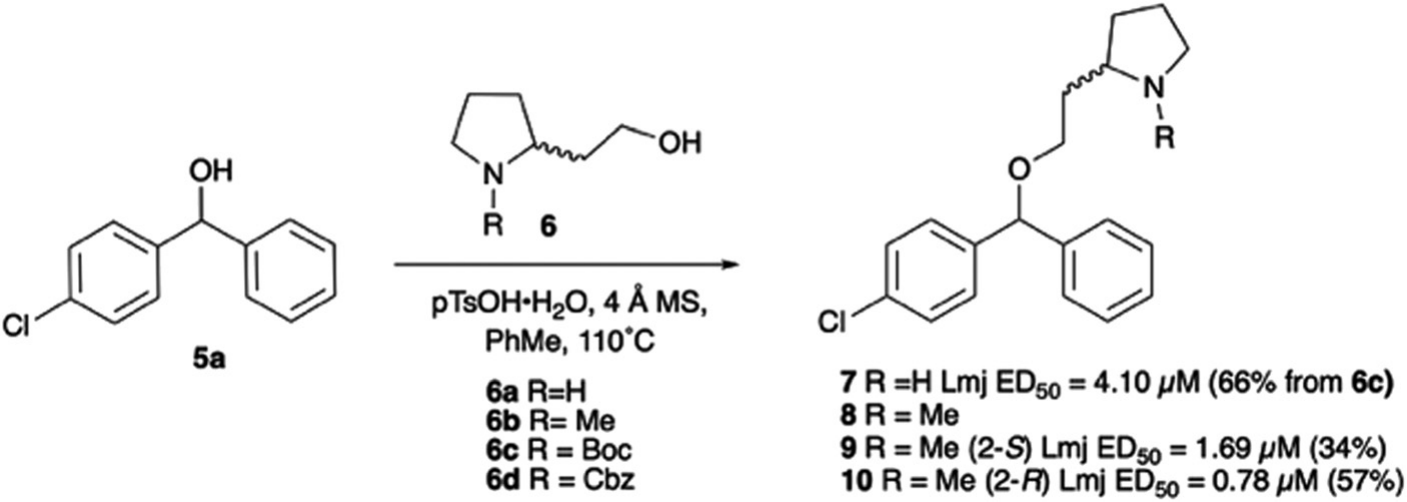


With the reported difficulties in assembling the tertiary ether in clemastine in mind, we initially explored the use of analogues lacking the quaternary methyl group to explore SAR around the diaryl carbinol, pyrrolidine head group and linker components. To verify this approach, simple protic acid (TsOH) mediated combination of diaryl carbinol 5a with 2-hydroxyethyl-*N*-methylpyrrolidine 6b provided nor-methyl clemastine 8 as a mixture of stereoisomers at the benzhydrol centre (Scheme 1).

**Scheme 2:**
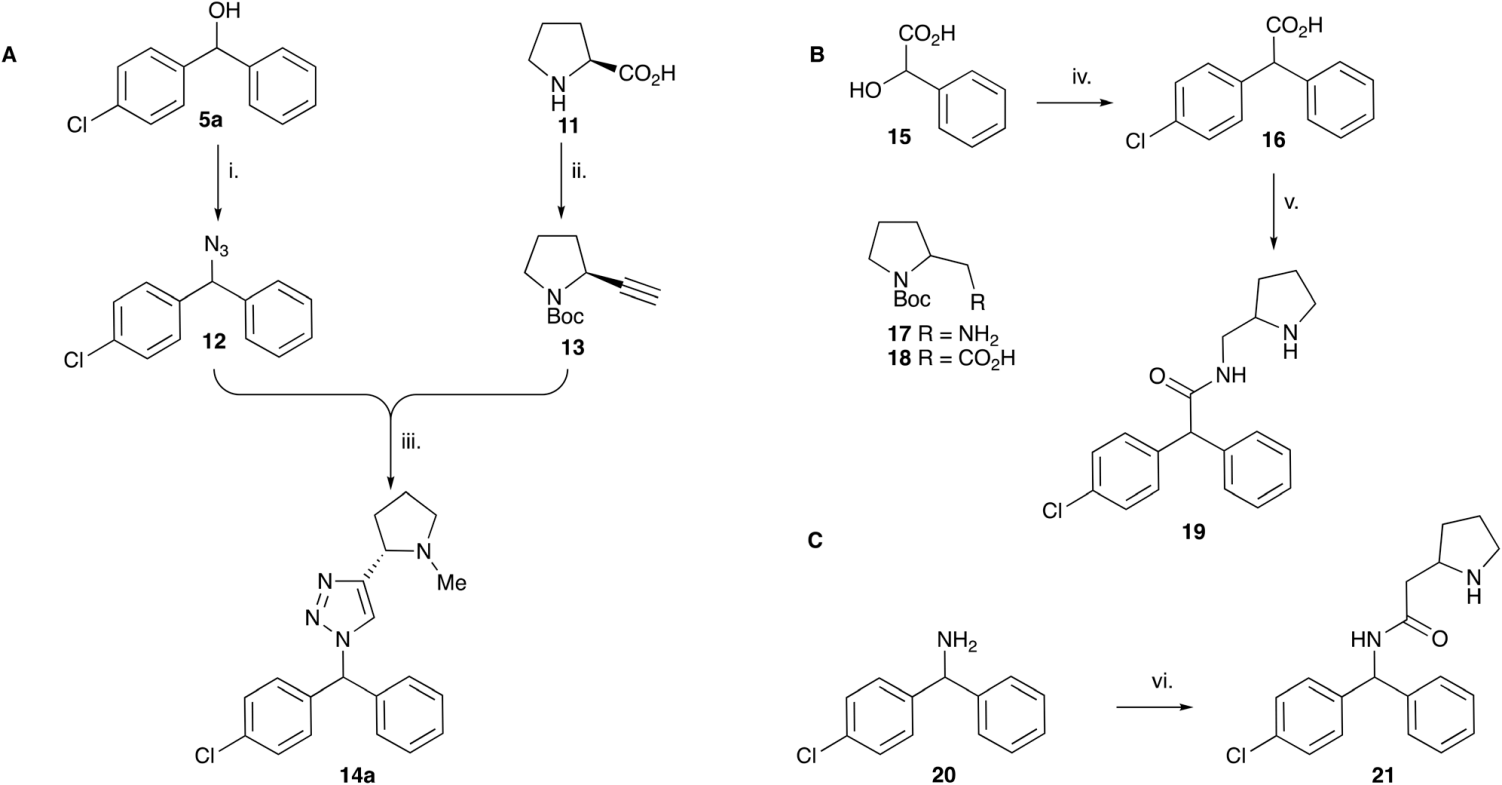
Synthesis of triazole and amide CF analogues. Reagents: **A** i. TMSN_3_, BF_3_·OEt_2_, TsOH, PhMe, 30 min (90%); ii. (a) Boc_2_O, py; (b) BH_3_·SMe_2_, THF, Δ (97%); (c) SO_3_·Py, NEt_3_, DMSO (86%); (d) PPh_3_, CBr_4_, DCM, 30 min (59%); (e) ^*n*^BuLi, THF, −78 °C, 1 h (75%); iii. (a) CuSO_4_ (0.4 equiv.), Na ascorbate (2 equiv.), MeOH, H_2_O, μW, 60 °C, 30 min (79%); (b) LiAlH_4_, Et_2_O, rt (60%). **B** iv. PhCl, SnCl_2_, Δ (40%); v. (a) 17, EDCI, NMM, HOBt, DCM, rt; (b) TFA, DCM (28%). **C** vi. (a) 18, EDCI, NMM, HOBt, DCM, rt; (b) TFA, DCM (77%).

Pleasingly employing enantiomerically pure homoprolinol derivatives afforded analogues 9 and 10 which exhibited comparable *in vitro* activity to CF 1a, *vide infra* (Scheme 1, Table S1†) with slightly higher activity observed for the *R*-stereochemistry configured pyrrolidine ring 10. Although facile, this etherification proved capricious and alternative linkers based on amide and triazole units, that enabled more efficiently SAR assessment, were explored (Scheme 2). The required precursors for each of these could be accessed commercially or synthesised in relatively few steps and the coupling reactions proved efficient enabling multiple analogues to be prepared in parallel (Table S1†).

**Fig. 2:**
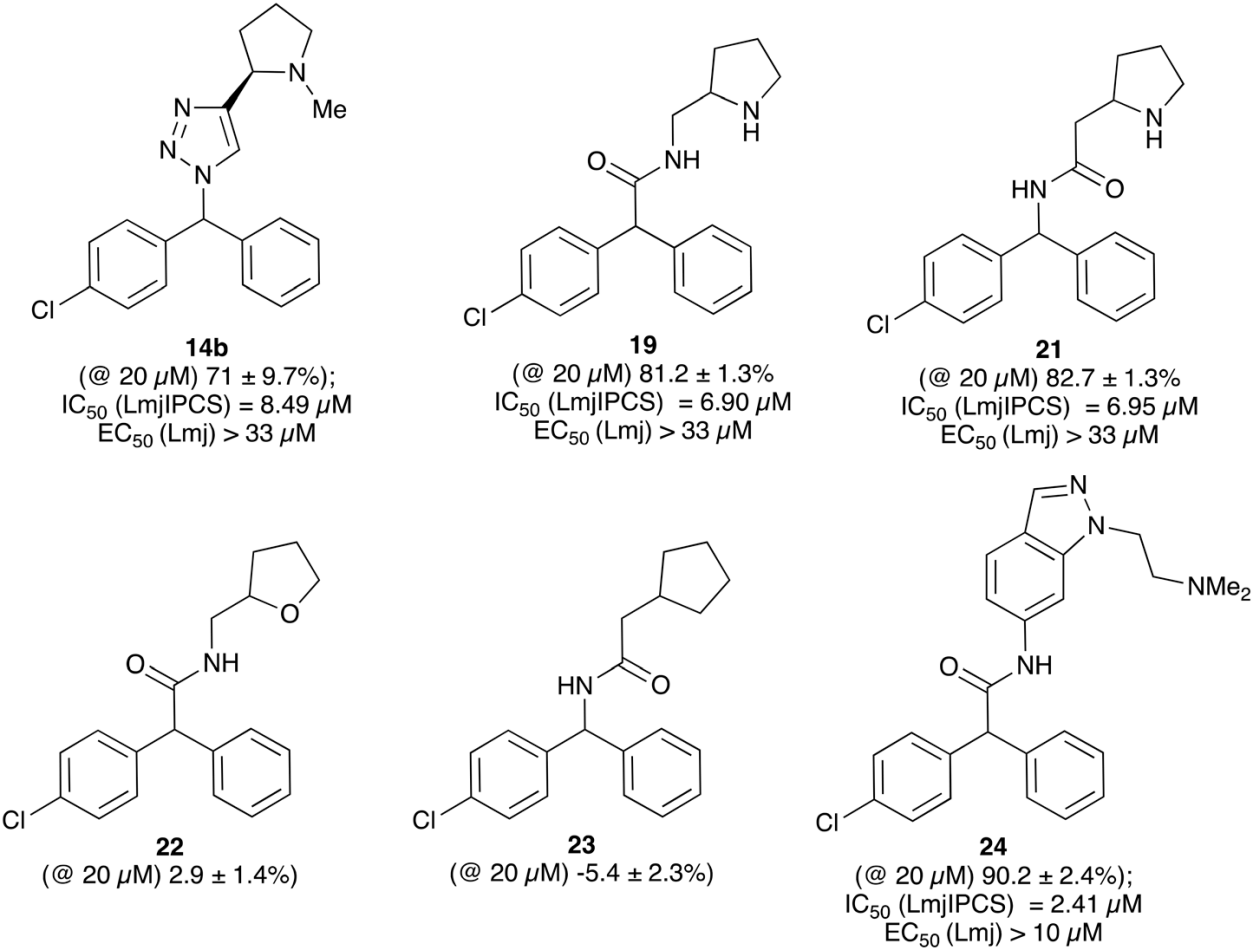
Activity of a selection of simple amide and triazole linked CF analogues against a microsomal preparation of *L. maj*IPCS and *L. major* promastigote parasites, respectively. Values in parenthesis indicate relative inhibition of the enzyme preparation at 20 μM. For further details and a full list of analogues see ESI.†

**Scheme 3:**
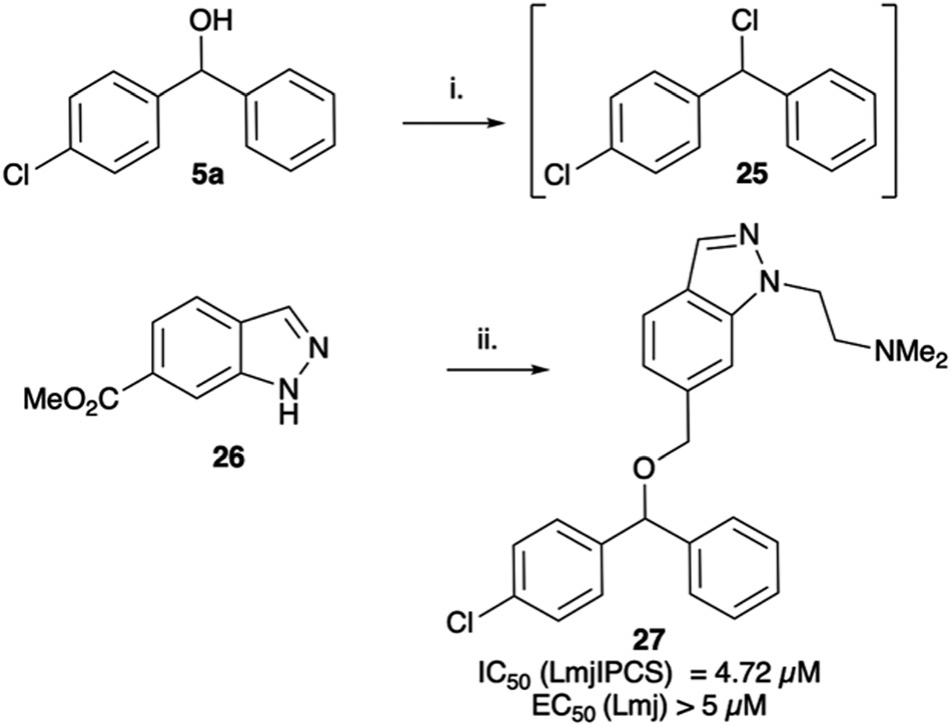
Synthesis of indazole analogue 27. i. MsCl, NEt_3_, DCM; ii. ClH·Me_2_NCH_2_CH_2_Cl, K_2_CO_3_, DMF, Δ (54%), iii. LiAlH_4_, THF (100%); iv. NaH, DMF then add 25 (14%).

When tested against microsomal enzyme preparations of *L. maj*IPCS^16^ (Fig. 2, Table S1†) the comparisons of pyrrolidine, tetrahydrofuran and cyclopentane derivatives 19, 21–23 supported earlier observations that all active compounds retained a basic nitrogen in the head group.^17^ In general, whilst enzyme inhibitory activity was retained in a number of these analogues, much lower activity was observed against promastigote parasites. The most active analogue was the *N*-dimethylaminoethyl indazole derivative 24, which exhibited equivalent levels of inhibition of *L. maj*IPCS activity as observed for CF. Speculating that this lower activity in promastigote parasite assays reflected a (membrane) transport issue, we then explored the corresponding ether derivative 27. Attempts to prepare this by acid catalysed ether formation failed and ultimately this was achieved through displacement of the corresponding benzylic chloride 25 (Scheme 3). Whilst this provided enhanced antiparasitic effects when compared to the corresponding amide 24, this was significantly lower than that observed with CF.

**Scheme 4:**
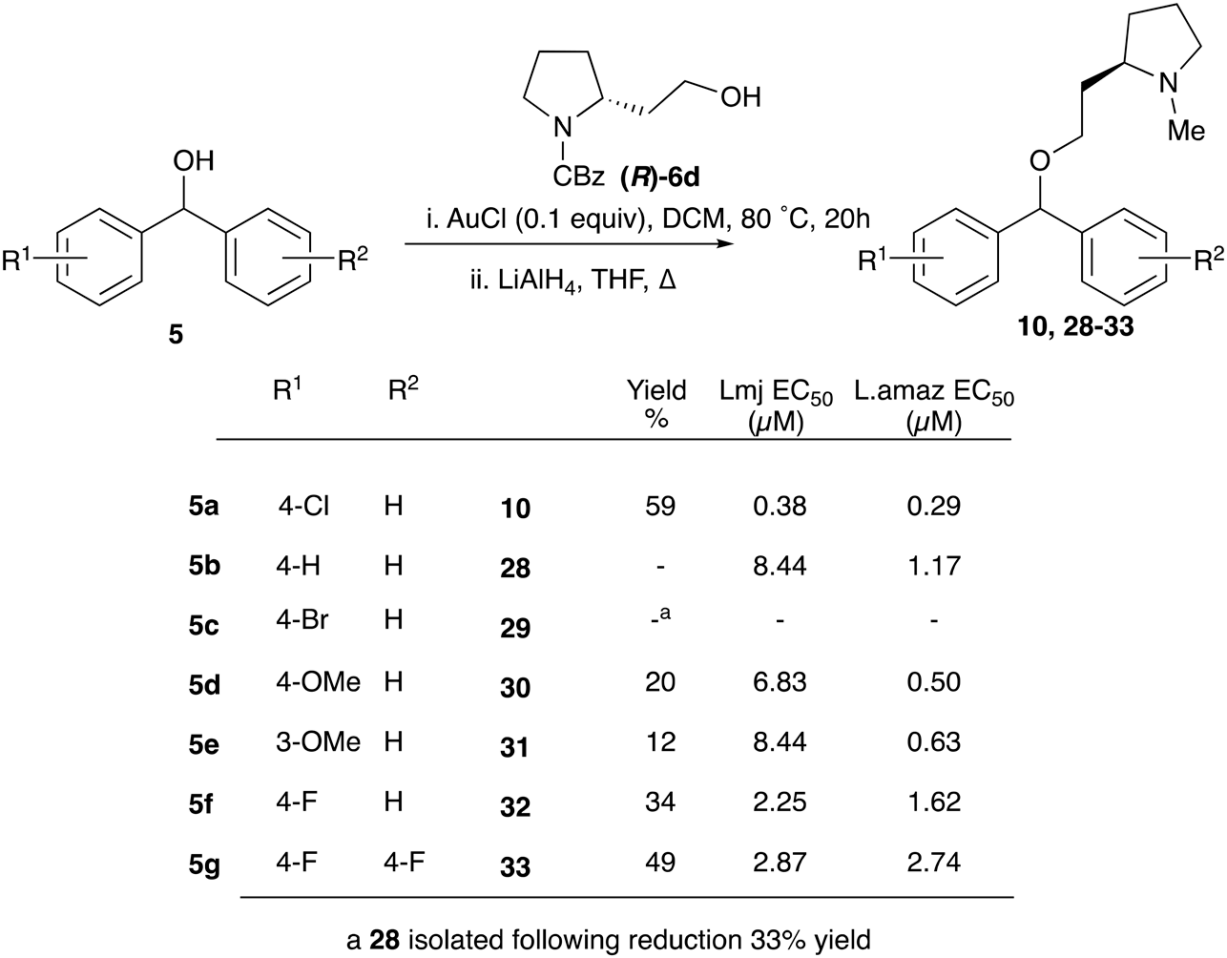
Synthesis, and anti-promastigote activity, of biarylcarbinol analogues generated through AuCl catalysed etherification.

Given the enhanced activity observed with simple ether type linkages, we then attempted to optimise the synthetic route to this connection using simple alcohol precursors. A screen of Lewis acids using 4-bromobenzhydrol and 3-phenyl-1-propanol (Fig. S1†), identified AuCl as the most efficient catalyst for this transformation. However, successful etherification with homoprolinol required *N*-Cbz protection to prevent inhibition of the reaction by the free amine. The carbamate was then reduced to form the desired clemastine analogues in a reliable fashion (Scheme 4). Although limited to functionality stable to the reduction conditions, this synthetic route provided a simple method to explore SAR around the biaryl fragments (table, Scheme 4). Disappointingly none of these derivatives displayed better antiparasitic activity than that observed for CF.

**Scheme 5:**
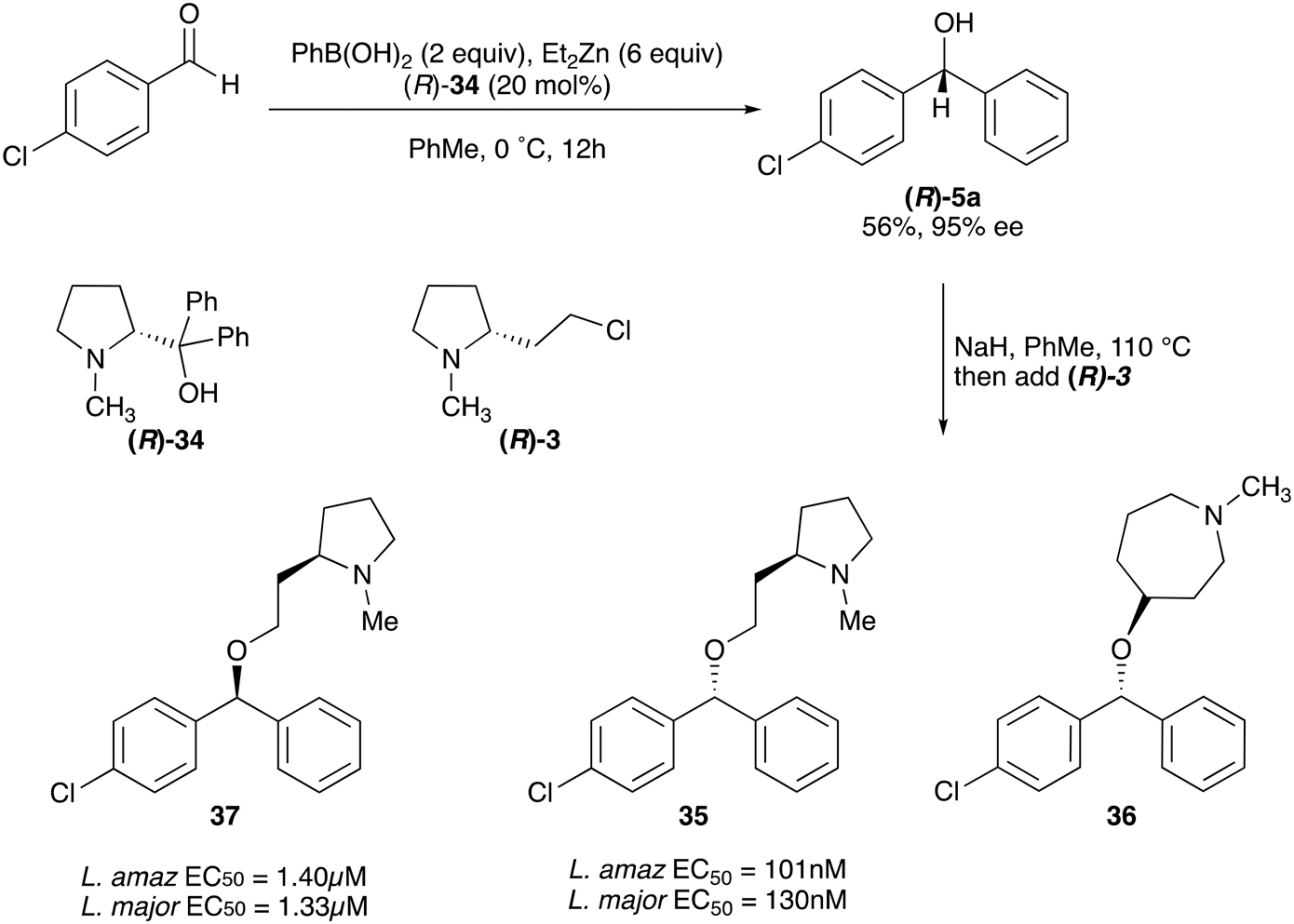
Enantioselective synthesis and anti-promastigote activity of nor methyl clemastine analogues.

Whilst nor methyl clemastine 10 retained good levels of activity in both assays this was not as effective as commercial samples of CF. Speculating that the stereochemistry at the diarylcarbinol might be crucial we then explored enantioselective strategies towards this component. Employing methodology described by Braga *et al.*,^18^ enantiomerically pure (*R*)-configured benzhydrol 5a was prepared by (*R*)-diphenylpyrrolidinemethanol 34 mediated arylation of 4-chlorobenzaldehyde (Scheme 5). In order to preserve the integrity of this chiral centre we returned to the S_N_2 methodology described by Clayden *et al.*^14^ Using the (*R*)-configured pyrrolidine-2-ethyl chloride (*R*)-3 as this had shown higher activity in earlier assays, preparation of the (*R*,*R*) analogue 35 was achieved; albeit in low yield and accompanied by the azepane by-product 36. The (*S*,*R*) analogue 37 could similarly be prepared either by arylating benzaldehyde with 4-ClC_6_H_4_B(OH)_2_ or using the enantiomeric chiral auxiliary (*S*)-34. Testing against both *L. major* and *L. amazonensis* (*L. amaz*) promastigotes revealed that optimal activity resided in the (*R*,*R*) diastereoisomer 35 which showed comparable activity to CF in both enzyme and parasite assays.

Whilst good levels of activity could be realised, the synthetic challenge remained, particularly that caused by ring expansion during alkylation with the proline-derived alkylating agents. To address this, we explored a simple positional shift in the amine component. These compounds could be generated by AuCl ether formation with a bromoalcohol followed by S_N_2 alkylation with a proline derived amine. As before the stereochemistry of the pyrrolidine component had minimal effect (Table 1 entries 1 & 2). Replacing the 2-methyl substituent with a longer functionalised sidechain was deleterious to activity and an unsubstituted pyrrolidine showed only modest effects (Table 1 entries 3–6). Despite the simplicity of the synthetic chemistry these initial compounds had only moderate efficacy when compared with CF. Speculating that the position of the basic nitrogen relative to the benzhydryl centre was important we then prepared examples with 2, 3 and 4 carbon linkers (Table 1, entries 1,7, 8). Consistent with this hypothesis, the 3-carbon linker analogues, which have an equivalent spacing between heteroatoms as that found in CF, afforded optimal anti-parasitic activity.

**Table 1:** Preparation and antileishmanial activity of N-linked analogues of CF

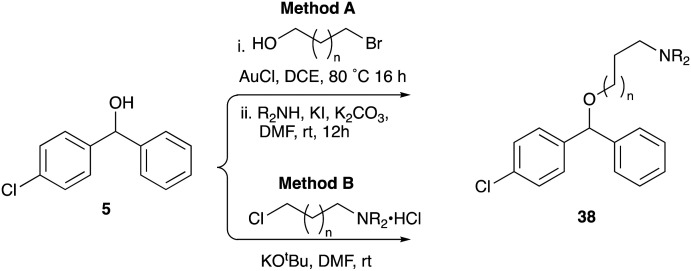								
Entry	38	Carbinol stereochemistry	NR_2_	n	Method yield (%)	L.mj EC_50_^a^ (μM)	L.amaz EC_50_^a^ (μM)	BMDM CC^50^ (μM)
1	**a**	RS	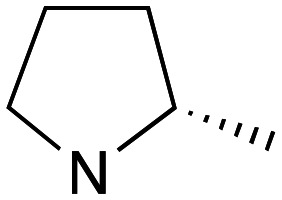	0	**A** (54%)	2.05	3.40	—
2	**b**	RS	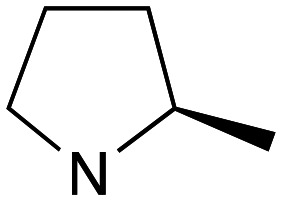	0	**A** (57%)	2.65	4.10	—
3	**c**	RS	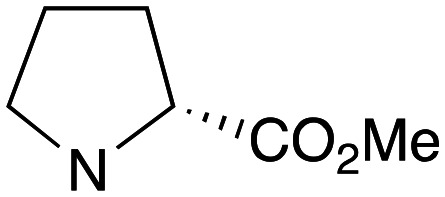	0	**A** (26%)	23.83	17.39	—
4	**d**	RS	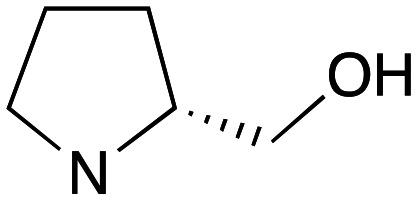	0	**A** b	5.88	3.20	—
5	**e**	RS	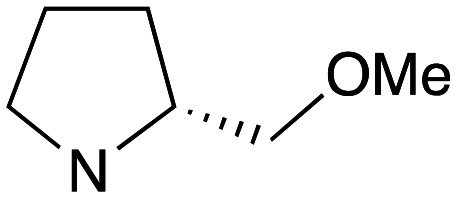	0	**A** (57%)	14.41	12.35	—
6	**f**	RS	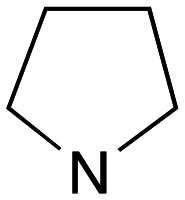	0	**A** (47%)	—	3.27	57
7	**g**	RS	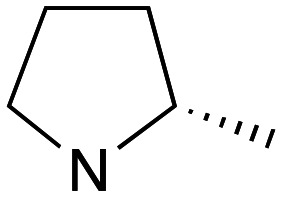	2	**A** (40%)	1.21	—	—
8	**h**	RS	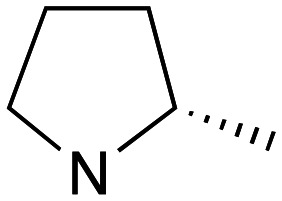	1	**A** (57%)	0.09	—	—
9	**i**	S	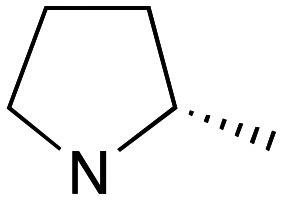	0	**B** (55%)	7.05	—	—
10	**j**	S	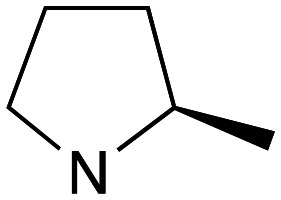	0	**B** (57%)	11.5	—	—
11	**k**	R	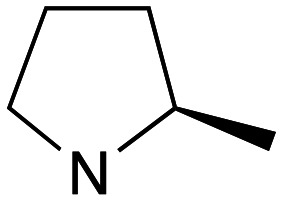	0	**A** (61%)	1.7	3.2	—
12	**l**	R	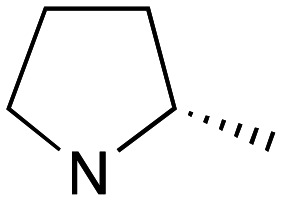	1	**B** (55%)	0.030	0.041	73
13	**m**	R	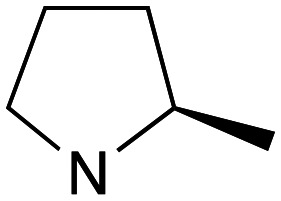	1	**B** (42%)	0.031	0.042	—
14	**n**	R	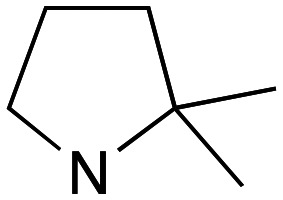	1	**B** (44%)	0.027	0.017	—
15	**o**	R	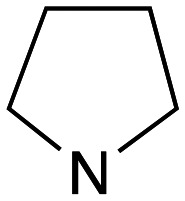	1	**B** (42%)	0.08	0.10	—

a EC_50_ determined using promastigote parasites – see ESI† for details.

b Obtained in 65% yield after reduction of 38c with LiAlH_4_, THF.

Whilst this enabled easy assessment of SAR this came at the cost of stereochemical insights. Consequently, and in contrast to the CF head group, direct alkylation of the diaryl carbinol with a preformed N-linked alkyl halide proved viable yielding a single enantiomer ether product in good yields with no evidence for ring expanded side products. Whilst alkylations of 10b, using preformed alkoxide in toluene, were susceptible to rapid oxidation to the benzophenone, presumably caused by trace amounts of oxygen in the reaction mixture, this issue could be circumvented by switching to a more polar solvent (DMF) and *in situ* deprotonation and alkylation. As before the stereochemistry of the diaryl carbinol was crucial with higher activity observed in the *R*-configured series of compounds (Table 1, entries 9–11, 8–13). As before, the presence of a 2-substituted pyrrolidine was desirable although the stereochemistry had minimal impact with a gem dimethyl variant proving equally effective in these assays. As the simple 2-methylpyrrolidines were more accessible, we arbitrarily selected the *S* isomer for all further analyses.

**Fig. 3:**
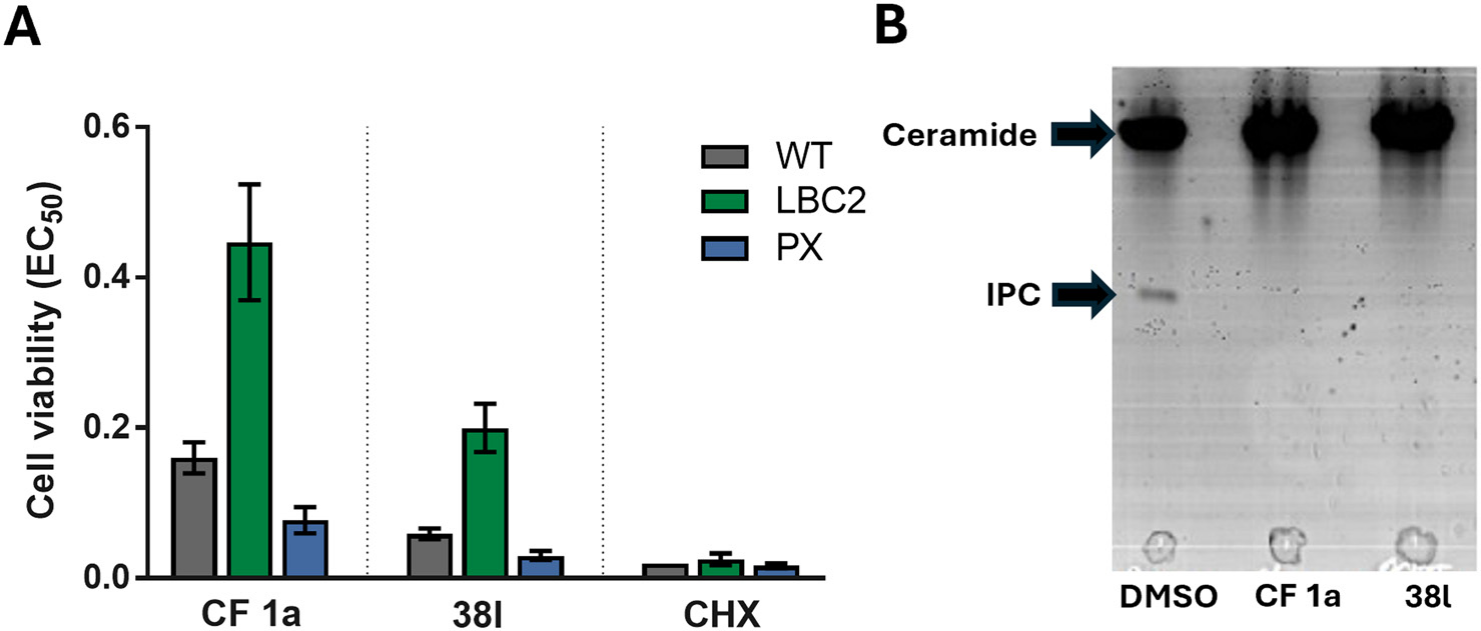
On-target assays for analogue 38l. A EC_50_ values of CF 1a and analogue 38l against WT, Δ LCB2 and PX *L. major* promastigotes; cycloheximide (CHX) was used as a positive control; values are presented as mean ± 95% CI from at least three experiments. B Separation of NBD-C_6_-IPC 81 product from NBD-C_6_-ceramide 80 substrate by HPTLC; microsomes were treated with 5 mM of analogue 38l and negative and positive controls were DMSO and 5 mM of CF 1a, respectively.

CF was originally selected from a screen for inhibitors of the sphingolipid synthase, (*L. maj*IPCS) and to verify that this new analogue retained this profile we applied the same assays as used before. As described previously, the dose-dependent growth inhibition of *L. major* wildtype (WT) and mutant Δ LCB2, in which *L. maj*IPCS is redundant, was assessed and compared to validate the on target effects of N-linked analogue 38l (Fig. 3A). Using cycloheximide as a control these assays showed that, similar to CF 1a, analogue 38l is approximately 3 times more active against WT than the mutant Δ LCB2 promastigotes. Sensitivity was restored in both series in the add-back cell line (PX) suggesting that both CF 1a and N-linked analogue 38l are disrupting the sphingolipid pathway. To support inhibition of the IPCS, the HPTLC biochemical assay described by Mina *et al.*^16^ was employed. In this, using a microsomal preparation of the *L. maj*IPCS, a fluorescent substrate NBD-C_6_-ceramide was combined with PI and the two drugs. Following the reaction, the product, NBD-C_6_-IPC, and residual starting material were separated *via* HPTLC and analysed by fluorescence spectroscopy. No product was observed in either of the compound treated reactions demonstrating that 38l was a functional analogue of CF 1a, acting as an inhibitor of IPCS, (Fig. 3B).

In order to retain comparisons with CF we then evaluated activity in an intramacrophage model of *L. amazonensis*. Pleasingly, comparable levels of antiparasitic activity (0.51 ± 14 μM) were retained with the enhanced benefit of a lower toxicity against the host bone-marrow derived macrophage (CC_50_ = 73 ± 14 μM; SI ∼140). The significantly enhanced selectivity index (SI) was encouraging. It is known that, as an early H_1_ antagonist, CF was not particularly selective with reported activity against other receptors notably the muscarinic receptors (M1, M2, M3 and M5).^19–21^ Given the lower cytotoxicity it was of interest to explore whether this simple structural modification had changed other activities. Accordingly, CF, and analogues (*R*,*R*)-35 and 38l were assessed in a simple radioligand displacement assay. CF and the simple nor-methyl analogue (*R*,*R*)-35 showed very similar binding profiles with activity against a broad representation of receptor sub-types. However, the N linked analogue 38l showed greater selectivity with effective binding (>40%) observed for only the H1, M1, and M4 receptors and lower affinity to the M5 and 5-HTB2 receptors (Fig. S2†).

**Fig. 4:**
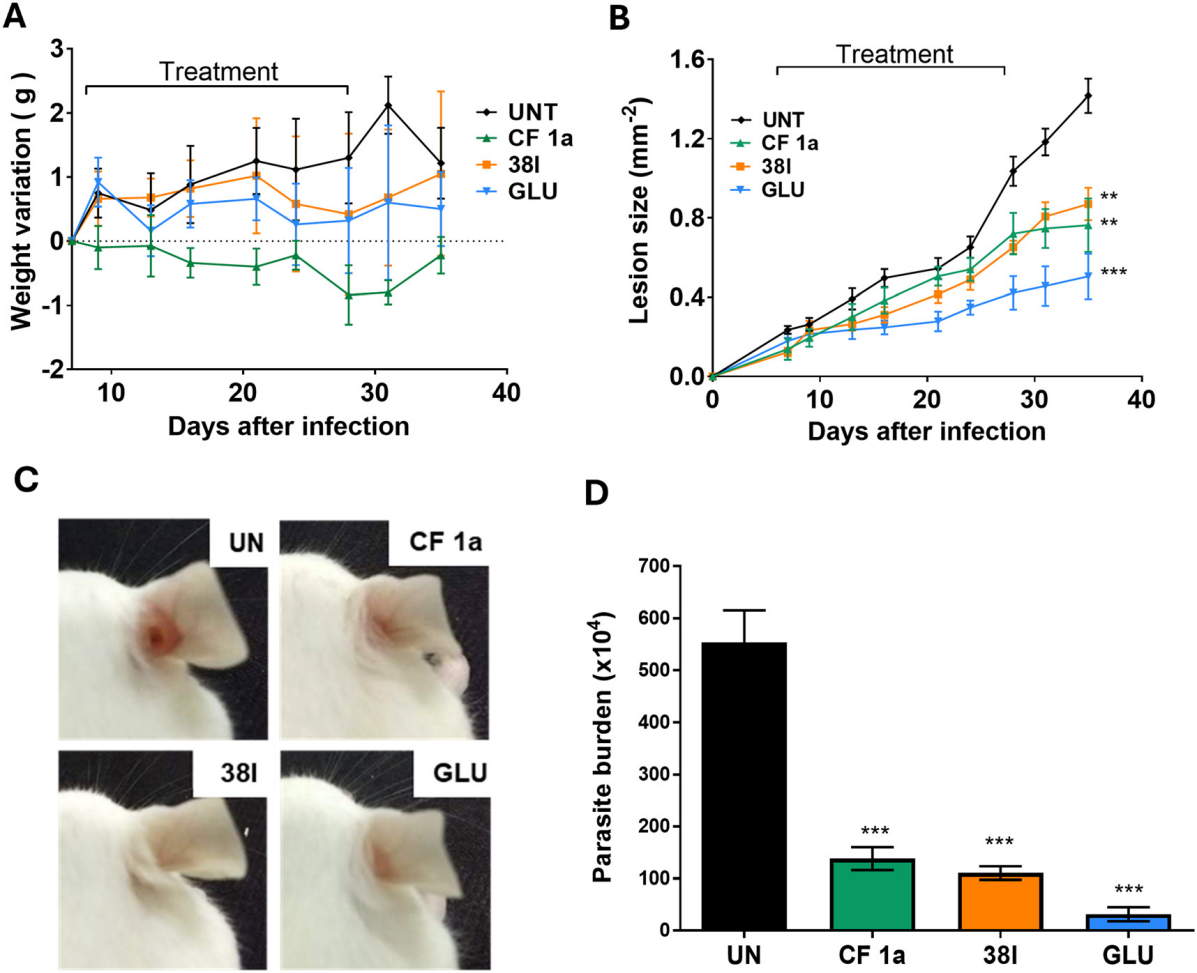
*In vivo* assessment of 38l BALB/c mice, randomized into drug-treated and control groups of 5/6 mice each, were infected with 2 × 10^6^*L. amazonensis* GFP promastigotes in the right ear and treated with the stated drug for 28 days (UNT: untreated control; CLEM: **CF**1a; 38l; GLU: Glucantime). A Weight variation of mice during 28 days of treatment. B Progression of lesion thickness over 28 day treatment period. C Representative photographs of the infected ear for each group taken after completion of treatment, D parasite burden was evaluated by LDA on 36 days post infection. Asterisks indicate that the difference between control and drug-treated groups are statistically significant. **P ≤ 0.01, ***P ≤ 0.001.

Finally, with good levels of intramacrophage activity and low cellular toxicity in cell culture, efficacy in a mouse model of *L. amazonensis* infection was assessed. In our earlier studies we were unable to deliver CF to the mouse orally, an observation we attributed to the high rate of metabolism and low kinetic solubility of the parent drug in this model. As analysis of 38l suggested that the physicochemical parameters were unsurprisingly similar to clemastine (38l clog *P* 4.25, log *S* −5.16; 1 clog *P* 4.18, log *S* −5.12)^22^ it was likely that these challenges remained. Consequently, we used IL dosing for this experiment. To enable direct comparison with CF, the same treatment regimen (dose of 1.2 mg kg^−1^*via* IL injection twice a week for 4 weeks starting on DPI) was used for both compounds. Whilst CF-treated mice showed a small weight loss, all other groups; trial and positive and negative controls; showed little variation in the weight of mice (Fig. 4A). Analysis of the mice 35 days post-infection revealed that lesion growth trends were similar for the groups treated with analogue 38l and CF 1a*via* IL administration (Fig. 4B). Overall, both treatments showed a statistically significant reduction in parasitaemia when compared to the untreated group (Fig. 4D).

## Discussion

Sphingolipids are essential components of eukaryotic cell membranes having critical roles in a variety of cell processes including signal transduction, intracellular membrane trafficking and the regulation of cell growth and survival. The *de novo* biosynthesis of these lipid species is highly conserved across the various kingdoms and, as a result, selective inhibitors of the enzymes within this pathway are relatively rare.^23^ However, a divergence appears in the synthesis of complex sphingolipids. Whilst *Leishmania* spp. synthesise inositol phosphorylceramide (IPC) as their primary phosphosphingolipid, through the action of inositol phosphorylceramide synthase (IPCS), mammals use sphingomyelin synthase (SMS) to generate sphingomyelin. Whilst SMS employs phosphatidylcholine, IPCS catalyses the transfer of the phosphorylinositol group from phosphatidylinositol (PI) to ceramide with the concomitant release of diacylglycerol (DAG). Consequently, in addition to producing the complex sphingolipid required for membrane integrity this reaction is also important in maintaining homeostasis in the levels of the key signaling components ceramide and DAG. Since the former is pro-apoptotic and the latter mitogenic, modulating the activity of this enzyme can have catastrophic effects on cell function and survival.

Whilst the fungal enzyme catalysing this reaction, known as AUR1p, has long been characterised as essential and a target for antifungals, studies in other kingdoms are less well established. In recent work we identified the leishmanial orthologue and used a microsomal preparation to screen a library of compounds for inhibitory activity. This led to the discovery of the histamine H_1_ antagonist CF as a potential antileishmanial. Although exhibiting good activity in a mouse model of infection, CF is challenging to further optimise as reported synthetic routes are non-trivial requiring the enantioselective formation of a quaternary benzhydryl carbon centre and its subsequent alkylation with a proline derived alkyl halide which is prone to isomerisation.^14,24^

In this report, we have described a solution to both these problems, enabling the rapid assembly of a clemastine analogue 38l that retains the antileishmanial activity of CF but with lower host cell toxicity. Intriguingly this lower toxicity is mirrored by an enhancement in the selectivity of 38l in binding to other neurotransmitter receptor subtypes. Given that CF has other activities, including a suppression of the host immune system,^19,25^ this relationship may be coincidental and requires further study. With good antileishmanial activity in an intramacrophage amastigote assay, analogue 38l was progressed into an *in vivo* infection study. Whilst CF is an oral drug, efforts to administer both CF and 38l to mice in this fashion were not successful. However, IL administration showed that 38l has comparable efficacy to CF and is, therefore, a potential therapy against CL.

Future work will now focus on the generation of analogues to further enhance activity and selectivity as well as to address the delivery challenges. These studies will be greatly simplified using the alternative structural design developed in this work. The outcomes of these efforts together with use of novel formulation technologies designed to enhance oral bioavailability are in progress and will be reported in due course.

## Ethical statement

All mice used in the experiments were maintained under controlled temperature, filtered air and water, autoclave bedding, and commercial food at the animal facilities at Federal University of Rio de Janeiro.

The animal protocols for this study were approved by the Federal University of Rio de Janeiro Institutional Animal Care and Use Committee under the number 030/17. The research was conducted in compliance with the principles stated in the *Guide for the Care and Use of Laboratory Animals* (NIH).^26^

## Data availability

The data supporting this article have been included as part of the ESI.†

## Author contributions

PGS, BRB and PWD conceived and designed the project. PGS, BRB, PWD and AM supervised the project. RLC, DOE, CB, VSA, EP, NT and TA designed and conducted the experiments and collected the data. RLC, DOE, BRB, PWD and PGS analysed the results. RLC and PGS drafted the manuscript. All authors read, reviewed, and approved the final draft.

## Conflicts of interest

There are no conflicts of interest to declare.

## Supplementary Material

MD-016-D4MD01004C-s001
