# Supplementary material for: Simple accessible clemastine fumarate analogues as effective antileishmanials
Source: RSC Med Chem. 2025 Jan 31;16(4):1686–94. doi: 10.1039/d4md01004c (PMC11862611; doi:10.1039/d4md01004c)

Supplementary Information (SI) for RSC Medicinal Chemistry.
This journal is © The Royal Society of Chemistry 2025

Simple Accessible Clemastine Fumarate Analogues as Effective Antileishmanials

Rebecca L. Charlton,[1,2] Douglas O. Escrivani,[1,2] Christopher Brown,[1] Niranjan Thota,[1] Victor S. Agostino,[1] Exequiel O. J. Porta,[1] Timur Avkiran,[3] Andy Merritt,[3] Paul W. Denny,[4] Bartira Rossi-Bergmann,[2] Patrick G. Steel*[1]

[1] *Department of Chemistry, Durham University, Lower Mountjoy, South Rd, Durham DH1 3LE, UK*

[2] *Institute of Biophysics, Carlos Chagas Filho, Universidade Federal do Rio de Janeiro, 21941-902 Rio de Janeiro – RJ, Brazil*

[3] *LifeArc, Accelerator Building, Open Innovation Campus, Stevenage, SG1 2FX, UK*

[4] *Department of Biosciences, Durham University, Lower Mountjoy, South Rd, Durham DH1 3LE, UK*

Supporting Information

1. Supplementary Table S1: Assay data for compounds prepared in this study **2**
2. Supplementary Figure S1. Lewis acid screen for biarylcarbinol ether formation **13**
3. Supplementary Figure S2: Relative selectivity of Clemastine fumarate **1a** and analogues **(R,R)-35** and **38l** **14**
4. Experimental Procedures **15**
   4.1. Synthetic Chemistry Protocols **15**
   4.2. Biological Methods **76**
5. References **81**
6. ^{1}H and ^{13}C NMR Spectra for Compounds assayed in this Study **82**

Supplementary Table S1: Assay data for compounds prepared in this study

Nº	Structure	*Lmj*IPCS inhibition % at 20 µM ± SD	*Lmj*IPCS IC_{50} (µM)	*L. major* promastigote EC_{50} (µM)	*L. amaz promastigote* EC_{50} (µM)	*BMDM* CC_{50} *(µM)*
**1a**[a]	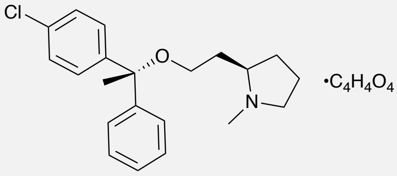<br>Clemastine Fumarate	82.6 ± 9.3	2.87	0.179	0.027	19.13
**7**	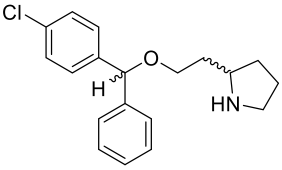	78.1 ± 12.5	6.26	4.10	_	_
**9**	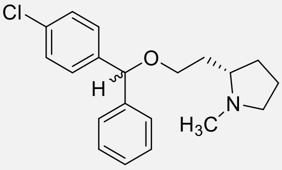	81.2 ± 10.6	4.45	1.69	_	_
**10**	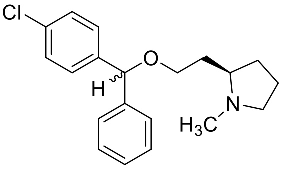	78.1 ± 9.7	4.13	0.78	_	_
**14a**	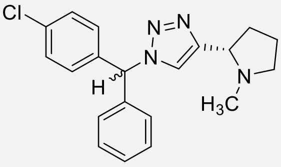	60.6 ± 9.2	14.52	>33	_	_
**14b**	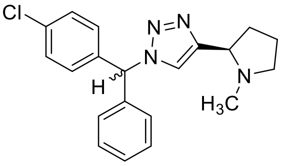	71.0 ± 9.7	8.49	>33	_	_
**19**	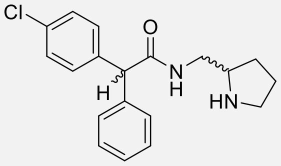	81.2 ± 1.3	6.90	>33	_	_

**21**	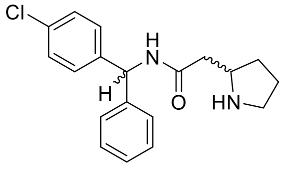	82.7	6.95	>33	_	_
**22**	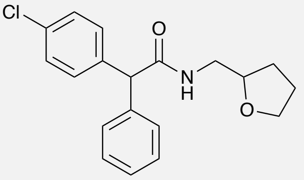	2.9 ± 1.4	-	_	_	_
**23**	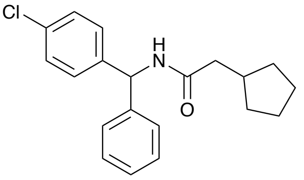	-5.4 ± 2.3	-	_	_	_
**24**	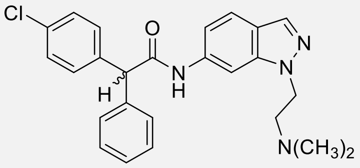	90.2 ± 2.4	2.41	>10	_	_
**27**	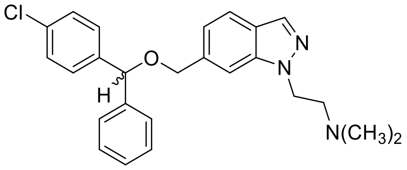	–	4.72	>5	_	_
**28**	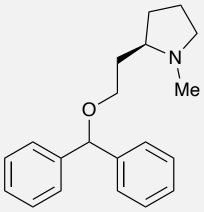	–	_	8.44	1.17	_
**29**	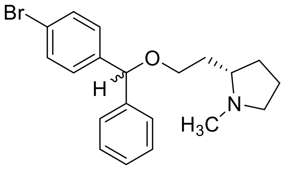	–	3.21	1.36	_	_
**30**	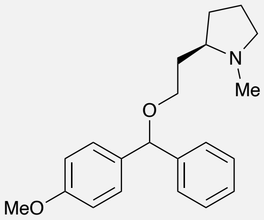	_	_	6.83	0.50	_

**31**	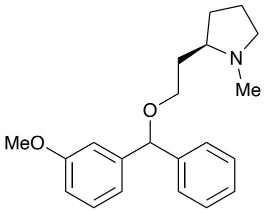	_	_	8.44	0.63	_
**32**	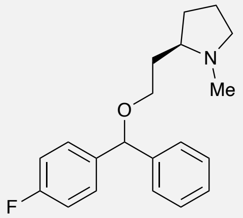	_	_	2.25	1.62	_
**33**	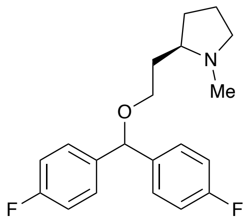	_	_	2.87	2.74	_
**35**	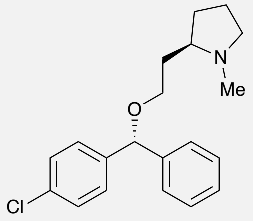	_	_	0.13	0.101	_
**37**	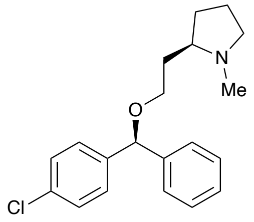	_	_	1.33	1.40	_
**38a**	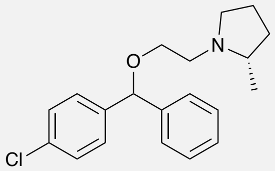	_	_	2.05	3.40	_
**38b**	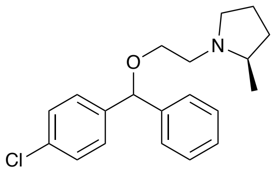	_	_	2.65	4.10	_
**38c**	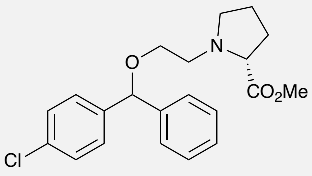	_	_	23.83	17.39	_

**38d**	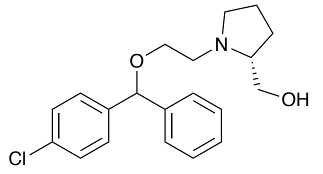	–	–	5.88	3.20	–
**38e**	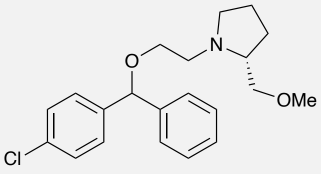	–	–	14.41	12.35	–
**38f**	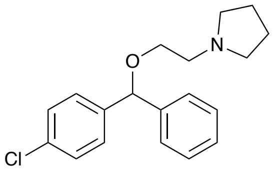	–	–	–	3.27	57
**38g**	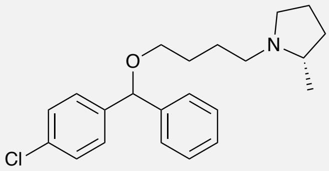	–	–	1.21	–	–
**38h**	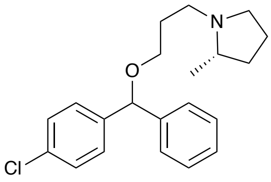	–	–	0.09	–	–
**38i**	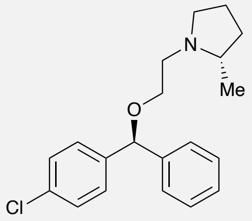	–	–	7.05	–	–
**38j**	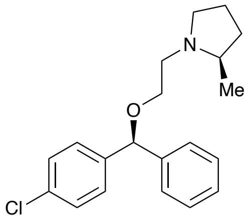	–	–	11.5	–	–
**38k**	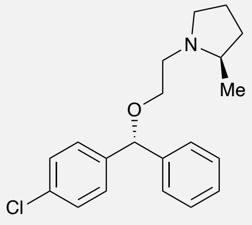	–	–	1.7	3.2	–

**38l**	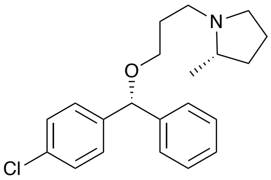	_	_	0.030	0.041	73
**38m**	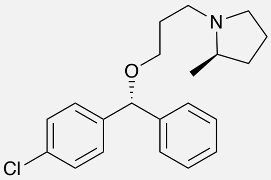	_	_	0.031	0.042	_
**38n**	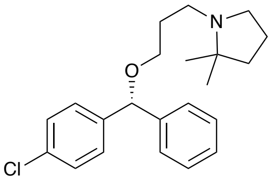	_	_	0.027	0.017	_
**38o**	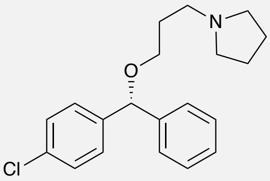	_	_	0.08	0.10	_
**S1**	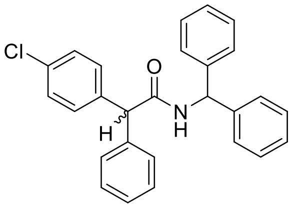	4.7 ± 2.5	–	–	_	_
**S2**	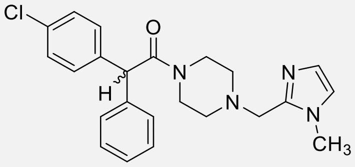	8.6 ± 2.1	–	–	_	_
**S3**	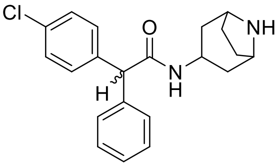	89.2 ± 2.6	5.40	–	_	_
**S4**	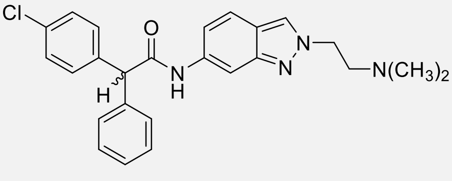	90.2 ± 1.7	4.37	–	_	_
**S5**	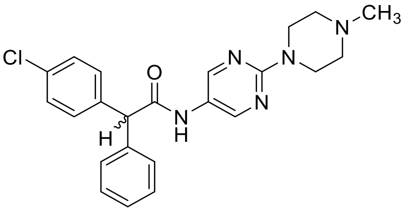	89.6 ± 2.2	6.11	–	_	_

**S6**	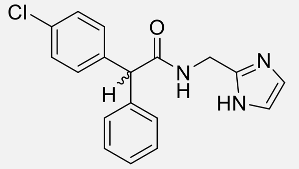	53.4 ± 8.5	23.89	–	_	_
**S7**	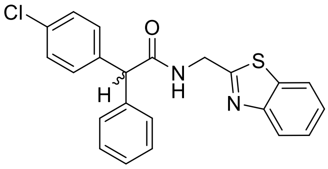	79.4 ± 9.0	36.21	–	_	_
**S8**	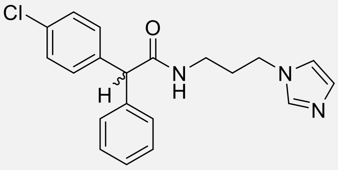	28.3 ± 5.3	–	–	_	_
**S9**	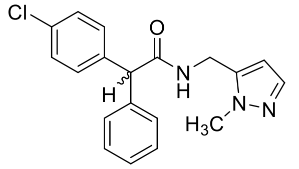	2.5 ± 7.8	–	–	_	_
**S10**	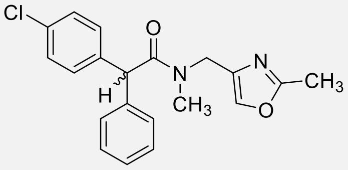	0.6 ± 1.8	–	–	_	_
**S11**	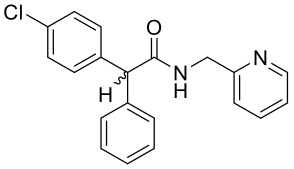	2.7 ± 1.7	–	–	_	_
**S12**	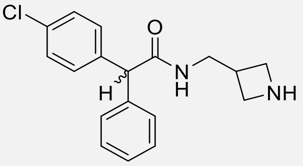	86.0 ± 2.7	13.09	–	_	_
**S13**	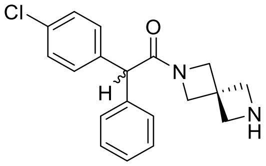	77.9 ± 5.1	11.38	–	_	_
**S14**	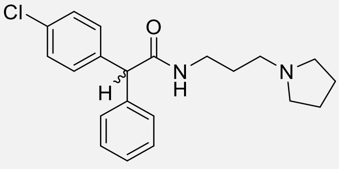	76.1 ± 8.5	20.49	–	_	_

**S15**	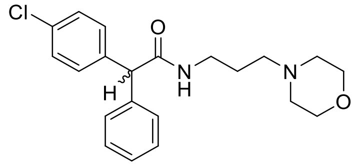	35.8 ± 3.9	–	–	_	_
**S16**	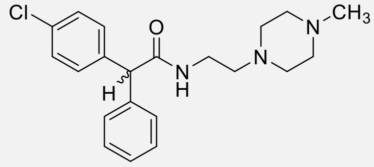	16.3 ± 3.8	–	–	_	_
**S17**	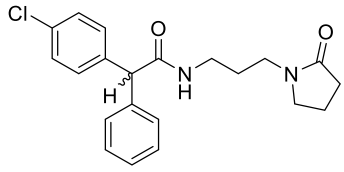	−0.3 ± 0.8	–	–	_	_
**S18**	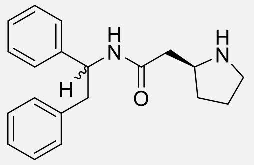	44.4 ± 4.9	24.86	–	_	_
**S19**	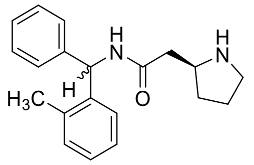	89.4 ± 4.1	18.94	–	_	_
**S20**	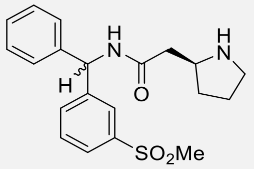	11.3 ± 7.4	–	–	_	_
**S21**	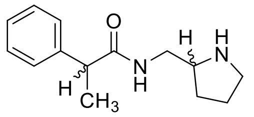	−1.1 ± 2.5	–	–	_	_
**S22**	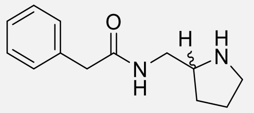	1.7 ± 3.4	–	–	_	_
**S23**	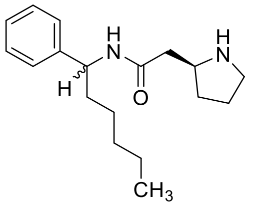	81.9 ± 2.8	12.67	–	_	_
**S24**	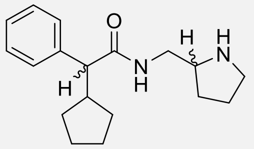	46.4 ± 5.8	28.31	–	_	_

**S25**	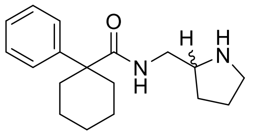	24.7 ± 7.5	–	–	_	_
**S26**	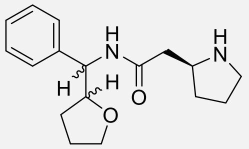	7.3 ± 1.9	–	–	_	_
**S27**	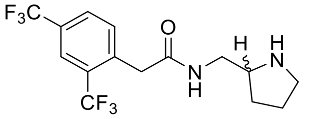	57.2 ± 10.9	16.51	–	_	_
**S28**	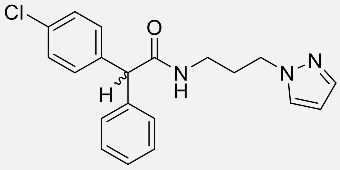	7.5 ± 7.3	–	–	_	_
**S29**	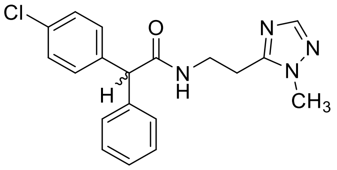	2.4 ±1.7	–	–	_	_
**S30**	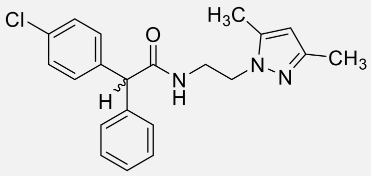	7.3 ± 6.5	–	–	_	_
**S31**	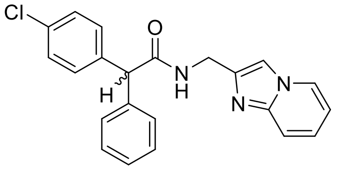	24.4 ± 3.3	–	–	_	_
**S32**	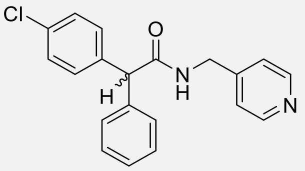	0.5 ± 2.1	–	–	_	_
**S33**	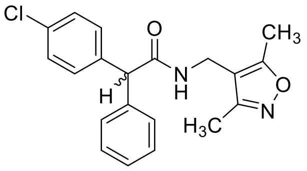	12.0 ± 3.1	–	–	_	_
**S34**	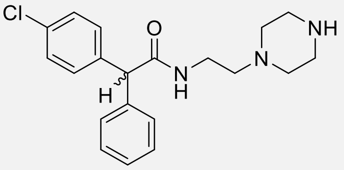	27.5 ± 8.3	–	–	_	_

**S35**	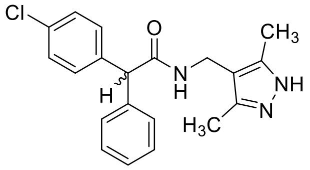	4.3 ± 1.8	–	–	_	_
**S36**	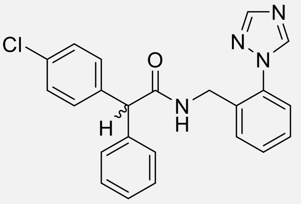	4.3 ± 3.1	–	–	_	_
**S37**	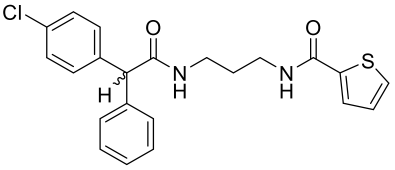	9.0 ± 2.1	–	–	_	_
**S38**	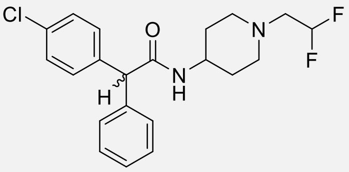	3.5 ± 3.1	–	–	_	_
**S39**	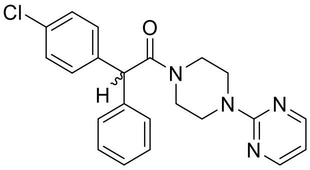	−1.7 ± 5.3	–	–	_	_
**S40**	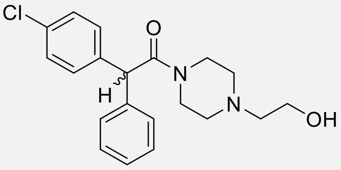	16.0 ± 0.6	–	–	_	_
**S41**	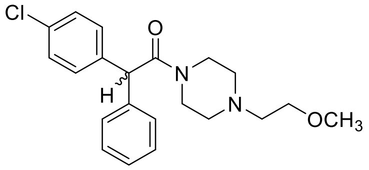	9.3 ± 1.8	–	–	_	_
**S42**	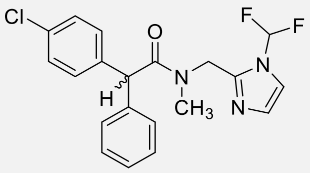	8.5 ± 1.2	–	–	_	_
**S43**	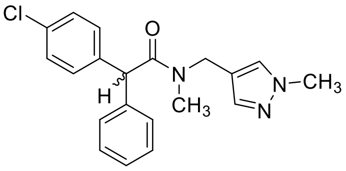	−3.2 ± 1.7	–	–	_	_

**S44**	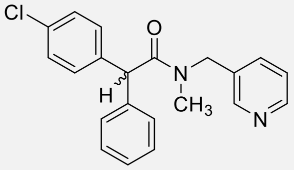	8.0 ± 1.2	–	–	_	_
**S45**	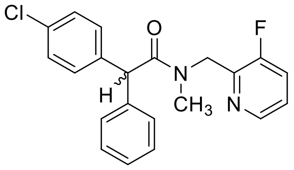	5.1 ± 1.5	–	–	_	_
**S46**	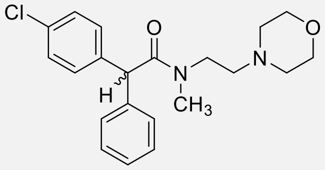	5.5 ± 3.2	–	–	_	_
**S47**	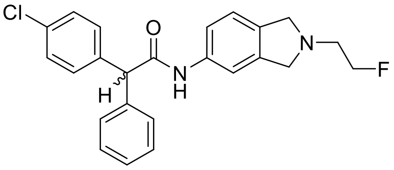	59.8 ± 29.9	17.07	–	_	_
**S48**	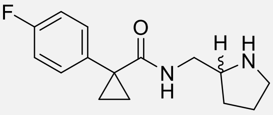	0.4 ± 5.3	–	–	_	_
**S49**	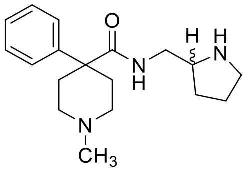	26.5 ± 12.1	–	–	_	_
**S50**	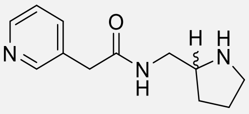	−1.8 ± 6.4	–	–	_	_
**S51**	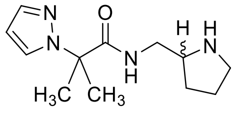	−1.5 ± 4.2	–	–	_	_
**S52**	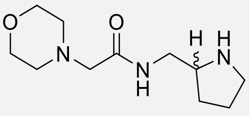	−1.1 ± 8.2	–	–	_	_
**S53**	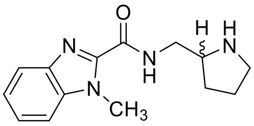	0.9 ± 5.3	–	–	_	_

**S54**	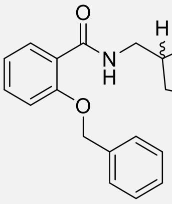	40.6 ± 15.7	–	–	_	_
**S55**	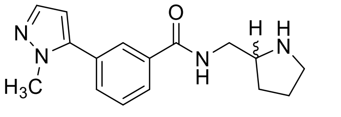	−2.1 ± 2.7	–	–	_	_
**S56**	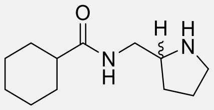	−1.3 ± 3.7	–	–	–	–
**S57**	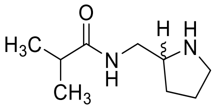	−4.9 ± 3.6	–	–	–	–
**S58**	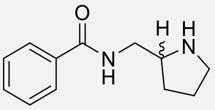	−0.7 ± 2.4	–	–	–	–

a. data from Mina *et al.*[1]; – Value not determined

Figure S1. Lewis acid screen for biarylcarbinol ether formation

Following General procedure C, eight Lewis acids were assessed using benzhydrol and 3-phenyl-1-propanol as model substrates (Scheme). The yields were estimated using integrals from GC-MS analysis with dodecane as the standard. The standardised data (arbitrarily setting the output for $CuCl_2$ to equal 1) is shown in the chart below.

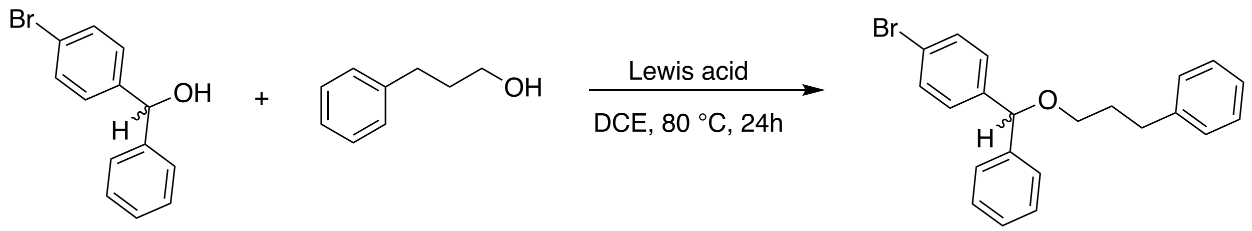

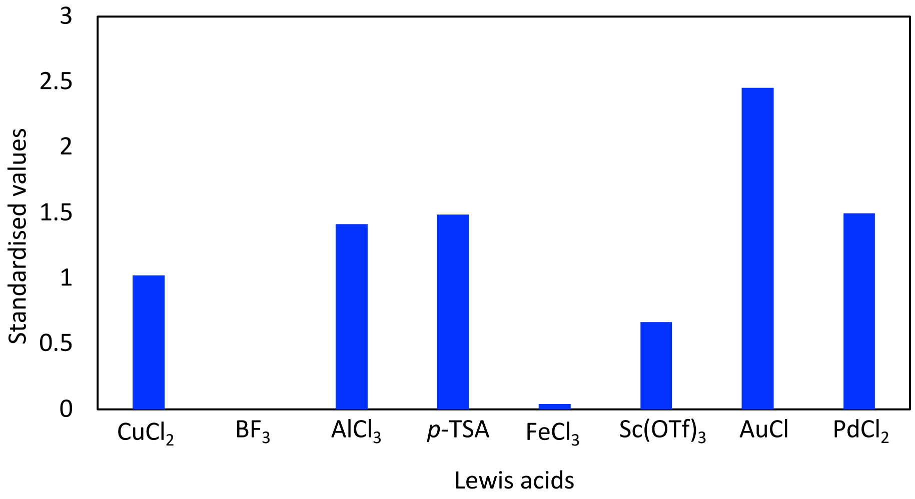

Supplementary Figure S2: Relative selectivity of Clemastine fumarate **CF 1a**, and analogues **(RR)-35** and **38l** against a panel of neurotransmitter receptors as determined by radioligand displacement .

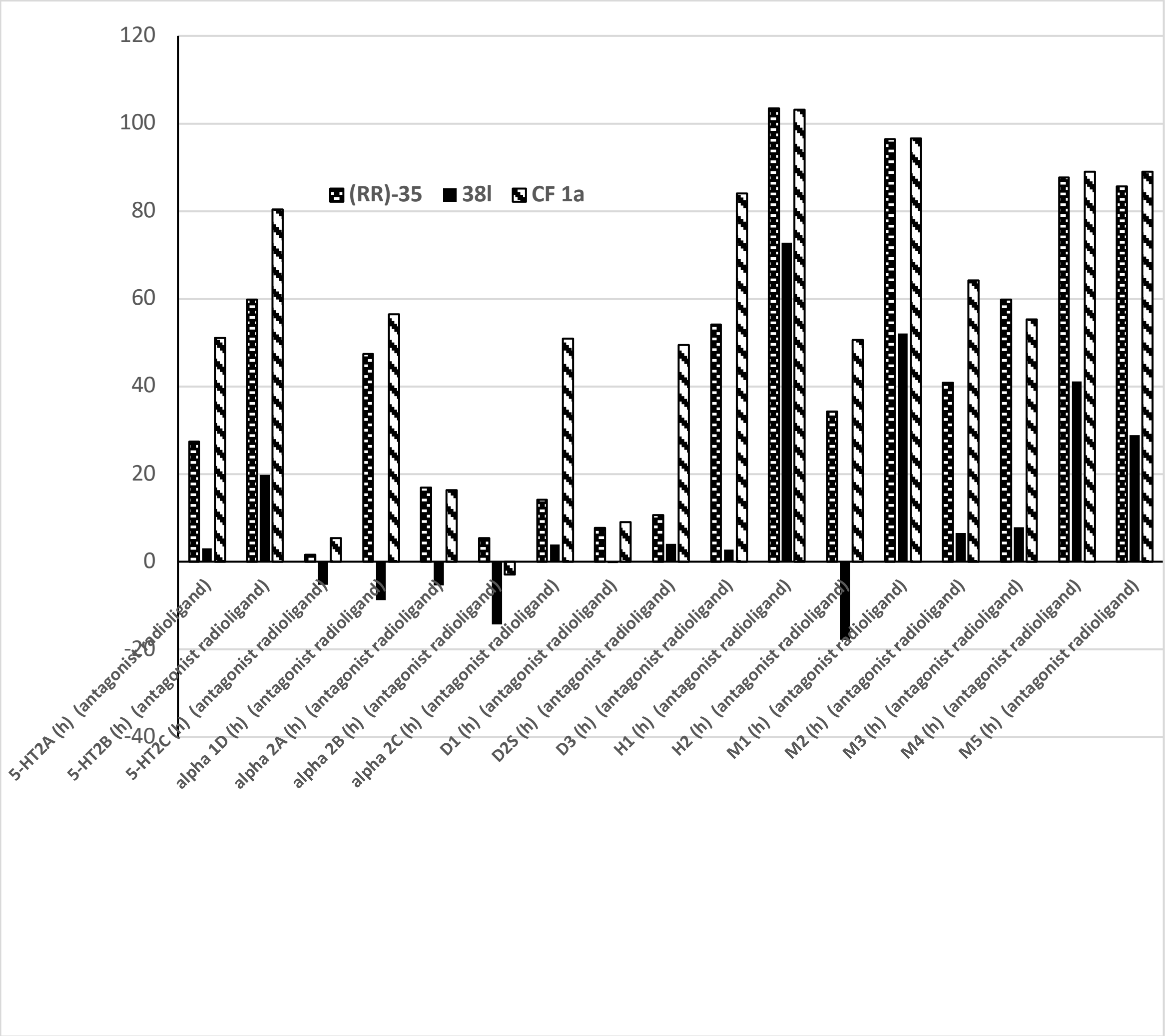

4 Experimental Procedures

4.1 Chemical Synthesis

4.1.1 General experimental details

SOLVENTS AND REAGENTS: All analytical grade solvents and commercially available reagents were used as received from their respective suppliers or dried as required, using standard procedures. All reactions were performed under an inert atmosphere of argon unless otherwise stated.

CHARACTERISATION: Reactions were monitored by LC-MS, GC-MS or by TLC using aluminium backed plates. Methods to visualise the spots included ultra-violet light (254 nm) and colour reagents. The visualising stains used were potassium permanganate, phosphomolybdic acid or ninhydrin. Column chromatography was performed using a Teledyne Isco CombiFlash® System with RediSep® Rf normal-phase and C-18 reversed- phase columns. Both carbon and hydrogen NMR spectra were acquired at 295 K on Varian VNMRS 700 (^{1}H at 700 MHz, ^{13}C at 176 MHz), Varian VNMRS 600 (^{1}H at 600 MHz, ^{13}C at 151 MHz), in $CDCl_3$ unless otherwise stated. 2D COSY, HSQC, HMBC and NOESY were run to aid assignment of peaks. Chemical shifts are reported in parts per million, ppm, to 2 decimal places; with the multiplicity of the signal reported as s, singlet; d, doublet; t, triplet; coupling constants, *J*, are quoted to the nearest ± 0.5 Hz. Ar refers to aryl resonances which could not be accurately assigned. Infrared (IR) spectra were acquired using a Perkin-Elmer Paragon 1000 FT-IR and absorption maxima (ν_{max}) are reported in wavenumbers (cm^{-1}) and assigned as strong (s), medium (m), weak (w) or broad (br). Mass spectra were recorded using Shimadzu gas chromatography *via* electron ionization (EI) or on a Waters TQD spectrometer coupled to an Acquity UPLC. Melting points are measured using Fisher ScientificTM IA9000 melting point apparatus.

4.1.2 Key Building blocks

General Procedure A: Preparation of diaryl carbinols 5

Magnesium turnings (1.7 equiv.) were added to a 2-necked flask equipped with a reflux condenser and a dropping funnel containing a solution of bromobenzene (1.3 equiv.) in THF (1M). About one-quarter of the bromobenzene solution was added to the magnesium turnings and heated to 60 °C. Once the reaction had initiated bromobenzene solution was added dropwise and the reaction was heated under reflux for 1 h. The solution was cooled to rt and the substituted benzaldehyde (1 equiv.) in THF (1M) was added dropwise. The reaction was stirred overnight at rt and then quenched with saturated NH_4Cl(aq.). The mixture was washed with EtOAc (3 x 20 mL), dried over $MgSO_4$ filtered, concentrated and the residue purified by chromatography.

4-Chlorophenyl(phenyl)methanol **5a**

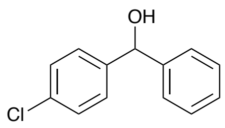

Obtained following general procedure A in 98% yield as a white solid. m.p. 59 – 60 °C; ν_{max} (ATR) 3355 (br, m), 3065 (w), 3030 (w), 2981 (w), 1601 (w), 1486 (s), 1453 (m), 1402 (m), 1345 (w), 1319 (w), 1288 (w), 1273 (w), 1247 (w), 1192 (m), 1086 (m), 1034 (m), 1022 (s), 1011 (s) cm^{-1}. δ_H (700 MHz, $CDCl_3$) 7.36 – 7.26 (9H, m, Ar***H***), 5.81 (1H, s, Ar_2C***H***), 2.25 (1H, s, O***H***). δ_C (176 MHz, $CDCl_3$) 143.6 (Ar***C***), 142.3 (Ar***C***), 133.4 (Ar***C***), 128.8 (Ar***C***), 128.7 (Ar***C***), 128.0 (Ar***C***), 128.0 (Ar***C***), 126.7 (Ar***C***), 75.6 (Ar_2***C***). *m/z* (LC-MS, ESI$^+$) 218 (M(^{35}Cl)H$^+$), 220 (M(^{37}Cl)H$^+$).

4-Bromophenyl(phenyl)methanol **5c**

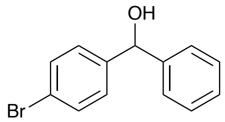

Obtained following general procedure A in 66% yield as an off-white solid. m.p. 64 – 65 °C; ν_{max} (ATR) 3352 (br), 3063 (w), 2904 (w), 1906 (w), 1600 (w), 1481 (s), 1397 (m), 1190 (m), 1008 (s) cm^{-1}; δ_H (700 MHz, $CDCl_3$) 7.47 – 7.43 (2H, m, Ar***H***), 7.36 – 7.31 (4H, m, Ar***H***), 7.30 – 7.27 (1H, m, Ar***H***), 7.26 – 7.21 (2H, m, Ar***H***), 5.76 (1H, s, Ar_2C***H***), 2.39 (1H, s, O***H***); δ_C (176 MHz, $CDCl_3$) 143.5 (***C***-1'), 142.8 (***C***-1), 131.7 (Ar***C***), 128.8 (Ar***C***), 128.3 (Ar***C***), 128.0 (Ar***C***), 126.6 (Ar***C***), 121.5 (***C***-4), 75.8 (Ar_2***C***HO); *m/z* (LC-MS, ESI$^+$) 245 (M(^{79}Br)–OH$^+$), 247 (M(^{81}Br)–OH$^+$).

(4-Methoxyphenyl)(phenyl)methanol **5d**

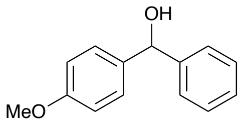

Obtained following general procedure A in 43% yield as a white solid. m.p. 63 – 64 °C; ν_{max} (ATR) 3401 (br, w), 1610 (m), 1587 (w), 1510 (s), 1494 (s), 1445 (s), 1304 (w), 1249 (s), 1239 (s), 1172 (s), 1110 (w), 1031 (s), 1018 (s), 1008 (s) cm^{-1}. δ_H (700 MHz, $CDCl_3$) 7.40 - 7.36 (2H, m, Ar***H***), 7.36 - 7.32 (2H, m, Ar***H***), 7.32 – 7.26 (3H, m, Ar***H***), 6.89 – 6.86 (2H, m, Ar***H***), 5.83 – 5.80 (1H, m, Ar_2C***H***), 3.80 – 3.78 (3H, m, C***H***$_3$), 2.19 – 2.17 (1H, m, O***H***). δ_C (176 MHz, $CDCl_3$) 159.2 (Ar***C***), 144.2 (Ar***C***), 136.3 (Ar***C***), 128.6 (Ar***C***), 128.1 (Ar***C***), 127.6 (Ar***C***), 126.5 (Ar***C***), 114.0 (Ar***C***), 76.0 (Ar_2***C***), 55.4 (***C***H$_3$). *m/z* (GC-MS, EI) 214 (M$^+$).

(3-Methoxyphenyl)(phenyl)methanol **5e**

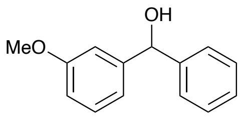

Obtained following general procedure A in 66% yield as a yellow oil. ν_{max} (ATR) 3404 (w), 1597 (m), 1585 (m), 1487 (m), 1453 (m), 1435 (m), 1314 (w), 1255 (s), 1186 (w), 1147 (m), 1021 (s) cm^{-1}. δ_H (700 MHz, $CDCl_3$) 7.40 - 7.37 (2H, m, Ar***H***), 7.36 - 7.33 (2H, m, Ar***H***), 7.30 – 7.24 (2H, m, Ar***H***), 6.98 – 6.95 (2H, m, Ar***H***), 6.83 – 6.80 (1H, m, Ar***H***), 5.79 (1H, s, Ar_2C***H***), 3.79 (3H, s, C***H***$_3$), 2.45 (1H, s, O***H***). δ_C (176 MHz, $CDCl_3$) 159.8 (***C***-3), 145.6 (***C***-1), 143.8 (***C***-1'), 129.6 (Ar***C***), 128.6 (Ar***C***), 127.7 (ArC), 126.6 (Ar***C***), 119.0 (Ar***C***), 113.1 (Ar***C***), 112.2 (Ar***C***), 76.2 (Ar_2***C***), 55.3 (***C***H$_3$). *m/z* (GC-MS, EI) 214 (M^+).

(4-Fluorophenyl)(phenyl)methanol **5f**

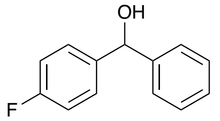

Obtained following general procedure A in 99% yield as a white solid m.p. 48– 49 °C; ν_{max} (ATR) 3378 (br, m), 1602 (m), 1508 (s), 1494 (s), 1447 (m), 1419 (w), 1335 (w), 1270 (w), 1227 (s), 1173 (m), 1159 (m), 1097 (w), 1014 (s). δ_H (700 MHz, $CDCl_3$) 7.38 – 7.28 (7H, m, Ar***H***), 7.04 – 7.00 (2H, m, Ar***H***), 5.77 (1H, s, Ar_2C***H***), 2.64 (1H, s, O***H***). δ_C (176 MHz, $CDCl_3$) 162.2 (d, *J* = 245.5 Hz, ***C***-4), 143.7 (***C***-1'), 139.7 (d, *J* = 3.0 Hz, ***C***-1), 128.7 (Ar***C***), 128.3 (d, *J* = 8.0 Hz, ***C***-2), 127.8 (Ar***C***), 126.6 (Ar***C***), 115.4 (d, *J* = 21.5 Hz, ***C***-3), 75.6 (Ar_2***C***H). δ_F (376 MHz, $CDCl_3$) - 115.06 (s, 4-***F***). m/z (GC-MS, EI) 214 (M^+).

Bis(4-fluorophenyl)methanol **5g**

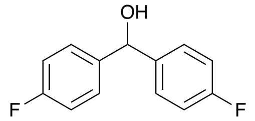

Obtained following general procedure A in 71% yield as a low melting solid. m.p. 46 - 47 °C; ν_{max} (ATR) 3247 (br, m), 1603 (m), 1506 (s), 1416 (w), 1326 (w), 1299 (w), 1218 (s), 1182 (m), 1155 (m), 1023 (m), 1011 (m). δ_H (700 MHz, $CDCl_3$) 7.34 – 7.29 (4H, m, 2-***H***), 7.06 – 6.99 (4H, m, 3-***H***), 5.79 (1H, s, Ar_2C***H***). δ_C (176 MHz, $CDCl_3$) 162.4 (***C***-4, d, *J* = 246.0 Hz), 139.5 (***C***-1, d, *J* = 3.0 Hz), 128.3 (***C***-2, d, *J* = 8.0 Hz), 115.5 (***C***-3, d, *J* = 21.5 Hz), 75.1 (Ar_2***C***H). δ_F (376 MHz, $CDCl_3$) -114.70 (4-***F***). *m/z* (LC-MS, ESI$^+$) 203 (M–OH$^+$). Accurate mass: Found (M–OH$^+$), 203.0678: $C_{13}H_9F_2$ requires *M*, 203.0672.

(R)-(4-chlorophenyl)(phenyl)methanol **(*R*)-5a** [2]

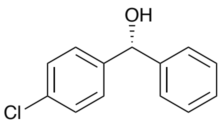

Et_2Zn (2.4 mL, 3.6 mmol) was added dropwise to a solution of phenylboronic acid (146 mg, 1.2 mmol) in toluene (3 mL) under an argon atmosphere. After stirring for 12 h at 60 °C, the mixture was cooled to 0 °C and a toluene solution of [(2*R*)-1-methylpyrrolidin-2-yl]diphenylmethanol (27 mg, 0.1 mmol) was

introduced. The reaction was stirred for an additional 15 min and the 4-chlorobenzaldehyde (70 mg, 0.5 mmol) then added. After stirring for 12 h at 0 °C the reaction was quenched with H_2O (2 mL) and extracted with DCM (3 x 5 mL). The combined organic layers were dried over $MgSO_4$, filtered, and concentrated. Purification by chromatography (10% EtOAc in hexanes) to afford the desired product (77 mg, 56%) as a white solid. $[\alpha]_D$ (c = 1.00 g/100 mL, $CHCl_3$)-27° lit:[2] $[\alpha]_D$ (c = 1.00 g/100 mL, $CHCl_3$) -16°); 95 % ee (determined by chiral HPLC analysis). ν_{max} (ATR) 3347 (br, m), 1489 (s), 1453 (m), 1089 (s), 1012 (s). δ_H (400 MHz, $CDCl_3$) 7.41 - 7.28 (9H, m, Ar***H***), 5.81 (1H, s, Ar_2C***H***), 2.42 (1H, s, O***H***). δ_C (101 MHz, $CDCl_3$) 143.5 (Ar***C***), 142.3 (Ar***C***), 133.4 (Ar***C***), 128.8 (Ar***C***), 128.7 (Ar***C***), 127.9(9) (Ar***C***), 127.9(6) (Ar***C***), 126.6 (Ar***C***), 75.7 (Ar_2***C***H). *m/z* (LC-MS, ESI^+) 201 ($M(^{35}Cl)–OH^+$), 203 ($M(^{37}Cl)$–OH+).

(S)-(4-chlorophenyl)(phenyl)methanol **(*S*)-5a**

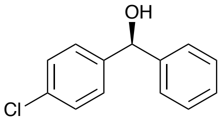

Et_2Zn (2.4 mL, 3.6 mmol) was added dropwise to a solution of phenylboronic acid (146 mg, 1.2 mmol) in toluene (3 mL) under an argon atmosphere. After stirring for 12 h at 60 °C, the mixture was cooled to 0 °C and a toluene solution of [(2S)-1-methylpyrrolidin-2-yl]diphenylmethanol (27 mg, 0.1 mmol) was introduced. The reaction was stirred for an additional 15 min and 4-chlorobenzaldehyde **8** (70 mg, 0.5 mmol) then added. After stirring for 12 h at 0 °C, the reaction was quenched with H_2O (2 mL) and extracted with DCM (3 x 5 mL). The combined organic layers were dried over $MgSO_4$, filtered and concentrated. Purification by chromatography (10% EtOAc in hexanes) to afford the desired product (134 mg, 97%) as a white solid. $[\alpha]_D$ (c = 1.00 g/100 mL, $CHCl_3$) +17.9° (lit:[2] $[\alpha]_D$ (c = 1.00 g/100 mL, $CHCl_3$) +19°) 96 % ee (determined by chiral HPLC analysis). All other data identical to that described above for the (*R*)-isomer.

(2R)-2-(2-chloroethyl)-1-methylpyrrolidine hydrochloride **(*R*)-3** [3]

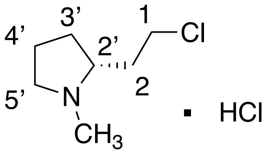

To a solution of *2-[(2R)-N-methylpyrrolidin-2'-yl]ethan-1'-ol* (77 mg, 0.59 mmol) in chloroform (1.2 mL), a solution of thionyl chloride (0.12 mL) in chloroform (0.4 ml) was added dropwise at 0 °C, and the resulting mixture was refluxed for 2h and concentrated under reduced pressure to afford the crude product as a brown oil. This was recrystalised from ethanol-diethylether to provide product (63 mg, 72%) as a brown solid. $[\alpha]_D$ (c = 1.00 g/100 mL, $CHCl_3$) +47° (lit: [3] $[\alpha]_D$ (c = 1.00 g/100 mL, $CHCl_3$) -49.8°. M.p. 126 - 127 °C (lit.[3]: 119 – 121 °C); ν_{max} (ATR) 3410 (br, w), 2959 (w), 2550 (br, w), 1495 (w). δ_H (700 MHz, $CDCl_3$) 12.56 –

12.44 (1H, s, N^+H), 3.92 - 3.84 (2H, m, 5′-***H***H′, 1-***H***H′), 3.58 - 3.53 (1H, m, 1-H***H′***), 3.41 - 3.34 (1H, m, 2′-***H***), 2.91 - 2.85 (1H, m, 5′-H***H′***), 2.84 (3H, d, *J* = 5.0 Hz, C***H₃***), 2.57 - 2.51 (1H, m, 2-***H***H′), 2.49 - 2.42 (1H, m, 2-H***H′***), 2.38 - 2.31 (1H, m, 3′-***H***H′), 2.30 - 2.23 (1H, m, 4′-***H***H′), 2.11 - 2.03 (1H, m, 4′-H***H′***), 2.02 - 1.95 (1H, m, 3′-H***H′***). δ_C (176 MHz, $CDCl_3$) 66.6 (***C***-2′), 56.4 (***C***-5′), 41.7 (***C***-1), 39.6 (***C***H_3), 32.6 (***C***-2), 29.4 (***C***-3′), 21.7 (***C***-4′). *m/z* (LC-MS, ESI^+)148 (MH+).

*4-Chlorophenyl(phenyl)azidomethane **12*** [4]

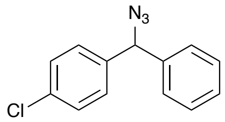

To a 0 °C solution of $TMSN_3$ (94%, 2.82 mL, 20.04 mmol), TsOH•H_2O (98%, 1.30 g, 6.68 mmol) and BF_3•Et_2O (1.68 mL, 13.36 mmol) in toluene (17 mL) was slowly added a solution of alcohol **5a** (1.46 g, 6.68 mmol) in toluene (17 mL). The reaction mixture was stirred at 0 °C for 30 min, after which TLC analysis revealed consumption of the starting material. The mixture was quenched with the addition of saturated $NaHCO_{3(aq.)}$ (15 mL) and extracted with EtOAc (3 × 20 mL). The combined organic extracts were washed with saturated $NaHCO_{3(aq.)}$ (30 mL) and brine (30 mL), then concentrated *in vacuo* before purification by chromatography on SiO_2 (10% → 20% ether in petrol) to afford the title compound (1.47 g, 90%) as a pale yellow oil. Rf 0.61 (25% ether in petrol); ν_{max} (ATR) 3083 (w), 3058 (w), 3023 (w), 2095 (s), 1594 (w), 1486 (m), 1447 (m), 1244 (m), 1089 (m), 1011 (m) cm^{-1}; δ_H (700 MHz, CDCl3) 7.39 – 7.35 (2H, m, 3′-H_2), 7.34 – 7.31 (3H, m, 3-H_2, 4′-H), 7.28 (2H, d, J = 7.5 Hz, 2′-*H_2*), 7.25 (2H, d, J = 8.5 Hz, 2-*H_2*), 5.68 (1H, s, $Ar_2CH(N_3)$); δ_C (176 MHz, CDCl3) 139.1 (C-1′), 138.2 (C-1), 133.9 (C-4), 128.9 (C-3′), 128.8 (C-3), 128.7 (C-2), 128.3 (C-4′), 127.4 (C-2′), 67.8 (Ar2CH(N3)); m/z (LCMS,ESI^+) 216 ($M(^{35}Cl) - N_2 + H^+$), 218 ($M(^{37}Cl) - N_2 + H^+$).

*(2S)-(N-tert-Butoxycarbonyl)-2-ethynylpyrrolidine **13*** [5, 6]

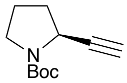

A solution of *(S) N-Boc-prolinal* (1.44 g, 7.23 mmol) in DCM (14 mL) was added dropwise to a 0 °C solution of PPh_3 (7.58 g, 28.91 mmol) and CBr_4 (4.79 g, 14.45 mmol) in DCM (120 mL). The resulting solution was stirred at rt for 30 min before being poured into cooled saturated $NaHCO_{3(aq.)}$ (100 mL) and the phases separated. The aqueous phase was washed with DCM (20 mL) and the combined organic extracts dried over $MgSO_4$ and concentrated under reduced pressure. The crude residue was purified by column chromatography (40:1:1 → 10:1:1, petrol : EtOAc : EtOH) to afford *(2S)-(N-tert-Butoxycarbonyl)-2-(2′,2′-dibromoethenyl)pyrrolidine* (1.52 g, 59%) as an off-white solid. R_f 0.45 (19:1:1, petrol : EtOAc : EtOH); M.p. 62 – 64 °C (lit.:[6] 58 – 59 °C); To a solution of *(2S)-(N-tert-Butoxycarbonyl)-2-(2′,2′-dibromoethenyl)pyrrolidine* (1.33 g, 3.75 mmol) in THF (33 mL), cooled to −78 °C, was added butyllithium

(1.6 M in hexanes, 24.70 mL, 7.49 mmol). The reaction mixture was stirred at −78 °C for 1 h, when consumption of starting material was observed by TLC, then quenched by the addition of saturated $NH_4Cl_{(aq.)}$ (30 mL). The mixture was allowed to warm to RT, after which THF was removed under reduced pressure before extraction with ether (3 × 20 mL). The combined organic extracts were washed with brine (30 mL), dried over $MgSO_4$ and concentrated *in vacuo* prior to purification by SiO_2 column (10% → 30% EtOAc in petrol) to furnish the title compound (549 mg, 75%) as a yellow oil. R_f 0.31 (19:1:1, petrol : EtOAc : EtOH); $[\alpha]^{27}_D$ (c = 1.00 g/100 mL, $CHCl_3$) −49.5° (lit.:[5] $[\alpha]^{25}_D$ (c = 1.35 g/100 mL, $CHCl_3$) −66.3°); ν_{max} (ATR) 3304 (w), 3239 (w), 2971 (m), 2872 (w), 1689 (s), 1392 (s), 1365 (m), 1244 (m), 1158 (s), 1115 (m) cm^{-1}; δ_H (700 MHz, $CDCl_3$, mixture of rotamers) 4.50 – 4.42 (0.4H, m, 2-***H*** rotamer B), 4.40 – 4.33 (0.6H, m, 2-***H*** rotamer A), 3.46 – 3.38 (1H, m, 5-***H***H′), 3.31 – 3.23 (1H, m, 5-H***H′***), 2.21 – 2.15 (1H, m, 2′-***H***), 2.08 – 2.00 (2H, m, 3-***H***H′, 4-***H***H′), 2.00 – 1.96 (1H, m, 3-H***H′***), 1.89 – 1.83 (1H, m, 4-H***H′***), 1.44 (9H, s, C(C***H***$_3$)$_3$); δ_C (176 MHz, $CDCl_3$, mixture of rotamers) 154.0 (***C***=O), 84.4 (***C***-1′ rotamer A), 84.1 (***C***-1′ rotamer B), 79.7 (***C***(CH_3)$_3$), 69.8 (***C***-2′ rotamer B), 69.4 (***C***-2′ rotamer A), 48.0 (***C***-2 rotamer A), 47.7 (***C***-2 rotamer B), 45.9 (***C***-5 rotamer B), 45.4 (***C***-5 rotamer A), 33.6 (***C***-3 rotamer A), 32.9 (***C***-3 rotamer B), 28.4 (C(***C***H$_3$)$_3$), 24.4 (***C***-4 rotamer B), 23.5 (***C***-4 rotamer A); *m/z* (LCMS, ESI^+) 218 (MNa^+).

(4′-Chlorophenyl)phenylacetic acid **16**[7]

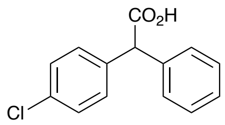

$SnCl_4$ (1.89 mL, 16.00 mmol) was added slowly to a solution of mandelic acid (1.54 g, 10.00 mmol) in chlorobenzene (6 mL). The reaction mixture was heated to reflux for 5.5 h before being cooled in an ice bath and quenched by the slow addition of saturated $NH_4Cl_{(aq.)}$ (20 mL) and H_2O (10 mL). The biphasic mixture was extracted with DCM (3 × 15 mL), concentrated and the resultant crude product then recrystallised from acetone and petrol to provide the title compound (985 mg, 40%) as an off-white solid. R_f 0.52 (10% MeOH in DCM); M.p. 109 – 111 °C (lit.[7]: 116 °C); ν_{max} (ATR) 3028 (br), 1707 (s), 1491 (m), 1403 (w), 1277 (w), 1215 (w), 1092 (w), 1015 (w) cm^{-1}; δ_H (700 MHz, $CDCl_3$) 7.37 – 7.26 (9H, m, Ar***H***), 5.02 (1H, s, 2-***H***); δ_C (176 MHz, $CDCl_3$) 178.2 (***C***=O), 137.6 (Ar***C***), 136.5 (Ar***C***), 133.7 (***C***-4′), 130.2 (Ar***C***), 128.9(6) (Ar***C***), 128.9(5), (Ar***C***), 128.7 (Ar***C***), 127.9 (Ar***C***), 56.4 (***C***-2); *m/z* (LCMS, ESI^-) 201 (M(^{35}Cl) − CO_2), 203 (M(^{37}Cl) − CO_2).

1-[(2-Bromoethoxy)(phenyl)methyl]-4-chlorobenzene [8]

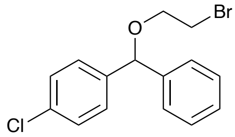

To a solution of *4-chlorophenyl(phenyl)methanol* **5a** (218 mg, 1.00 mmol) and AuCl (33 mg, 0.23 mmol) in DCE was added 2-bromoethanol (0.1 mL, 1.41 mmol). The reaction was heated to 80 °C for 20 h. The solvent was then removed under reduced pressure and the residue purified by flash column chromatography (0 → 20% EtOAc in hexanes) to afford the title compound (282 mg, 87%) as a colourless oil. ν_{max} (ATR) 2858 (w), 1490 (m), 1453 (w), 1276 (w), 1185 (w), 1089 (m), 1029 (w), 1015 (m) cm^{-1}. δ_H (700 MHz, $CDCl_3$) 7.37 – 7.33 (4H, m, Ar***H***), 7.32 – 7.27 (5H, m, Ar***H***), 5.41 (1H, s, Ar_2C***H***), 3.82 – 3.74 (2H, m, 2'-***H***), 3.53 (2H, t, *J* = 6.0 Hz, 1'-***H***). δ_C (176 MHz, $CDCl_3$) 141.3 (Ar***C***), 140.5 (Ar***C***), 133.5 (Ar***C***), 128.7(2) (Ar***C***), 128.7(1) (Ar***C***), 128.4(5) (Ar***C***), 128.0 (Ar***C***), 127.1 (Ar***C***), 83.4 (Ar_2C***H***), 69.1 (***C***-2'), 30.7 (***C***-1'). *m/z* (LC-MS, ESI$^+$) 347 (M(^{35}Cl)(^{79}Br) Na$^+$), 349 (M(^{37}Cl)(^{79}Br) Na$^+$) (M(^{35}Cl)(^{81}Br) Na$^+$), 351 (M(^{37}Cl)(^{81}Br) Na$^+$).

1-[(3-bromopropoxy)(phenyl)methyl]-4-chlorobenzene

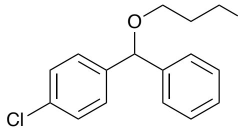

To a solution of *4-chlorophenyl(phenyl)methanol* **5a** (500 mg, 2.29 mmol) and AuCl (33 mg, 0.23 mmol) in DCE was added 3-bromopropanol (318 mg, 2.29 mmol). This reaction was heated to 80 °C for 16 h. The solvent was then removed under reduced pressure and the residue purified by flash column chromatography (0 → 20% EtOAc in hexanes) to afford the product as a colourless oil (244 mg, 54%). ν_{max} (ATR) 3029 (w), 2866 (w), 1489 (m), 1088 (s), 1014 (m). δ_H (700 MHz, $CDCl_3$) 7.37 – 7.27 (9H, m, Ar***H***), 5.5 (1H, s, Ar_2C***H***), 3.61 – 3.56 (4H, m, 3'-***H***$_2$, 1'-***H***$_2$), 2.18 (2H, p, *J* = 6.0 Hz, 2'-***H***$_2$). δ_C (176 MHz, $CDCl_3$) 141.8 (Ar***C***), 140.9 (Ar***C***), 133.3 (Ar***C***), 128.7 (Ar***C***), 128.6 (Ar***C***), 128.4 (Ar***C***), 127.9 (Ar***C***), 127.0 (Ar***C***), 83.3 (Ar_2***C***H), 66.6 (***C***-3'), 33.1 (***C***-2'), 30.8 (***C***-1'). *m/z* (LC-MS, ESI$^+$) 201 (M(^{35}Cl)-$OC_3H_6Br^+$), 203 (M(^{37}Cl)- $OC_3H_6Br^+$). Accurate mass: Found (M-$OC_3H_6Br^+$), 201.0474: $C_{13}H_{10}{}^{35}Cl$ requires *M,* 201.0471.

1-[(4'-bromobutoxy)(phenyl)methyl]-4-chlorobenzene

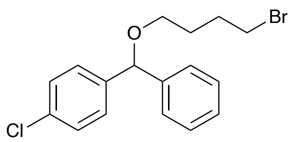

To a solution of *4-chlorophenyl(phenyl)methanol* **5a** (531 mg, 2.43 mmol) and AuCl (56 mg, 0.24 mmol) in DCE was added 4-bromobutanol (372 mg, 2.43 mmol). The reaction was heated to 80 °C for 20 h. The solvent was then removed under reduced pressure and the residue purified by flash column chromatography (0 → 20% EtOAc in hexanes) to afford the title compound (350 mg, 41%) as a colourless oil. ν_{max} (ATR) 3029 (w), 2941 (w), 2863 (w), 1489 (m), 1086 (s), 1014 (m). δ_H (600 MHz, $CDCl_3$) 7.36 – 7.24 (9H, m, Ar***H***), 5.30 (1H, s, Ar_2C***H***), 3.47 (2H, t, *J* = 6.5 Hz, 4'-***H***$_2$), 3.43 (2H, t, *J* = 6.5 Hz, 1'-***H***$_2$), 2.00 (2H, p, *J* = 6.5 Hz, 2'-***H***), 1.79 (2H, p, *J* = 6.5 Hz, 3'-***H***). δ_C (176 MHz, $CDCl_3$) 142.0 (***C***-1^{Ph}), 141.1 (***C***-1'^{Ph}), 133.2 (***C***-4^{Ph}),

128.6(4) (***C***-3′^{Ph}), 128.6(1) (***C***-3^{Ph}), 128.3 (***C***-2^{Ph}), 127.8 (***C***-4′^{Ph}), 127.0 (***C***-2′^{Ph}), 83.1 (Ar_2***C***H), 68.1 (***C***-4′), 33.8 (***C***-1′), 29.9 (***C***-2′), 28.5 (***C***-3′). *m/z* (LC-MS, ESI^+) 201 ($M(^{35}Cl)-OC_4H_8Br]^+$), 203 ($M(^{37}Cl)-OC_4H_8Br]^+$). Accurate mass: Found ($[M-OC_4H_8Br]^+$), 201.0480: $C_{13}H_{10}{}^{35}Cl$ requires *M,* 201.0471.

(2R)-1-(2-chloroethyl)-2-methylpyrrolidine hydrochloride

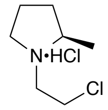

2-Bromoethanol (0.23 mL, 3.20 mmol) in dry MeCN (2 mL) was added dropwise to a mixture of *(R)-2-methylpyrrolidine* (415mg, 3.32 mmol) and K_2CO_3 (918 mg, 6.64 mmol) in dry MeCN (2 mL) heated under reflux. After 15 h the mixture was cooled to rt, filtered and concentrated. Et_2O (5 mL) was then added and the product extracted with 1M HCl (2 x 5 mL). The aqueous phase was made basic with solid NaOH, then extracted with DCM (3 x 5 mL). The organic layers were combined, dried over Na_2SO_4, filtered and concentrated to afford *2-((2R)-2-methylpyrrolidin-1-yl)ethan-1-ol* (200 mg, 48%) as a colourless oil which was used directly in the next step without further purification. $[\alpha]_D$ (c = 1.00 g/100 mL, $CHCl_3$) -58.8˚. ν_{max} (ATR) 3385 (br, m), 2963 (m), 2875 (m), 2806 (m), 1677 (m), 1416 (m), 1509 (m). δ_H (400 MHz, $CDCl_3$) 4.03 – 3.86 (3H, m, 2-***H*₂**, 5′-***H***H′), 3.42 – 3.35 (1H, m, 1-***H***H′), 3.33 – 3.23 (1H, m, 2′-***H***), 3.01 – 2.91 (2H, m, 1-H***H′***, 5′-H***H′***), 2.29 – 2.18 (2H, m, 3′-***H***H′, 4-***H***H′), 2.08 – 1.98 (1H, m, 4-H***H′***), 1.97 - 1.87 (2H, m, 3′-H***H′***), 1.54 (3H, d, *J* = 6.5 Hz, C***H₃***). δ_C (176 MHz, $CDCl_3$) 64.6 (***C***-2′), 57.6 (***C***-2), 57.2 (***C***-5′), 54.6 (***C***-1), 31.4 (***C***-3′), 21.7 (***C***-4′), 15.9 (***C***H_3). *m/z* (LC-MS, ESI^+) 130 (MH+). Accurate mass: Found (MH^+), 130.1232: $C_7H_{16}NO$ requires *M*, 130.1232. A solution of thionyl chloride (0.57 mL, 7.87 mmol) in chloroform (3 mL) was added dropwise to a solution of *2-((2R)-2-methylpyrrolidin-1-yl)ethan-1-ol* (377 mg, 2.92 mmol) in chloroform (4 mL) at 0 °C. The resulting mixture was heated under reflux for 2 h and then concentrated under reduced pressure. Precipitation from a mixture of EtOH and Et_2O afforded the title compound contaminated with 19% 2-((2*R*)-2-methylpyrrolidin-1-yl)ethan-1-ol hydrochloride (353 mg, 35%) as a brown semi-solid. $[\alpha]_D$ (c = 1.00 g/100 mL, $CHCl_3$) -23.7˚. ν_{max} (ATR) 3392 (br, m), 2974 (m), 2554 (m), 2453 (m), 1452 (m), 1422 (m), 1393 (m). δ_H (700 MHz, $CDCl_3$, 19% 2-((2R)-2-methylpyrrolidin-1-yl)ethan-1-ol hydrochloride) 12.56 (1H, s, N***H⁺***), 4.15 – 4.10 (1H, m, 4′-***H***H′), 4.05 – 3.99 (1H, m, 4-H***H′***), 3.97 – 3.90 (1H, m, 5′-***H***H′), 3.72 – 3.66 (1H, m, 3′-***H***H′), 3.31 – 3.24 (1H, m, 2′-***H***), 3.12 – 3.05 (1H, m, 3′-H***H′***), 3.04 – 2.97 (1H, m, 5′-H***H′***), 2.30 – 2.17 (2H, m, 2-***H₂***), 2.08 – 1.95 (2H, m, 1-***H₂***), 1.64 (3H, d, *J* = 6.5 Hz, C***H₃***). δ_C (176 MHz, $CDCl_3$) 65.3 (***C***-2′), 54.0 (***C***-3′), 53.8 (***C***-5′), 37.6 (***C***-4′), 31.1 (***C***-2), 21.6 (***C***-1), 15.7 (***C***H_3). *m/z* (LC-MS, ESI^+) 148 ($M(^{35}Cl)H^+$), 150 ($M(^{37}Cl)H^+$). In an identical fashion *(2S)-1-(2-chloroethyl)-2-methylpyrrolidine hydrochloride* was prepared from *(S)-2-methylpyrrolidine* $[\alpha]_D$ (c = 1.00 g/100 mL, $CHCl_3$) +20.4˚. All spectroscopic data identical with that reported for the *R* enantiomer.

(2S)-1-(3-chloropropyl)-2-methylpyrrolidine hydrochloride

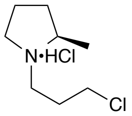

3-Bromopropanol (0.72 mL, 7.9 mmol) in dry MeCN (5 mL) was added dropwise to a mixture of (*S*)-2-methylpyrrolidine (1 g, 8.2 mmol) and K_2CO_3 (2.27g, 16.4 mmol) in dry MeCN (5 mL) heated under reflux. After 15 h the mixture was cooled to rt, filtered and concentrated. Et_2O (10 mL) was then added and the product extracted with 1M HCl (2 x 10 mL). The aqueous phase was made more basic with solid NaOH, then extracted with DCM (3 x 10 mL). The organic layers were combined, dried over Na_2SO_4, filtered and concentrated to afford *3-[(2S)-2-methylpyrrolidin-1-yl]propan-1-ol* (620 mg, 55%) as a colourless oil which was used directly in the next step without further purification. ν_{max} (ATR) 3372 (br), 2961 (m), 2872 (w), 1114 (w). δ_H (400 MHz, $CDCl_3$) 3.81 – 3.72 (2H, m, 3-***H₂***), 3.35 – 3.26 (1H, m, 1-***H***H'), 3.02 – 2.91 (1H, m, 5'-***H***H'), 2.43 – 2.34 (1H, m, 5'-H***H'***), 2.33 – 2.23 (1H, m, 2'-***H***H'), 2.13 - 2.02 (1H, m, 1-H***H'***), 2.01 - 1.81 (2H, m, 2-***H***H', 3'-***H***H'), 1.79 – 1.60 (2H, m, 4'-***H₂***), 1.58 - 1.46 (1H, m, 2-H***H'***), 1.43 - 1.29 (1H, m, 3'-H***H'***), 1.11 (3H, d, *J* = 6.0 Hz, C***H₃***). δ_C (176 MHz, $CDCl_3$) 69.3 (***C***-2'), 61.9 (***C***-5'), 54.0 (***C***-1), 50.9 (***C***-3), 32.2 (***C***-3'), 27.3 (***C***-2), 21.6 (***C***-4'), 18.1 (***C***H₃). *m/z* (LC-MS, ESI^+) 144 (MH^+). Accurate mass: Found (MH^+), 144.1393: $C_8H_{18}NO$ requires *M,* 144.1388. A solution of thionyl chloride (0.74 mL, 10.24 mmol) in chloroform (2 mL) was added dropwise to a solution of *3-[(2S)-2-methylpyrrolidin-1-yl]propan-1-ol* (491 mg, 3.43 mmol) in chloroform (6 mL) at 0 °C. The mixture was heated to reflux for 2 h and concentrated under reduced pressure. The product was precipitated from ethanol and diethylether to afford the title compound (172 mg, 25%) contaminated with ~20% 3-[(2*S*)-2-methylpyrrolidin-1-yl]propan-1-ol hydrochloride as a white solid. m.p. 125 – 127 °C. ν_{max} (ATR) 3404 (br), 2964 (m), 2600 (w), 2511 (w), 1633 (w), 1453 (w), 1063 (w). δ_H (700 MHz, $CDCl_3$, ~20% 3-[(2*S*)-2-methylpyrrolidin-1-yl]propan-1-ol hydrochloride) 12.15 (1H, s, N^+H), 3.93 - 3.83 (1H, m, 5'-***H***H'), 3.74 – 3.62 (2H, m, 3-***H₂***), 3.44 - 3.37 (1H, m, 1-***H***H'), 3.24 – 3.12 (1H, m, 2'-***H***), 3.00 – 2.92 (1H, m, 1-***H***H'), 2.89 – 2.79 (1H, m, 5'-H***H'***), 2.72 – 2.61 (1H, m, 2-***H***H'), 2.29 - 2.16 (3H, m, 3'-***H***H', 4'-***H***H', 2-H***H'***), 2.11 – 1.96 (2H, m, 3'-H***H'***, 4'- H***H'***), 1.64 (3H, d, *J* = 6.5 Hz, C***H₃***). δ_C (176 MHz, $CDCl_3$) 65.3 (***C***-2'), 53.5 (***C***-5'), 51.4 (***C***-1), 42.1 (***C***-3), 31.5 (***C***-3'), 28.2 (***C***-2), 21.5 (***C***-4'), 15.7 (***C***H₃). *m/z* (LC-MS, ESI^+) 162 ($M(^{35}Cl)H^+$), 164 ($M(^{37}Cl)H^+$). Accurate mass: Found (MH^+), 162.1041: $C_8H_{17}N^{35}Cl$ requires *M,* 162.1050.

4.1.3 Final Products

2-(2′-[{4′′′Chlorophenyl}{phenyl}methoxy]ethyl)pyrrolidine **7**

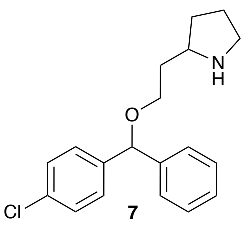

Racemic homoprolinol **6b** (100 mg, 0.45 mmol, 1 eq.), diarylcarbinol **5a** (99 mg, 0.45 mmol), *p*-toluenesulfonic acid monohydrate (1.1 eq.), toluene (0.25M) and 4 Å molecular sieves where combined and the mixture was heated under reflux for 2 h before being cooled and quenched by the addition of 1 M $NaOH_{(aq)}$. The mixture was extracted with EtOAc and the organic extracts washed with brine, dried over $MgSO_4$ and concentrated *in vacuo*. Purification of the resultant crude material by chromatography afforded (0% → 5% MeOH in $CHCl_3$ with 1% NEt_3), furnished the *title compound* (94 mg, 66%) as a clear light brown oil. R_f 0.35 (10% MeOH and 1% $NH_4OH_{(aq.)}$ in $CHCl_3$); ν_{max} (ATR) 3368 (br), 2938 (m), 2866 (m), 1489 (m), 1452 (w), 1400 (w), 1181 (w), 1087 (s), 1012 (m) cm^{-1}; δ_H (700 MHz, $CDCl_3$ mixture of diastereomers) 7.33 – 7.27 (4H, m, Ar***H***), 7.27 – 7.23 (5H, m, Ar***H***), 5.30 (1H, s, 1′′-***H***), 4.30 – 4.10 (1H, m, N***H***), 3.55 – 3.49 (2H, m, 2′-***H***$_2$), 3.31 – 3.25 (1H, m, 2-***H***), 3.06 (1H, dddd, *J* = 10.5, 9.0, 8.0, 6.0 Hz, 5-***H***H′), 2.93 (1H, dtd, *J* = 10.5, 8.5, 6.5 Hz, 5-H***H′***), 1.96 – 1.87 (2H, m, 3-***H***H′, 1′-***H***H′), 1.86 – 1.78 (2H, m, 4-***H***H′, 1′-H***H′***), 1.78 – 1.71 (1H, m, 4-H***H′***), 1.41 – 1.34 (1H, m, 3-H***H′***); δ_C (176 MHz, $CDCl_3$, mixture of diastereomers) 142.0 (Ar***C***), 141.9 (Ar***C***), 141.1(1) (Ar***C***), 141.0(5) (Ar***C***), 133.3 (Ar***C***), 133.2 (Ar***C***), 128.7 (Ar***C***), 128.6(3) (Ar***C***), 128.6(1) (Ar***C***), 128.3(8) (Ar***C***), 128.3(7) (Ar***C***), 127.7(9) (Ar***C***), 127.7(7) (Ar***C***), 127.0(3) (Ar***C***), 127.0(0) (Ar***C***), 83.2(2) (***C***-1′′), 83.2(0) (***C***-1′′), 67.3 (***C***-2′), 67.2 (***C***-2′), 57.1(7) (***C***-2), 57.1(5) (***C***-2), 46.1(3) (***C***-5), 46.1(2) (***C***-5), 35.4 (***C***-1′), 35.3 (***C***-1′), 31.6(0) (***C***-3), 31.5(7) (***C***-3), 25.0 (***C***-4), 24.9 (***C***-4); *m/z* (LCMS, ESI^+) 316 ($M(^{35}Cl)H^+$), 318 ($M(^{37}Cl)H^+$); Accurate mass: Found MH^+, 316.1467: $C_{19}H_{23}NO^{35}Cl$ requires *M*, 316.1468.

(S,RS) 2-(2′-[{4′′′-Chlorophenyl}{phenyl}methoxy]ethyl)-1-methylpyrrolidine **9**

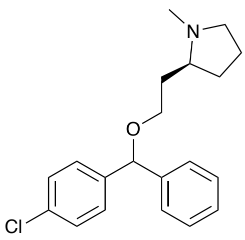

Following the same procedure as described for **7**, diarylcarbinol **5a** (109 mg, 0.50 mmol) and N-methyl homoprolinol **(*S*)-6b** (65 mg, 0.50 mmol) were combined to afford following chromatography (0% → 10% MeOH in $CHCl_3$ with 1% NEt_3), the *title compound* (56 mg, 34%) as a colourless oil. R_f 0.36 (10% MeOH and 1% NEt_3 in $CHCl_3$); ν_{max} (ATR) 2946 (m), 2940 (w), 2771 (m), 1488 (m), 1452 (m), 1086 (s), 1013 (m) cm^{-1}; δ_H (400 MHz, $CDCl_3$, mixture of diastereomers) 7.28 – 7.16 (9H, m, Ar***H***), 5.22 (1H, s, 1′′-***H***), 3.48 – 3.35 (2H,

m, 2′-***H***$_2$), 3.07 – 3.00 (1H, m, 5-***H***H′), 2.27 (3H, s, NC***H***$_3$), 2.22 – 1.97 (3H, m, 2-***H***, 5-H***H′***, 1′-***H***H′), 1.91 – 1.79 (1H, m, 3-***H***H′), 1.77 – 1.67 (1H, m, 4-***H***H′), 1.66 – 1.58 (1H, m, 4-H***H′***), 1.57 – 1.47 (1H, m, 1′-H***H′***), 1.46 – 1.36 (1H, m, 3-H***H′***); δ_C (101 MHz, $CDCl_3$, mixture of diastereomers) 142.0(9) (Ar***C***), 142.0(5) (Ar***C***), 141.2(2) (Ar***C***), 141.1(9) (Ar***C***), 133.2(4) (Ar***C***), 133.2(1) (Ar***C***), 128.6(4) (Ar***C***), 128.6(3) (Ar***C***), 128.6(0) (Ar***C***), 128.4 (Ar***C***), 128.3 (Ar***C***), 127.8 (Ar***C***), 127.7 (Ar***C***), 127.0(3) (Ar***C***), 126.9(7) (Ar***C***), 83.2(4) (***C***-1′′), 83.2(2) (***C***-1′′), 67.2 (***C***-2′), 67.1 (***C***-2′), 64.2 (***C***-2), 57.2 (***C***-5), 40.5 (N***C***H$_3$), 33.9 (***C***-1′), 31.0 (***C***-3), 30.9 (***C***-3), 22.0 (***C***-4); *m/z* (LCMS, ESI$^+$) 330 (M(^{35}Cl)H$^+$), 332 (M(^{37}Cl)H$^+$); Accurate mass: Found MH$^+$, 330.1617: $C_{20}H_{25}NO^{35}Cl$ requires *M*, 330.1625.

(R, RS) 2-(2′-[{4′′′-Chlorophenyl}{phenyl}methoxy]ethyl)-1-methylpyrrolidine **10**

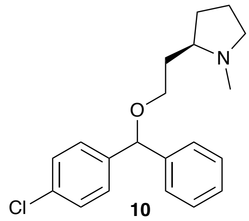

10

Following the same procedure as described for **7**, diarylcarbinol **5a** (109 mg, 0.50 mmol) and N-methyl homoprolinol **(*S*)-6b** (65 mg, 0.50 mmol) were combined to afford following chromatography (0% → 10% MeOH in $CHCl_3$ with 1% NEt_3), the *title ether 9b* (94 mg, 57%) as a viscous colourless oil. R_f 0.36 (10% MeOH and 1% NEt_3 in $CHCl_3$); ν_{max} (ATR) 3026 (w), 2938 (m), 2840 (w), 2774 (m), 1488 (m), 1452 (m), 1087 (s), 1013 (m) cm^{-1}; δ_H (700 MHz, $CDCl_3$, mixture of diastereomers) 7.33 – 7.22 (9H, m, Ar***H***), 5.28 (1H, s, Ar$_2$C***H***O), 3.52 – 3.42 (2H, m, 2′-***H***$_2$), 3.07 – 3.03 (1H, m, 5-***H***H′), 2.30 (3H, s, NC***H***$_3$), 2.18 – 2.04 (3H, m, 2-***H***, 5-H***H′***, 1′-***H***H′), 1.92 – 1.86 (1H, m, 3-***H***H′), 1.77 – 1.70 (1H, m, 4-***H***H′), 1.68 – 1.62 (1H, m, 4-H***H′***), 1.57 – 1.50 (1H, m, 1′-H***H′***), 1.48 – 1.41 (1H, m, 3-H***H′***); δ_C (176 MHz, $CDCl_3$, mixture of diastereomers) 142.2 (Ar***C***), 142.1 (Ar***C***), 141.2(8) (Ar***C***), 141.2(5) (Ar***C***), 133.2(2) (Ar***C***), 133.1(9) (Ar***C***), 128.6(4) (Ar***C***), 128.6(3) (Ar***C***), 128.6(0) (Ar***C***), 128.4 (Ar***C***), 128.3 (Ar***C***), 127.8 (Ar***C***), 127.7 (Ar***C***), 127.0(3) (Ar***C***), 126.9(8) (Ar***C***), 83.2(3) (Ar$_2$***C***HO), 83.2(1) (Ar$_2$***C***HO), 67.2(9) (***C***-2′), 67.2(6) (***C***-2′), 63.9(4) (***C***-2), 63.9(3) (***C***-2), 57.3 (***C***-5), 40.6 (N***C***H$_3$), 34.2 (***C***-1′), 31.1 (***C***-3), 31.0 (***C***-3), 22.1 (***C***-4); *m/z* (LCMS, ESI$^+$) 330 (M(^{35}Cl)H$^+$), 332 (M(^{37}Cl)H$^+$); Accurate mass: Found MH$^+$, 330.1619: $C_{20}H_{25}NO^{35}Cl$ requires *M*, 330.1625.

(S, SR) (N-Methyl)-2-(1′-[{4′′′-chlorophenyl}{phenyl}methyl]-1′,2′,3′-triazol-4′-yl)pyrrolidine **14a**

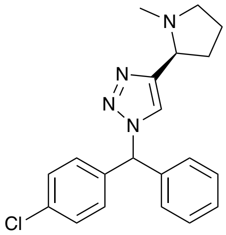

14a

A microwave vial was charged with azide **12** (139 mg, 0.57 mmol), alkyne **(*S*)-13** (98 mg, 0.50 mmol), $CuSO_4•5H_2O_{(aq.)}$ (1 M, 0.20 mL, 0.20 mmol), freshly prepared Na ascorbate$_{(aq)}$ (1 M, 1.00 mL, 1.00 mmol), H_2O (1 mL) and MeOH (1 mL). The vial was sealed and reacted at 60 °C for 40 min, with a 30 s pre-mixing time. The mixture was then cooled, diluted with H_2O (5 mL) and extracted with DCM (3 × 10 mL) and the combined organic extracts washed with brine (10 mL), dried over $MgSO_4$ and concentrated under reduced pressure. The crude residue was then purified by column chromatography (50% → 70% ether in petrol) to provide the *(N-tert-Butoxycarbonyl)-2-(1′-[{4′′′-chlorophenyl}{phenyl}methyl]-1′,2′,3′-triazol-4′-yl)pyrrolidine* (153 mg, 70%) as a white solid. R_f 0.10 (50% ether in petrol); m.p. 56 – 58 °C; ν_{max} (ATR) 2976 (w), 2928 (w), 2881 (w), 2017 (w), 1676 (s), 1490 (m), 1452 (w), 1391 (s), 1248 (w), 1162 (m), 1110 (w) cm^{-1}; δ_H (500 MHz, $(CD_3)_2SO$, 90 °C, mixture of diastereomers) 7.71 (1H, apparent d, *J* = 1.0 Hz, 1′′-***H***), 7.42 (2H, d, *J* = 8.5 Hz, Ar***H***), 7.40 – 7.34 (3H, m, Ar***H***), 7.24 – 7.18 (5H, m, Ar***H***), 4.88 (1H, dd, *J* = 8.0, 3.0 Hz, 2-***H***), 3.42 – 3.32 (2H, m, 5-$\boldsymbol{H}_2$), 2.25 – 2.16 (1H, m, 3-***H***H′), 2.10 – 2.01 (1H, m, 3-H***H′***), 2.00 – 1.91 (1H, m, 4-***H***H′), 1.89 – 1.81 (1H, m, 4-H***H′***), 1.23 (9H, s, C(C$\boldsymbol{H}_3$)$_3$); δ_C (176 MHz, $CDCl_3$, mixture of rotamers and diastereomers) 154.2 (***C***=O), 150.7 (Ar***C***), 149.1 (Ar***C***), 137.8 (Ar***C***), 136.9 (Ar***C***), 134.6 (Ar***C***), 129.3 (Ar***C***), 129.0 (Ar***C***), 127.9 (Ar***C***), 122.6 (Ar***C***), 120.9 (Ar***C***), 79.5 (***C***$(CH_3)_3$ rotamer A), 79.3 (***C***$(CH_3)_3$ rotamer B), 67.3 (***C***-1′′) 53.6 (***C***-2 rotamer A), 52.7 (***C***-2 rotamer B), 46.6 (***C***-5 rotamer B), 46.4 (***C***-5 rotamer A), 32.8 (***C***-3), 28.3 (C(***C***H_3)$_3$), 24.6 (***C***-4 rotamer B), 23.4 (***C***-4 rotamer A); *m/z* (LCMS, ESI$^+$) 439 (M(^{35}Cl)H$^+$), 441 (M(^{37}Cl)H$^+$); Accurate mass: Found MH$^+$, 439.1898: $C_{24}H_{28}N_4O_2{}^{35}Cl$ requires *M*, 439.1901. To a suspension of $LiAlH_4$ (95%, 45 mg, 1.12 mmol) in ether (4.5 mL), cooled in an ice bath, was added *(N-tert-Butoxycarbonyl)-2-(1′-[{4′′′-chlorophenyl}{phenyl}methyl]-1′,2′,3′-triazol-4′-yl)pyrrolidine* (123 mg, 0.28 mmol). The reaction mixture was allowed to warm to RT and stirred for 2 h, after which the solution was re-cooled to 0 °C quenched according to Fieser's method. The residue was purified by column chromatography (0% → 5% EtOH in petrol with 5% EtOAc and 10% Net_3) to furnish the *title compound* **14a** (34 mg, 34%) as a white solid. R_f 0.22 (5% EtOH, 5% EtOAc and 10% NEt_3 in petrol); M.p. 100 – 103 °C; ν_{max} (ATR) 2971 (m), 2933 (m), 2876 (m), 2784 (m), 1491 (m), 1438 (m) cm^{-1}; δ_H (700 MHz, $CDCl_3$, mixture of diastereomers) 7.37 – 7.30 (6H, m, Ar***H***), 7.10 – 7.07 (3H, m, Ar***H***, 1′′-***H*** isomer 1), 7.04 – 7.00 (2H, m, Ar***H***, 1′′-***H*** isomer 2), 3.44 (1H, t, *J* = 8.0 Hz, 2-***H***), 3.15 (1H, apparent t, *J* = 8.5 Hz, 5-***H***H′), 2.32 – 2.24 (2H, m, 5-H***H′***, 3-***H***H′), 2.26 (3H, s, NC$\boldsymbol{H}_3$), 1.94 – 1.86 (1H, m, 4-***H***H′), 1.86 – 1.77 (2H, m, 3-H***H′***, 4-H***H′***);

δ_C (176 MHz, $CDCl_3$, mixture of diastereomers) 150.4 (***C***-4′ isomer 1), 150.1 (***C***-4′ isomer 2), 138.3 (Ar***C***), 137.7 (Ar***C***), 136.9 (Ar***C***), 134.5 (Ar***C***), 129.4 (Ar***C***), 129.3 (Ar***C***), 129.1 (Ar***C***), 129.0 (Ar***C***), 128.9 (Ar***C***), 128.7 (Ar***C***), 128.5 (Ar***C***), 128.4 (Ar***C***), 128.1 (Ar***C***), 128.0 (Ar***C***), 120.6 (***C***-5′ isomer 1), 120.5 (***C***-5′ isomer 2), 68.1 (***C***-1′′ isomer 1), 67.4 (***C***-1′′ isomer 2), 62.8 (***C***-2 isomer 1), 62.7 (***C***-2 isomer 2), 56.8 (***C***-5), 40.6 (***C***-6), 33.2 (***C***-3), 22.3 (***C***-4); *m/z* (LCMS, ESI$^+$) 353 (M(^{35}Cl)H$^+$), 355 (M(^{37}Cl)H$^+$); Accurate mass: Found MH$^+$, 353.1530: $C_{20}H_{22}N_4{}^{35}Cl$ requires *M*, 353.1533.

(R, SR)-(N-Methyl)-2-(1′-[{4′′′-chlorophenyl}{phenyl}methyl]-1′,2′,3′-triazol-4′-yl)pyrrolidine **14b**

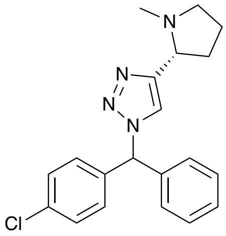

14b

In an identical fashion to that describe above for **14a**, *(N-Methyl)-2-(1′-[{4′′′-chlorophenyl}{phenyl}methyl]-1′,2′,3′-triazol-4′-yl)pyrrolidine* **14b** was prepared via cycloaddition of azide with *(R)*-alkyne **13** followed by reduction of the resultant triazole with $LiAlH_4$. R_f 0.22 (5% EtOH, 5% EtOAc and 10% NEt_3 in petrol); ν_{max} (ATR) 2950 (m), 2840 (m), 2773 (m), 1491 (s), 1451 (m), 1210 (m), 1090 (s), 1041 (s) cm^{-1}; δ_H (400 MHz, $CDCl_3$, mixture of diastereomers) 7.38 – 7.29 (6H, m, Ar***H***), 7.12 – 7.00 (5H, m, Ar***H***, 1′′-***H***), 3.46 (1H, t, *J* = 8.0 Hz, 2-***H***), 3.16 (1H, ddd, *J* = 10.0, 8.5, 2.5 Hz, 5-***H***H′), 2.36 – 2.22 (5H, m, 3-***H***H′, 5-H***H′***, NC***H₃***), 1.96 – 1.77 (3H, m, 3-H***H′***, 4-***H₂***); δ_C (101 MHz, $CDCl_3$, mixture of diastereomers) 150.4 (***C***-4′), 138.4 (Ar***C***), 137.9 (Ar***C***), 137.0 (Ar***C***), 134.6 (Ar***C***), 129.6 (Ar***C***), 129.5 (Ar***C***), 129.2(3) (Ar***C***), 129.1(8) (Ar***C***), 129.0 (Ar***C***), 128.9(0) (Ar***C***), 128.8(7) (Ar***C***), 128.6(2) (Ar***C***), 128.6(0) (Ar***C***), 128.3 (Ar***C***), 128.2 (Ar***C***), 120.8 (***C***-5′), 67.6 (***C***-1′′), 62.9 (***C***-2), 57.0 (***C***-5), 40.7 (N***C***H₃), 33.3 (***C***-3), 22.5 (***C***-4); *m/z* (LCMS, ESI$^+$) 353 (M(^{35}Cl)H$^+$), 355 (M(^{37}Cl)H$^+$); Accurate mass: Found MH$^+$, 353.1527: $C_{20}H_{22}N_4{}^{35}Cl$ requires *M*, 353.1533.

General procedure B: formation of amides 19, 21-24, S1-S58

To a mixture of amine (1 eq.), *N*-methylmorpholine (NMM) (1.1 eq.) and EDCI•HCl (1.1 eq.) in DCM (3 mL.mmol^{-1}) at 0 °C was added acid (1 eq.) and HOBt (1.1 eq.) in DCM (3 mL.mmol^{-1}). DMF was added as required to aid solvation. After being stirred at RT overnight, the reaction mixture was quenched with H_2O (4 mL.mmol^{-1}) and passed through a phase separator. The resultant solution was concentrated and purified *via* reversed-phase flash column chromatography to afford the amide. Where relevant, deprotection of the N Boc group was achieved by dissolving the protected amide in a mixture of DCM (0.9 mL) and TFA (0.1 mL). After stirring at rt for 3 h, all volatiles were removed under high vacuum to provide the *title compound*.

N-([Pyrrolidin-2′′-yl]methyl)-2-(4′-chlorophenyl)-2-phenylacetamide TFA salt **19**

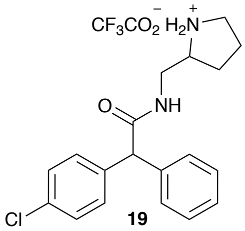

M.p. 149 – 151 °C; ν_{max} (ATR) 3252 (br), 3066 (w), 1665 (s), 1646 (s), 1563 (m), 1191 (m), 1156 (m) 1133 (s) cm^{-1}; δ_H (600 MHz, $CDCl_3$, mixture of diastereomers) 10.13 – 9.95 (1H, m, 1′′-N***H***$_2^+$), 8.85 – 8.65 (1H, m, 1′′-N***H***$_2^+$), 8.06 (0.5H, t, *J* = 6.0 Hz, 1-N***H*** isomer 1), 7.98 (0.5H, t, *J* = 6.0 Hz, 1-N***H*** isomer 2), 7.40 – 7.10 (9H, m, Ar***H***), 4.92 (1H, s, 2-***H***), 3.76 – 3.63 (1H, m, 2′′-***H***), 3.62 – 3.50 (1H, m, NH(C***H***H′)), 3.45 – 3.29 (1H, m, NH(CH***H′***)), 3.13 – 2.94 (2H, m, 5′′-***H***$_2$), 2.02 – 1.93 (1H, m, 3′′-***H***H′), 1.92 – 1.80 (2H, m, 4′′-***H***$_2$), 1.65 – 1.52 (1H, m, 3′′-H***H′***); δ_C (151 MHz, $CDCl_3$, mixture of diastereomers) 174.4 (9) (***C***-1), 174.4(8) (***C***-1), 162.3 (q, *J* = 37.5 Hz, TFA ***C***=O), 138.8 (Ar***C***), 138.6 (Ar***C***), 137.7 (Ar***C***), 137.5 (Ar***C***), 133.4(3) (Ar***C***), 133.3(9) (Ar***C***), 130.3(4) (Ar***C***), 130.2(7) (Ar***C***), 129.0 (Ar***C***), 128.9 (Ar***C***), 128.8 (Ar***C***), 128.7 (Ar***C***), 127.7(0) (Ar***C***), 127.6(6) (Ar***C***), 60.7(1) (***C***-2′′), 60.6(9) (***C***-2′′), 57.4(3) (***C***-2), 57.3(8) (***C***-2), 45.4 (***C***-5′′), 45.3 (***C***-5′′), 40.8 (NH(***C***HH′)), 27.6 (***C***-3′′), 24.0 (***C***-4′′); δ_F (376 MHz, $CDCl_3$) −75.88 (C***F***$_3$CO$_2$H); *m/z* (LCMS, ESI$^+$) 329 (M(^{35}Cl)H$^+$), 331 (M(^{37}Cl)H$^+$); Accurate mass: Found MH$^+$, 329.1426: $C_{19}H_{22}N_2O^{35}Cl$ requires *M*, 329.1421.

N-([4′′′-Chlorophenyl]phenylmethyl)-2-(pyrrolidin-2′-yl)acetamide TFA salt ***21***

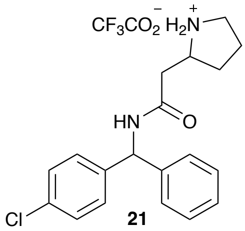

M.p. 189 – 191 °C; ν_{max} (ATR) 3262 (br), 3031 (w), 2951 (w), 2360 (w), 1653 (br, s), 1542 (m), 1491 (m), 1197 (s), 1137 (s) cm^{-1}; δ_H (600 MHz, $CDCl_3$, mixture of diastereomers) 9.17 – 9.06 (1H, m, 1′-N***H***$_2^+$), 8.92 – 8.82 (1H, m, 1′-N***H***$_2^+$), 7.52 (0.5H, d, *J* = 7.0 Hz, 1-N***H*** isomer 1), 7.47 (0.5H, d, *J* = 7.0 Hz, 1-N***H*** isomer 2), 7.34 – 7.24 (5H, m, Ar***H***), 7.20 – 7.12 (4H, m, Ar***H***), 6.03 – 5.97 (1H, m, 1′′-***H***), 3.79 – 3.71 (1H, m, 2′-***H***), 3.21 – 3.13 (1H, m, 5′-***H***H′), 3.13 – 3.05 (1H, m, 5′-H***H′***), 3.01 – 2.94 (1H, m, 2-***H***H′), 2.77 – 2.69 (1H, m, 2-H***H′***), 2.12 – 2.04 (1H, m, 3′-***H***H′), 2.02 – 1.94 (1H, m, 4′-***H***H′), 1.91 – 1.82 (1H, m, 4′-H***H′***), 1.70 – 1.62 (1H, m, 3′-H***H′***); δ_C (151 MHz, $CDCl_3$, mixture of diastereomers) 170.0(4) (***C***-1), 170.0(2) (***C***-1), 161.3 (q, *J* = 38.5 Hz, TFA ***C***=O), 140.3(2) (Ar***C***), 140.3(1) (Ar***C***), 139.4 (Ar***C***), 133.8 (Ar***C***), 133.6 (Ar***C***), 129.1 (Ar***C***), 129.0(3) (Ar***C***), 129.0(0) (Ar***C***), 128.9 (Ar***C***), 128.7 (Ar***C***), 128.2 (Ar***C***), 128.0 (Ar***C***), 127.5 (Ar***C***), 127.2 (Ar***C***), 57.6 (***C***-1′′), 57.5 (***C***-1′′), 57.2 (***C***-2′), 57.1 (***C***-2′), 45.5 (***C***-5′), 36.3(6) (***C***-2), 36.3(5) (***C***-2), 30.0 (***C***-3′), 29.9 (***C***-

3′), 23.7(7) (***C***-4′), 23.7(5) (***C***-4′); δ_F (376 MHz, $CDCl_3$) −76.19 ($C\boldsymbol{F}_3CO_2H$); *m/z* (LCMS, ESI^+) 329 ($M(^{35}Cl)H^+$), 331 ($M(^{37}Cl)H^+$); Accurate mass: Found MH^+, 329.1422: $C_{19}H_{22}N_2O^{35}Cl$ requires *M*, 329.1421.

N-([Tetrahydrofuran-2′′-yl]methyl)-2-(4′-chlorophenyl)-2-phenylacetamide **22**

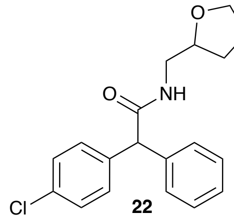

22

Obtained in 66% yield as an off-white solid. m.p. 62 – 64 °C; ν_{max} (ATR) 3312 (br), 3086 (w), 3026 (w), 3026 (w), 2964 (w), 2875 (w), 1650 (s), 1544 (m), 1490 (s), 1448 (w), 1360 (w), 1222 (w), 1086 (m), 1015 (m) cm^{-1}; δ_H (400 MHz, $CDCl_3$, mixture of diastereomers) 7.36 – 7.19 (9H, m, Ar***H***), 6.05 – 5.98 (1H, m, N***H***), 4.87 (0.5H, s, 2-***H***), 4.86 (0.5H, s, 2-***H***), 3.94 (1H, qd, *J* = 7.0, 3.5 Hz, 2′′-***H***), 3.74 – 3.64 (2H, m, 5′′-$\boldsymbol{H}_2$), 3.60 (0.5H, ddd, *J* = 5.5, 3.5, 2.5 Hz, NHC***H***H′ isomer A), 3.56 (0.5H, ddd, *J* = 5.5, 3.5, 2.5 Hz, NHC***H***H′ isomer B), 3.24 (0.5H, ddd, *J* = 7.0, 5.5, 4.5 Hz, NHCH***H′*** isomer A), 3.21 (0.5H, ddd, *J* = 7.0, 5.5, 4.5 Hz, NHCH***H′*** isomer B), 1.98 – 1.85 (1H, m, 3′′-***H***H′), 1.89 – 1.72 (2H, m, 4′′-$\boldsymbol{H}_2$), 1.53 – 1.43 (1H, m, 3′′-H***H′***); δ_C (101 MHz, $CDCl_3$, mixture of diastereomers) 171.6(8) (***C***=O), 171.6(6) (***C***=O), 139.1(2) (Ar***C***), 139.0(8) (Ar***C***), 138.1 (Ar***C***), 138.0 (Ar***C***), 133.2 (***C***-4′), 130.4 (Ar***C***), 130.3 (Ar***C***), 129.0 (Ar***C***), 128.9(3) (Ar***C***), 128.8(8) (Ar***C***), 128.8(5) (Ar***C***), 128.8 (Ar***C***), 127.6 (Ar***C***), 77.7 (***C***-2′′), 77.6 (***C***-2′′), 68.3 (***C***-5′′), 58.6 (***C***-2), 58.5 (***C***-2), 43.4(2) (NH***C***HH′ isomer B), 43.4(1) (NH***C***HH′ isomer A), 28.6(2) (***C***-3′′), 28.6(0) (***C***-3′′), 26.0 (***C***-4′′); *m/z* (LCMS, ESI^+) 330 ($M(^{35}Cl)H^+$), 332 ($M(^{37}Cl)H^+$); Accurate mass: Found MH^+, 330.1261: $C_{19}H_{21}NO_2{}^{35}Cl$ requires *M*, 330.1261.

N-([4′-Chlorophenyl]phenylmethyl)-2-cyclopentylacetamide **23**

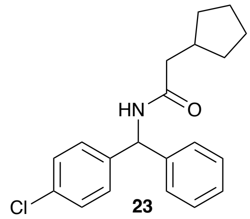

23

Obtained in 79% yield as colourless needles. R_f 0.36 (10% EtOAc in hexanes); M.p 146 – 148 °C; ν_{max} (ATR) 3274 (br), 3030 (w), 2949 (m), 2867 (w), 1637 (s), 1533 (m), 1490 (m), 1366 (m), 1217 (m), 1091 (m) cm^{-1}; δ_H (700 MHz, $CDCl_3$) 7.33 (2H, t, *J* = 7.5 Hz, 3^{Ph}-$\boldsymbol{H}_2$), 7.31 – 7.27 (3H, m, 3′-$\boldsymbol{H}_2$, 4^{Ph}-***H***), 7.19 (2H, d, *J* = 7.5 Hz, 2^{Ph}-$\boldsymbol{H}_2$), 7.16 (2H, d, *J* = 8.0 Hz, 2′-$\boldsymbol{H}_2$), 6.23 (1H, d, *J* = 8.0 Hz, NHC***H***), 5.97 (1H, d, *J* = 8.0 Hz, N***H***CH), 2.29 – 2.23 (3H, m, 2-$\boldsymbol{H}_2$, 1^{Cyp}-***H***), 1.86 – 1.80 (2H, m, 2^{Cyp}-$\boldsymbol{H}_2H'_2$), 1.66 – 1.59 (2H, m, 3^{Cyp}-$\boldsymbol{H}_2H'_2$), 1.59 – 1.52 (2H, m, 3^{Cyp}-$H_2\boldsymbol{H'}_2$), 1.19 – 1.12 (2H, m, 2^{Cyp}-$H_2\boldsymbol{H'}_2$); δ_C (176 MHz, $CDCl_3$) 172.0 (***C***-1), 141.3 (***C***-1^{Ph}), 140.3 (***C***-1′), 133.4 (***C***-4′), 129.0 (Ar***C***), 128.9 (Ar***C***), 128.8 (Ar***C***), 127.9 (***C***-4^{Ph}), 127.6 (***C***-2^{Ph}), 56.5 (NH***C***H), 43.2 (***C***-2), 37.3

(***C***-1[Cyp]), 32.7(7) (***C***-2[Cyp]), 32.7(6) (***C***-2[Cyp]), 25.2 (***C***-3[Cyp]); *m/z* (LCMS, ESI$^+$) 328 (M(^{35}Cl)H$^+$), 330 (M(^{37}Cl)H$^+$); Accurate mass: Found MH$^+$, 328.1484: $C_{20}H_{23}NO^{35}Cl$ requires *M*, 328.1468.

N-(1′′-[2′′′-Dimethylaminoethyl]indazol-6′′-yl)-2-(4′-chlorophenyl)-2-phenylacetamide **24**

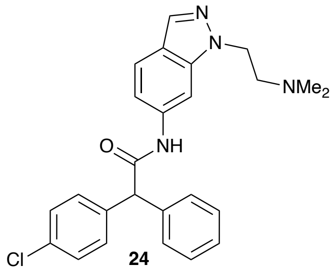

24

Obtained in 9% yield as a yellow oil. ν_{max} (ATR) 3306 (br), 3264 (w), 3070 (w), 2952 (w), 2862 (w), 2818 (w), 2780 (w), 1661 (m), 1625 (m), 1587 (m), 1546 (m), 1489 (s), 1462 (m), 1359 (m), 1310 (m), 1256 (w), 1167 (m), 1092 (m) cm^{-1}; δ_H (600 MHz, $CDCl_3$) 8.23 (1H, br s, 7′′-***H***), 7.91 (1H, s, 3′′-***H***), 7.57 (1H, d, *J* = 8.5 Hz, 4′′-***H***), 7.45 (1H, br s, N***H***), 7.42 – 7.37 (2H, m, 3[Ph]-***H***2), 7.36 – 7.31 (5H, m, 3′-***H***2, 2[Ph]-***H***2, 4[Ph]-***H***), 7.30 – 7.27 (2H, m, 2′-***H***2), 6.68 (1H, dd, *J* = 8.5, 1.5 Hz, 5′′-***H***), 5.08 (1H, s, 2-***H***), 4.43 (2H, t, *J* = 7.0 Hz, 1′′′-***H***2), 2.81 (2H, t, *J* = 7.0 Hz, 2′′′-***H***2), 2.29 (6H, s, N(C***H***3)2); δ_C (151 MHz, $CDCl_3$) 169.9 (***C***=O), 140.1 (***C***-9′′), 138.6 (***C***-1[Ph]), 137.5 (***C***-1′), 136.0 (***C***-6′′), 133.7 (***C***-4′), 133.1 (***C***-3′′), 130.5 (***C***-2′), 129.4 (***C***-3[Ph]), 129.2 (***C***-3′), 129.0 (***C***-2[Ph]), 128.1 (***C***-4[Ph]), 121.7 (***C***-4′′), 121.2 (***C***-8′′), 114.2 (***C***-5′′), 99.4 (***C***-7′′), 59.7 (***C***-2), 58.5 (***C***-2′′′), 47.2 (***C***-1′′′), 45.9 (N(***C***H3)); *m/z* (LCMS, ESI$^+$) 433 (M(^{35}Cl)H$^+$), 435 (M(^{37}Cl)H$^+$); Accurate mass: Found MH$^+$, 433.1769: $C_{25}H_{26}N_4O^{35}Cl$ requires *M*, 433.1795.

1-(2′-[Dimethylamino]ethyl)-6-([{4′′-chlorophenyl}{phenyl}methoxy]methyl)indazole **27**

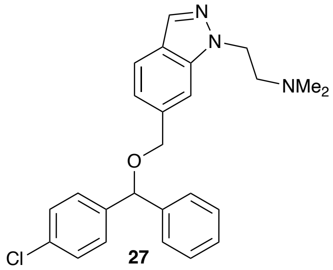

27

Methyl 1H-indazole-6-carboxylate **26** (264 mg, 1.50 mmol) was stirred with K_2CO_3 (622 mg, 4.50 mmol) and 2-chloro-*N*,*N*-dimethylethylamine hydrochloride salt (99%, 327 mg, 2.25 mmol) in DMF (5 mL) at 80 °C for 14 h. The reaction mixture was then cooled and diluted with H_2O (60 mL) prior to washing with EtOAc (6 × 10 mL). After combining the organic fractions, the volatiles were removed under reduced pressure and the crude mixture separated by flash chromatography on silica (10% → 90% EtOAc in hexanes with 1% NEt_3) to afford m*ethyl-1-(2′-[dimethylamino]ethyl)indazole-6-carboxylate* (199 mg, 54%) found as a yellow oil.

To a stirred suspension of $LiAlH_4$ (300 mg, 7.89 mmol) in THF (15 mL) at 0 °C was slowly added a solution of m*ethyl-1-(2′-[dimethylamino]ethyl)indazole-6-carboxylate* (650 mg, 2.63 mmol) in THF (3 mL). After 1 h at RT, the mixture was re-cooled in an ice bath and quenched according to Fieser's method. Subsequent removal of volatiles *in vacuo* afforded the *1-(2′-[dimethylamino]ethyl)-6-(hydroxymethyl)indazole* (577 mg, quantitative) as a pale yellow oil.

To a cooled solution of diarylcarbinol **5a** (139 mg, 0.64 mmol) in DCM (1 mL) was added NEt_3 (116 µL, 0.83 mmol) and methanesulfonyl chloride (59 µL, 0.76 mmol). The mixture was stirred at RT until consumption of starting material was observed by TLC. After dilution with DCM (10 mL), the mixture was washed with H_2O (5 mL), 1 M $HCl_{(aq.)}$ (5 mL) then saturated $NaHCO_{3(aq.)}$ (5 mL), before being dried over $MgSO_4$. Removal of volatiles under reduced pressure afforded the crude benzhydryl chloride **25** which was used immediately.

To a 0 °C solution of NaH (60%, 18 mg, 0.45 mmol) in DMF (0.25 mL) was added *1-(2′-[dimethylamino]ethyl)-6-(hydroxymethyl)indazole* (93 mg, 0.42 mmol) in DMF (0.5 mL). After stirring at RT for 2 h, a solution of crude benzhydryl chloride **25** in DMF (0.5 mL) was added and the reaction mixture heated to 80 °C for 4.5 h. The mixture was re-cooled to 0 °C and quenched by the addition of ice cold H_2O (15 mL) prior to extraction with EtOAc (6 × 5 mL). The combined organic washings were washed with brine (15 mL), concentrated *in vacuo* then purified on SiO_2 column (50% EtOAc in $CHCl_3$ with 1% NEt_3) to furnish the *title compound* (25 mg, 14%) as a clear colourless oil. R_f 0.28 (40% EtOAc in $CHCl_3$ with 1% NEt_3); ν_{max} (ATR) 3063 (w), 3031 (w), 2971 (w), 2861 (w), 2819 (w), 2768 (w), 1489 (m), 1455 (m), 1366 (s), 1217 (s), 1087 (s), 1014 (m) cm^{-1}; δ_H (600 MHz, $CDCl_3$) 7.98 (1H, s, 3-***H***), 7.69 (1H, d, *J* = 8.0 Hz, 4-***H***), 7.41 (1H, s, 7-***H***), 7.39 – 7.26 (9H, m, Ar***H***), 7.13 (1H, d, *J* = 8.0 Hz, 5-***H***), 5.45 (1H, s, Ar_2C***H***), 4.68 (2H, s, $OC\boldsymbol{H}_2$), 4.49 (2H, t, *J* = 7.0 Hz, 1′-$\boldsymbol{H}_2$), 2.85 (2H, t, *J* = 7.0 Hz, 2′-$\boldsymbol{H}_2$), 2.32 (6H, s, $N(C\boldsymbol{H}_3)_2$); δ_C (151 MHz, $CDCl_3$) 141.7 (***C***-1^{Ph}), 140.8 (***C***-1′′), 139.9 (***C***-9), 136.9 (***C***-6), 133.4 (***C***-4′′), 133.2 (***C***-3), 128.7(4) (Ar***C***), 128.7(2) (Ar***C***), 128.6 (Ar***C***), 128.0 (Ar***C***), 127.2 (Ar***C***), 123.8 (***C***-8), 121.3 (***C***-4), 120.8 (***C***-5), 107.9 (***C***-7), 82.1 ($Ar_2\boldsymbol{C}H$), 71.0 ($O\boldsymbol{C}H_2$), 58.5 (***C***-2′), 47.1 (***C***-1′), 45.7 ($N(\boldsymbol{C}H_3)_2$); *m/z* (LCMS, ESI^+) 420 ($M(^{35}Cl)H^+$), 422 ($M(^{37}Cl)H^+$); Accurate mass: Found MH^+, 420.1845: $C_{25}H_{27}N_3O^{35}Cl$ requires *M*, 420.1843.

N-(Diphenylmethyl)-2-(4′-chlorophenyl)-2-phenylacetamide **S1**

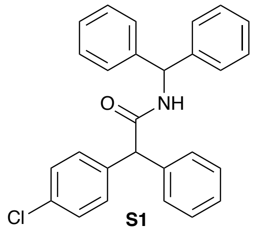

Obtained in 67% yield as an off-white solid. M.p. 196 – 199 °C; ν_{max} (ATR) 3270 (br), 3070 (w), 3021 (w), 1647 (s), 1490 (m), 1481 (m), 1368 (w), 1222 (w) cm^{-1}; δ_H (600 MHz, $CDCl_3$) 7.35 – 7.26 (11H, m, 3′-$\boldsymbol{H}_2$,

$3^{Ph'}$-($\boldsymbol{H}_2$)$_2$, $4^{Ph'}$-($\boldsymbol{H}$)$_2$, 3^{Ph}-$\boldsymbol{H}_2$, 4^{Ph}-$\boldsymbol{H}$), 7.22 (2H, d, *J* = 7.5 Hz, 2^{Ph}-$\boldsymbol{H}_2$), 7.18 (2H, d, *J* = 8.0 Hz, 2′-$\boldsymbol{H}_2$), 7.11 (4H, d, *J* = 8.0 Hz, $2^{Ph'}$-($\boldsymbol{H}_2$)$_2$), 6.29 (1H, d, *J* = 8.0 Hz, NHC***H***Ph$_2$), 6.17 (1H, d, *J* = 8.0 Hz, N***H***CHPh$_2$), 4.93 (1H, s, 2-***H***); δ_C (151 MHz, CDCl$_3$) 170.6 (***C***=O), 141.3(3) (***C***-$1^{Ph'}$), 141.3(0) (***C***-$1^{Ph'}$), 139.0 (***C***-1^{Ph}), 137.9 (***C***-1′), 133.4 (***C***-4′), 130.4 (***C***-2′), 129.1 (Ar***C***), 129.0 (Ar***C***), 128.9 (Ar***C***), 128.8 (Ar***C***), 127.8 (Ar***C***), 127.7 (Ar***C***), 127.4 (***C***-$2^{Ph'}$), 58.5 (***C***-2), 57.3 (NH***C***HPh$_2$); *m/z* (LCMS, ESI$^+$) 412 (M(^{35}Cl)H$^+$), 414 (M(^{37}Cl)H$^+$); Accurate mass: Found MH$^+$, 412.1473: $C_{27}H_{23}NO^{35}Cl$ requires *M*, 412.1468.

1-(4′′-[{1′′′-Methylimidazol-2′′′-yl}methyl]piperazin-1′′-yl)-2-(4′-chlorophenyl)-2-phenylethan-1-one **S2**

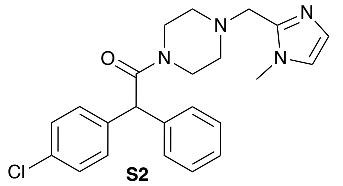

S2

Obtained in 37% yield as an orange-brown oil. ν_{max} (ATR) 3061 (w), 3026 (w), 2942 (w), 2817 (w), 1640 (s), 1434 (m), 1366 (m), 1267 (m), 1199 (s), 1126 (s), 1032 (m) cm^{-1}; δ_H (600 MHz, CDCl$_3$) 7.34 – 7.30 (2H, m, 3^{Ph}-$\boldsymbol{H}_2$), 7.28 – 7.24 (3H, m, 3′-$\boldsymbol{H}_2$, 4^{Ph}-$\boldsymbol{H}$), 7.21 – 7.17 (2H, m, 2^{Ph}-$\boldsymbol{H}_2$), 7.14 – 7.10 (3H, m, 2′-$\boldsymbol{H}_2$, 4′′′-***H***), 6.94 (1H, d, *J* = 1.0 Hz, 5′′′-***H***), 5.13 (1H, s, 2-***H***), 3.81 – 3.70 (3H, m, 2′′-***H***H′, NC$\boldsymbol{H}_2$), 3.77 (3H, s, NC$\boldsymbol{H}_3$), 3.68 – 3.60 (1H, m, 2′′-H***H′***), 3.43 – 3.34 (2H, m, 2′′-$\boldsymbol{H}_2$), 2.55 – 2.45 (2H, m, 3′′-$\boldsymbol{H}_2$(HH′)), 2.33 – 2.27 (1H, m, 3′′-H$_2$(***H***H′)), 2.23 – 2.16 (1H, m, 3′′-H$_2$(H***H′***)); δ_C (151 MHz, CDCl$_3$) 170.0 (***C***=O), 143.7 (***C***-2′′′), 138.7 (***C***-1^{Ph}), 138.1 (***C***-1′), 133.1 (***C***-4′), 130.6 (***C***-2′), 129.0 (***C***-3^{Ph}), 128.8(2) (Ar***C***), 128.7(8) (Ar***C***), 127.5 (Ar***C***), 123.7 (***C***-4′′′), 122.4 (***C***-5′′′), 54.4 (***C***-2), 52.9 (***C***-3′′), 52.6 (N***C***H$_2$), 52.5 (***C***-3′′), 45.8 (***C***-2′′), 42.1 (***C***-2′′), 33.9 (N***C***H$_3$); *m/z* (LCMS, ESI$^+$) 409 (M(^{35}Cl)H$^+$), 411 (M(^{37}Cl)H$^+$); Accurate mass: Found MH$^+$, 409.1799: $C_{23}H_{26}N_4O^{35}Cl$ requires *M*, 409.1795.

N-(8′′-Azabicyclo[3.2.1]octan-3′′-yl)-2-(4′-chlorophenyl)-2-phenylacetamide **S3**

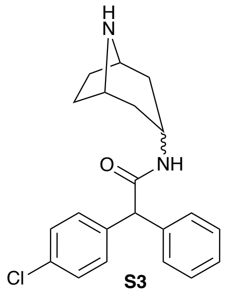

S3

Obtained in 17% yield as a white solid. M.p. 189 – 191 °C (decomp.); ν_{max} (ATR) 3278 (br), 3066 (w), 2958 (w), 2876 (w), 1644 (s), 1550 (m), 1490 (s), 1408 (w), 1312 (w), 1226 (w), 1162 (w), 1090 (m), 1015 (m) cm^{-1}; δ_H (600 MHz, CDCl$_3$) 7.33 – 7.28 (2H, m, $3^{Ph}$$\boldsymbol{H}_2$), 7.28 – 7.23 (3H, m, 3′-$\boldsymbol{H}_2$, 4^{Ph}-$\boldsymbol{H}$), 7.20 – 7.17 (2H, m, 2^{Ph}-$\boldsymbol{H}_2$), 7.17 – 7.15 (2H, m, 2′-$\boldsymbol{H}_2$), 5.36 (1H, d, *J* = 8.5 Hz, N***H***), 4.79 (1H, s, 2-***H***), 4.21 (1H, tdt, *J* = 11.5, 8.5, 5.5 Hz, 3′′-***H***), 3.53 – 3.49 (2H, m, 1′′-$\boldsymbol{H}_2$), 1.92 – 1.83 (2H, m, 2′′-$\boldsymbol{H}_2$H′$_2$), 1.78 – 1.73 (4H, m, 6′′-($\boldsymbol{H}_2$)$_2$),

1.72 – 1.61 (1H, m, 8′′-***H***), 1.29 – 1.21 (2H, m, 2′′-H_2***H′***$_2$); δ_C (151 MHz, $CDCl_3$) 170.7 (***C***=O), 139.1 (***C***-1^{Ph}), 138.1 (***C***-1′), 133.2 (***C***-4′), 130.3 (***C***-2′), 129.0 (***C***-3^{Ph}), 128.9 (***C***-3′), 128.8 (***C***-2^{Ph}), 127.6 (***C***-4^{Ph}), 58.5 (***C***-2), 54.6 (***C***-1′′), 42.1 (***C***-3′′), 39.8(3) (***C***-2′′), 39.8(1) (***C***-2′′), 29.5 (***C***-6′′); *m/z* (LCMS, ESI^+) 355 ($M(^{35}Cl)H^+$), 357 ($M(^{37}Cl)H^+$); Accurate mass: Found MH^+, 355.1563: $C_{21}H_{24}N_2O^{35}Cl$ requires *M*, 355.1577.

N-(2′′-[2′′′-Dimethylaminoethyl]indazol-6′′-yl)-2-(4′-chlorophenyl)-2-phenylacetamide **S4**

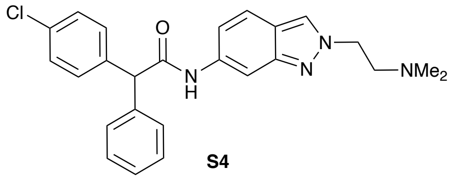

S4

Obtained in 36% yield as a light brown solid. m.p. 76 – 79 °C; ν_{max} (ATR) 3292 (br), 3046 (w), 2950 (w), 2838 (w), 2790 (w), 1672 (m), 1569 (s), 1488 (s), 1368 (m), 1226 (m), 1148 (w), 1088 (w), 1014 (w) cm^{-1}; δ_H (600 MHz, $CDCl_3$) 7.94 (1H, s, 3′′-***H***), 7.93 (1H, br s, 7′′-***H***), 7.55 (1H, d, *J* = 9.0 Hz, 4′′-***H***), 7.40 – 7.36 (2H, m, 3^{Ph}-***H***$_2$), 7.35 – 7.31 (5H, m, 3′-***H***$_2$, 2^{Ph}-***H***$_2$, 4^{Ph}-***H***), 7.30 – 7.27 (2H, m, 2′-***H***$_2$), 7.02 (1H, dd, *J* = 9.0, 2.0 Hz, 5′′-***H***), 5.06 (1H, s, 2-***H***), 4.46 (2H, t, *J* = 6.5 Hz, 1′′′-***H***$_2$), 2.87 (2H, t, *J* = 6.5 Hz, 2′′′-***H***$_2$), 2.27 (6H, s, N(C***H***$_3$)$_2$); δ_C (151 MHz, $CDCl_3$) 169.6 (***C***=O), 148.9 (***C***-9′′), 138.9 (***C***-1^{Ph}), 137.8 (***C***-1′), 135.3 (***C***-6′′), 133.5 (***C***-4′), 130.5 (***C***-2′), 129.3 (***C***-3^{Ph}), 129.1 (***C***-3′), 129.0 (***C***-2^{Ph}), 127.9 (***C***-4^{Ph}), 123.5 (***C***-3′′), 121.0 (***C***-4′′), 119.5 (***C***-8′′), 117.1 (***C***-5′′), 106.7 (***C***-7′′), 59.6 (***C***-2), 59.4 (***C***-2′′′), 52.0 (***C***-1′′′), 45.8 (N(***C***H_3)$_2$); *m/z* (LCMS, ESI^+) 433 ($M(^{35}Cl)H^+$), 435 ($M(^{37}Cl)H^+$); Accurate mass: Found MH^+, 433.1783: $C_{25}H_{26}N_4O^{35}Cl$ requires *M*, 433.1795.

N-(2′′-[4′′′-Methylpiperazin-1′′′-yl]pyrimidin-5′′-yl)-2-(4′-chlorophenyl)-2-phenylacetamide **S5**

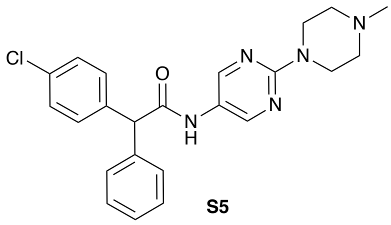

S5

Obtained in 36% yield as an off-white solid. M.p. 152 – 154 °C; ν_{max} (ATR) 3270 (br), 2942 (w), 2846 (w), 2800 (w), 1655 (m), 1606 (m), 1498 (br, s), 1447 (m), 1407 (m), 1360 (s), 1300 (m), 1261 (m), 1170 (w), 1096 (w) cm^{-1}; δ_H (600 MHz, $CDCl_3$) 8.37 (2H, s, 4′′-***H***$_2$), 7.39 – 7.35 (2H, m, 3^{Ph}-***H***$_2$), 7.34 – 7.31 (3H, m, 3′-***H***$_2$, 4^{Ph}-***H***), 7.31 – 7.28 (2H, m, 2^{Ph}-***H***$_2$), 7.26 – 7.23 (2H, m, 2′-***H***$_2$), 7.01 (1H, s, N***H***), 5.01 (1H, s, 2-***H***), 3.80 (4H, t, *J* = 5.0 Hz, 2′′′-(***H***$_2$)$_2$), 2.43 (4H, t, *J* = 5.0 Hz, 3′′′-(***H***$_2$)$_2$), 2.32 (3H, s, NC***H***$_3$); δ_C (151 MHz, $CDCl_3$) 170.2 (***C***=O), 159.7 (***C***-2′′), 151.6 (***C***-4′′), 138.6 (***C***-1^{Ph}), 137.5 (***C***-1′), 133.7 (***C***-4′), 130.4 (***C***-2′), 129.3 (***C***-3^{Ph}), 129.2 (***C***-3′), 128.9 (***C***-2^{Ph}), 128.0 (***C***-4^{Ph}), 122.3 (***C***-5′′), 58.8 (***C***-2), 55.0 (***C***-3′′′), 46.4 (N***C***H_3), 44.2 (***C***-2′′′); *m/z* (LCMS, ESI^+) 422 ($M(^{35}Cl)H^+$), 424 ($M(^{37}Cl)H^+$); Accurate mass: Found MH^+, 422.1757: $C_{23}H_{25}N_5O^{35}Cl$ requires *M*, 422.1748.

N-([1′′H-Imidazol-2′′-yl]methyl)-2-(4′-chlorophenyl)-2-phenylacetamide **S6**

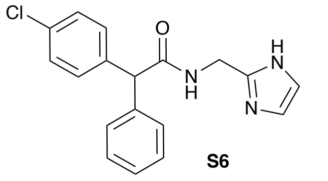

S6

Obtained in 25% yield as a beige solid. M.p. 127 – 130 °C; ν_{max} (ATR) 3232 (br), 3032 (w), 2930 (w), 1656 (s), 1558 (w), 1490 (s), 1420 (w), 1133 (s), 1091 (s), 1015 (m) cm^{-1}; δ_H (600 MHz, $CDCl_3$) 9.14 – 9.08 (1H, m, N***H***), 7.26 – 7.23 (3H, m, 3^{Ph}-***H***$_2$, 4^{Ph}-***H***), 7.22 – 7.19 (2H, m, 3′-***H***$_2$), 7.11 – 7.07 (2H, m, 2^{Ph}-***H***$_2$), 7.07 – 7.04 (2H, m, 2′-***H***$_2$), 6.89 (2H, s, 4′′-(***H***)$_2$,), 4.83 (1H, s, 2-***H***), 4.46 (2H, d, *J* = 6.0 Hz, NHC***H***$_2$); δ_C (151 MHz, $CDCl_3$) 174.3 (***C***=O), 145.2 (***C***-2′′), 138.6 (***C***-1^{Ph}), 137.4 (***C***-1′), 133.4 (***C***-4′), 130.2 (***C***-2′), 128.9(1) (***C***-3^{Ph}), 128.8(8) (***C***-3′), 128.7 (***C***-2^{Ph}), 127.7 (***C***-4^{Ph}), 120.4 (***C***-4′′), 57.2 (***C***-2), 35.9 (NH***C***H$_2$); *m/z* (LCMS, ESI$^+$) 326 (M(^{35}Cl)H$^+$), 328 (M(^{37}Cl)H$^+$); Accurate mass: Found MH$^+$, 326.1050: $C_{18}H_{17}N_3O^{35}Cl$ requires *M*, 326.1060.

N-([Benzothiazol-2′′-yl]methyl)-2-(4′-chlorophenyl)-2-phenylacetamide **S7**

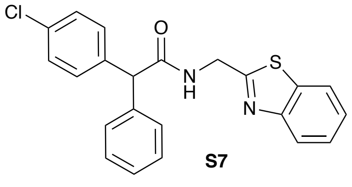

S7

Obtained in 23% yield as a beige solid. M.p. 161 – 163 °C; ν_{max} (ATR) 3294 (br), 3062 (w), 3018 (w), 1654 (s), 1528 (m), 1489 (s), 1412 (w), 1334 (w), 1218 (w), 1156 (w), 1090 (m), 1015 (m) cm^{-1}; δ_H (600 MHz, $CHCl_3$) 7.94 (1H, d, *J* = 8.0 Hz, 7′′-***H***), 7.85 (1H, d, *J* = 8.0 Hz, 4′′-***H***), 7.47 (1H, t, *J* = 8.0 Hz, 5′′-***H***), 7.39 (1H, t, *J* = 8.0 Hz, 6′′-***H***), 7.37 – 7.30 (2H, m, 3^{Ph}-***H***$_2$), 7.33 – 7.26 (5H, m, 3′-***H***$_2$, 2^{Ph}-***H***$_2$, 4^{Ph}-***H***), 7.28 – 7.22 (2H, m, 2′-***H***$_2$), 6.59 (1H, t, *J* = 6.0 Hz, N***H***), 4.98 (1H, s, 2-***H***), 4.89 – 4.85 (2H, m, NHC***H***$_2$); δ_C (151 MHz, $CDCl_3$) 171.8 (***C***=O), 167.9 (***C***-2′′), 152.8 (***C***-8′′), 138.7 (***C***-1^{Ph}), 137.7 (***C***-1′), 135.3 (***C***-9′′), 133.5 (***C***-4′), 130.5 (***C***-2′), 129.2 (***C***-3^{Ph}), 129.1 (Ar***C***), 129.0 (Ar***C***), 127.8 (***C***-4^{Ph}), 126.4 (***C***-5′′), 125.5 (***C***-6′′), 123.0 (***C***-7′′), 121.9 (***C***-4′′), 58.4 (***C***-2), 42.3 (NH***C***H$_2$); *m/z* (LCMS, ESI$^+$) 393 (M(^{35}Cl)H$^+$), 395 (M(^{37}Cl)H$^+$); Accurate mass: Found MH$^+$, 393.0827: $C_{22}H_{18}N_2OS^{35}Cl$ requires *M*, 393.0828.

N-(3′′-[Imidazol-1′′′-yl]prop-1′′-yl)-2-(4′-chlorophenyl)-2-phenylacetamide **S8**

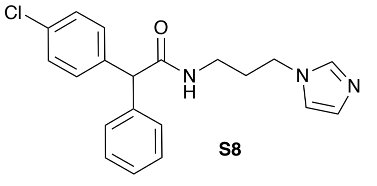

S8

Obtained in 42% yield as a white solid. M.p. 116 – 118 °C; ν_{max} (ATR) 3312 (br), 3044 (w), 2952 (w), 1650 (s), 1560 (m), 1489 (s), 1458 (w), 1352 (w), 1226 (m), 1098 (w) cm^{-1}; δ_H (700 MHz, $CDCl_3$) 7.58 (1H, s, 2′′′-

H), 7.32 (2H, t, *J* = 7.5 Hz, 3^{Ph}-***H***$_2$), 7.28 (2H, d, *J* = 8.0 Hz, 3′-***H***$_2$), 7.26 – 7.23 (3H, m, 2^{Ph}-***H***$_2$, 4^{Ph}-***H***), 7.20 (2H, d, *J* = 8.0 Hz, 2′-***H***$_2$), 7.02 (1H, apparent s, 4‴-***H***), 6.89 (1H, apparent s, 5‴-***H***), 6.31 (1H, t, *J* = 6.5 Hz, N***H***), 4.86 (1H, s, 2-***H***), 3.93 (2H, t, *J* = 7.0 Hz, 3″-***H***$_2$), 3.25 (2H, q, *J* = 6.5 Hz, 1″-***H***$_2$), 1.97 (2H, tt, *J* = 7.0, 6.5 Hz, 2″-***H***$_2$); δ_C (176 MHz, $CDCl_3$) 172.2 (***C***=O), 139.1 (***C***-1^{Ph}), 137.9 (***C***-1′), 137.1 (***C***-2‴), 133.3 (***C***-4′), 130.3 (***C***-2′), 129.1 (***C***-3^{Ph}), 129.0 (***C***-3′), 128.8 (***C***-2^{Ph}), 128.7 (***C***-4‴), 119.2 (***C***-5‴), 58.2 (***C***-2), 44.9 (***C***-3″), 37.0 (***C***-1″), 31.0 (***C***-2″); *m/z* (LCMS, ESI^+) 354 ($M(^{35}Cl)H^+$), 356 ($M(^{37}Cl)H^+$); Accurate mass: Found MH^+, 354.1383: $C_{20}H_{21}N_3O^{35}Cl$ requires *M*, 354.1373.

N-([N-Methylpyrazol-5″-yl]methyl)-2-(4′-chlorophenyl)-2-phenylacetamide **S9**

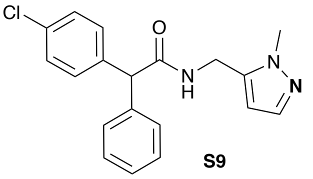

Obtained in 37% yield as a white solid. M.p. 148 – 150 °C; ν_{max} (ATR) 3274 (br), 3030 (w), 2934 (w), 1648 (s), 1544 (m), 1490 (s), 1400 (w), 1344 (w), 1280 (w), 1218 (w), 1090 (m), 1015 (m) cm^{-1}; δ_H (700 MHz, $CDCl_3$) 7.37 (1H, d, *J* = 2.0 Hz, 3″-***H***), 7.36 – 7.33 (2H, m, 3^{Ph}-***H***$_2$), 7.31 – 7.27 (3H, m, 3′-***H***$_2$, 4^{Ph}-***H***), 7.24 – 7.22 (2H, m, 2^{Ph}-***H***$_2$), 7.21 – 7.18 (2H, m, 2′-***H***$_2$), 6.05 (1H, d, *J* = 2.0 Hz, 4″-***H***), 5.79 (1H, t, *J* = 5.5 Hz, N-***H***), 4.87 (1H, s, 2-***H***), 4.52 (2H, d, *J* = 5.5 Hz, NHC***H***$_2$), 3.77 (3H, s, NC***H***$_3$); δ_C (101 MHz, $CDCl_3$) 171.4 (***C***=O), 138.6(3) (Ar***C***), 138.5(9) (Ar***C***), 138.4 (***C***-3″), 137.6 (***C***-1′), 133.5 (***C***-4′), 130.3 (***C***-2′), 129.1 (Ar***C***), 129.0 (Ar***C***), 128.7 (***C***-2^{Ph}), 127.9 (***C***-4^{Ph}), 105.8 (***C***-4″), 58.3 (***C***-2), 36.6 (N***C***H_3), 34.6 (NH***C***H_2); *m/z* (LCMS, ESI^+) 340 ($M(^{35}Cl)H^+$), 342 ($M(^{37}Cl)H^+$); Accurate mass: Found MH^+, 340.1226: $C_{19}H_{19}N_3O^{35}Cl$ requires *M*, 340.1217.

N-Methyl-N-([2″-methyloxazol-4″-yl]methyl)-2-(4′-chlorophenyl)-2-phenylacetamide **S10**

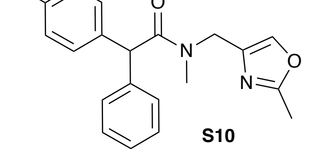

Obtained in 49% yield as a yellow oil. ν_{max} (ATR) 3050 (w), 2928 (w), 1643 (s), 1580 (m), 1489 (s), 1397 (m), 1278 (w), 1204 (w), 1089 (s), 1015 (m) cm^{-1}; δ_H (600 MHz, $CDCl_3$, mixture of rotamers) 7.42 (0.6H, s, 5″-***H*** rotamer B), 7.32 – 7.28 (2H, m, 3^{Ph}-***H***$_2$), 7.27 – 7.23 (4.2H, m, 3′-***H***$_2$, 2^{Ph}-***H***$_2$ rotamer A, 4^{Ph}-***H***, 5″-***H*** rotamer A), 7.21 – 7.18 (2H, m, 2′-***H***$_2$ rotamer A, 2^{Ph}-***H***$_2$ rotamer B), 7.15 – 7.12 (1.2H, m, 2′-***H***$_2$, rotamer B), 5.49 (0.4H, s, 2-***H*** rotamer A), 5.15 (0.6H, s, 2-***H*** rotamer B), 4.45 (1.2H, s, $N(CH_3)C$***H***$_2$ rotamer B), 4.36 (0.4H, d, *J* = 17.0 Hz, $N(CH_3)C$***H***H′ rotamer A), 4.21 (0.4H, d, *J* = 17.0 Hz, $N(CH_3)H$***H***′ rotamer A), 3.05 (1.8H, s, N(C***H***$_3$)CH_2 rotamer B), 3.00 (1.2H, s, N(C***H***$_3$)CHH′ rotamer A), 2.46 (1.2H, s, 2″-C***H***$_3$ rotamer A), 2.41 (1.8H, s, 2″-C***H***$_3$ rotamer B); δ_C (151 MHz, $CDCl_3$, mixture of rotamers) 171.8 (***C***=O rotamer A), 171.4 (***C***=O

rotamer B), 162.5 (***C***-2′′ rotamer A), 161.5 (***C***-2′′ rotamer B), 139.1 (***C***-1^{Ph} rotamer A), 138.9 (***C***-1^{Ph} rotamer B), 138.5 (***C***-1′ rotamer A), 138.2 (***C***-1′ rotamer B), 137.0 (***C***-4′′ rotamer A), 136.8 (***C***-4′′ rotamer B), 136.2 (***C***-5′′ rotamer B), 135.1 (***C***-5′′ rotamer A), 133.0(4) (***C***-4′ rotamer B), 133.0(1) (***C***-4′ rotamer A), 130.7 (***C***-2′ rotamer A), 130.6 (***C***-2′ rotamer B), 129.0 (Ar***C***), 128.9(1) (Ar***C***), 128.8(8) (Ar***C***), 128.8(6) (Ar***C***), 128.7(1) (Ar***C***), 128.6(5) (Ar***C***), 127.4(1) (***C***-4^{Ph} rotamer A), 127.3(8) (***C***-4^{Ph} rotamer B), 54.3 (***C***-2 rotamer B), 53.8 (***C***-2 rotamer A), 46.1 (N(CH_3)***C***HH′ rotamer A), 44.0 (N(CH_3)***C***H_2 rotamer B), 36.2 (N(***C***H_3)CH_2 rotamer B), 34.3 (N(***C***H_3)CHH′ rotamer A), 14.1 (2′′-***C***H_3 rotamer A), 14.0 (2′′-***C***H_3 rotamer B); *m/z* (LCMS, ESI$^+$) 355 (M(^{35}Cl)H$^+$), 357 (M(^{37}Cl)H$^+$); Accurate mass: Found MH$^+$, 355.1220: $C_{20}H_{20}N_2O_2{}^{35}Cl$ requires *M*, 355.1213.

N-([Pyridin-2′′-yl]methyl)-2-(4′-chlorophenyl)-2-phenylacetamide **S11**

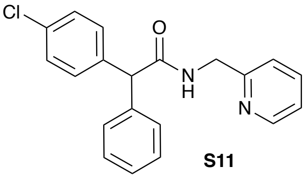

S11

Obtained in 70% yield as a white solid. M.p. 119 – 120 °C; ν_{max} (ATR) 3308 (br), 3056 (w), 2930 (w), 1649 (s), 1600 (w), 1591 (m), 1490 (s), 1437 (w), 1204 (w), 1091 (m), 1016 (m) cm^{-1}; δ_H (700 MHz, $CDCl_3$) 8.48 – 8.46 (1H, m, 6′′-***H***), 7.64 (1H, td, *J* = 7.5, 2.0 Hz, 5′′-***H***), 7.35 – 7.31 (2H, m, 3^{Ph}-***H***$_2$), 7.30 – 7.26 (5H, m, 3′-***H***$_2$, 2^{Ph}-***H***$_2$, 4^{Ph}-***H***), 7.26 – 7.24 (2H, m, 2′-***H***$_2$), 7.23 – 7.21 (1H, m, 3′′-***H***), 7.20 – 7.16 (1H, m, 4′′-***H***), 6.97 – 6.92 (1H, m, N-***H***), 4.96 (1H, s, 2-***H***), 4.59 (2H, d, *J* = 5.0 Hz, NHC***H***$_2$); δ_C (176 MHz, $CDCl_3$) 171.6 (***C***=O), 156.1 (***C***-2′′), 149.1 (***C***-6′′), 139.2 (***C***-1^{Ph}), 138.2 (***C***-1′), 136.9 (***C***-5′′), 133.2 (***C***-4′), 130.4 (***C***-2′), 129.0 (***C***-3^{Ph}), 128.9(3) (Ar***C***), 128.9(1) (Ar***C***), 127.6 (***C***-4^{Ph}), 122.6 (***C***-4′′), 122.2 (***C***-3′′), 58.5 (***C***-2), 44.8 (NH***C***H_2); *m/z* (LCMS, ESI$^+$) 337 (M(^{35}Cl)H$^+$), 339 (M(^{37}Cl)H$^+$); Accurate mass: Found MH$^+$, 337.1111: $C_{20}H_{18}N_2O^{35}Cl$ requires *M*, 337.1108.

N-([Azetidin-3′′-yl]methyl)-2-(4′-chlorophenyl)-2-phenylacetamide **S12**

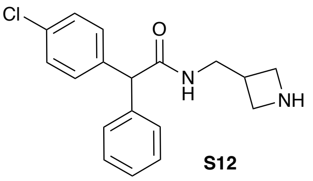

S12

Obtained in 10% yield as a yellow solid. M.p. 102 – 105 °C; ν_{max} (ATR) 3284 (br), 3066 (w), 2956 (w), 2924 (w), 2866 (w), 1649 (s), 1555 (m), 1490 (s), 1364 (w), 1226 (w), 1098 (w), 1018 (w) cm^{-1}; δ_H (700 MHz, $CDCl_3$) 7.35 – 7.30 (2H, m, 3^{Ph}-***H***$_2$), 7.30 – 7.26 (3H, m, 3′-***H***$_2$, 4^{Ph}-***H***), 7.25 – 7.22 (2H, m, 2^{Ph}-***H***$_2$), 7.22 – 7.19 (2H, m, 2′-***H***$_2$), 6.21 – 6.13 (1H, m, N***H***), 4.88 (1H, s, 2-***H***), 3.72 – 3.63 (2H, m, 2′′-***H***$_2$H′$_2$), 3.47 (2H, t, *J* = 6.0 Hz, NHC***H***$_2$), 3.29 – 3.20 (2H, m, 2′′-H_2***H***′$_2$), 2.86 – 2.78 (1H, m, 3′′-***H***), 2.04 – 1.87 (1H, m, 1′′-***H***); δ_C (176 MHz, $CDCl_3$) 172.0 (***C***=O), 139.2 (***C***-1^{Ph}), 138.1 (***C***-1′), 133.3 (***C***-4′), 130.4 (***C***-2′), 129.0 (***C***-3^{Ph}), 128.9 (***C***-3′), 128.8

(***C***-2^{Ph}), 127.6 (***C***-4^{Ph}), 58.6 (***C***-2), 50.9 (***C***-2′′), 43.0 (NH***C***H$_2$), 34.5 (***C***-3′′); *m/z* (LCMS, ESI$^+$) 315 (M(^{35}Cl)H$^+$), 317 (M(^{37}Cl)H$^+$); Accurate mass: Found MH$^+$, 315.1257: $C_{18}H_{20}N_2O^{35}Cl$ requires *M*, 315.1264.

1-(2′′,6′′-Diazaspiro[3.3]heptan-2′′-yl)-2-(4′-chlorophenyl)-2-phenylethan-1-one **S13**

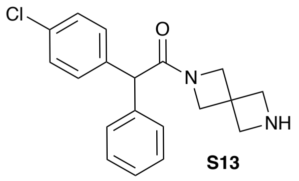

S13

Obtained in 38% yield as a white crystalline solid. M.p. 90 – 92 °C; ν_{max} (ATR) 2942 (w), 2866 (w), 1645 (s), 1490 (m), 1437 (s), 1326 (w), 1290 (w), 1152 (w), 1092 (w), 1015 (w) cm^{-1}; δ_H (700 MHz, CDCl$_3$) 7.33 – 7.29 (2H, m, 3^{Ph}-***H***$_2$), 7.28 – 7.25 (5H, m, 3′-***H***$_2$, 2^{Ph-}***H***$_2$, 4^{Ph}-***H***), 7.24 – 7.21 (2H, m, 2′-***H***$_2$), 4.77 (1H, s, 2-***H***), 4.22 (1H, d, *J* = 9.0 Hz, 1′′-(***H***H′)(H$_2$)), 4.15 (1H, d, *J* = 9.0 Hz, 1′′-(H***H′***)(H$_2$)), 4.11 (2H, s, 1′′-(HH′)***H***$_2$), 3.77 (1H, d, *J* = 8.5 Hz, 5′′-(***H***H′)(HH′)), 3.75 (1H, d, *J* = 8.5 Hz, 5′′-(HH′)(***H***H′)), 3.68 (1H, d, *J* = 8.5 Hz, 5′′-(H***H′***)(HH′)), 3.65 (1H, d, *J* = 8.5 Hz, 5′′-(HH′)(H***H′***)); δ_C (176 MHz, CDCl$_3$) 171.0 (***C***=O), 138.6 (***C***-1^{Ph}), 137.6 (***C***-1′), 133.2 (***C***-4′), 130.4 (***C***-2′), 128.9 (***C***-3^{Ph}), 128.7(9) (Ar***C***), 128.7(7) (Ar***C***), 127.5 (***C***-4^{Ph}), 61.5 (***C***-1′′), 58.6 (***C***-1′′), 57.6(4) (Al***C***), 57.5(5) (Al***C***), 52.9 (***C***-2), 37.4 (***C***-4′′); *m/z* (LCMS, ESI$^+$) 327 (M(^{35}Cl)H$^+$), 329 (M(^{37}Cl)H$^+$); Accurate mass: Found MH$^+$, 327.1248: $C_{19}H_{20}N_2O^{35}Cl$ requires *M*, 327.1264.

N-(3′′-[Pyrrolidin-1′′′-yl]prop-1′′-yl)-2-(4′-chlorophenyl)-2-phenylacetamide **S14**

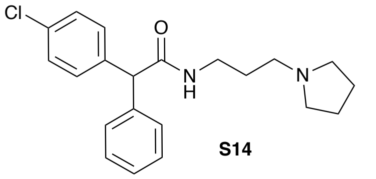

S14

Obtained in 41% yield as a yellow oil. ν_{max} (ATR) 3278 (br), 3064 (w), 2968 (w), 2930 (w), 2880 (w), 2788 (w), 1654 (s), 1548 (m), 1494 (s), 1452 (w), 1352 (w), 1222 (w), 1176 (w), 1096 (w), 1015 (w) cm^{-1}; δ_H (700 MHz, CDCl$_3$) 7.45 – 7.41 (1H, m, N***H***), 7.34 – 7.30 (2H, m, Ar***H***), 7.29 – 7.26 (4H, m, Ar***H***), 7.26 – 7.23 (3H, m, Ar***H***), 4.81 (1H, s, 2-***H***), 3.42 – 3.38 (2H, m, 1′′-***H***$_2$), 2.61 (2H, t, *J* = 6.5 Hz, 3′′-***H***$_2$), 2.60 – 2.54 (4H, m, 2′′′-(***H***$_2$)$_2$), 1.79 – 1.72 (6H, m, 2′′-***H***$_2$, 3′′′-(***H***$_2$)$_2$); δ_C (176 MHz, CDCl$_3$) 171.8 (***C***=O), 139.5 (Ar***C***), 138.4 (Ar***C***), 133.1 (***C***-4′), 130.4 (Ar***C***), 128.9 (Ar***C***), 128.8(4) (Ar***C***), 128.8(1) (Ar***C***), 127.4 (Ar***C***), 58.6 (***C***-2), 54.8 (***C***-3′′), 54.1 (***C***-2′′′), 39.3 (***C***-1′′), 26.6 (***C***-2′′), 23.5 (***C***-3′′′); *m/z* (LCMS, ESI$^+$) 357 (M(^{35}Cl)H$^+$), 358 (M(^{37}Cl)H$^+$); Accurate mass: Found MH$^+$, 357.1741: $C_{21}H_{26}N_2O^{35}Cl$ requires *M*, 357.1734.

N-(3″-[Morpholin-4‴-yl]prop-1″-yl)-2-(4′-chlorophenyl)-2-phenylacetamide **S15**

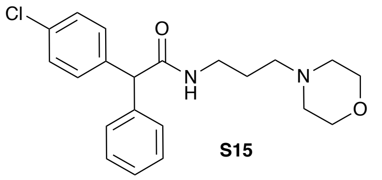

S15

Obtained in 47% yield as a white solid. m.p. 129 – 131 °C; ν_{max} (ATR) 3290 (br), 3054 (w), 2942 (w), 2852 (w), 2808 (w), 1645 (s), 1544 (m), 1489 (s), 1456 (w), 1356 (w), 1276 (w), 1222 (w), 1117 (s), 1090 (w), 1015 (m) cm^{-1}; δ_H (700 MHz, $CDCl_3$) 7.34 – 7.30 (2H, m, 3^{Ph}-$\boldsymbol{H}_2$), 7.29 – 7.26 (3H, m, 3′-$\boldsymbol{H}_2$, 4^{Ph}-***H***), 7.26 – 7.24 (2H, m, 2^{Ph}-$\boldsymbol{H}_2$), 7.22 – 7.19 (2H, m, 2′-$\boldsymbol{H}_2$), 6.82 (1H, t, *J* = 5.0 Hz, N***H***), 4.80 (1H, s, 2-***H***), 3.60 – 3.53 (4H, m, 2‴-($\boldsymbol{H}_2$)$_2$), 3.43 – 3.33 (2H, m, 1″-$\boldsymbol{H}_2$), 2.36 (2H, t, *J* = 6.5 Hz, 3″-$\boldsymbol{H}_2$), 2.38 – 2.29 (4H, m, 3‴-($\boldsymbol{H}_2$)$_2$), 1.65 (2H, p, *J* = 6.5 Hz, 2″-$\boldsymbol{H}_2$); δ_C (176 MHz, $CDCl_3$) 171.3 (***C***=O), 139.4 (***C***-1^{Ph}), 138.3 (***C***-1′), 133.2 (***C***-4′), 130.3 (***C***-2′), 129.0 (***C***-3^{Ph}), 128.9 (***C***-3′), 128.8 (***C***-2^{Ph}), 127.6 (***C***-4^{Ph}), 67.1 (***C***-2‴), 58.7 (***C***-2), 57.6 (***C***-3″), 53.8 (***C***-3‴), 39.6 (***C***-1″), 25.0 (***C***-2″); *m/z* (LCMS, ESI^+) 373 ($M(^{35}Cl)H^+$), 375 ($M(^{37}Cl)H^+$); Accurate mass: Found MH^+, 373.1675: $C_{21}H_{26}N_2O_2{}^{35}Cl$ requires *M*, 373.1683.

N-(2″-[4‴-Methylpiperazin-1‴-yl]eth-1″-yl)-2-(4′-chlorophenyl)-2-phenylacetamide **S16**

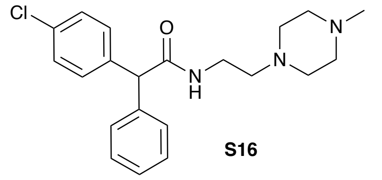

S16

Obtained in 39% yield as a pale yellow oil. ν_{max} (ATR) 3308 (br), 2934 (w), 2800 (w), 1651 (s), 1544 (w), 1491 (s), 1456 (w), 1288 (w), 1176 (w), 1096 (w), 1015 (m) cm^{-1}; δ_H (700 MHz, $CDCl_3$) 7.36 – 7.32 (2H, m, 3^{Ph}-$\boldsymbol{H}_2$), 7.31 – 7.26 (3H, m, 3′-$\boldsymbol{H}_2$, 4^{Ph}-***H***), 7.25 – 7.23 (2H, m, 2^{Ph}-$\boldsymbol{H}_2$), 7.22 – 7.20 (2H, m, 2′-$\boldsymbol{H}_2$), 6.28 – 6.18 (1H, m, N***H***), 4.90 (1H, s, 2-***H***), 3.40 – 3.31 (2H, m, 1″-$\boldsymbol{H}_2$), 2.55 – 2.11 (8H, m, 2‴-($\boldsymbol{H}_2$)$_2$, 3‴-($\boldsymbol{H}_2$)$_2$), 2.44 (2H, t, *J* = 6.0 Hz, 2″-$\boldsymbol{H}_2$), 2.27 (3H, s, NC$\boldsymbol{H}_3$); δ_C (176 MHz, $CDCl_3$) 171.5 (***C***=O), 139.4 (***C***-1^{Ph}), 138.3 (***C***-1′), 133.2 (***C***-4′), 130.5 (***C***-2′), 129.0(3) (Ar***C***), 128.9(90) (Ar***C***), 128.9(85) (Ar***C***), 127.6 (***C***-4^{Ph}), 58.8 (***C***-2), 56.0 (***C***-2″), 55.1 (***C***-3‴), 52.6 (***C***-2‴), 46.1 (N***C***H_3), 36.3 (***C***-1″); *m/z* (LCMS, ESI^+) 372 ($M(^{35}Cl)H^+$), 374 ($M(^{37}Cl)H^+$); Accurate mass: Found MH^+, 372.1836: $C_{21}H_{27}N_3O^{35}Cl$ requires *M*, 372.1843.

N-(3′′-[2′′′-Pyrrolidinon-1′′′-yl]prop-1′′-yl)-2-(4′-chlorophenyl)-2-phenylacetamide **S17**

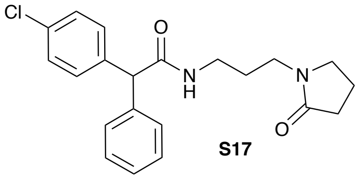

S17

Obtained in 51% yield as a colourless oil. ν_{max} (ATR) 3282 (br), 3052 (w), 2963 (w), 2934 (w), 2880 (w), 1655 (br, s), 1545 (w), 1490 (m), 1428 (w), 1291 (w), 1222 (w), 1089 (w) cm^{-1}; δ_H (400 MHz, $CDCl_3$) 7.33 – 7.21 (9H, m, Ar***H***), 7.08 – 7.00 (1H, m, N***H***), 4.82 (1H, s, 2-***H***), 3.33 (2H, apparent ddd, *J* = 8.5, 7.0, 1.5 Hz, 5′′′-***H***$_2$), 3.23 – 3.16 (4H, m, 1′′-***H***$_2$, 3′′-***H***$_2$), 2.34 (2H, t, *J* = 8.0 Hz, 3′′′-***H***$_2$), 1.98 (2H, apparent pd, *J* = 7.5, 2.0 Hz, 4′′′-***H***$_2$), 1.64 (2H, p, *J* = 6.0 Hz, 2′′-***H***$_2$); δ_C (101 MHz, $CDCl_3$) 176.1 (***C***-2′′′), 171.6 (***C***=O), 139.4 (***C***-1^{Ph}), 138.3 (***C***-1′), 133.0 (***C***-4′), 130.3 (Ar***C***), 128.8 (Ar***C***), 128.7 (Ar***C***), 127.3 (Ar***C***), 58.3 (***C***-2), 47.5 (***C***-5′′′), 39.6 (Al***C***), 36.1 (Al***C***), 31.0 (***C***-3′′′), 26.5 (***C***-2′′), 18.0 (***C***-4′′′); *m/z* (LCMS, ESI$^+$) 371 (M(^{35}Cl)H$^+$), 373 (M(^{37}Cl)H$^+$); Accurate mass: Found MH$^+$, 371.1516: $C_{21}H_{24}N_2O_2{}^{35}Cl$ requires *M*, 371.1526.

N-(1′′,2′′-Diphenylethyl)-2-(pyrrolidin-2′-yl)acetamide **S18**

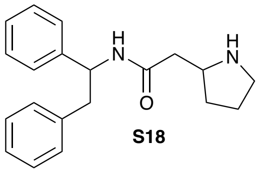

S18

Obtained in 52% yield as a yellow oil. ν_{max} (ATR) 3270 (br), 3094 (w), 3022 (w), 2918 (w), 1670 (s), 1544 (m), 1496 (w), 1199 (s), 1177 (s), 1129 (s), 1028 (w) cm^{-1}; δ_H (700 MHz, $CDCl_3$, mixture of diastereomers) 8.20 (0.5H, d, *J* = 8.0 Hz, 1-N***H*** isomer A), 7.88 (0.5H, d, *J* = 8.0 Hz, 1-N***H*** isomer B), 7.29 – 7.15 (8H, m, Ar***H***), 7.13 (1H, d, *J* = 7.5 Hz, 2^{Ph}-***H***$_2$ isomer A), 7.09 (1H, d, *J* = 7.0 Hz, 2^{Ph}-***H***$_2$ isomer B), 5.19 (0.5H, q, *J* = 8.0 Hz, 1′′-***H*** isomer A), 5.10 (0.5H, td, *J* = 8.0, 6.5 Hz, 1′′-***H*** isomer B), 3.61 – 3.53 (1H, m, 2′-***H***), 3.10 – 3.03 (1H, m, 5′-***H***H′), 3.05 (1H, d, *J* = 8.0 Hz, 2′′-***H***$_2$ isomer A), 3.02 (0.5H, d, *J* = 6.5 Hz, 2′′-***H***H′ isomer B), 3.02 (0.5H, d, *J* = 8.0 Hz, 2′′-H***H′*** isomer B), 3.00 – 2.95 (1H, m, 5′-H***H′***), 2.77 (0.5H, dd, *J* = 15.5, 7.5 Hz, 2-***H***H′ isomer B), 2.68 (0.5H, dd, *J* = 15.0, 7.0 Hz, 2-***H***H′ isomer A), 2.51 (0.5H, dd, *J* = 15.0, 5.0 Hz, 2-H***H′*** isomer A), 2.49 (0.5H, dd, *J* = 15.5, 5.0 Hz, 2-H***H′*** isomer B), 1.94 – 1.83 (2H, m, 4′-***H***H′ isomer A, 4′-***H***H′ isomer B, 3′-***H***H′ isomer A, 3′-***H***H′ isomer B), 1.81 – 1.72 (1H, m, 4′-H***H′*** isomer A, 4′-H***H′*** isomer B), 1.49 (0.5H, dq, *J* = 12.5, 9.0 Hz, 3′-H***H′*** isomer B), 1.40 (0.5H, dq, *J* = 13.5, 9.5, 8.5 Hz, 3′-H***H′*** isomer A); δ_C (176 MHz, $CDCl_3$, mixture of diastereomers) 169.6(1) (***C***=O isomer A), 169.5(8) (***C***=O isomer B), 141.9 (Ar***C***), 141.7 (Ar***C***), 138.1 (Ar***C***), 137.8 (Ar***C***), 129.5 (***C***-2^{Ph} isomer A), 129.4 (***C***-2^{Ph} isomer B), 128.7(0) (Ar***C***), 128.6(7) (Ar***C***), 128.5 (Ar***C***), 128.4 (Ar***C***), 127.6 (Ar***C***), 127.5 (Ar***C***), 126.8 (Ar***C***), 126.7 (Ar***C***), 126.5(9) (Ar***C***), 126.5(8) (Ar***C***), 57.5 (***C***-2′ isomer A), 57.0 (***C***-2′ isomer B), 55.4 (***C***-1′′ isomer B), 55.0 (***C***-1′′ isomer A), 45.0(0) (***C***-5′), 44.9(6) (***C***-5′), 43.0 (***C***-2′′ isomer B), 42.7 (***C***-2′′ isomer A), 36.9 (***C***-2 isomer B), 36.5 (***C***-2 isomer A), 29.9 (***C***-3′ isomer B),

29.4 (***C***-3′ isomer A), 23.7(3) (***C***-4′), 23.7(0) (***C***-4′); *m/z* (LCMS, ESI^+) 309 (MH^+); Accurate mass: Found MH^+, 309.1961: $C_{20}H_{25}N_2O$ requires *M*, 309.1967.

N-([2‴-Methylphenyl]phenylmethyl)-2-(pyrrolidin-2′-yl)acetamide **S19**

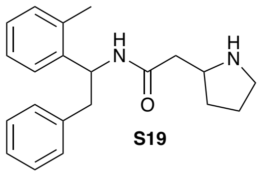

S19

Obtained in 73% yield as a white solid. m.p. 178 – 181 °C; ν_{max} (ATR) 3254 (w), 3060 (w), 3022 (w), 1674 (s), 1632 (s), 1558 (s), 1200 (s), 1188 (m), 1142 (w) cm^{-1}; δ_H (700 MHz, $CDCl_3$, mixture of diastereomers) 7.31 – 7.27 (2H, m, Ar***H***) 7.26 – 7.22 (2H, m, Ar***H***), 7.21 – 7.10 (5H, m, Ar***H***), 6.30 (0.5H, d, *J* = 7.5 Hz, 1″-***H***), 6.26 (0.5H, d, *J* = 7.5 Hz, 1″-***H***), 3.80 – 3.74 (1H, m, 2′-***H***), 3.17 – 3.07 (2H, m, 5′-$\boldsymbol{H}_2$), 2.91 (0.5H, dd, *J* = 16.5, 7.5 Hz, 2-***H***H′ isomer A), 2.88 (0.5H, dd, *J* = 16.5, 7.5 Hz, 2-***H***H′ isomer B), 2.76 (0.5H, dd, *J* = 16.5, 5.0 Hz, 2-H***H′*** isomer B), 2.73 (0.5H, dd, *J* = 16.5, 4.5 Hz, 2-H***H′*** isomer A), 2.27 (1.5H, s, 2‴-C$\boldsymbol{H}_3$), 2.22 (1.5H, s, 2‴-C$\boldsymbol{H}_3$), 2.10 – 2.04 (1H, m, 3′-***H***H′), 2.00 – 1.91 (1H, m, 4′-***H***H′), 1.91 – 1.82 (1H, m, 4′-H***H′***), 1.71 – 1.61 (1H, m, 3′-H***H′***); δ_C (176 MHz, $CDCl_3$, mixture of diastereomers) 169.6(0) (***C***-1), 169.5(8) (***C***-1), 140.5 (***C***-1^{Ph}), 140.3 (***C***-1^{Ph}), 139.2 (Ar***C***), 139.0 (Ar***C***), 136.1 (Ar***C***), 136.0 (Ar***C***), 130.9(2) (***C***-3‴), 130.9(0) (***C***-3‴), 128.9(3) (Ar***C***), 128.8(6) (Ar***C***), 127.8(4) (Ar***C***), 127.7(9) (Ar***C***), 127.7(5) (Ar***C***), 127.7 (Ar***C***), 127.5 (Ar***C***), 127.0 (Ar***C***), 126.6 (Ar***C***), 126.5(3) (Ar***C***), 126.4(9) (Ar***C***), 56.9(0) (***C***-2′), 56.8(5) (***C***-2′), 54.8 (***C***-1″), 54.5 (***C***-1″), 45.0(0) (***C***-5′), 44.9(8) (***C***-5′), 36.4 (***C***-2 isomer A), 36.2 (***C***-2 isomer B), 30.0 (***C***-3′), 29.9 (***C***-3′), 23.9(2) (***C***-4′), 23.9(1) (***C***-4′), 19.6 (2‴-***C***H_3), 19.5 (2‴-***C***H_3); *m/z* (LCMS, ESI^+) 309 (MH^+); Accurate mass: Found MH^+, 309.1978: $C_{20}H_{25}N_2O$ requires *M*, 309.1967.

N-([3‴-{Methanesulfonyl}phenyl]phenylmethyl)-2-(pyrrolidin-2′-yl)acetamide **S20**

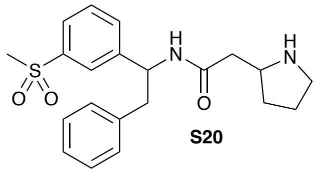

S20

Obtained in 53% yield as a yellow oil. ν_{max} (ATR) 3300 (br), 3038 (w), 2964 (w), 2866 (w), 1658 (m), 1532 (m), 1418 (w), 1302 (s), 1146 (s), 1092 (w) cm^{-1}; δ_H (700 MHz, $CDCl_3$, mixture of diastereomers) 9.14 (0.5H, d, *J* = 8.0 Hz, 1-N***H*** isomer B), 9.10 (0.5H, d, *J* = 8.0 Hz, 1-N***H*** isomer A), 7.92 (0.5H, t, *J* = 2.0 Hz, 2‴-***H***), 7.87 (0.5H, t, *J* = 2.0 Hz, 2‴-***H***), 7.82 – 7.78 (1H, m, 4‴-***H***), 7.54 – 7.51 (1H, m, 6‴-***H***), 7.51 – 7.47 (1H, m, 5‴-***H***), 7.34 – 7.29 (2H, m, 3^{Ph}-$\boldsymbol{H}_2$), 7.28 – 7.25 (1H, m, 4^{Ph}-***H***), 7.22 – 7.18 (2H, m, 2^{Ph}-$\boldsymbol{H}_2$), 6.30 (1H, d, *J* = 8.0 Hz, 1″-***H***), 3.45 (0.5H, ddt, *J* = 8.0, 7.5, 3.5 Hz, 2′-***H*** isomer A), 3.40 (0.5H, ddt, *J* = 8.5, 7.5, 3.5 Hz, 2′-***H*** isomer B), 3.02 (1.5H, s, $SO_2C\boldsymbol{H}_3$), 3.01 (1.5H, s, $SO_2C\boldsymbol{H}_3$), 2.92 (0.5H, ddd, *J* = 11.0, 7.5, 6.0 Hz, 5′-***H***H′ isomer B),

2.89 (0.5H, ddd, *J* = 11.0, 8.0, 6.0 Hz, 5′-H***H′*** isomer B), 2.87 (0.5H, ddd, *J* = 11.0, 8.0, 6.5 Hz, 5′-***H***H′ isomer A), 2.79 (0.5H, ddd, *J* = 11.0, 7.5, 6.5 Hz, 5′-H***H′*** isomer A), 2.47 (0.5H, dd, *J* = 15.5, 3.5 Hz, 2-***H***H′ isomer A), 2.46 (0.5H, dd, *J* = 15.5, 3.5 Hz, 2-***H***H′ isomer B), 2.32 (0.5H, dd, *J* = 15.5, 8.5 Hz, 2-H***H′*** isomer B), 2.30 (0.5H, dd, *J* = 15.5, 8.0 Hz, 2-H***H′*** isomer A), 1.92 – 1.86 (1H, m, 3′-***H***H′ isomer A, 3′-***H***H′ isomer B), 1.80 – 1.73 (0.5H, m, 4′-***H***H′ isomer B), 1.72 – 1.67 (0.5H, m, 4′-H***H′*** isomer B), 1.67 – 1.60 (1H, m, 4′-$\boldsymbol{H}_2$ isomer A), 1.42 – 1.34 (0.5H, m, 3′-H***H′*** isomer B), 1.38 – 1.30 (0.5H, m, 3′-H***H′*** isomer A); δ_C (176 MHz, $CDCl_3$, mixture of diastereomers) 171.7 (***C***-1 isomer A), 171.6 (***C***-1 isomer B), 144.5 (***C***-1‴), 144.4 (***C***-1‴), 141.2 (***C***-1^{Ph} isomer A), 141.1 (***C***-1^{Ph} isomer B), 141.0 (***C***-3‴), 140.9 (***C***-3‴), 132.9(1) (***C***-6‴), 132.8(6) (***C***-6‴), 129.7 (***C***-5‴), 129.6 (***C***-5‴), 129.1 (***C***-3^{Ph}), 129.0 (***C***-3^{Ph}), 128.0 (***C***-4^{Ph}), 127.9 (***C***-4^{Ph}), 127.7 (***C***-2^{Ph}), 127.5 (***C***-2^{Ph}), 126.2 (***C***-4‴), 126.1 (***C***-4‴), 125.7 (***C***-2‴), 125.5 (***C***-2‴), 56.4 (***C***-1″), 55.7(2) (***C***-2′), 55.6(9) (***C***-2′), 46.2 (***C***-5′ isomer B), 46.1 (***C***-5′ isomer A), 44.5(7) ($SO_2\boldsymbol{C}H_3$), 44.5(6) ($SO_2\boldsymbol{C}H_3$), 40.8 (***C***-2 isomer A), 40.7 (***C***-2 isomer B), 31.4 (***C***-3′ isomer B), 31.2 (***C***-3′ isomer A), 25.3(9) (***C***-4′ isomer A), 25.3(6) (***C***-4′ isomer B); *m/z* (LCMS, ESI^+) 373 (MH^+); Accurate mass: Found MH^+, 373.1592: $C_{20}H_{25}N_2O_3S$ requires *M*, 373.1586.

N-([Pyrrolidin-2″-yl]methyl)-2-phenylpropionamide **S21**

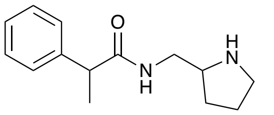

S21

Obtained in 28% yield as a yellow oil. ν_{max} (ATR) 3282 (br), 3058 (w), 3020 (w), 2964 (w), 2876 (w), 1647 (s), 1542 (br, m), 1456 (w), 1408 (w), 1360 (w), 1234 (w), 1184 (w), 1118 (w), 1070 (w) cm^{-1}; δ_H (600 MHz, $CDCl_3$, mixture of diastereomers) 7.35 – 7.28 (4H, m, 2^{Ph}-$\boldsymbol{H}_2$, 3^{Ph}-$\boldsymbol{H}_2$), 7.26 – 7.22 (1H, m, 4^{Ph}-***H***), 6.01 – 5.87 (1H, m, N***H***), 3.55 (0.5H, q, *J* = 7.0 Hz, 2-***H*** isomer A), 3.54 (0.5H, q, *J* = 7.0 Hz, 2-***H*** isomer B), 3.31 (0.5H, ddd, *J* = 13.5, 6.5, 4.5 Hz, NHC***H***H′ isomer A), 3.30 (0.5H, ddd, *J* = 13.0, 6.5, 4.5 Hz, NHC***H***H′ isomer B), 3.20 (0.5H, qd, *J* = 7.5, 4.5 Hz, 2″-***H*** isomer B), 3.15 (0.5H, tdd, *J* = 7.5, 7.0, 4.5 Hz, 2″-***H*** isomer A), 3.05 – 2.97 (1H, m, NHCH***H′*** isomer A, NHCH***H′*** isomer B), 2.85 – 2.76 (1.5H, m, 5″-$\boldsymbol{H}_2$ isomer A, 5″-***H***H′ isomer B), 2.74 (0.5H, dt, *J* = 10.5, 7.0 Hz, 5″-H***H′*** isomer B), 1.81 – 1.72 (1H, m, 3″-***H***H′ isomer A, 3″-***H***H′ isomer B), 1.72 – 1.63 (0.5H, m, 4″-***H***H′ isomer A), 1.64 – 1.57 (1.5H, m, 4″-H***H′*** isomer A, 4″-$\boldsymbol{H}_2$ isomer B), 1.51 (3H, d, *J* = 7.0 Hz, 3-$\boldsymbol{H}_3$), 1.28 (0.5H, ddt, *J* = 13.5, 8.5, 7.0 Hz, 3″-H***H′*** isomer A), 1.27 – 1.18 (0.5H, m, 3″-H***H′*** isomer B); δ_C (151 MHz, $CDCl_3$, mixture of diastereomers) 174.5 (***C***=O isomer A), 174.4 (***C***=O isomer B), 141.7(7) (***C***-1^{Ph} isomer B), 141.7(5) (***C***-1^{Ph} isomer A), 128.9 (***C***-3^{Ph}), 127.7(0) (***C***-2^{Ph}), 127.6(9) (***C***-2^{Ph}), 127.3 (***C***-4^{Ph}), 57.6(7) (***C***-2″ isomer A), 57.6(5) (***C***-2″ isomer B), 47.3(3) (***C***-2), 47.3(1) (***C***-2), 46.7 (***C***-5″ isomer B), 46.6 (***C***-5″ isomer A), 44.0 (NH***C***HH′ isomer A), 43.9 (NH***C***HH′ isomer B), 29.1 (***C***-3″ isomer A), 29.0 (***C***-3″ isomer B), 26.0 (***C***-4″), 18.7 (***C***-3), 18.6 (***C***-3); *m/z* (LCMS, ESI^+) 233 (MH^+); Accurate mass: Found MH^+, 233.1654: $C_{14}H_{21}N_2O$ requires *M*, 233.1654.

N-([Pyrrolidin-2′-yl]methyl)-2-phenylacetamide **S22**

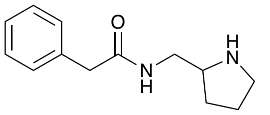

S22

Obtained in 16% yield as a colourless oil. ν_{max} (ATR) 3014 (br), 2892 (w), 1674 (s), 1558 (w), 1444 (w), 1364 (s), 1201 (s), 1128 (w) cm^{-1}; δ_H (600 MHz, $CDCl_3$) 7.82 – 7.77 (1H, m, N***H***), 7.31 (2H, t, *J* = 7.5 Hz, 3^{Ph}-***H***$_2$), 7.26 – 7.25 (1H, m, 4^{Ph}-***H***), 7.23 (2H, d, *J* = 7.5 Hz, 2^{Ph}-***H***$_2$), 3.77 – 3.71 (1H, m, 2′-***H***), 3.56 (1H, apparent dd, *J* = 15.0, 7.5 Hz, NHC***H***H′), 3.53 (2H, s, 2-***H***$_2$), 3.37 (1H, dt, *J* = 15.0, 3.5 Hz, NHCH***H′***), 3.15 (1H, ddd, *J* = 11.5, 8.0, 6.0 Hz, 5′-***H***H′), 3.07 (1H, dt, *J* = 11.5, 7.5 Hz, 5′-H***H′***), 2.09 – 2.00 (1H, m, 3′-***H***H′), 1.99 – 1.87 (2H, m, 4′-***H***$_2$), 1.62 (1H, dq, *J* = 13.0, 8.5 Hz, 3′-H***H′***); δ_C (151 MHz, $CDCl_3$) 173.8 (***C***=O), 134.8 (***C***-1^{Ph}), 129.5 (***C***-2^{Ph}), 129.0 (***C***-3^{Ph}), 127.5 (***C***-4^{Ph}), 60.3 (***C***-2′), 45.2 (***C***-5′), 43.2 (***C***-2), 41.0 (NH***C***HH′), 27.8 (***C***-3′), 24.1 (***C***-4′); *m/z* (LCMS, ESI$^+$) 219 (MH$^+$); Accurate mass: Found MH$^+$, 219.1497: $C_{13}H_{19}N_2O$ requires *M*, 219.1497.

N-(1′′-Phenylhexyl)-2-(pyrrolidin-2′-yl)acetamide **S23**

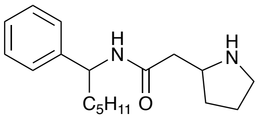

S23

Obtained in 31% yield as a yellow oil. ν_{max} (ATR) 3290 (br), 3076 (w), 2964 (w), 2930 (w), 2858 (w), 1650 (s), 1558 (m), 1456 (w), 1418 (w), 1199 (s), 1175 (s), 1130 (s) cm^{-1}; δ_H (700 MHz, $CDCl_3$, mixture of diastereomers) 7.74 (0.5H, d, *J* = 7.5 Hz, 1-N***H*** isomer A), 7.68 (0.5H, d, *J* = 7.5 Hz, 1-N***H*** isomer B), 7.31 – 7.27 (2H, m, 3^{Ph}-***H***$_2$), 7.26 – 7.23 (2H, m, 2^{Ph}-***H***$_2$), 7.23 – 7.19 (1H, m, 4^{Ph}-***H***), 4.78 (0.5H, q, *J* = 7.5 Hz, 1′′-***H*** isomer B), 4.76 (0.5H, q, *J* = 7.5 Hz, 1′′-***H*** isomer A), 3.79 – 3.72 (1H, m, 2′-***H***), 3.21 – 3.15 (1H, m, 5′-***H***H′), 3.13 – 3.07 (1H, m, 5′-H***H′***), 2.86 (0.5H, dd, *J* = 16.0, 7.5 Hz, 2-***H***H′ isomer B), 2.81 (0.5H, dd, *J* = 15.5, 7.0 Hz, 2-***H***H′ isomer A), 2.65 (0.5H, dd, *J* = 16.0, 5.0 Hz, 2-H***H′*** isomer B), 2.62 (0.5H, dd, *J* = 15.5, 5.0 Hz, 2-H***H′*** isomer A), 2.11 – 2.04 (1H, m, 3′-***H***H′ isomer A, 3′-***H***H′ isomer B), 2.02 – 1.94 (1H, m, 4′-***H***H′), 1.92 – 1.83 (1H, m, 4′-H***H′***), 1.80 – 1.60 (3H, m, 2′′-***H***$_2$, 3′-H***H′*** isomer A, 3′-H***H′*** isomer B), 1.32 – 1.13 (6H, m, 3′′-***H***$_2$, 4′′-***H***$_2$, 5′′-***H***$_2$), 0.88 – 0.80 (3H, m, 6′′-***H***$_3$); δ_C (176 MHz, $CDCl_3$, mixture of diastereomers) 169.8 (***C***=O isomer B), 169.7 (***C***=O isomer A), 142.7 (***C***-1^{Ph} isomer B), 142.5 (***C***-1^{Ph} isomer A), 128.7 (***C***-3^{Ph}), 127.4 (***C***-4^{Ph}), 127.3 (***C***-4^{Ph}), 126.7 (***C***-2^{Ph} isomer A), 126.5 (***C***-2^{Ph} isomer B), 57.0(9) (***C***-2′ isomer A), 57.0(5) (***C***-2′ isomer B), 54.4(4) (***C***-1′′ isomer A), 54.3(6) (***C***-1′′ isomer B), 45.0 (***C***-5′ isomer B), 44.9 (***C***-5′ isomer A), 36.9 (***C***-2 isomer B), 36.6(4) (***C***-2′′ isomer B), 36.6(2) (***C***-2 isomer A), 36.3 (***C***-2′′ isomer A), 31.6(4) (***C***-4′′), 31.6(1) (***C***-4′′), 30.1 (***C***-3′ isomer B), 29.8 (***C***-3′ isomer A), 26.0(9) (***C***-3′′ isomer B), 26.0(6) (***C***-3′′ isomer A), 24.0 (***C***-4′ isomer A), 23.9 (***C***-4′ isomer B), 22.6(1) (***C***-5′′), 22.5(9) (***C***-5′′), 14.1(3) (***C***-6′′), 14.1(2) (***C***-6′′); *m/z* (LCMS, ESI$^+$) 289 (MH$^+$); Accurate mass: Found MH$^+$, 289.2284: $C_{18}H_{29}N_2O$ requires *M*, 289.2280.

N-([Pyrrolidin-2′′-yl]methyl)-2-(phenyl)-2-cyclopentylacetamide **S24**

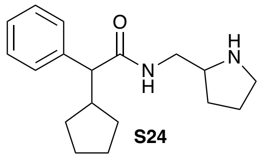
S24

Obtained in 11% yield as a pale yellow oil. ν_{max} (ATR) 3296 (br), 2950 (m), 2868 (w), 1645 (s), 1542 (m), 1446 (w), 1374 (w), 1222 (w) cm^{-1}; δ_H (600 MHz, CDCl$_3$, mixture of diastereomers) 7.38 – 7.31 (2H, m, 2^{Ph}-***H***$_2$), 7.28 (2H, td, *J* = 7.5, 2.5 Hz, 3^{Ph}-***H***$_2$), 7.24 – 7.20 (1H, m, 4^{Ph}-***H***), 6.21 – 6.11 (1H, m, N***H***), 3.37 (0.5H, ddd, *J* = 13.5, 6.0, 4.5 Hz, NHC***H***H′ isomer A), 3.29 (0.5H, ddd, *J* = 13.0, 6.0, 4.0 Hz, NHC***H***H′ isomer B), 3.22 (0.5H, qd, *J* = 7.5, 4.5 Hz, 2′′-***H*** isomer A), 3.17 (0.5H, ddt, *J* = 7.5, 7.0, 4.0 Hz, 2′′-***H*** isomer B), 3.06 (0.5H, ddd, *J* = 13.0, 7.5, 5.5 Hz, NHCH***H′*** isomer B), 2.99 (1H, d, *J* = 11.0 Hz, 2-***H***), 2.96 (0.5H, ddd, *J* = 13.5, 7.5, 5.0 Hz, NHCH***H′*** isomer A), 2.88 – 2.81 (1.5H, m, 5′′-***H***H′ isomer A, 5′′-***H***$_2$ isomer B), 2.78 (0.5H, dt, *J* = 10.5, 6.5 Hz, 5′′-H***H′*** isomer A), 2.67 – 2.56 (1H, m, 1^{Cyp}-***H***), 1.98 – 1.91 (1H, m, 2^{Cyp}-(***H***H′)(HH′)), 1.82 – 1.73 (1H, m, 3′′-***H***H′ isomer A, 3′′-***H***H′ isomer B), 1.73 – 1.68 (0.5H, m, 4′′-***H***H′ isomer B), 1.68 – 1.51 (4.5H, m, 3^{Cyp}-(***H***H′)H$_2$, 3^{Cyp}-(HH′)***H***$_2$, 4′′-***H***$_2$ isomer A, 4′′-H***H′*** isomer B), 1.51 – 1.45 (1H, m, 3^{Cyp}-(H***H′***)H$_2$), 1.45 – 1.38 (1H, m, 2^{Cyp}-(HH′)(***H***H′)), 1.31 (0.5H, ddt, *J* = 12.5, 8.5, 7.0 Hz, 3′′-H***H′*** isomer B), 1.28 – 1.19 (1.5H, m, 2^{Cyp}-(H***H′***)(HH′), 3′′-H***H′*** isomer A), 0.98 (1H, dq, *J* = 12.5, 8.5 Hz, 2^{Cyp}-(HH′)(H***H′***)); δ_C (151 MHz, CDCl$_3$, mixture of diastereomers) 173.8 (***C***=O isomer B), 173.7 (***C***=O isomer A), 140.5(3) (***C***-1^{Ph}), 140.5(2) (***C***-1^{Ph}), 128.6 (***C***-3^{Ph}), 128.1 (***C***-2^{Ph}), 127.1 (***C***-4^{Ph}), 60.2 (***C***-2), 57.9 (***C***-2′′ isomer A), 57.7 (***C***-2′′ isomer B), 46.6(7) (***C***-5′′), 46.6(5) (***C***-5′′), 43.7 (NH***C***HH′), 43.4 (***C***-1^{Cyp}), 43.3 (***C***-1^{Cyp}), 31.8(6) (***C***-2^{Cyp}), 31.8(5) (***C***-2^{Cyp}), 31.1 (***C***-2^{Cyp}), 29.0(2) (***C***-3′′ isomer A), 28.9(9) (***C***-3′′ isomer B), 26.0(2) (***C***-4′′ isomer A), 25.9(9) (***C***-4′′ isomer B), 25.3(4) (***C***-3^{Cyp}), 25.3(3) (***C***-3^{Cyp}), 25.0(4) (***C***-3^{Cyp}), 25.0(3) (***C***-3^{Cyp}); *m/z* (LCMS, ESI$^+$) 287 (MH$^+$); Accurate mass: Found MH$^+$, 287.2120: $C_{18}H_{27}N_2O$ requires *M*, 287.2123.

N-([Pyrrolidin-2′-yl]methyl)-1-phenyl-cyclohexane-1-carboxamide **S25**

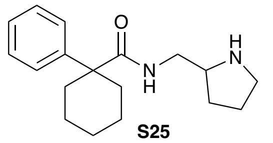
S25

Obtained in 58% yield as an orange oil. ν_{max} (ATR) 3340 (br), 2942 (m), 2856 (w), 2782 (w), 1671 (s), 1594 (s), 1524 (s), 1446 (m), 1199 (s), 1131 (s) cm^{-1}; δ_H (600 MHz, CDCl$_3$) 7.41 (2H, d, *J* = 7.5 Hz, 2^{Ph}-***H***$_2$), 7.34 – 7.28 (1H, m, N***H***), 7.30 (2H, t, *J* = 7.5 Hz, 3^{Ph}-***H***$_2$), 7.20 (1H, t, *J* = 7.5 Hz, 4^{Ph}-***H***), 3.69 – 3.61 (2H, m, NHC***H***H′, 2′-***H***), 3.29 – 3.23 (1H, m, NHCH***H′***), 2.99 (1H, ddd, *J* = 11.5, 7.5, 4.5 Hz, 5′-***H***H′), 2.60 (1H, ddd, *J* = 11.5, 9.0, 7.0 Hz, 5′-H***H′***), 2.40 – 2.29 (2H, m, 2-(***H***H′)(HH′), 2-(HH′)(***H***H′)), 2.00 – 1.93 (1H, m, 2-(H***H′***)(HH′)), 1.93 – 1.82 (2H, m, 3′-***H***H′, 2-(HH′)(H***H′***)), 1.79 – 1.68 (1H, m, 4′-***H***H′), 1.68 – 1.58 (2H, m, 4′-H***H′***, 3-(***H***H′)H$_2$), 1.58 – 1.47 (4H, m, 3-(H***H′***)H$_2$, 4-***H***H′, 3-(HH′)***H***$_2$), 1.43 (1H, ddd, *J* = 16.5, 13.5, 8.0 Hz, 3′-H***H′***), 1.38 – 1.30 (1H,

m, 4-H***H'***); δ_C (151 MHz, $CDCl_3$) 177.5 (***C***=O), 144.1 (***C***-1^{Ph}) 128.7 (***C***-3^{Ph}), 126.8 (***C***-4^{Ph}), 126.3 (***C***-2^{Ph}), 59.9 (***C***-2′), 50.6 (***C***-1), 45.2 (***C***-5′), 39.9 (NH***C***HH′), 34.6 (***C***-2), 33.8 (***C***-2), 27.1 (***C***-3′), 25.8 (***C***-4), 24.6 (***C***-4′), 23.3 (***C***-3); *m/z* (LCMS, ESI^+) 287 (MH^+); Accurate mass: Found MH^+, 287.2133: $C_{18}H_{27}N_2O$ requires *M*, 287.2123.

N-([Tetrahydrofuran-2′′′-yl]phenylmethyl)-2-(pyrrolidin-2′-yl)acetamide **S26**

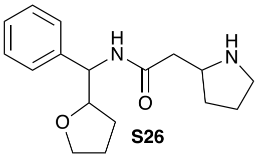
S26

Obtained in 29% yield as a pale yellow oil. ν_{max} (ATR) 3282 (br), 2968 (w), 2876 (w), 1641 (s), 1536 (s), 1070 (m) cm^{-1}; δ_H (600 MHz, $CDCl_3$, mixture of diastereomers) 8.44 (0.3H, d, *J* = 8.5 Hz, 1-N***H***), 8.42 (0.3H, d, *J* = 8.5 Hz, 1-N***H***), 8.22 (0.2H, d, *J* = 8.5 Hz, 1-N***H***), 8.18 (0.2H, d, *J* = 8.5 Hz, 1-N***H***), 7.37 – 7.27 (4H, m, Ar***H***), 7.25 – 7.20 (1H, m, Ar***H***), 5.05 (0.2H, dd, *J* = 8.5, 4.0 Hz, 1′′-***H***), 5.04 (0.2H, dd, *J* = 8.5, 4.0 Hz, 1′′-***H***), 5.00 (0.3H, dd, *J* = 8.5, 5.0 Hz, 1′′-***H***), 4.99 (0.3H, dd, *J* = 8.5, 5.0 Hz, 1′′-***H***), 4.22 (0.3H, td, *J* = 7.0, 5.0 Hz, 2′′′-***H***), 4.20 (0.3H, td, *J* = 7.0, 5.0 Hz, 2′′′-***H***), 4.16 (0.2H, td, *J* = 6.5, 4.0 Hz, 2′′′-***H***), 4.14 (0.2H, td, *J* = 6.5, 2.0 Hz, 2′′′-***H***), 3.91 (0.2H, td, *J* = 7.0, 3.0 Hz, 5′′′-***H***H′), 3.90 (0.2H, td, *J* = 7.0, 3.0 Hz, 5′′′-H***H′***), 3.78 (0.2H, td, *J* = 8.0, 6.0 Hz, 5′′′-***H***H′), 3.77 (0.2H, td, *J* = 8.0, 6.0 Hz, 5′′′-H***H′***), 3.73 – 3.66 (1.2H, m, 5′′′-$\boldsymbol{H_2}$), 3.42 – 3.31 (1H, m, 2′-***H***), 3.00 (0.3H, ddd, *J* = 11.0, 8.0, 6.0 Hz, 5′-***H***H′), 2.99 (0.3H, ddd, *J* = 10.5, 8.0, 5.5 Hz, 5′-***H***H′), 2.96 – 2.86 (1.4H, m, 5′-H***H′***, 5′-$\boldsymbol{H_2}$), 2.46 (0.2H, dd, *J* = 15.0, 4.0 Hz, 2-***H***H′), 2.43 (0.3H, dd, *J* = 15.5, 4.0 Hz, 2-***H***H′), 2.42 (0.2H, dd, *J* = 15.0, 4.0 Hz, 2-H***H′***), 2.39 (0.3H, dd, *J* = 15.5, 4.0 Hz, 2-H***H′***), 2.31 (0.2H, dd, *J* = 15.5, 8.5 Hz, 2-***H***H′), 2.28 (0.2H, dd, *J* = 15.5, 8.5 Hz, 2-H***H′***), 2.27 (0.3H, dd, *J* = 15.0, 8.5 Hz, 2-***H***H′), 2.24 (0.3H, dd, *J* = 15.0, 8.0 Hz, 2-H***H′***), 1.97 – 1.78 (3.4H, m, 4′-$\boldsymbol{H_2}$, 2′′′-$\boldsymbol{H_2}$, 4′′′-$\boldsymbol{H_2}$, 3′′′-$\boldsymbol{H_2}$), 1.77 – 1.63 (2.4H, m, 4′-$\boldsymbol{H_2}$, 4′′′-$\boldsymbol{H_2}$, 3′′′-$\boldsymbol{H_2}$), 1.63 – 1.56 (0.6H, m, 3′′′-$\boldsymbol{H_2}$), 1.56 – 1.48 (0.6H, m, 4′′′-$\boldsymbol{H_2}$), 1.44 – 1.26 (1H, m, 3′-$\boldsymbol{H_2}$); δ_C (151 MHz, $CDCl_3$, mixture of diastereomers) 171.8 (***C***=O), 171.3 (***C***=O), 141.5(3) (***C***-1^{Ph}), 141.4(6) (***C***-1^{Ph}), 139.6(0) (***C***-1^{Ph}), 139.5(5) (***C***-1^{Ph}), 128.6 (Ar***C***), 128.4 (Ar***C***), 128.3(1) (Ar***C***), 128.2(6) (Ar***C***), 127.5 (Ar***C***), 127.4 (Ar***C***), 127.3(0) (Ar***C***), 127.2(6) (Ar***C***), 127.2 (Ar***C***), 127.1 (Ar***C***), 81.8 (***C***-2′′′), 81.7 (***C***-2′′′), 81.4(4) (***C***-2′′′), 81.4(2) (***C***-2′′′), 69.0 (***C***-5′′′), 68.9(0) (***C***-5′′′), 68.8(5) (***C***-5′′′), 68.8 (***C***-5′′′), 56.3 (***C***-1′′), 56.2 (***C***-1′′), 56.0(2) (***C***-2′), 55.9(5) (***C***-2′), 55.9 (***C***-2′), 55.8 (***C***-2′), 55.5(4) (***C***-1′′), 55.4(8) (***C***-1′′), 46.3(4) (***C***-5′), 46.3(0) (***C***-5′), 46.2 (***C***-5′), 41.6(3) (***C***-2), 41.5(9) (***C***-2), 41.5 (***C***-2), 41.3 (***C***-2), 31.5 (***C***-3′), 31.4 (***C***-3′), 31.3 (***C***-3′), 31.1 (***C***-3′), 29.2(4) (***C***-3′′′), 29.1(8) (***C***-3′′′), 28.4(3) (***C***-3′′′), 28.4(0) (***C***-3′′′), 26.0(3) (***C***-4′′′), 25.9(8) (***C***-4′′′), 25.7(8) (***C***-4′′′), 25.7(5) (***C***-4′′′), 25.4 (***C***-4′), 25.3 (***C***-4′), 25.2(3) (***C***-4′), 25.1(8) (***C***-4′); *m/z* (LCMS, ESI^+) 289 (MH^+); Accurate mass: Found MH^+, 289.1902: $C_{17}H_{25}N_2O_2$ requires *M*, 289.1916.

N-([Pyrrolidin-2′′-yl]methyl)-2-(2′,4′-di[trifluoromethyl]phenyl)acetamide **S27**

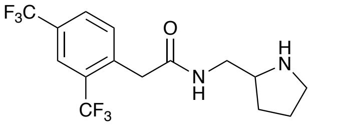

S27

Obtained in 49% yield an off-white solid. m.p. 90 – 93 °C; ν_{max} (ATR) 3282 (br), 3072 (w), 2964 (w), 2868 (w), 1655 (m), 1564 (w), 1524 (w), 1346 (s), 1276 (s), 1125 (s), 1056 (m) cm^{-1}; δ_H (700 MHz, $CDCl_3$) 7.91 (1H, s, 3′-***H***), 7.79 (1H, d, *J* = 8.0 Hz, 5′-***H***), 7.68 (1H, d, *J* = 8.0 Hz, 6′-***H***), 6.14 (1H, br m, N-***H***), 3.76 (2H, s, 2-***H***$_2$), 3.38 (1H, ddd, *J* = 13.5, 6.0, 4.5 Hz, NHC***H***H′), 3.26 (1H, dddd, *J* = 8.0, 7.5, 7.0, 4.5 Hz, 2′′-***H***), 3.02 (1H, ddd, *J* = 13.5, 7.5, 5.0 Hz, NHCH***H′***), 2.90 (1H, ddd, *J* = 10.5, 7.5, 6.0 Hz, 5′′-***H***H′), 2.83 (1H, dt, *J* = 10.5, 6.5 Hz, 5′′-H***H′***), 1.93 (1H, br m, 1′′-***H***), 1.83 (1H, dtd, *J* = 13.0, 8.0, 5.5 Hz, 3′′-***H***H′), 1.77 – 1.69 (1H, m, 4′′-***H***H′), 1.72 – 1.63 (1H, m, 4′′-H***H′***), 1.33 (1H, ddt, *J* = 13.0, 9.0, 7.0 Hz, 3′′-H***H′***); δ_C (176 MHz, $CDCl_3$) 168.7 (***C***=O), 137.9 (***C***-1′), 133.6 (***C***-6′), 130.0 (q, *J* = 33.5 Hz, ***C***-4′), 129.62 (q, *J* = 31.0 Hz, ***C***-2′), 128.9 (***C***-5′), 124.53 (q, *J* = 274.0 Hz, 2′-***C***F$_3$), 123.48 (q, *J* = 272.0 Hz, 4′-***C***F$_3$), 123.4 (***C***-3′), 57.4 (***C***-2′′), 46.7 (***C***-5′′), 44.1 (NH***C***HH′), 40.1 (***C***-2), 29.2 (***C***-3′′), 26.1 (***C***-4′′); δ_F (376 MHz, $CDCl_3$) −60.40 (Al***F***), −63.30 (Al***F***); *m/z* (LCMS, ESI$^+$) 355 (MH$^+$); Accurate mass: Found MH$^+$, 355.1249: $C_{15}H_{17}N_2OF_6$ requires *M*, 355.1245.

N-(3′′-[Pyrazol-1′′′-yl]prop-1′′-yl)-2-(4′-chlorophenyl)-2-phenylacetamide **S28**

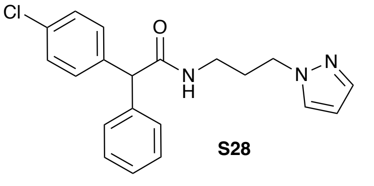

S28

Obtained in 44% yield as a a white solid. m.p. 61 – 63 °C; ν_{max} (ATR) 3300 (br), 3072 (w), 3018 (w), 2942 (w), 1646 (s), 1544 (m), 1489 (s), 1402 (m), 1368 (m), 1275 (m), 1212 (m), 1180 (w), 1092 (m), 1019 (s) cm^{-1}; δ_H (700 MHz, $CDCl_3$) 7.43 (1H, dd, *J* = 2.0, 0.5 Hz, 3′′′-***H***), 7.34 – 7.31 (3H, m, Ar***H***), 7.30 – 7.26 (3H, m, Ar***H***), 7.25 – 7.22 (2H, m, Ar***H***), 7.21 – 7.18 (2H, m, Ar***H***), 6.23 – 6.19 (1H, m, 4′′′-***H***), 6.16 (1H, t, *J* = 5.5 Hz, N***H***), 4.78 (1H, s, 2-***H***), 4.13 (2H, t, *J* = 6.5 Hz, 3′′-***H***$_2$), 3.32 – 3.22 (2H, m, 1′′-***H***$_2$), 2.02 (2H, p, *J* = 6.5 Hz, 2′′-***H***$_2$); δ_C (176 MHz, $CDCl_3$) 171.7 (***C***=O), 139.5 (***C***-3′′′), 139.2 (Ar***C***), 138.1 (Ar***C***), 133.3 (***C***-4′), 130.4 (Ar***C***), 129.5 (***C***-5′′′), 129.0 (Ar***C***), 128.9 (Ar***C***), 128.8 (Ar***C***), 127.6 (Ar***C***), 105.9 (***C***-4′′′), 58.6 (***C***-2), 49.7 (***C***-3′′), 37.4 (***C***-1′′), 30.2 (***C***-2′′); *m/z* (LCMS, ESI$^+$) 354 (M(^{35}Cl)H$^+$), 356 (M(^{37}Cl)H$^+$); Accurate mass: Found MH$^+$, 354.1378: $C_{20}H_{21}N_3O^{35}Cl$ requires *M*, 354.1373.

N-(2″-[N-Methyl-1‴,2‴,4‴-triazol-5‴-yl]eth-1″-yl)-2-(4′-chlorophenyl)-2-phenylacetamide **S29**

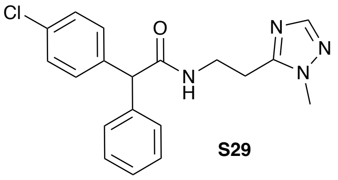

S29

Obtained in 31% yield as a a colourless oil. ν_{max} (ATR) 3308 (br), 3040 (w), 2958 (w), 1655 (s), 1524 (w), 1491 (s), 1368 (m), 1222 (m), 1088 (w), 1015 (w) cm^{-1}; δ_H (700 MHz, $CDCl_3$) 7.69 (1H, s, 3‴-***H***), 7.32 – 7.29 (2H, m, 3^{Ph}-***H***$_2$), 7.28 – 7.25 (3H, m, 3′-***H***$_2$, 4^{Ph}-***H***), 7.19 – 7.17 (2H, m, 2^{Ph}-***H***$_2$), 7.16 – 7.14 (2H, m, 2′-***H***$_2$), 6.67 – 6.63 (1H, m, N***H***), 4.80 (1H, s, 2-***H***), 3.75 (3H, s, NC***H***$_3$), 3.74 (2H, q, *J* = 6.0 Hz, 1″-***H***$_2$), 2.91 (2H, t, *J* = 6.0 Hz, 2″-***H***$_2$); δ_C (176 MHz, $CDCl_3$) 171.9 (***C***=O), 153.3 (***C***-5‴), 150.2 (***C***-3‴), 138.9 (***C***-1^{Ph}), 137.8 (***C***-1′), 133.3 (***C***-4′), 130.3 (***C***-2′), 129.0(1) (Ar***C***), 128.9(5) (Ar***C***), 128.8 (***C***-2^{Ph}), 127.6 (***C***-4^{Ph}), 58.6 (***C***-2), 36.9 (***C***-1″), 35.2 (N***C***H$_3$), 25.5 (***C***-2″); *m/z* (LCMS, ESI^+) 355 ($M(^{35}Cl)H^+$), 357 ($M(^{37}Cl)H^+$); Accurate mass: Found MH^+, 355.1329: $C_{19}H_{20}N_4O^{35}Cl$ requires *M*, 355.1326.

N-(2″-[3‴,5‴-Dimethylpyrazol-1‴-yl]eth-1″-yl)-2-(4′-chlorophenyl)-2-phenylacetamide **S30**

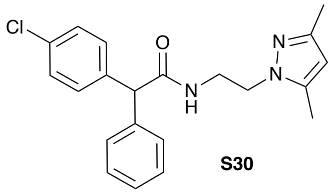

S30

Obtained in 49% yield as a white solid. m.p. 100 – 101 °C; ν_{max} (ATR) 3282 (br), 3086 (w), 3060 (w), 2922 (w), 1649 (s), 1551 (s), 1489 (s), 1453 (m), 1360 (w), 1222 (w), 1090 (m), 1015 (m) cm^{-1}; δ_H (700 MHz, $CDCl_3$) 7.32 – 7.29 (2H, m, 3^{Ph}-***H***$_2$), 7.28 – 7.24 (3H, m, 3′-***H***$_2$, 4^{Ph}-***H***), 7.20 – 7.18 (2H, m, 2^{Ph}-***H***$_2$), 7.17 – 7.14 (2H, m, 2′-***H***$_2$), 6.49 (1H, t, *J* = 6.0 Hz, N***H***), 5.75 (1H, s, 4‴-***H***), 4.82 (1H, s, 2-***H***), 4.03 (2H, dd, *J* = 6.5, 5.0 Hz, 2″-***H***$_2$), 3.69 (2H, ddd, *J* = 6.5, 6.0, 5.0 Hz, 1″-***H***$_2$), 2.12 (3H, s, 5‴-C***H***$_3$), 2.11 (3H, s, 3‴-C***H***$_3$); δ_C (176 MHz, $CDCl_3$) 171.9 (***C***=O), 148.1 (***C***-3‴), 139.6 (***C***-5‴), 138.9 (***C***-1^{Ph}), 137.9 (***C***-1′), 133.3 (***C***-4′), 130.4 (***C***-2′), 129.0 (Ar***C***), 128.9(4) (Ar***C***), 128.8(8) (Ar***C***), 127.6 (***C***-4^{Ph}), 105.3 (***C***-4‴), 58.7 (***C***-2), 46.9 (***C***-2″), 39.8 (***C***-1″), 13.6 (3‴-***C***H$_3$), 11.0 (5‴-***C***H$_3$); *m/z* (LCMS, ESI^+) 368 ($M(^{35}Cl)H^+$), 370 ($M(^{37}Cl)H^+$); Accurate mass: Found MH^+, 368.1525: $C_{21}H_{23}N_3O^{35}Cl$ requires *M*, 368.1530.

N-([Imidazo[1,2-a]pyridin-2′′-yl]methyl)-2-(4′-chlorophenyl)-2-phenylacetamide **S31**

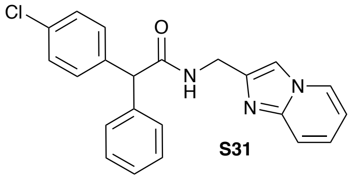

S31

Obtained in 13% yield as a yellow oil. ν_{max} (ATR) 3282 (br), 3062 (w), 3020 (w), 1668 (s), 1534 (w), 1490 (s), 1364 (w), 1208 (m), 1136 (w), 1018 (w) cm^{-1}; δ_H (600 MHz, $CDCl_3$) 8.10 (1H, d, *J* = 7.0 Hz, 5′′-***H***), 7.69 – 7.62 (1H, m, N***H***), 7.65 (1H, d, *J* = 9.0 Hz, 8′′-***H***), 7.54 (1H, s, 3′′-***H***), 7.39 – 7.36 (1H, m, 7′′-***H***), 7.25 – 7.23 (4H, m, 2^{Ph}-$\boldsymbol{H}_2$, Ar***H***), 7.23 – 7.19 (5H, m, 2′-$\boldsymbol{H}_2$, Ar***H***), 6.96 (1H, t, *J* = 7.0 Hz, 6′′-***H***), 4.89 (1H, s, 2-***H***), 4.60 (2H, t, *J* = 5.5 Hz, NHC$\boldsymbol{H}_2$); δ_C (151 MHz, $CDCl_3$) 172.1 (***C***=O), 143.3 (***C***-9′′), 140.4 (***C***-2′′), 139.1 (***C***-1^{Ph}), 138.1 (***C***-1′), 133.1 (***C***-4′), 130.4 (Ar***C***), 128.8(4) (Ar***C***), 128.7(7) (Ar***C***), 127.9 (***C***-7′′), 127.5 (Ar***C***), 126.4 (***C***-5′′), 115.9 (***C***-8′′), 114.4 (***C***-6′′), 110.9 (***C***-3′′), 57.9 (***C***-2), 36.6 (NH***C***H_2); *m/z* (LCMS, ESI$^+$) 376 (M(^{35}Cl)H$^+$), 378 (M(^{37}Cl)H$^+$); Accurate mass: Found MH$^+$, 376.1211: $C_{22}H_{19}N_3O^{35}Cl$ requires *M*, 376.1217.

N-([Pyridin-4′′-yl]methyl)-2-(4′-chlorophenyl)-2-phenylacetamide **S32**

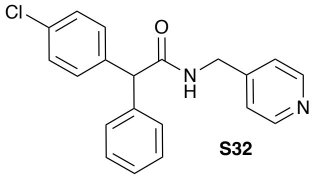

S32

Obtained in 40% yield as a-white solid. m.p. 148 – 149 °C; ν_{max} (ATR) 3094 (w), 3022 (w), 2946 (w), 1656 (s), 1371 (m), 1218 (m) cm^{-1}; δ_H (700 MHz, $CDCl_3$) 8.55 – 8.52 (2H, m, 2′′-$\boldsymbol{H}_2$), 7.37 – 7.34 (2H, m, 3^{Ph}-$\boldsymbol{H}_2$), 7.32 – 7.29 (3H, m, 3′-$\boldsymbol{H}_2$, 4^{Ph}-$\boldsymbol{H}_2$), 7.26 – 7.24 (2H, m, 2^{Ph}-$\boldsymbol{H}_2$), 7.23 – 7.20 (2H, m, 2′-$\boldsymbol{H}_2$), 7.11 – 7.09 (2H, m, 3′′-$\boldsymbol{H}_2$), 6.00 – 5.95 (1H, m, N***H***), 4.94 (1H, s, 2-***H***), 4.49 (2H, d, *J* = 6.0 Hz, NHC$\boldsymbol{H}_2$); δ_C (101 MHz, $CDCl_3$) 171.9 (***C***=O), 150.1 (***C***-2′′), 147.4 (***C***-4′′), 138.8 (***C***-1^{Ph}), 137.7 (***C***-1′), 133.5 (***C***-4′), 130.3 (***C***-2′), 129.2 (***C***-3^{Ph}), 129.0 (***C***-3′), 128.8 (***C***-2^{Ph}), 127.9 (***C***-4^{Ph}), 122.3 (***C***-3′′), 58.3 (***C***-2), 42.7 (NH***C***H_2); *m/z* (LCMS, ESI$^+$) 337 (M(^{35}Cl)H$^+$), 339 (M(^{37}Cl)H$^+$); Accurate mass: Found MH$^+$, 337.1110: $C_{20}H_{18}N_2O^{35}Cl$ requires *M*, 337.1108.

N-([3′′,5′′-Dimethylisoxazol-4′′-yl]methyl)-2-(4′-chlorophenyl)-2-phenylacetamide **S33**

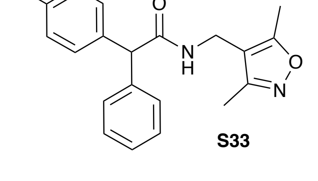

S33

Obtained in 14% yield as a-white solid. m.p. 169 – 171 °C; ν_{max} (ATR) 3290 (br), 3090 (w), 3044 (w), 2930 (w), 1647 (s), 1548 (m), 1491 (s), 1454 (m), 1200 (w), 1091 (w), 1015 (w) cm^{-1}; δ_H (600 MHz, $CDCl_3$) 7.35 –

7.31 (2H, m, 3^{Ph}-***H***$_2$), 7.31 – 7.27 (3H, m, 3′-***H***$_2$, 4^{Ph}-***H***), 7.20 (2H, d, *J* = 7.5 Hz, 2^{Ph}-***H***$_2$), 7.17 (2H, d, *J* = 8.5 Hz, 2′-***H***$_2$), 5.64 – 5.58 (1H, m, N***H***), 4.83 (1H, s, 2-***H***), 4.20 (2H, d, *J* = 5.5 Hz, NHC***H***$_2$), 2.33 (3H, s, 5′′-C***H***$_3$), 2.11 (3H, s, 3′′-C***H***$_3$); δ_C (151 MHz, $CDCl_3$) 171.4 (***C***=O), 167.3 (***C***-5′′), 159.4 (***C***-3′′), 138.8 (***C***-1^{Ph}), 137.6 (***C***-1′), 133.5 (***C***-4′), 130.3 (***C***-2′), 129.2 (***C***-3^{Ph}), 129.1 (***C***-3′), 128.8 (***C***-2^{Ph}), 127.9 (***C***-4^{Ph}), 110.8 (***C***-4′′), 58.5 (***C***-2), 32.5 (NH***C***H$_2$), 11.2 (5′′-***C***H$_3$), 10.2 (3′′-***C***H$_3$); *m/z* (LCMS, ESI$^+$) 355 (M(^{35}Cl)H$^+$), 357 (M(^{37}Cl)H$^+$); Accurate mass: Found MH$^+$, 355.1203: $C_{20}H_{20}N_2O_2{}^{35}Cl$ requires *M*, 355.1213.

N-(2′′-[Piperazin-1′′′-yl]eth-1′′-yl)-2-(4′-chlorophenyl)-2-phenylacetamide **S34**

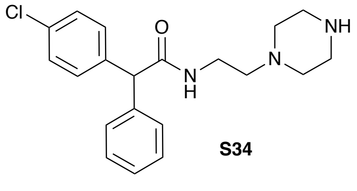

S34

Obtained in 24% yield as a yellow oil.ν_{max} (ATR) 3298 (br), 3040 (w), 2942 (w), 2818 (w), 1649 (s), 1556 (m), 1490 (s), 1444 (w), 1320 (w), 1226 (w), 1139 (w), 1089 (w) cm^{-1}; δ_H (700 MHz, $CDCl_3$) 7.35 – 7.31 (2H, m, 3^{Ph}-***H***$_2$), 7.31 – 7.27 (3H, m, 3′-***H***$_2$, 4^{Ph}-***H***), 7.25 – 7.22 (2H, m, 2^{Ph}-***H***$_2$), 7.22 – 7.20 (2H, m, 2′-***H***$_2$), 6.30 – 6.21 (1H, m, N***H***), 4.90 (1H, s, 2-***H***), 3.39 – 3.30 (2H, m, 1′′-***H***$_2$), 2.76 – 2.66 (4H, m, 3′′′-(***H***$_2$)$_2$), 2.40 (2H, app td, *J* = 6.0, 2.0 Hz, 2′′-***H***$_2$), 2.36 – 2.22 (4H, m, 2′′′-(***H***$_2$)$_2$); δ_C (176 MHz, $CDCl_3$) 171.5 (***C***=O), 139.3 (***C***-1_{Ph}), 138.3 (***C***-1′), 133.2 (***C***-4′), 130.5 (***C***-2′), 128.9(9) (***C***-3^{Ph}), 128.9(6) (Ar***C***), 128.9 (Ar***C***), 127.6 (***C***-4^{Ph}), 58.7 (***C***-2), 56.7 (***C***-2′′), 54.2 (***C***-2′′′), 46.2 (***C***-3′′′), 36.1 (***C***-1′′); *m/z* (LCMS, ESI$^+$) 358 (M(^{35}Cl)H$^+$), 360 (M(^{37}Cl)H$^+$); Accurate mass: Found MH$^+$, 358.1681: $C_{20}H_{25}N_3O^{35}Cl$ requires *M*, 358.1686.

N-([3′′,5′′-Dimethylpyrazol-4′′-yl]methyl)-2-(4′-chlorophenyl)-2-phenylacetamide **S35**

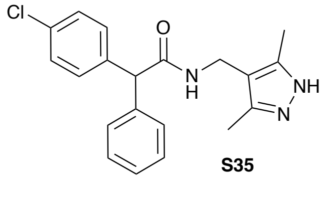

S35

Obtained in 34% yield as a a white solid. m.p. 177 – 178 °C; ν_{max} (ATR) 3262 (br), 3090 (w), 3032 (w), 2934 (w), 1637 (s), 1540 (m), 1494 (m), 1208 (m), 1090 (w) cm^{-1}; δ_H (700 MHz, $CDCl_3$) 7.34 – 7.31 (2H, m, 3^{Ph}-***H***$_2$), 7.30 – 7.27 (3H, m, 3′-***H***$_2$, 4^{Ph}-***H***), 7.22 – 7.20 (2H, m, 2^{Ph}-***H***$_2$), 7.19 – 7.17 (2H, m, 2′-***H***$_2$), 5.62 – 5.59 (1H, m, N***H***), 4.83 (1H, s, 2-***H***), 4.25 (2H, d, *J* = 5.2 Hz, NHC***H***$_2$), 2.18 (6H, s, 3′′-(C***H***$_3$)$_2$); δ_C (176 MHz, $CDCl_3$) 171.3 (***C***=O), 143.4 (***C***-3′′), 139.0 (***C***-1^{Ph}), 137.9 (***C***-1′), 133.4 (***C***-4′), 130.3 (***C***-2′), 129.1 (Ar***C***), 129.0 (Ar***C***), 128.8 (Ar***C***), 127.8 (Ar***C***), 112.8 (***C***-4′′), 58.5 (***C***-2), 33.2 (NH***C***H$_2$), 10.7 (3′′-(***C***H$_3$)$_2$); *m/z* (LCMS, ESI$^+$) 354 (M(^{35}Cl)H$^+$), 356 (M(^{37}Cl)H$^+$); Accurate mass: Found MH$^+$, 354.1384: $C_{20}H_{21}N_3O^{35}Cl$ requires *M*, 354.1373.

N-([1′′-{1′′′,2′′′,4′′′-Triazol-1′′′-yl}phen-2′′-yl]methyl)-2-(4′-chlorophenyl)-2-phenylacetamide **S36**

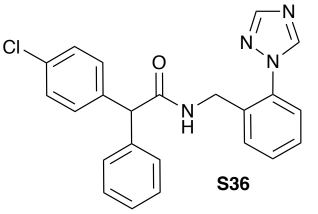

S36

Obtained as a white solid. m.p. 137 – 139 °C; ν_{max} (ATR) 3286 (br), 3098 (w), 3044 (w), 2934 (w), 1662 (s), 1494 (s), 1352 (w), 1276 (m), 1215 (m) cm^{-1}; δ_H (700 MHz, $CHCl_3$) 8.30 (1H, s, Ar***H***), 7.90 (1H, s, Ar***H***), 7.66 (1H, dd, *J* = 7.5, 1.5 Hz, 3′′-***H***), 7.47 (1H, td, *J* = 7.5, 1.5 Hz, 4′′-***H***), 7.43 (1H, td, *J* = 7.5, 1.5 Hz, 5′′-***H***), 7.32 – 7.24 (6H, m, Ar***H***), 7.18 – 7.16 (2H, m, 2^{Ph}-$\boldsymbol{H_2}$), 7.14 – 7.12 (2H, m, 2′-$\boldsymbol{H_2}$), 6.94 (1H, t, *J* = 6.5 Hz, N***H***), 4.87 (1H, s, 2-***H***), 4.32 (2H, d, *J* = 6.5 Hz, NHC$\boldsymbol{H_2}$); δ_C (176 MHz, $CDCl_3$) 171.4 (***C***=O), 152.5 (Ar***C***), 143.8 (Ar***C***), 139.1 (Ar***C***), 138.1 (Ar***C***), 135.9 (***C***-2′′), 133.6 (***C***-1′′), 133.2 (***C***-4′), 132.4 (***C***-3′′), 130.4 (***C***-2′), 130.2 (***C***-4′′), 129.1 (Ar***C***), 128.9(74) (Ar***C***), 128.9(67) (Ar***C***), 128.9 (Ar***C***), 127.5 (Ar***C***), 124.9 (***C***-6′′), 58.6 (***C***-2), 40.5 (NH***C***H_2); *m/z* (LCMS, ESI^+) 403 ($M(^{35}Cl)H^+$), 405 ($M(^{37}Cl)H^+$); Accurate mass: Found MH^+, 403.1327: $C_{23}H_{20}N_4O^{35}Cl$ requires *M*, 403.1326.

N-(3′′-[Thiophene-2′′′-carboxamido]prop-1′′-yl)-2-(4′-chlorophenyl)-2-phenylacetamide **S37**

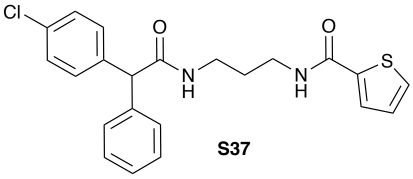

S37

Obtained in 54% yield as a white solid. m.p. 164 – 165 °C; ν_{max} (ATR) 3304 (br), 3076 (w), 2938 (w), 1626 (s), 1543 (s), 1490 (s), 1446 (w), 1353 (w), 1303 (m), 1234 (w), 1090 (m), 1015 (m) cm^{-1}; δ_H (600 MHz, $CHCl_3$) 7.53 (1H, dd, *J* = 3.5, 1.0 Hz, 3′′′-***H***), 7.47 – 7.43 (1H, m, 5′′′-***H***), 7.34 – 7.31 (2H, m, 3^{Ph}-$\boldsymbol{H_2}$), 7.29 – 7.24 (5H, m, 3′-$\boldsymbol{H_2}$, 2^{Ph}-$\boldsymbol{H_2}$, 4^{Ph}-***H***), 7.23 – 7.21 (2H, m, 2′-$\boldsymbol{H_2}$), 7.13 – 7.07 (1H, m, 3′′-N***H***), 7.06 – 7.00 (1H, m, 4′′′-***H***), 6.38 – 6.30 (1H, m, 1-N***H***), 4.89 (1H, s, 2-***H***), 3.40 – 3.33 (4H, m, 1′′-$\boldsymbol{H_2}$, 3′′-$\boldsymbol{H_2}$), 1.70 – 1.63 (2H, m, 2′′-$\boldsymbol{H_2}$); δ_C (151 MHz, $CDCl_3$) 172.8 (***C***-1), 162.5 (2′′′-***C***O), 139.3 (***C***-2′′′), 139.0 (***C***-1^{Ph}), 137.9 (***C***-1′), 133.4 (***C***-4′), 130.3 (***C***-2′), 130.2 (***C***-3^{Ph}), 129.1 (Ar***C***), 129.0 (Ar***C***), 128.8 (***C***-2^{Ph}), 128.1 (***C***-3′′′), 127.8 (***C***-4′′′), 127.7 (***C***-4^{Ph}), 58.6 (***C***-2), 36.5 (***C***-1′′), 36.1 (***C***-3′′), 29.9 (***C***-2′′); *m/z* (LCMS, ESI^+) 413 ($M(^{35}Cl)H^+$), 415 ($M(^{37}Cl)H^+$); Accurate mass: Found MH^+, 413.1097: $C_{22}H_{22}N_2O_2S^{35}Cl$ requires *M*, 413.1091.

N-[1′-(2′′,2′′-Difluoroethyl)piperidin-4′-yl]-2-(4′-chlorophenyl)-2-phenylacetamide **S38**

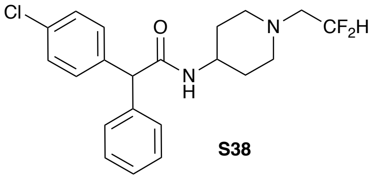

S38

Obtained in 32% yield as an off-white solid. m.p. 143 – 145 °C; ν_{max} (ATR) 3296 (br), 3058 (w), 2946 (w), 2794 (w), 1639 (s), 1541 (m), 1489 (s), 1290 (w), 1125 (m), 1089 (m), 1049 (s), 1015 (m) cm^{-1}; δ_H (400 MHz, $CDCl_3$) 7.36 – 7.26 (5H, m, Ar***H***), 7.23 – 7.16 (4H, m, Ar***H***), 5.84 (2H, tt, *J* = 56.0, 4.0 Hz, 2′′-***H***), 5.55 – 5.44 (1H, m, N***H***), 4.83 (1H, s, 2-***H***), 3.91 – 3.78 (1H, m, 4′-***H***), 2.90 – 2.79 (2H, m, 2′-***H***$_2$H′$_2$), 2.72 (2H, td, *J* = 15.0, 4.0 Hz, 1′′-***H***$_2$), 2.38 – 2.27 (2H, m, 2′-H$_2$***H′***$_2$), 1.97 – 1.84 (2H, m, 3′-***H***$_2$H′$_2$), 1.48 – 1.32 (2H, m, 3′-H$_2$***H′***$_2$); δ_C (101 MHz, $CDCl_3$) 170.9 (***C***=O), 139.1 (Ar***C***), 138.0 (Ar***C***), 133.3 (Ar***C***), 130.3 (Ar***C***), 129.1 (Ar***C***), 129.0 (Ar***C***), 128.8 (Ar***C***), 127.7 (Ar***C***), 115.5 (t, *J* = 241.5 Hz, ***C***-2′′), 59.9 (t, *J* = 25.0 Hz, ***C***-1′′), 58.5 (***C***-2), 53.2 (***C***-2′), 46.3 (***C***-4′), 31.9 (***C***-3′); δ_F (376 MHz, $CDCl_3$) −118.52 (dt, *J* = 56.0, 15.0 Hz, 2′′-***F***$_2$); *m/z* (LCMS, ESI$^+$) 393 (M(^{35}Cl)H$^+$), 395 (M(^{37}Cl)H$^+$); Accurate mass: Found MH$^+$, 393.1546: $C_{21}H_{24}N_2OF_2{}^{35}Cl$ requires *M*, 393.1545.

1-(4′′-[Pyrimidin-2′′′-yl]piperazin-1′′-yl)-2-(4′-chlorophenyl)-2-phenylethan-1-one **S39**

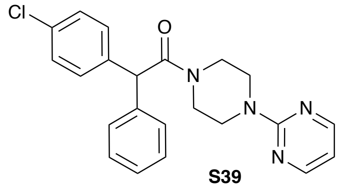

S39

Obtained in 23% yield as an off-white solid. m.p. 150 – 152 °C; ν_{max} (ATR) 3020 (w), 2916 (w), 2856 (w), 1645 (s), 1582 (s), 1548 (s), 1489 (s), 1428 (s), 1392 (m), 1355 (m), 1307 (w), 1261 (m), 1222 (w), 1089 (w), 1032 (w) cm^{-1}; δ_H (600 MHz, $CDCl_3$) 8.28 (2H, d, *J* = 4.5 Hz, 4′′′-***H***$_2$), 7.33 (2H, t, *J* = 7.5 Hz, 3^{Ph}-***H***$_2$), 7.29 – 7.22 (5H, m, 3′-***H***$_2$, 2^{Ph}-***H***$_2$, 4^{Ph}-***H***), 7.19 – 7.15 (2H, m, 2′-***H***$_2$), 6.51 (1H, t, *J* = 4.5 Hz, 5′′′-***H***), 5.21 (1H, s, 2-***H***), 3.90 – 3.79 (2H, m, 2′′-(***H***H′)H$_2$, 3′′-(***H***H′)(HH′)), 3.78 – 3.73 (1H, m, 3′′-(H***H′***)(HH′)), 3.70 (1H, ddd, *J* = 13.0, 7.5, 3.0 Hz, 2′′-(H***H′***)H$_2$), 3.64 (1H, dt, *J* = 13.0, 5.0 Hz, 3′′-(HH′)(***H***H′)), 3.51 (2H, t, *J* = 5.0 Hz, 2′′-(HH′)***H***$_2$), 3.45 (1H, dt, *J* = 13.0, 5.0 Hz, 3′′-(HH′)(H***H′***)); δ_C (151 MHz, $CDCl_3$) 170.2 (***C***=O), 161.6 (***C***-2′′′), 157.9 (***C***-4′′′), 138.8 (***C***-1^{Ph}), 138.1 (***C***-1′), 133.2 (***C***-4′), 130.6 (***C***-2′), 129.0 (***C***-3^{Ph}), 128.9 (Ar***C***), 128.8 (Ar***C***), 127.5 (***C***-4^{Ph}), 110.6 (***C***-5′′′), 54.5 (***C***-2), 45.9 (***C***-2′′), 43.6 (***C***-3′′), 42.22 (***C***-2′′); *m/z* (LCMS, ESI$^+$) 393 (M(^{35}Cl)H$^+$), 395 (M(^{37}Cl)H$^+$); Accurate mass: Found MH$^+$, 393.1470: $C_{22}H_{22}N_4O^{35}Cl$ requires *M*, 393.1482.

1-(4′′-[2′′′-Hydroxyethyl]piperazin-1′′-yl)-2-(4′-chlorophenyl)-2-phenylethan-1-one **S40**

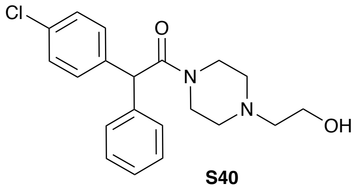

S40

Obtained in 49% yield as a yellow oil. ν_{max} (ATR) 3412 (br), 2938 (w), 2868 (w), 2804 (w), 1638 (s), 1490 (m), 1434 (m), 1290 (w), 1223 (m), 1144 (w), 1087 (m), 1014 (m) cm^{-1}; δ_H (600 MHz, $CDCl_3$) 7.35 – 7.31 (2H, m, 3^{Ph}-***H***$_2$), 7.29 – 7.26 (3H, m, 3′-***H***$_2$, 4^{Ph}-***H***), 7.22 – 7.20 (2H, m, 2^{Ph}-***H***$_2$), 7.16 – 7.13 (2H, m, 2′-***H***$_2$), 5.16 (1H, s, 2-***H***), 3.75 (1H, ddd, *J* = 13.5, 6.5, 3.5 Hz, 2′′-(***H***H′)H$_2$), 3.67 (1H, ddd, *J* = 13.5, 6.5, 3.5 Hz, 2′′-(H***H′***)H$_2$), 3.59 (2H, t, *J* = 5.5 Hz, 2′′′-***H***$_2$), 3.49 – 3.41 (2H, m, 2′′-(HH′)***H***$_2$), 2.53 – 2.42 (5H, m, 3′′-***H***$_2$(HH′), 1′′′-***H***$_2$, O***H***), 2.32 – 2.25 (1H, m, 3′′-H$_2$(***H***H′)), 2.22 – 2.16 (1H, m, 3′′-H$_2$(H***H′***)); δ_C (151 MHz, $CDCl_3$) 169.9 (***C***=O), 138.9 (***C***-1^{Ph}), 138.2 (***C***-1′), 133.1 (***C***-4′), 130.6 (***C***-2′), 129.0 (***C***-3^{Ph}), 128.9 (***C***-2^{Ph}), 128.8 (***C***-3′), 127.5 (***C***-4^{Ph}), 59.3 (***C***-1′′′), 57.9 (***C***-2′′′), 54.3 (***C***-2), 52.9 (***C***-3′′), 52.7 (***C***-3′′), 46.1 (***C***-2′′), 42.4 (***C***-2′′); *m/z* (LCMS, ESI$^+$) 359 (M(^{35}Cl)H$^+$), 361 (M(^{37}Cl)H$^+$); Accurate mass: Found MH$^+$, 359.1532: $C_{20}H_{24}N_2O_2{}^{35}Cl$ requires *M*, 359.1526.

1-(4′′-[2′′′-Methoxyethyl]piperazin-1′′-yl)-2-(4′-chlorophenyl)-2-phenylethan-1-one **S41**

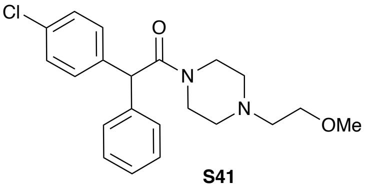

S41

Obtained in 11% yield as a yellow oil. ν_{max} (ATR) 2928 (w), 2882 (w), 2808 (w), 1639 (s), 1490 (m), 1432 (m), 1308 (w), 1225 (w), 1113 (m), 1015 (m) cm^{-1}; δ_H (600 MHz, $CDCl_3$) 7.32 – 7.29 (2H, m, 3^{Ph}-***H***$_2$), 7.27 – 7.22 (3H, m, 3′-***H***$_2$, 4^{Ph}-***H***), 7.21 – 7.18 (2H, m, 2^{Ph}-***H***$_2$), 7.14 – 7.11 (2H, m, 2′-***H***$_2$), 5.14 (1H, s, 2-***H***), 3.75 (1H, ddd, *J* = 13.0, 6.0, 3.5 Hz, 2′′-(***H***H′)H$_2$), 3.66 (1H, ddd, *J* = 13.0, 7.0, 3.5 Hz, 2′′-(H***H′***)H$_2$), 3.49 – 3.39 (2H, m, 2′′-(HH′)***H***$_2$), 3.45 (2H, t, *J* = 5.5 Hz, 2′′′-***H***$_2$), 3.31 (3H, s, OC***H***$_3$), 2.50 (2H, t, *J* = 5.5 Hz, 1′′′-***H***$_2$), 2.46 (1H, ddd, *J* = 11.0, 6.5, 3.5 Hz, 3′′-(***H***H′)(HH′)), 2.42 (1H, ddd, *J* = 11.0, 7.0, 3.5 Hz, 3′′-(H***H′***)(HH′)), 2.26 (1H, ddd, *J* = 11.0, 6.5, 3.5 Hz, 3′′-(HH′)(***H***H′)), 2.16 (1H, ddd, *J* = 11.0, 6.0, 4.0 Hz, 3′′-(HH′)(H***H′***)); δ_C (151 MHz, $CDCl_3$) 169.8 (***C***=O), 139.0 (***C***-1^{Ph}), 138.3 (***C***-1′), 133.0 (***C***-4′), 130.6 (***C***-2′), 128.8(8) (Ar***C***), 128.8(7) (Ar***C***), 128.7 (***C***-3′), 127.4 (***C***-4^{Ph}), 70.1 (***C***-2′′′), 59.0 (O***C***H$_3$), 57.9 (***C***-1′′′), 54.2 (***C***-2), 53.5 (***C***-3′′), 53.2 (***C***-3′′), 46.0 (***C***-2′′), 42.2 (***C***-2′′); *m/z* (LCMS, ESI$^+$) 373 (M(^{35}Cl)H$^+$), 375 (M(^{37}Cl)H$^+$); Accurate mass: Found MH$^+$, 373.1671: $C_{21}H_{26}N_2O_2{}^{35}Cl$ requires *M*, 373.1683.

N-Methyl-N-([1′′-difluoromethylimidazol-2′′-yl]methyl)-2-(4′-chlorophenyl)-2-phenylacetamide **S42**

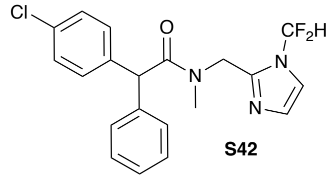

Obtained in 47% yield as a colourless oil. ν_{max} (ATR) 3136 (w), 2942 (w), 1639 (s), 1489 (s), 1460 (m), 1416 (m), 1348 (w), 1273 (s), 1192 (w), 1160 (m), 1091 (s), 1047 (s) cm^{-1}; δ_H (600 MHz, $CDCl_3$) 7.77 (1H, t, *J* = 59.0 Hz, C***H***F_2), 7.34 – 7.30 (2H, m, 3^{Ph}-***H***$_2$), 7.29 – 7.26 (4H, m, 3′-***H***$_2$, 4^{Ph}-***H***, 5′′-***H***), 7.18 – 7.14 (2H, m, 2^{Ph}-***H***$_2$), 7.12 – 7.08 (2H, m, 2′-***H***$_2$), 7.04 (1H, d, *J* = 1.5 Hz, 4′′-***H***), 5.17 (1H, s, 2-***H***), 4.78 (1H, d, *J* = 15.0 Hz, N(CH_3)C***H***H′), 4.72 (1H, d, *J* = 15.0 Hz, N(CH_3)CH***H′***), 3.05 (3H, s, NC***H***$_3$CHH′); δ_C (151 MHz, $CDCl_3$) 172.2 (***C***=O), 143.1 (***C***-2′′), 138.1 (***C***-1^{Ph}), 137.4 (***C***-1′), 133.4 (***C***-4′), 130.4 (***C***-2′), 129.7 (***C***-4′′), 129.1 (***C***-3^{Ph}), 128.9 (***C***-3′), 128.7 (***C*** 2^{Ph}), 127.7 (***C***-4^{Ph}), 116.1 (***C***-5′′), 108.4 (t, *J* = 250.0 Hz, ***C***HF_2), 54.3 (***C***-2), 43.4 (N(CH_3)***C***HH′), 35.3 (N***C***H_3CHH′); δ_F (376 MHz, $CDCl_3$) −91.65 (dd, *J* = 231.5, 59.0 Hz, CH***F***F′), −92.72 (dd, *J* = 231.5, 59.0 Hz, CHF***F′***); *m/z* (LCMS, ESI^+) 390 ($M(^{35}Cl)H^+$), 392 ($M(^{37}Cl)H^+$); Accurate mass: Found MH^+, 390.1181: $C_{20}H_{19}N_3OF_2{}^{35}Cl$ requires *M*, 390.1185.

N-Methyl-N-([1′′-methylpyrazol-4′′-yl]methyl)-2-(4′-chlorophenyl)-2-phenylacetamide **S43**

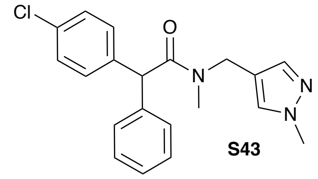

Obtained in 38% yield as a colourless oil. ν_{max} (ATR) 3050 (w), 2932 (w), 2868 (w), 1639 (s), 1489 (s), 1446 (m), 1394 (s), 1334 (w), 1252 (w), 1161 (m), 1088 (m), 1015 (m) cm^{-1}; δ_H (600 MHz, $CDCl_3$, mixture of rotamers) 7.39 (0.7H, s, 3′′-***H*** rotamer A), 7.33 (0.7H, s, 5′′-***H*** rotamer A), 7.32 – 7.28 (2H, m, 3^{Ph}-***H***$_2$), 7.28 – 7.22 (3.6H, m, 3′-***H***$_2$, 2^{Ph}-***H***$_2$ rotamer B, 4^{Ph}-***H***), 7.21 (0.3H, s, 3′′-***H*** rotamer B), 7.22 – 7.17 (1.4H, m, 2^{Ph}-***H***$_2$ rotamer A), 7.16 – 7.14 (0.6H, m, 2′-***H***$_2$ rotamer B), 7.15 – 7.12 (1.4H, m, 2′-***H***$_2$ rotamer A), 6.90 (0.3H, s, 5′′-***H*** rotamer B), 5.25 (0.3H, s, 2-***H*** rotamer B), 5.13 (0.7H, s, 2-***H*** rotamer A), 4.45 (0.7H, d, *J* = 14.5 Hz, N(CH_3)C***H***H′ rotamer A), 4.41 (0.7H, d, *J* = 14.5 Hz, N(CH_3)CH***H′*** rotamer A), 4.37 (0.6H, s, N(CH_3)C***H***$_2$ rotamer B), 3.85 (2.1H, s, 1′′-C***H***$_3$ rotamer A), 3.83 (0.9H, s, 1′′-C***H***$_3$ rotamer B), 2.98 (0.9H, s, NC***H***$_3$$CH_2$ rotamer B), 2.92 (2.1H, s, NC***H***$_3$CHH′ rotamer A); δ_C (151 MHz, $CDCl_3$, mixture of rotamers) 171.2 (***C***=O), 139.3 (***C***-3′′ rotamer A), 139.1 (***C***-1^{Ph} rotamer B), 138.9 (***C***-1^{Ph} rotamer A), 138.4 (***C***-1′ rotamer B), 138.2 (***C***-1′ rotamer A), 138.1 (***C***-3′′ rotamer B), 133.1 (***C***-4′ rotamer B), 133.0 (***C***-4′ rotamer A), 130.5(7) (***C***-2′ rotamer B), 130.5(5) (***C***-2′ rotamer A), 130.0 (***C***-5′′ rotamer A), 129.0 (Ar***C***), 128.9(1) (Ar***C***), 128.8(8) (Ar***C***), 128.8(5) (Ar***C***), 128.7 (Ar***C***), 128.5 (***C***-5′′ rotamer B), 127.5 (Ar***C***), 127.4 (Ar***C***), 117.4 (***C***-4′′ rotamer B), 117.3

(***C***-4′′ rotamer A), 54.4 (***C***-2 rotamer A), 54.1 (***C***-2 rotamer B), 44.8 (N(CH_3)***C***H_2 rotamer B), 42.4 (N(CH_3)***C***HH′ rotamer A), 39.2 (1′′-***C***H_3 rotamer B), 39.0 (1′′-***C***H_3 rotamer A), 35.3 (N(***C***H_3)CHH′ rotamer A), 34.1 (N(***C***H_3)CH_2 rotamer B); *m/z* (LCMS, ESI^+) 354 ($M(^{35}Cl)H^+$), 356 ($M(^{37}Cl)H^+$); Accurate mass: Found MH^+, 354.1367: $C_{20}H_{21}N_3O^{35}Cl$ requires *M*, 354.1373.

N-Methyl-N-([pyridin-3′′-yl]methyl)-2-(4′-chlorophenyl)-2-phenylacetamide **S44**

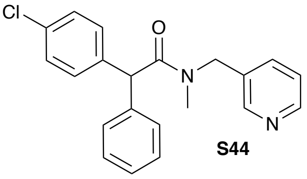

Obtained in 20% yield as a colourless oil. ν_{max} (ATR) 3036 (w), 2932 (w), 1642 (s), 1489 (m), 1396 (m), 1270 (w), 1174 (w), 1089 (m), 1015 (w) cm^{-1}; δ_H (600 MHz, $CDCl_3$, mixture of rotamers) 8.59 – 8.56 (0.3H, m, 6′′-***H*** rotamer B), 8.54 – 8.51 (0.7H, m, 6′′-***H*** rotamer A), 8.51 – 8.47 (0.7H, m, 2′′-***H*** rotamer A), 8.44 – 8.42 (0.3H, m, 2′′-***H*** rotamer B), 7.63 – 7.58 (0.7H, m, 4′′-***H*** rotamer A), 7.38 – 7.34 (0.3H, m, 4′′-***H*** rotamer B), 7.34 – 7.29 (2H, m, 3^{Ph}-***H_2***), 7.29 – 7.24 (4H, m, 3′-***H_2***, 4^{Ph}-***H***, 5′′-***H***), 7.23 – 7.22 (1.4H, m, 2^{Ph}-***H_2*** rotamer A), 7.23 – 7.19 (0.6H, m, 2^{Ph}-***H_2*** rotamer B), 7.19 – 7.15 (1.4H, m, 2′-***H_2*** rotamer A), 7.14 – 7.10 (0.6H, m, 2′-***H_2*** rotamer B), 5.20 (0.7H, s, 2-***H*** rotamer A), 5.11 (0.3H, s, 2-***H*** rotamer B), 4.66 (0.7H, d, *J* = 15.0 Hz, N(CH-$_3$)C***H***H′ rotamer A), 4.60 (0.7H, d, *J* = 15.0 Hz, N(CH_3)CH***H′*** rotamer A), 4.58 (0.3H, d, *J* = 17.0 Hz, N(CH_3)C***H***H′ rotamer B), 4.51 (0.3H, d, *J* = 17.0 Hz, N(CH_3)CH***H′*** rotamer B), 3.02 (0.9H, s, N(C***H_3***)CHH′ rotamer B), 2.94 (2.1H, s, N(C***H_3***)CHH′ rotamer A); δ_C (151 MHz, $CDCl_3$, mixture of rotamers) 171.8(4) (***C***=O rotamer A), 171.8(2) (***C***=O rotamer B), 149.6 (***C***-2′′ rotamer A), 149.5 (***C***-6′′ rotamer B), 149.2 (***C***-6′′ rotamer A), 148.4 (***C***-2′′ rotamer B), 138.7 (***C***-1^{Ph} rotamer B), 138.6 (***C***-1^{Ph} rotamer A), 138.0 (***C***-1′), 136.1 (***C***-4′′ rotamer A), 134.0 (***C***-4′′ rotamer B), 133.3 (***C***-4′ rotamer B), 133.2 (***C***-4′ rotamer A), 132.9 (***C***-3′′ rotamer A), 132.2 (***C***-3′′ rotamer B), 130.5(0) (***C***-2′ rotamer A), 130.4(5) (***C***-2′ rotamer B), 129.1 (Ar***C***), 129.0 (Ar***C***), 128.8(1) (Ar***C***), 128.7(8) (Ar***C***), 127.7 (Ar***C***), 127.5 (Ar***C***), 123.9 (***C***-5′′ rotamer B), 123.8 (***C***-5′′ rotamer A), 54.4 (***C***-2 rotamer A), 54.3 (***C***-2 rotamer B), 51.3 (N(CH_3)***C***HH′ rotamer B), 49.4 (N(CH_3)***C***HH′ rotamer A), 35.5 (N(***C***H_3)HH′ rotamer A), 34.8 (N(***C***H_3)HH′ rotamer B); *m/z* (LCMS, ESI^+) 351 ($M(^{35}Cl)H^+$), 353 ($M(^{37}Cl)H^+$); Accurate mass: Found MH^+, 351.1259: $C_{21}H_{20}N_2O^{35}Cl$ requires *M*, 351.1264.

N-Methyl-N-([3′′-fluoropyridin-2′′-yl]methyl)-2-(4′-chlorophenyl)-2-phenylacetamide **S45**

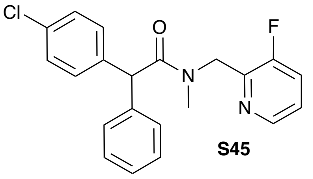

S45

Obtained in 43% yield as colourless oil. ν_{max} (ATR) 3058 (w), 2928 (w), 1647 (s), 1489 (m), 1443 (s), 1396 (m), 1243 (w), 1159 (w), 1090 (m), 1015 (w) cm^{-1}; δ_H (600 MHz, $CDCl_3$, mixture of rotamers) 8.45 (0.5H, dt, *J* = 5.0, 1.5 Hz, 6′′-***H*** rotamer A), 8.34 (0.5H, dt, *J* = 5.0, 1.5 Hz, 6′′-***H*** rotamer B), 7.40 (0.5H, ddd, *J* = 9.5, 8.5, 1.5 Hz, 4′′-***H*** rotamer A), 7.34 (0.5H, ddd, *J* = 9.5, 8.5, 1.5 Hz, 4′′-***H*** rotamer B), 7.32 – 7.28 (2.5H, m, 5′′-***H*** rotamer A, 3^{Ph}-$\boldsymbol{H}_2$), 7.27 – 7.22 (5H, m, 3′-$\boldsymbol{H}_2$, 2^{Ph}-$\boldsymbol{H}_2$, 4^{Ph}-***H***), 7.22 – 7.18 (1.5H, m, 2′-$\boldsymbol{H}_2$ rotamer B, 5′′-***H*** rotamer B), 7.18 – 7.16 (1H, m, 2′-$\boldsymbol{H}_2$ rotamer A), 5.60 (0.5H, s, 2-***H*** rotamer A), 5.27 (0.5H, s, 2-***H*** rotamer B), 4.89 (0.5H, dd, *J* = 15.5, 2.0 Hz, N(CH_3)C***H***H′ rotamer B), 4.79 (0.5H, dd, *J* = 15.5, 1.5 Hz, N(CH_3)CH***H′*** rotamer B), 4.70 (0.5H, dd, *J* = 16.5, 1.5 Hz, N(CH_3)C***H***H′ rotamer A), 4.54 (0.5H, dd, *J* = 16.5, 1.5 Hz, N(CH_3)CH***H′*** rotamer A), 3.08 (1.5H, s, N(C$\boldsymbol{H}_3$)CHH′ rotamer B), 3.04 (1.5H, s, N(C$\boldsymbol{H}_3$)CHH′ rotamer A); δ_C (151 MHz, $CDCl_3$, mixture of rotamers) 172.6 (***C***=O rotamer A), 171.8 (***C***=O rotamer B), 157.9 (d, *J* = 258.5 Hz, ***C***-3′′ rotamer B), 157.7 (d, *J* = 257.5 Hz, ***C***-3′′ rotamer A), 145.7 (d, *J* = 5.5 Hz, ***C***-6′′ rotamer A), 145.2 (d, *J* = 14.5 Hz, ***C***-2′′ rotamer B), 145.0 (d, *J* = 5.5 Hz, ***C***-6′′ rotamer B), 144.5 (d, *J* = 14.5 Hz, ***C***-2′′ rotamer A), 139.2 (***C***-1^{Ph} rotamer A), 139.1 (***C***-1^{Ph} rotamer B), 138.7 (***C***-1′ rotamer A), 138.4 (***C***-1′ rotamer B), 132.9(3) (***C***-4′ rotamer B), 132.8(6) (***C***-4′ rotamer A), 130.8 (***C***-2′ rotamer B), 130.7 (***C***-2′ rotamer A), 129.2 (Ar***C***), 129.0 (Ar***C***), 128.8 (***C***-3^{Ph}), 128.7 (***C***-3^{Ph}), 128.6(1) (Ar***C***), 128.5(8) (Ar***C***), 127.3 (Ar***C***), 124.6 (d, *J* = 3.5 Hz, ***C***-5′′ rotamer A), 123.8 (d, *J* = 3.5 Hz, ***C***-5′′ rotamer B), 123.3 (d, *J* = 19.0 Hz, ***C***-4′′ rotamer A), 122.9 (d, *J* = 18.5 Hz, ***C***-4′′ rotamer B), 54.4 (***C***-2 rotamer B), 53.8 (***C***-2 rotamer A), 49.4 (d, *J* = 1.5 Hz, N(CH_3)***C***HH′ rotamer A), 48.1 (d, *J* = 1.0 Hz, N(CH_3)***C***HH′ rotamer B), 36.8 (N(***C***H_3)CHH′ rotamer B), 35.2 (N(***C***H_3)CHH′ rotamer A); δ_F (376 MHz, $CDCl_3$) −125.79 (3′′-***F***); *m/z* (LCMS, ESI$^+$) 369 (M(^{35}Cl)H$^+$), 371 (M(^{37}Cl)H$^+$); Accurate mass: Found MH$^+$, 369.1154: $C_{21}H_{19}N_2OF^{35}Cl$ requires *M*, 369.1170.

N-Methyl-N-(2′′-[morpholin-4′′′-yl]eth-1′′-yl)-2-(4′-chlorophenyl)-2-phenylacetamide **S46**

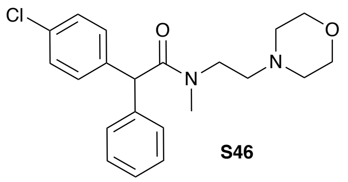

S46

Obtained in 12% yield as a yellow oil. ν_{max} (ATR) 2958 (w), 2855 (w), 2812 (w), 1643 (s), 1489 (m), 1456 (w), 1399 (m), 1304 (w), 1260 (w), 1152 (w), 1116 (s), 1015 (w) cm^{-1}; δ_H (600 MHz, $CDCl_3$, mixture of rotamers) 7.34 – 7.30 (2H, m, 3^{Ph}-$\boldsymbol{H}_2$), 7.28 – 7.23 (5H, m, 3′-$\boldsymbol{H}_2$, 2^{Ph}-$\boldsymbol{H}_2$, 4^{Ph}-***H***), 7.19 – 7.15 (2H, m, 2′-$\boldsymbol{H}_2$),

5.28 (0.3H, s, 2-***H*** rotamer B), 5.18 (0.7H, s, 2-***H*** rotamer A), 3.71 – 3.64 (4H, m, 2‴-(***H***$_2$)$_2$), 3.60 (0.7H, dt, *J* = 13.5, 6.5 Hz, 1″-***H***H′ rotamer A), 3.53 (0.7H, dt, *J* = 13.5, 6.5 Hz, 1″-H***H′*** rotamer A), 3.43 (0.3H, ddd, *J* = 14.5, 7.5, 6.0 Hz, 1″-***H***H′ rotamer B), 3.36 (0.3H, ddd, *J* = 14.5, 7.5, 6.0 Hz, 1″-H***H′*** rotamer B), 3.01 (0.9H, s, NC***H***$_3$ rotamer B), 2.99 (2.1H, s, NC***H***$_3$ rotamer A), 2.51 (1.4H, t, *J* = 6.5 Hz, 2″-***H***$_2$ rotamer A), 2.52 – 2.45 (2.8H, m, 3‴-(***H***$_2$)$_2$ rotamer A), 2.45 (0.3H, ddd, *J* = 13.0, 7.5, 6.0 Hz, 2″-***H***H′ rotamer B), 2.42 – 2.39 (1.2H, m, 3‴-(***H***$_2$)$_2$ rotamer B), 2.37 (0.3H, ddd, *J* = 13.0, 7.5, 6.0 Hz, 2″-H***H′*** rotamer B); δ_C (151 MHz, $CDCl_3$, mixture of rotamers) 171.5 (***C***=O rotamer B), 171.4 (***C***=O rotamer A), 139.2 (***C***-1^{Ph} rotamer B), 139.0 (***C***-1^{Ph} rotamer A), 138.6 (***C***-1′ rotamer B), 138.4 (***C***-1′ rotamer A), 133.0(4) (***C***-4′ rotamer B), 132.9(7) (***C***-4′ rotamer A), 130.7 (***C***-2′ rotamer A), 130.5 (***C***-2′ rotamer B), 129.0 (Ar***C***), 128.9 (Ar***C***), 128.8(4) (Ar***C***), 128.8(0) (Ar***C***), 128.7(0) (Ar***C***), 128.6(6) (Ar***C***), 127.4 (Ar***C***), 127.3 (Ar***C***), 67.2 (***C***-2‴ rotamer A), 67.0 (***C***-2‴ rotamer B), 57.3 (***C***-2″ rotamer B), 55.8 (***C***-2″ rotamer A), 54.5 (***C***-2 rotamer A), 54.2 (***C***-3‴ rotamer B), 53.8(9) (***C***-3‴ rotamer A), 53.8(6) (***C***-2 rotamer B), 47.6 (***C***-1″ rotamer B), 45.3 (***C***-1″ rotamer A), 36.2 (N***C***H$_3$ rotamer A), 34.6 (N***C***H$_3$ rotamer B); *m/z* (LCMS, ESI$^+$) 373 (M(^{35}Cl)H$^+$), 375 (M(^{37}Cl)H$^+$); Accurate mass: Found MH$^+$, 373.1673: $C_{21}H_{26}N_2O_2{}^{35}Cl$ requires *M*, 373.1683.

N-(1″,3″-Dihydro-2″-[2‴-fluoroethyl]isoindol-5″-yl)-2-(4′-chlorophenyl)-2-phenylacetamide **S47**

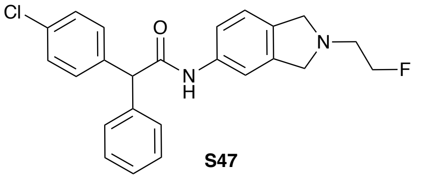

S47

Obtained in 9% yield as a brown oil. ν_{max} (ATR) 3274 (br), 3054 (w), 2946 (w), 2808 (w), 1661 (s), 1602 (w), 1550 (m), 1491 (s), 1442 (w), 1364 (w), 1210 (w), 1096 (w), 1032 (w) cm^{-1}; δ_H (700 MHz, $CDCl_3$) 7.48 (1H, d, *J* = 2.0 Hz, 4″-***H***), 7.38 – 7.35 (2H, m, 3^{Ph}-***H***$_2$), 7.33 – 7.31 (3H, m, 3′-***H***$_2$, 4^{Ph}-***H***), 7.31 – 7.29 (2H, m, 2^{Ph}-***H***$_2$), 7.26 – 7.24 (2H, m, 2′-***H***$_2$), 7.13 (1H, dd, *J* = 8.0, 2.0 Hz, 6″-***H***), 7.10 (1H, d, *J* = 8.0 Hz, 7″-***H***), 5.01 (1H, s, 2-***H***), 4.62 (2H, dt, *J* = 47.5, 5.0 Hz, 2‴-***H***$_2$), 3.97 (4H, s, 3″-***H***$_2$, 1″-***H***$_2$), 3.04 (2H, dt, *J* = 28.0, 5.0 Hz, 1‴-***H***$_2$); δ_C (176 MHz, $CDCl_3$) 169.7 (***C***=O), 141.1 (***C***-5″), 138.8 (***C***-1^{Ph}), 137.7 (***C***-1′), 136.5(0) (Ar***C***), 136.4(9) (Ar***C***), 133.6 (***C***-4′), 130.5 (***C***-2′), 129.3 (***C***-3^{Ph}), 129.1 (***C***-3′), 129.0 (***C***-2^{Ph}), 127.9 (***C***-4^{Ph}), 122.7 (***C***-7″), 118.7 (***C***-6″), 114.4 (***C***-4″), 83.4 (d, *J* = 167.5 Hz, ***C***-2‴), 59.7 (***C***-3″), 59.4 (***C***-2), 59.3 (***C***-1″), 55.6 (d, *J* = 19.5 Hz, ***C***-1‴); δ_F (376 MHz, $CDCl_3$) −220.22 – −220.69 (m, 2‴-***F***); *m/z* (LCMS, ESI$^+$) 409 (M(^{35}Cl)H$^+$), 411 (M(^{37}Cl)H$^+$); Accurate mass: Found MH$^+$, 409.1476: $C_{24}H_{23}N_2OF^{35}Cl$ requires *M*, 409.1483.

N-([Pyrrolidin-2′′-yl]methyl)-1-(4′-fluorophenyl)cyclopropane-1-carboxamide **S48**

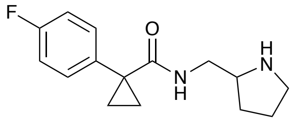

S48

Obtained in 50% yield as a yellow gum. ν_{max} (ATR) 3326 (br), 2958 (w), 2832 (w), 1658 (m), 1511 (s), 1408 (m), 1214 (m) cm^{-1}; δ_H (700 MHz, $CDCl_3$) 7.37 (2H, dd, *J* = 8.5, 5.5 Hz, 2′-***H***$_2$), 7.04 (2H, t, *J* = 8.5 Hz, 3′-***H***$_2$), 5.76 – 5.70 (1H, m, N***H***), 3.24 (1H, ddd, *J* = 13.0, 6.0, 4.5 Hz, NHC***H***H′), 3.16 (1H, qd, *J* = 7.0, 4.5 Hz, 2′′-***H***), 3.00 (1H, ddd, *J* = 13.0, 7.0, 5.5 Hz, NHCH***H′***), 2.81 (1H, ddd, *J* = 10.5, 7.5, 6.0 Hz, 5′′-***H***H′), 2.73 (1H, dt, *J* = 10.5, 6.5 Hz, 5′′-H***H′***), 1.81 – 1.73 (1H, m, 1′′-***H***), 1.80 – 1.72 (1H, m, 3′′-***H***H′), 1.68 – 1.60 (2H, m, 4′′-***H***$_2$), 1.61 – 1.56 (2H, m, 2-***H***$_2$H′$_2$), 1.26 (1H, ddt, *J* = 13.0, 8.5, 7.0 Hz, 3′′-H***H′***), 1.02 – 0.98 (2H, m, 2-H$_2$***H′***$_2$); δ_C (176 MHz, $CDCl_3$) 173.9 (***C***=O), 162.3 (d, *J* = 247.5 Hz, ***C***-4′), 135.9 (d, *J* = 3.5 Hz, ***C***-1′), 132.8 (d, *J* = 8.0 Hz, ***C***-2′), 116.0 (d, *J* = 21.5 Hz, ***C***-3′), 57.4 (***C***-2′′), 46.7 (***C***-5′′), 44.8 (NH***C***HH′), 29.9 (***C***-1), 29.1 (***C***-3′′), 26.0 (***C***-4′′), 15.8 (***C***-2), 15.7 (***C***-2); δ_F (376 MHz, $CDCl_3$) −114.22 – −114.31 (m, 4′-***F***); *m/z* (LCMS, ESI$^+$) 263 (MH$^+$); Accurate mass: Found MH$^+$, 263.1566: $C_{15}H_{20}N_2OF$ requires *M*, 263.1560.

N-([Pyrrolidin-2′-yl]methyl)-1-methyl-4-phenyl-piperidine-4-carboxamide **S49**

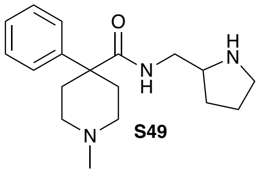

S49

Obtained in 50% yield as a yellow oil. ν_{max} (ATR) 3308 (br), 2946 (m), 2798 (w), 1640 (s), 1532 (s), 1450 (m), 1412 (w), 1290 (m) cm^{-1}; δ_H (700 MHz, $CDCl_3$) 7.40 – 7.37 (2H, m, 2^{Ph}-***H***$_2$), 7.34 (2H, t, *J* = 7.5 Hz, 3^{Ph}-***H***$_2$), 7.27 – 7.22 (1H, m, 4^{Ph}-***H***), 5.95 – 5.88 (1H, m, N***H***), 3.28 (1H, ddd, *J* = 13.0, 5.0, 4.5 Hz, NHC***H***H′), 3.14 (1H, qd, *J* = 7.0, 4.5 Hz, 2′-***H***), 2.99 (1H, ddd, *J* = 13.0, 7.0, 5.5 Hz, NHCH***H′***), 2.79 (1H, dt, *J* = 10.5, 7.0 Hz, 5′-***H***H′), 2.69 (1H, dt, *J* = 10.5, 6.5 Hz, 5′-H***H′***), 2.66 – 2.52 (2H, m, 2-***H***$_2$H′$_2$), 2.47 – 2.43 (2H, m, 3-***H***$_2$H′$_2$), 2.43 – 2.35 (2H, m, 2-H$_2$***H′***$_2$), 2.25 (3H, s, NC***H***$_3$), 2.14 – 2.06 (2H, m, 3-H$_2$***H′***$_2$), 1.71 (1H, dq, *J* = 12.5, 7.0 Hz, 3′-***H***H′), 1.57 (2H, dtd, *J* = 7.5, 7.0, 6.5 Hz, 4′-***H***$_2$), 1.17 (1H, dtd, *J* = 12.5, 7.5, 7.0 Hz, 3′-H***H′***); δ_C (176 MHz, $CDCl_3$) 174.9 (***C***=O), 143.6 (***C***-1^{Ph}) , 128.9 (***C***-3^{Ph}), 127.0 (***C***-4^{Ph}), 126.2 (***C***-2^{Ph}), 57.4 (***C***-2′), 53.0 (***C***-2), 48.7 (***C***-4), 46.7 (***C***-5′), 46.4 (N***C***H$_3$), 43.9 (NH***C***HH′), 34.2 (***C***-3), 28.9 (***C***-3′), 26.0 (***C***-4′); *m/z* (LCMS, ESI$^+$) 302 (MH$^+$); Accurate mass: Found MH$^+$, 302.2239: $C_{18}H_{28}N_3O$ requires *M*, 302.2232.

N-([Pyrrolidin-2′′-yl]methyl)-2-(pyridin-3′-yl)acetamide **S50**

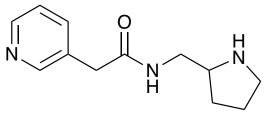

S50

Obtained in 30% yield as a yellow oil. ν_{max} (ATR) 3262 (br), 3034 (w), 2950 (w), 2870 (w), 1650 (s), 1558 (s), 1524 (s), 1478 (w), 1432 (s), 1406 (s), 1360 (m), 1192 (w) cm^{-1}; δ_H (700 MHz, $CDCl_3$) 8.51 – 8.48 (2H, m, 2′-***H***, 6′-***H***), 7.65 (1H, dt, *J* = 8.0, 2.0 Hz, 4′-***H***), 7.24 (1H, dd, *J* = 8.0, 5.0 Hz, 5′-***H***), 6.63 – 6.54 (1H, m, N***H***), 3.51 (2H, s, 2-***H***$_2$), 3.40 (1H, ddd, *J* = 13.5, 6.0, 4.0 Hz, NHC***H***H′), 3.29 – 3.22 (1H, m, 2′′-***H***), 3.03 (1H, ddd, *J* = 13.5, 8.0, 5.0 Hz, NHCH***H′***), 3.06 – 2.95 (1H, m, 1′′-***H***), 2.89 (1H, ddd, *J* = 10.5, 7.5, 6.0 Hz, 5′′-***H***H′), 2.84 (1H, dt, *J* = 10.5, 7.0 Hz, 5′′-H***H′***), 1.84 (1H, dtd, *J* = 13.0, 8.0, 5.5 Hz, 3′′-***H***H′), 1.77 – 1.70 (1H, m, 4′′-***H***H′), 1.70 – 1.62 (1H, m, 4′′-H***H′***), 1.33 (1H, ddt, *J* = 13.0, 8.5, 7.0 Hz, 3′′-H***H′***); δ_C (176 MHz, $CDCl_3$) 170.1 (***C***=O), 150.4 (***C***-2′), 148.6 (***C***-6′), 136.9 (***C***-4′), 131.1 (***C***-3′), 123.7 (***C***-5′), 57.7 (***C***-2′′), 46.5 (***C***-5′′), 43.7 (NH***C***HH′), 40.8 (***C***-2), 29.2 (***C***-3′′), 25.9 (***C***-4′′); *m/z* (LCMS, ESI$^+$) 220 (MH$^+$); Accurate mass: Found MH$^+$, 220.1453: $C_{12}H_{18}N_3O$ requires *M*, 220.1450.

N-([Pyrrolidin-2′′-yl]methyl)-2-methyl-2-(pyrazol-1′-yl)propionamide **S51**

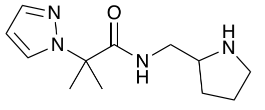

S51

Obtained in 45% yield as a yellow oil. ν_{max} (ATR) 3326 (br), 2938 (w), 2868 (w), 1655 (s), 1516 (br, s), 1394 (s), 1316 (w), 1250 (w), 1200 (w), 1156 (w), 1088 (w), 1058 (w) cm^{-1}; δ_H (700 MHz, $CDCl_3$) 7.63 (1H, d, *J* = 2.0 Hz, Ar***H***), 7.60 (1H, d, *J* = 2.0 Hz, Ar***H***), 6.46 – 6.41 (1H, m, N***H***), 6.32 (1H, t, *J* = 2.0 Hz, 4′-***H***), 3.23 (1H, ddd, *J* = 13.0, 6.0, 4.5 Hz, NHC***H***H′), 3.15 (1H, qd, *J* = 7.0, 4.5 Hz, 2′′-***H***), 3.01 (1H, ddd, *J* = 13.0, 7.0, 5.5 Hz, NHCH***H′***), 2.85 – 2.78 (2H, m, 5′′-***H***$_2$), 1.85 (3H, s, 2-(C***H***$_3$)(CH_3)), 1.84 (3H, s, 2-(CH_3)(C***H***$_3$)), 1.74 (1H, dddd, *J* = 12.5, 8.5, 7.5, 5.5 Hz, 3′′-***H***H′), 1.70 – 1.58 (2H, m, 4′′-***H***$_2$), 1.23 (1H, ddt, *J* = 12.5, 8.5, 7.0 Hz, 3′′-H***H′***); δ_C (176 MHz, $CDCl_3$) 173.4 (***C***=O), 140.4 (Ar***C***), 128.0 (Ar***C***), 106.2 (***C***-4′), 65.2 (***C***-2), 57.3 (***C***-2′′), 46.6 (***C***-5′′), 44.3 (NH***C***HH′), 29.0 (***C***-3′′), 26.1 (2-(***C***H$_3$)(CH_3)), 26.02 (2-(CH_3)(***C***H$_3$)), 25.80 (***C***-4′′); *m/z* (LCMS, ESI$^+$) 237 (MH$^+$); Accurate mass: Found MH$^+$, 237.1723: $C_{12}H_{21}N_4O$ requires *M*, 237.1715.

N-([Pyrrolidin-2′′-yl]methyl)-2-(morpholin-4′-yl)acetamide **S52**

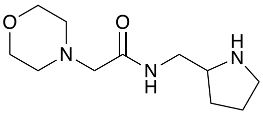

S52

Obtained in 56% yield as an orange oil. ν_{max} (ATR) 3340 (br), 2954 (w), 2864 (w), 2812 (w), 1654 (s), 1523 (s), 1404 (m), 1290 (w), 1114 (s), 1014 (w) cm^{-1}; δ_H (700 MHz, $CDCl_3$) 7.47 – 7.39 (1H, m, N***H***), 3.75 – 3.67 (4H, m, 2′-(***H***$_2$)$_2$), 3.40 – 3.33 (1H, m, NHC***H***H′), 3.29 – 3.23 (1H, m, 2′′-***H***), 3.13 – 3.07 (1H, m, NHCH***H′***), 3.02 – 2.97 (2H, m, 2-***H***$_2$), 2.93 – 2.87 (2H, m, 5′′-***H***$_2$), 2.56 – 2.47 (4H, m, 3′-(***H***$_2$)$_2$), 1.88 – 1.81 (1H, m, 3′′-***H***H′), 1.78 – 1.72 (1H, m, 4′′-***H***H′), 1.71 – 1.64 (1H, m, 4′′-H***H′***), 1.38 – 1.31 (1H, m, 3′′-H***H′***); δ_C (176 MHz, $CDCl_3$) 170.07 (***C***=O), 67.1 (***C***-2′), 62.2 (***C***-2), 57.9 (***C***-2′′), 54.0 (***C***-3′), 46.8 (***C***-5′′), 43.7 (NH***C***HH′), 29.3 (***C***-3′′), 26.0 (***C***-4′′); *m/z* (LCMS, ESI$^+$) 228 (MH$^+$); Accurate mass: Found MH$^+$, 228.1706: $C_{11}H_{22}N_3O_2$ requires *M*, 228.1712.

N-([Pyrrolidin-2′-yl]methyl)1-methylbenzimidazole-2-carboxamide **S53**

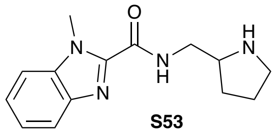

S53

Obtained in 36% yield as an orange oil. ν_{max} (ATR) 3326 (br), 2954 (w), 2886 (w), 1665 (s), 1536 (s), 1463 (s), 1395 (m), 1332 (m), 1264 (m), 1006 (w) cm^{-1}; δ_H (700 MHz, $CDCl_3$) 8.08 – 8.02 (1H, m, N***H***), 7.77 (1H, dt, *J* = 8.0, 1.0 Hz, 4-***H***), 7.43 (1H, dt, *J* = 8.0, 1.0 Hz, 7-***H***), 7.38 (1H, ddd, *J* = 8.0, 7.0, 1.0 Hz, 6-***H***), 7.33 (1H, ddd, *J* = 8.0, 7.0, 1.0 Hz, 5-***H***), 4.23 (3H, s, NC***H***$_3$), 3.56 (1H, ddd, *J* = 13.5, 6.5, 5.0 Hz, NHC***H***H′), 3.42 – 3.37 (1H, m, 2′-***H***), 3.31 (1H, ddd, *J* = 13.0, 7.5, 5.5 Hz, NHCH***H′***), 3.01 – 2.92 (2H, m, 5′-***H***$_2$), 1.93 (1H, dddd, *J* = 12.5, 9.0, 7.5, 5.5 Hz, 3′-***H***H′), 1.88 – 1.79 (1H, m, 4′-***H***H′), 1.76 – 1.69 (1H, m, 4′-H***H′***), 1.48 (1H, ddt, *J* = 13.0, 9.0, 6.5 Hz, 3′-H***H′***); δ_C (176 MHz, $CDCl_3$) 160.1 (***C***=O), 143.8 (***C***-2), 141.2 (***C***-8), 137.2 (***C***-9), 124.6 (***C***-6), 123.5 (***C***-5), 120.8 (***C***-4), 110.5 (***C***-7), 57.9 (***C***-2′), 46.7 (***C***-5′), 44.1 (NH***C***HH′), 32.2 (N***C***H$_3$), 29.5 (***C***-3′), 25.9 (***C***-4′); *m/z* (LCMS, ESI$^+$) 259 (MH$^+$); Accurate mass: Found MH$^+$, 259.1565: $C_{14}H_{19}N_4O$ requires *M*, 259.1559.

N-([Pyrrolidin-2′-yl]methyl)-2-(benzyloxy)benzamide **S54**

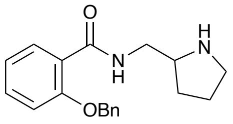

S54

Obtained in 53% yield as an off-white solid. m.p. 83 – 85 °C; ν_{max} (ATR) 3396 (br), 3058 (w), 2942 (w), 2864 (w), 1643 (s), 1598 (m), 1534 (m), 1482 (m), 1450 (m), 1290 (m), 1230 (m), 1166 (w), 1105 (w), 1006 (w) cm^{-1}; δ_H (600 MHz, $CDCl_3$) 8.21 (1H, dd, *J* = 8.0, 2.0 Hz, 6-***H***), 8.12 – 8.06 (1H, m, N***H***), 7.50 – 7.46 (2H, m, 2^{Ph}-***H***$_2$), 7.45 – 7.35 (4H, m, 4-***H***, 3^{Ph}-***H***$_2$, 4^{Ph}-***H***), 7.09 (1H, td, *J* = 8.0, 1.0 Hz, 5-***H***), 7.05 (1H, dd, *J* = 8.5, 1.0 Hz, 3-***H***), 5.17 (2H, s, OC***H***$_2$), 3.48 (1H, ddd, *J* = 13.0, 6.0, 4.5 Hz, NHC***H***H′), 3.19 (1H, ddd, *J* = 13.0, 7.5, 5.0 Hz, NHCH***H′***), 3.13 (1H, tdd, *J* = 7.5, 7.0, 4.5 Hz, 2′-***H***), 2.81 – 2.72 (2H, m, 5′-***H***$_2$), 1.75 – 1.65 (2H, m, 4′-***H***H′, 3′-***H***H′), 1.64 – 1.56 (1H, m, 4′-H***H′***), 1.30 – 1.22 (1H, m, 3′-H***H′***); δ_C (151 MHz, $CDCl_3$) 165.6 (***C***=O), 157.0 (***C***-2), 135.8 (***C***-1^{Ph}), 132.7 (***C***-4), 132.5 (***C***-6), 129.0 (***C***-3^{Ph}), 128.9 (***C***-4^{Ph}), 128.5 (***C***-2^{Ph}), 122.2 (***C***-1), 121.7 (***C***-5), 112.6 (***C***-3), 71.5 (O***C***H$_2$), 57.8 (***C***-2′), 46.6 (***C***-5′), 44.8 (NH***C***H$_2$), 29.3 (***C***-3′), 25.8 (***C***-4′); *m/z* (LCMS, ESI$^+$) 311 (MH^+); Accurate mass: Found MH^+, 311.1757: $C_{19}H_{23}N_2O_2$ requires *M*, 311.1760.

N-([Pyrrolidin-2′′-yl]methyl)-3-(1′-methylpyrazol-5′-yl)benzamide **S56**

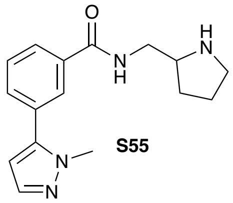

S55

Obtained in 60% yield as an orange oil. ν_{max} (ATR) 3278 (br), 2942 (w), 2876 (w), 1641 (s), 1537 (s), 1476 (w), 1404 (w), 1296 (w), 1188 (w), 1104 (w) cm^{-1}; δ_H (700 MHz, $CDCl_3$) 7.88 (1H, td, *J* = 1.5, 0.5 Hz, 2-***H***), 7.80 (1H, dt, *J* = 7.5, 1.5 Hz, 6-***H***), 7.52 (1H, dt, *J* = 7.5, 1.5 Hz, 4-***H***), 7.52 (1H, d, *J* = 2.0 Hz, 3′-***H***), 7.49 (1H, td, *J* = 7.5, 0.5 Hz, 5-***H***), 7.14 – 7.07 (1H, m, N***H***), 6.34 (1H, d, *J* = 2.0 Hz, 4′-***H***), 3.89 (3H, s, NC***H***$_3$), 3.63 (1H, ddd, *J* = 13.5, 6.0, 4.0 Hz, NHC***H***H′), 3.43 (1H, dddd, *J* = 8.0, 7.5, 7.0, 4.0 Hz, 2′′-***H***), 3.23 (1H, ddd, *J* = 13.5, 8.0, 5.0 Hz, NHCH***H′***), 2.96 (1H, ddd, *J* = 11.0, 8.0, 6.0 Hz, 5′′-***H***H′), 2.92 (1H, dt, *J* = 11.0, 7.0 Hz, 5′′-H***H′***), 1.94 (1H, dddd, *J* = 13.0, 9.0, 7.5, 5.5 Hz, 3′′-***H***H′), 1.86 – 1.79 (1H, m, 4′′-***H***H′), 1.77 – 1.70 (1H, m, 4′′-H***H′***), 1.47 (1H, ddt, *J* = 13.0, 8.5, 7.0 Hz, 3′′-H***H′***); δ_C (176 MHz, $CDCl_3$) 167.0 (***C***=O), 142.8 (***C***-5′), 138.8 (***C***-3′), 135.4 (***C***-1), 131.6 (***C***-4), 131.4 (***C***-3), 129.0 (***C***-5), 127.7 (***C***-2), 126.9 (***C***-6), 106.5 (***C***-4′), 57.8 (***C***-2′′), 46.6 (***C***-5′′), 44.0 (NH***C***HH′), 37.7 (N***C***H$_3$), 29.3 (***C***-3′′), 26.1 (***C***-4′′); *m/z* (LCMS, ESI$^+$) 285 (MH^+); Accurate mass: Found MH^+, 285.1712: $C_{16}H_{21}N_4O$ requires *M*, 285.1715.

N-([Pyrrolidin-2’-yl]methyl)cyclohexanecarboxamide **S56**

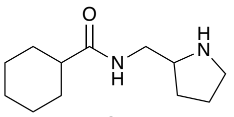

S56

Obtained in 52% yield as a yellow oil pale yellow solid. m.p. 107 – 110 °C; ν_{max} (ATR) 3282 (br), 2927 (s), 2860 (m), 1637 (s), 1542 (s), 1456 (m), 1404 (m), 1256 (w) cm^{-1}; δ_H (700 MHz, $CDCl_3$) 6.12 – 6.03 (1H, m, N***H***), 3.40 – 3.34 (1H, m, NHC***H***H’), 3.24 (1H, dtd, *J* = 8.0, 7.0, 4.5 Hz, 2’-***H***), 3.02 (1H, ddd, *J* = 13.0, 8.0, 5.0 Hz, NHCH***H’***), 2.93 – 2.86 (2H, m, 5’-$\boldsymbol{H_2}$), 2.06 (1H, tt, *J* = 12.0, 3.5 Hz, 1^{Cy}-***H***), 1.88 – 1.79 (3H, m, 2^{Cy}-$\boldsymbol{H_2}$H’$_2$, 3’-***H***H’), 1.79 – 1.71 (3H, m, 3^{Cy}-$\boldsymbol{H_2}$H’$_2$, 4’-***H***H’), 1.71 – 1.62 (2H, m, 4^{Cy}-***H***H’, 4’-H***H’***), 1.41 (2H, tdd, *J* = 12.5, 12.0, 3.0 Hz, 2^{Cy}-H$_2$$\boldsymbol{H'_2}$), 1.35 (1H, ddt, *J* = 13.0, 8.5, 7.0 Hz, 3’-H***H’***), 1.29 – 1.15 (3H, m, 3^{Cy}-H$_2$$\boldsymbol{H'_2}$, 4^{Cy}-H***H’***); δ_C (176 MHz, $CDCl_3$, mixture of rotamers) 176.4 (***C***=O), 57.9 (***C***-2’), 46.7 (***C***-5’), 45.7 (***C***-1^{Cy}), 43.5 (NH***C***HH’), 29.9(0) (***C***-2^{Cy}), 29.8(5) (***C***-2^{Cy}), 29.2 (***C***-3’), 26.0 (***C***-4’), 25.9(1) (***C***-4^{Cy}), 25.9(0) (***C***-3^{Cy}); *m/z* (LCMS, ESI$^+$) 211 (MH$^+$); Accurate mass: Found MH$^+$, 211.1808: $C_{12}H_{23}N_2O$ requires *M*, 211.1810. All data agrees with commercial sample.

*N-([Pyrrolidin-2’-yl]methyl)-2-methylpropionamide **S57***

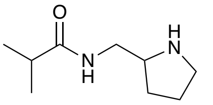

S57

Obtained in 59% yield as a yellow oil. ν_{max} (ATR) 3300 (br), 3058 (w), 2958 (w), 2928 (w), 2872 (w), 1649 (s), 1542 (br, s), 1460 (w), 1394 (s), 1244 (w), 1170 (w), 1110 (w) cm^{-1}; δ_H (600 MHz, $CDCl_3$) 6.08 – 5.98 (1H, m, NH), 3.38 (1H, ddd, J = 13.0, 6.0, 4.5 Hz, NHCHH’), 3.29 – 3.21 (1H, m, 2’-H), 3.02 (1H, ddd, J = 13.0, 8.0, 5.0 Hz, NHCHH’), 2.94 – 2.85 (2H, m, 5’-H$_2$), 2.35 (1H, hept, J = 7.0 Hz, 2-H), 1.89 – 1.80 (1H, m, 3’-HH’), 1.80 – 1.72 (1H, m, 4’-HH’), 1.72 – 1.64 (1H, m, 4’-HH’), 1.41 – 1.31 (1H, m, 3’-HH’), 1.14 (6H, d, J = 7.0 Hz, 2-(CH_3)(CH_3)); δ_C (151 MHz, $CDCl_3$) 177.2 (C=O), 57.9 (C-2’), 46.7 (C-5’), 43.6 (NHCHH’), 35.8 (C-2), 29.2 (C-3’), 26.1 (C-4’), 19.8(4) (2-(CH_3)(CH_3)), 19.7(9) (2-(CH_3)(CH_3)); m/z (LCMS, ESI$^+$) 171 (MH$^+$); Accurate mass: Found MH$^+$, 171.1501: $C_9H_{19}N_2O$ requires M, 171.1497. All data agrees with commercial sample.

N-([Pyrrolidin-2-yl]methyl)benzamide **S58**

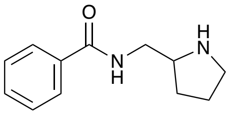

S58

Obtained in 17% yield as a yellow oil. ν_{max} (ATR) 3296 (br), 3054 (w), 2928 (w), 2872 (w), 1636 (s), 1538 (s), 1486 (m), 1404 (m), 1356 (m), 1300 (w) cm^{-1}; δ_H (700 MHz, $CDCl_3$) 7.82 – 7.79 (2H, m, 2^{Ph}-$\boldsymbol{H_2}$), 7.50 –

7.45 (1H, m, 4^{Ph}-***H***), 7.44 – 7.39 (2H, m, 3^{Ph}-***H***$_2$), 7.00 – 6.94 (1H, m, N***H***), 3.61 (1H, ddd, *J* = 13.5, 6.0, 4.0 Hz, NHC***H***H'), 3.41 (1H, dddd, *J* = 8.0, 7.5, 7.0, 4.0 Hz, 2-***H***), 3.23 (1H, ddd, *J* = 13.5, 8.0, 5.0 Hz, NHCH***H'***), 2.96 – 2.89 (2H, m, 5-***H***$_2$), 1.91 (1H, dddd, *J* = 13.0, 9.0, 7.5, 5.5 Hz, 3-***H***H'), 1.84 – 1.78 (1H, m, 4-***H***H'), 1.75 – 1.68 (1H, m, 4-H***H'***), 1.46 (1H, ddt, *J* = 13.0, 8.5, 7.0 Hz, 3-H***H'***); δ_C (176 MHz, $CDCl_3$) 167.7 (***C***=O), 134.8 (***C***-1^{Ph}), 131.5 (***C***-4^{Ph}), 128.6 (***C***-3^{Ph}), 127.1 (***C***-2^{Ph}), 57.9 (***C***-2), 46.6 (***C***-5), 44.0 (NH***C***HH'), 29.3 (***C***-3), 26.1 (***C***-4); *m/z* (LCMS, ESI^+) 205 (MH^+); Accurate mass: Found MH^+, 205.1347: $C_{12}H_{17}N_2O$ requires *M*, 205.1341. All data agrees with commercial sample.

(2R)-2-{2-[(4-chlorophenyl)(phenyl)methoxy]ethyl}-1-methylpyrrolidine **10 (General procedure C)**

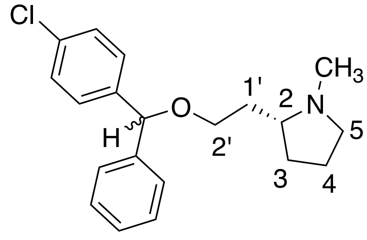

To a solution of *(2R) benzyl 2-(2'-hydroxyethyl)pyrrolidine-1-carboxylate* **(*R*)-6d** (1 equiv.) and diarylcarbinol **5a** (1 equiv.) in DCE (0.2M) was added AuCl (0.1 equiv.). The reaction mixture was heated at 80 °C for 48 h. The solvent was then removed under reduced pressure to afford the crude product which was purified by chromatography. This was then dissolved in THF (0.1 M) and $LiAlH_4$ (2.4 M solution in THF, 2.5 equiv.) slowly added. After stirring at rt, rt for 4 h, the reaction mixture was cooled in an ice bath and any reactive salts were quenched according to Fieser's method. The crude product was purified by reversed phase column chromatography (5 → 100% MeCN in H_2O) to afford the title amine in 59% yield as a yellow oil identical in all respect to that described above.

(2R)-2-[2-(diphenylmethoxy)ethyl]-1-methylpyrrolidine **28**

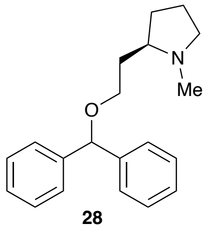

28

To a 0 ˚C solution of Benzyl (*2R)-2-{2'-[(4''-bromophenyl)(phenyl)methoxy]ethyl}pyrrolidine-1-carboxylate* (256 mg, 0.52 mmol) in THF (6 mL) was slowly added $LiAlH_4$ (2.4M solution in THF, 0.54 mL, 1.30 mmol), before being heated under reflux for 16h. The reaction mixture was then cooled in an ice bath and any reactive salts were quenched according to Fieser's method. The crude product was purified by reversed

phase column chromatography (5% → 100% MeCN in H_2O with 0.1% formic acid) to afford title compound (102 mg, 52%) as a yellow oil. ν_{max} (ATR) 3028 (w), 2944 (w), 2869 (w), 2778 (w), 1697 (w), 1590 (w), 1485 (m), 1453 (m), 1346 (w), 1184 (w), 1091 (s), 1071 (s), 1010 (s) cm^{-1}; δ_H (600 MHz, $CDCl_3$) 7.34 - 7.29 (8H, m, Ar***H***), 7.27 – 7.23 (2H, m, Ar***H***), 5.32 (1H, m, Ar_2C***H***), 3.69 – 3.60 (2H, m, 2'-***H***H', 5-***H***H'), 3.53 – 3.46 (1H, m, 2'-H***H'***), 3.13 - 2.93 (1H, m, 2-***H***), 2.68 – 2.62 (4H, m, 5-H***H'***, N***C***H_3), 2.36 – 2.24 (1H, m, 1'-***H***H'), 2.18 – 1.99 (3H, m, 1'-H***H'***, 3-***H***H', 4-***H***H'), 1.96 – 1.76 (2H, m, 3-H***H'***, 4-H***H'***); δ_C (151 MHz, $CDCl_3$) 142.1 (***C***-$1^{Ph}/1'^{Ph}$), 142.0 (***C***-$1^{Ph}/1'^{Ph}$), 128.6 (***C***-$4^{Ph}/4'^{Ph}$), 127.8 (***C***-$3^{Ph}/3'^{Ph}$), 127.7 (***C***-$3^{Ph}/3'^{Ph}$), 127.0(3) (***C***-$2^{Ph}/2'^{Ph}$), 127.9(5) (***C***-$2^{Ph}/2'^{Ph}$), 84.1 (Ar_2***C***H), 66.3 (***C***-2), 65.9 (***C***-2'), 56.4 (***C***-5), 39.6 (N***C***H_3), 31.4 (***C***-1'), 30.1 (***C***-3), 21.8 (***C***-4); *m/z* (LC-MS, ESI^+) 296 (MH^+).

2-(2'-[{4'''-Bromophenyl}{phenyl}methoxy]ethyl)-1-methylpyrrolidine **29**

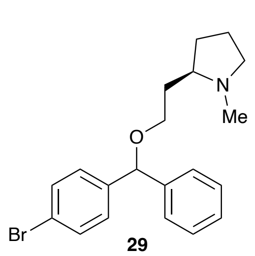

29

Following the same procedure as described for **7**, diarylcarbinol **5c** (226 mg, 0.86 mmol) and N-methyl homoprolinol **(*S*)-6b** (101 mg, 0.78 mmol) were combined to afford, following reverse-phase column chromatography (5% → 100% MeCN in H_2O with 0.1% formic acid), the *title compound* (89 mg, 30%) as a colourless oil. R_f 0.29 (10% MeOH in $CHCl_3$ with 1% NEt_3);ν_{max} (ATR) 3033 (w), 3945 (w), 2870 (w), 2775 (w), 1486 (m), 1453 (m), 1366 (s), 1218 (s), 1092 (s), 1010 (s) cm^{-1}; δ_H (700 MHz, $CDCl_3$, mixture of diastereomers) 7.43 (2H, dd, *J* = 8.5, 2.5 Hz, 3'''-***H***$_2$), 7.35 – 7.28 (4H, m, 2^{Ph}-***H***$_2$, 3^{Ph}-***H***$_2$), 7.26 – 7.23 (1H, m, 4^{Ph}-***H***), 7.21 (2H, dd, *J* = 8.5, 2.5 Hz, 2'''-***H***$_2$), 5.27 (1H, s, 1''-***H***), 3.55 – 3.49 (1H, m, 2'-***H***H'), 3.49 – 3.44 (1H, m, 2'-H***H'***), 3.14 (1H, apparent t, *J* = 9.0 Hz, 5-***H***H'), 2.36 (3H, app d, *J* = 5.0, NC***H***$_3$ isomer 1, NC***H***$_3$ isomer 2), 2.32 – 2.25 (1H, m, 2-***H***), 2.20 (1H, apparent q, *J* = 9.0 Hz, 5-H***H'***), 2.14 – 2.07 (1H, m, 1'-***H***H'), 1.97 – 1.88 (1H, m, 3-***H***H'), 1.84 – 1.74 (1H, m, 4-***H***H'), 1.74 – 1.66 (1H, m, 4-H***H'***), 1.65 – 1.57 (1H, m, 1'-H***H'***), 1.55 – 1.46 (1H, m, 3-H***H'***); δ_C (176 MHz, $CDCl_3$, mixture of diastereomers) 142.0(0) (***C***-1^{Ph} isomer 1), 141.9(6) (***C***-1^{Ph} isomer 2), 141.7(2) (***C***-1''' isomer 1), 141.7(0) (***C***-1''' isomer 2), 131.6(0) (***C***-3''' isomer 1), 131.5(9) (***C***-3''' isomer 2), 128.7(2) (***C***-2''' isomer 1), 128.6(8) (***C***-2''' isomer 2), 128.6 (***C***-3^{Ph}), 127.8(0) (***C***-4^{Ph} isomer 1), 127.7(7) (***C***-4^{Ph} isomer 2), 127.0(3) (***C***-2^{Ph} isomer 1), 126.9(7) (***C***-2^{Ph} isomer 2), 121.4(1) (***C***-4''' isomer 1), 121.3(8) (***C***-4''' isomer 2), 83.3(0) (***C***-1'' isomer 1), 83.2(7) (***C***-1'' isomer 2), 67.1(3) (***C***-2' isomer 1), 67.0(9) (***C***-2' isomer 2), 64.2 (***C***-2), 57.1 (***C***-5), 40.5 (N***C***H_3), 33.8 (***C***-1'), 31.0 (***C***-3 isomer 1), 30.9 (***C***-3 isomer 2), 22.0(4) (***C***-4 isomer 1), 22.0(3) (***C***-4 isomer 2); *m/z* (LCMS, ESI^+) 374 ($M(^{79}Br)H^+$), 376 ($M(^{81}Br)H^+$); Accurate mass: Found MH^+, 374.1134: $C_{20}H_{25}NO^{79}Br$ requires *M*, 374.1120.

(2R)-2-{2-[(4-Methoxyphenyl)(phenyl)methoxy]ethyl}-1-methylpyrrolidine **30**

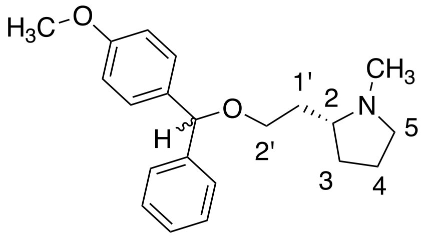

Obtained following general procedure C in 20% overall yield as a colourless oil. ν_{max} (ATR) 2961 (w), 1611(w), 1510 (m), 1453 (w), 1247 (m), 1172 (w), 1090 (m), 1031 (m) cm^{-1}. δ_H (700 MHz, $CDCl_3$) 7.32 - 7.12 (7H, m, Ar***H***), 6.83 – 6.77 (2H, m, Ar***H***), 5.23 (1H, s, Ar_2C***H***), 3.72 (3H, s, OC***H₃***), 3.55 – 3.49 (1H, m, 2’-***H***H’), 3.48 – 3.38 (2H, m, 2’-H***H’***, 5-***H***H’), 2.77 – 2.69 (1H, m, 2-***H***), 2.50 (3H, s, NC***H₃***), 2.47 – 2.40 (1H, m, 5-H***H’***), 2.21 – 2.12 (1H, m, 1’-***H***H’), 2.05 – 1.96 (1H, m, 3-***H***H’), 1.95 – 1.85 (1H, m, 4-***H***H’), 1.82 – 1.71 (2H, m, 1’-H***H’***, 4-H***H’***), 1.68 - 1.59 (1H, m, 3-H***H’***). δ_C (176 MHz, $CDCl_3$, mixture of diastereomers) 159.0(4) (Ar***C***), 159.0(1) (Ar***C***), 142.5 (Ar***C***), 142.4 (Ar***C***), 134.3(4) (Ar***C***), 134.2(9) (Ar***C***), 128.4 (Ar***C***), 128.3 (Ar***C***), 128.2 (Ar***C***), 127.4(4) (Ar***C***), 127.3(9) (Ar***C***), 126.8 (Ar***C***), 126.7 (Ar***C***), 113.8 (Ar***C***), 83.5 (Ar_2***C***), 83.4 (Ar_2***C***), 66.1(0) (***C***-2’), 66.0(6) (***C***-2’), 65.0 (***C***-2), 56.0 (***C***-5), 55.3 (O***C***H₃), 39.5 (N***C***H₃), 31.8(4) (***C***-1’), 31.8(1) (***C***-1’), 30.2(3) (***C***-3), 30.2(0) (***C***-3), 21.7 (***C***-4). *m/z* (LC-MS, ESI$^+$) 326 (MH$^+$). Accurate mass: Found MH$^+$, 326.2129: $C_{21}H_{28}NO_2$ requires *M,* 326.2120.

(2R)-2-{2-[(3-methoxyphenyl)(phenyl)methoxy]ethyl}-1-methylpyrrolidine **31**

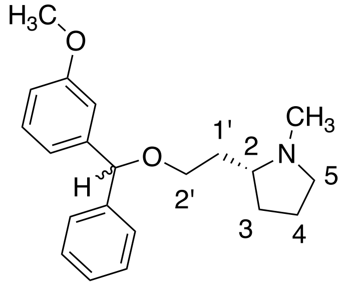

Obtained following general procedure C in 12% overall yield as a colourless oil. ν_{max} (ATR) 2946 (m), 1599 (m), 1488 (m), 1454 (m), 1265 (m), 1154 (w), 1095 (m), 1049 (m) cm^{-1}. δ_H (700 MHz, $CDCl_3$) 7.35 - 7.29 (4H, m, Ar***H***), 7.25 – 7.21 (2H, m, Ar***H***), 6.93 – 6.90 (2H, m, Ar***H***), 6.80 – 6.76 (1H, m, Ar***H***), 5.29 (1H, s, Ar_2C***H***), 3.78 (3H, s, OC***H₃***), 3.57 – 3.51 (1H, m, 2’-***H***H’), 3.51 – 3.45 (1H, m, 2’-H***H’***), 3.15 – 3.09 (1H, m, 5-***H***H’), 2.35 (3H, s, NC***H₃***), 2.32 – 2.24 (1H, m, 2-***H***), 2.22 – 2.16 (1H, m, 5-H***H’***), 2.15 – 2.09 (1H, m, 1’-***H***H’), 1.98 – 1.90 (1H, m, 3-***H***H’), 1.84 – 1.75 (1H, m, 4-***H***H’), 1.73 - 1.65 (1H, m, 4-H***H’***), 1.64 – 1.56 (1H, m, 1’-H***H’***), 1.55 – 1.46 (1H, m, 3-H***H’***). δ_C (176 MHz, $CDCl_3$, mixture of diastereomers) 159.8 (Ar***C***), 144.2(2) (Ar***C***), 144.2(0) (Ar***C***), 142.4(7) (Ar***C***), 142.4(3) (Ar***C***), 129.5 (Ar***C***), 128.5 (Ar***C***), 127.6 (Ar***C***), 127.5 (Ar***C***), 127.0(3) (Ar***C***), 126.9(8) (Ar***C***), 119.5(2) (Ar***C***), 119.4(7) (Ar***C***), 112.8(0) (Ar**C**), 112.7(6) (Ar**C**), 112.6(9) (Ar***C***), 112.6(5) (Ar***C***),

83.8 (Ar_2***C***H), 67.1 (***C***-2'), 64.2 (***C***-2), 57.2 (***C***-5), 55.3 (O***C***H_3), 40.5 (N***C***H_3), 33.9 (***C***-1'), 31.0 (***C***-3), 22.0 (***C***-4). *m/z* (LC-MS, ESI^+) 326 (MH^+). Accurate mass: Found MH^+, 326.2122: $C_{21}H_{28}NO_2$ requires *M,* 326.2120.

(2R)-2-{2-[(4-fluorophenyl)(phenyl)methoxy]ethyl}-1-methylpyrrolidine **32**

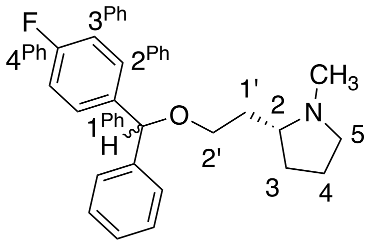

Obtained following general procedure C in 34% overall yield as a yellow oil. ν_{max} (ATR) 2942, 2870, 2777, 1604, 1508, 1454, 1348, 1294, 1221, 1184, 1156, 1090, 1029, 1016. δ_H (700 MHz, $CDCl_3$, mixture of diastereomers) 7.35 – 7.23 (7H, m, Ar***H***), 7.02 – 6.97 (2H, m, 3^{Ph}-***H_2***), 5.31 (1H, s, Ar_2C***H***), 3.55 – 3.49 (1H, m, 2'-***H***H'), 3.49 – 3.43 (1H, m, 2'-H***H'***), 3.14 – 3.08 (1H, m, 5-***H***H'), 2.34 (1.5H, s, NC***H_3***), 2.34 (1.5H, s, NC***H_3***), 2.29 – 2.21 (1H, m, 2-***H***), 2.21 – 2.15 (1H, m, 5-***H***H'), 2.14 – 2.06 (1H, m, 1'-***H***H'), 1.96 – 1.88 (1H, m, 3-***H***H'), 1.83 – 1.73 (1H, m, 4-***H***H'), 1.72 – 1.64 (1H, m, 4-H***H'***), 1.63 – 1.55 (1H, m, 1'-H***H'***), 1.53 – 1.44 (1H, m, 3-H***H'***). δ_C (176 MHz, $CDCl_3$, mixture of diastereomers) 162.2(1) (***C***-4^{Ph}, d, *J* = 245.3), 162.1(9) (***C***-4^{Ph}, d, *J* = 245.3 Hz), 142.4 (Ar***C***), 142.3 (Ar***C***), 138.4(5) (***C***-1^{Ph}, d, *J* = 3.0 Hz), 138.4(2) **(*C***-1^{Ph}, d, *J* = 3.0 Hz), 128.7(0) (Ar***C***), 128.6(5) (Ar***C***), 128.5(9) (Ar***C***), 128.5(5) (Ar***C***), 127.7 (Ar***C***), 127.6 (Ar***C***), 127.0 (***C***-2^{Ph}, d, *J* = 9.0 Hz), 115.3(0) (***C***-3^{Ph}, d, *J* = 21.4 Hz), 115.2(9) (***C***-3^{Ph}, d, *J* = 21.4 Hz), 83.2 (Ar_2***C***H), 66.1(0) (***C***-2'), 66.0(8) (***C***-2'), 64.1(2) (***C***-2), 64.1(1) (***C***-2), 57.2 (***C***-5), 40.5 (***C***H_3), 33.9 (***C***-1'), 31.0 (***C***-3), 30.9 (***C***-3), 22.1 (***C***-4). δ_F (376 MHz, $CDCl_3$) -115.3(2) (s, 4-***F***, isomer 1), -115.3(8) (s, 4^{Ph}-***F***, isomer 2). *m/z* (LC-MS, ESI^+) 314 (MH^+). Accurate mass: Found (MH^+), 314.1909: $C_{20}H_{25}NOF$ requires *M*, 314.1920.

(2R)-2-{2-[bis(4-fluorophenyl)methoxy]ethyl}-1-methylpyrrolidine **33**

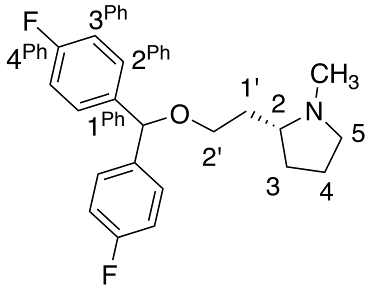

Obtained following general procedure C in 49% yield as a colourless oil. ν_{max} (ATR) 2946 (w), 2871 (w), 1603 (w), 1506 (s), 1220 (s), 1154 (w), 1086 (w). δ_H (700 MHz, $CDCl_3$) 7.28 - 7.24 (4H, m, 2^{Ph}-***H***), 7.02 - 6.98 (4H, m, 3^{Ph}-***H***), 5.28 (1H, s, Ar_2C***H***), 3.53 - 3.49 (1H, m, 2'-***H***H'), 3.46 - 3.42 (1H, m, 2'-H***H'***), 3.21 - 3.15 (1H, m, 5-***H***H'), 2.38 (3H, s, C***H_3***), 2.37 - 2.32 (1H, m, 2-***H***), 2.29 - 2.22 (1H, m, 5-H***H'***), 2.14 - 2.09 (1H, m, 1-***H***H'), 1.97 - 1.90 (1H, m, 4-***H***H'), 1.87 - 1.78 (1H, m, 3-***H***H'), 1.75 - 1.62 (2H, m, 3-H***H'***, 1'-H***H'***), 1.58 - 1.49 (1H,

m, 4-H***H'***). δ_C (176 MHz, $CDCl_3$) 162.2(6) (***C***-4^{Ph}, d, *J* = 245.5 Hz), 162.2(4) (***C***-4^{Ph}, d, *J* = 245.5 Hz), 138.0(9) (***C***-1^{Ph}, d, *J* = 3.0), 138.0(6) (***C***-1^{Ph}, d, *J* = 3.0), 128.6(2) (***C***-2^{Ph}, d, *J* = 8.0 Hz), 128.5(6) (***C***-2^{Ph}, d, *J* = 8.0 Hz), 115.4(1) (***C***-3^{Ph}, d, *J* = 21.5), 115.3(9) (***C***-3^{Ph}, d, *J* = 21.5), 82.5 (Ar_2***C***H), 66.9 (***C***-2'), 64.4 (***C***-2), 57.0 (***C***-5), 40.4 (***C***H_3), 33.4 (***C***-1'), 30.8 (***C***-4), 22.0 (***C***-3). δ_F (376 MHz, $CDCl_3$) -115.01- -115.11 (m, 4^{Ph}-***F***). *m/z* (LC-MS, ESI$^+$) 332 (MH^+). Accurate mass: Found (MH^+), 332.1828: $C_{20}H_{24}F_2NO$ requires *M*, 332.1826.

General procedure D: Alkylation of diarylcarbinols 5 using NaH

Diarylcarbinol **5** (1 equiv.) and NaH (60%, 3 equiv.) were dissolved in anhydrous toluene (0.2M) and heated under reflux for 3 h under nitrogen. The solution was cooled to rt and chloroethylpyrrolidine (1 equiv.) in toluene (0.4M) was added. The reaction mixture was then reacted under reflux overnight. The reaction mixture was cooled, quenched with H_2O and diluted with EtOAc. The aqueous layer was separated and extracted with EtOAc. The combined organic layers were then dried over Na_2SO_4, filtered, and then concentrated *in vacuo*. The crude product was purified by flash column chromatography (0 → 100% EtOAc in hexanes with 1% NEt_3) to afford the title compounds.

General procedure E: Alkylation of diarylcarbinols 5 using KO^{t}Bu

Diarylcarbinol **(*S*)-5a** (1 equiv.) was dissolved in anhydrous DMF (0.3M) in an oven dried sealed tube and the solution was degassed with nitrogen. Then ^{t}BuOK (2.2 equiv.), chloroethylpyrrolidine **(*R*)-3** (1.2 equiv.) and tetra-*n*-butylammonium iodide (0.2 equiv.) were added to it and the mixture was degassed again and stirred at 50 ºC overnight. The reaction mixture was cooled, quenched with H_2O and diluted with EtOAc. The aqueous layer was separated and extracted with EtOAc. The combined organic layers were then dried over Na_2SO_4, filtered, and then concentrated in vacuo. The crude product was purified by flash column chromatography (0 → 100% EtOAc in hexanes with 1% NEt_3) to afford the title compounds.

(2R)-2-{2'-[(R)-(4"-chlorophenyl)(phenyl)methoxy]ethyl}-1-methylpyrrolidine **(*R*, *R*)-35**

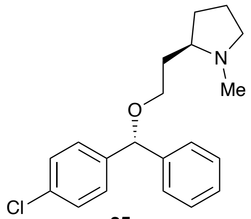

35

General procedure D was followed in the reaction of (*R*)-4-chlorophenyl(phenyl)methanol **(*R*)-5a** (120 mg, 0.43 mmol) and (2*R*)-2-(2-chloroethyl)-1-methylpyrrolidine **(*R*)-3** (63mg, 0.43 mmol). The crude product was purified by column chromatography (0 → 100% EtOAc in hexanes with 1% NEt_3) to afford title compound **(*R*, *R*)-35** as a colourless oil (53 mg, 38%) and by-product **(*S*, *R*)-36** (6 mg, 4%) as a colourless oil. R_f = 0.32 (80% EtOAc in hexanes with 1% NEt_3). ν_{max} (ATR) 2943 (w), 2776 (w), 1489 (w), 1453 (w), 1088

(m), 1015 (m). δ_H (700 MHz, $CDCl_3$) 7.32 – 7.29 (4H, m, 2^{Ph}-***H***, 3^{Ph}-***H***), 7.29 – 7.22 (5H, m, 2'^{Ph}-***H***, 3'^{Ph}-***H***, 4'^{Ph}-***H***), 5.28 (1H, s, Ar_2C***H***), 3.53 – 3.48 (1H, m, 2'-***H***H'), 3.47 - 3.42 (1H, m, 2'-H***H'***), 3.08 – 3.01 (1H, m, 5-***H***H'), 2.30 (3H, s, C***H₃***), 2.19 – 2.04 (3H, m, 1'-***H***H', 2-***H***, 5-H***H'***), 1.93 – 1.86 (1H, m, 3-***H***H'), 1.79 – 1.70 (1H, m, 4-***H***H'), 1.69 - 1.61 (1H, m, 4-H***H'***), 1.58 – 1.50 (1H, m, 1'-H***H'***), 1.49 -1.40 (1H, m, 3-H***H'***). δ_C (151 MHz, $CDCl_3$) 142.1 (Ar***C***), 141.2 (Ar***C***), 133.2 (Ar***C***), 128.6(0) (Ar***C***), 128.5(6) (Ar***C***), 128.4 (Ar***C***), 127.7 (Ar***C***), 127.0 (Ar***C***), 83.2 (Ar_2C***H***), 67.3 (***C***-2'), 63.9 (***C***-2), 57.3 (***C***-5), 40.6 (***C***H_3), 34.1 (***C***-1'), 31.0 (***C***-3), 22.1 (***C***-4). *m/z* (LC-MS, ESI$^+$) 330 (M(^{35}Cl)H$^+$), 332 (M(^{37}Cl)H$^+$). Accurate mass: Found (MH$^+$), 330.1608: $C_{20}H_{25}NO^{35}Cl$ requires *M*, 330.1625.

(4S)-4-[(R)-(4'-Chlorophenyl)(phenyl)methoxy]-1-methylazepane **36**

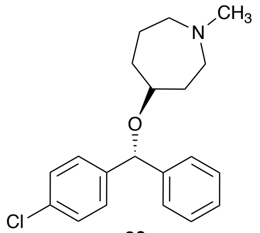

36

R_f = 0.27 (80% EtOAc in hexanes with 1% NEt_3). ν_{max} (ATR) 2936 (w), 1489 (w). δ_H (700 MHz, $CDCl_3$) 7.32 – 7.22 (9H, m, Ar***H***), 5.40 (1H, s, Ar_2C***H***), 3.67 – 3.61 (1H, m, 4-***H***), 2.81 - 2.72 (1H, m, 2-***H***H'), 2.69 – 2.63 (1H, m, 7-***H***H'), 2.62 – 2.56 (1H, m, 7-H***H'***), 2.54 – 2.47 (1H, m, 2-H***H'***), 2.36 (3H, s, C***H₃***), 2.07 – 2.00 (1H, m, 3-***H***H'), 1.98 – 1.89 (2H, m, 3-H***H'***, 5-***H***H'), 1.85 – 1.77 (2H, m, 5-H***H'***, 6-***H***H'), 1.60 – 1.52 (1H, m, 6-H***H'***). δ_C (176 MHz, $CDCl_3$) 142.5 (Ar***C***), 141.6 (Ar***C***), 133.2 (Ar***C***), 128.6(3) (Ar***C***), 128.5(8) (Ar***C***), 128.5 (Ar***C***), 127.7 (Ar***C***), 127.1 (Ar***C***), 79.9 (Ar_2CH), 75.6 (***C***-4), 58.9 (***C***-7), 52.7 (***C***-2), 47.0 (***C***H_3), 33.1 (***C***-3), 32.9 (***C***-5), 23.0 (***C***-6). *m/z* (LC-MS, ESI$^+$) 330 (M(^{35}Cl)H$^+$), 332 (M(^{37}Cl)H$^+$). Accurate mass: Found (MH$^+$), 330.1620: $C_{20}H_{25}NO^{35}Cl$ requires *M,* 330.1625.

(2R) 2-(2'-[(S)-(4"-Chlorophenyl)(phenyl)methoxy]ethyl)-1-methylpyrrolidine **37**

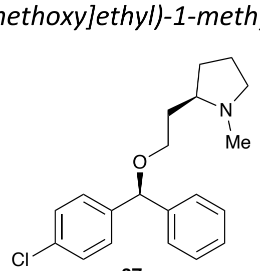

37

General procedure D was used in the reaction of (*S*)-4-chlorophenyl(phenyl)methanol **(*S*)-5a** (105 mg, 0.38 mmol) and (2*R*)-2-(2-chloroethyl)-1-methylpyrrolidine **(*R*)-3** (58 mg, 0.39 mmol). The crude product was purified by column chromatography (0 → 100% EtOAc in hexanes with 1% NEt_3) to afford the title compound as a colourless oil (27 mg, 22%). ν_{max} (ATR) 2943 (w), 2870 (w), 2776 (w), 1490 (m), 1453 (w), 1088 (s), 1015 (m). δ_H (700 MHz, $CDCl_3$) 7.32 – 7.22 (9H, m, Ar***H***), 5.28 (1H, s, Ar_2C***H***), 3.51 - 3.42 (2H, m,

2′-***H₂***), 3.05 - 3.01 (1H, m, 5-***H***H′), 2.29 (3H, s, C***H₃***), 2.17 - 2.03 (3H, m, 1′-***H***H′, 2-***H***, 5-H***H′***), 1.91 - 1.84 (1H, m, 3-***H***H′), 1.77 - 1.69 (1H, m, 4-***H***H′), 1.67 - 1.60 (1H, m, 4-H***H′***), 1.56 - 1.50 (1H, m, 1′-H***H′***), 1.46 - 1.40 (1H, m, 3-H***H′***). δ_C (176 MHz, $CDCl_3$) 142.1 (Ar***C***), 141.3 (Ar***C***), 133.2 (Ar***C***), 128.6(2) (Ar***C***), 128.5(9) (Ar***C***), 128.3 (Ar***C***), 127.7 (Ar***C***), 127.0 (Ar***C***), 83.2 (Ar_2***C***H), 67.3 (***C***-2′), 63.9 (***C***-2), 57.3 (***C***-5), 40.6 (NC***H₃***), 34.2 (***C***-1′), 31.1 (***C***-3), 22.1 (***C***-4). *m/z* (LC-MS, ESI^+) 330 ($M(^{35}Cl)H^+$), 332 ($M(^{37}Cl)H^+$). Accurate mass: Found (MH^+), 330.1643: $C_{20}H_{25}NO^{35}Cl$ requires *M*, 330.1625.

(2S) N-{2′-[(4″-Chlorophenyl)(phenyl)methoxy]ethyl}-2-methylpyrrolidine **38a**

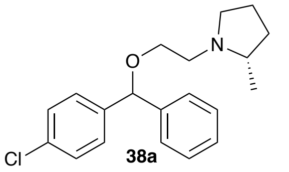

1-[(2-bromoethoxy)(phenyl)methyl]-4-chlorobenzene (310 mg, 0.95 mmol) in DMF (1 mL) was added dropwise to a suspension of the *(S)-2-methyl-pyrrolidine hydrochloride* (115 mg, 0.95 mmol), KI (17 mg, 0.10 mmol) and K_2CO_3 (263 mg, 1.90 mmol) in DMF (1.5 mL). The reaction mixture was stirred at rt for 24 h. Following the addition of H_2O (5 mL) and extraction with EtOAc (3 x 5 mL), the combined organic layers were dried and concentrated *in vacuo*. The crude product was purified by column chromatography (10% MeOH in $CHCl_3$) to afford the title compound (195 mg, 62 %) as a brown oil. ν_{max} (ATR) 2962 (w), 2869 (w), 2785 (w), 1489 (m), 1453 (w), 1375 (w), 1293 (w), 1184 (w), 1087 (s), 1014 (m). δ_H (700 MHz, $CDCl_3$) 7.32 - 7.22 (9H, m, Ar***H***), 5.36 (1H, s, Ar_2C***H***), 3.65 – 3.55 (2H, m, 2′-***H₂***), 3.20 – 3.13 (1H, m, 5-***H***H′), 3.10 – 3.04 (1H, m, 1′-***H***H′), 2.47 – 2.35 (2H, m, 5-H***H′,*** 2-***H***), 2.28 – 2.20 (1H, m, 1′ –H***H′***), 1.94 – 1.85 (1H, m, 3-***H***H′), 1.81 - 1.72 (1H, m, 4-***H***H′), 1.71 – 1.63 (1H, m, 4-H***H′***), 1.46 – 1.36 (1H, m, 3-H***H′***), 1.10 (3H, d, *J* = 6.0, C***H₃***). δ_C (176 MHz, $CDCl_3$, mixture of diastereomers) 141.9(3) (Ar***C***), 141.8(6) (Ar***C***), 141.1 (Ar***C***), 141.0 (Ar***C***), 133.2(2) (Ar***C***), 133.1(9) (Ar***C***), 128.6(0) (Ar***C***), 128.5(8) (Ar***C***), 128.5(5) (Ar***C***), 128.4(2) (Ar***C***), 128.3(8) (Ar***C***), 127.8 (Ar***C***), 127.7 (Ar***C***), 127.1 (Ar***C***), 127.0 (Ar***C***), 83.4 (Ar_2CH), 68.3(2) (***C***-2′), 68.2(9) (***C***-2′), 60.5(9) (***C***-2), 60.5(6) (***C***-2), 54.9 (***C***-5), 54.8 (***C***-5), 53.4 (***C***-1′), 53.3 (***C***-1′), 32.5 (***C***-3), 32.4 (***C***-3), 22.0(0) (***C***-4), 21.9(9) (***C***-4), 18.9(3) (***C***H_3), 18.9(1) (***C***H_3). *m/z* (LC-MS, ESI^+) 330 ($M(^{35}Cl)Na^+$), 332 ($M(^{37}Cl)Na^+$). Accurate mass: Found ($M(^{35}Cl)H^+$), 330.1636: $C_{20}H_{25}{}^{35}ClNO$ requires *M*, 330.1625.

(2R) N-{2′-[(4″-Chlorophenyl)(phenyl)methoxy]ethyl}-2-methylpyrrolidine **38b**

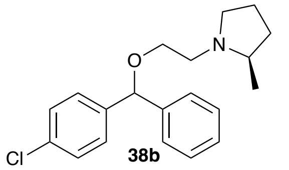

1-[(2-bromoethoxy)(phenyl)methyl]-4-chlorobenzene (326 mg, 1.03 mmol) in DMF (1 mL) was added dropwise to a suspension of the (*R*)-*2-methyl-pyrrolidine hydrochloride* (125 mg, 1.03 mmol), KI (17 mg, 0.10 mmol) and K_2CO_3 (285 mg, 2.06 mmol) in DMF (1.5 mL). The reaction mixture was stirred at rt for 24 h. Following the addition of H_2O (5 mL) and extraction with EtOAc (3 x 5 mL), the combined organic layers were dried and concentrated *in vacuo*. The crude product was purified by column chromatography (10% MeOH in $CHCl_3$) to afford the title compound (217 mg, 65 %) as a brown oil. ν_{max} (ATR) 2962 (w), 2869 (w), 2786 (w), 1490 (m), 1453 (w), 1375 (w), 1185 (w), 1088 (s) 1015 (m). δ_H (700 MHz, $CDCl_3$, mixture of diastereomers) 7.33 – 7.23 (9H, m, ArH), 5.36 (1H, s, Ar_2***H***), 3.62 – 3.55 (2H, m, 2'-***H₂***), 3.16 – 3.11 (1H, m, 5-***H***H'), 3.09 – 3.03 (1H, m, 1'-***H***H'), 2.44 - 2.38 (1H, m, 5-H***H'***), 2.38 – 2.31 (1H, m, 2-***H***), 2.24 – 2.16 (1H, m, 1' –H***H'***), 1.93 – 1.85 (1H, m, 3-***H***H'), 1.80 – 1.62 (2H, m, 4-***H₂***), 1.43 – 1.34 (1H, m, 3-H***H'***), 1.09 (1.5H, d, *J* = 6.0 Hz, C***H₃***), 1.08 (1.5H, d, *J* = 6.0 Hz, C***H₃***). δ_C (176 MHz, $CDCl_3$, mixture of diastereomers) 142.0(2) (Ar***C***), 141.9(6) (Ar***C***), 141.2 (Ar***C***), 141.1 (Ar***C***), 133.2(2) (Ar***C***), 133.1(9) (Ar***C***), 128.6(2) (Ar***C***), 128.5(7) (Ar***C***), 128.5 (Ar***C***), 128.4 (Ar***C***), 127.8 (Ar***C***), 127.7 (Ar***C***), 127.1(0) (Ar***C***), 127.0(6) (Ar***C***), 83.4 (Ar_2***C***H), 68.6(0) (***C***-2'), 68.5(6) (***C***-2'), 60.4(3) (***C***-2), 60.4(0) (***C***-2), 55.0 (***C***-5), 54.9 (***C***-5), 53.5 (***C***-1'), 53.4 (***C***-1'), 32.6 (***C***-3), 22.0 (***C*-4**), 19.2 (***C***H₃). *m/z* (LC-MS, ESI^+) 330 ($M(^{35}Cl)Na^+$), 332 ($M(^{37}Cl)Na^+$). Accurate mass: Found ($M(^{35}Cl)H^+$), 330.1630: $C_{20}H_{25}{}^{35}ClNO$ requires *M*, 330.1625.

Methyl (2R) N-{2'-[(4''-chlorophenyl)(phenyl)methoxy]ethyl}pyrrolidine-2-carboxylate **38c**

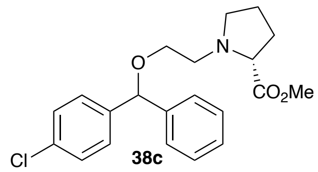

38c

1-[(2-Bromoethoxy)(phenyl)methyl]-4-chlorobenzene (462 mg, 1.42 mmol) is dissolved in MeCN (7 mL) and D-proline methyl ester hydrochloride (282 mg, 1.70 mmol) and K_2CO_3 (471 mg, 3.41 mmol) are added. The reaction is heated to 60 °C and left to stir overnight. The reaction was extracted with EtOAc (3 x 10 mL) and washed with H_2O (10 mL) and dried over Na_2SO_4. The crude product was purified by reversed phase column chromatography (5 → 100% MeCN in H_2O with 0.1% formic acid) to afford the title compound (161 mg, 30%) as a colourless oil. ν_{max} (ATR) 2950 (w), 2870 (w), 1733 (m), 1489 (m), 1455 (w), 1435 (w), 1196 (m), 1169 (m), 1087 (s), 1072 (m), 1012 (m) cm^{-1}. δ_H (700 MHz, $CDCl_3$) 7.32 – 7.20 (9H, m, Ar***H***), 5.31 – 5.29 (1H, m, Ar_2C***H***), 3.58 – 3.53 (5H, m, 2'-***H₂***, OC***H₃***), 3.35 – 3.30 (1H, m, 2-***H***), 3.21 – 3.16 (1H, m, 1'-***H***H'), 2.97 – 2.91 (1H, m, 5-***H***), 2.84 – 2.78 (1H, m, 5-***H***), 2.53 – 2.46 (1H, m, 1'-H***H'***), 2.15 – 2.06 (1H, m, 3-***H***H'), 1.93 – 1.83 (2H, m, 4-***H₂***), 1.81 – 1.74 (1H, m, 3-H***H'***). δ_C (176 MHz, $CDCl_3$, mixture of diastereomers) 174.6(8) (***C***O), 174.6(5) (***C***O), 141.8(8) (Ar***C***), 141.8(5) (Ar***C***), 141.1 (Ar***C***), 141.0 (Ar***C***), 133.2 (Ar***C***), 133.1 (Ar***C***), 128.5(7) (Ar***C***), 128.5(5) (Ar***C***), 128.5(2) (Ar***C***), 128.5(0) (Ar***C***), 128.4 (Ar***C***), 128.3 (Ar***C***), 127.7(3) (Ar***C***), 127.6(5) (Ar***C***), 127.1 (Ar***C***), 126.9 (Ar***C***), 83.3 (Ar_2***C***H), 68.2(1) (***C***-2'), 68.1(8) (***C***-2'), 65.9(4) (***C***-2), 65.9(2)

($\boldsymbol{C}$-2), 54.5 ($\boldsymbol{C}$-5), 54.0(3) ($\boldsymbol{C}$-1'), 53.9(9) ($\boldsymbol{C}$-1'), 51.7 (O$\boldsymbol{C}$H$_3$), 29.7 ($\boldsymbol{C}$-3), 29.6 ($\boldsymbol{C}$-3), 23.4(0) ($\boldsymbol{C}$-4), 23.3(9) ($\boldsymbol{C}$-4). *m/z* (LC-MS, ESI$^+$) 396.343 (M(^{35}Cl)Na$^+$), 398.256 (M(^{37}Cl)Na$^+$). Accurate mass: Found (M(^{35}Cl)H$^+$), 374.1530: $C_{21}H_{25}{}^{35}ClNO_3$ requires *M*, 374.1523.

[(2R) N-{2'-[(4"-Chlorophenyl)(phenyl)methoxy]ethyl}pyrrolidin-2-yl]methanol **38d**

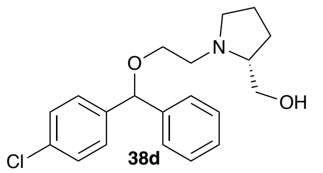

38d

To a 0 °C solution of methyl (2*R*)-1-{2-[(4-chlorophenyl)(phenyl)methoxy]ethyl}pyrrolidine-2-carboxylate **(*R*)-38c** (115 mg, 0.30 mmol) in THF (4 mL) was slowly added $LiAlH_4$ (2.4M solution in THF, 0.31 mL, 0.75 mmol), before being warmed to rt and stirred for 3h. The reaction mixture was then cooled in an ice bath and any reactive salts were quenched according to Fieser's method. The product was purified by reversed phase column chromatography (5 → 100% MeCN in H_2O with 0.1% formic acid). To afford the title compound (64 mg, 62%) as a yellow oil. ν_{max} (ATR) 3392 (br, w), 2946 (w), 2870 (w), 1489 (m), 1453 (w), 1403 (w), 1295 (w), 1185 (w), 1086 (s), 1028 (m), 1014 (s) cm^{-1}. δ_H (700 MHz, $CDCl_3$) 7.35 - 7.23 (9H, m, Ar$\boldsymbol{H}$), 5.35 (1H, s, Ar$_2$C$\boldsymbol{H}$), 3.68 – 3.63 (1H, m, HOC$\boldsymbol{H}$H'), 3.63 – 3.56 (2H, m, 2'-$\boldsymbol{H_2}$), 3.45 – 3.40 (1H, m, HOCH$\boldsymbol{H'}$), 3.27 – 3.18 (1H, m, 5–$\boldsymbol{H}$H'), 3.14 – 3.06 (1H, m, 1'-$\boldsymbol{H}$H'), 2.85 – 2.75 (1H, m, 2-$\boldsymbol{H}$), 2.71 – 2.65 (1H, m, 1-H$\boldsymbol{H'}$), 2.48 – 2.38 (1H, m, 5-H$\boldsymbol{H'}$), 1.93 – 1.85 (1H, m, 4-$\boldsymbol{H}$H'), 1.81 – 1.70 (3H, m, 4-H$\boldsymbol{H'}$, 3-$\boldsymbol{H_2}$). δ_C (176 MHz, $CDCl_3$, mixture of diastereomers) 141.7 (Ar$\boldsymbol{C}$), 141.6 (Ar$\boldsymbol{C}$), 140.9 (Ar$\boldsymbol{C}$), 140.8 (Ar$\boldsymbol{C}$), 133.4 (Ar$\boldsymbol{C}$), 133.3 (Ar$\boldsymbol{C}$), 128.6(9) (Ar$\boldsymbol{C}$), 128.6(8) (Ar$\boldsymbol{C}$), 128.6(6) (Ar$\boldsymbol{C}$), 128.3(2) (Ar$\boldsymbol{C}$), 128.2(8) (Ar$\boldsymbol{C}$), 127.9 (Ar$\boldsymbol{C}$), 127.8 (Ar$\boldsymbol{C}$), 127.0 (Ar$\boldsymbol{C}$), 126.9 (Ar$\boldsymbol{C}$), 83.5(3) (Ar$_2$C$\boldsymbol{H}$), 83.5(1) (Ar$_2$C$\boldsymbol{H}$), 67.9(5) ($\boldsymbol{C}$-2'), 67.8(9) ($\boldsymbol{C}$-2'), 65.7 ($\boldsymbol{C}$-2), 62.4(1) ($\boldsymbol{C}$H$_2$OH), 62.3(8) ($\boldsymbol{C}$H$_2$OH), 55.1(8) ($\boldsymbol{C}$-5), 55.1(6) ($\boldsymbol{C}$-5), 54.5 ($\boldsymbol{C}$-1'), 27.5 ($\boldsymbol{C}$-3), 24.1 ($\boldsymbol{C}$-4). *m/z* (LC-MS, ESI$^+$) 368 (M(^{35}Cl)Na$^+$), 370 (M(^{37}Cl)Na$^+$). Accurate mass: Found (M(^{35}Cl)H$^+$), 346.1579: $C_{20}H_{25}{}^{35}ClNO_2$ requires *M*, 346.1574.

(2R) N-{2-[(4-chlorophenyl)(phenyl)methoxy]ethyl}-2-(methoxymethyl)pyrrolidine **38e**

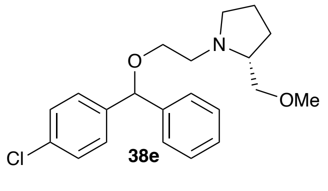

38e

1-[(2-bromoethoxy)(phenyl)methyl]-4-chlorobenzene (160 mg, 0.49 mmol) in DMF (0.8 mL) was added dropwise to a suspension of (2*R*) 2-*methoxymethylpyrrolidine* (0.06 mL, 0.49 mmol), KI (8.3 mg, 0.08 mmol) and K_2CO_3 (135 mg, 0.98 mmol) in DMF (0.5 mL). The reaction mixture was stirred at rt for 4 h. Following the addition of H_2O (5 mL) and extraction with EtOAc (3 x 5mL), the combined organic layers were dried over Na_2SO_4 and concentrated *in vacuo*. The crude product was purified by column

chromatography (10% MeOH in $CHCl_3$) to afford the title compound (114 mg, 65 %) as a brown oil. ν_{max} (ATR) 2871 (w), 2810 (w), 1489 (m), 1453 (w), 1185 (w), 1088 (s), 1015 (m). δ_H (700 MHz, $CDCl_3$) 7.33 – 7.23 (9H, m, Ar***H***), 5.37 (1H, s, Ar_2C***H***), 3.74 – 3.57 (2H, m, 2'-***H₂***), 3.56 - 3.41 (1H, m, 1-***H***H'), 3.37 – 3.29 (4H, m, C***H₃***, 1-H***H'***), 3.29 – 3.11 (2H, m, 1'-***H***H', 5-***H***H'), 2.95 – 2.60 (2H, m, 1'-H***H'***, 2-***H***), 2.58 – 2.32 (1H, m, 5-H***H'***), 1.97 – 1.86 (1H, m, 3-***H***H'), 1.86 – 1.70 (2H, m, 4-***H₂***), 1.70 – 1.57 (1H, m, 3-H***H'***). δ_C (176 MHz, $CDCl_3$, mixture of diastereomers) 141.8 (Ar***C***), 141.7 (Ar***C***), 141.0 (Ar***C***), 140.9 (Ar***C***), 133.3 (Ar***C***), 133.2 (Ar***C***), 128.6(3) (Ar***C***), 128.6(1) (Ar***C***), 128.5(9) (Ar***C***), 128.4(4) (Ar***C***), 128.3(8) (Ar***C***), 127.8(2) (Ar***C***), 127.7(7) (Ar***C***), 127.1 (Ar***C***), 127.0 (Ar***C***), 83.4 (Ar_2CH), 75.4 (***C***-1), 67.8 (***C***-2'), 64.3 (***C***-2), 59.2 (***C***H₃), 55.6 (***C***-5), 55.5 (***C***-5), 54.9 (***C***-1'), 28.0 (***C***-3), 23.2 (***C***-4). *m/z* (LC-MS, ESI$^+$) 382 (M(^{35}Cl)Na$^+$), 384 (M(^{37}Cl)Na$^+$). Accurate mass: Found (MH$^+$), 360.1734: $C_{21}H_{27}NO_2{}^{35}Cl$ requires *M*, 360.1730.

N-{2'-[(4"-Chlorophenyl)(phenyl)methoxy]ethyl}pyrrolidine **38f**

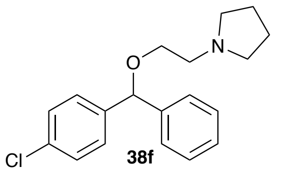

1-[(2-Bromoethoxy)(phenyl)methyl]-4-chlorobenzene (117 mg, 0.36 mmol) was dissolved in MeCN (3 mL) and pyrrolidine (0.04 mL, 0.43 mmol) and K_2CO_3 (69 mg, 0.50 mmol) were added. The reaction was heated to 60 °C and left to stir overnight. The reaction mixture was extracted with EtOAc (3 x 5 mL) and washed with H_2O (5 mL) and dried over Na_2SO_4. The crude product was purified by reversed phase column chromatography (5 → 100% MeCN in H_2O with 0.1% formic acid) to afford the title compound (61 mg, 54%) as a yellow oil. ν_{max} (ATR) 2962 (w), 2787 (w), 1490 (m), 1453 (w), 1088 (s), 1015 (m) cm^{-1}. δ_H (700 MHz, $CDCl_3$) 7.33 – 7.23 (9H, m, Ar***H***), 5.35 (1H, s, Ar_2C***H***), 3.62 (2H, t, *J* = 6.0 Hz, 2'-***H₂***), 2.82 (2H, t, *J* = 6.0 Hz, 1'-***H₂***), 2.66 – 2.61 (4H, m, 2-***H₂***, 5-***H₂***), 1.82 – 1.76 (4H, m, 3-***H₂***, 4-***H₂***). δ_C (176 MHz, $CDCl_3$) 141.8 (Ar***C***), 141.0 (Ar***C***), 133.3 (Ar***C***), 128.6(2) (Ar***C***), 128.5(9) (Ar***C***), 128.4 (Ar***C***), 127.8 (Ar***C***), 127.1 (Ar***C***), 83.4 (Ar_2***C***H), 68.2 (***C***-2'), 55.7 (***C***-1'), 54.8 (***C***-2, ***C***-5), 23.6 (***C***-3, ***C***-4). *m/z* (LC-MS, ESI$^+$) 316 (M(^{35}Cl)H$^+$), 318 (M(^{37}Cl)H$^+$). Accurate mass: Found (M(^{35}Cl)H$^+$), 316.1461: $C_{19}H_{23}{}^{35}ClNO$ requires *M*, 316.1468.

(2S) N-{4'-[(4"-chlorophenyl)(phenyl)methoxy]but-1'-yl}-2-methylpyrrolidine **38g**

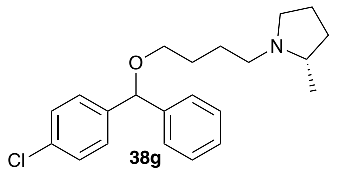

1-[(4-bromobutoxy)(phenyl)methyl]-4-chlorobenzene (106 mg, 0.3 mmol) in DMF (0.5 mL) was added dropwise to a suspension of *(2R)-2-methylpyrrolidine hydrochloride* (46 mg, 0.38 mmol), KI (7 mg, 0.04 mmol) and K_2CO_3 (105 mg, 0.76 mmol) in DMF (1 mL). The reaction was stirred at rt for 24 h. Following the addition of EtOAc (10 mL), the organic layer was washed with H_2O (5 x 10 mL), dried over Na_2SO_4, filtered and concentrated *in vacuo*. The crude product was purified by column chromatography (0 → 100% EtOAc in hexanes with 1% NEt_3) to afford the title compound as a colourless oil (49 mg, 46 %). ν_{max} (ATR) 2954 (w), 2867 (w), 2789 (w), 1490 (m), 1453 (w), 1089 (s), 1015 (m). δ_H (600 MHz, $CDCl_3$) 7.31 – 7.28 (4H, m, Ar***H***), 7.27 - 7.21 (5H, m, Ar***H***), 5.28 (1H, s, Ar_2C***H***), 3.47 – 3.41 (2H, m, 3'-***H₂***), 3.18 – 3.12 (1H, m, 5-***H***H'), 2.81 – 2.74 (1H, m, 1'-***H***H'), 2.29 – 2.19 (1H, m, 2-***H***), 2.09 –1.96 (2H, m, 5-H***H'***, 1'-H***H'***), 1.93 – 1.85 (1H, m, 3-***H***H'), 1.81 – 1.72 (1H, m, 4-***H***H'), 1.72 – 1.55 (5H, m, 4-H***H'***, 3'-***H₂***, 2'-***H₂***), 1.45 – 1.38 (1H, m, 3-H***H'***), 1.07 (3H, d, *J* = 6.0, C***H₃***). δ_C (176 MHz, $CDCl_3$, mixture of diastereomers) 142.2(1) (***C***-$1'^{Ph}$), 142.2(0) (***C***-$1'^{Ph}$), 141.3 (***C***-1^{Ph}), 133.2 (***C***-4^{Ph}), 128.6(0) (Ar***C***), 128.5(9) (Ar***C***), 128.5(7) (Ar***C***), 128.5(6) (Ar***C***), 128.3(9) (Ar***C***), 128.3(8) (Ar***C***), 127.7 (***C***-$4'^{Ph}$), 127.0(3) (Ar***C***), 127.0(1) (Ar***C***), 83.1 (Ar_2CH), 69.1(6) (***C***-4'), 69.1(5) (***C***-4'), 60.5 (***C***-2), 54.3 (***C***-1'), 54.2 (***C***-1'), 54.1 (***C***-5), 32.8 (***C***-3), 28.2(4) (***C***-2'), 28.2(3) (***C***-2'), 25.7 (***C***-3'), 21.7 (***C***-4), 19.0 (***C***H_3). *m/z* (LC-MS, ESI^+) 358 ($M(^{35}Cl)H^+$), 360 ($M(^{37}Cl)H^+$). Accurate mass: Found (MH^+), 358.1949: $C_{22}H_{28}{}^{35}ClNO$ requires *M,* 358.1938.

(2S) N-{3'-[(4"-chlorophenyl)(phenyl)methoxy]prop-1'-yl}-2-methylpyrrolidine **38h**

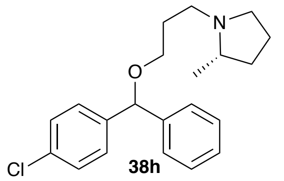

38h

1-[(3'-bromopropoxy)(phenyl)methyl]-4-chlorobenzene (320 mg, 0.94 mmol) in DMF (5 mL) was added dropwise to a suspension of (2*S*)-2-methylpyrrolidine hydrochloride (115 mg, 0.94 mmol), KI (17g, 0.1 mmol) and K_2CO_3 (210mg, 1.88 mmol) in DMF (10 mL). The reaction was then stirred at rt for 24 h. Following the addition of EtOAc (10 mL), the organic layer was washed with H_2O (5 x 10 mL), dried over Na_2SO_4, filtered and concentrated *in vacuo*. The crude product was purified by column chromatography (10% MeOH in $CHCl_3$) to afford the title compound (217 mg, 65 %) as a colourless oil. ν_{max} (ATR) 2960 (w), 2869 (w), 2789 (w), 1489 (m), 1087 (s), 1014 (m). δ_H (600 MHz, $CDCl_3$, mixture of diastereomers) 7.32 – 7.29 (4H, m, Ar***H***), 7.28 – 7.26 (4H, m, Ar***H***), 7.26 – 7.22 (1H, m, Ar***H***), 5.30 (1H, s, Ar_2C***H***), 3.51 – 3.47 (2H, m, 3'-***H₂***), 3.17 – 3.09 (1H, m, 5-***H***H'), 2.95 – 2.86 (1H, m, 1'-***H***H'), 2.31 – 2.22 (1H, m, 2-***H***), 2.16 – 2.10 (1H, m, 1'-H***H'***), 2.10 – 2.04 (1H, m, 5-H***H'***), 1.93 – 1.81 (3H, m, 2'-***H₂***, 3-***H***H'), 1.80 – 1.71 (1H, m, 4-***H***H'), 1.70 – 1.62 (1H, m, 4-H***H'***), 1.46 – 1.36 (1H, m, 3-H***H'***), 1.08 (1.5H, d, *J* = 6.0, C***H₃***), 1.08 (1.5H, d, *J* = 6.0, C***H₃***). δ_C (151 MHz, $CDCl_3$, mixture of diastereomers) 142.2 (***C***-$1'^{Ph}$), 141.2(8) (***C***-1^{Ph}), 141.2(8) (***C***-1^{Ph}), 133.1(3) (***C***-4^{Ph}), 133.1(2) (***C***-4^{Ph}), 128.5(7) (Ar***C***), 128.5(5) (Ar***C***), 128.5(4) (Ar***C***), 128.5(2) (Ar***C***), 128.4 (Ar***C***), 127.6(6)

(**C**-4′^{Ph}), 127.6(5) (**C**-4′^{Ph}), 127.0 (Ar**C**), 83.0 (Ar_2**C**H), 67.8(3) (**C**-3′), 67.8(0) (**C**-3′), 60.3 (**C**-2), 54.0(7) (**C**-5), 54.0(6) (**C**-5), 51.3 (**C**-1′), 51.2 (**C**-1′), 32.8 (**C**-3), 29.2 (**C**-2′). 21.7 (**C**-4), 19.1 (**C**H_3). m/z (LC-MS, ESI$^+$) 344 (M(^{35}Cl)H$^+$), 346 (M(^{37}Cl)H$^+$). Accurate mass: Found (M(^{35}Cl)H$^+$), 344.1784: $C_{21}H_{27}{}^{35}ClNO$ requires *M,* 344.1781.

(2S) N-{2′-[(S)-(4″-chlorophenyl)(phenyl)methoxy]ethyl}-2-methylpyrrolidine **38i**

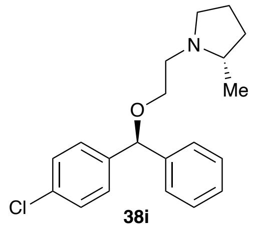

38i

General procedure E was used in the reaction of *(S)-4-chlorophenyl(phenyl)methanol* **(*S*)-5a** (119 mg, 0.43 mmol) and *(2S) N-(2′-chloroethyl)-2-methylpyrrolidine* (63 mg, 0.43 mmol). The crude product was purified by flash column chromatography (0 → 100% EtOAc in hexanes with 1% NEt_3) to afford the title compound (78mg, 55%) as a colourless oil. ν_{max} (ATR) 2962 (w), 2869 (w), 2787 (w), 1489 (m), 1453 (w), 1375 (w), 1086 (s), 1014 (m). δ_H (700 MHz, $CDCl_3$) 7.31 - 7.22 (9H, m, Ar***H***), 5.34 (1H, s, Ar_2C***H***), 3.57 (2H, t, *J* = 6.5 Hz, 2′-***H₂***), 3.15 – 3.09 (1H, m, 5-***H***H′), 3.04 (1H, dt, *J* = 12.5, 6.5 Hz, 1′-***H***H′), 2.39 (1H, dt, *J* = 12.5, 6.5 Hz, 1′-H***H′***), 2.36 – 2.30 (1H, m, 2-***H***), 2.22 – 2.16 (1H, m, 5-H***H′***), 1.91 – 1.84 (1H, m, 3-***H***H′), 1.78 – 1.69 (1H, m, 4-***H***H′), 1.69 - 1.61 (1H, m, 4-H***H′***), 1.41 - 1.33 (1H, m, 3-***H***H′), 1.07 (3H, d, *J* = 6.0 Hz, CH_3). δ_C (176 MHz, $CDCl_3$) 142.0 (Ar***C***), 141.2 (Ar***C***), 133.2 (Ar***C***), 128.6(1) (Ar***C***), 128.6(0) (Ar***C***), 128.4 (Ar***C***), 127.8 (Ar***C***), 127.1 (Ar***C***), 83.4 (Ar_2C***H***), 68.6 (***C***-2′), 60.5 (***C***-2), 54.9 (***C***-5), 53.4 (***C***-1′), 32.6 (***C***-3), 22.0 (***C***-4), 19.1 (***C***H_3). *m/z* (LC-MS, ESI$^+$) 330 (M(^{35}Cl)H$^+$), 332 (M(^{37}Cl)H$^+$). Accurate mass: Found (MH$^+$), 330.1624: $C_{20}H_{25}NO^{35}Cl$ requires *M*, 330.1625.

(2R) N-{2′-[(S)-(4″-chlorophenyl)(phenyl)methoxy]ethyl}-2-methylpyrrolidine **38j**

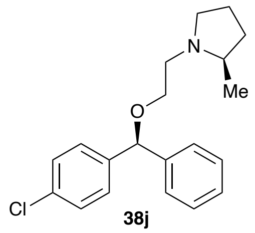

38j

General procedure E was used in the reaction of *(S)-4-chlorophenyl(phenyl)methanol* **(*S*)-5b** (67 mg, 0.24 mmol) and *(2R)-1-(2′-chloroethyl)-2-methylpyrrolidine* (12 mg, 0.08 mmol). The crude product was purified by flash column chromatography (0 → 100% EtOAc in hexanes with 1% NEt_3) to afford the title compound (15 mg, 57 %) as a colourless oil. ν_{max} (ATR) 2963 (w), 2870 (w), 2789 (w), 1490 (w), 1453 (w), 1375 (w), 1089 (m), 1015 (w). δ_H (700 MHz, $CDCl_3$) 7.33 – 7.29 (4H, m, Ar***H***), 7.29 - 7.27 (4H, m, Ar***H***), 7.26

– 7.23 (1H, m, Ar***H***), 5.36 (1H, s, Ar_2C***H***), 3.69 – 3.54 (2H, m, 2'-$\boldsymbol{H_2}$), 3.22 - 3.14 (1H, m, 5-***H***H'), 3.12 – 3.05 (1H, m, 1'-***H***H'), 2.54 – 2.36 (2H, m, 1-H***H'***, 2-***H***), 2.34 – 2.22 (1H, m, 5-H***H'***), 1.98 – 1.86 (1H, m, 3-***H***H'), 1.84 – 1.74 (1H, m, 4-***H***H'), 1.73 – 1.64 (1H, m, 4-H***H'***), 1.49 – 1.36 (1H, m, 3-H***H'***), 1.12 (3H, d, *J* = 6.0 Hz, C$\boldsymbol{H_3}$). δ_C (176 MHz, $CDCl_3$) 141.9 (Ar***C***), 141.0 (Ar***C***), 133.3 (Ar***C***), 128.7 (Ar***C***), 128.6 (Ar***C***), 128.5 (Ar***C***), 127.8 (Ar***C***), 127.1 (Ar***C***), 83.5 (Ar_2***C***H), 68.2 (***C***-2'), 60.7 (***C***-2), 54.8 (***C***-5), 53.4 (***C***-1'), 32.4 (***C***-3), 22.0 (***C***-4), 18.9 (***C***H_3). *m/z* (LC-MS, ESI^+) 330 ($M(^{35}Cl)H^+$), 332 ($M(^{37}Cl)H^+$). Accurate mass: Found (MH^+), 330.1634: $C_{20}H_{25}NO^{35}Cl$ requires *M*, 330.1625.

(2R) N-{2'-[(R)-(4"-chlorophenyl)(phenyl)methoxy]ethyl}-2-methylpyrrolidine **38k**

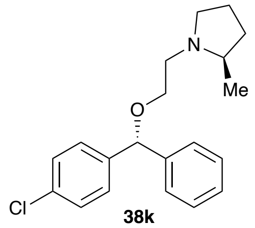

General procedure E was used in the reaction of *(R)-4-chlorophenyl(phenyl)methanol* (100 mg, 0.36 mmol) and *(2R)-1-(2-chloroethyl)-2-methylpyrrolidine* (***R*)-10b** (53 mg, 0.36 mmol). The crude product was purified by flash column chromatography (0 → 100% EtOAc in hexanes with 1% NEt_3) to afford the title compound as a colourless oil (72 mg, 61%). ν_{max} (ATR) 2962 (w), 2869 (w), 2788 (w), 1490 (w), 1088 (m), 1015 (w). δ_H (700 MHz, $CDCl_3$) 7.32 - 7.20 (9H, m, Ar***H***), 5.35 (1H, s, Ar_2C***H***), 3.58 (2H, td, *J* = 6.5, 1.5 Hz, 2'-$\boldsymbol{H_2}$), 3.16 – 3.12 (1H, m, 5-***H***H'), 3.06 (1H, dt, *J* = 12.5, 6.5 Hz, 1'-***H***H'), 2.41 (1H, dt, *J* = 12.5, 6.5 Hz, 1'-H***H'***), 2.38 – 2.32 (1H, m, 2-***H***), 2.24 – 2.18 (1H, m, 5-H***H'***), 1.91 – 1.85 (1H, m, 3-***H***H'), 1.80 – 1.71 (1H, m, 4-***H***H'), 1.70 - 1.63 (1H, m, 4-H***H'***), 1.42-1.35 (1H, m, 3-H***H'***), 1.09 (3H, d, *J* = 6.0 Hz, C$\boldsymbol{H_3}$). δ_C (176 MHz, $CDCl_3$) 141.9 (Ar***C***), 141.1 (Ar***C***), 133.2 (Ar***C***), 128.5(8) (Ar***C***), 128.5(7) (ArC), 128.4 (Ar***C***), 127.7 (Ar***C***), 127.1 (Ar***C***), 83.4 (Ar_2CH), 68.5 (***C***-2'), 60.5 (***C***-2), 54.9 (***C***-5), 53.4 (***C***-1'), 32.5 (***C***-3), 22.0 (***C***-4), 19.1 (***C***H_3). m/z (LC-MS, ESI^+) 330 ($M(^{35}Cl)H^+$), 332 ($M(^{37}Cl)H^+$). Accurate mass: Found (MH^+), 330.1632: $C_{20}H_{25}NO^{35}Cl$ requires *M*, 330.1625.

(2S) N-{3'-[(R)-(4"-chlorophenyl)(phenyl)methoxy]propyl}-2-methylpyrrolidine **38l**

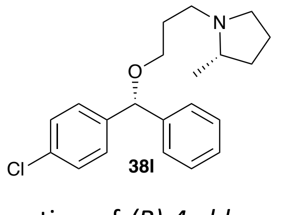

General procedure E was used for the reaction of *(R)-4-chlorophenyl(phenyl)methanol* **5b** (76 mg, 0.27 mmol) and *(2R)-1-(2-chloropropyl)-2-methylpyrrolidine* (101 mg, 0.62 mmol). The crude product was purified by flash column chromatography (0 → 100% EtOAc in hexanes with 1% NEt_3) to afford the title

compound (78 mg, 55%) as a colourless oil. ν_{max} (ATR) 2960 (w), 1489 (w), 1089 (w**)**. δ_H (700 MHz, CDCl$_3$) 7.32 – 7.29 (4H, m, Ar***H***), 7.27 – 7.22 (5H, m, Ar***H***), 5.30 (1H, s, Ar$_2$C***H***), 3.49 (2H, td, *J* = 6.0, 1.5 Hz, 3'-***H$_2$***), 3.21 – 3.15 (1H, m, 5-***H***H'), 2.98 – 2.90 (1H, m, 1'-***H***H'), 2.39 – 2.31 (1H, m, 2-***H***), 2.22 – 2.10 (2H, m, 1'-H***H'***, 5-H***H'***), 1.96 – 1.85 (3H, m, 2'-***H$_2$***, 3-***H***H'), 1.83 – 1.75 (1H, m, 4-***H***H'), 1.73 – 1.65 (1H, m, 4-H***H'***), 1.50 – 1.42 (1H, m, 3-H***H'***), 1.12 (3H, d, *J* = 6.0 Hz, C***H$_3$***). δ_C (176 MHz, CDCl$_3$) 142.1 (***C***-1'^{Ph}), 141.2 (***C***-1^{Ph}), 133.2 (***C***-4^{Ph}), 128.5(7) (Ar***C***), 128.5(6) (Ar***C***), 128.4 (Ar***C***), 127.7 (***C***-4'^{Ph}), 127.0 (Ar***C***), 83.0 (Ar$_2$***C***H), 67.6 (***C***-3'), 60.7 (***C***-2), 53.9 (***C***-5), 51.2 (***C***-1'), 32.7 (***C***-3), 28.9 (***C***-2'), 21.7 (***C***-4), 18.7 (***C***H$_3$). *m/z* (LC-MS, ESI$^+$) 344 (M(^{35}Cl)H$^+$), 346 (M(^{37}Cl)H$^+$). Accurate mass: Found (MH$^+$), 344.1783: C$_{21}$H$_{27}$NO^{35}Cl requires *M,* 344.1781.

(2R) N-{3'-[(R)-(4''-chlorophenyl)(phenyl)methoxy]propyl}-2-methylpyrrolidine **38m**

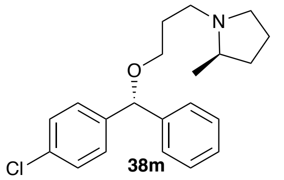

General procedure E was used for the reaction of *(R)-(4-chlorophenyl)(phenyl)methanol* ***R*-5b** (100 mg, 0.45 mmol) and *(2R)-1-(3-chloropropyl)-2-methylpyrrolidine hydrochloride* (109 mg, 0.55 mmol). The crude product was purified by flash column chromatography (0 → 100% EtOAc in hexanes with 1% NEt$_3$) to afford the title compound (66 mg, 42%) as a colourless oil. δ_H (400 MHz, CDCl$_3$) δ 7.32 – 7.30 (m, 4H), 7.29 – 7.27 (m, 5H), 5.31 (s, 1H), 3.49 (t, J = 6.4 Hz, 2H), 3.19 – 3.08 (m, 1H), 2.90 (ddd, J = 11.7, 9.5, 6.7 Hz, 1H), 2.24 (dt, J = 8.6, 6.5 Hz, 1H), 2.14 – 2.02 (m, 2H), 1.96 – 1.63 (m, 5H), 1.41 (dddd, J = 12.3, 10.6, 8.7, 6.0 Hz, 1H), 1.07 (d, J = 6.1 Hz, 3H). δ_C (101 MHz, CDCl$_3$) δ 141.85, 140.96, 132.84, 128.30, 128.26, 128.07, 127.38, 126.71, 82.65, 67.58, 60.00, 53.81, 51.01, 32.52, 28.95, 21.40, 18.84. Accurate mass: Found (MH$^+$), 344.1764: C$_{21}$H$_{27}$NO^{35}Cl requires *M,* 344.1781.

1-{3-[(R)-(4-chlorophenyl)(phenyl)methoxy]propyl}-2,2-dimethylpyrrolidine **38n**

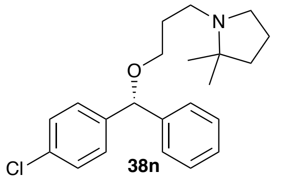

General procedure E was used for the reaction of *(R)-4-chlorophenyl(phenyl)methanol* **(*R*)-*5b*** (100 mg, 0.46 mmol) and *1-(3-chloropropyl)-2,2-dimethylpyrrolidine hydrochloride* (116. 4 mg, 0.55 mmol) The crude product was purified by flash column chromatography (0→100% EtOAc in hexanes with 1% NEt$_3$) to afford the title compound as a colourless oil. δ_H (400 MHz, CDCl$_3$) δ 7.31 (d, J = 3.9 Hz, 4H), 7.27 (d, J = 6.8 Hz, 5H), 5.31 (s, 1H), 3.50 (t, J = 6.3 Hz, 2H), 2.72 (t, J = 6.9 Hz, 2H), 2.46 (t, J = 7.4 Hz, 2H), 1.86 – 1.68 (m, 4H), 1.63 (t, J = 7.9 Hz, 2H), 0.96 (s, 6H). δ_C (101 MHz, CDCl$_3$) δ 142.26, 141.38, 133.16, 128.60, 128.57,

128.45, 127.69, 127.08, 82.98, 67.83, 51.06, 45.86, 40.02, 29.92, 22.80, 22.74, 20.52. Accurate mass: Found (MH^+), 358.1927: $C_{22}H_{29}NO^{35}Cl$ requires *M,* 358.1930.

1-{3-[(R)-(4-chlorophenyl)(phenyl)methoxy]propyl}pyrrolidine **38o**

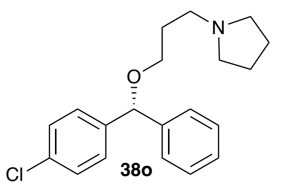

38o

General procedure E was used for the reaction of *(R)-4-chlorophenyl(phenyl)methanol* ***(R)-5b*** (100 mg, 0.46 mmol) and *1-(3-chloropropyl)pyrrolidine hydrochloride* (101 mg, 0.55 mmol). The crude product was purified by flash column chromatography (0→100% EtOAc in hexanes with 1% NEt_3) to afford the title compound as a colourless oil. δ_H (400 MHz, $CDCl_3$) δ 7.34 – 7.29 (m, 4H), 7.27 (d, J = 5.6 Hz, 5H), 5.31 (s, 1H), 3.49 (t, J = 6.4 Hz, 2H), 2.57 – 2.52 (m, 2H), 2.52 – 2.44 (m, 4H), 1.91 – 1.82 (m, 2H), 1.76 (q, J = 3.4 Hz, 4H). δ_C (101 MHz, $CDCl_3$) δ 142.19, 141.31, 133.18, 128.61, 128.58, 128.40, 127.71, 127.03, 83.00, 67.79, 54.38, 53.64, 29.61, 23.58. Accurate mass: Found (MH^+), 330.1622: $C_{20}H_{25}NO^{35}Cl$ requires *M,* 358.1625.

4.2. Biological Methods

4.2.1. General experimental details

MATERIALS: Biological grade materials, solvents, reagents and media components were purchased from commercial suppliers and used as provided. NBD-C_6-ceramide **80** was obtained from Invitrogen and AG 4-X4 ion exchange resin from Bio-Rad. Reactions and media were prepared using ultrapure water from Milli-Q® water purification system. FBS refers to heat-inactivated foetal bovine serum. Solutions of test compounds were made up in DMSO, unless otherwise stated.

INSTRUMENTS AND EQUIPMENT: 1.5 mL Eppendorfs were used during the preparation of serial dilutions. Media were filter-sterilised using a vacuum filter with a 0.22 µm pore CA membrane. Centrifugation steps were carried out using Sorvall® Legend *RT* centrifuge, Sorvall® Legend Micro 17R centrifuge, Beckman Coulter® centrifuges and ultracentrifuges. Eppendorf tubes were centrifuged using Sigma 1-14 microfuge. Disruption of yeast cells was performed using an IKA® Vortex Genius 3. Protein content and optical density (OD) were determined using a Boeco S-32 spectrophotometer. Eppendorf contents were dried using an Eppendorf Vacuum Concentrator 5301. Cells were counted using a Neubauer haemocytometer. 96-well plates used were Nest Biotechnology Co., Ltd cell culture plates (clear); Corning® Costar® cell culture plates (clear); Corning® V-bottom (clear); MultiScreen® Solvinert filter plates (Merck Millipore) and PerkinElmer OptiPlate-96 (black). 24-well plates were supplied by Nest Biotechnology Co., Ltd and cover slips from Thomas Scientific®. Fluorescence quantification was carried out using SpectraMax® microplate reader with SoftMax® Pro 6.4 data analysis software from Molecular Devices and Synergy H4 and FLx800 microplate readers with Gen5® 1.08 data analysis software from Biotek. HPTLC silica plates were from Merck Millipore and imaged using a Fuji FLA-3000 plate reader with AIDA image analyser® (version 3.52).

4.2.2. Protocols

All of the following biological procedures were carried out under sterile conditions unless otherwise stated.

4.2.2.1. *Leishmania* culture

Leishmania amazonensis (MHOM/Br/75/JOSEFA), *Leishmania major* (FV1) WT and Δ *LCB2* promastigotes were maintained at 26 °C in Schneider's insect medium at pH 7, supplemented with 15% FBS. *Leishmania major* (FV1) PX promastigotes were maintained at 26 °C in Schneider's insect medium at pH 7,

supplemented with 15% FBS and 40 μg mL^{-1} G418 (Gibco BRL). *Leishmania amazonensis*-GFP were selected for bright green fluorescence by 48 h-incubation in the presence of 1000 μg mL^{-1} G418 (Gibco BRL).

4.2.2.2. Animals and ethics statement

All mice used in the experiments were maintained under controlled temperature, filtered air and water, autoclave bedding, and commercial food at the animal facilities at Federal University of Rio de Janeiro.

The animal protocols for this study were approved by the Federal University of Rio de Janeiro Institutional Animal Care and Use Committee under the number 030/17. The research was conducted in compliance with the principles stated in the *Guide for the Care and Use of Laboratory Animals* (NIH). [9]

4.2.2.3. Isolation of Bone Marrow Derived Macrophages (BMDM)

BMDM were differentiated from bone marrow of BALB/C, C57BL/6 and knock out C57BL/6 mice using L929-cell conditioned medium (LCCM) as a source of macrophage colony-stimulating factor (M-CSF) as described by Zamboni *et al.*[272] Briefly, bone marrow was extracted from the femurs and tibias and re-suspended in bone marrow differentiated media (which is RPMI 1640 medium supplemented with 20% LCCM) in Petri dishes for 7 days at 37 °C with 5% CO_2. After, the plates were washed with warm PBS to remove detached cells, the adherent BMDM were gently scraped off the surface and re-suspended in RPMI (without LCCM). These cells are ready to use in the assays described in section 4.2.3.

4.2.2.4. Drugs

Clemastine fumarate, amphotericin B and cycloheximide were purchased from Sigma-Aldrich. Glucantime solution (meglumine antimoniate, 300 mg mL^{-1}) was a gift from Sanofi Aventis. Clemastine derivatives were synthesised using the procedure outline in the previous section. Stock solutions of clemastine and its derivatives (10 mM) were prepared in dimethyl sulfoxide (DMSO) and kept at 0 - 4° C. Subsequent dilutions were done in culture media. For *in vitro* assays, all drugs were serially diluted in 100% DMSO, and then diluted 1:100 in culture medium, so that all final drug concentrations contained 1% DMSO.

4.2.3. Assays

4.2.3.1. Anti-promastigote assays

L. major promastigotes (FV1) WT, PX and Δ *LCB2* in Schneider's insect medium (100 µL at 1×10^6 mL^{-1}) were incubated in 96-well plates with compounds in triplicate (amphotericin B and cycloheximide were used as positive controls, and untreated parasites with 1% DMSO as a negative control) at 26 °C for 48 h. Resazurin Solution (10 µL) was then added and the plate incubated at 26 °C for 4 h prior to measurement using a fluorescence plate reader (555 - 585 nm). EC_{50} values were calculated using sigmoidal regression analysis (GraphPad Prism).

Protocol 1: *L. amazonensis* promastigotes in Medium 199 (100 µL at 5×10^5 mL^{-1}) were incubated in 96-well plates with compounds in triplicate (amphotericin B was used as a positive control, and untreated parasites with DMSO as a negative control) at 26 °C for 72 h. Resazurin Solution (10 µL) was then added and the plate incubated at 26 °C for 4 h prior to measurement using a fluorescence plate reader (555 - 585 nm). EC_{50} values were calculated using sigmoidal regression analysis (GraphPad Prism).
Protocol 2: Same procedure as above with the modification of a 48 h incubation time as opposed to 72 h and *Leishmania amazonensis* (MHOM/Br/75/JOSEFA) in Schneider's insect medium in the place of *Leishmania amazonensis* (MHOM/Br/75/JOSEFA) in Medium 199.

4.2.3.2. Anti-amastigote intramacrophage assay

L929-cell conditioned medium (LCCM) as a source of macrophage colony-stimulating factor (M-CSF) as described by Zamboni *et al.*[10] BMDM were diluted in RPMI 1640 medium to a concentration of 2×10^5 $well^{-1}$ in a 24-well plate with round cover slips and incubated for 24 h at 37 °C and 5% CO_2. Bone marrow derived macrophages obtained as in 4.2.2.3. were diluted in RPMI 1640 medium to a concentration of 2×10^5 $well^{-1}$ in a 24-well plate with round cover slips and incubated for 24 h at 37 °C and 5% CO_2. They were infected with *L. amazonensis* promastigotes (10:1) at 37 °C for 4 h. Then washed with PBS twice to remove extracellular promastigotes and fresh RPMI medium supplemented with 5% FBS was added. After 24 h serial dilutions of the test compounds in RPMI medium (350 µL) were added and the cells were incubated at 37 °C for 48 h. The adherent infected cells were then stained with Giemsa modified solution and amastigotes were counted using an optical Nikon® microscope.

4.2.3.3. Macrophage cytotoxicity assay

Bone marrow derived macrophages in RPMI 1640 medium were seeded (1×10^6 mL^{-1}, 100 µL $well^{-1}$) in 96-well plates and incubated for 24 h at 37 °C and 5% CO_2. Following removal of media, serial dilutions of the test compounds in fresh RPMI medium (100 µL) were added and the cells were incubated for 48 h at 37

°C and 5% CO_2. Aliquots of resazurin solution (10 µL) were then added and the cells were incubated at 37 °C and 5% CO_2 for 4 h. Cell-viability measurement was carried out using a fluorescence plate reader.

4.2.3.4. *In vivo* assay

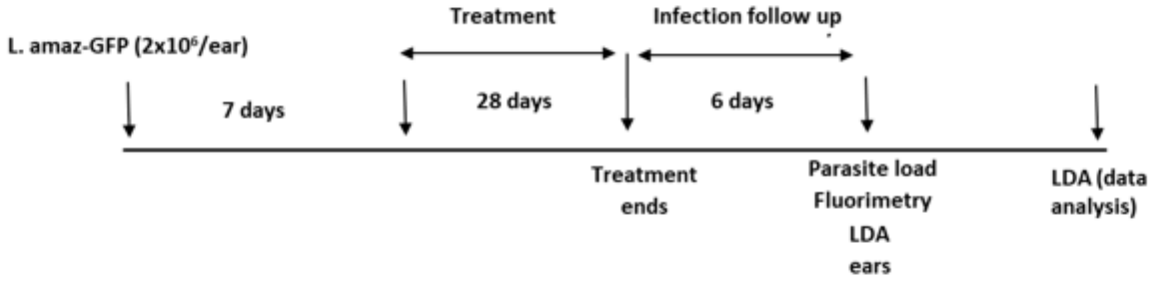

In vivo assays were conducted as previously reported [1]. Two month old BALB/c female mice, weighing 20 - 25 g and of approximately the same age were used for the study. For infection of mice, stationary phase GFP *L. amazonensis* promastigotes were collected, washed and suspended in sterile PBS. A volume of 10 µl of sterile PBS containing 2 x 10^6 parasites was injected into the right ear. On day seven of infection, animals were randomly distributed into 5 groups; oral clemastine fumarate (5 animals), IP clemastine fumarate (5 animals), IL clemastine fumarate (5 animals), IP glucantime solution (5 animals) and untreated (6 animals). Mice were treated with clemastine fumarate at a dose of 134 mg kg^{-1} by oral gavage five times a week for 28 days. Mice were treated with clemastine fumarate and glucantime solution at a dose of 11.65 mg kg^{-1} and 1.30 g kg^{-1} respectively, by intraperitoneal injection twice a week for 28 days. Mice were treated with clemastine fumarate at a dose of 1.17 mg kg^{-1} by intralesional injection twice a week for 28 days. Infected ear thicknesses were measured once or twice a week with a caliper gauge, and the lesion sizes were expressed as the difference between the thickness of infected and non-infected ear. On day 41 animals were sacrificed and the fluorescence measured (485 - 528 nm) and parasite load quantified using a limiting dilution assay (LDA).

Data on lesion progression were analysed for statistical significance by using the Dunnett test as part of the one-way ANOVA (GraphPad Prism 8 software). A result was considered significant at * $P \leq 0.05$, ** $P \leq 0.01$, *** $P \leq 0.001$, **** $P \leq 0.0001$.

4.2.3.5. High performance thin layer chromatography (HPTLC) based *Lmj*IPCS assay

The following protocol was adapted from a literature procedure and carried out under non- sterile conditions.[11]

Stock 1: Dry PI (1 mM, 30 µL) in a LoBind Eppendorf tube using a vacuum concentrator. To the dried PI, phosphate buffer (71.4 mM , pH 7.0, 105 µL), CHAPS (3 mM, 30 µL) and NBD- C6-ceramide **80** (200 µM, 1.7 µL) was added. The solution was mixed by vortex and stored on ice.

Stock 2: Phosphate buffer (71.4 mM , pH 7.0, 105 µL), CHAPS (3 mM, 30 µL), storage buffer (6 µL) and microsomal membranes (1.5 µL) were combined in a LoBind Eppendorf tube.

Stock 2 (23.75 µL) was added to n LoBind Eppendorf tubes (where n is the number of test compounds + controls), followed by the addition test compounds in DMSO (5 mM, 1 µL). After pre-incubation at 30 °C for 20 min, the reaction was started by the addition of stock 1 (23.75 µL) to each tube and incubation at 30 °C for 30 min. The reaction mixtures were quenched with $CHCl_3$:MeOH:H_2O (10:10:3, 150 µL).

The mixtures were centrifuged to separate phases, the organic layer was removed and dried using a vacuum concentrator. The residue was re-suspended in $CHCl_3$:MeOH:H_2O (10:10:3, 20 µL) and loaded (3 x 3 µL) onto HPTLC plates (silica gel 60 F254). This was run using the solvent system $CHCl_3$:MeOH:0.25% KCl(aq) (55:45:10) and the *Rf* values for the excess NBD-C6-ceramide **80** and the product NBD-C6-IPC **81** were 0.96 and 0.57 respectively. Product quantification was carried out using a fluorescence plate reader (Ex473/Em520).

4.2.3.6. Radioligand Binding Assay (Supplemental Figure S3)

These assays were run by Eurofins under contract to LifeArc FR095-0011887. A representative protocol describing the human histamine H2 receptor (antagonist radioligand) assay is shown below.[12]

Cell membrane homogenates (24 µg protein) are incubated for 120 min at 22°C with 0.075 nM [125I]APT in the absence or presence of the test compound in a buffer containing 50 mM Na2HPO4/KH2PO4 (pH 7.4) and 0.05% BSA. Nonspecific binding is determined in the presence of 100 µM tiotidine. Following incubation, the samples are filtered rapidly under vacuum through glass fiber filters (GF/B, Packard) presoaked with 0.3% PEI and rinsed several times with ice-cold 50 mM Tris-HCl buffer using a 96-sample cell harvester (Unifilter, Packard). The filters are dried then counted for radioactivity in a scintillation counter (Topcount, Packard) using a scintillation cocktail (Microscint 0, Packard). The results are expressed as a percent inhibition of the control radioligand specific binding. The standard reference compound is cimetidine, which is tested in each experiment at several concentrations to obtain a competition curve from which its IC_{50} is calculated.

5. References

1. J. G. M. Mina, R. L. Charlton, E. Alpizar-Sosa, D. O. Escrivani, C. Brown, A. Alqaisi, M. P. G. Borsodi, C. P. Figueiredo, E. V. de Lima, E. A. Dickie, W. B. Wei, R. Coutinho-Silva, A. Merritt, T. K. Smith, M. P. Barrett, B. Rossi-Bergmann, P. W. Denny and P. G. Steel, *ACS Infectious Diseases*, 2021, **7**, 47-63.
2. Y. X. Yang, Y. Liu, L. Zhang, Y. E. Jia, P. Wang, F. F. Zhuo, X. T. An and C. S. Da, *J. Org. Chem.*, 2014, **79**, 10696-10702.
3. A. M. Fournier, R. A. Brown, W. Farnaby, H. Miyatake-Ondozabal and J. Clayden, *Org. Lett.*, 2010, **12**, 2222-2225.
4. V. Terrasson, S. Marque, M. Georgy, J. M. Campagne and D. Prim, *Adv. Synth. Catal.*, 2006, **348**, 2063-2067.
5. A. G. M. Barrett, B. T. Hopkins, A. C. Love and L. Tedeschi, *Org. Lett.*, 2004, **6**, 835-837.
6. E. J. Trybulski, R. H. Kramss, R. M. Mangano and A. Rusinko, *J. Med. Chem.*, 1990, **33**, 3190-3198.
7. R. Csuk, A. Barthel and D. Ströhl, *Zeitschrift Fur Naturforschung Section B-a Journal of Chemical Sciences*, 2011, **66**, 95-97.
8. K. M. Darwish, I. Salama, S. Mostafa, M. S. Gomaa, E. S. Khafagy and M. A. Helal, *Bioorg. Med. Chem. Lett.*, 2018, **28**, 1595-1602.
9. National Research Council (US) Committee for the Update of the Guide for the Care and Use of Laboratory Animals, *Guide for the Care and Use of Laboratory Animals,* , National Academies Press (US), Washington (DC),, 8th Ed edn., 2011.
10. F. M. Marim, T. N. Silveira, D. S. Lima and D. S. Zamboni, *PLoS One*, 2010, **5**, 0015263.
11. J. G. Mina, J. A. Mosely, H. Z. Ali, H. Shams-Eldin, R. T. Schwarz, P. G. Steel and P. W. Denny, *Int. J. Biochem. Cell Biol.*, 2010, **42**, 1553-1561.
12. R. Leurs, M. J. Smit, W. Menge and H. Timmerman, *Br. J. Pharmacol.*, 1994, **112**, 847.

6. ^{1}H and ^{13}C NMR Spectra

^{1}H and ^{13}C NMR Spectra for **7**

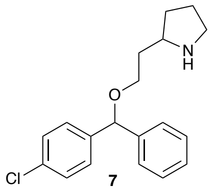

7

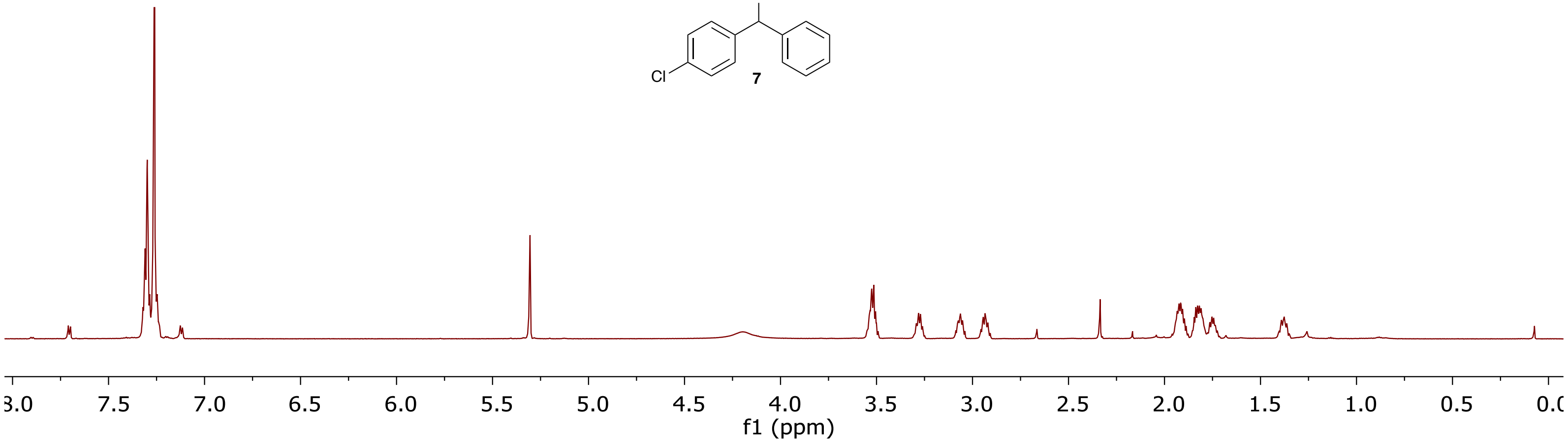

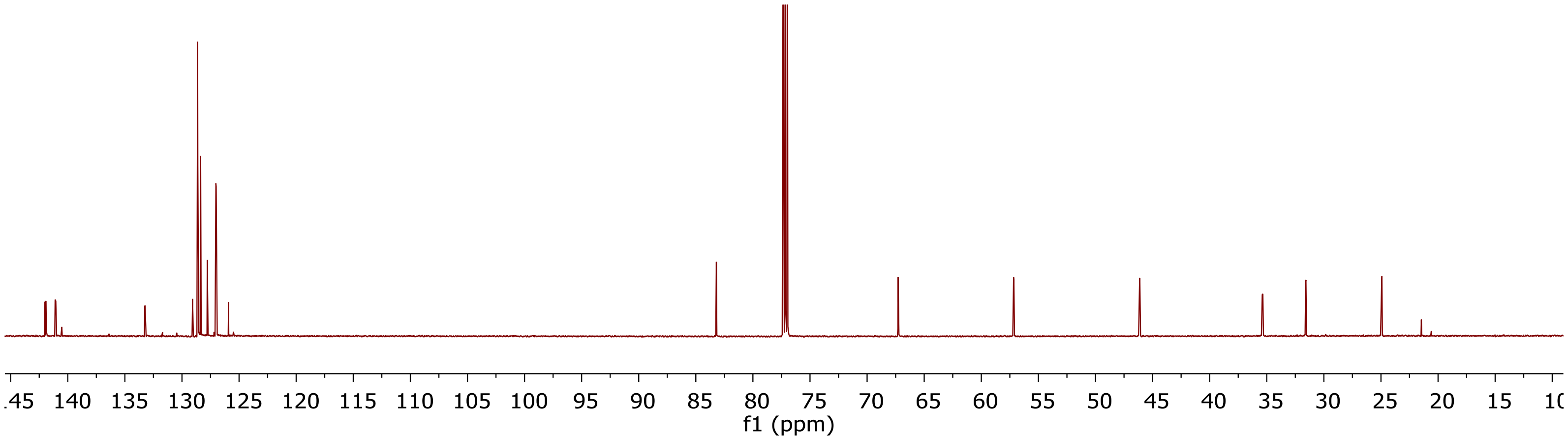

^{1}H and ^{13}C NMR Spectra for **9**

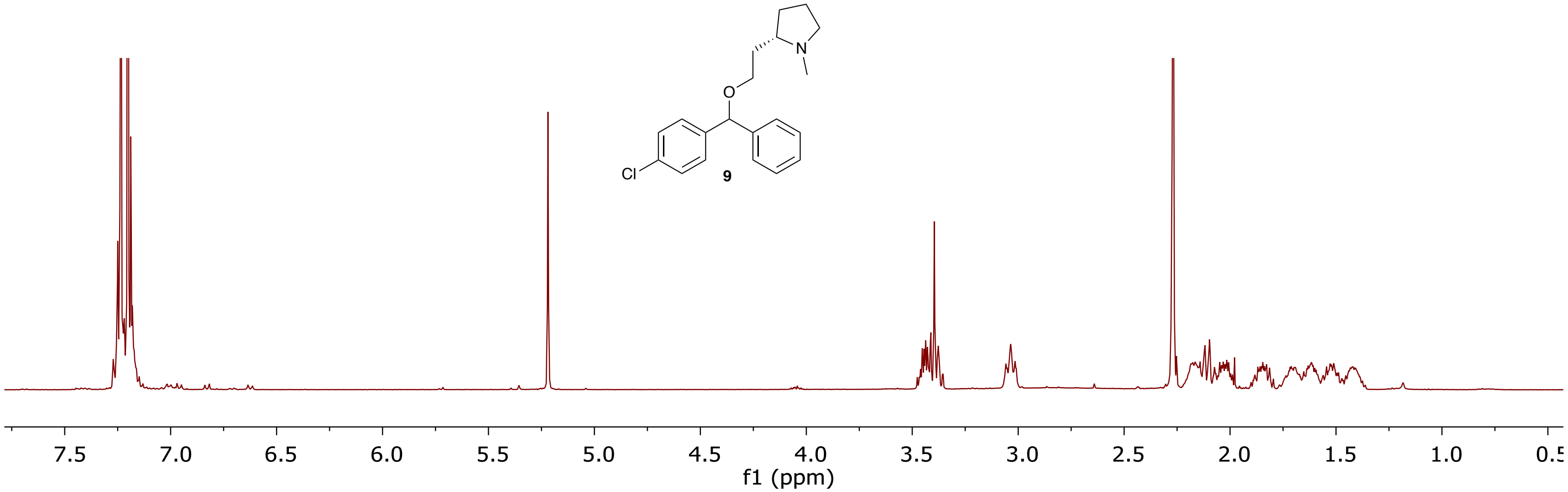

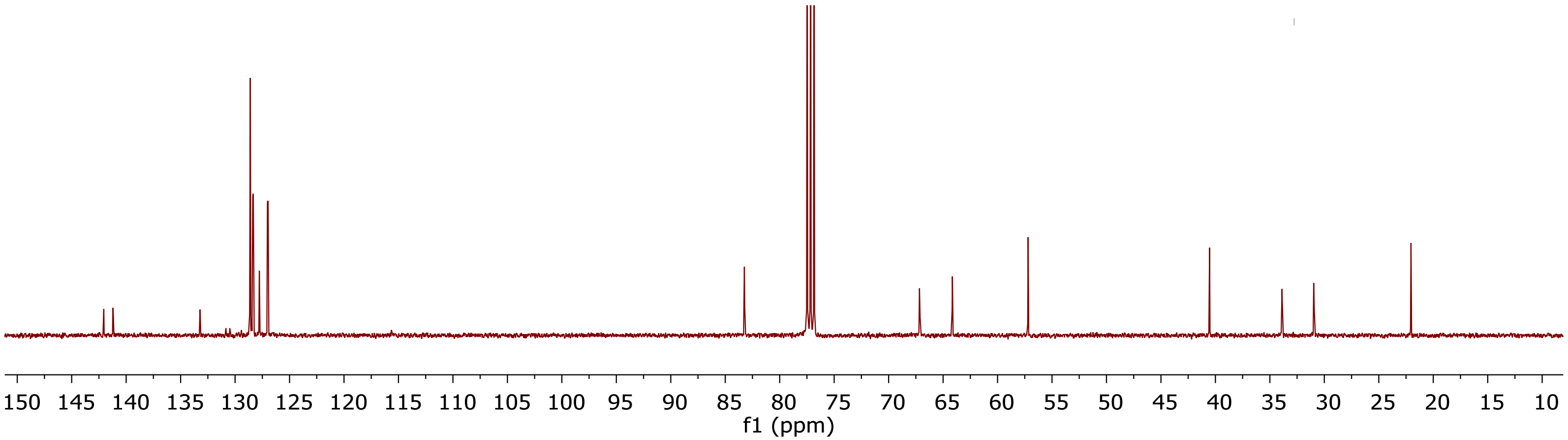

^{1}H and ^{13}C NMR Spectra for **10**

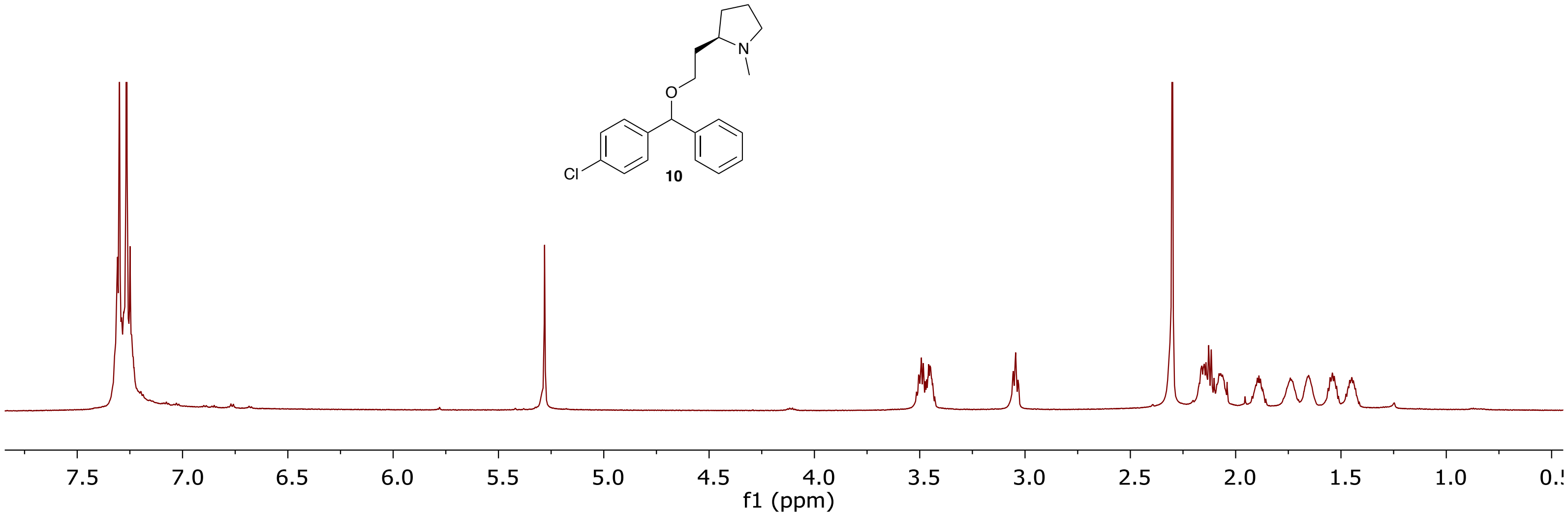

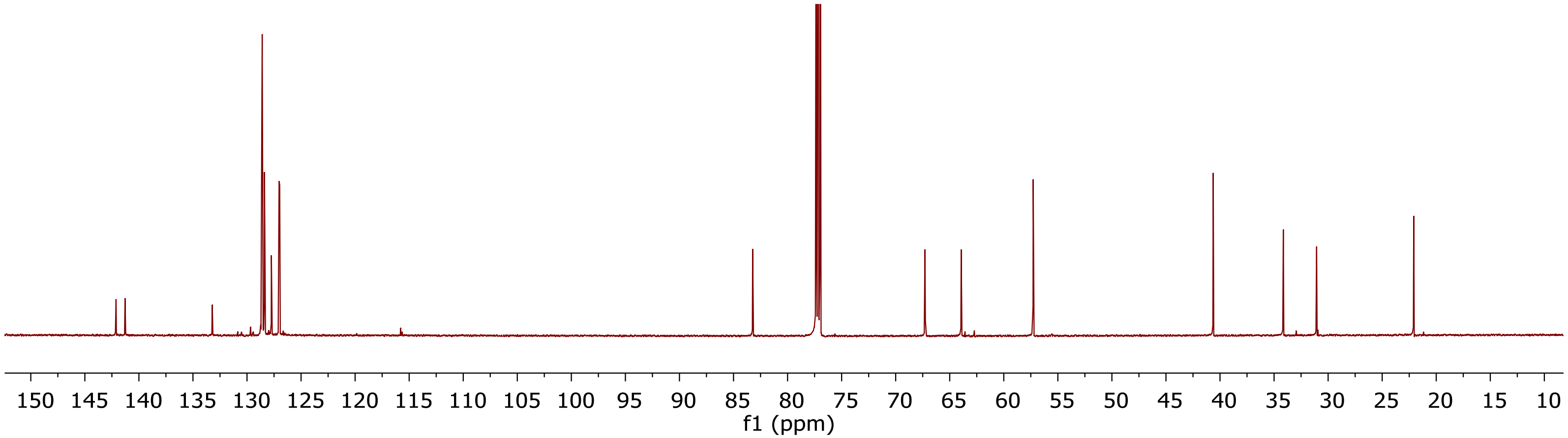

^{1}H and ^{13}C NMR Spectra for **14b**

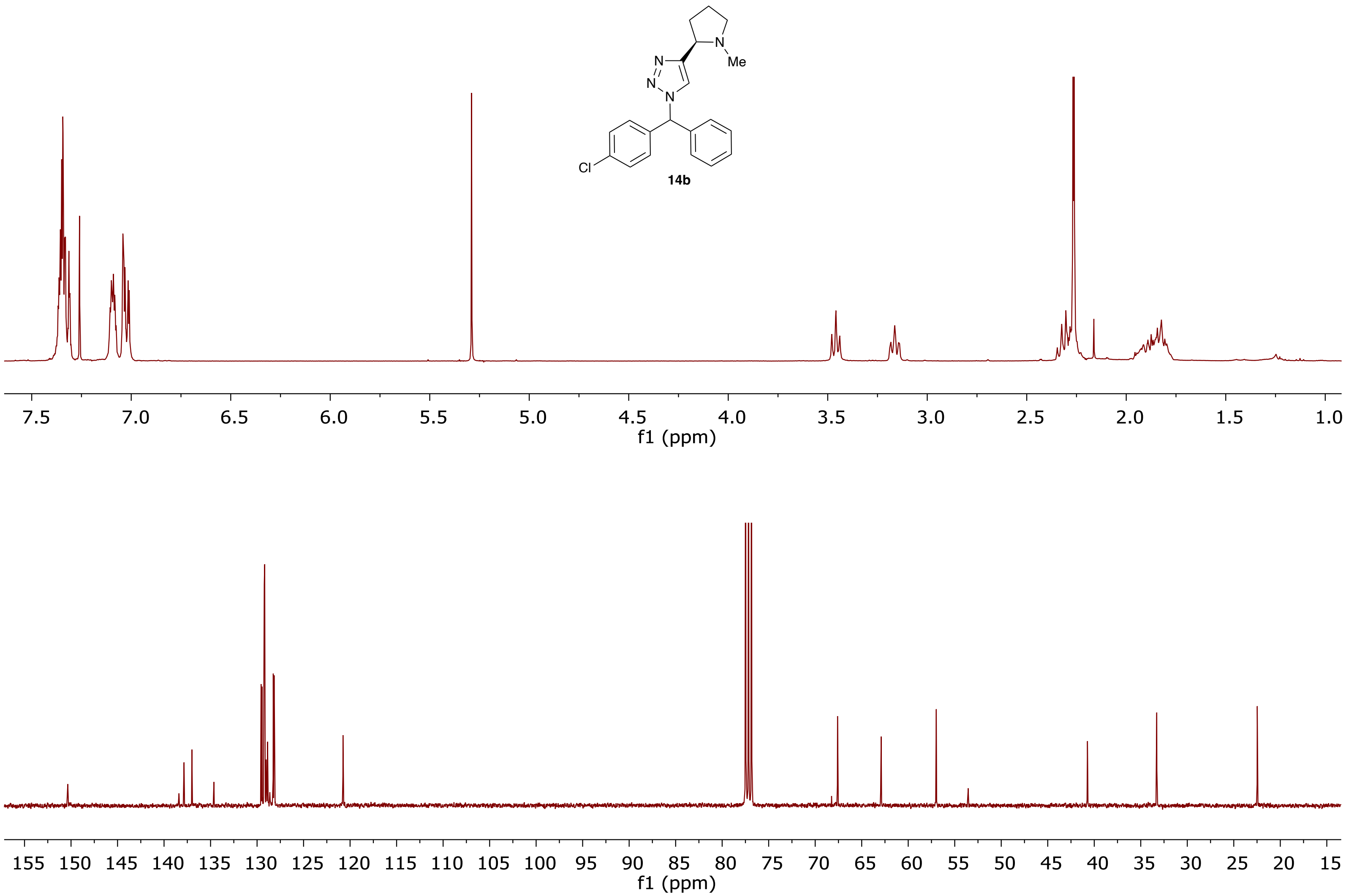

14b

^{1}H and ^{13}C NMR Spectra for **14a**

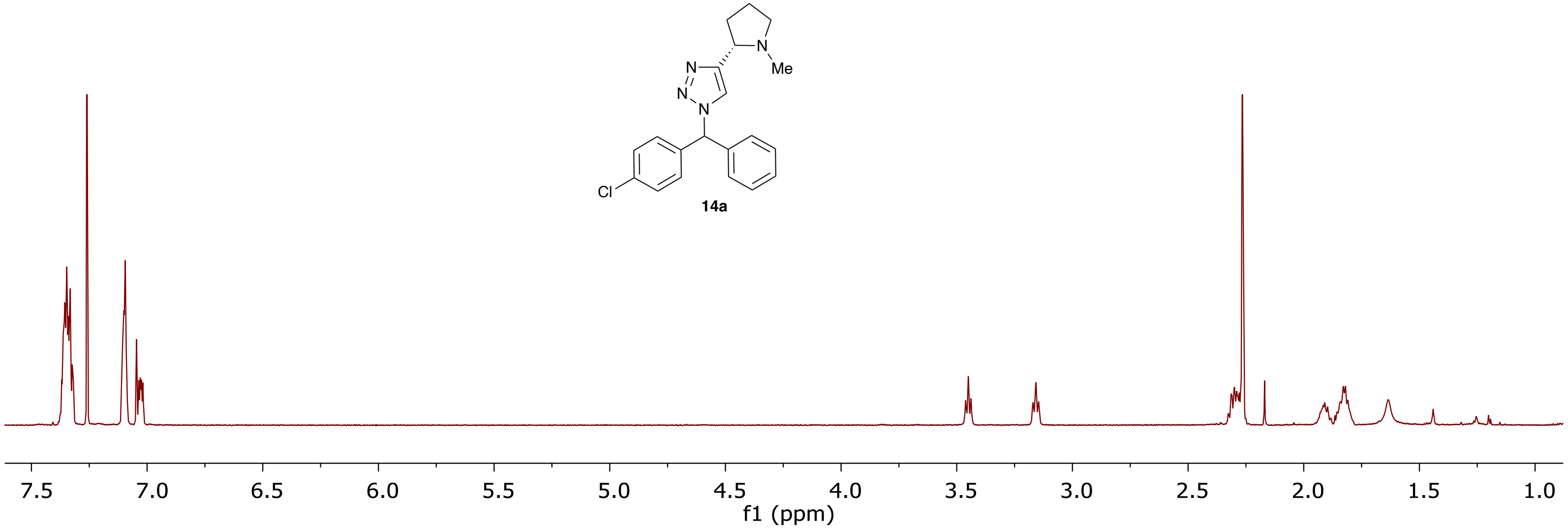

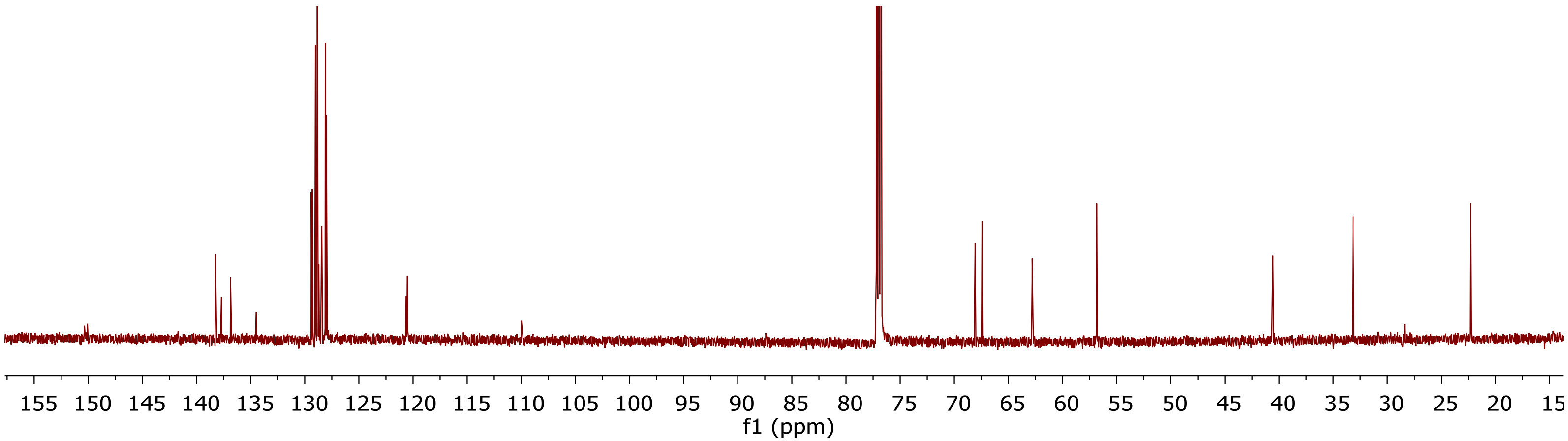

^{1}H and ^{13}C NMR Spectra for **19**

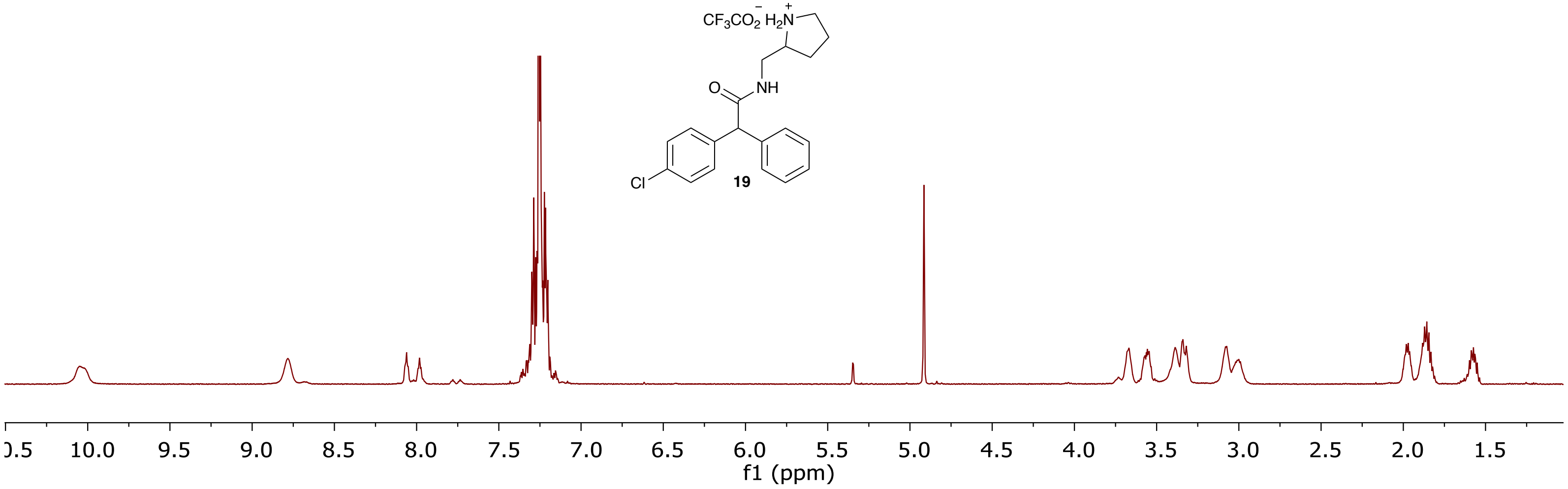

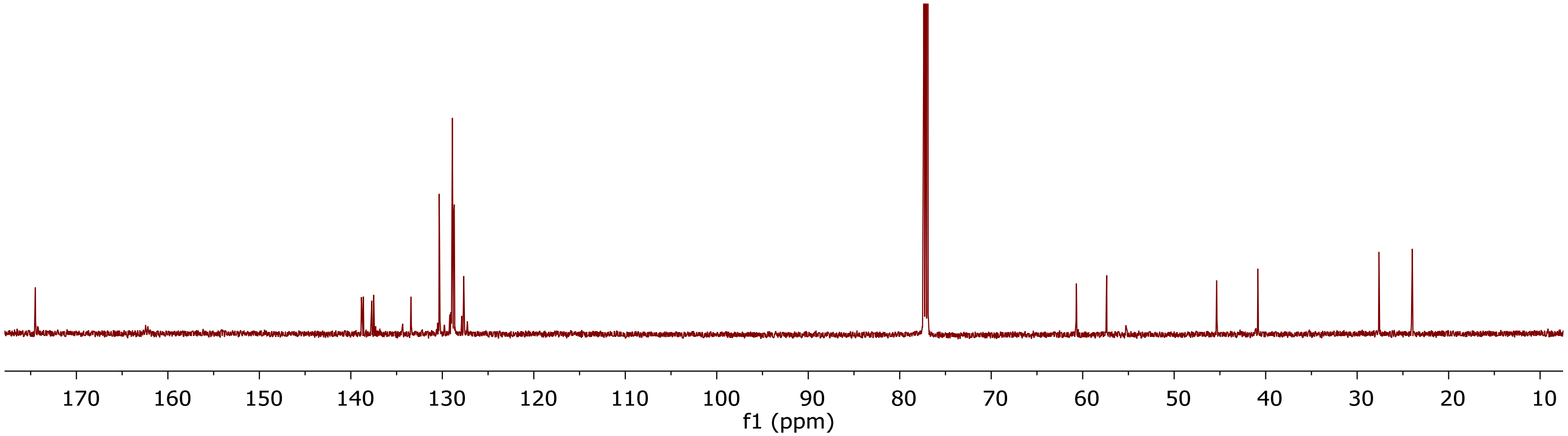

^{1}H and ^{13}C NMR Spectra for **21**

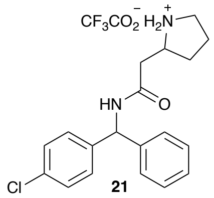

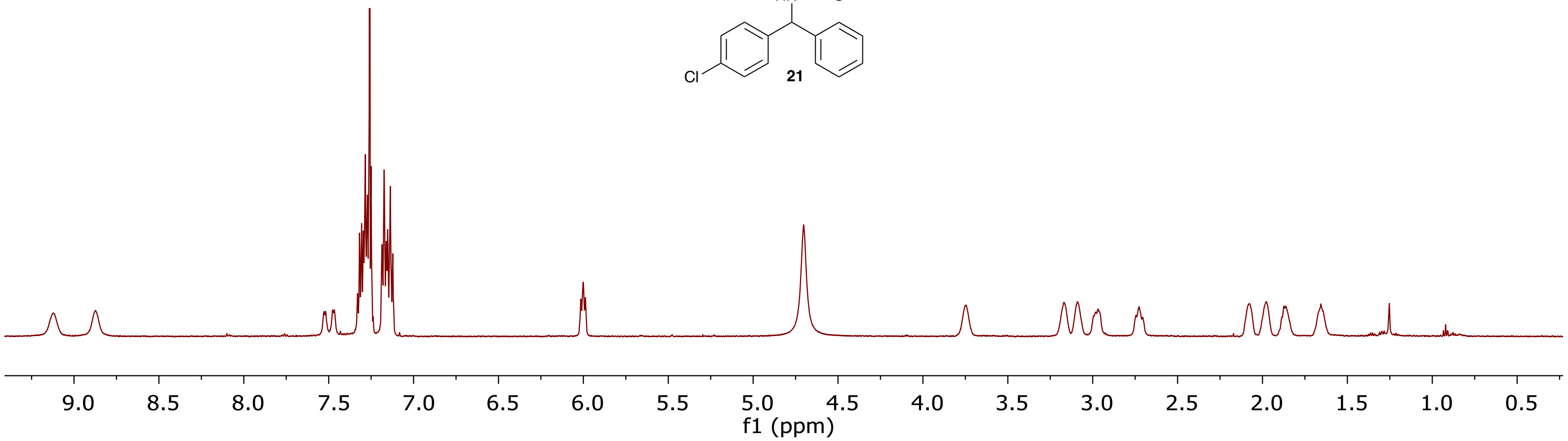

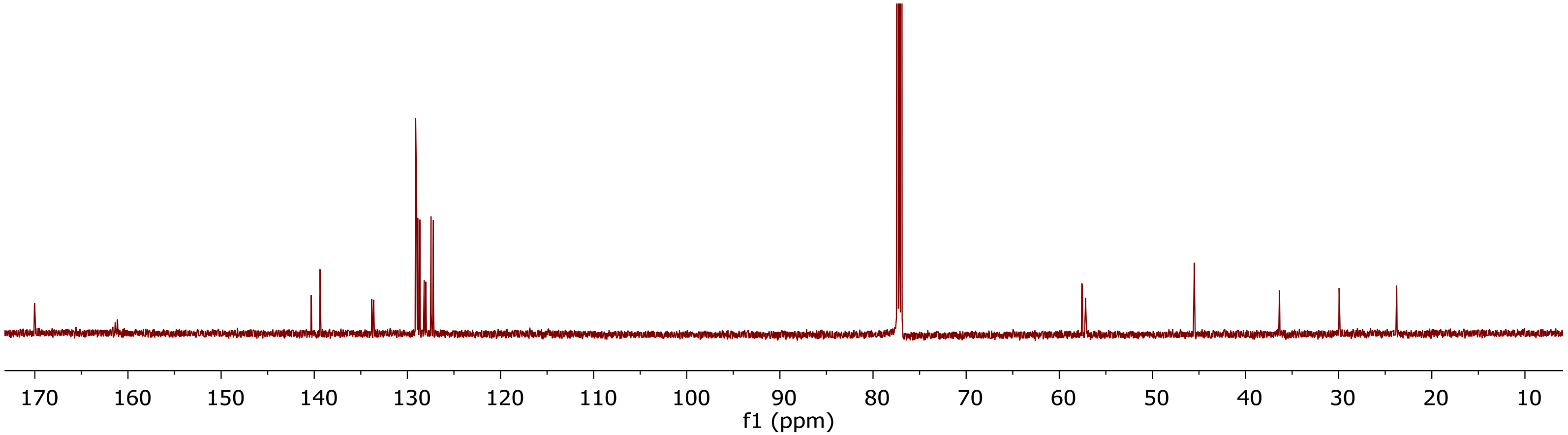

^{1}H and ^{13}C NMR Spectra for **24**

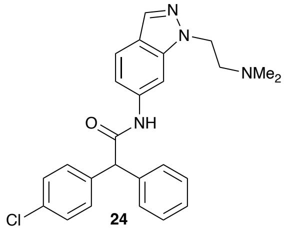

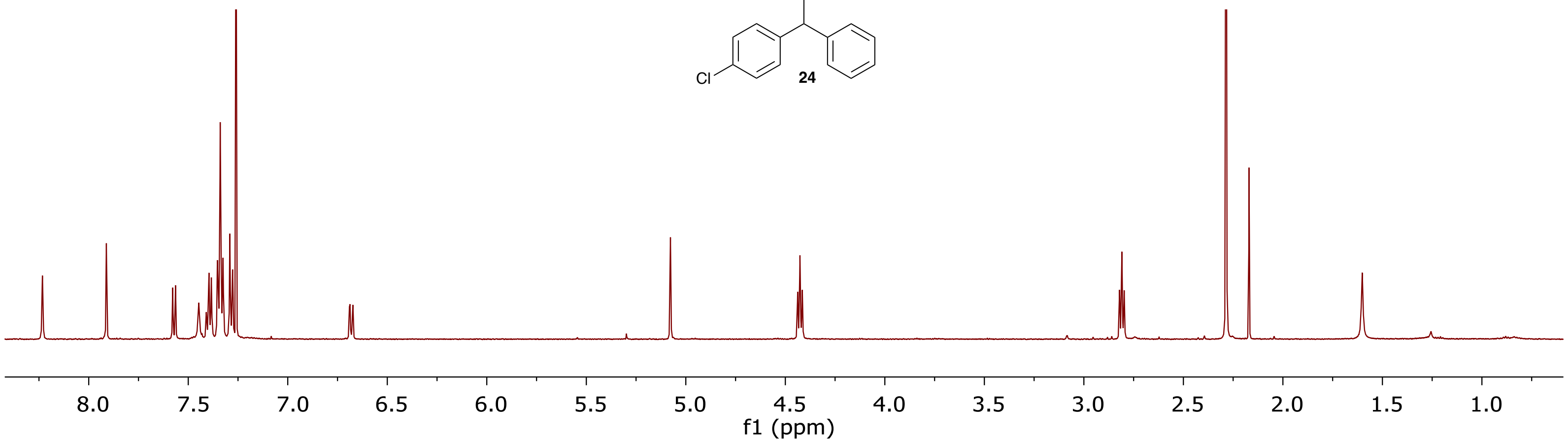

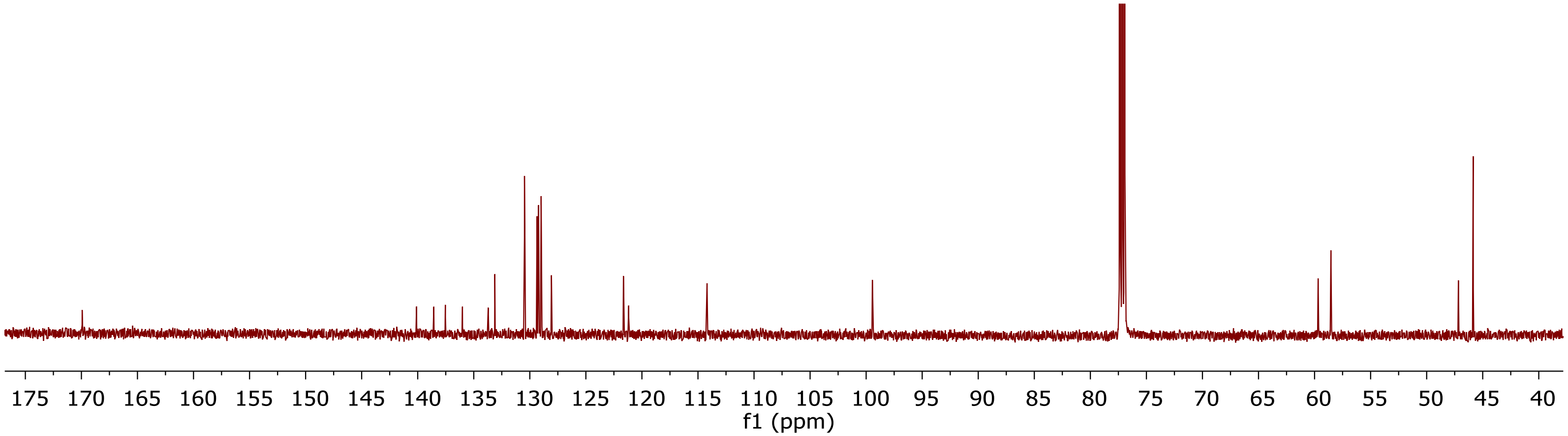

^{1}H and ^{13}C NMR Spectra for **27**

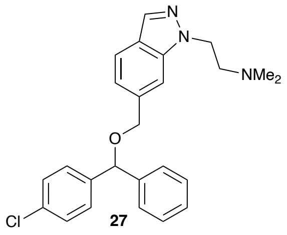

27

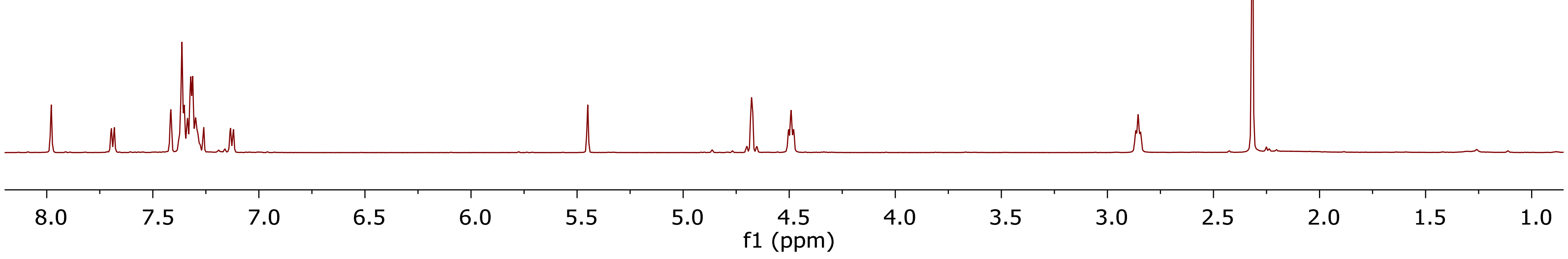

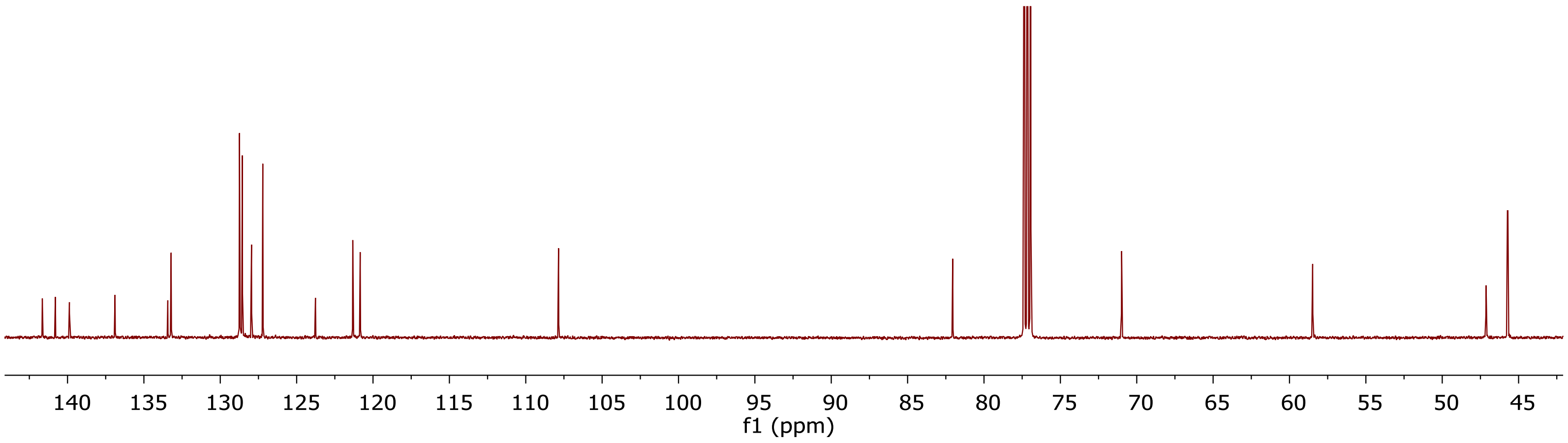

^1H and ^{13}C NMR Spectra for **28**

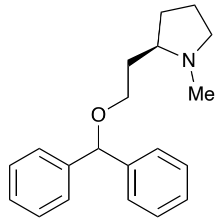

28

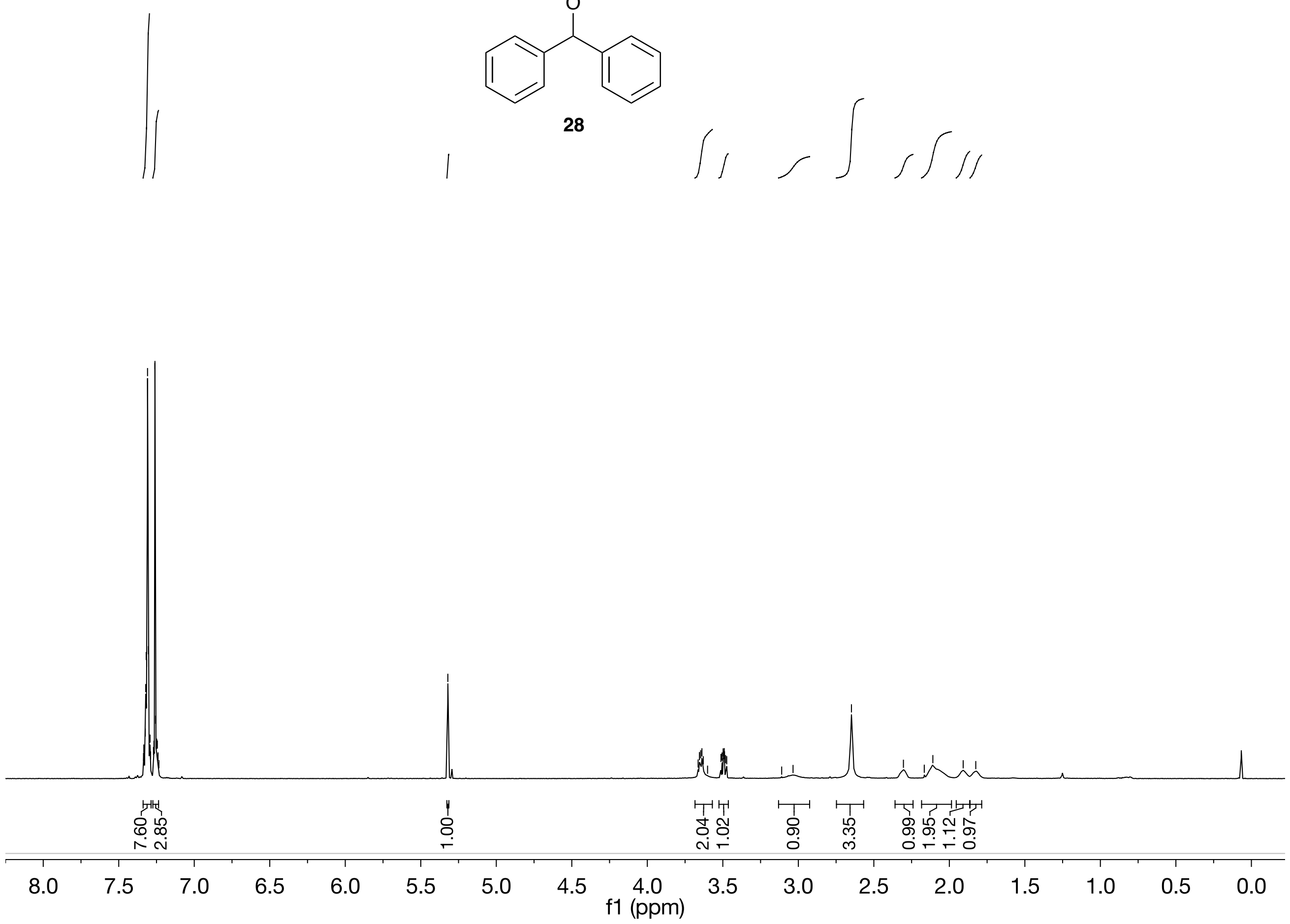

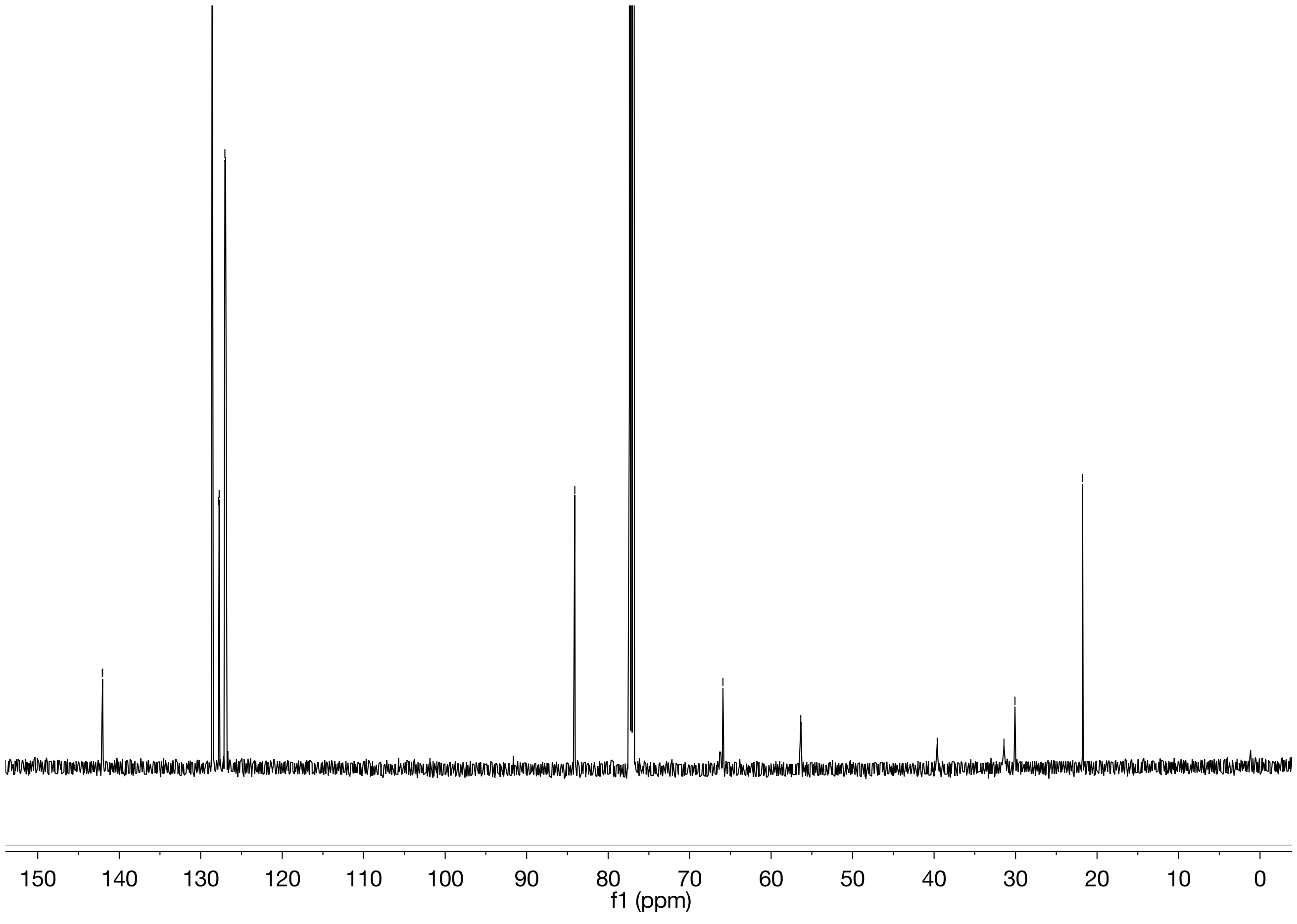

^1H and ^{13}C NMR Spectra for **29**

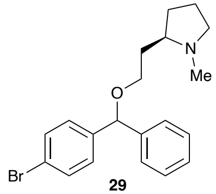

29

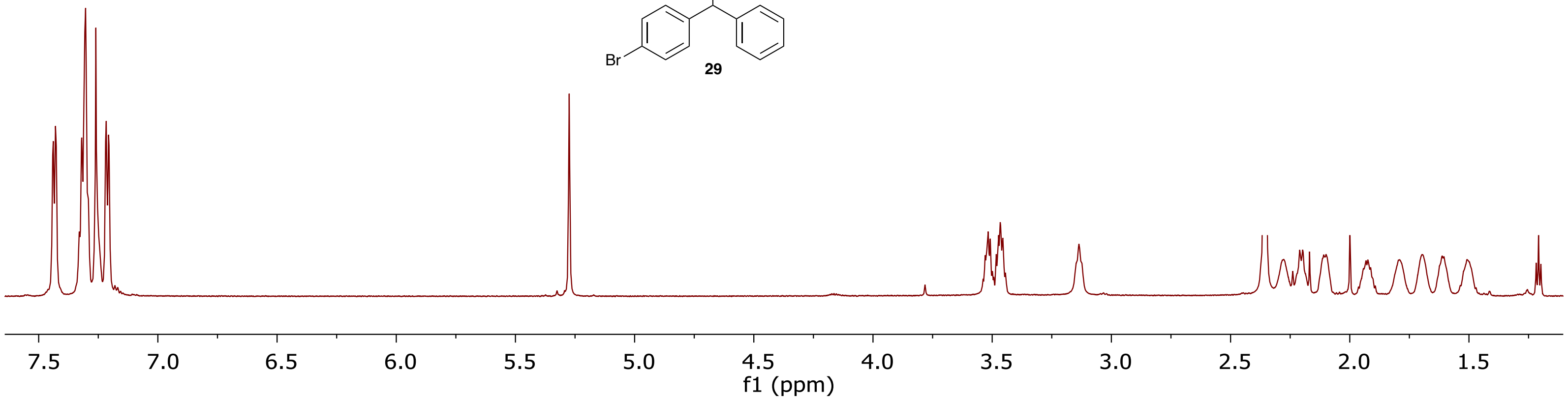

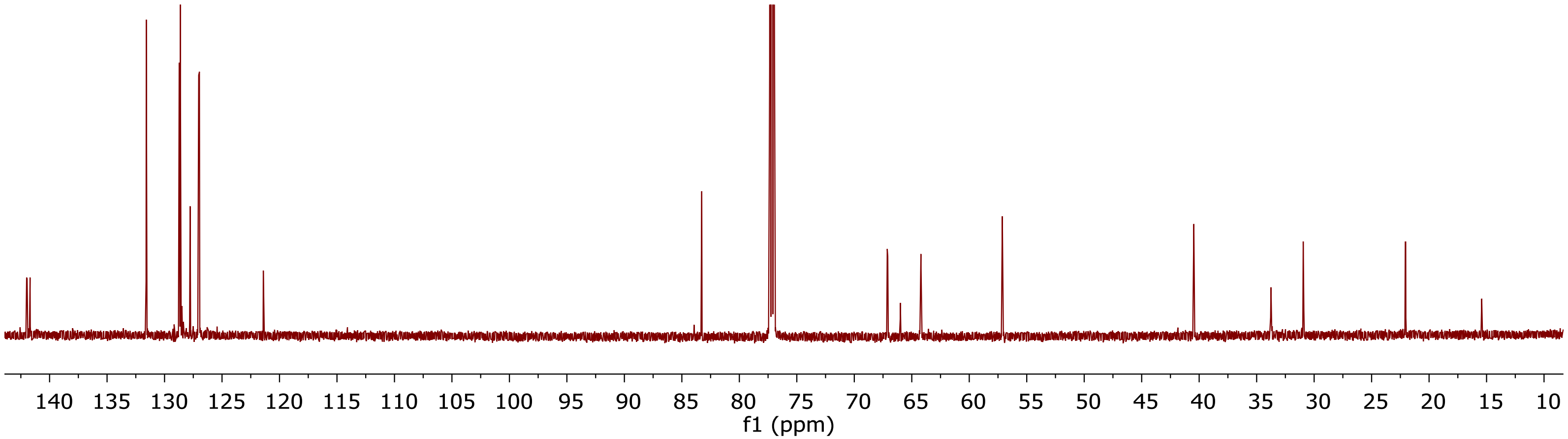

Note: Residual ether peaks observable at δ_H = 1.21 ppm; δ_C = 66.0 and 15.4 ppm

^1H and ^{13}C NMR Spectra for **30**

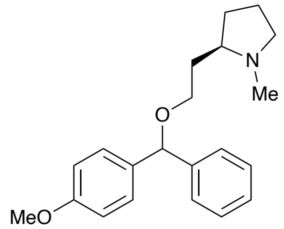

30

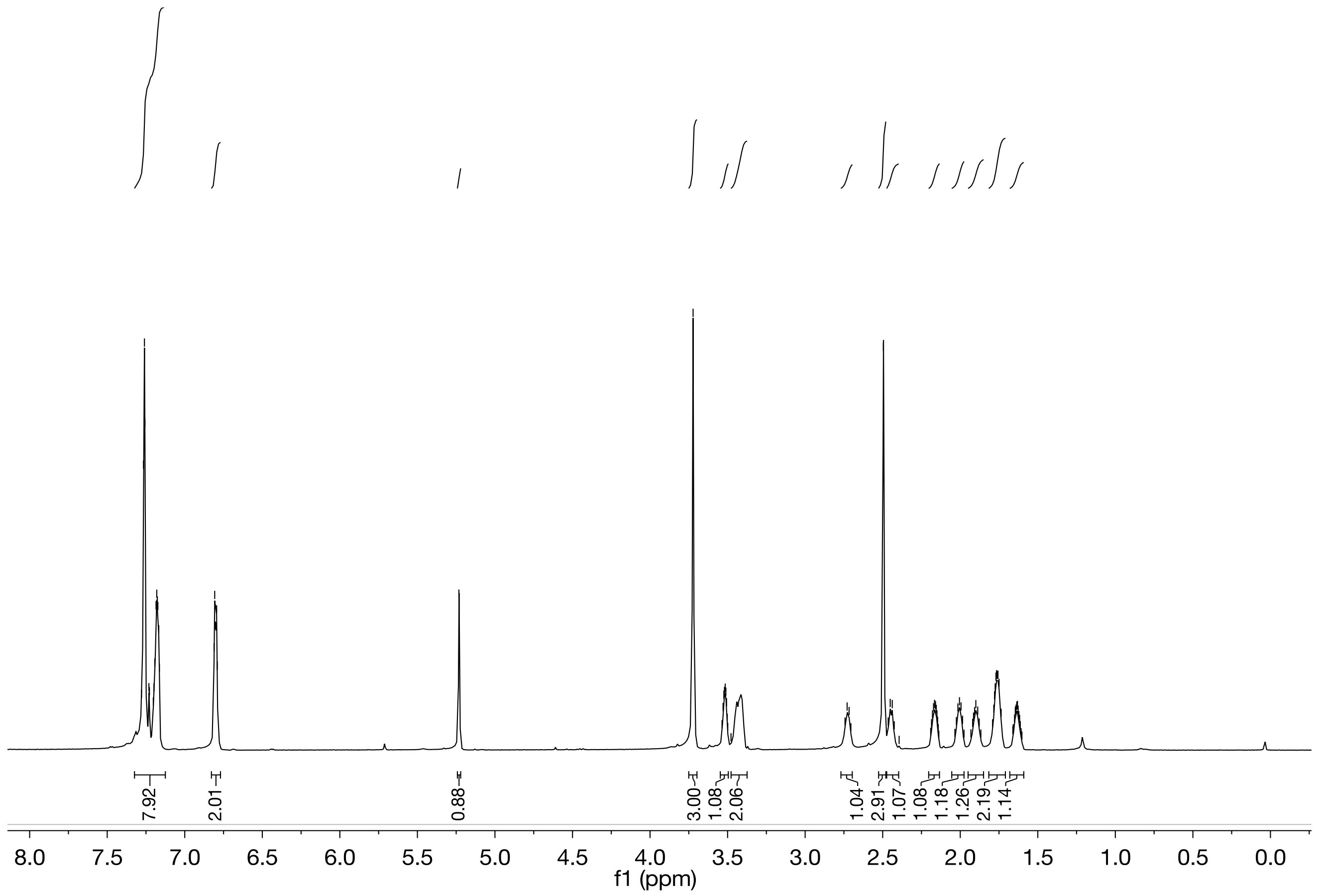

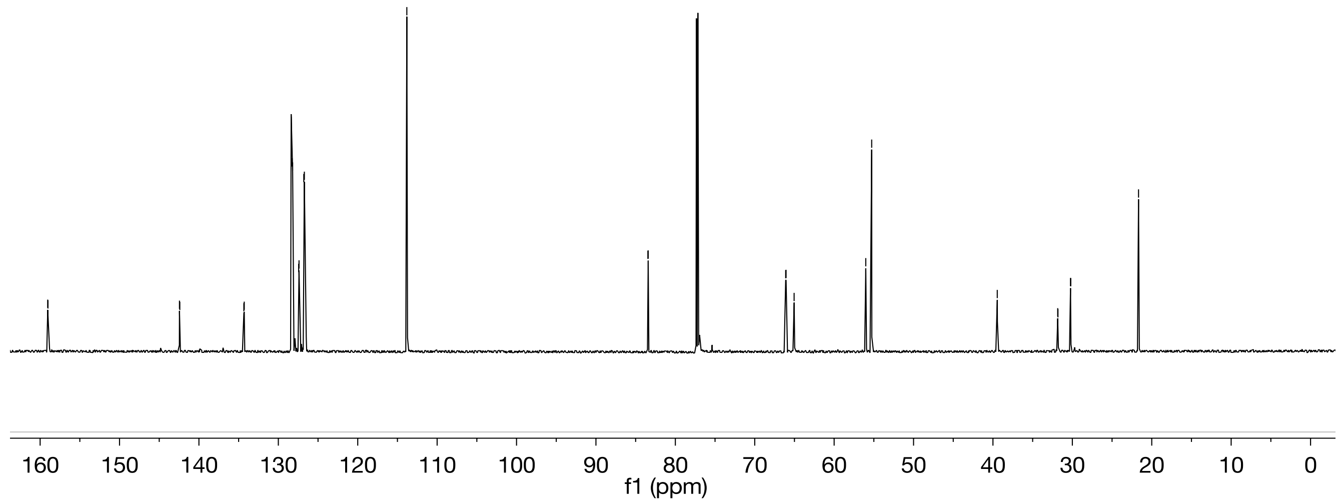

^{1}H and ^{13}C NMR Spectra for **31**

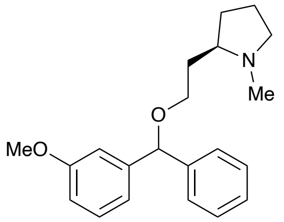

31

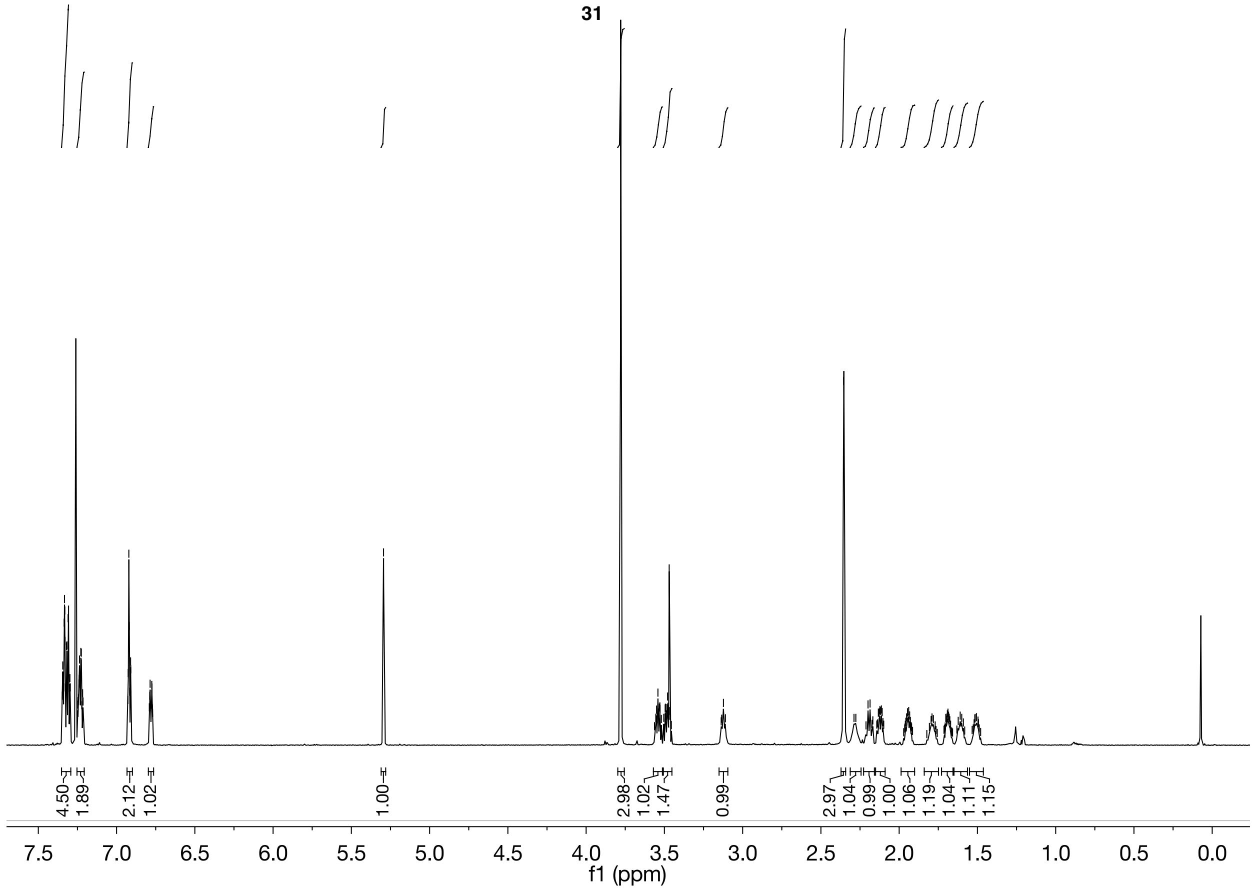

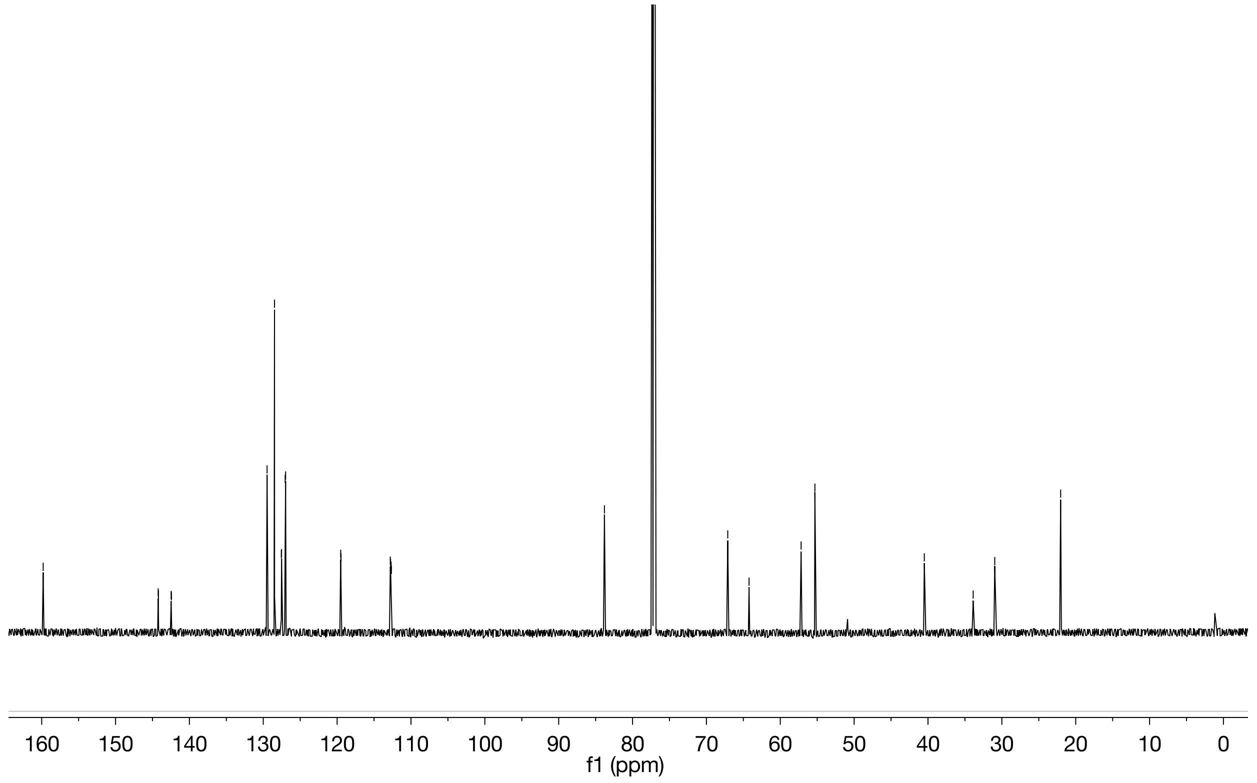

^{1}H and ^{13}C NMR Spectra for **32**

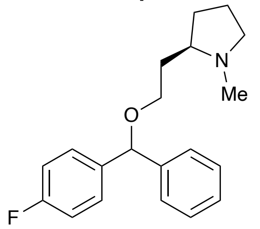

32

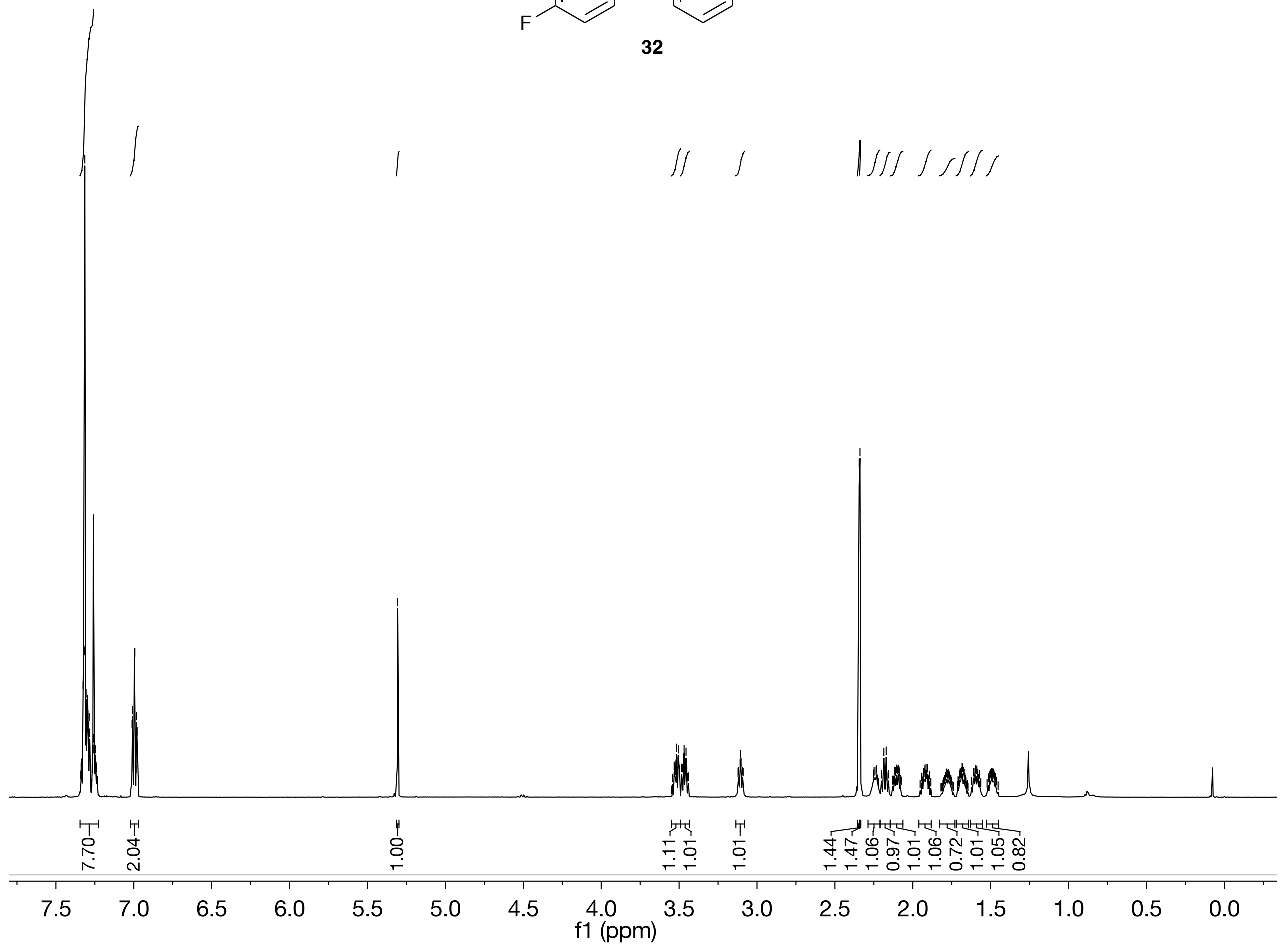

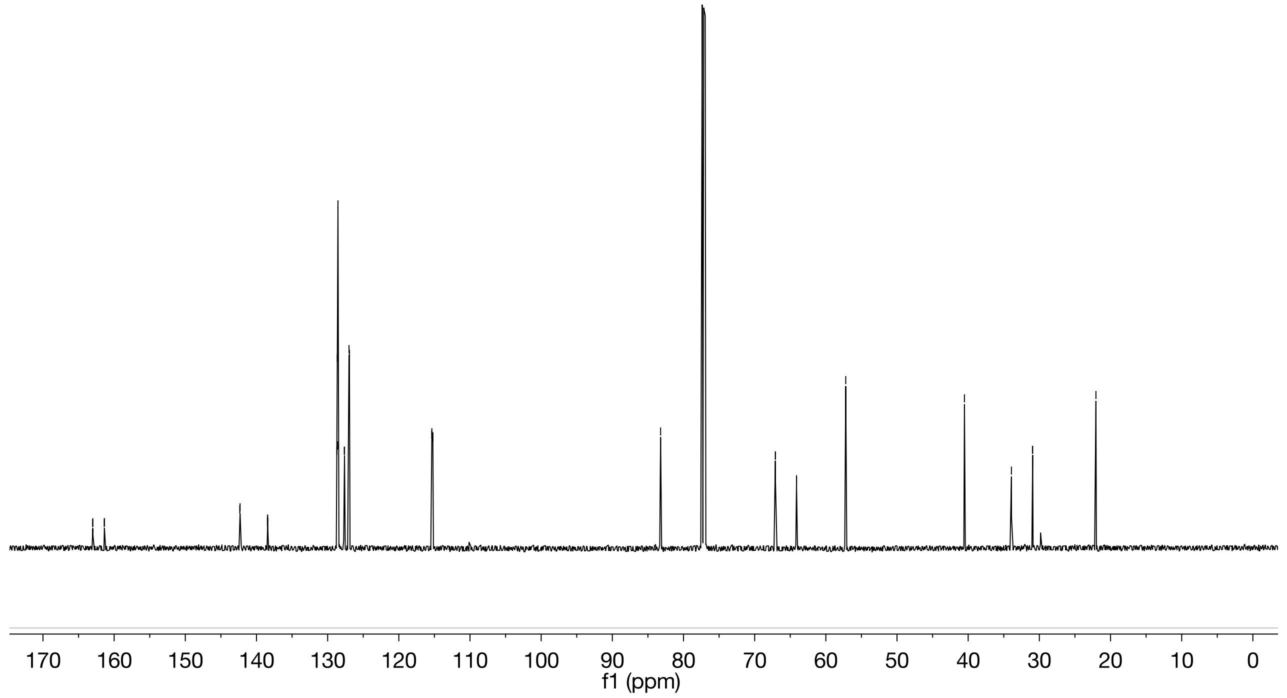

^{1}H and ^{13}C NMR Spectra for **33**

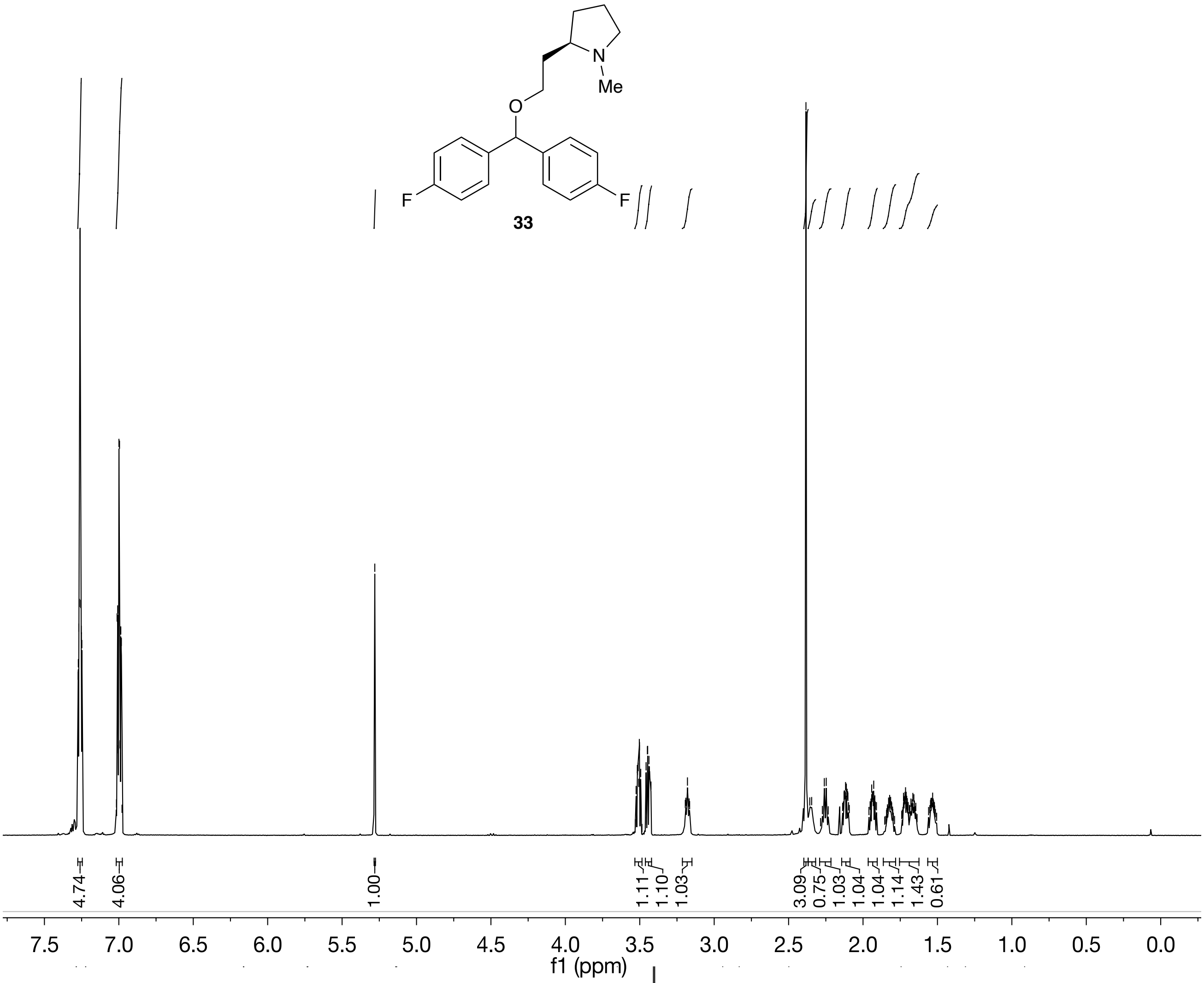

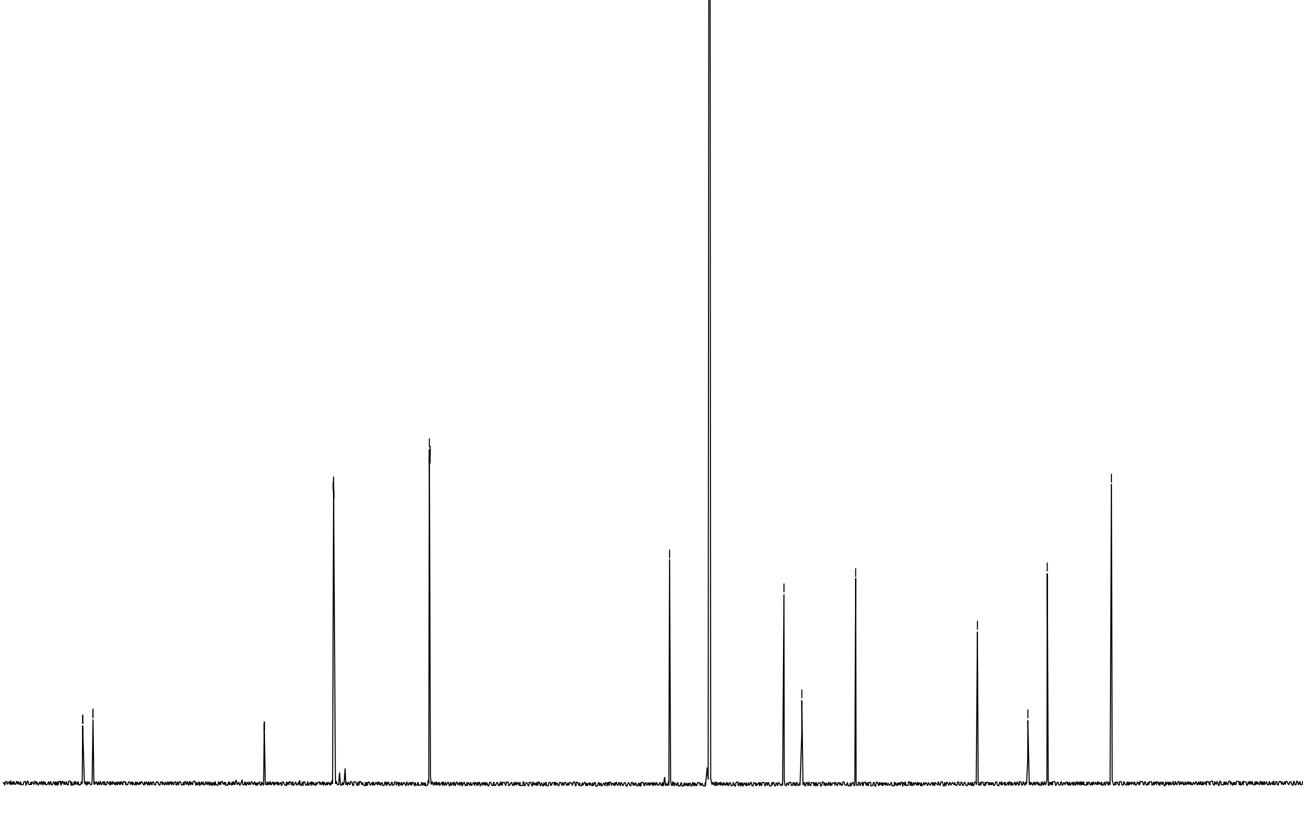

^{1}H and ^{13}C NMR Spectra for **35**

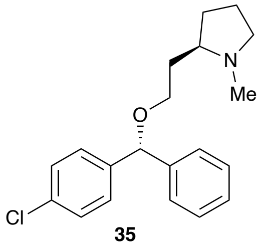

35

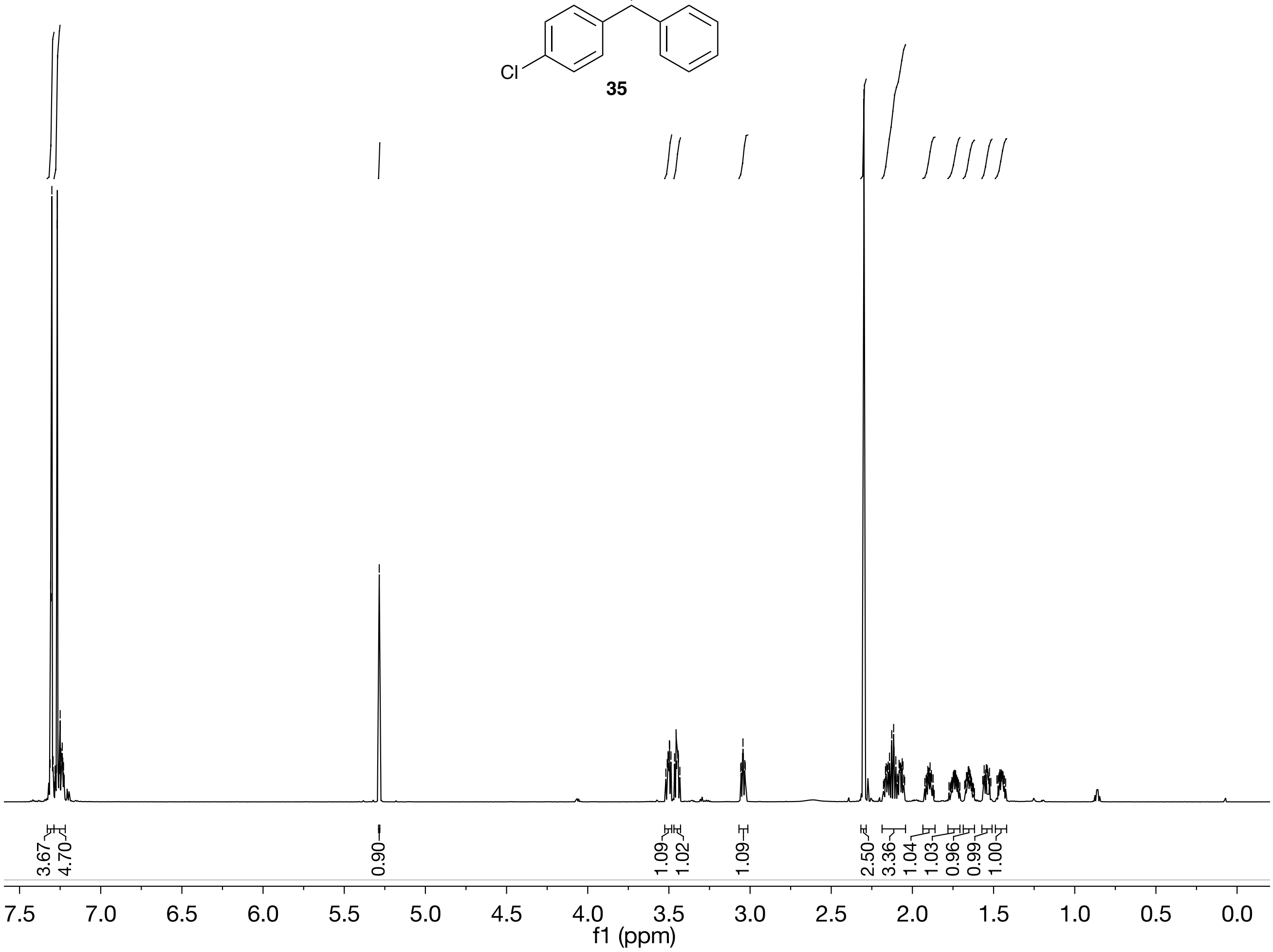

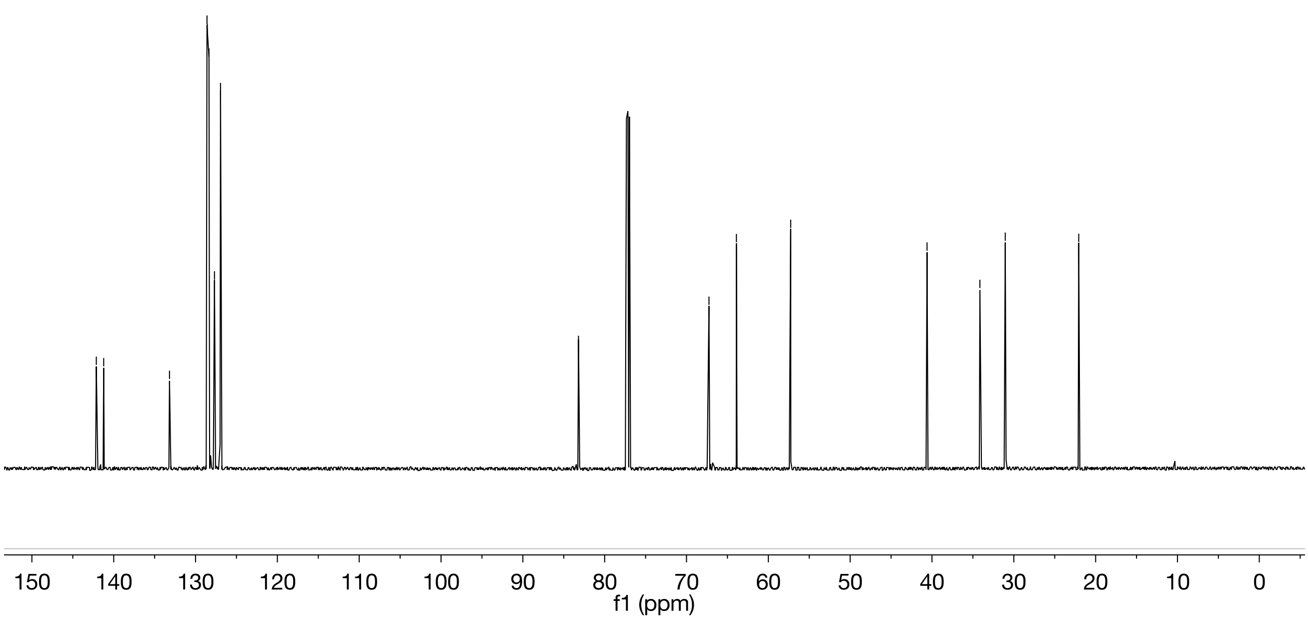

^{1}H and ^{13}C NMR Spectra for **36**

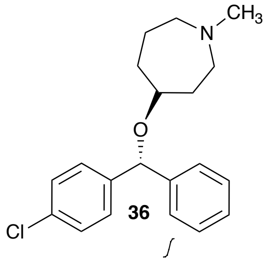

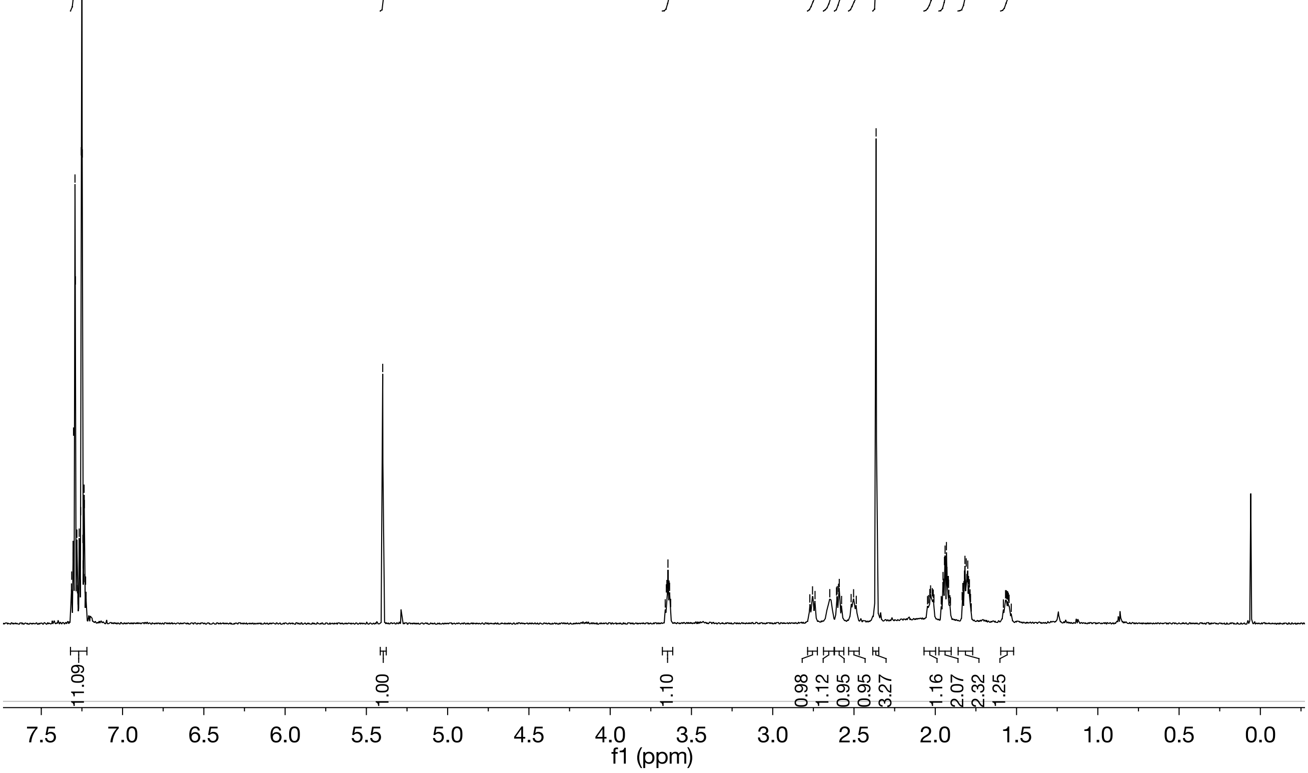

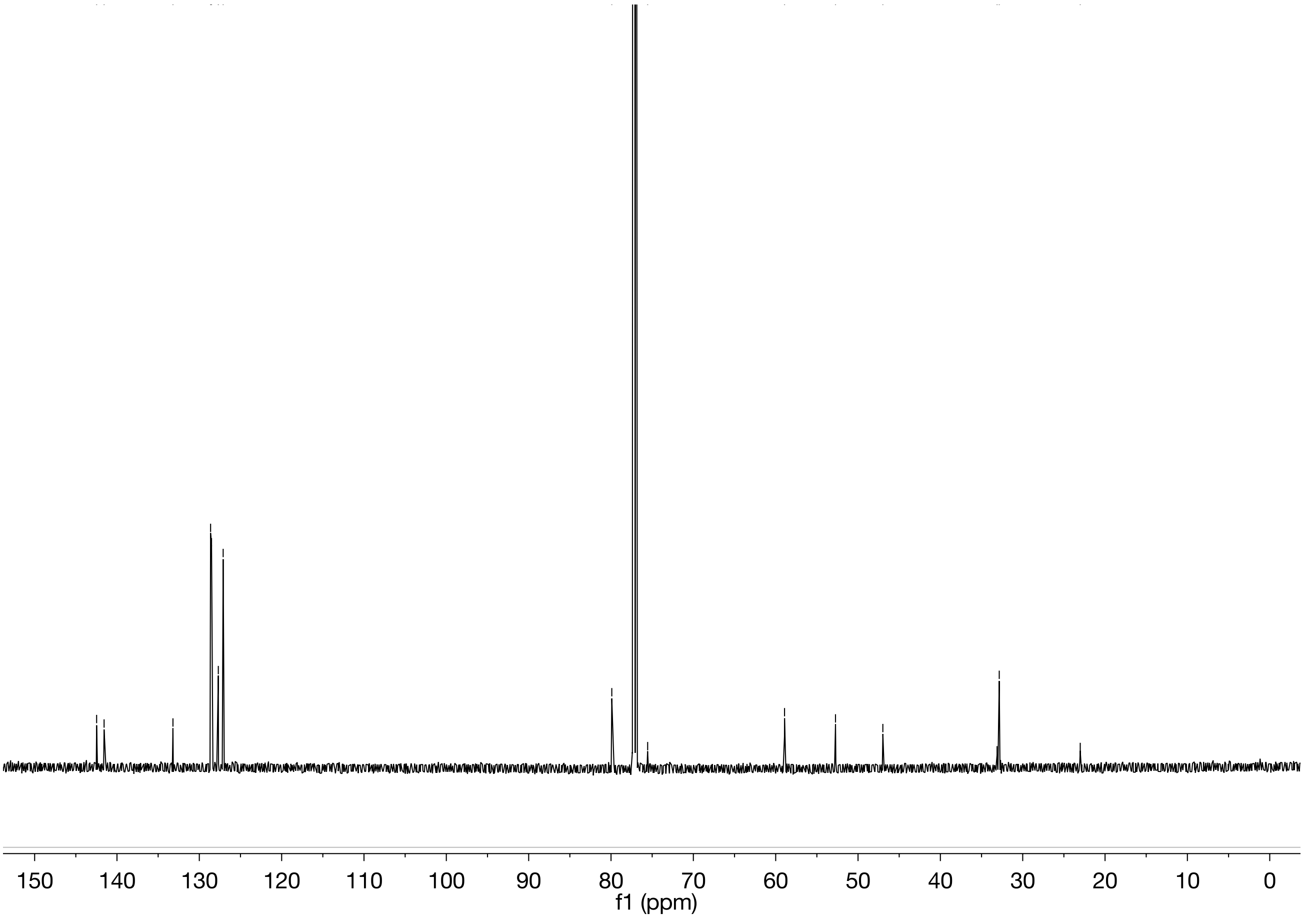

^{1}H and ^{13}C NMR Spectra for **37**

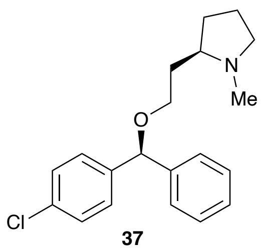

37

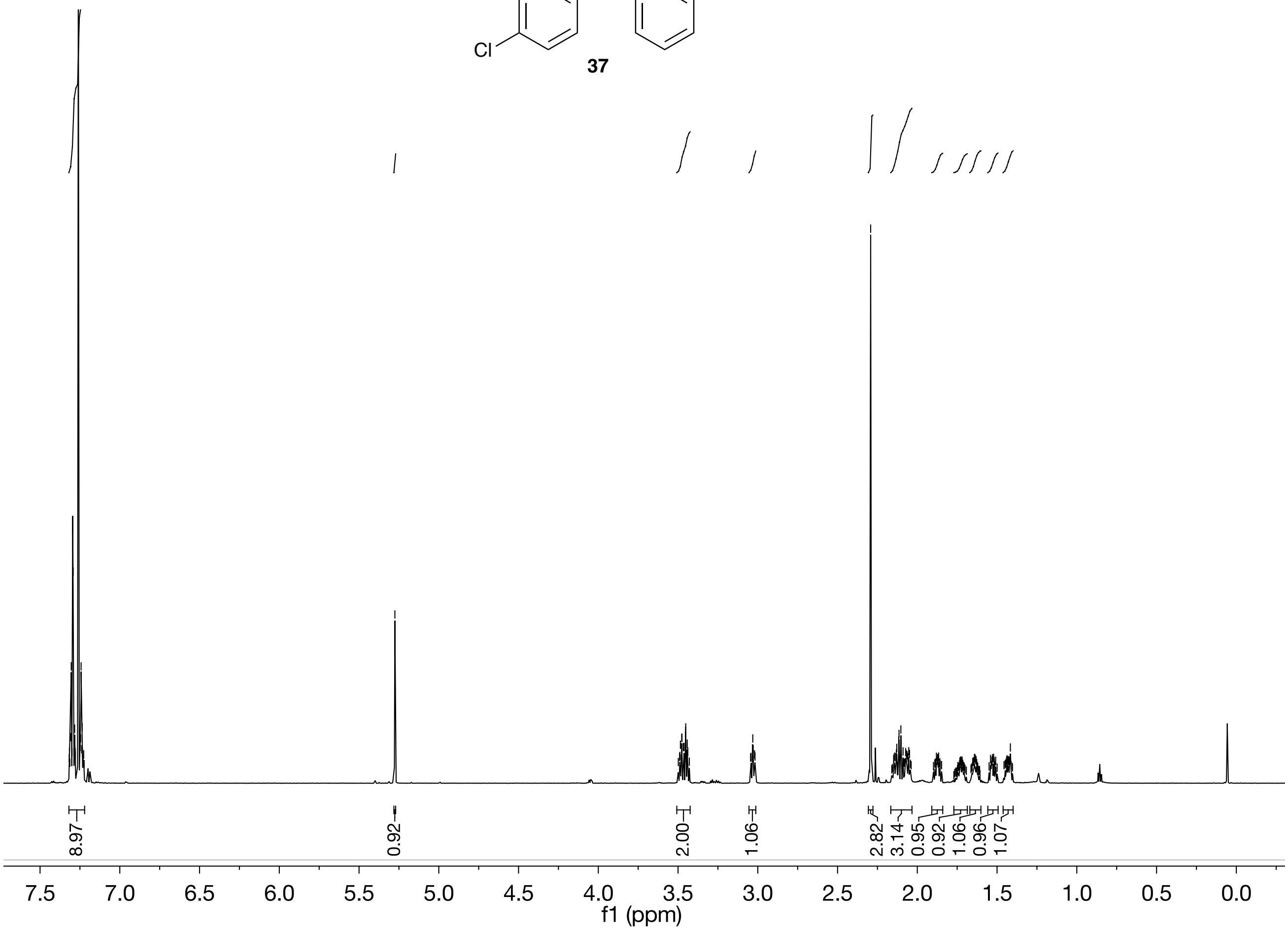

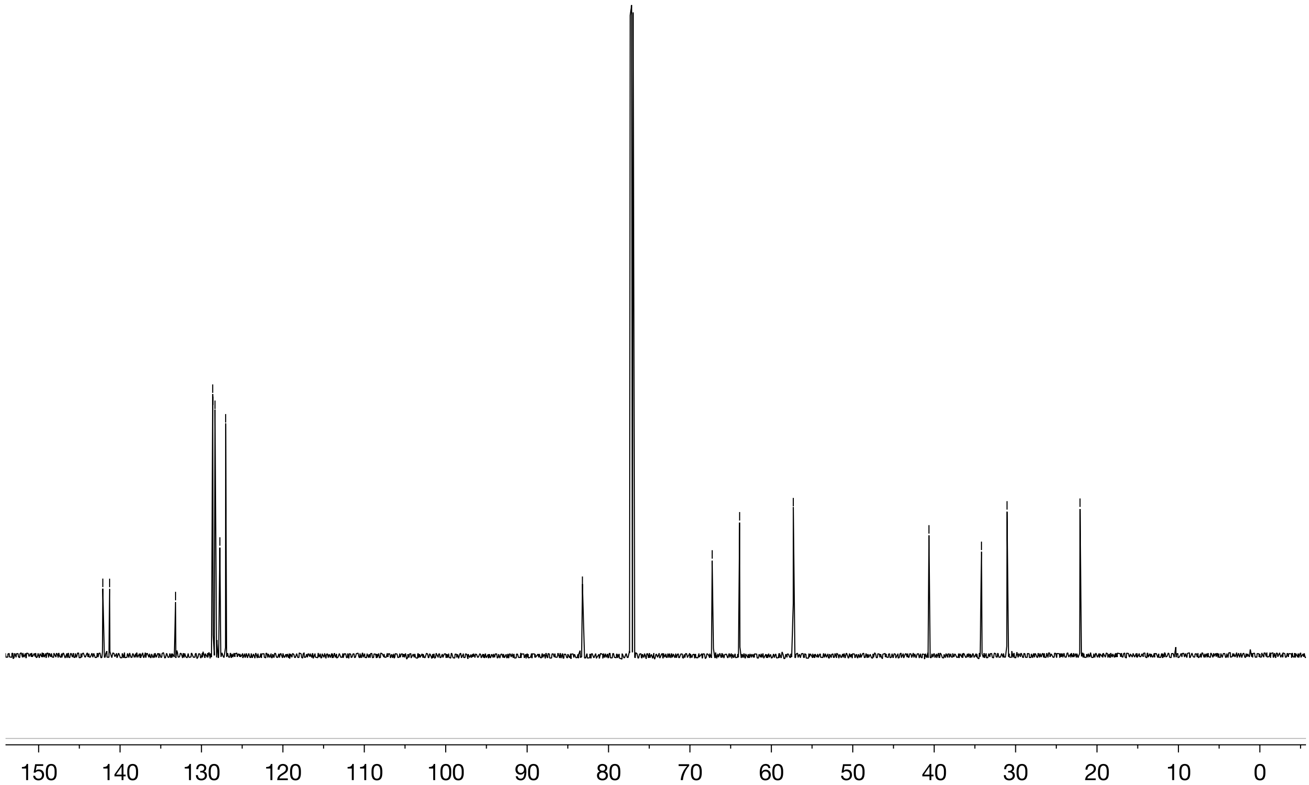

^{1}H and ^{13}C NMR Spectra for **38a**

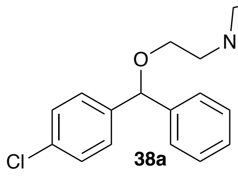

38a

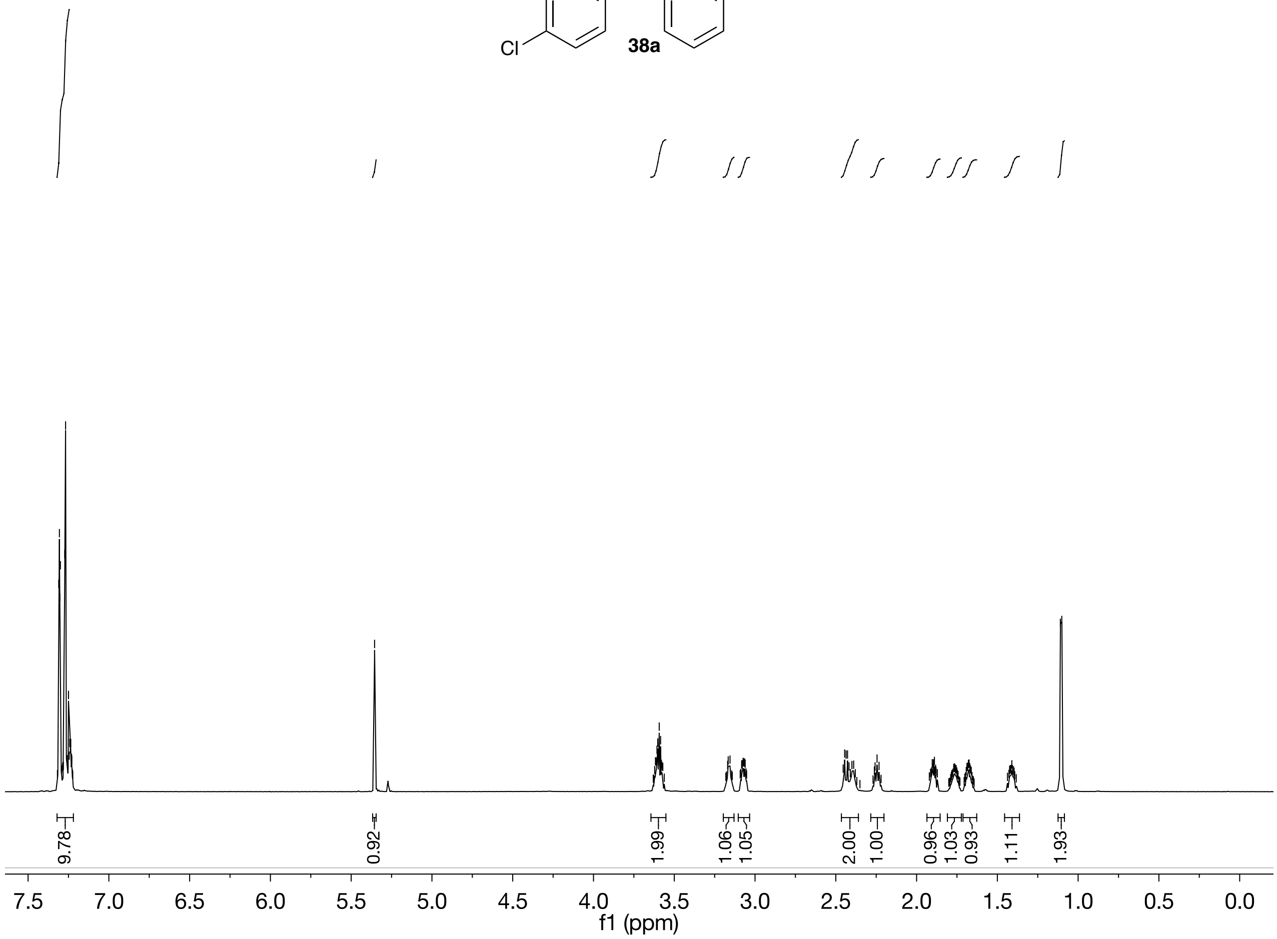

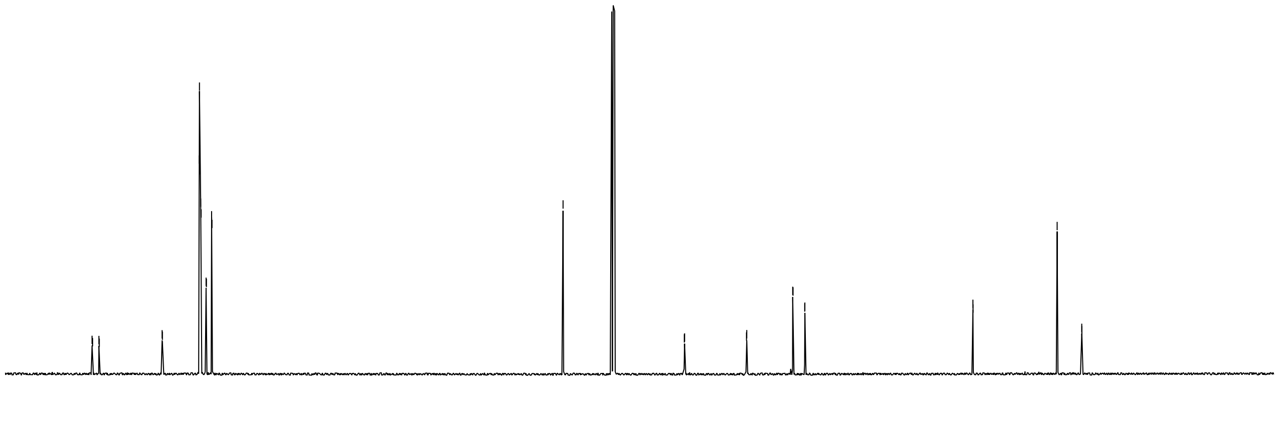

^{1}H and ^{13}C NMR Spectra for **38b**

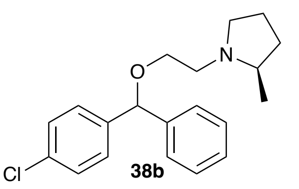

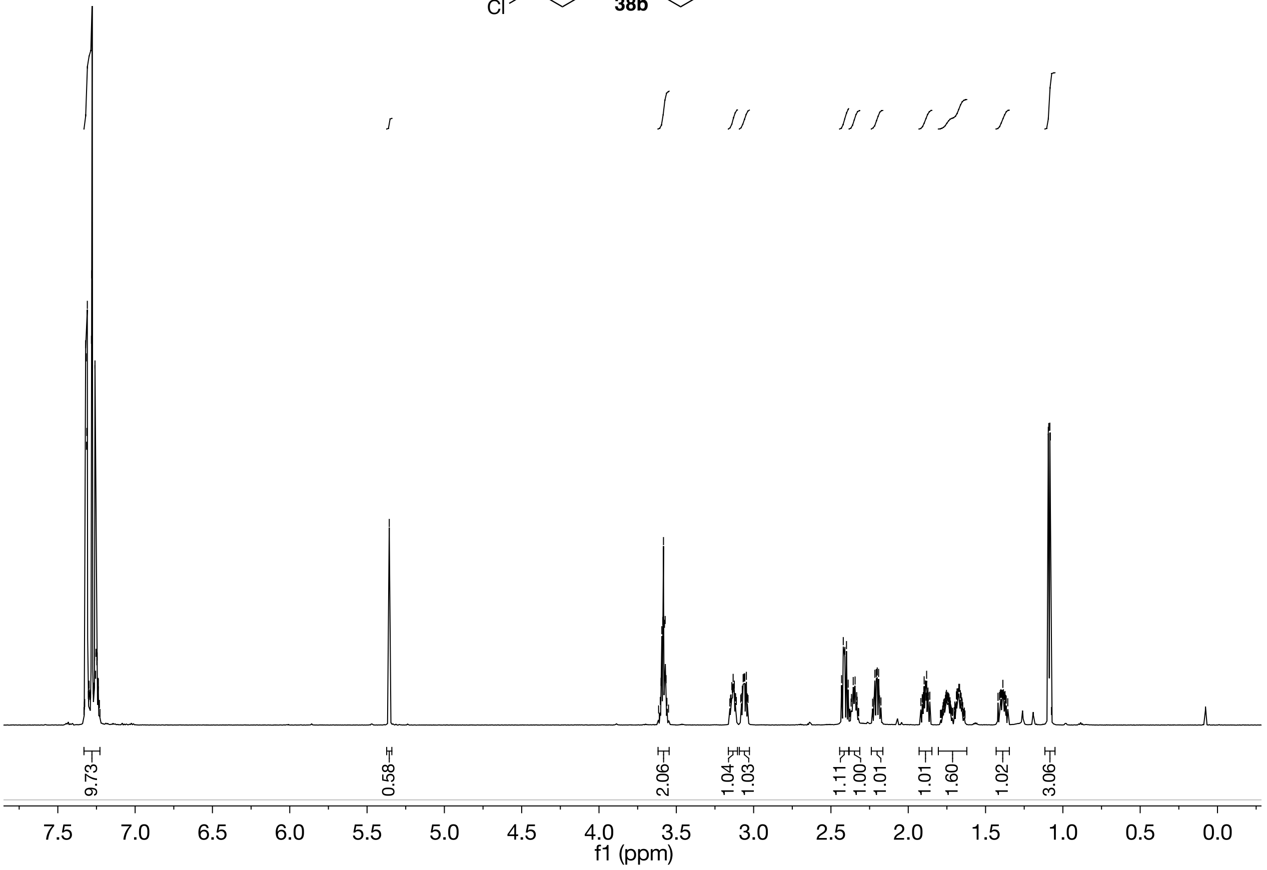

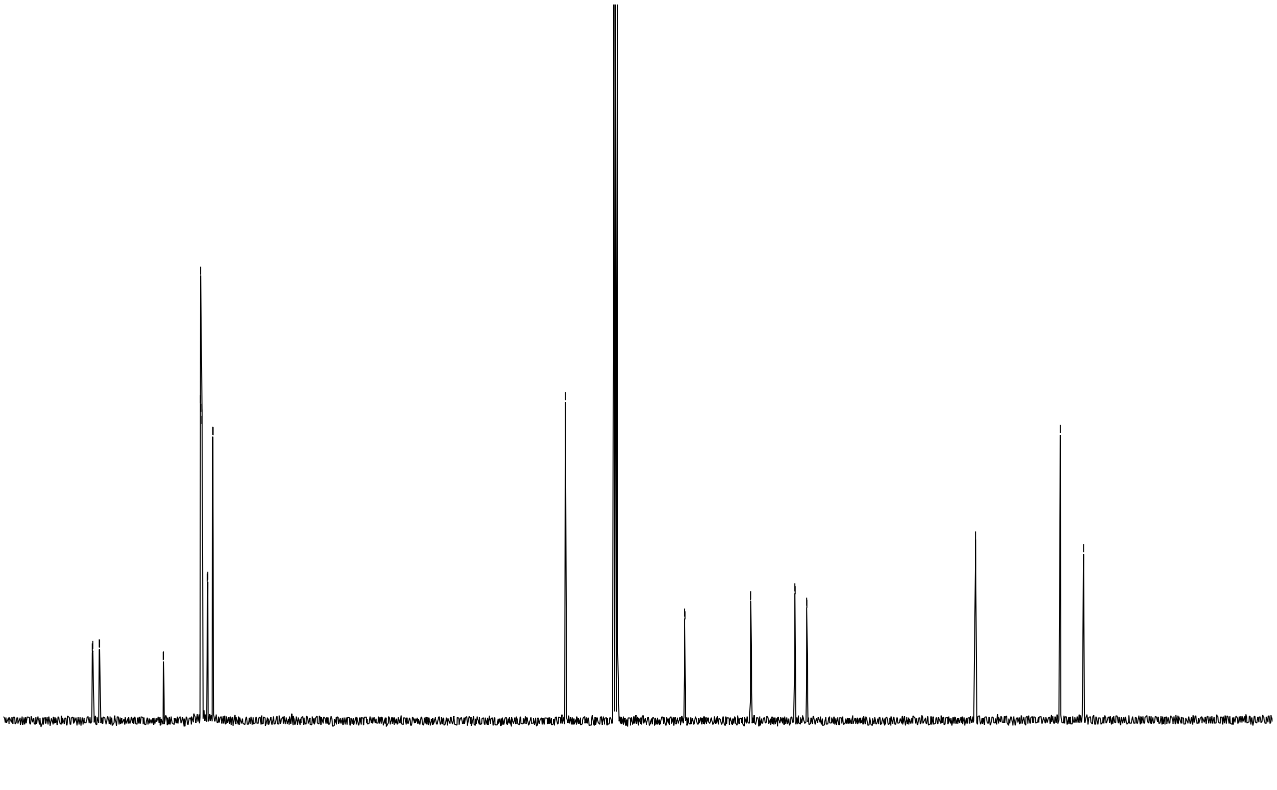

^{1}H and ^{13}C NMR Spectra for **38c**

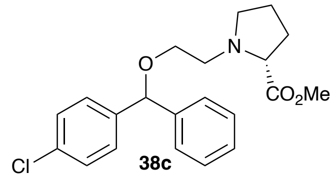

38c

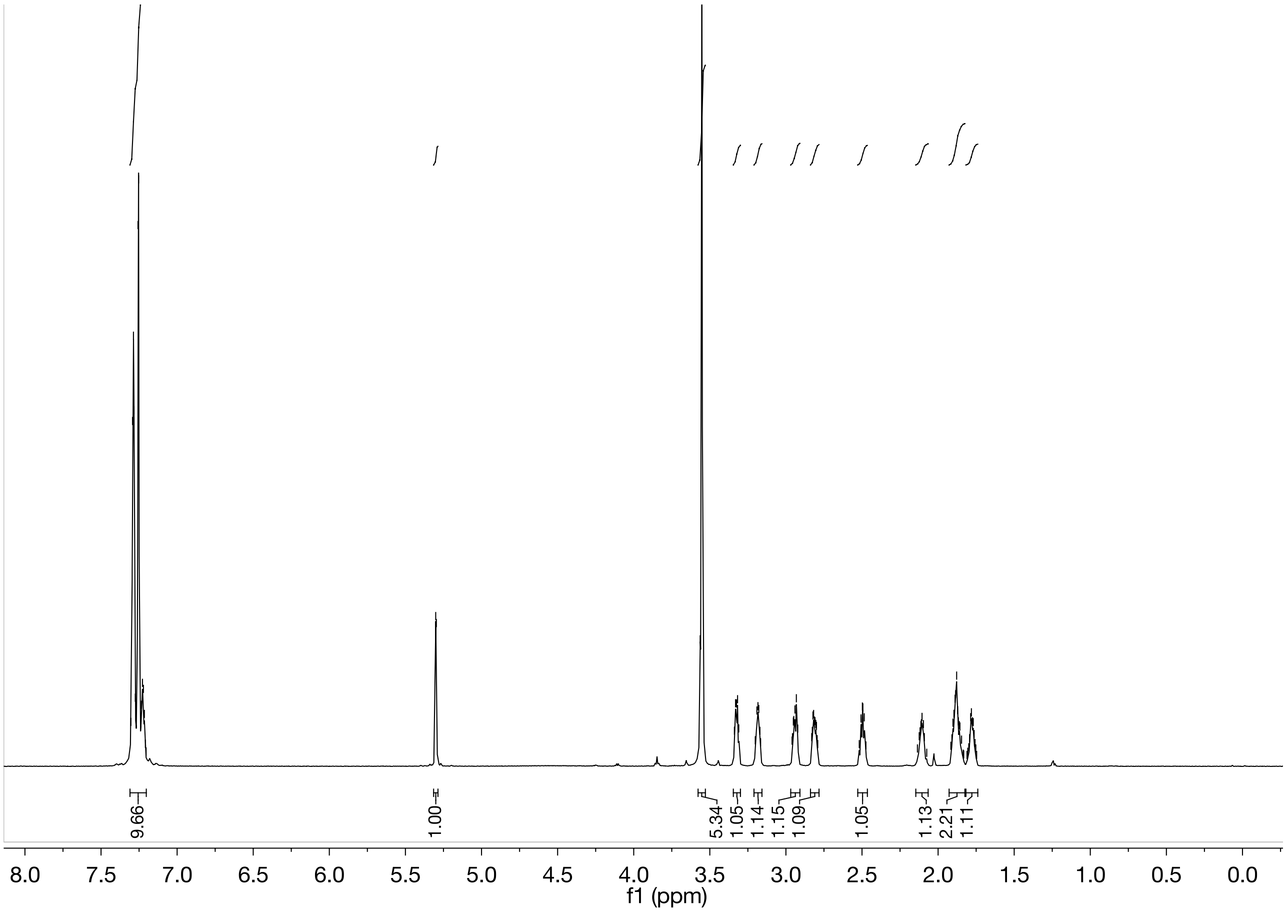

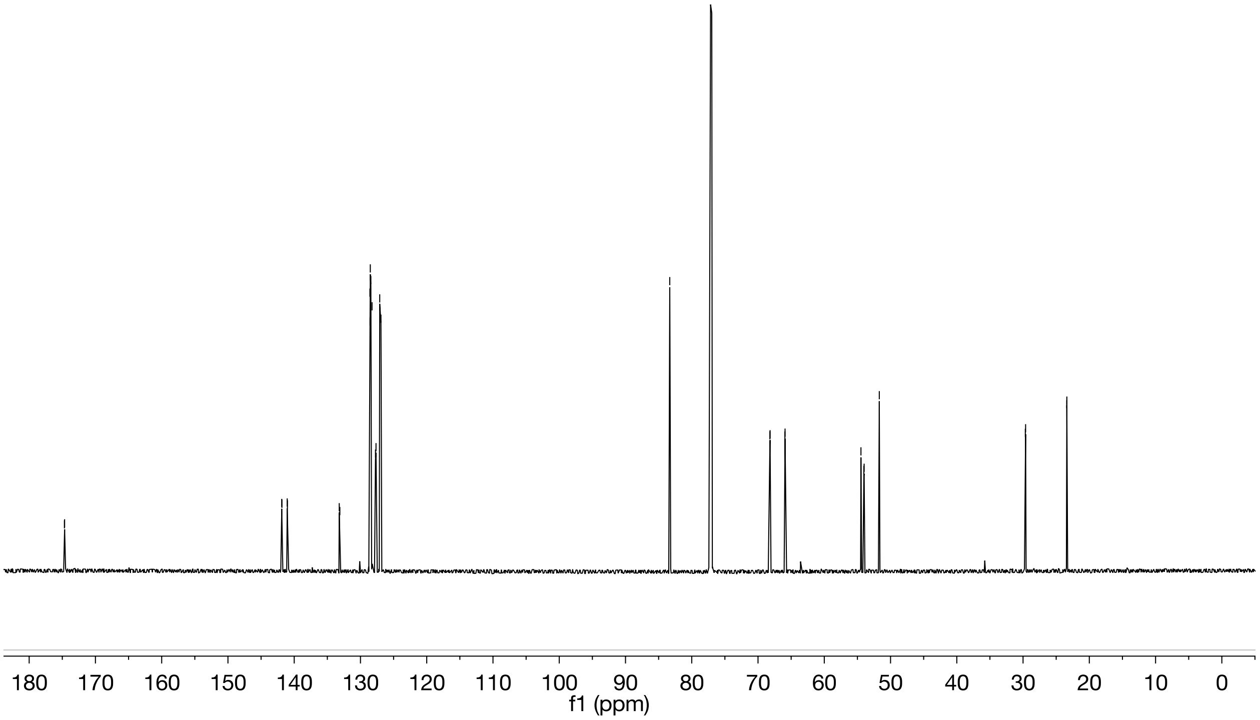

^{1}H and ^{13}C NMR Spectra for **38d**

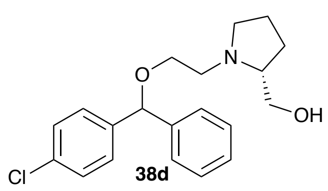

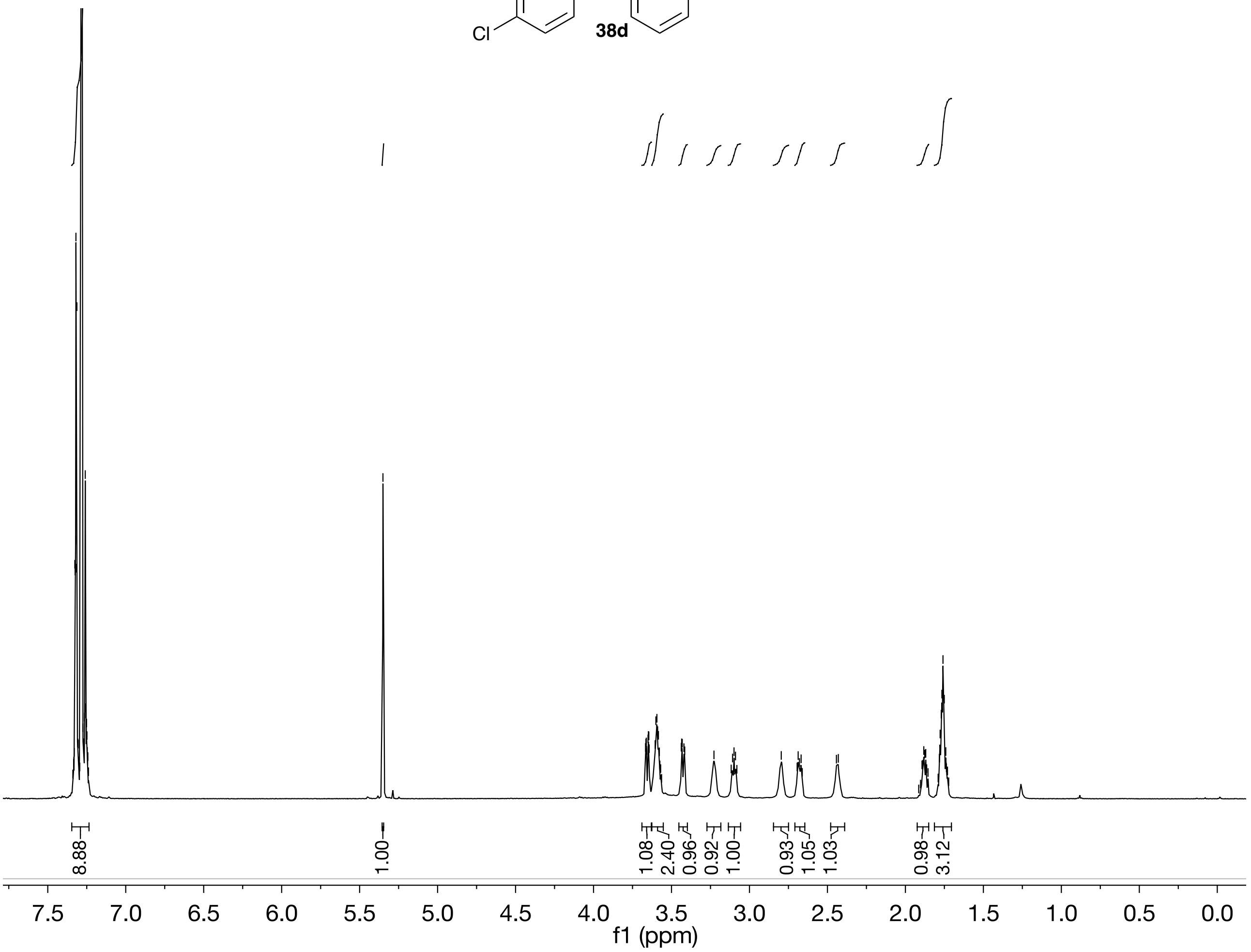

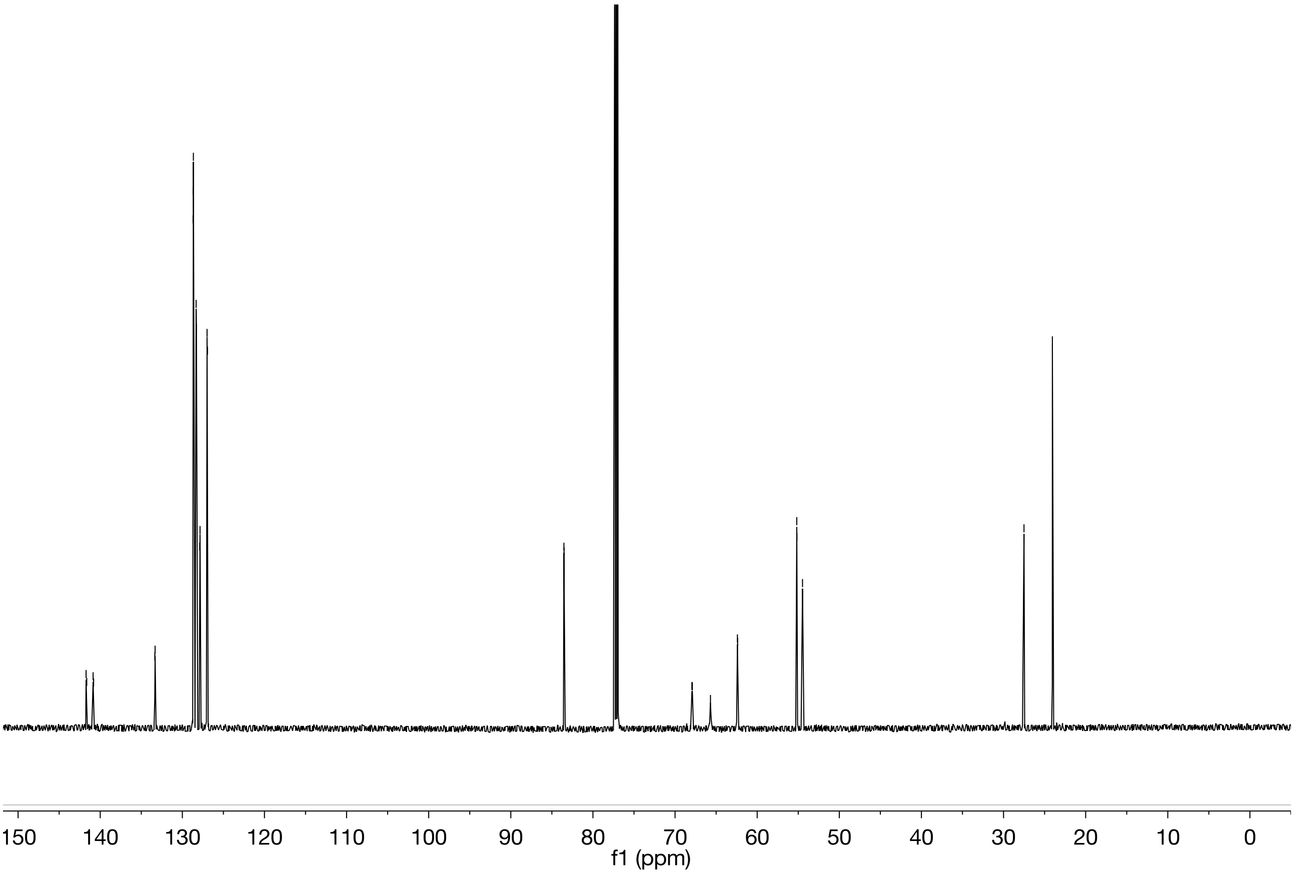

^{1}H and ^{13}C NMR Spectra for **38e**

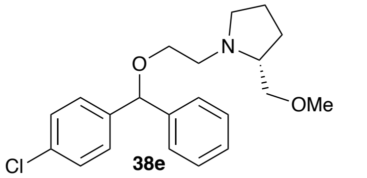

38e

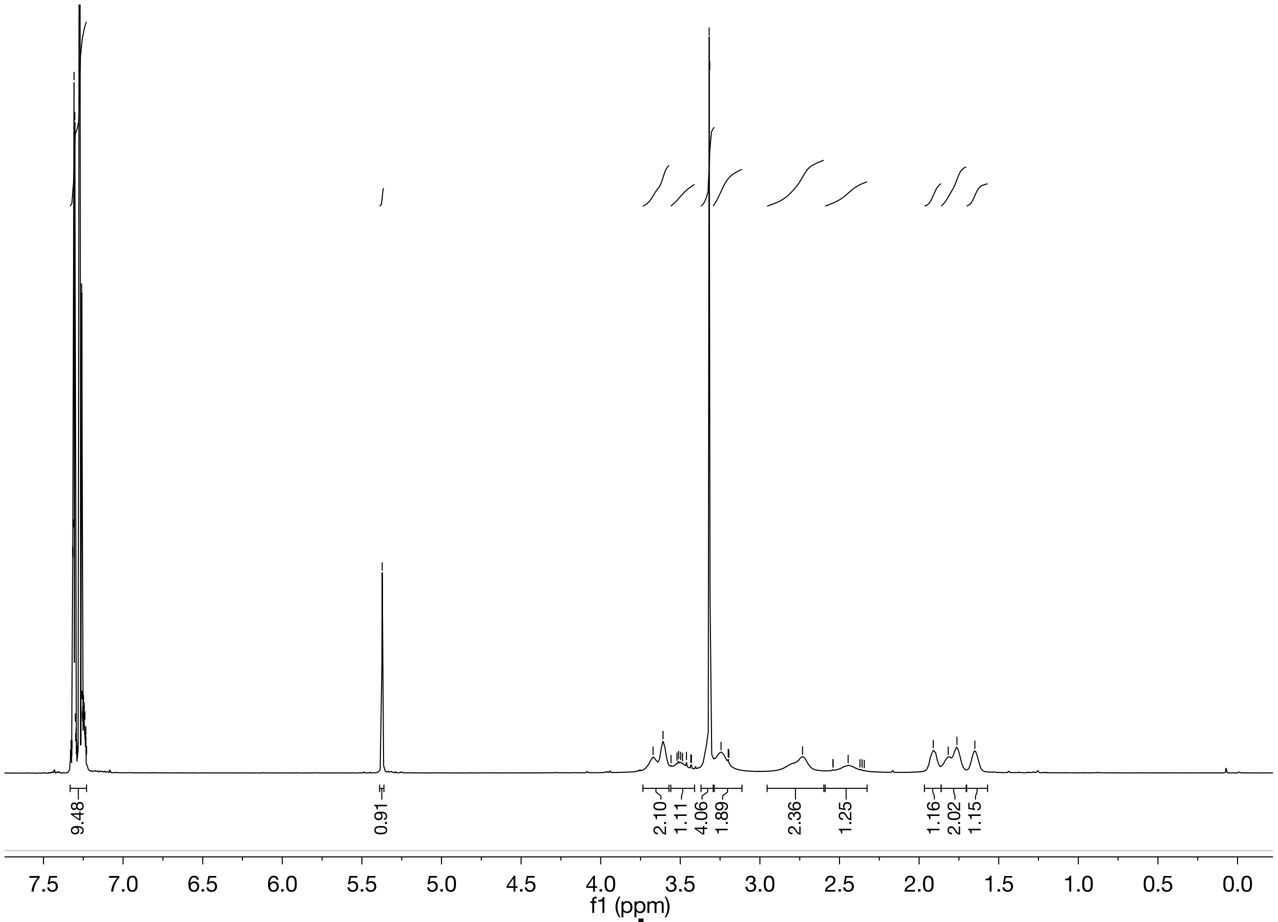

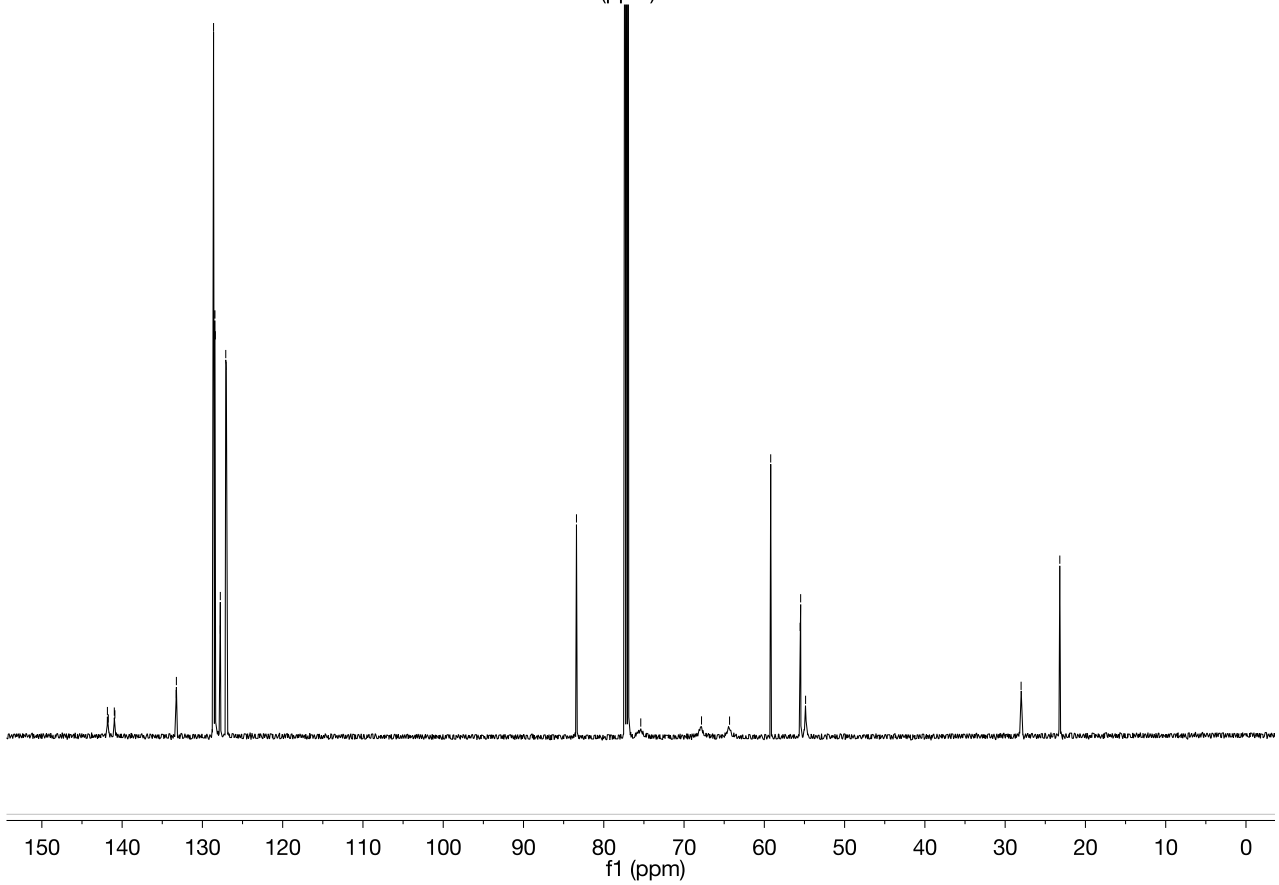

^{1}H and ^{13}C NMR Spectra for **38f**

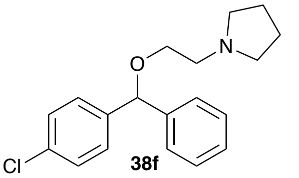

38f

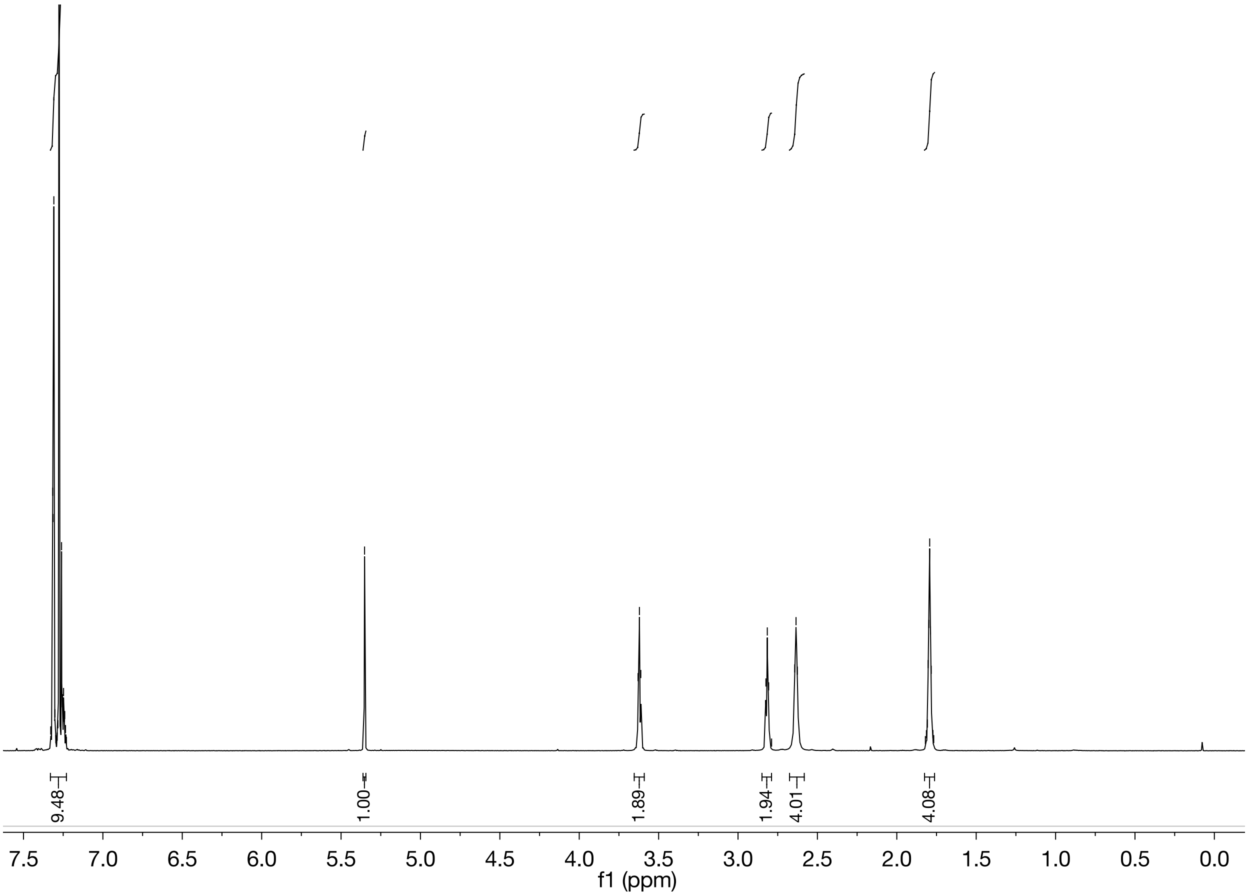

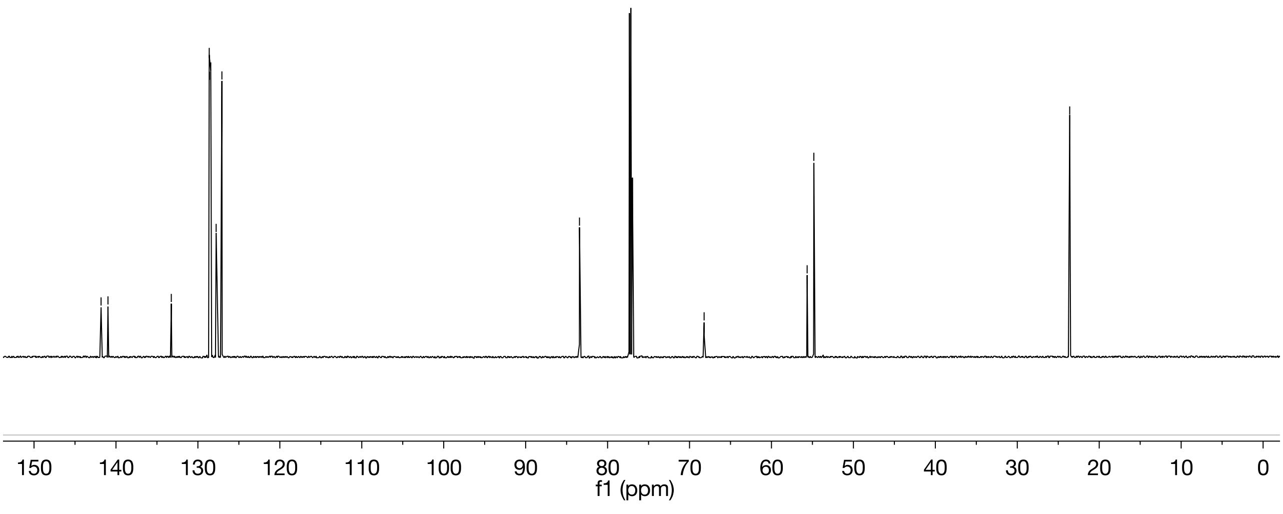

^{1}H and ^{13}C NMR Spectra for **38g**

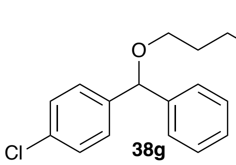

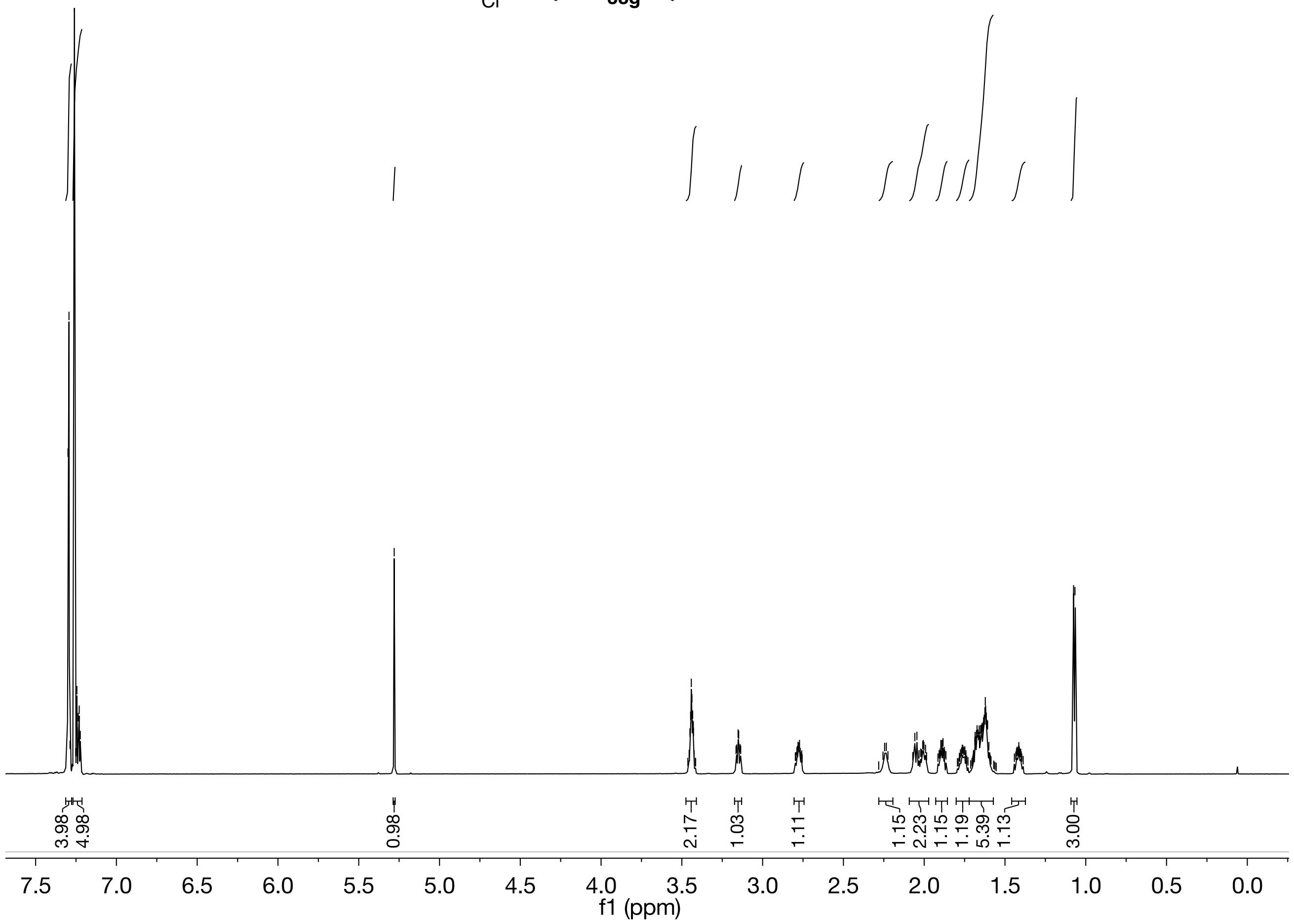

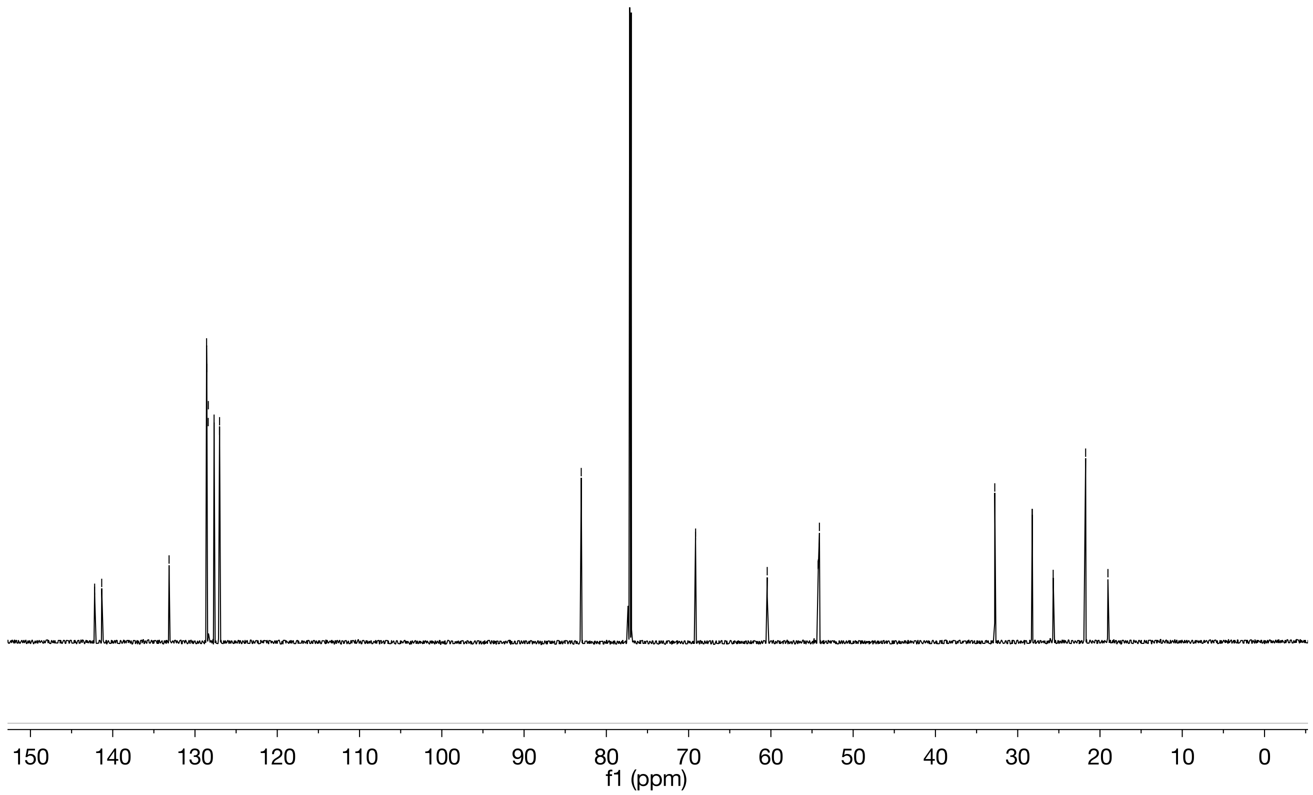

^1H and ^{13}C NMR Spectra for **38h**

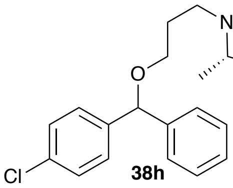

38h

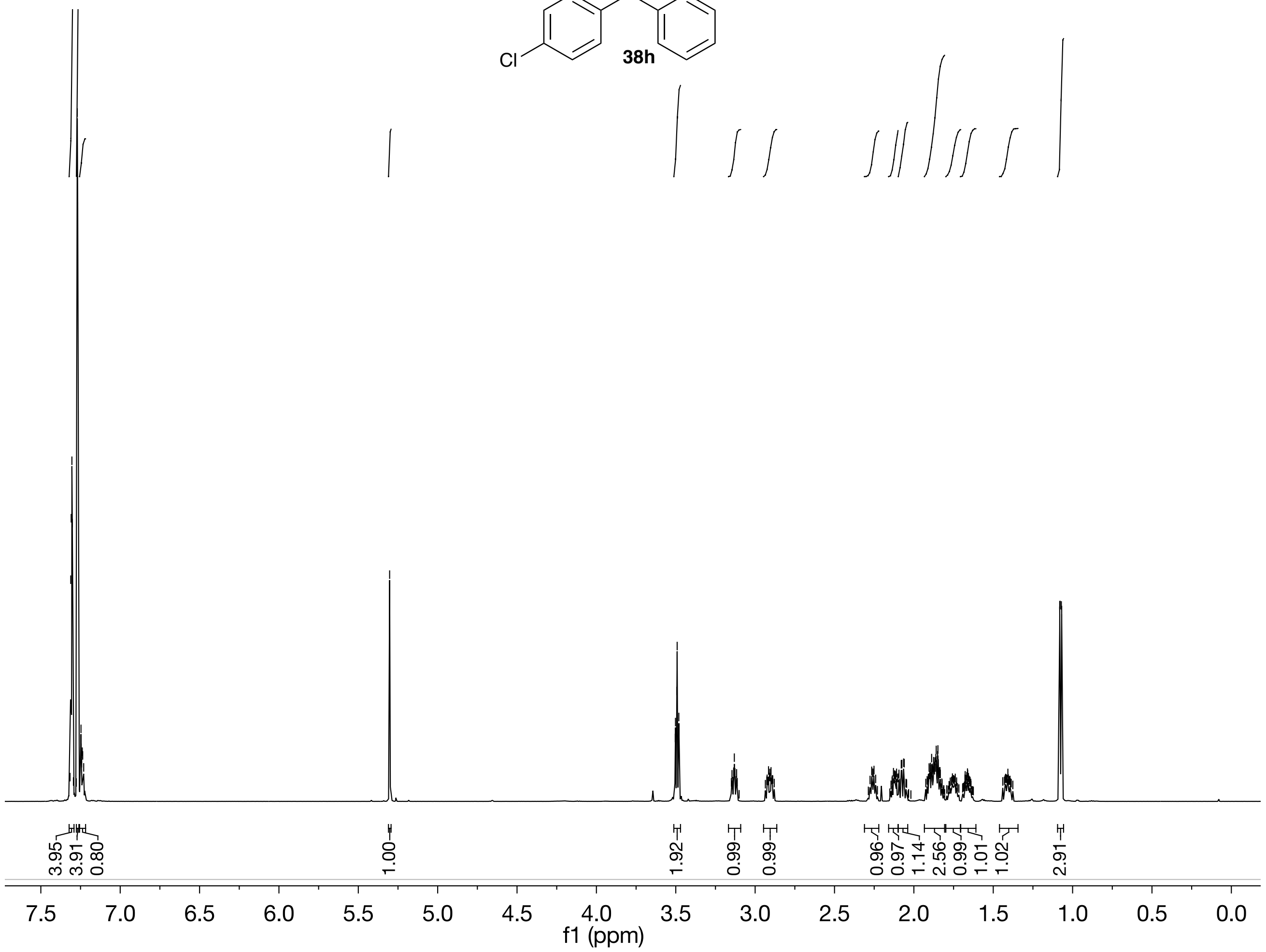

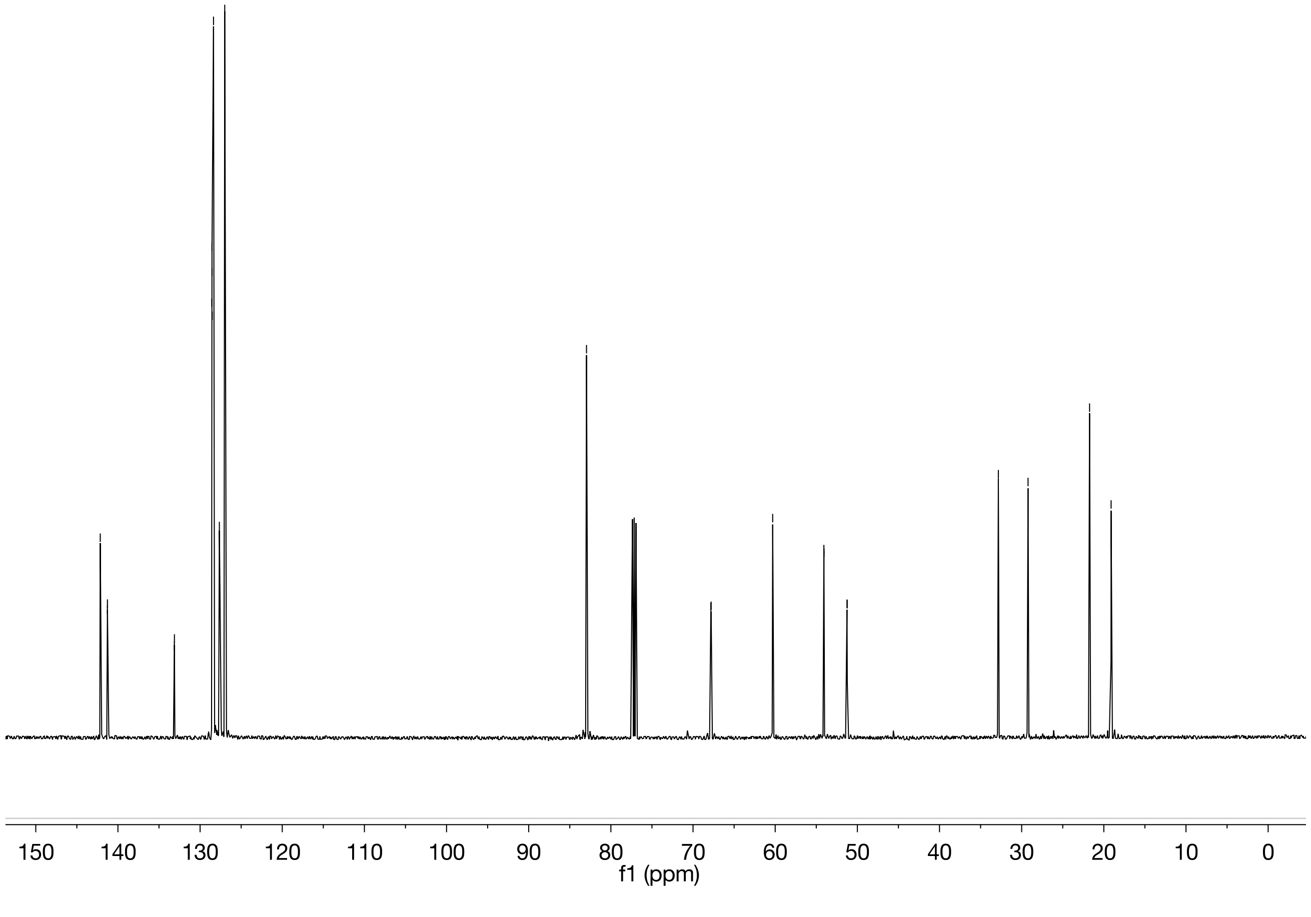

^{1}H and ^{13}C NMR Spectra for **38i**

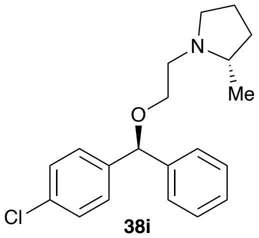

38i

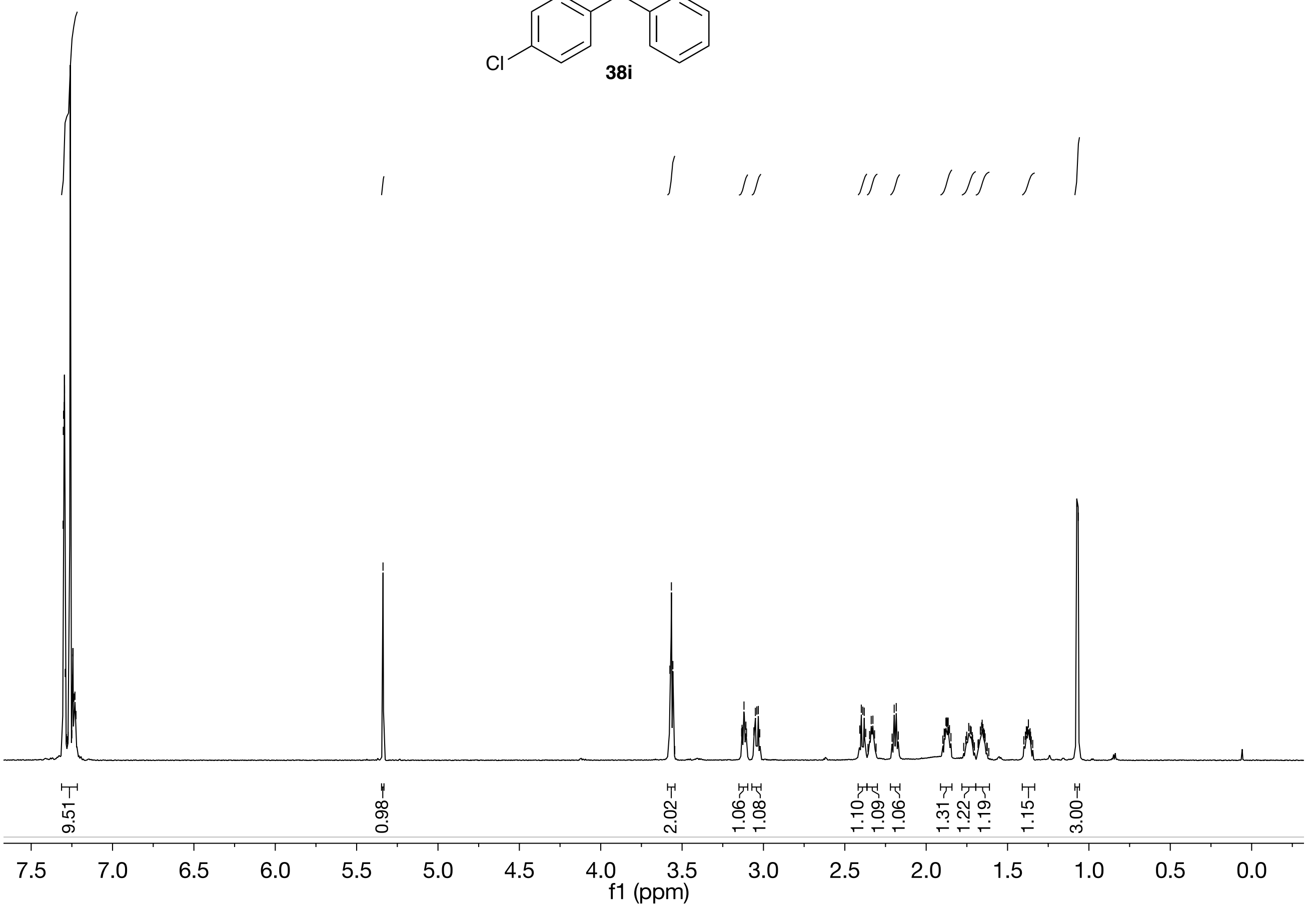

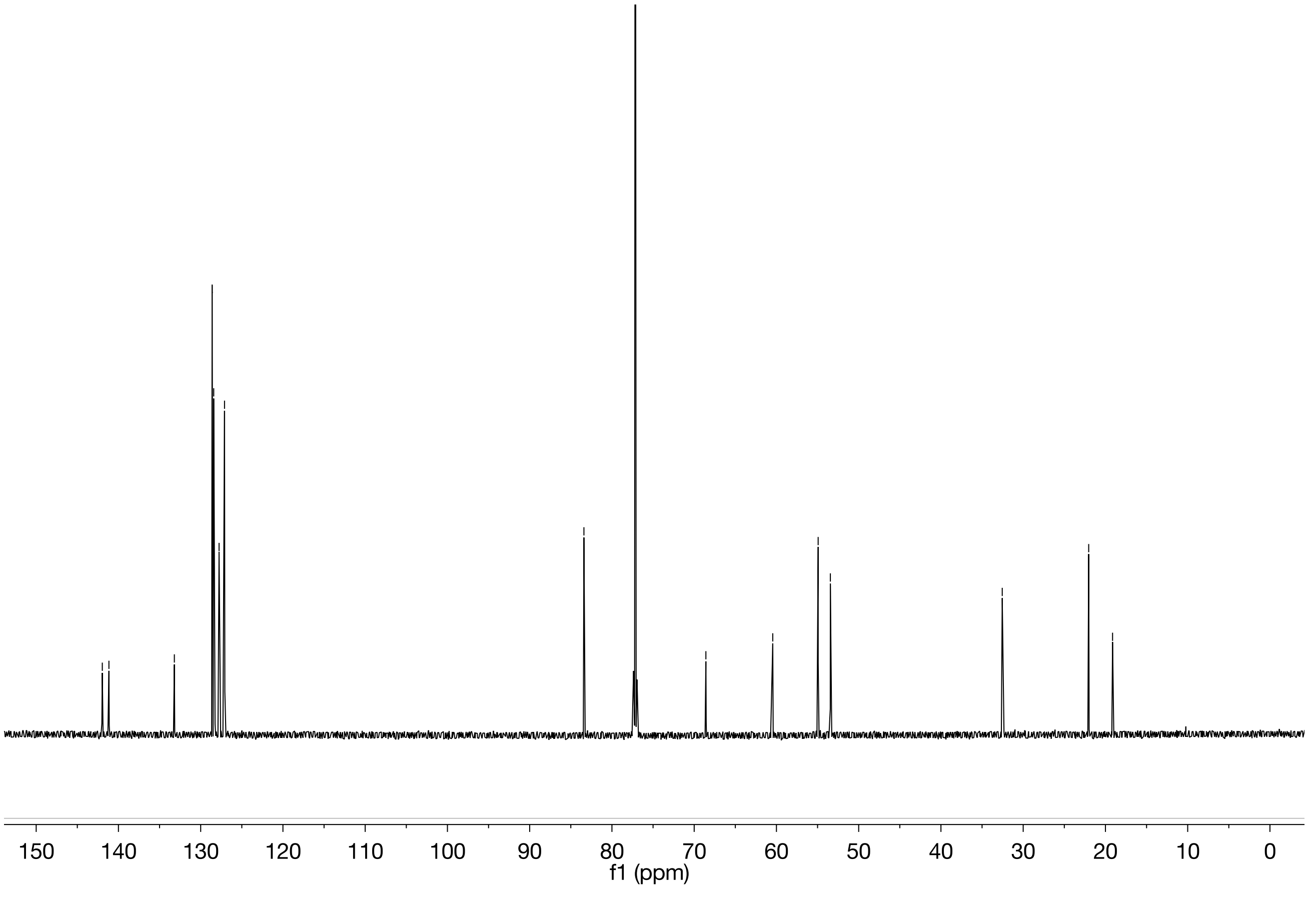

^1H and ^{13}C NMR Spectra for **38j**

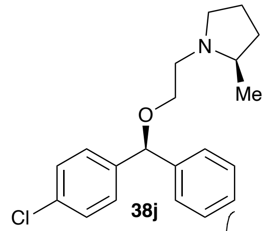

38j

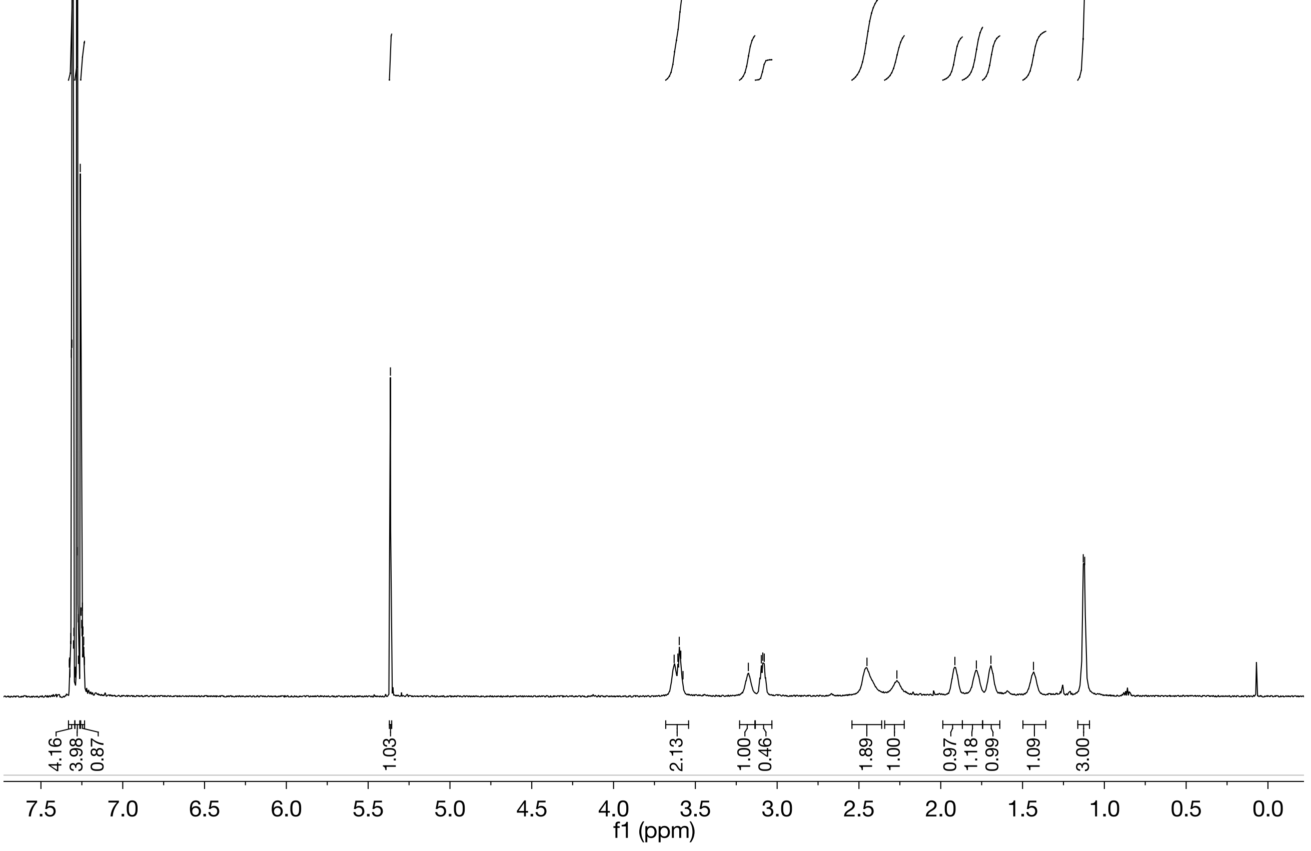

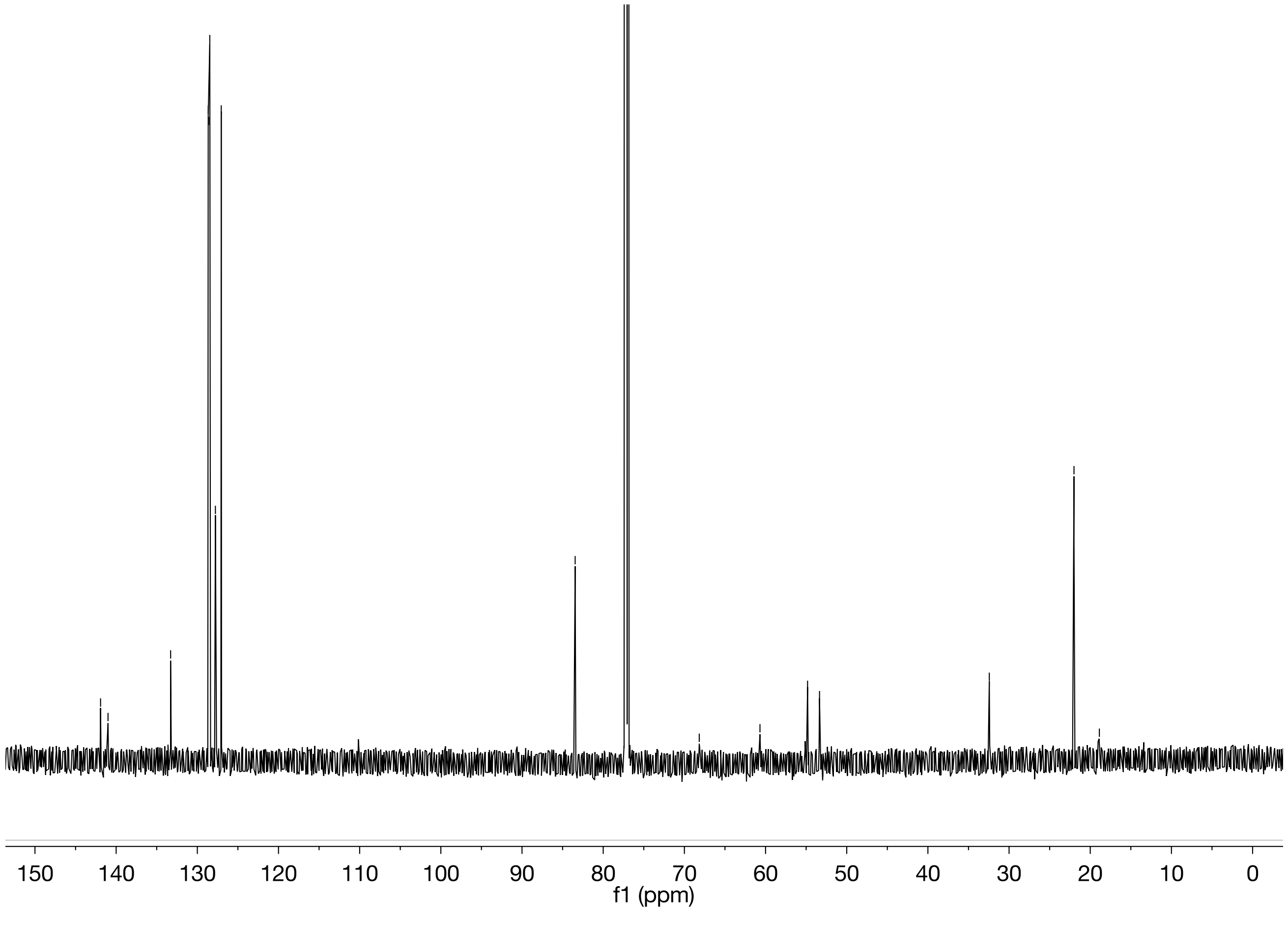

^{1}H and ^{13}C NMR Spectra for **38k**

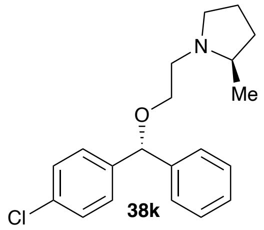

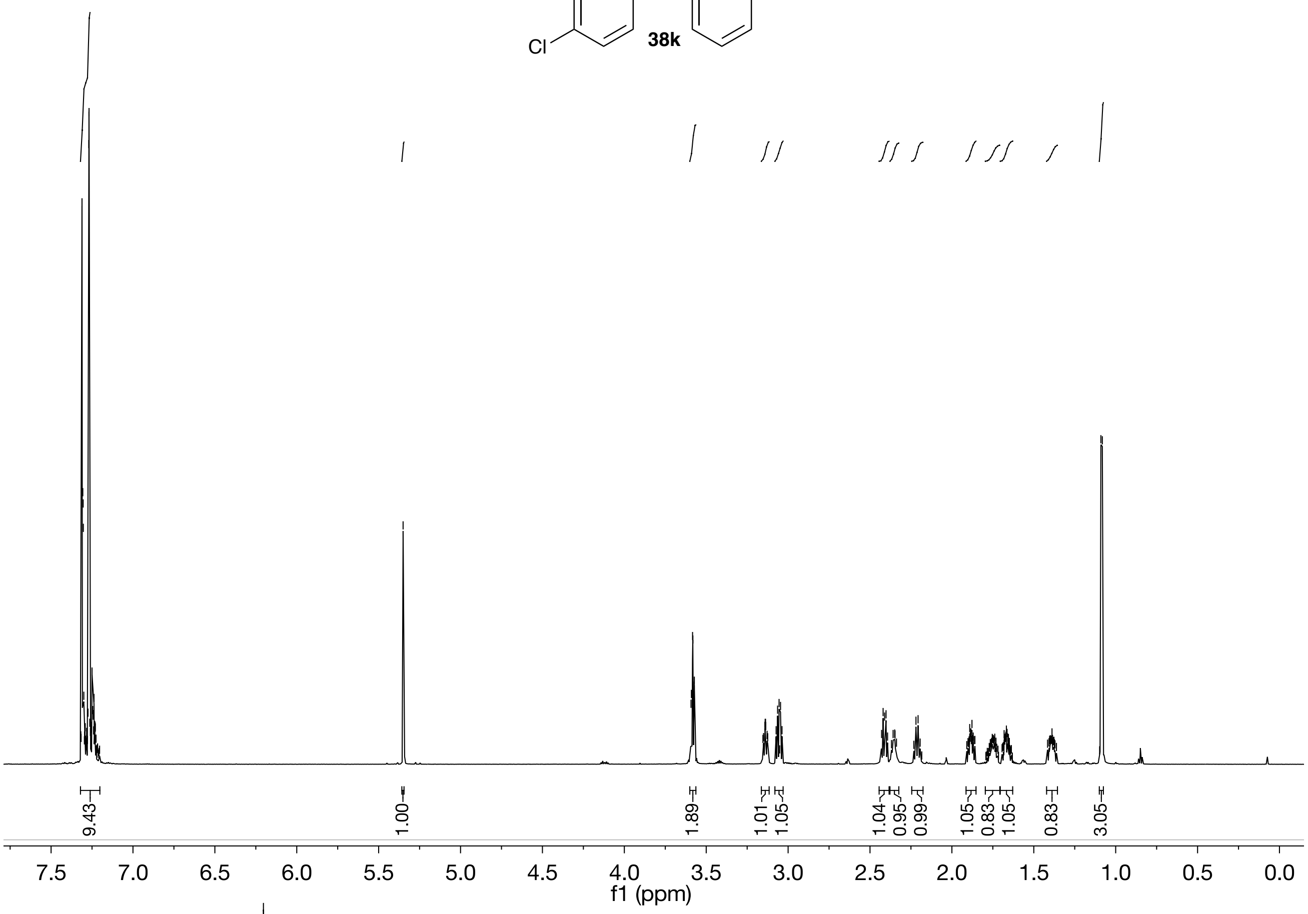

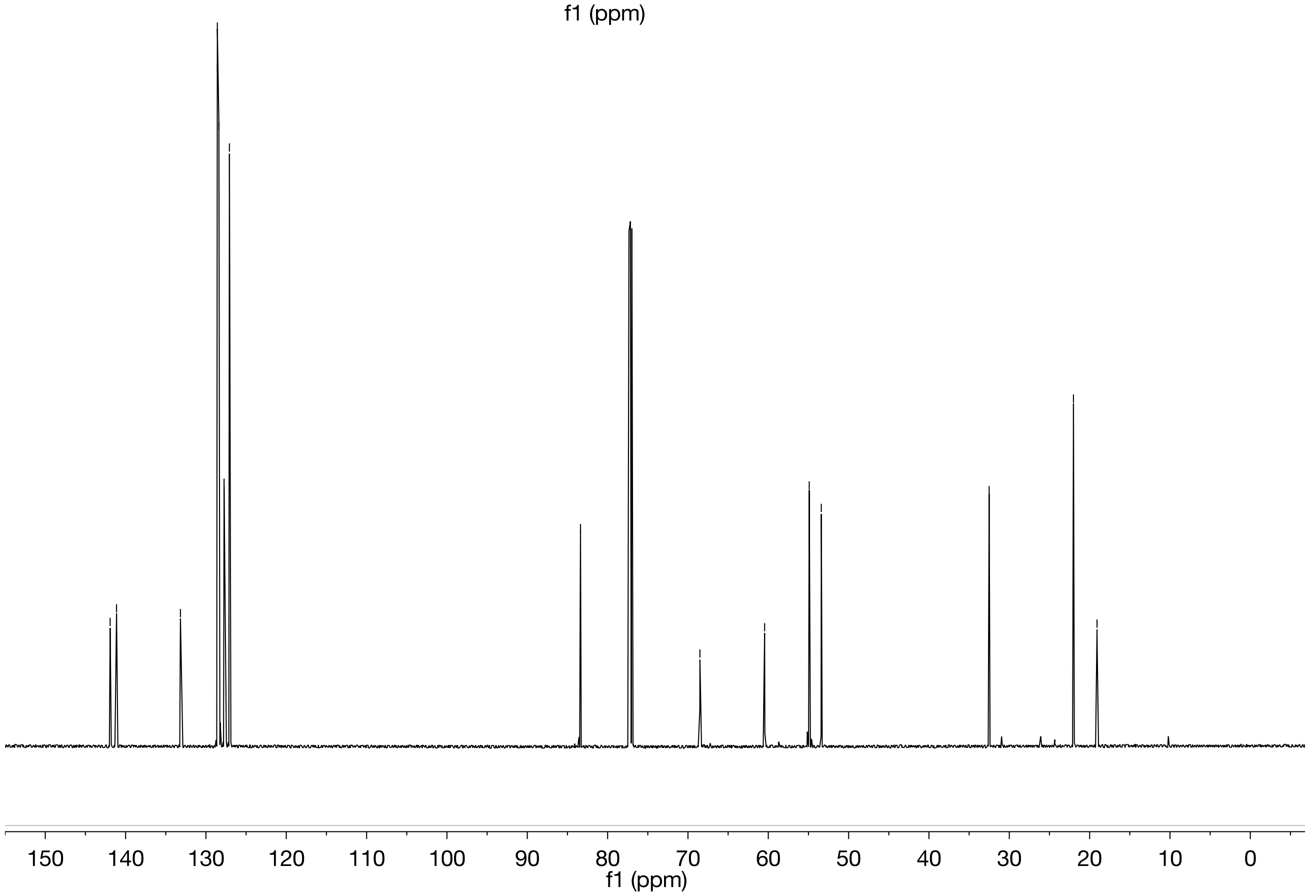

^{1}H and ^{13}C NMR Spectra for **38l**

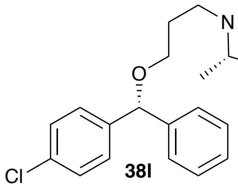

38l

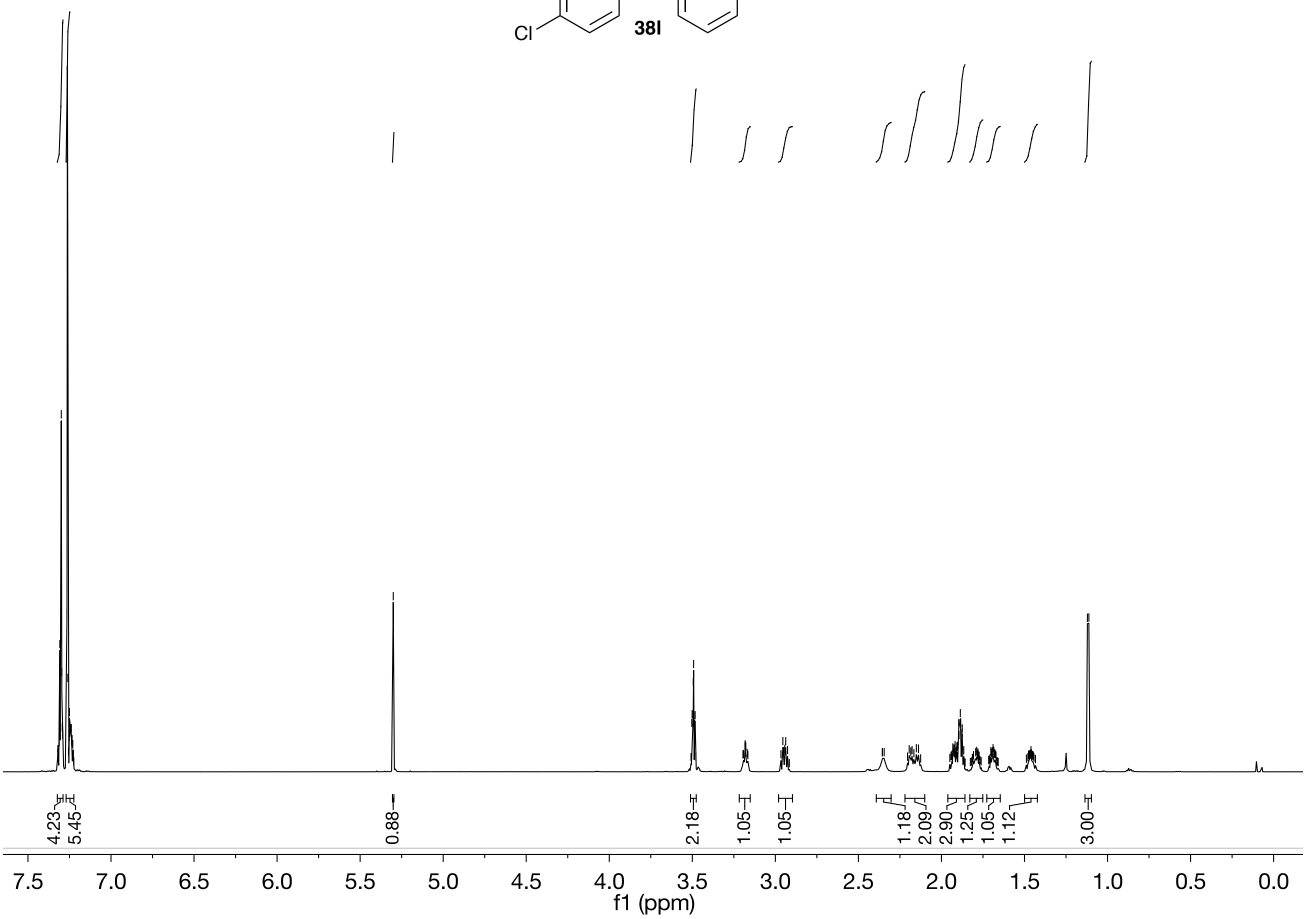

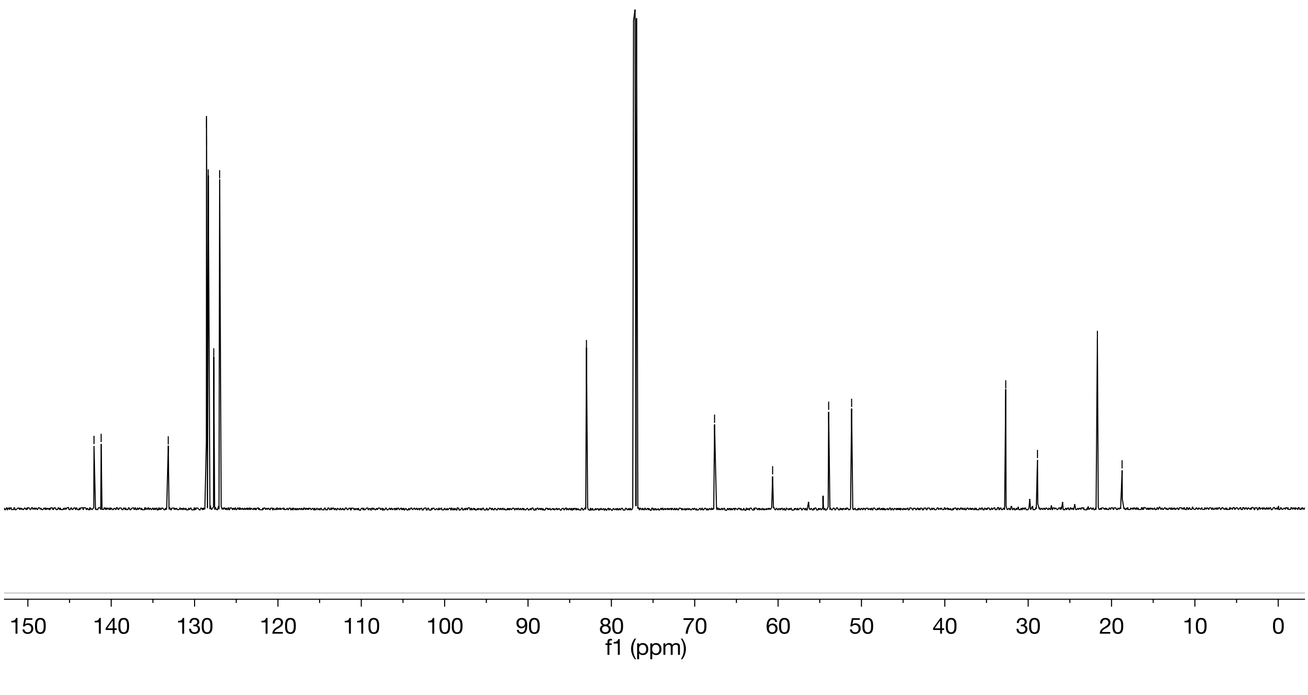

^{1}H and ^{13}C NMR Spectra for **38m**

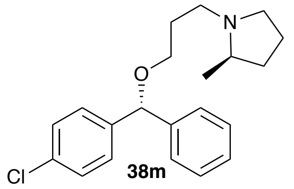

38m

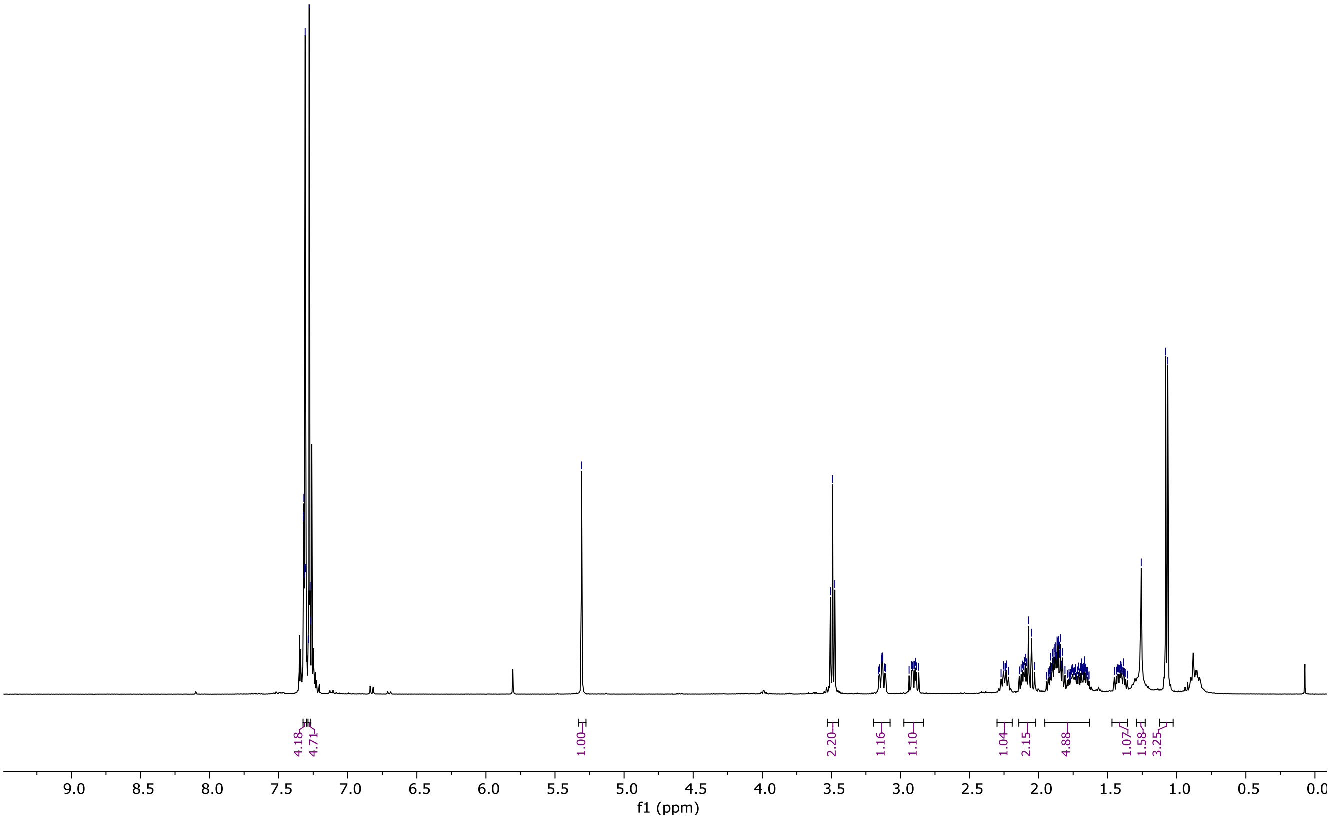

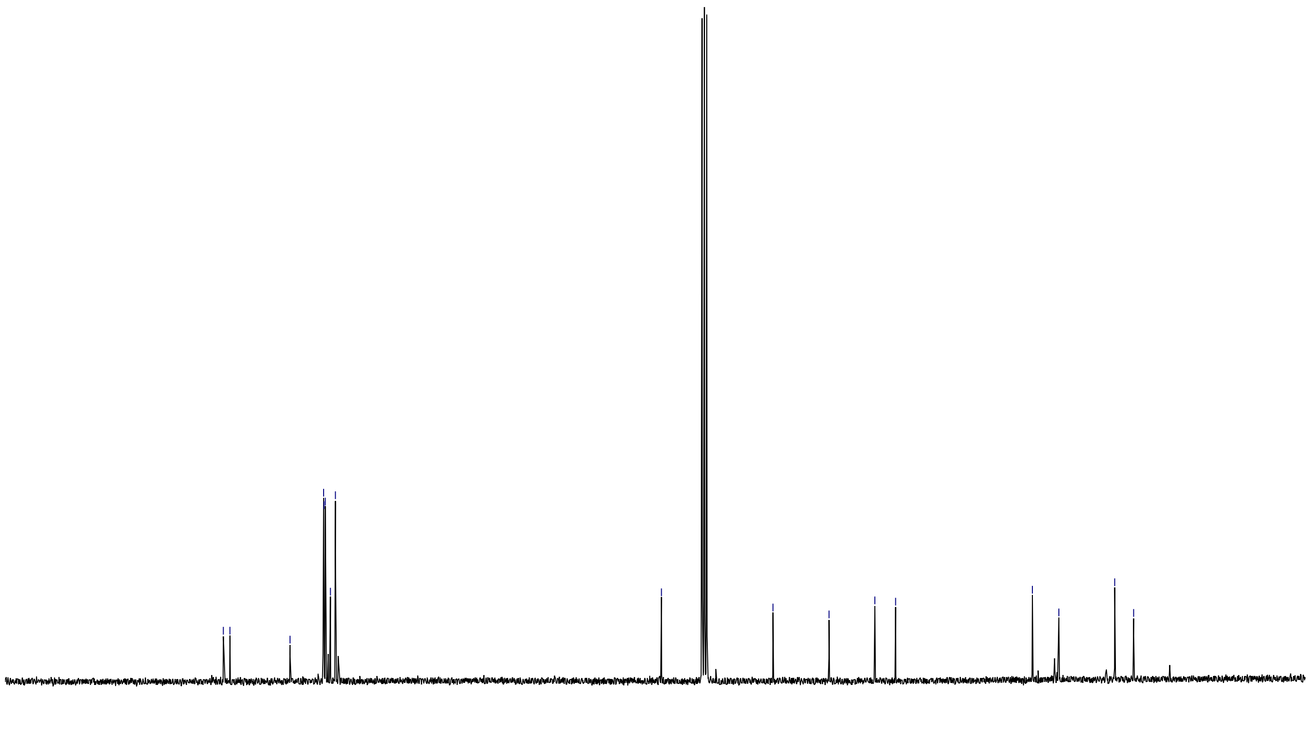

^{1}H and ^{13}C NMR Spectra for **38n**

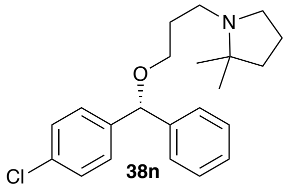

38n

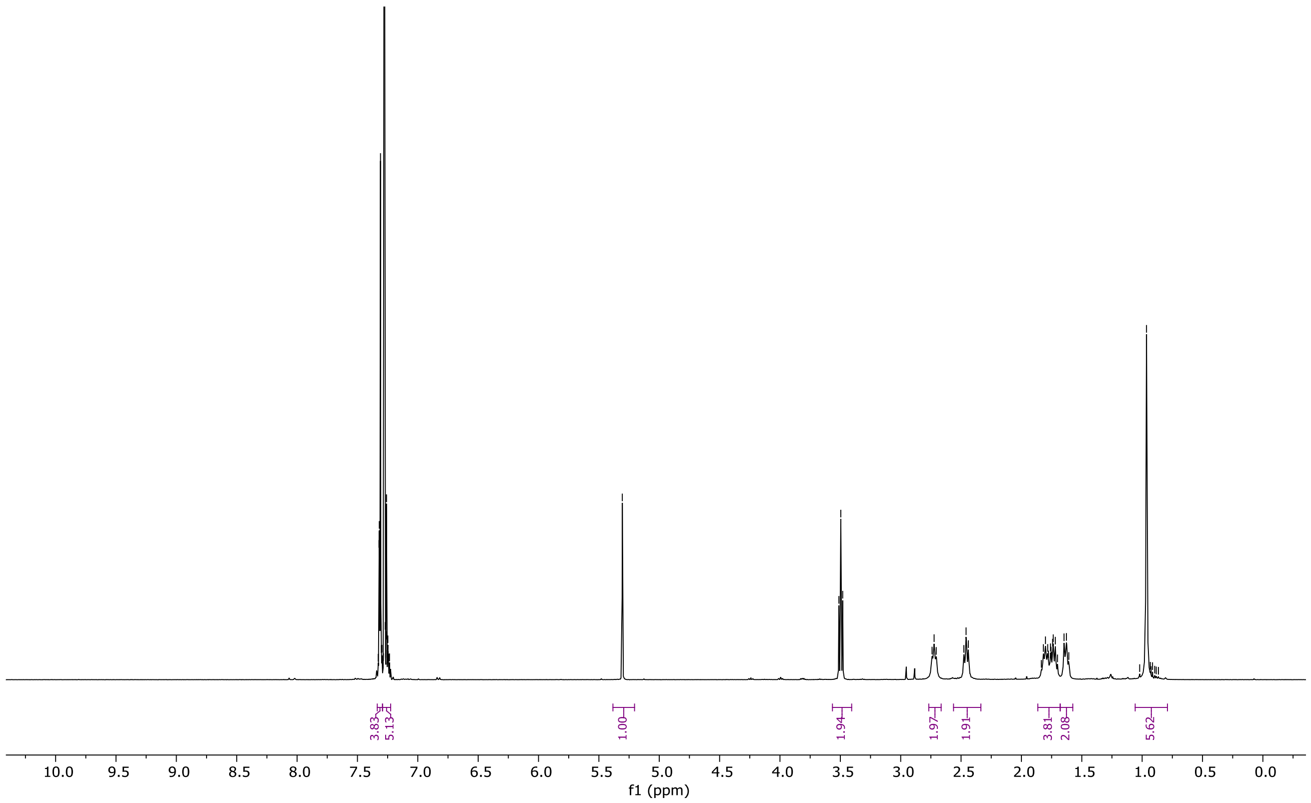

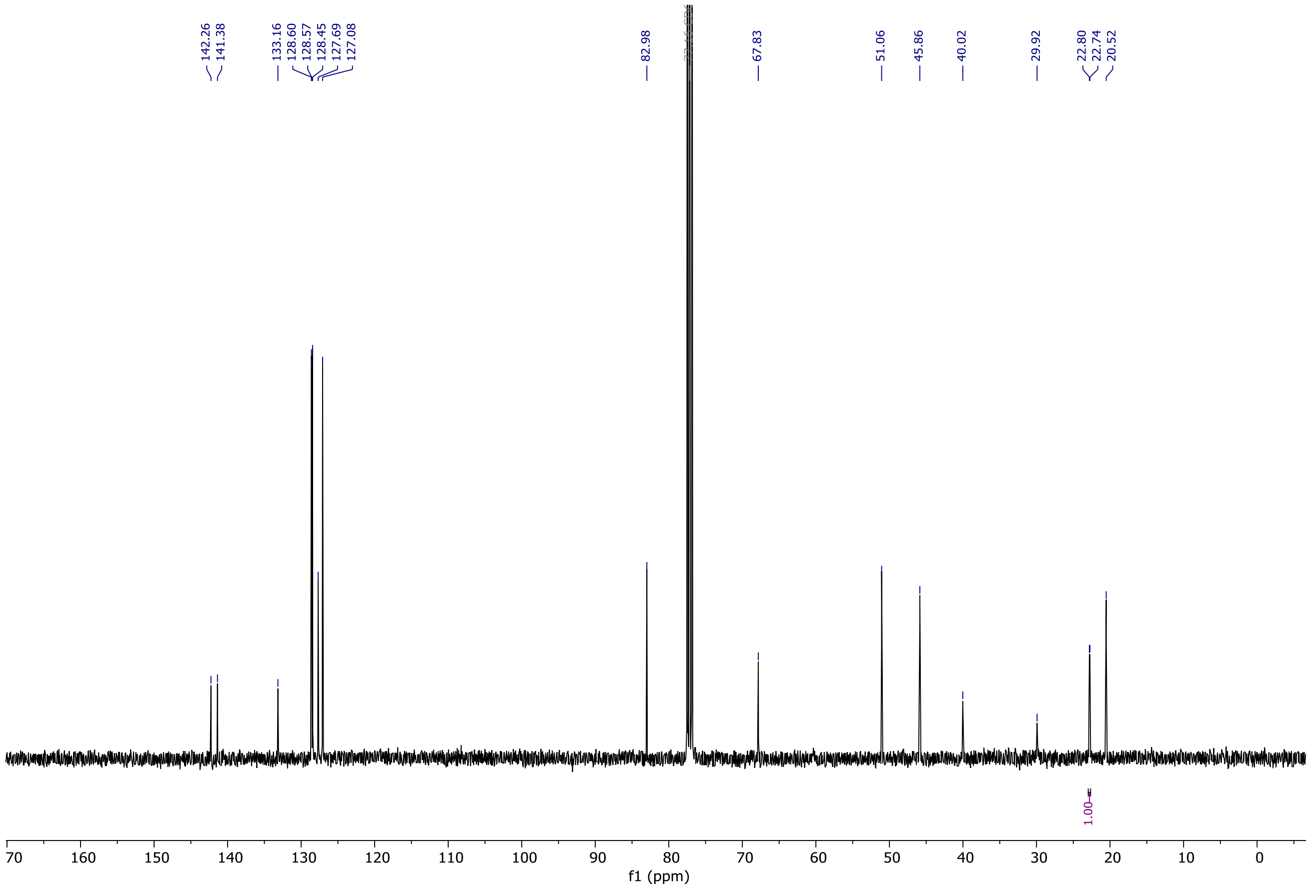

^{1}H and ^{13}C NMR Spectra for **38o**

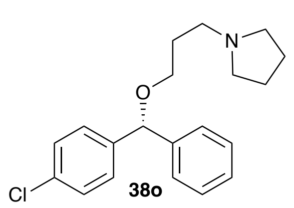

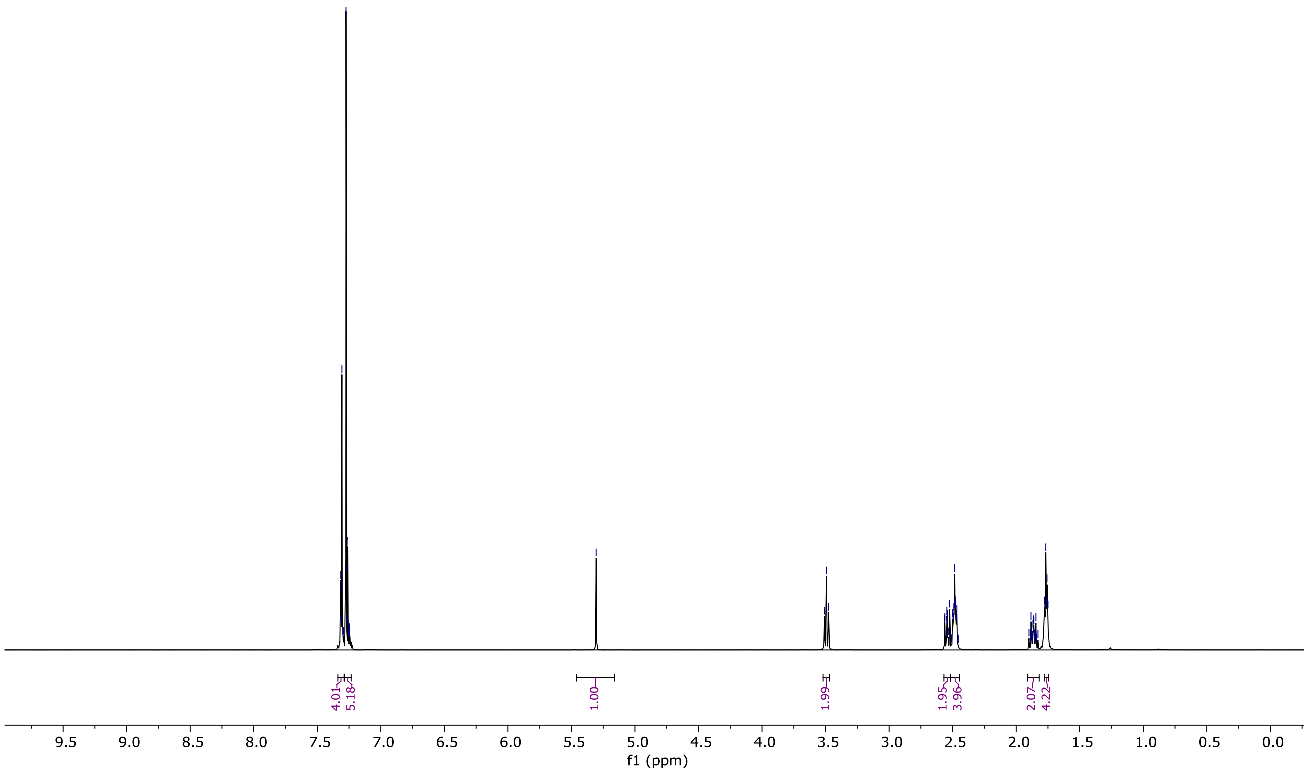

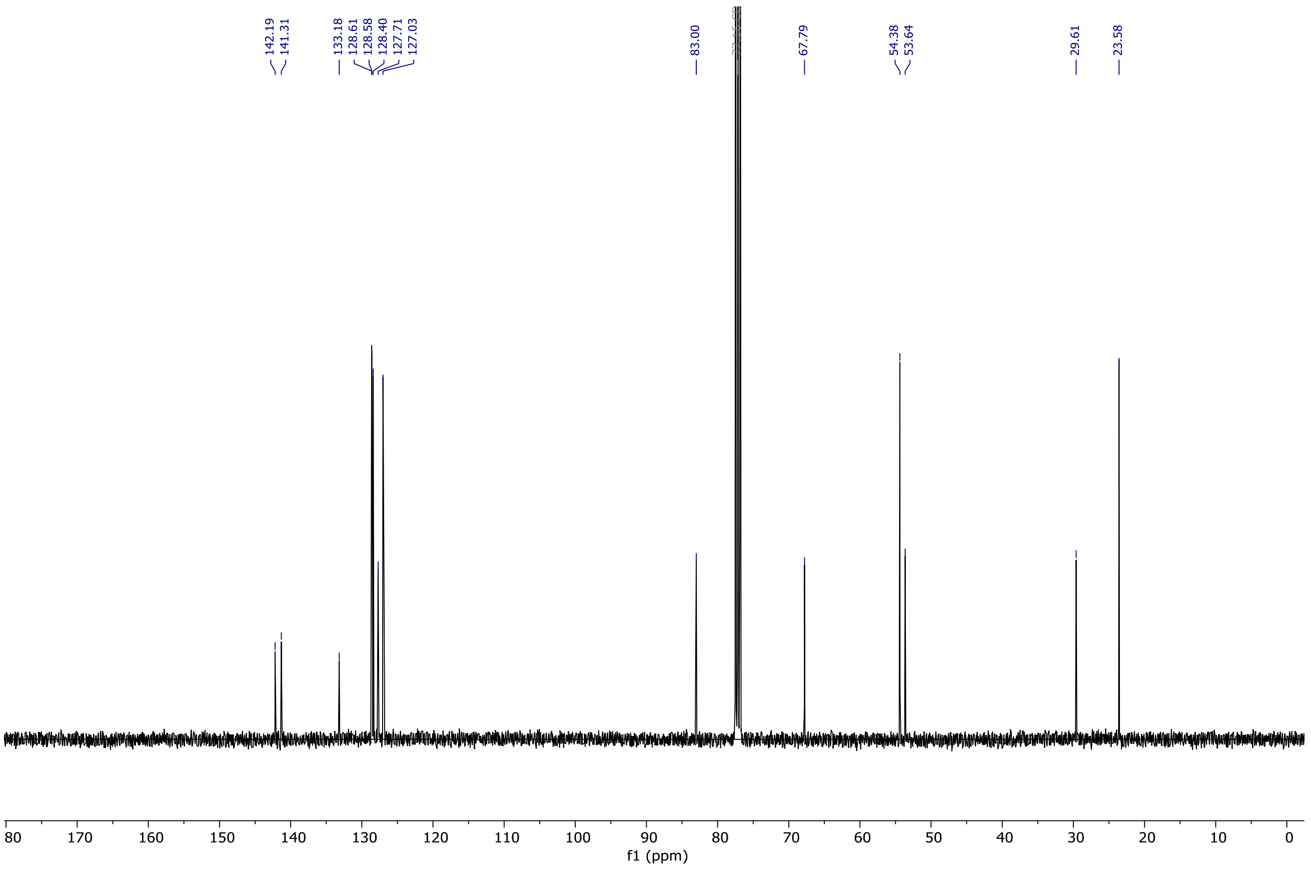

^{1}H and ^{13}C NMR Spectra for **S1**

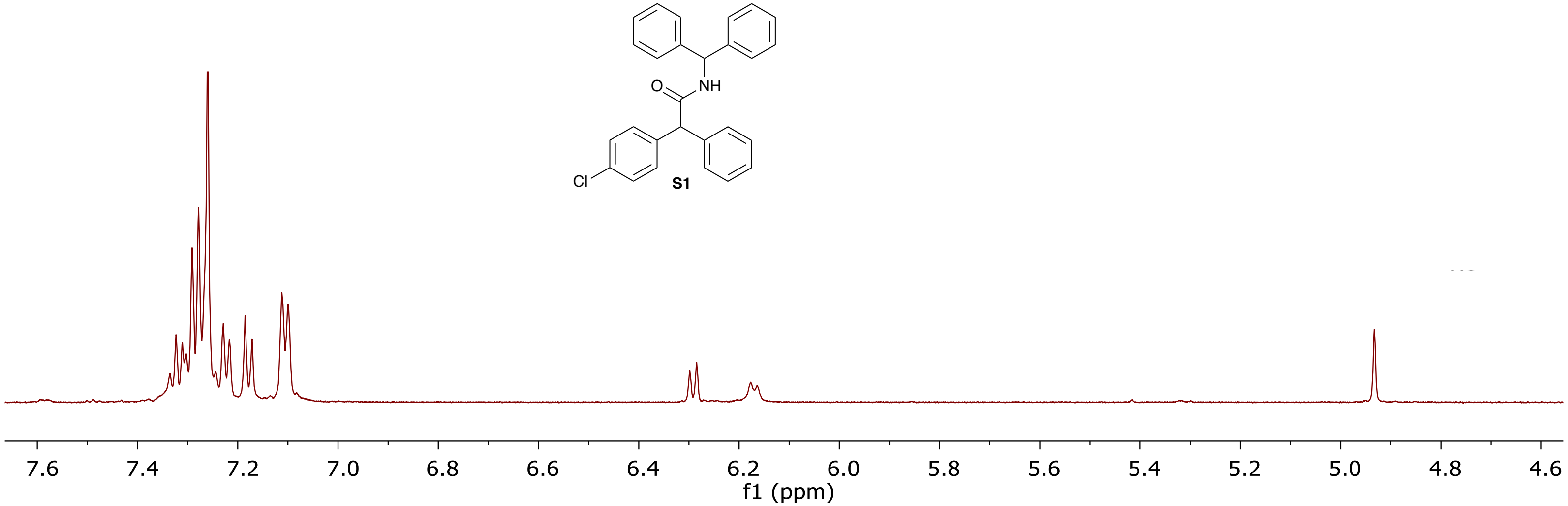

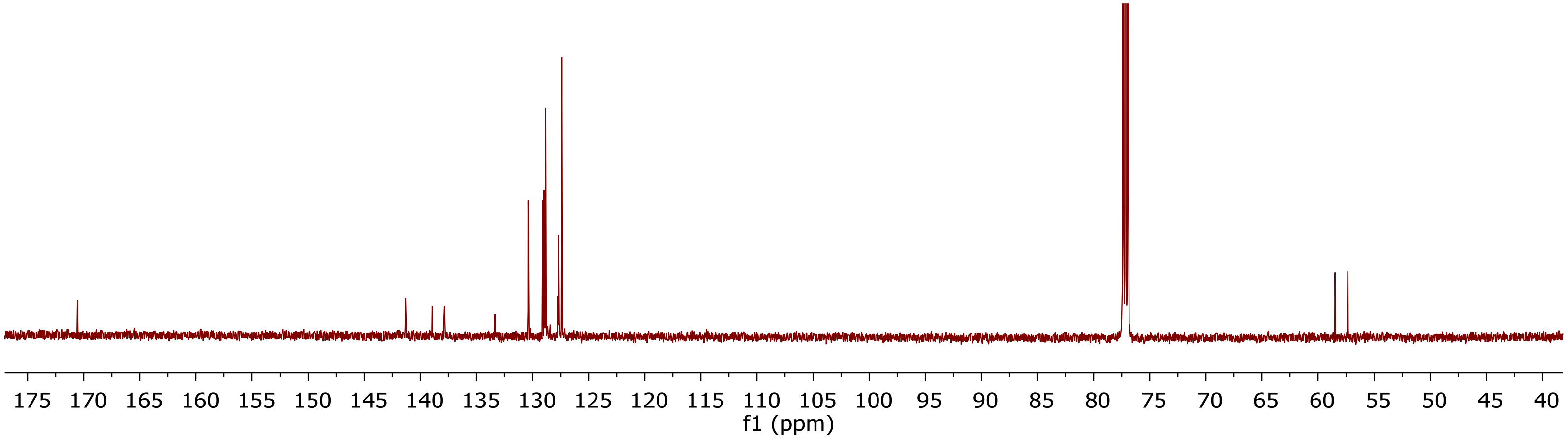

^{1}H and ^{13}C NMR Spectra for **S2**

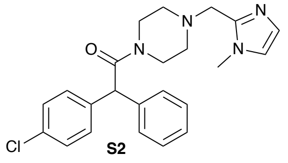

S2

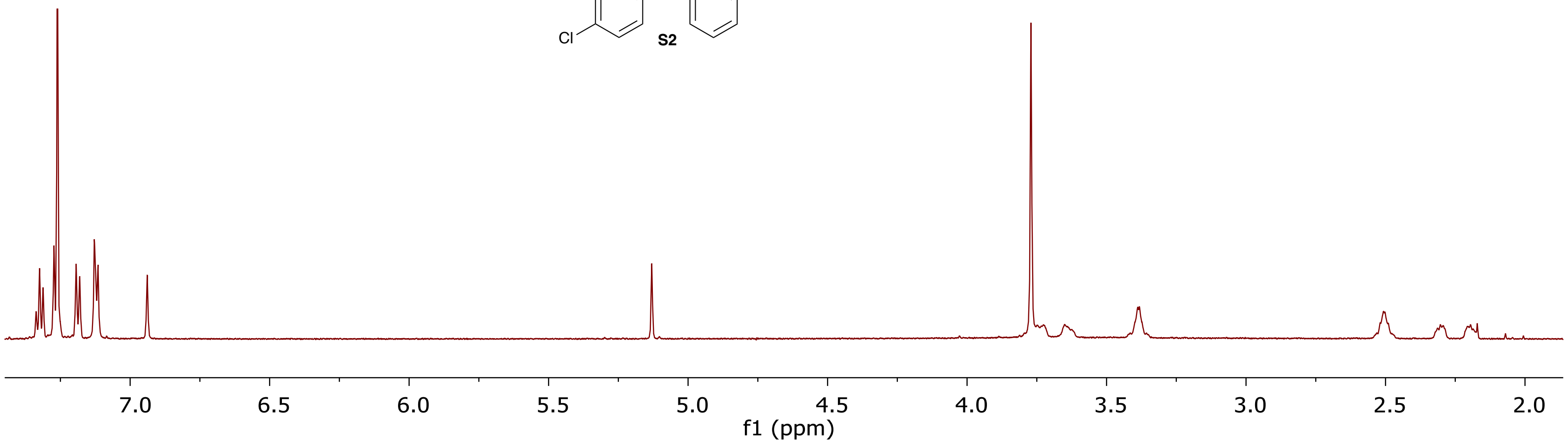

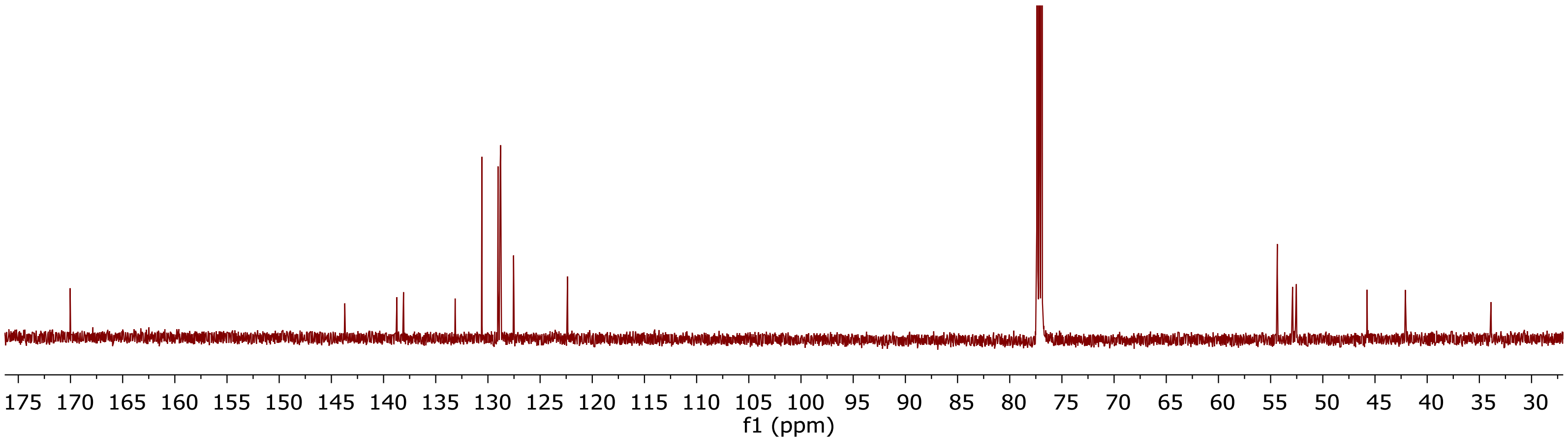

^1H and ^{13}C NMR Spectra for **S3**

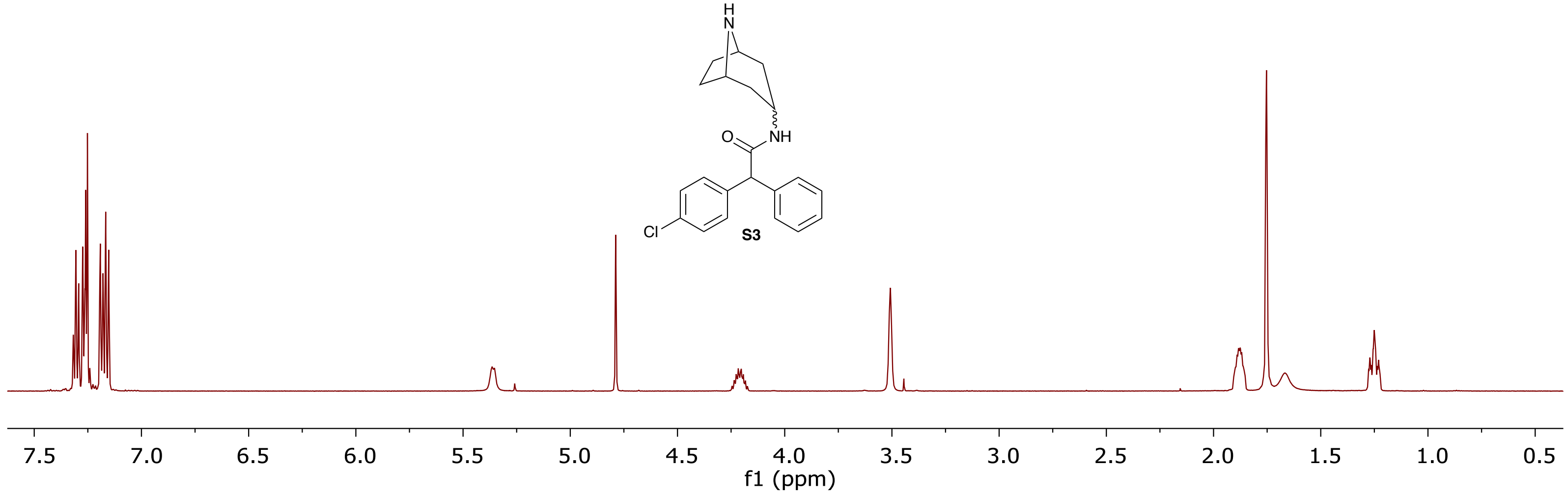

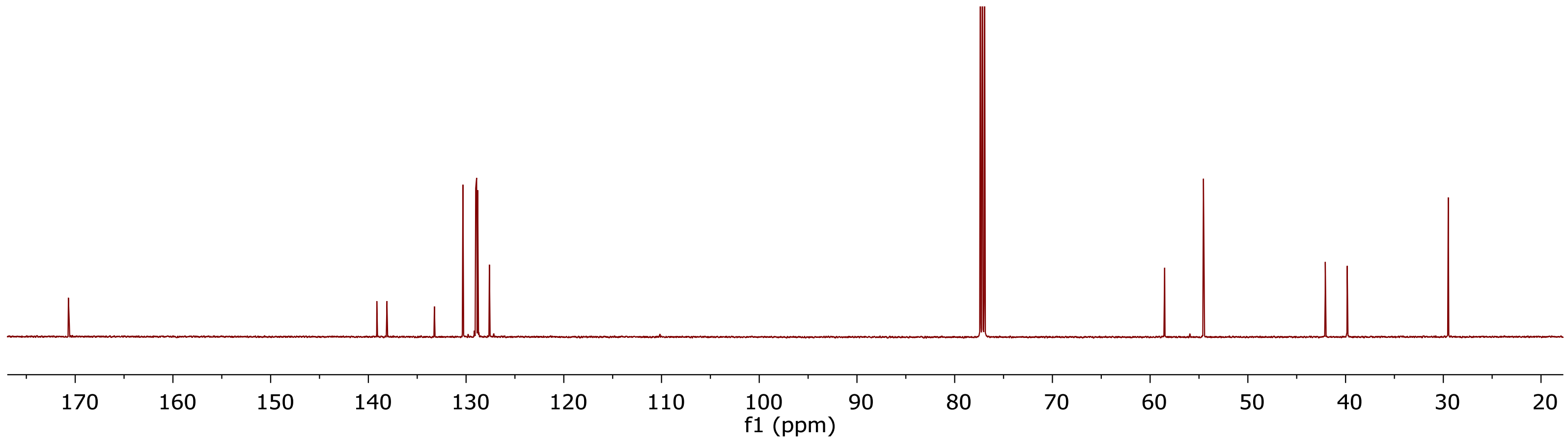

^{1}H and ^{13}C NMR Spectra for **24**

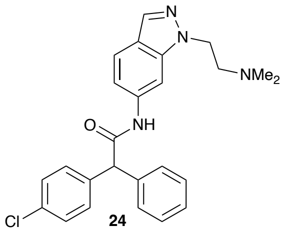

24

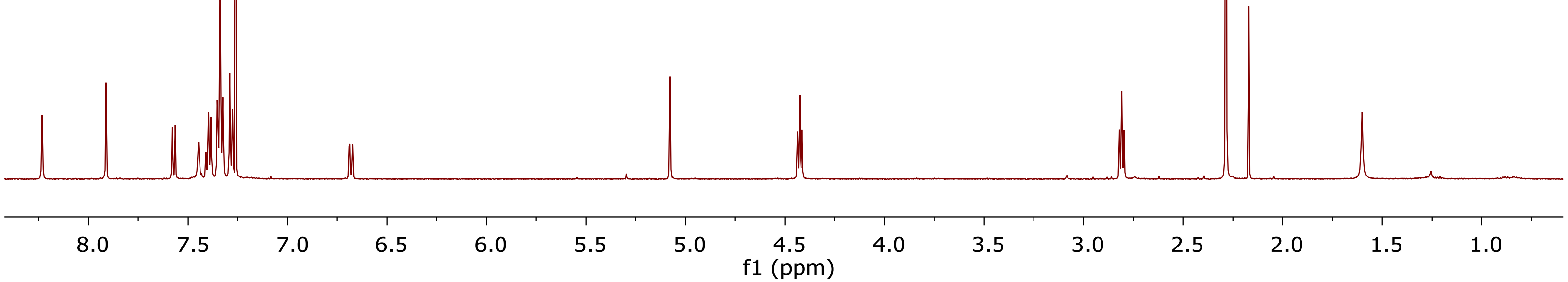

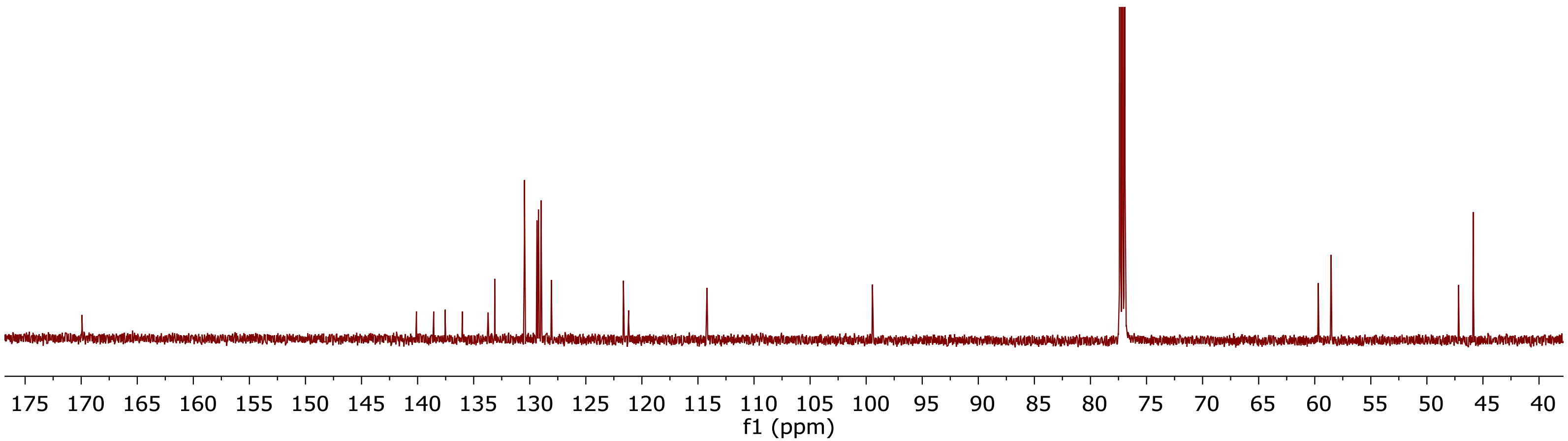

^{1}H and ^{13}C NMR Spectra for **S4**

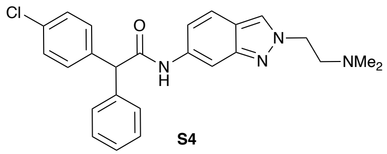

S4

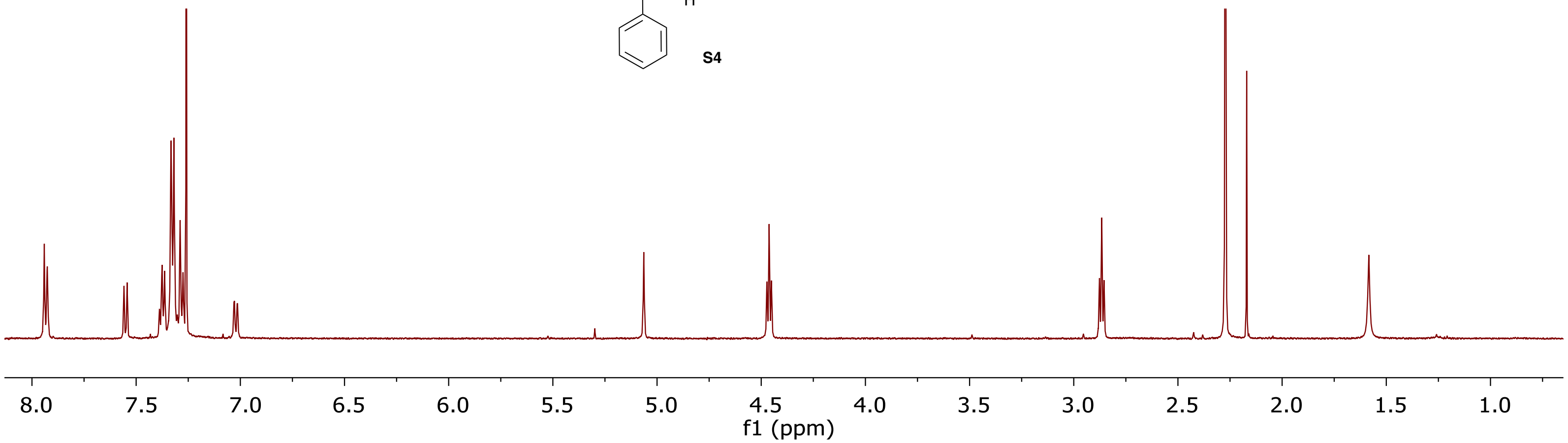

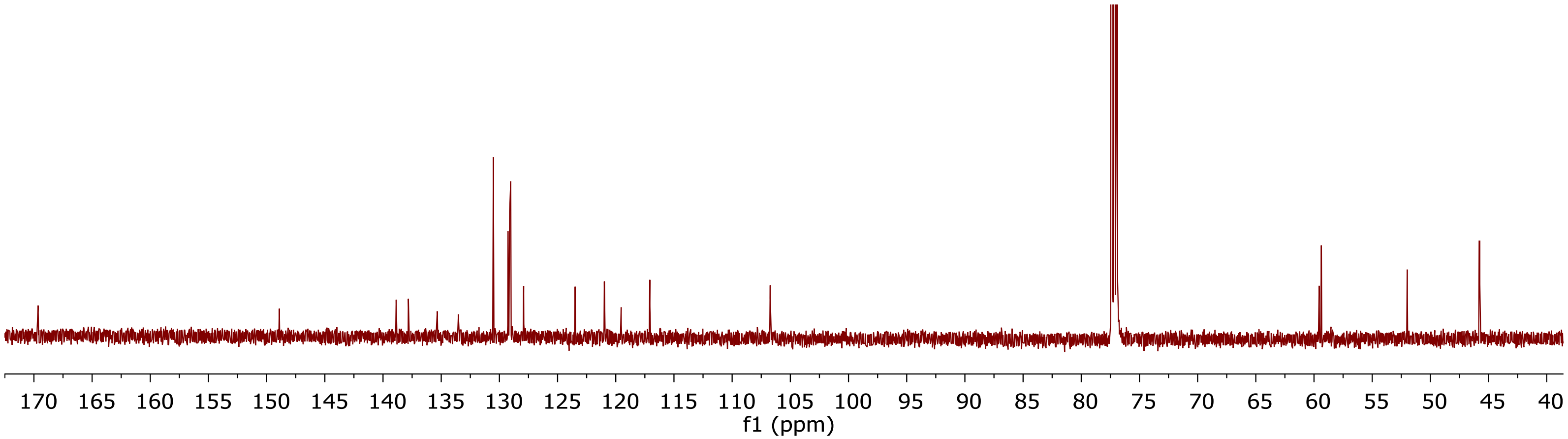

^{1}H and ^{13}C NMR Spectra for **S5**

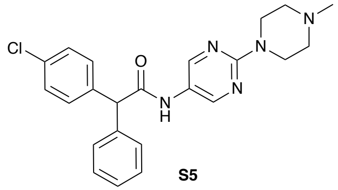

S5

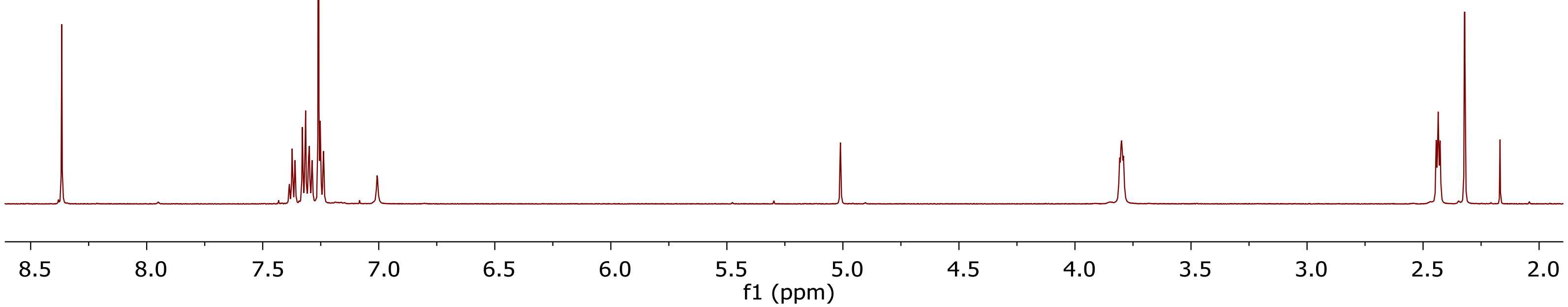

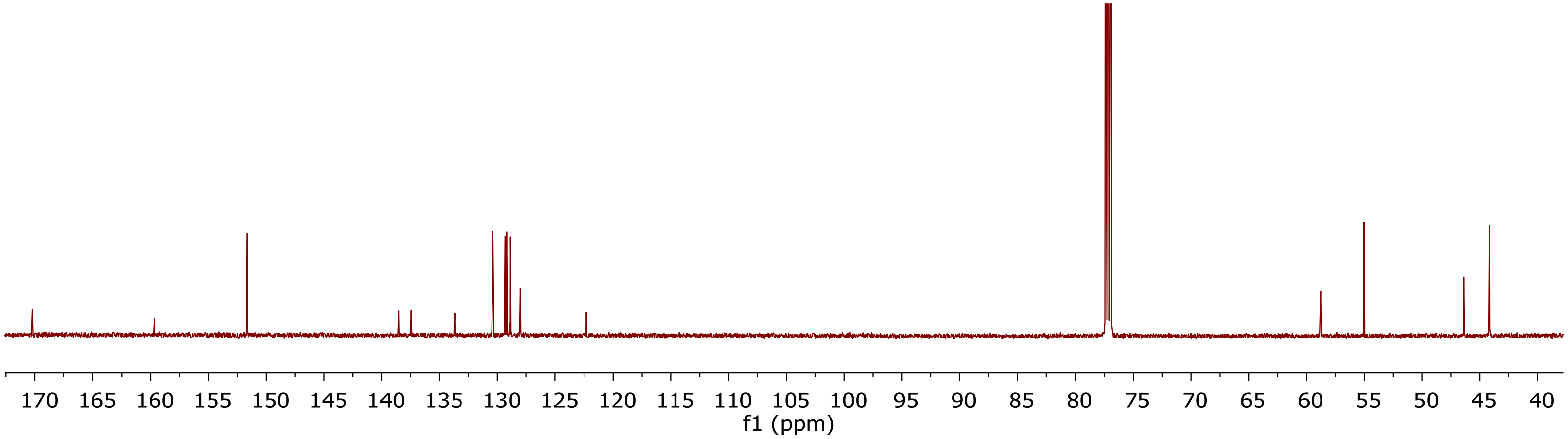

^{1}H and ^{13}C NMR Spectra for **S6**

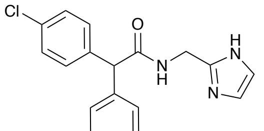

S6

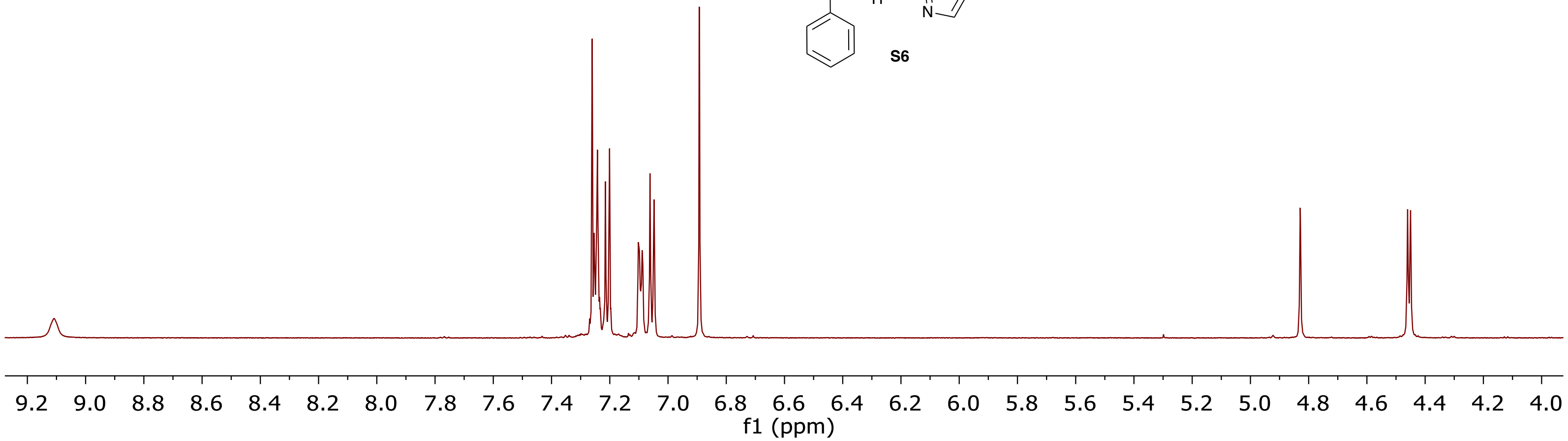

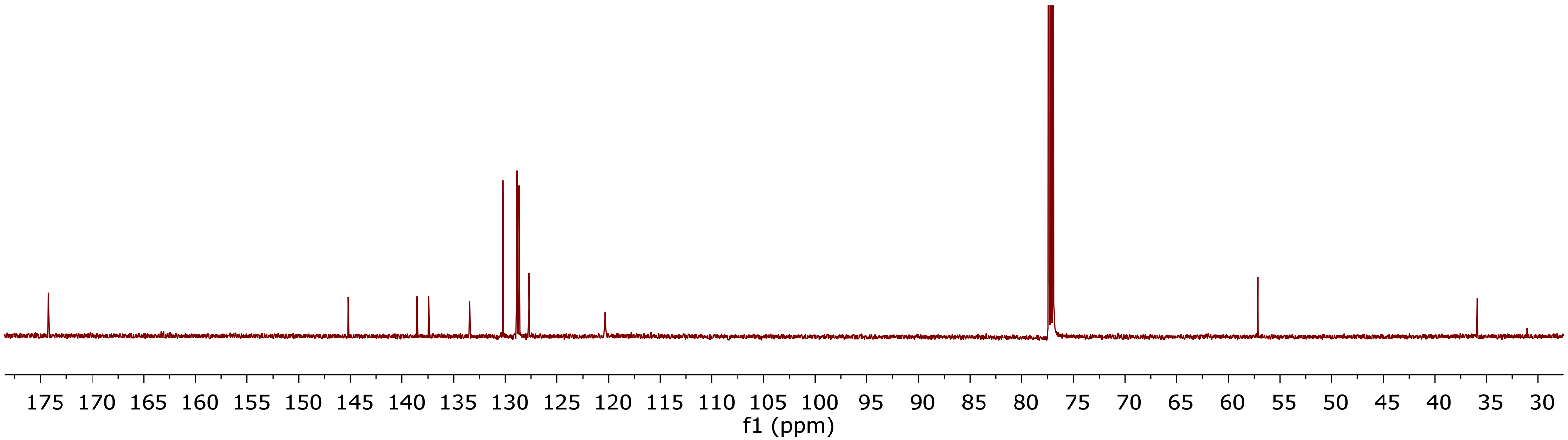

^{1}H and ^{13}C NMR Spectra for **S7**

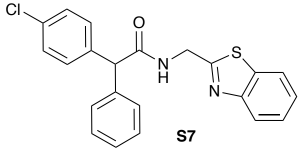

S7

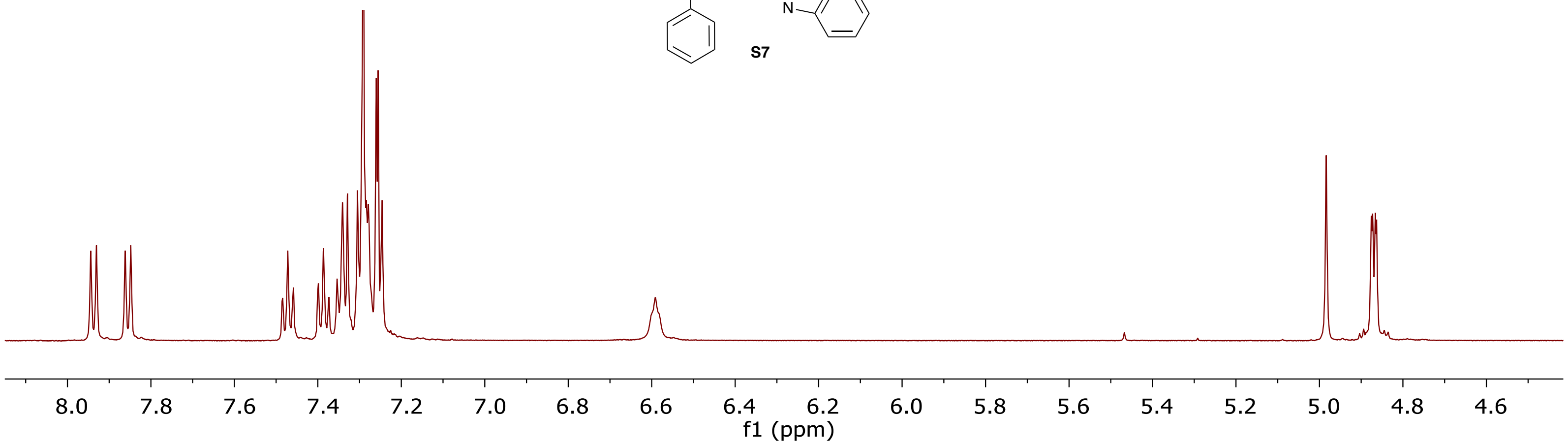

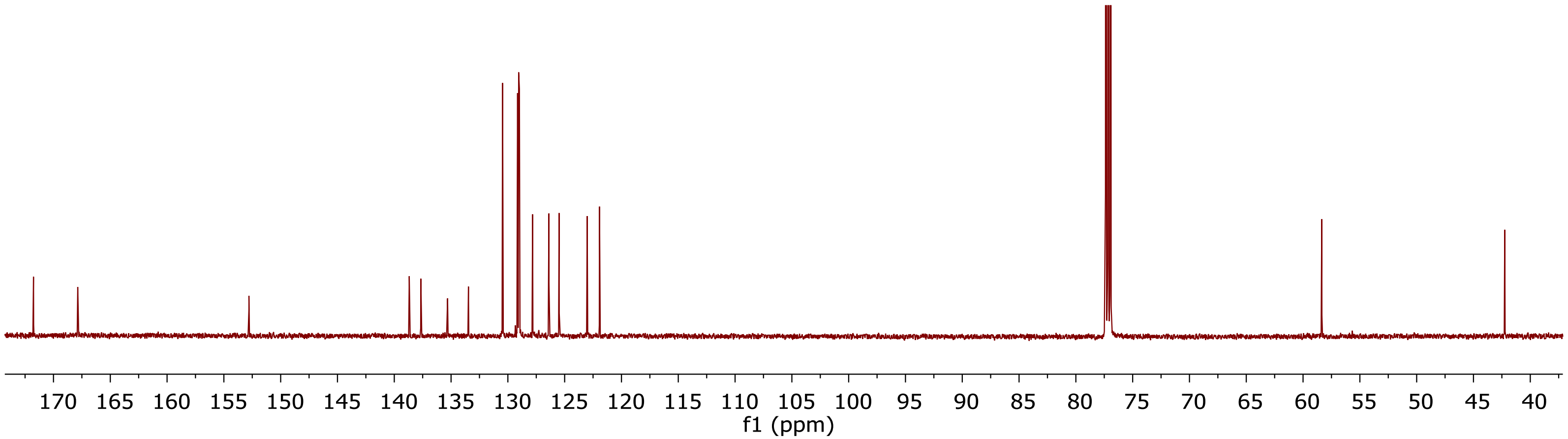

^{1}H and ^{13}C NMR Spectra for **S8**

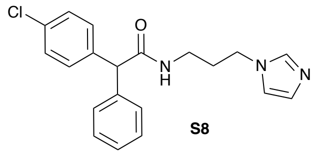

S8

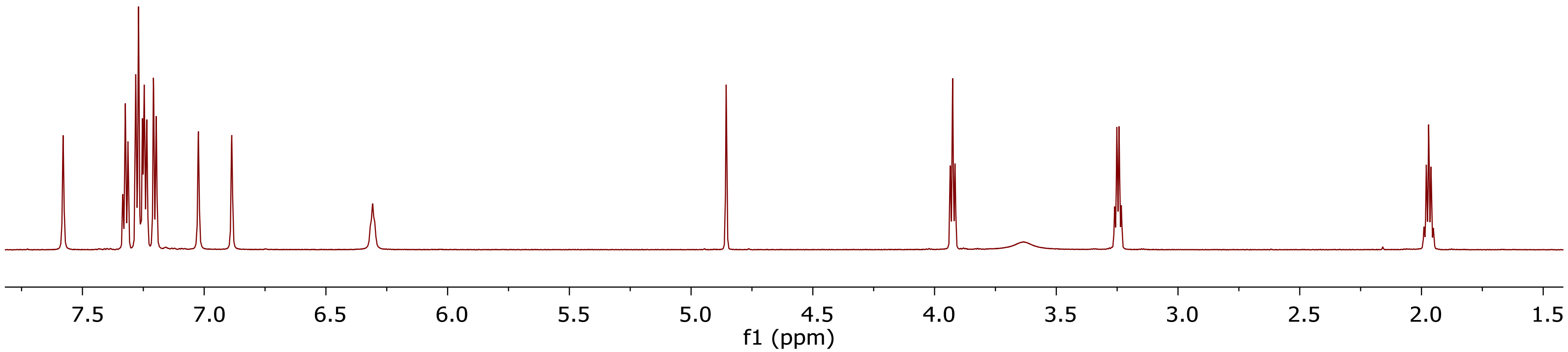

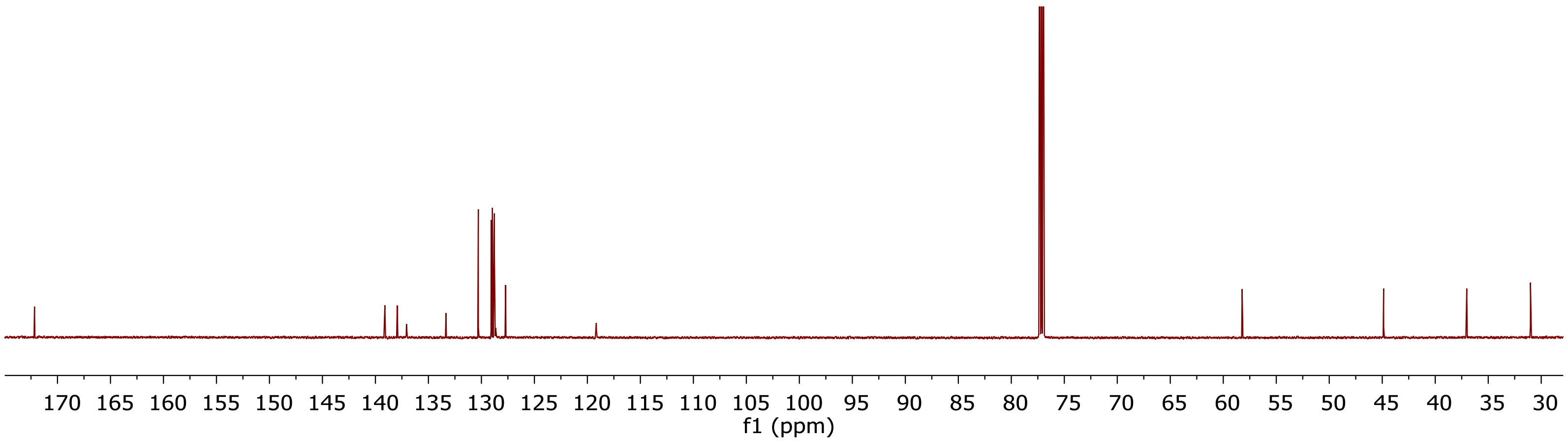

^{1}H and ^{13}C NMR Spectra for **S9**

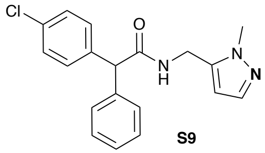

S9

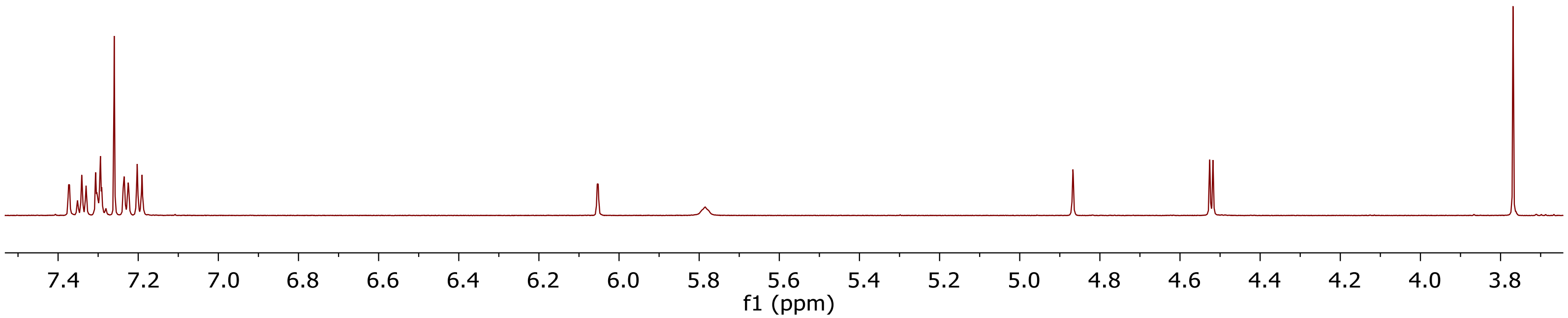

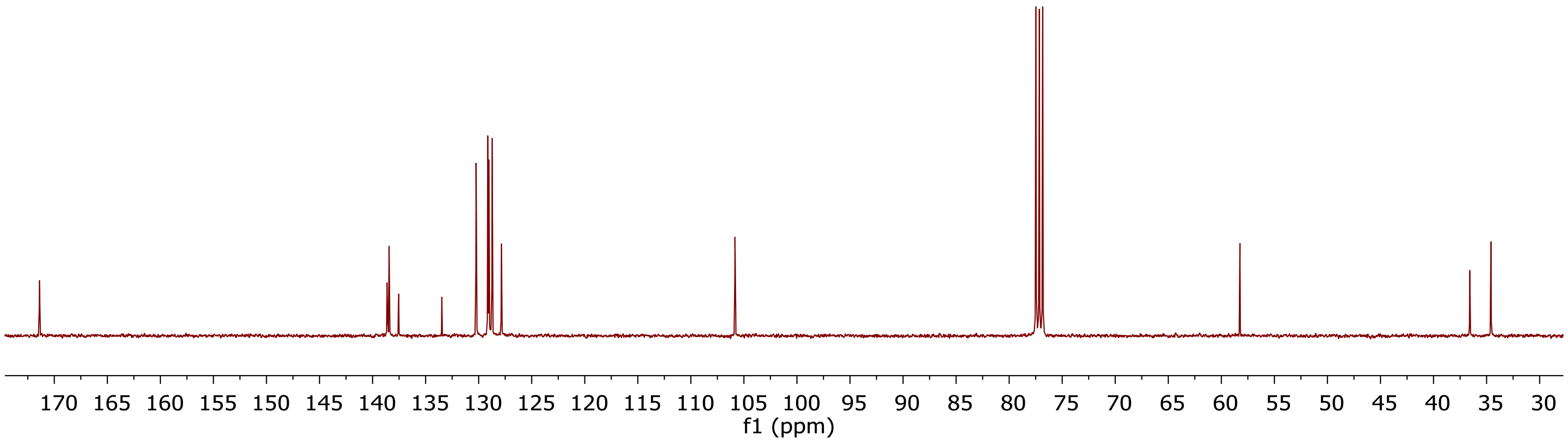

^{1}H and ^{13}C NMR Spectra for **S10**

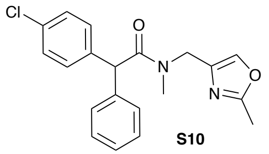

S10

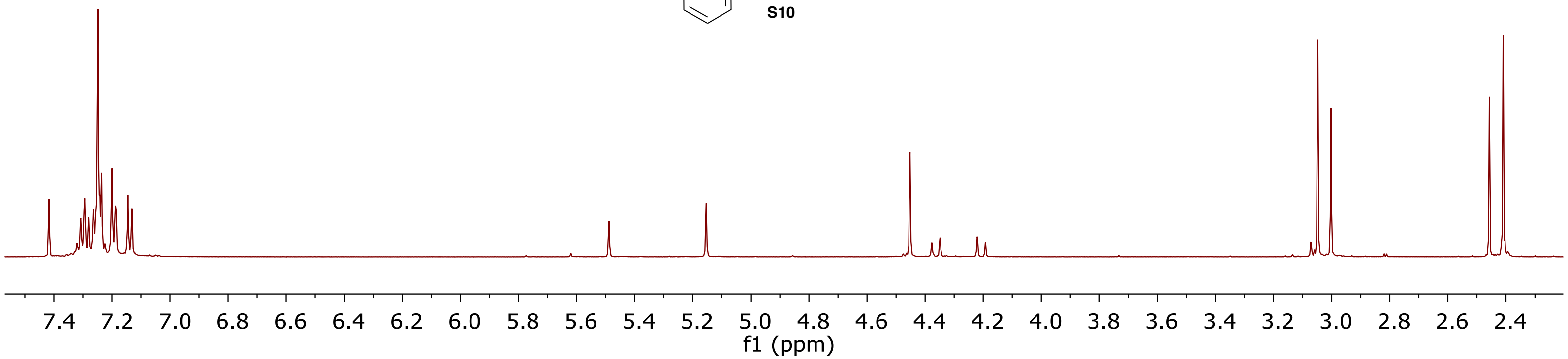

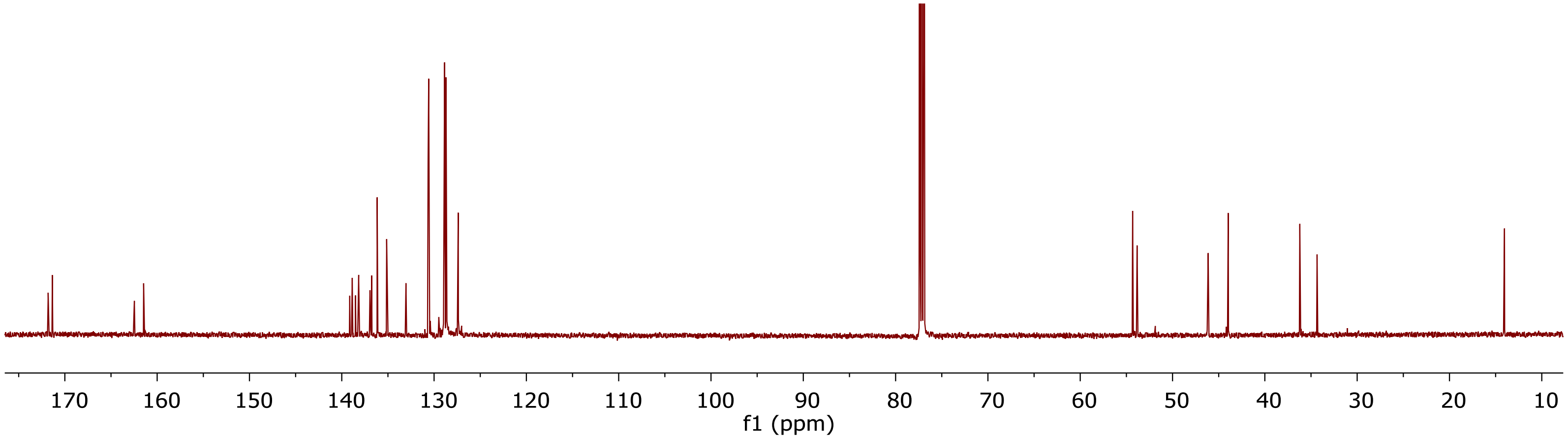

^{1}H and ^{13}C NMR Spectra for **S11**

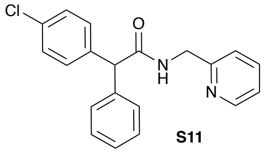

S11

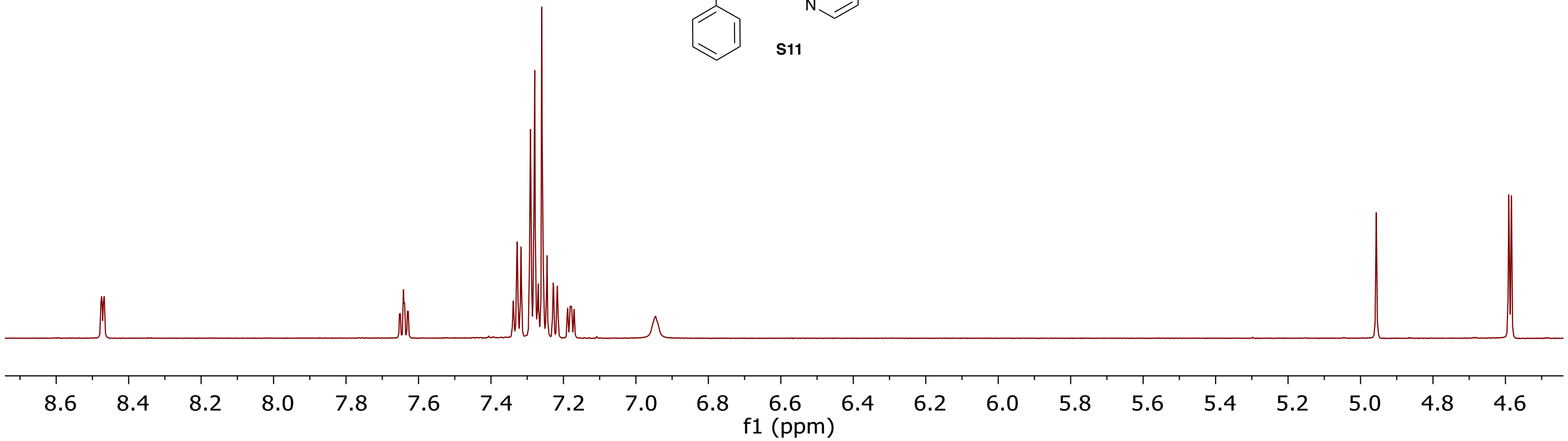

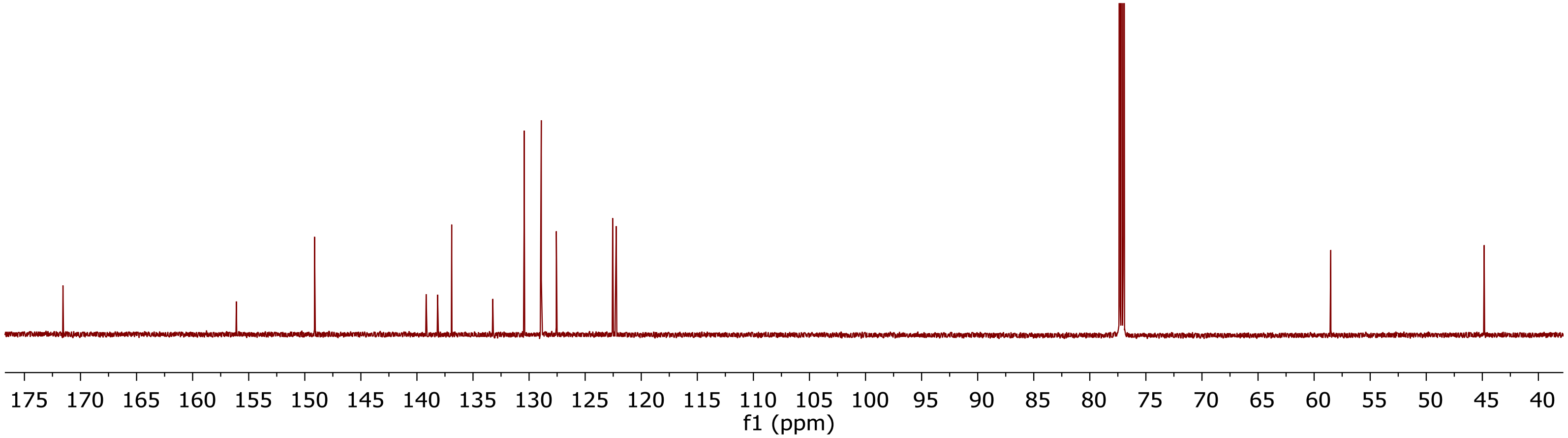

^{1}H and ^{13}C NMR Spectra for **S12**

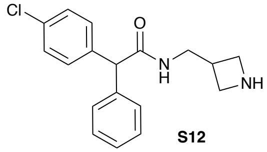

S12

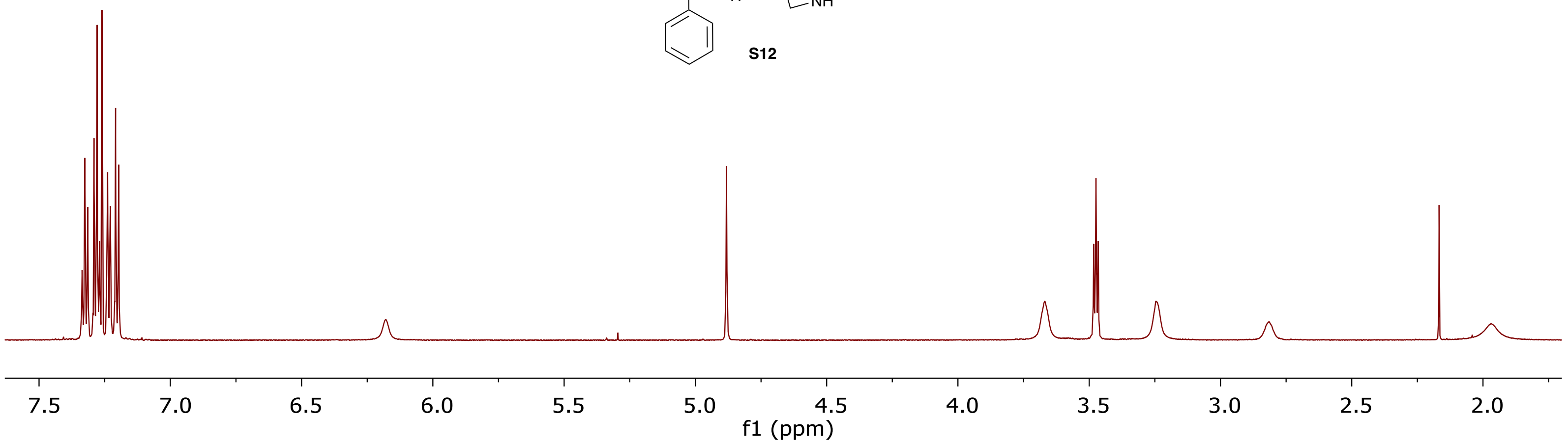

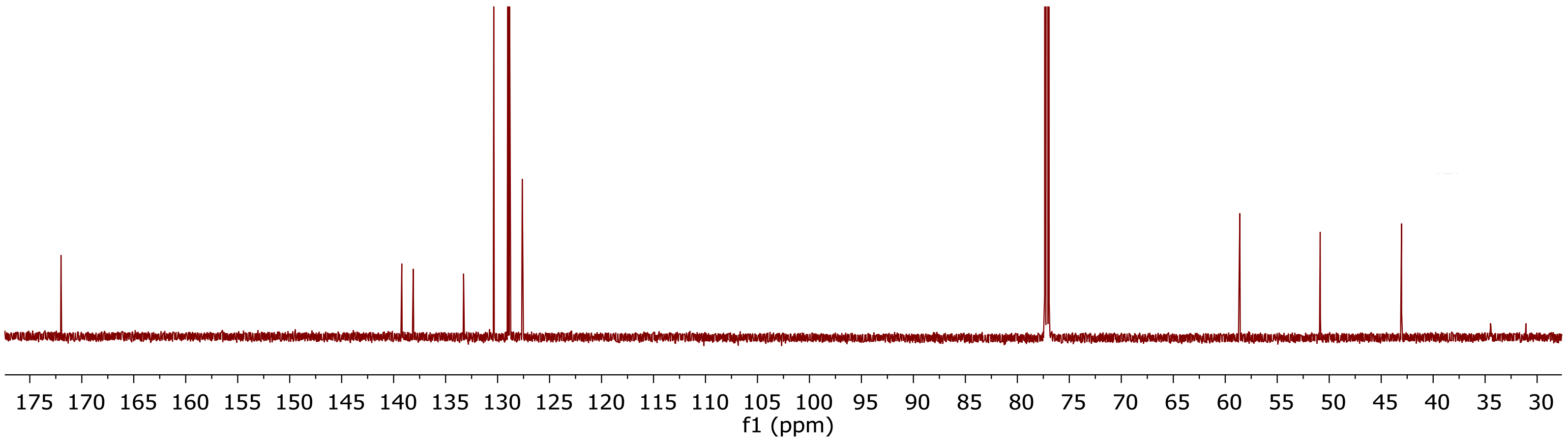

^{1}H and ^{13}C NMR Spectra for **S13**

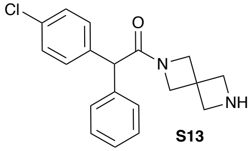

S13

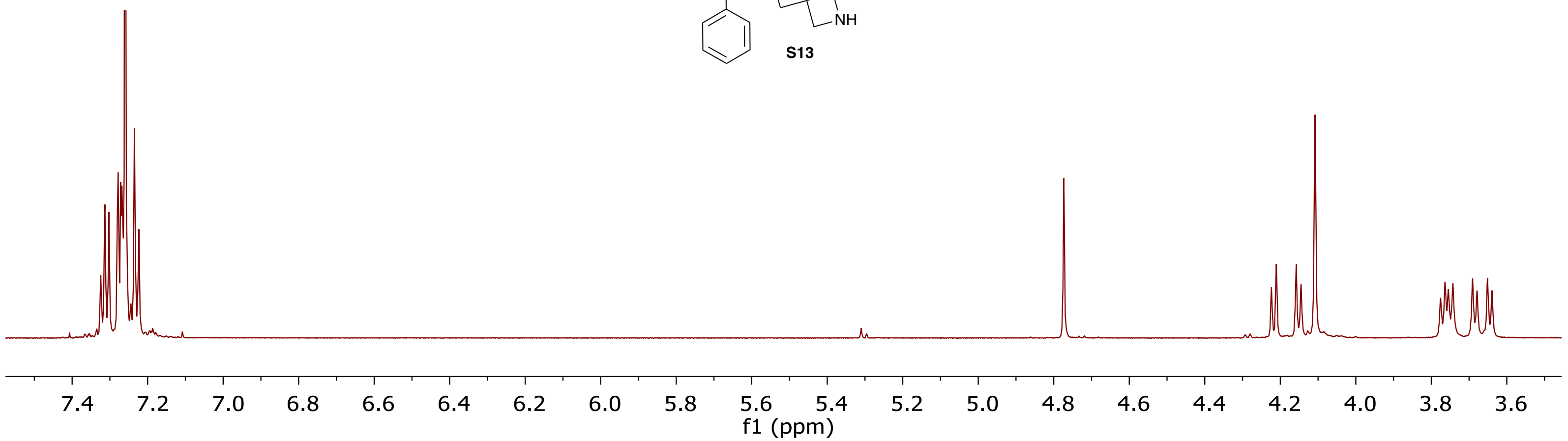

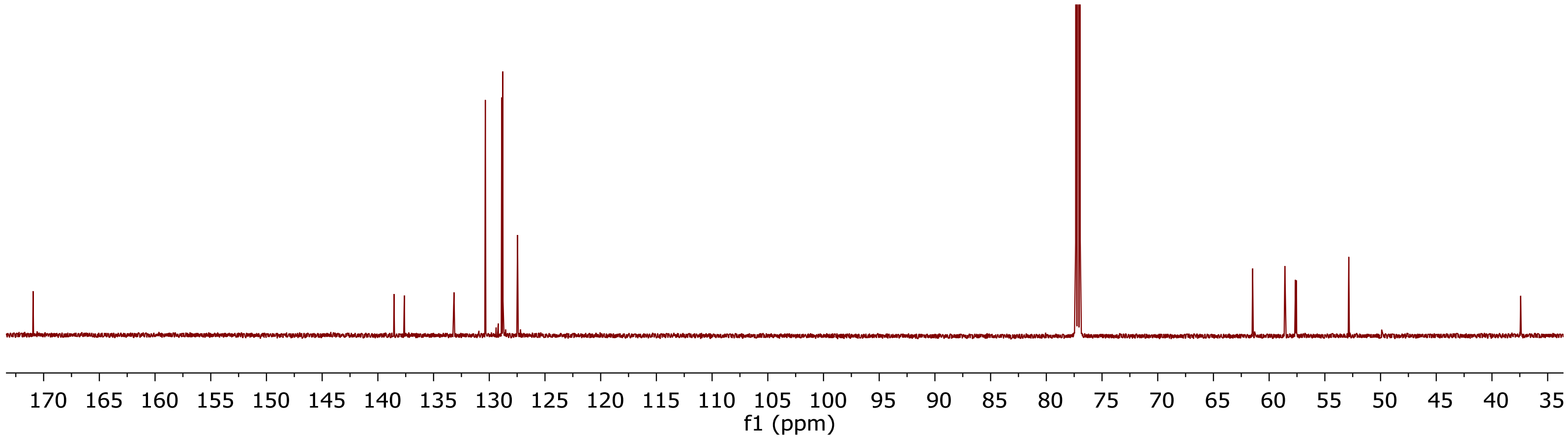

^1H and ^{13}C NMR Spectra for **S14**

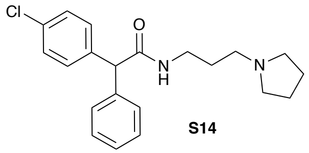

S14

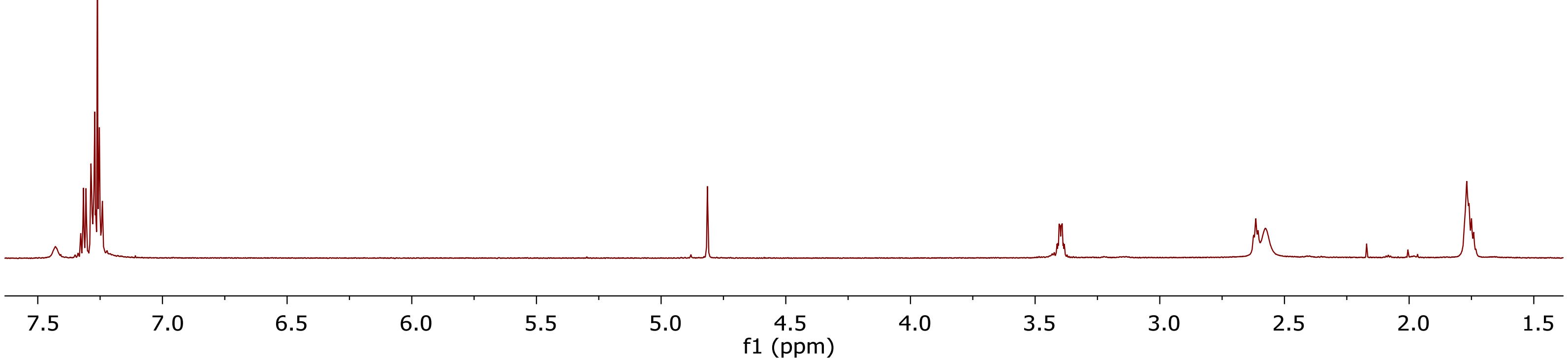

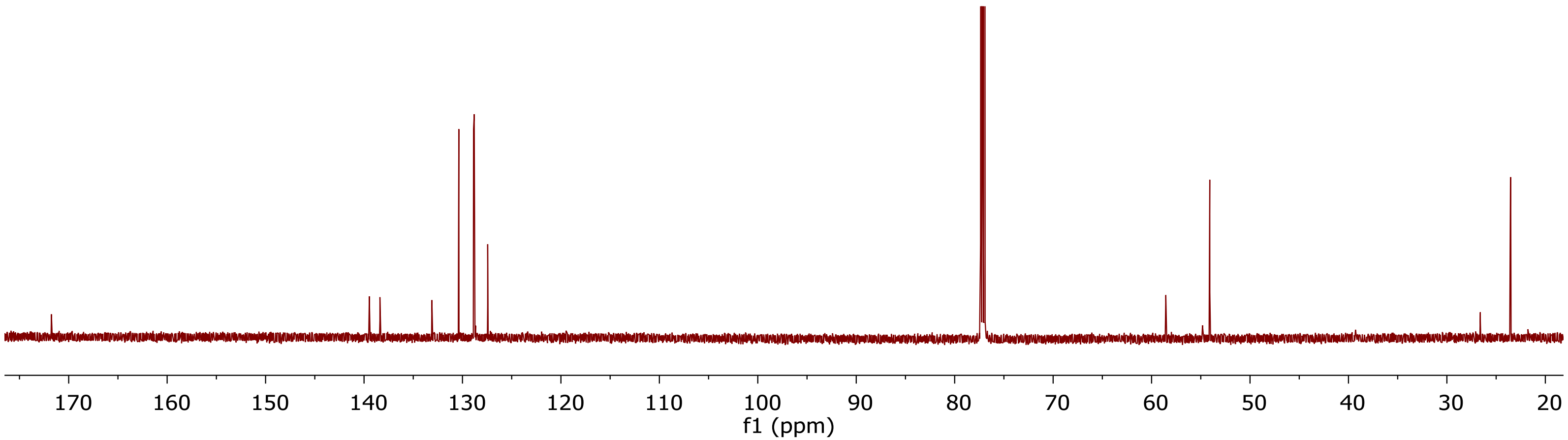

^{1}H and ^{13}C NMR Spectra for **S15**

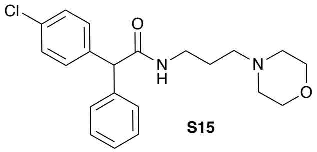

S15

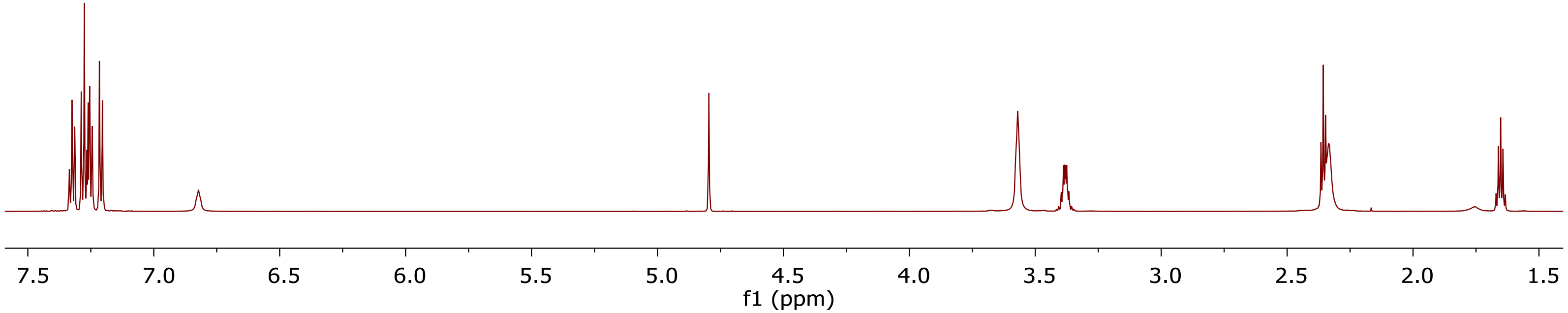

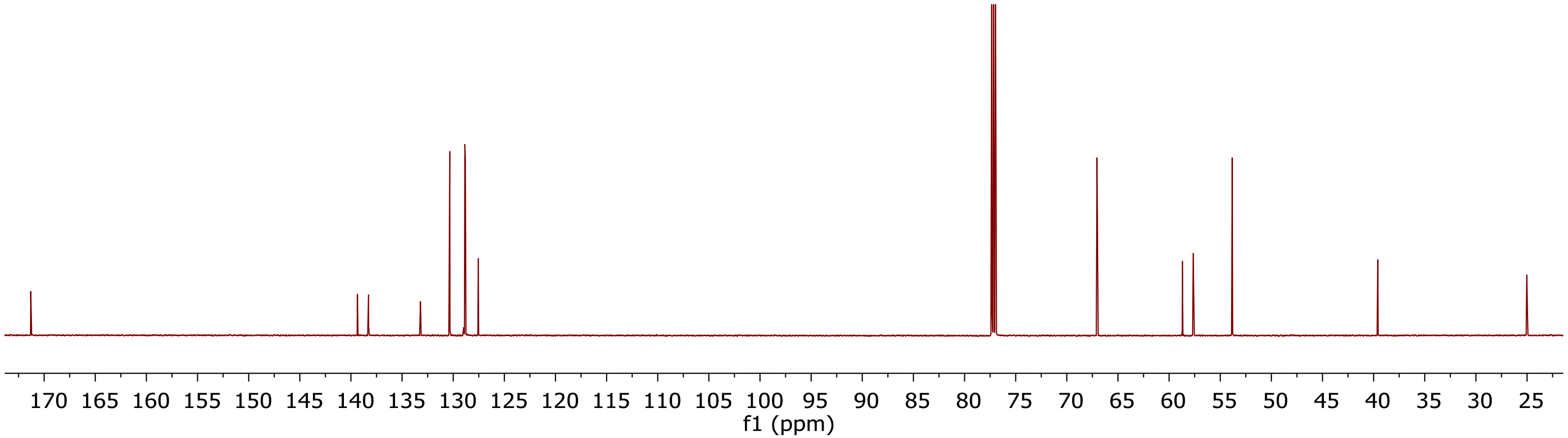

^{1}H and ^{13}C NMR Spectra for **S16**

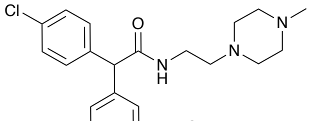

S16

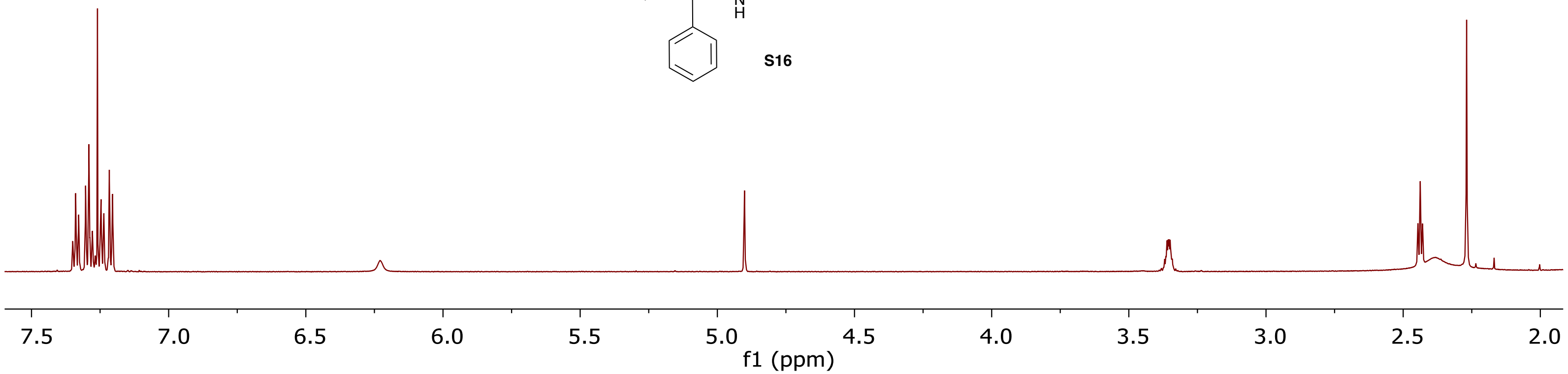

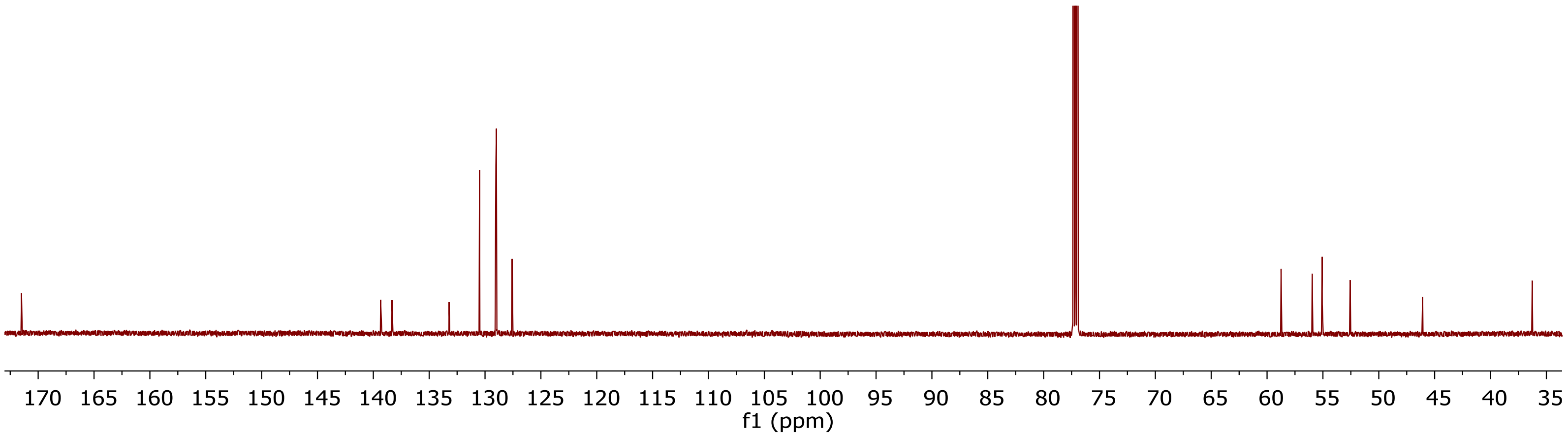

^{1}H and ^{13}C NMR Spectra for **S17**

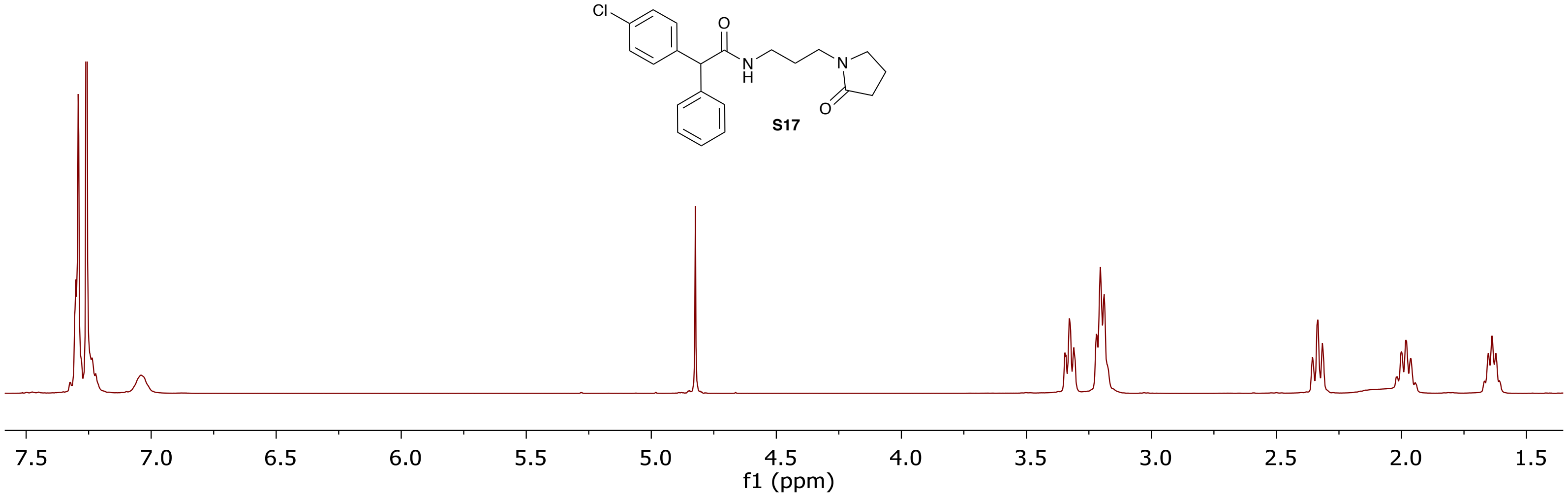

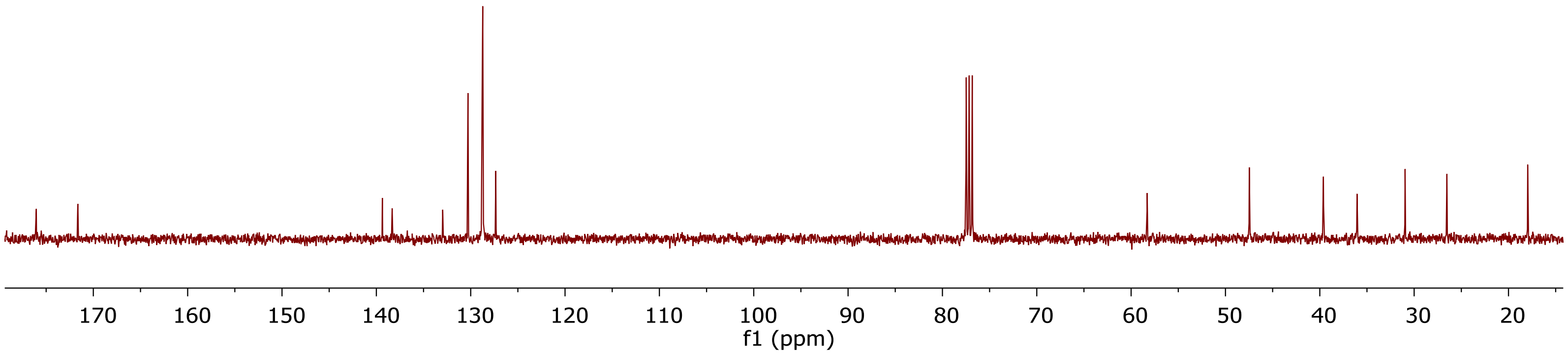

^{1}H and ^{13}C NMR Spectra for **S18**

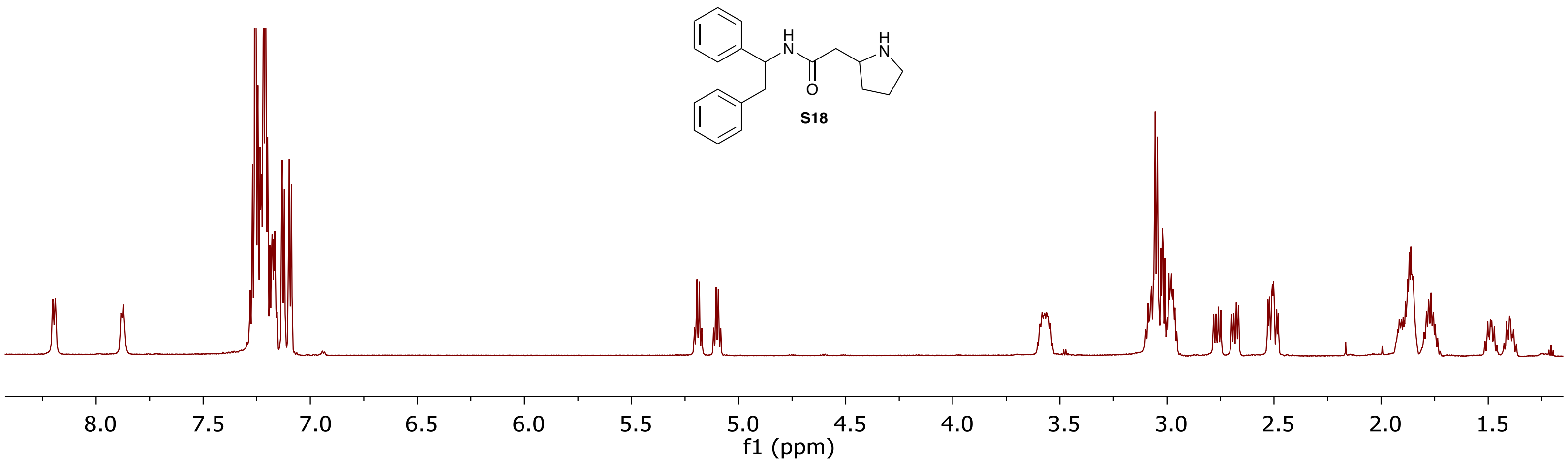

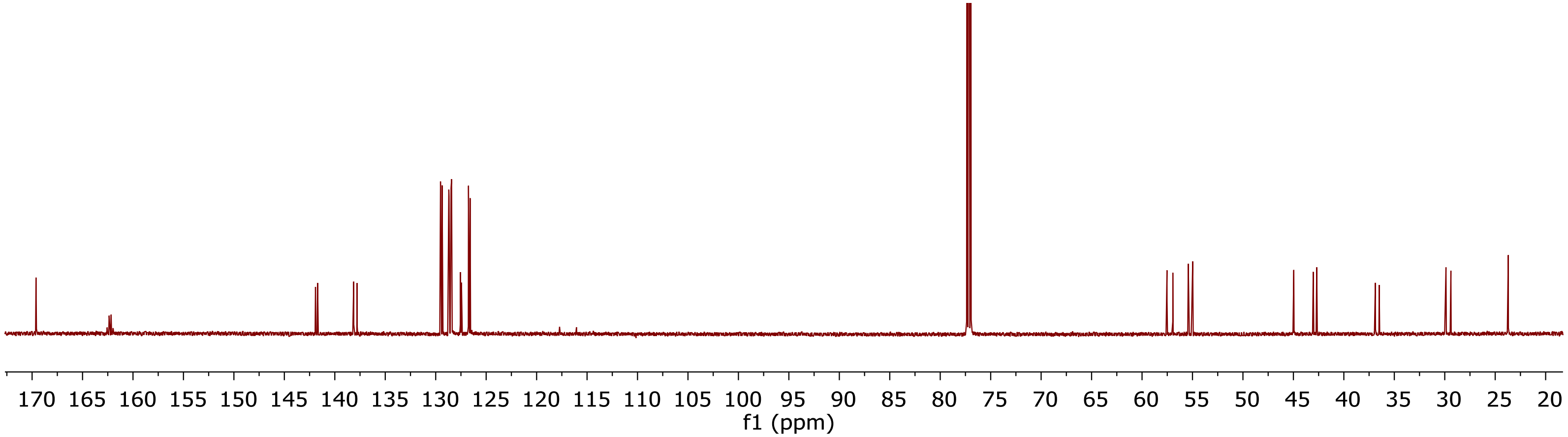

^{1}H and ^{13}C NMR Spectra for **S19**

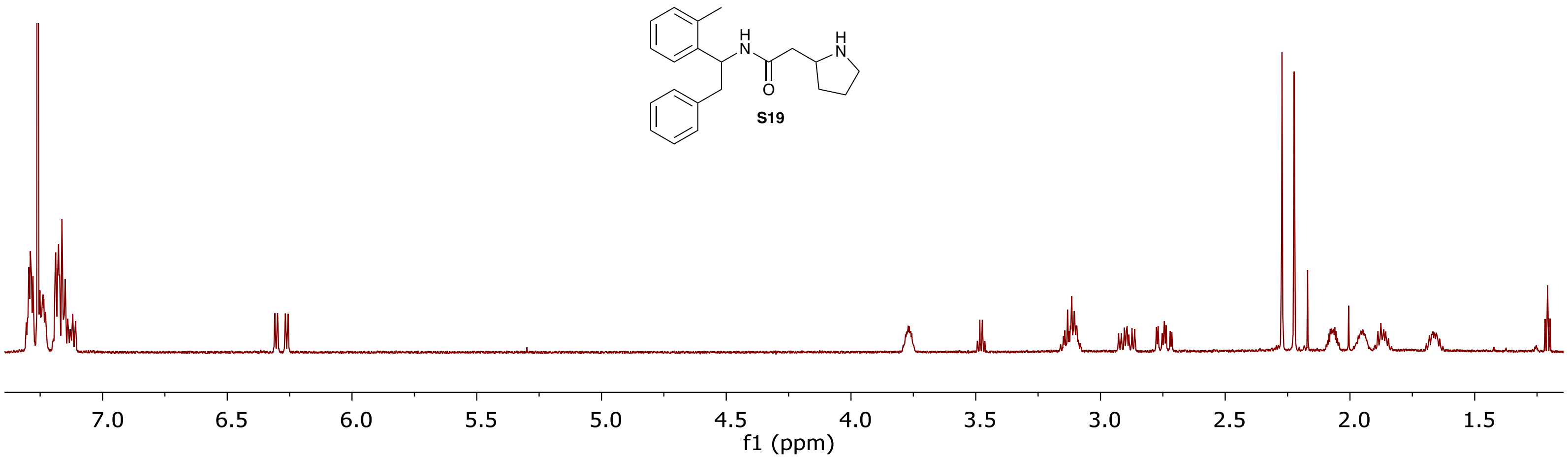

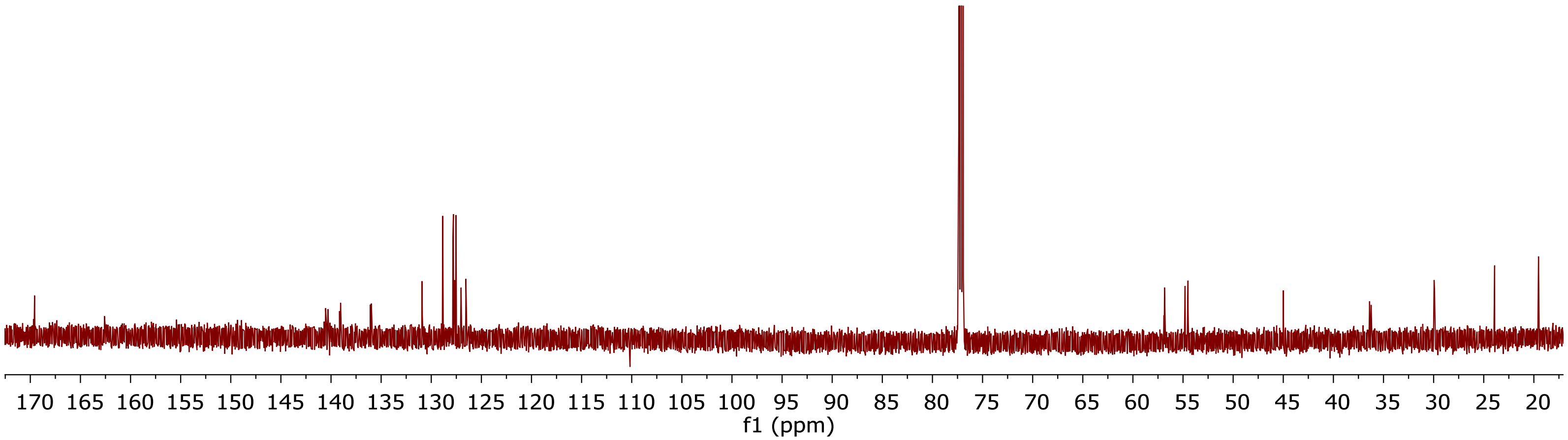

^{1}H and ^{13}C NMR Spectra for **S20**

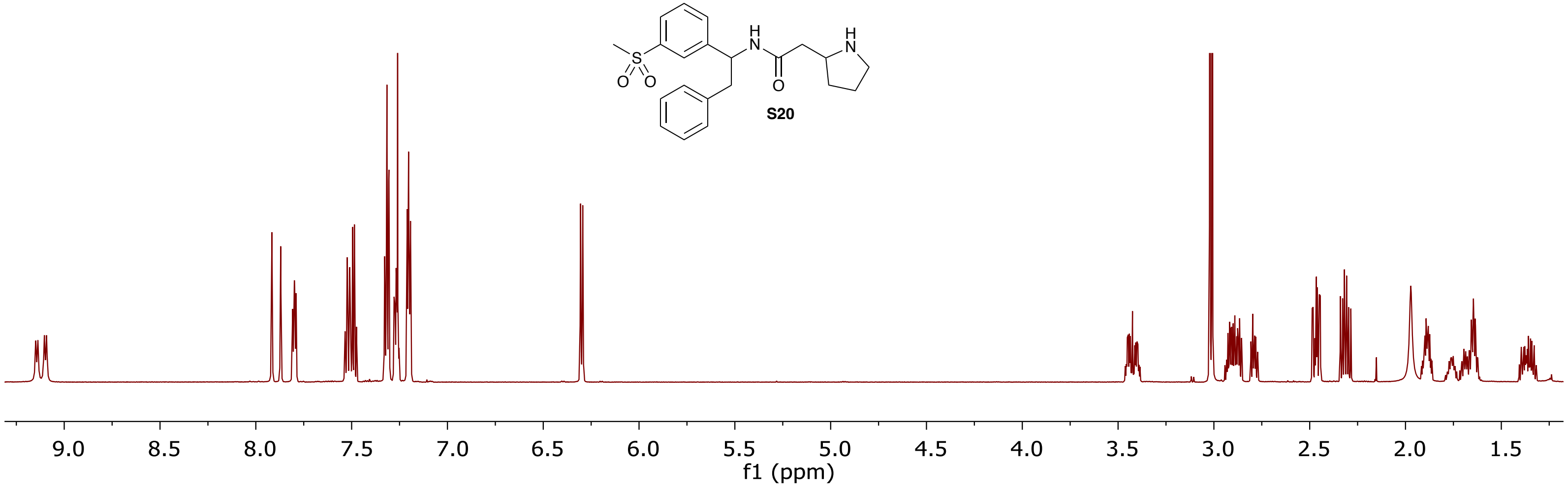

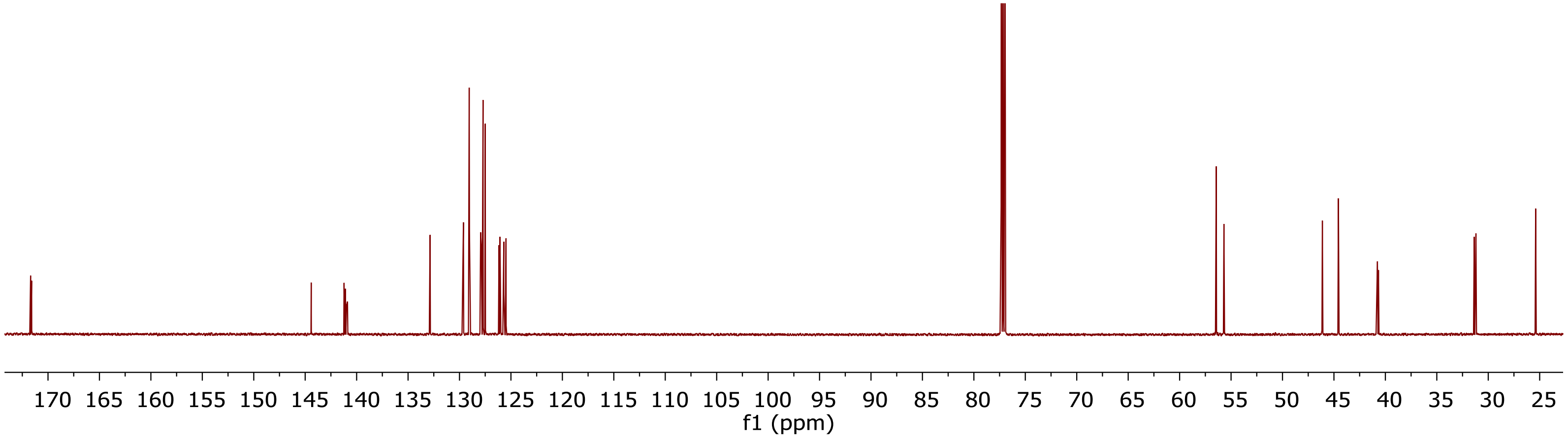

^{1}H and ^{13}C NMR Spectra for **S21**

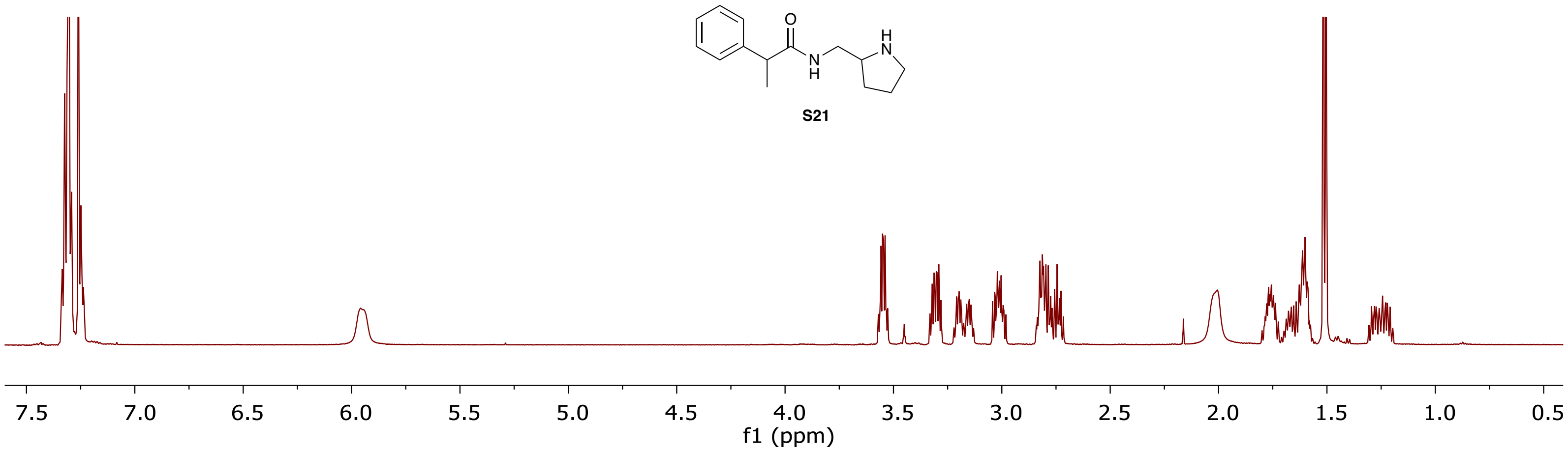

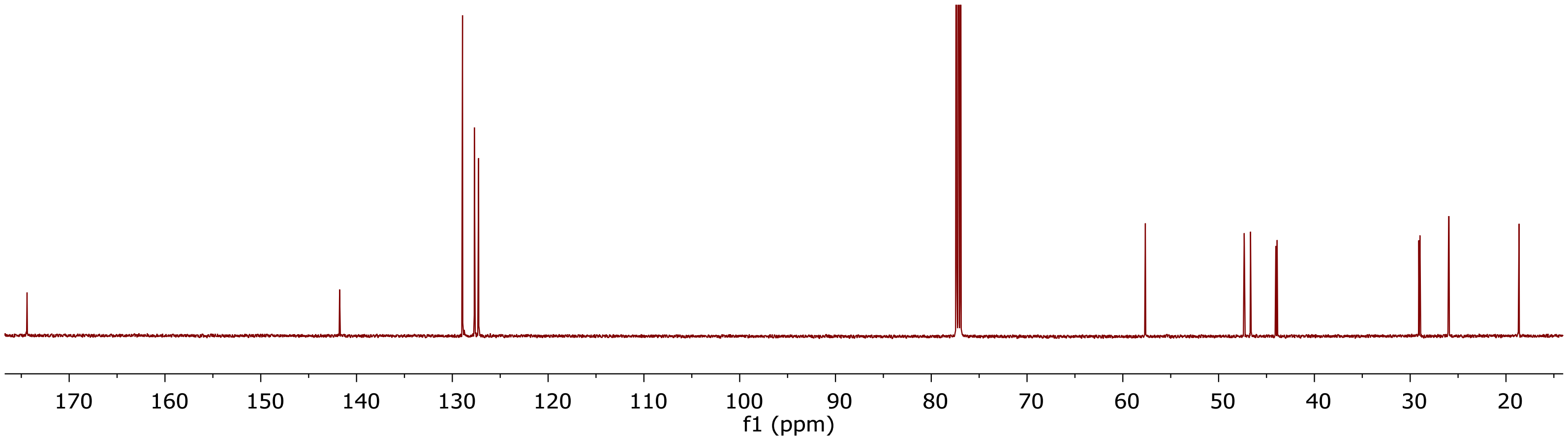

^{1}H and ^{13}C NMR Spectra for **S23**

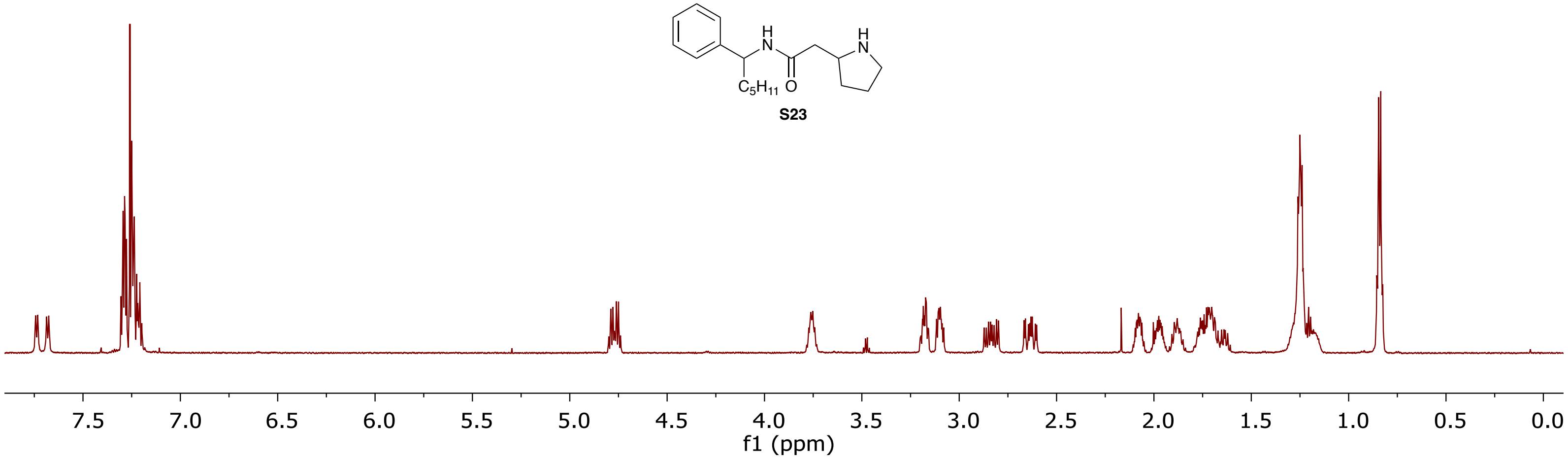

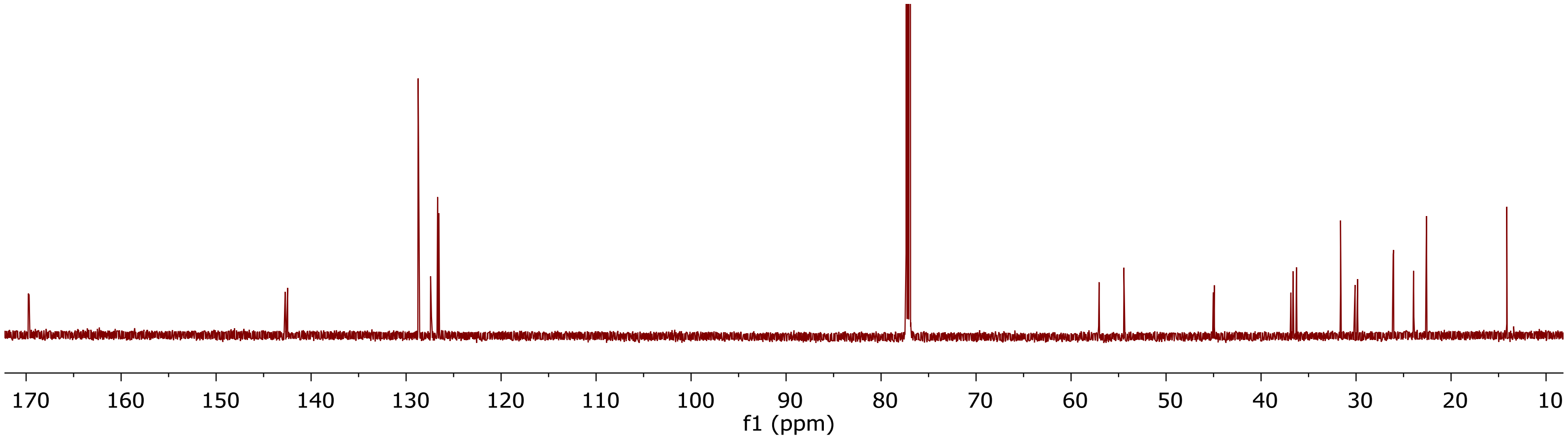

^{1}H and ^{13}C NMR Spectra for **S24**

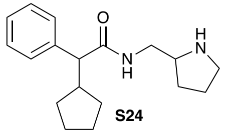

S24

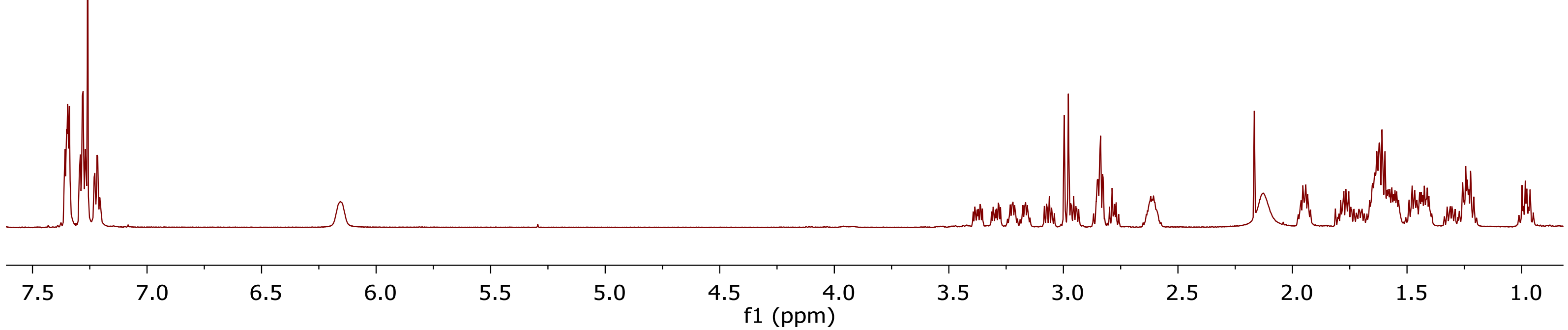

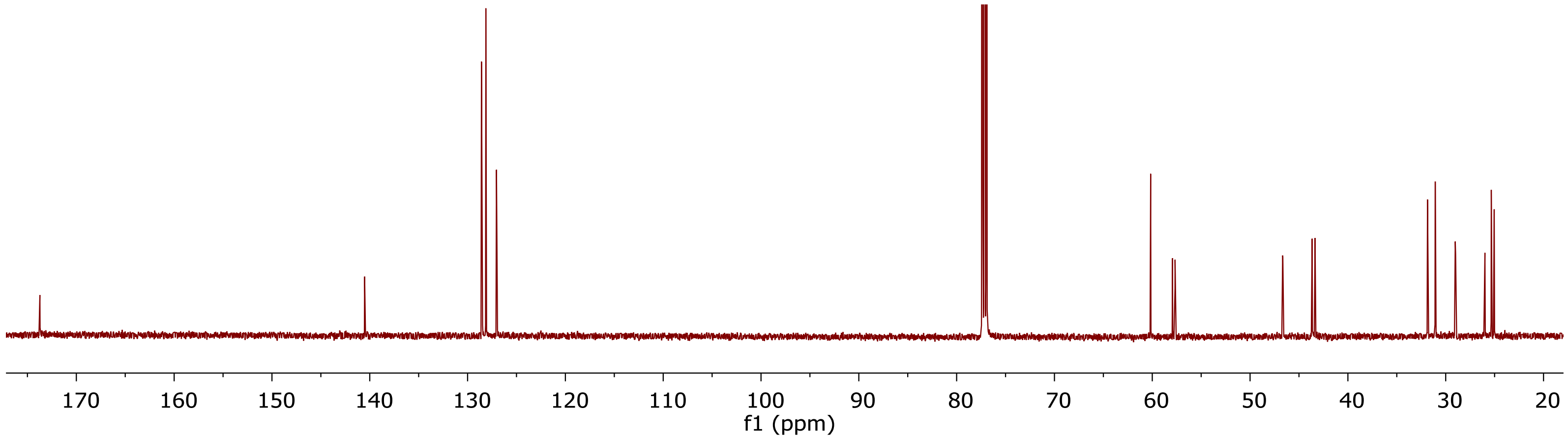

^{1}H and ^{13}C NMR Spectra for **S25**

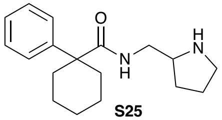

S25

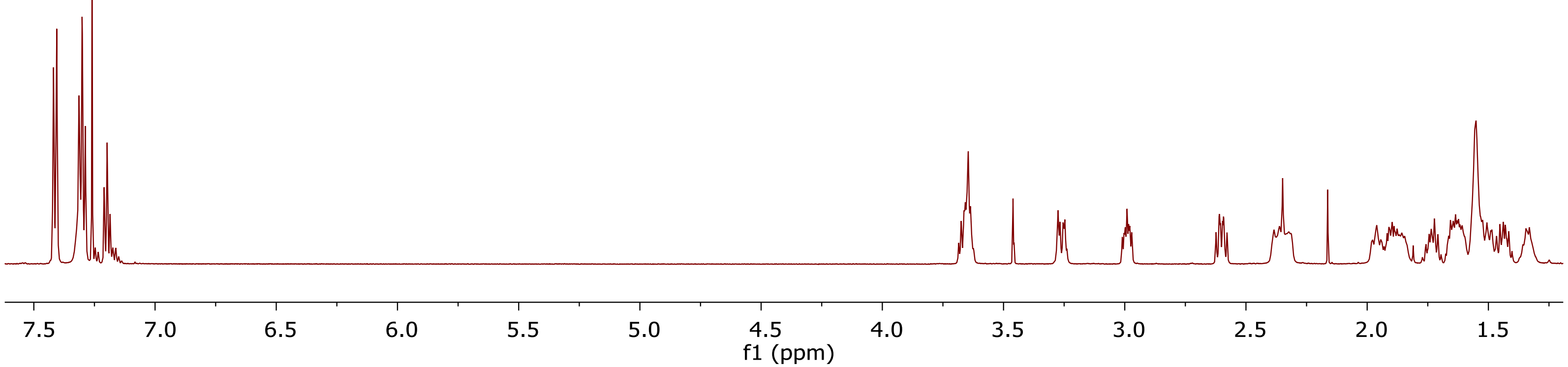

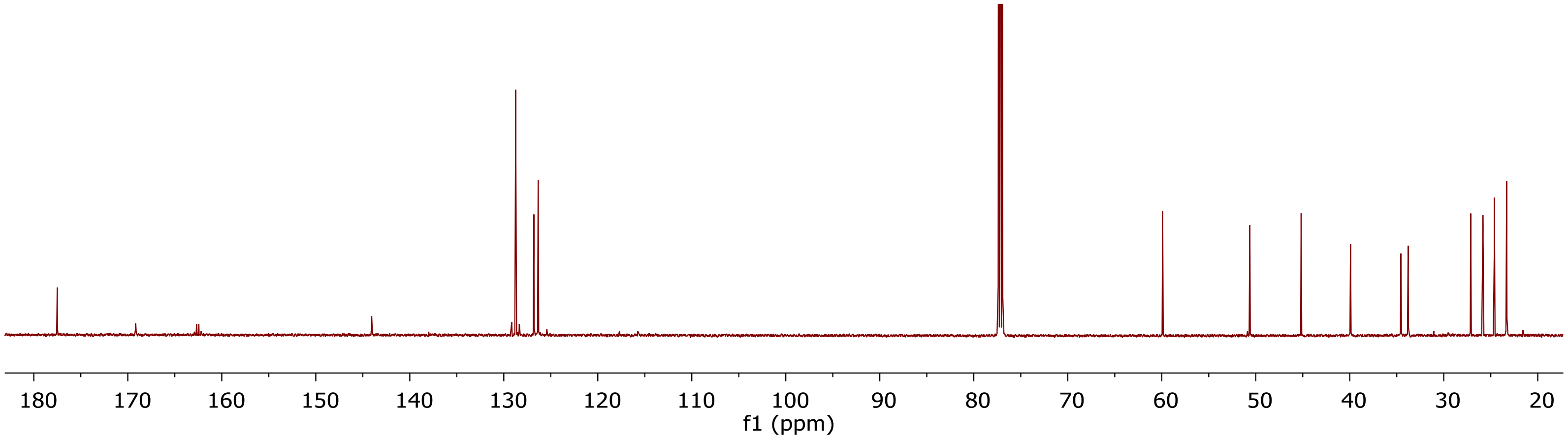

^{1}H and ^{13}C NMR Spectra for **S26**

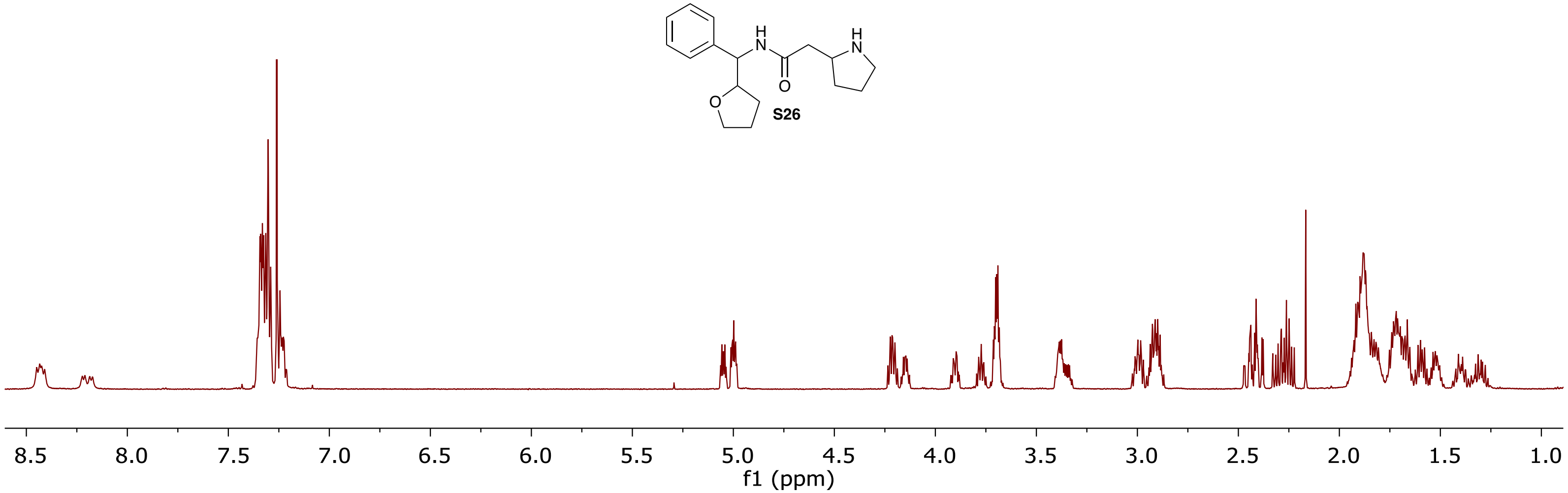

S26

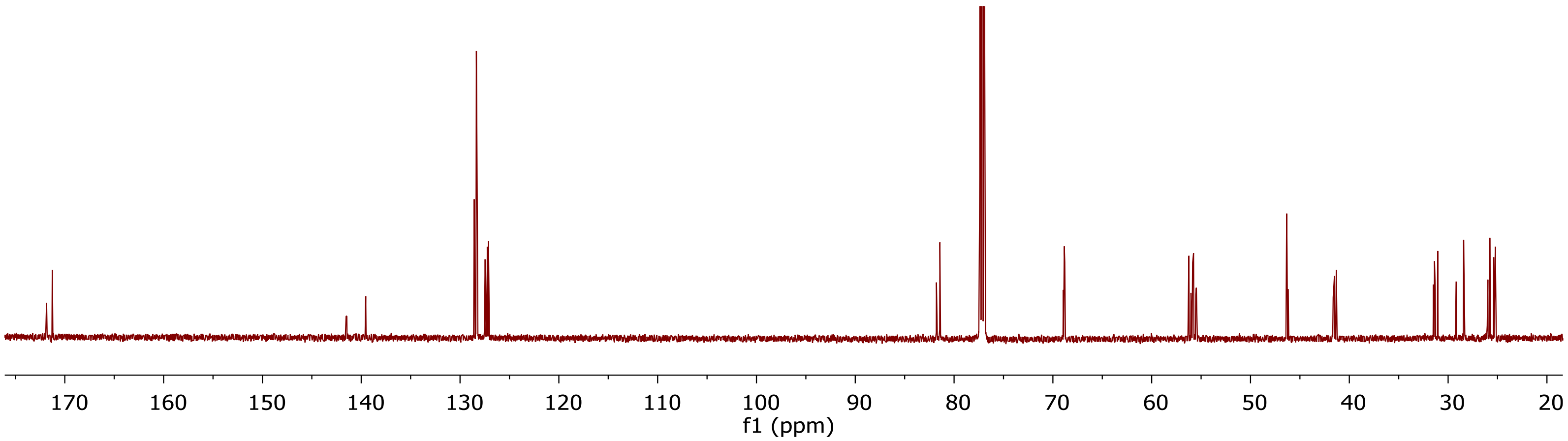

^{1}H and ^{13}C NMR Spectra for **S27**

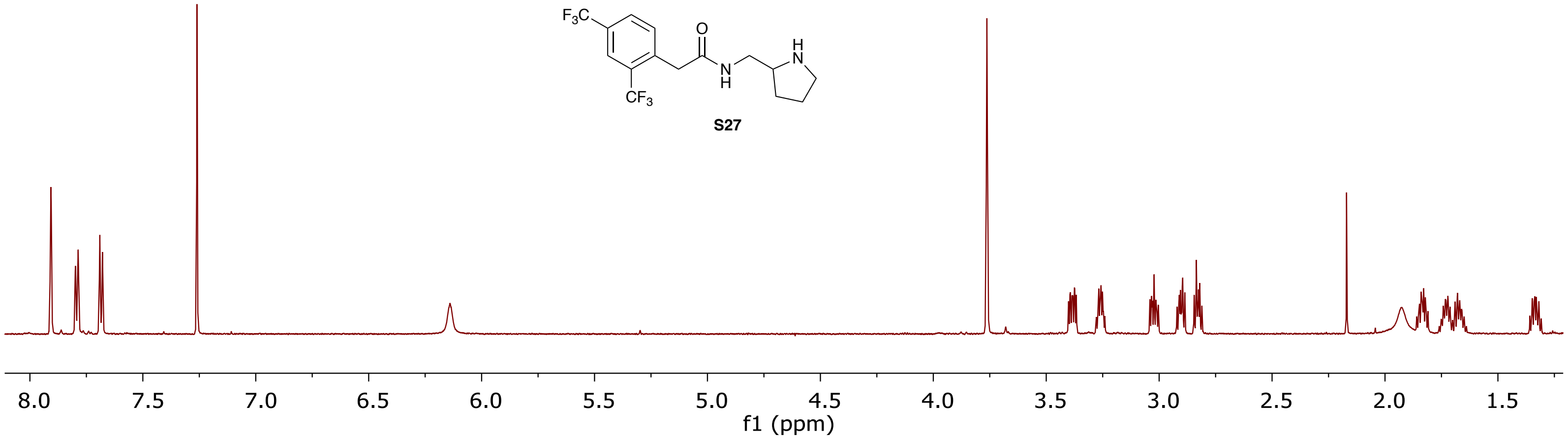

S27

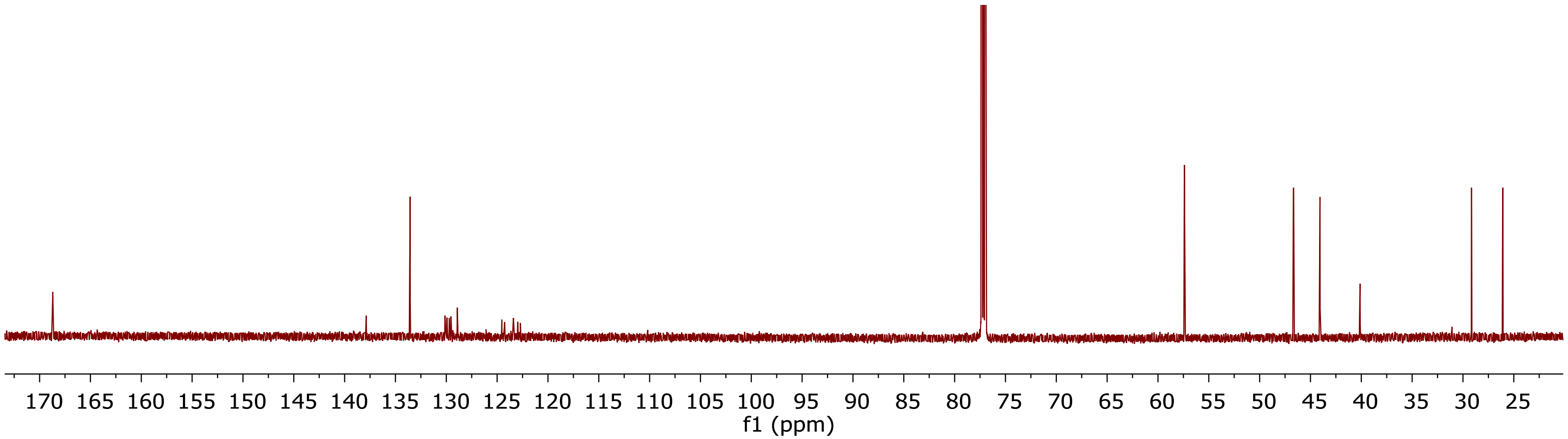

^1H and ^{13}C NMR Spectra for **S28**

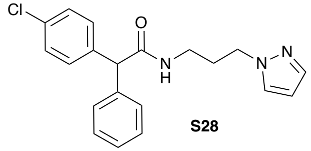

S28

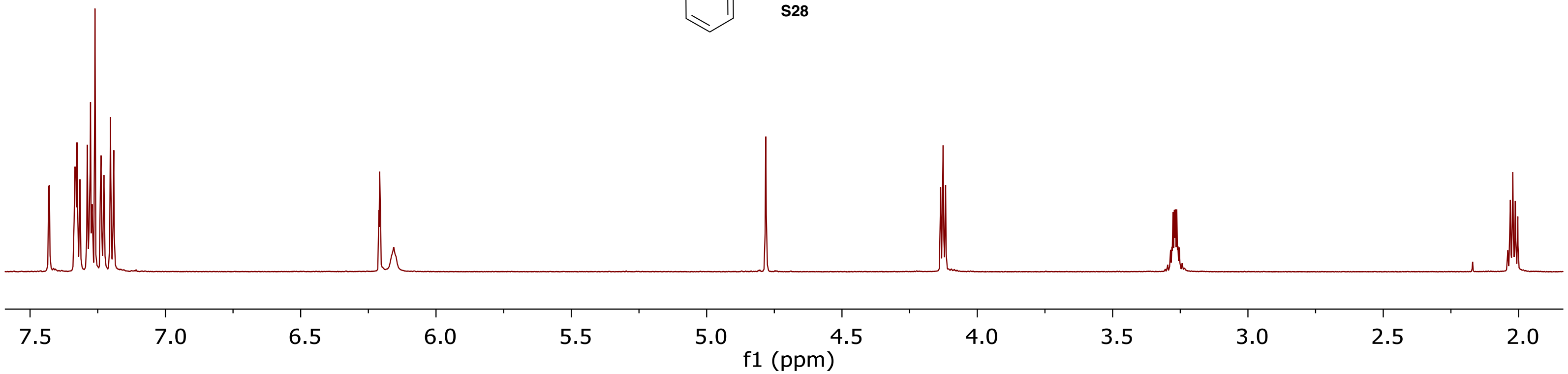

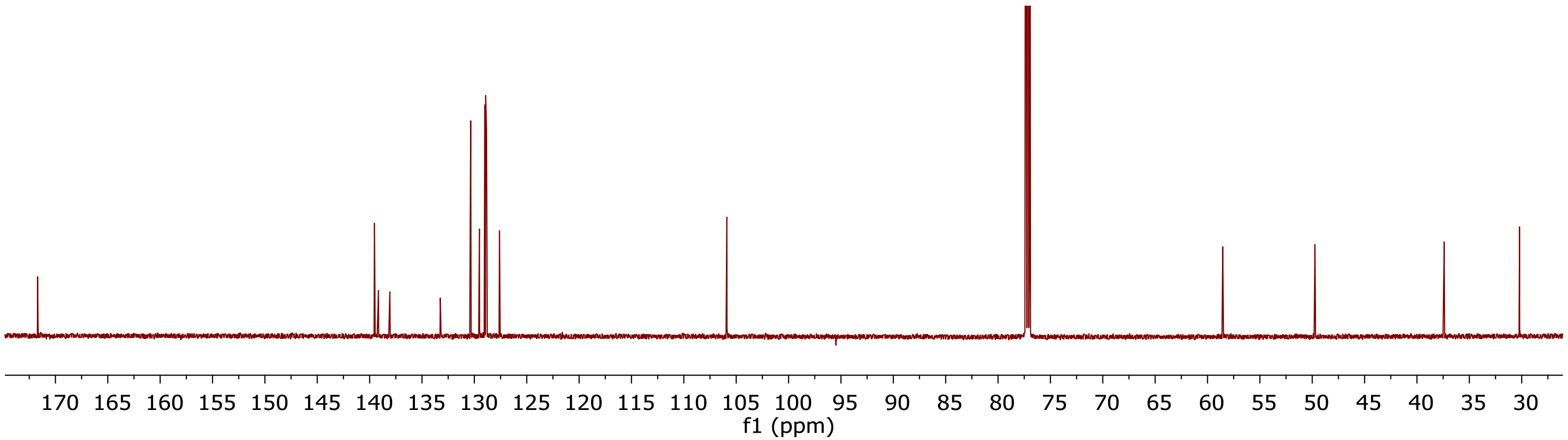

^{1}H and ^{13}C NMR Spectra for **S29**

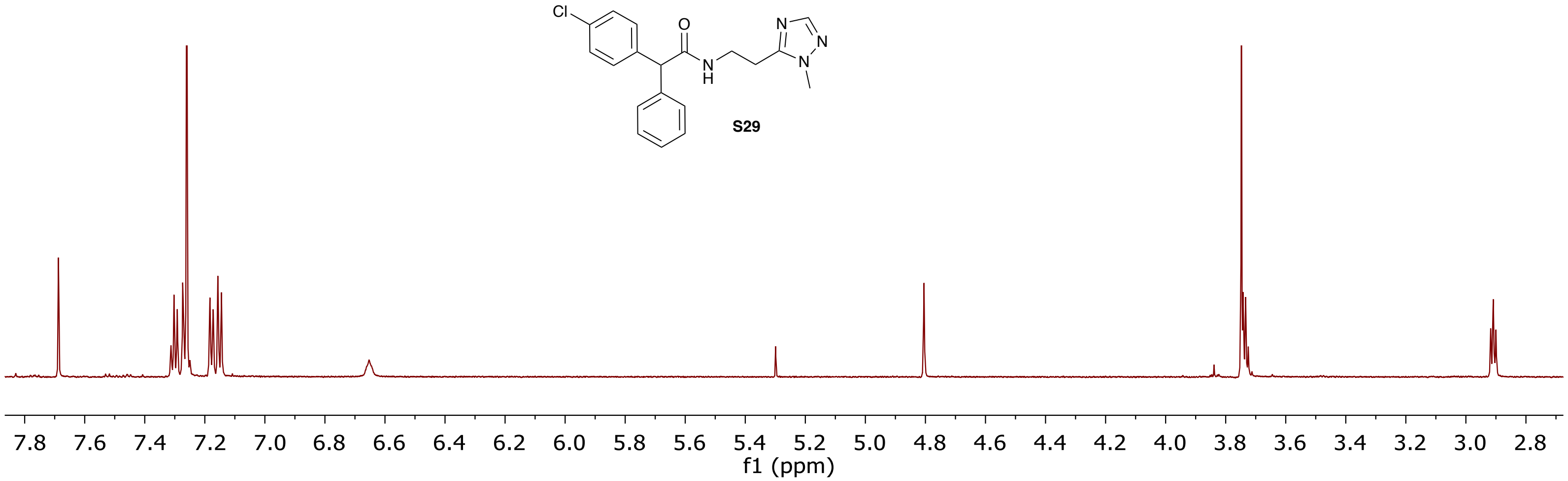

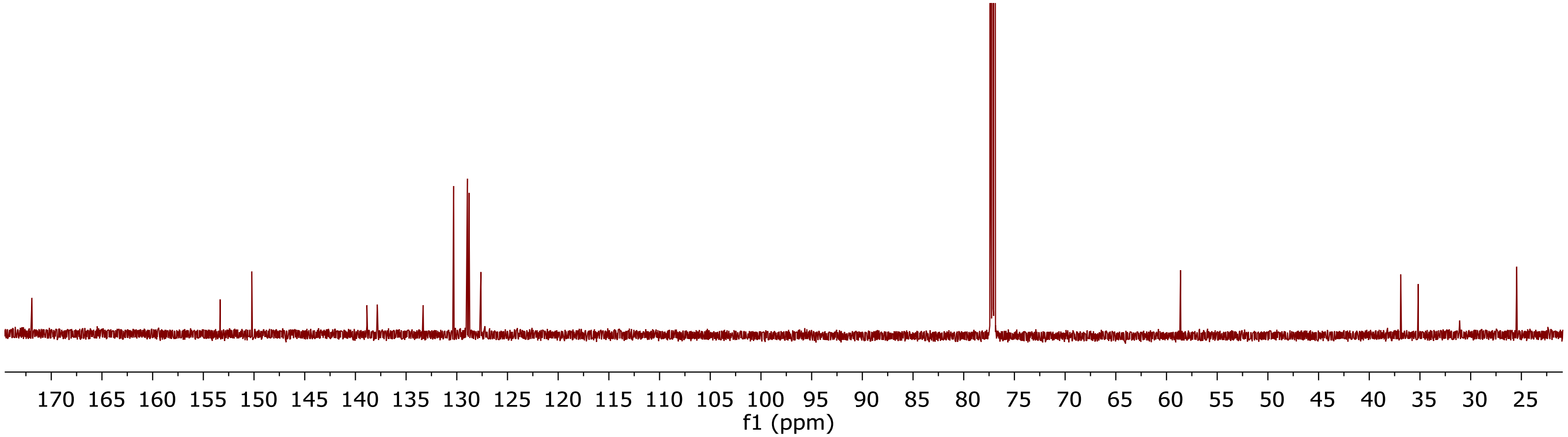

^{1}H and ^{13}C NMR Spectra for **S30**

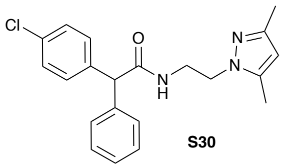

S30

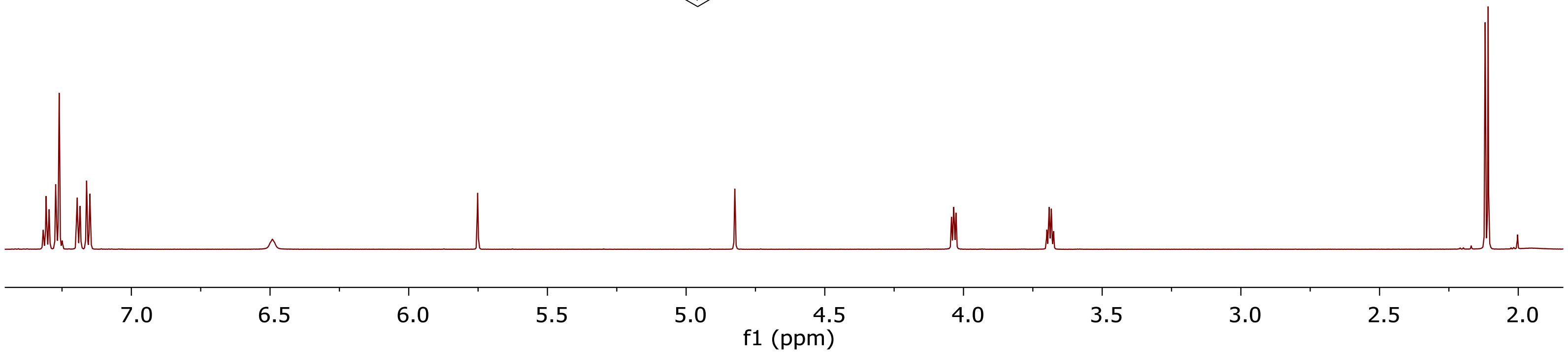

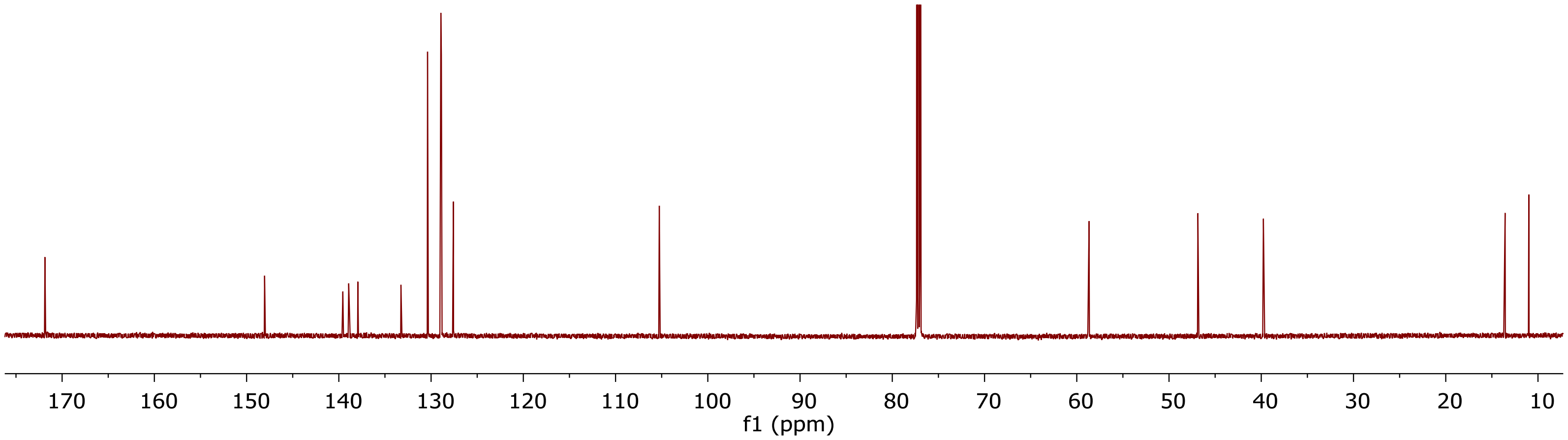

^{1}H and ^{13}C NMR Spectra for **S31**

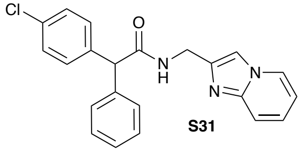

S31

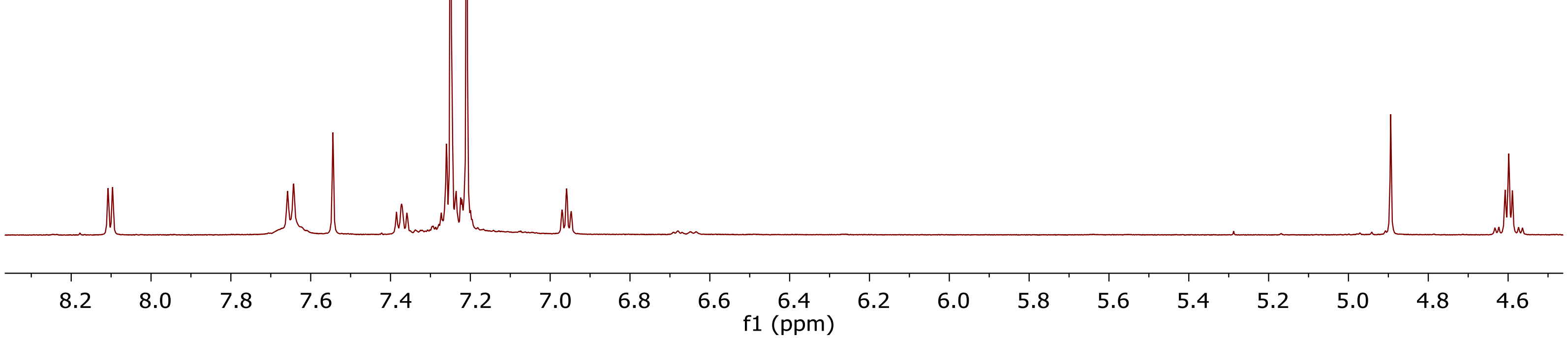

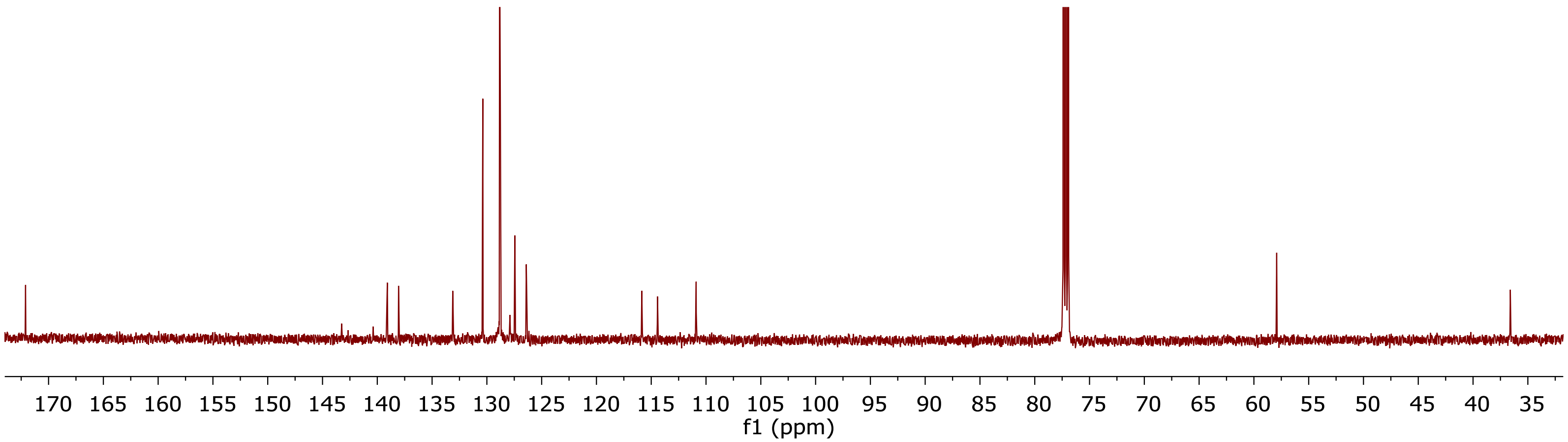

^{1}H and ^{13}C NMR Spectra for **S32**

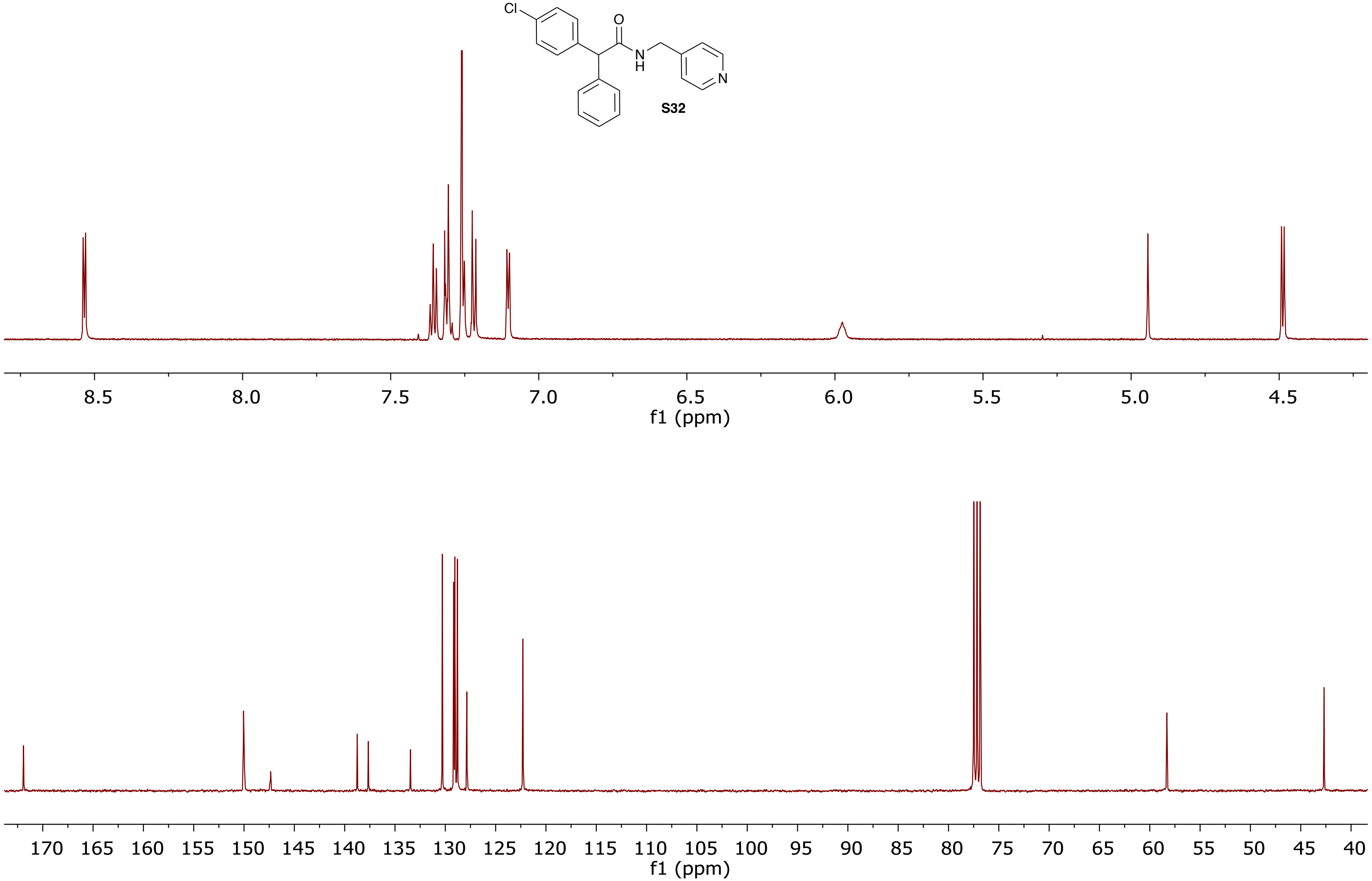

^{1}H and ^{13}C NMR Spectra for **S33**

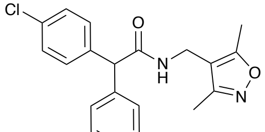

S33

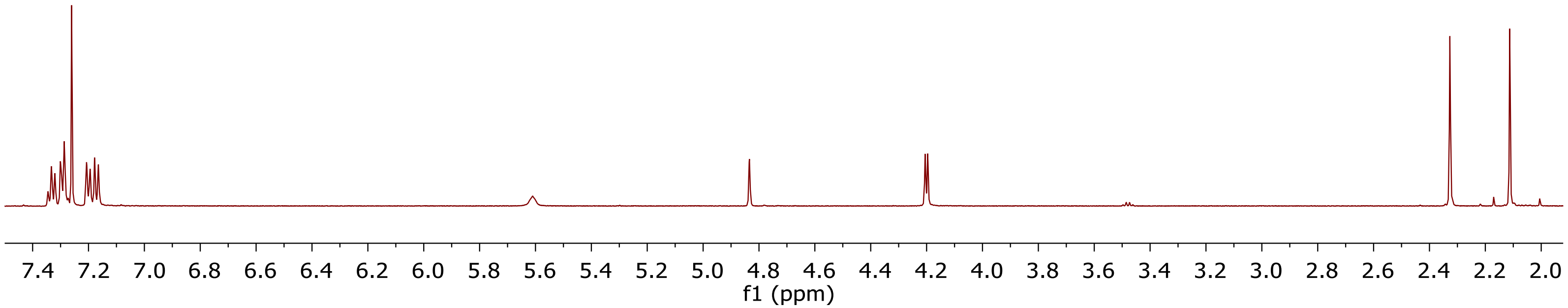

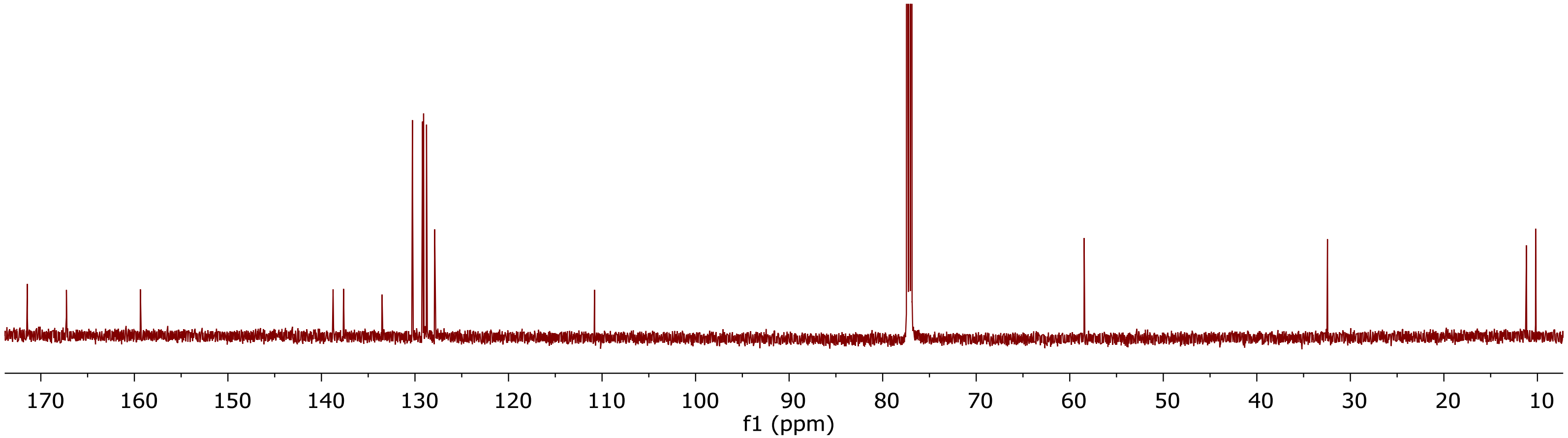

^{1}H and ^{13}C NMR Spectra for **S34**

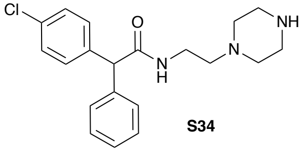

S34

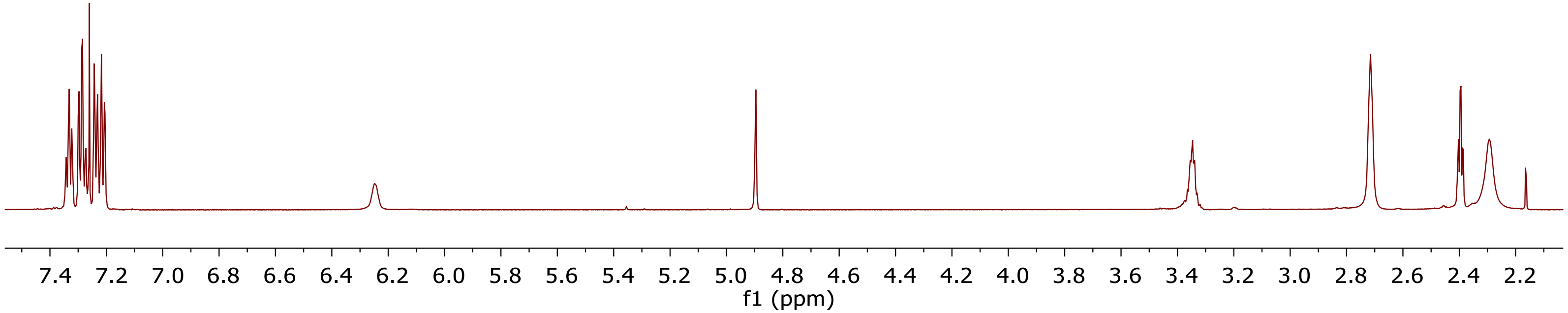

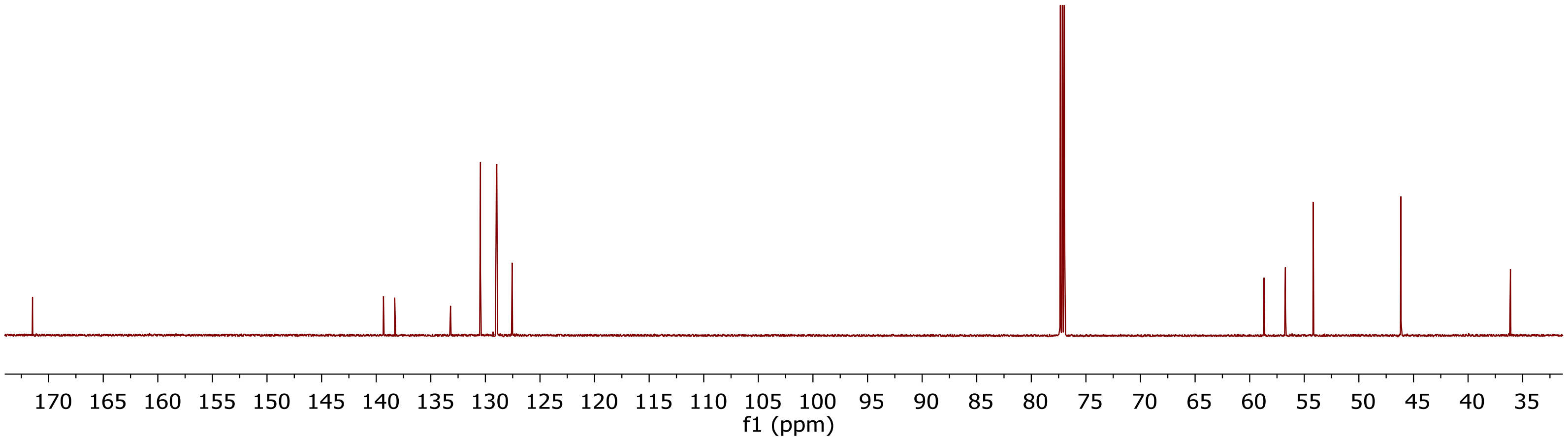

^{1}H and ^{13}C NMR Spectra for **S35**

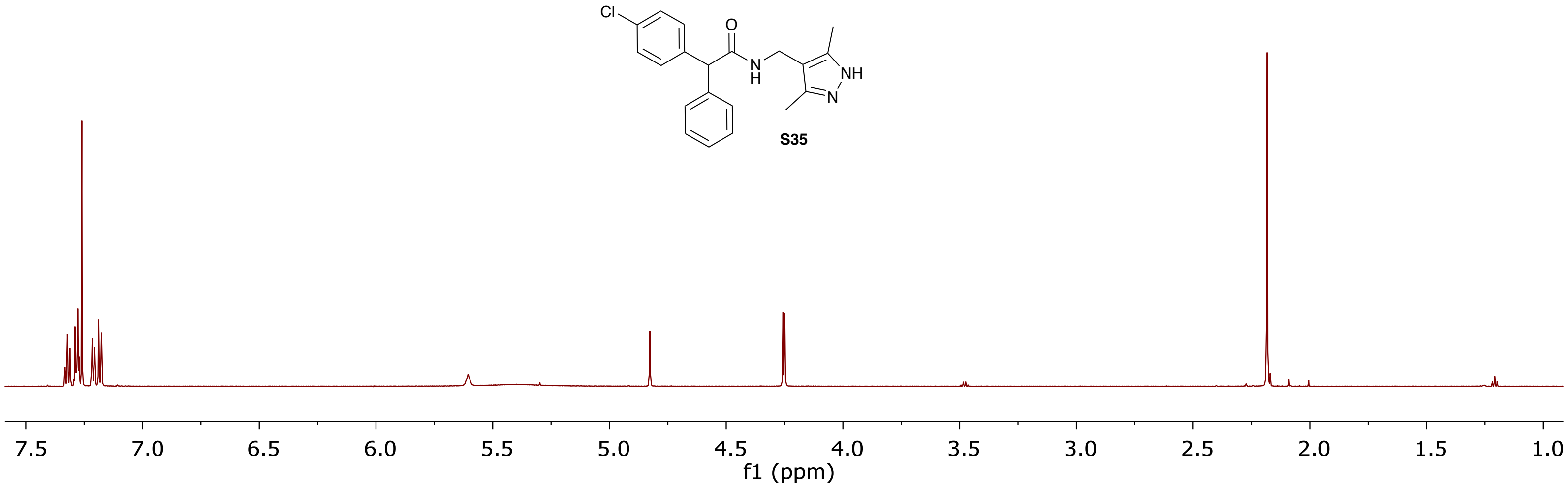

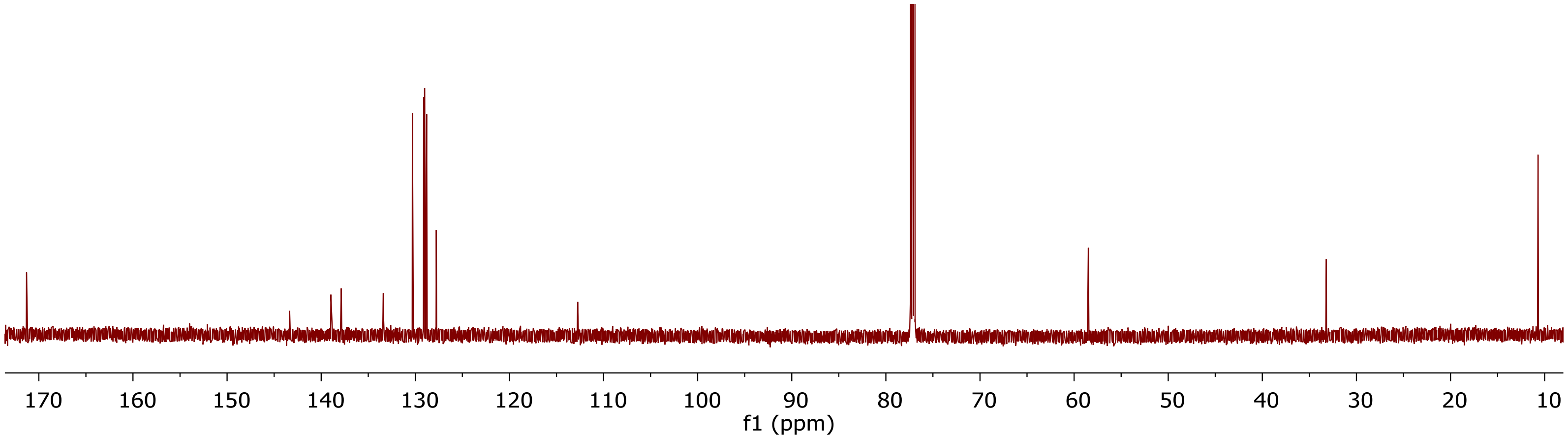

^{1}H and ^{13}C NMR Spectra for **S36**

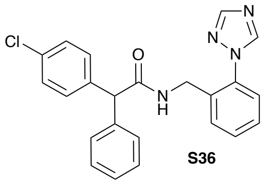

S36

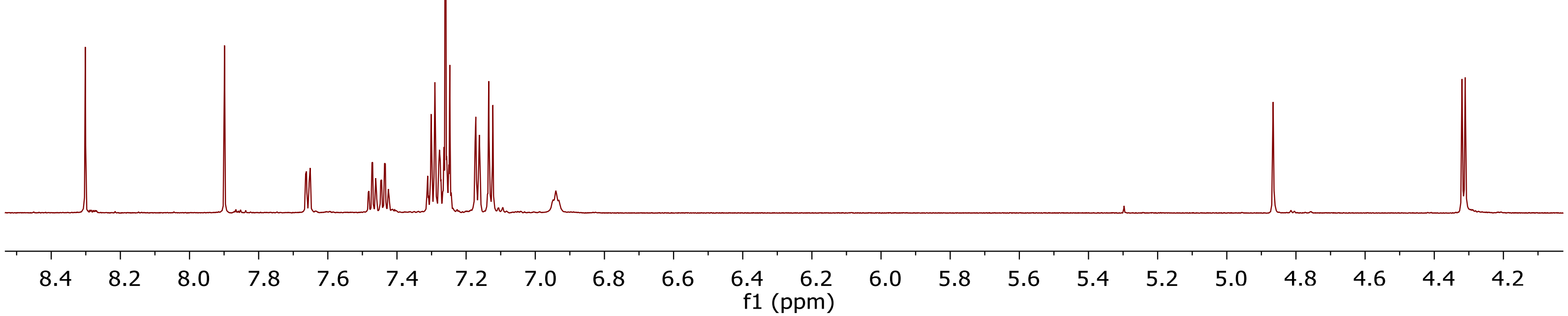

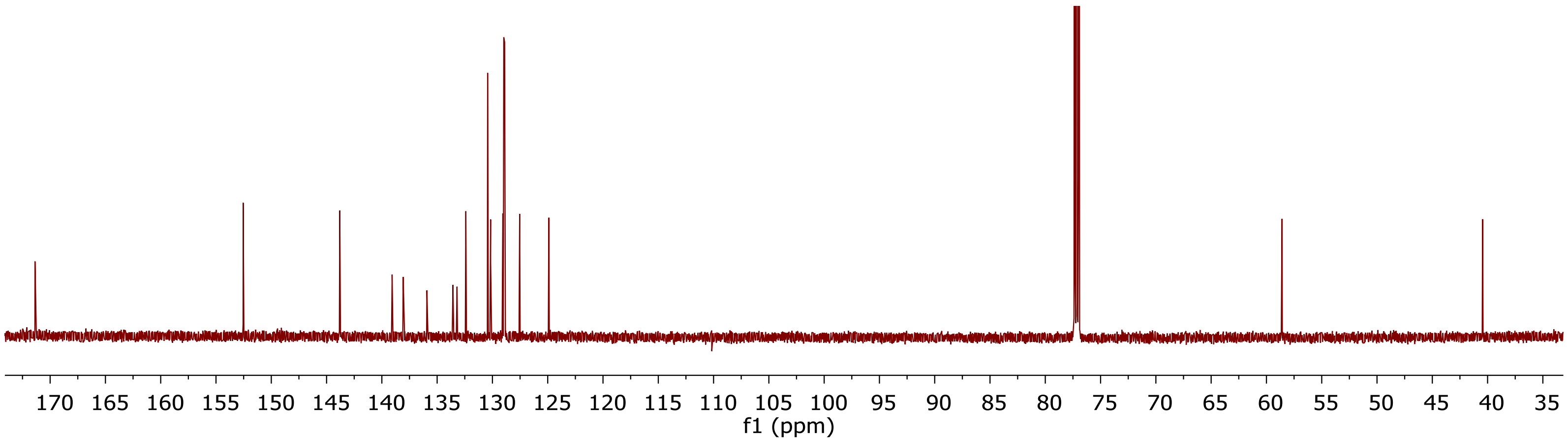

^{1}H and ^{13}C NMR Spectra for **S37**

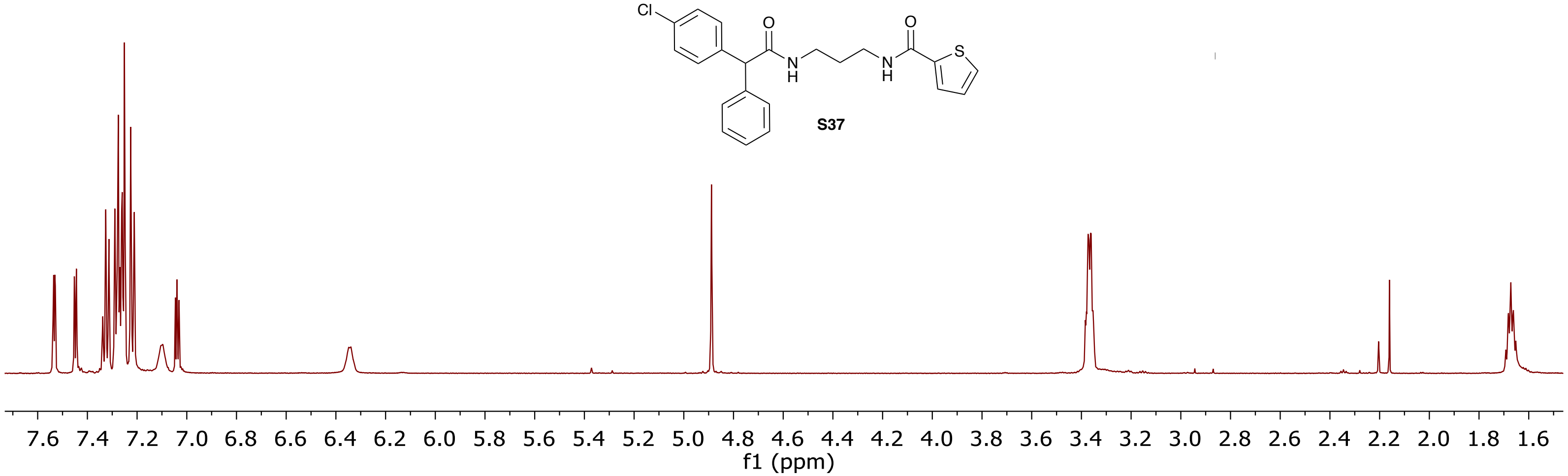

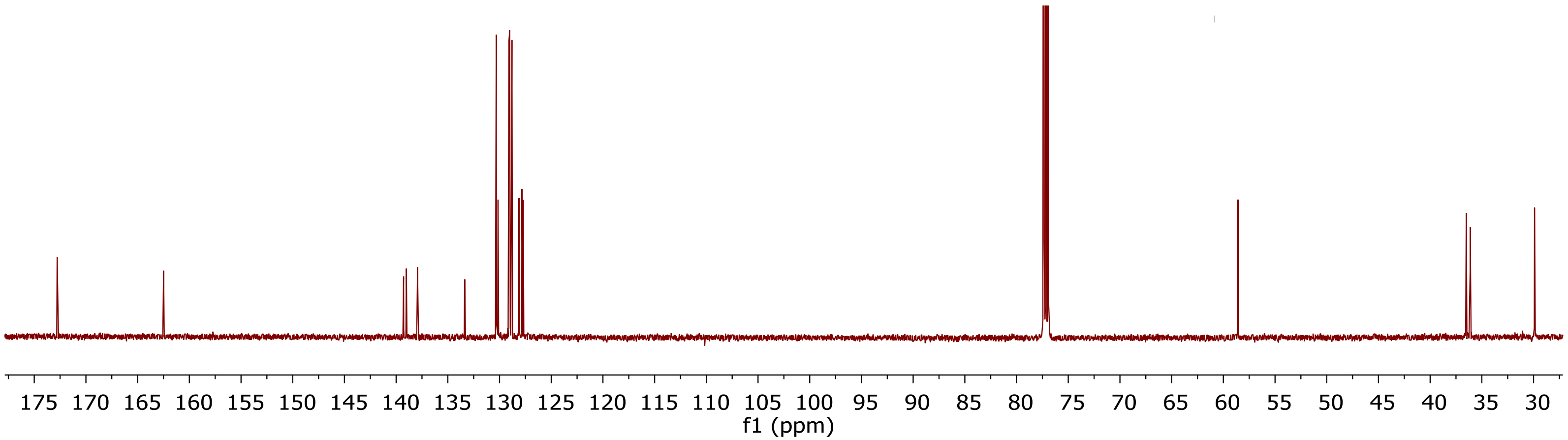

^{1}H and ^{13}C NMR Spectra for **S38**

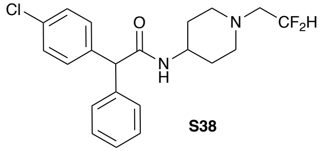

S38

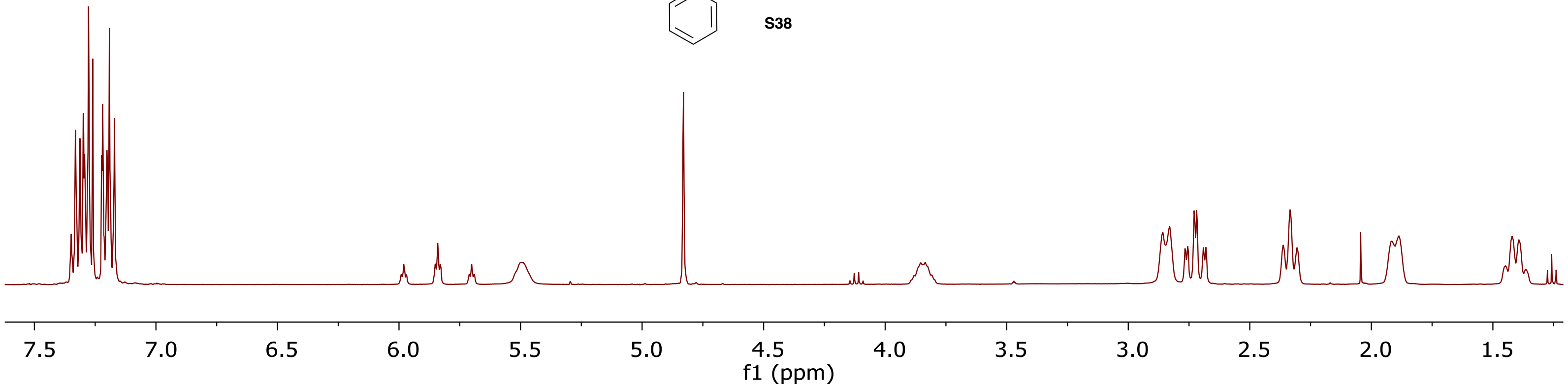

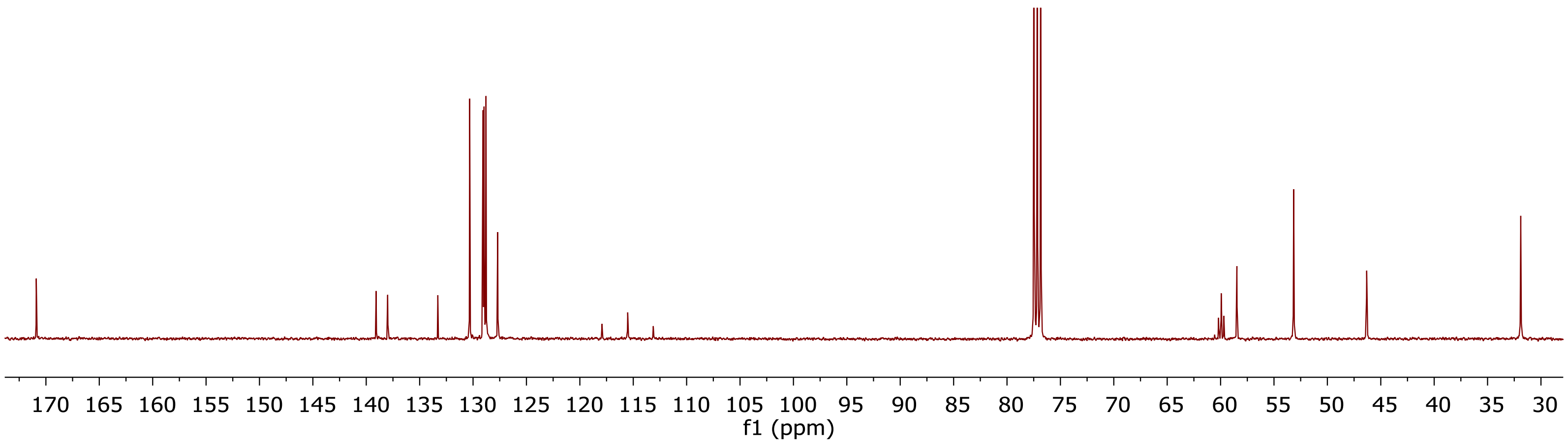

^{1}H and ^{13}C NMR Spectra for **S39**

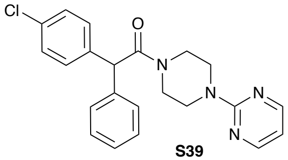

S39

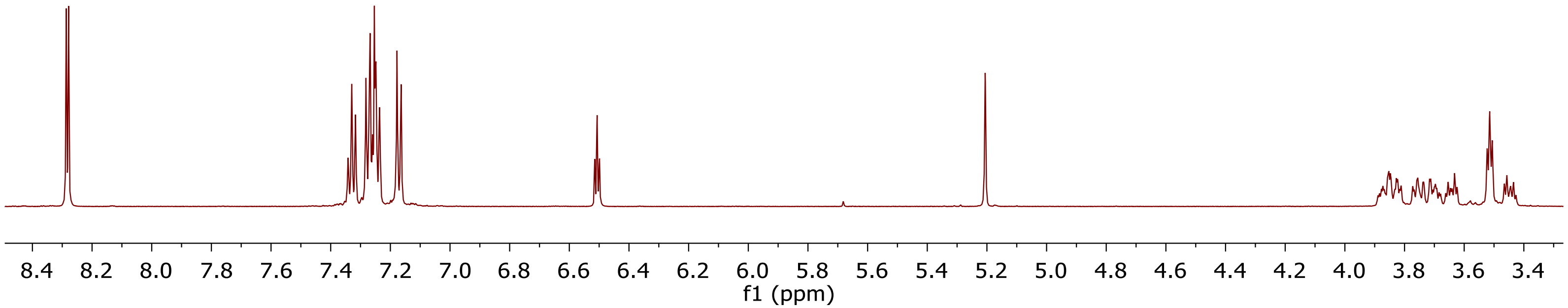

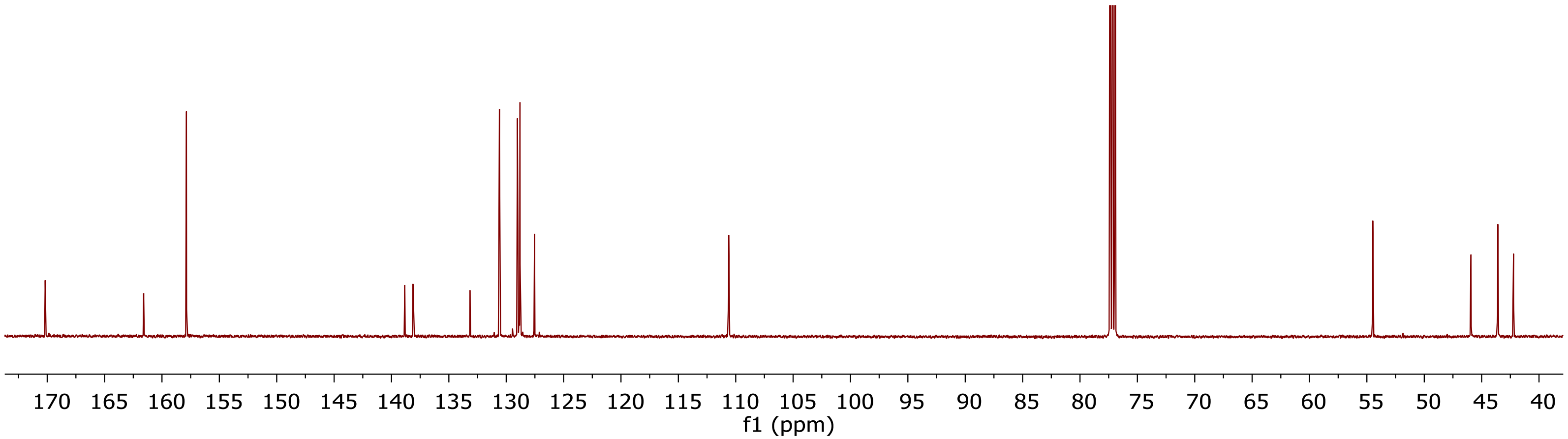

^{1}H and ^{13}C NMR Spectra for **S40**

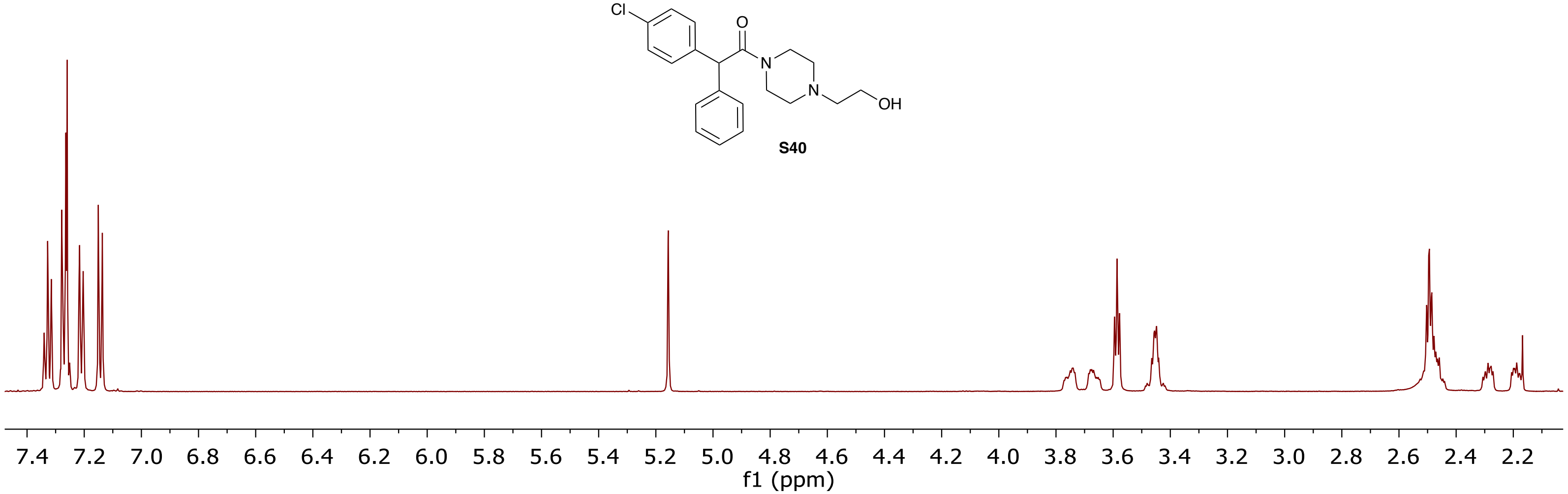

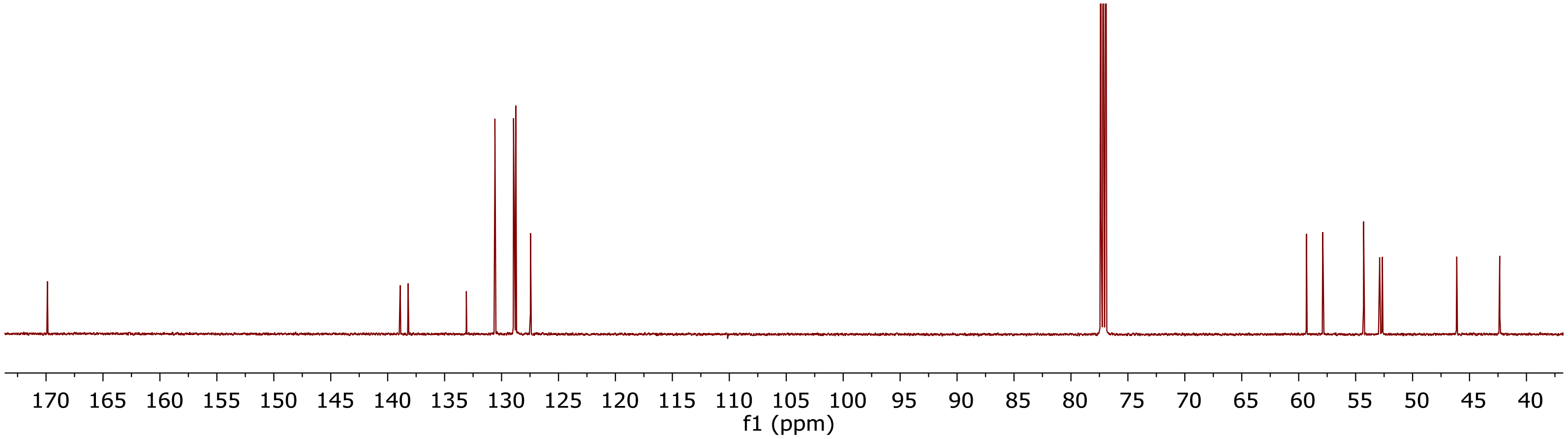

^{1}H and ^{13}C NMR Spectra for **S41**

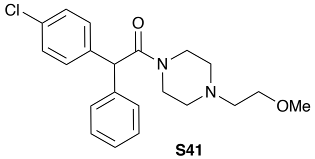

S41

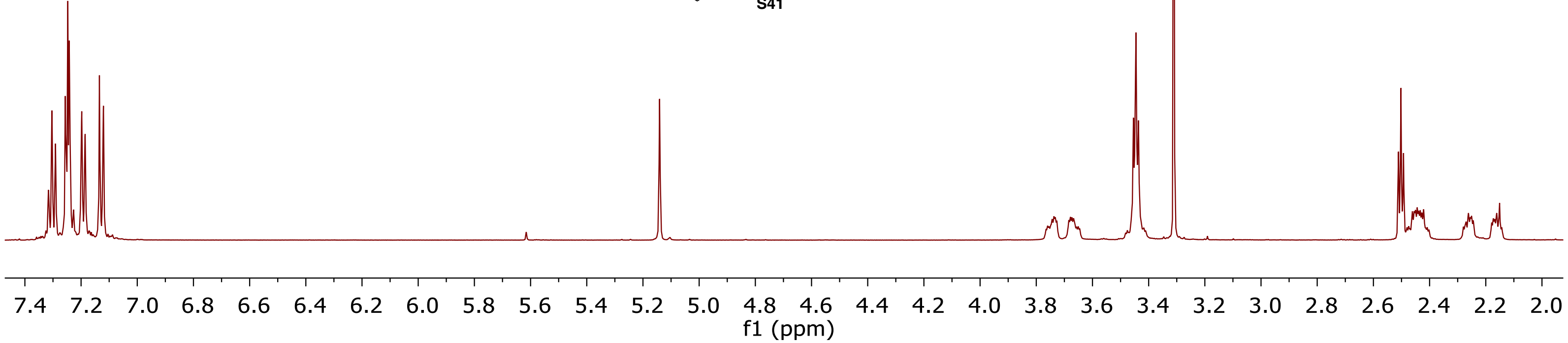

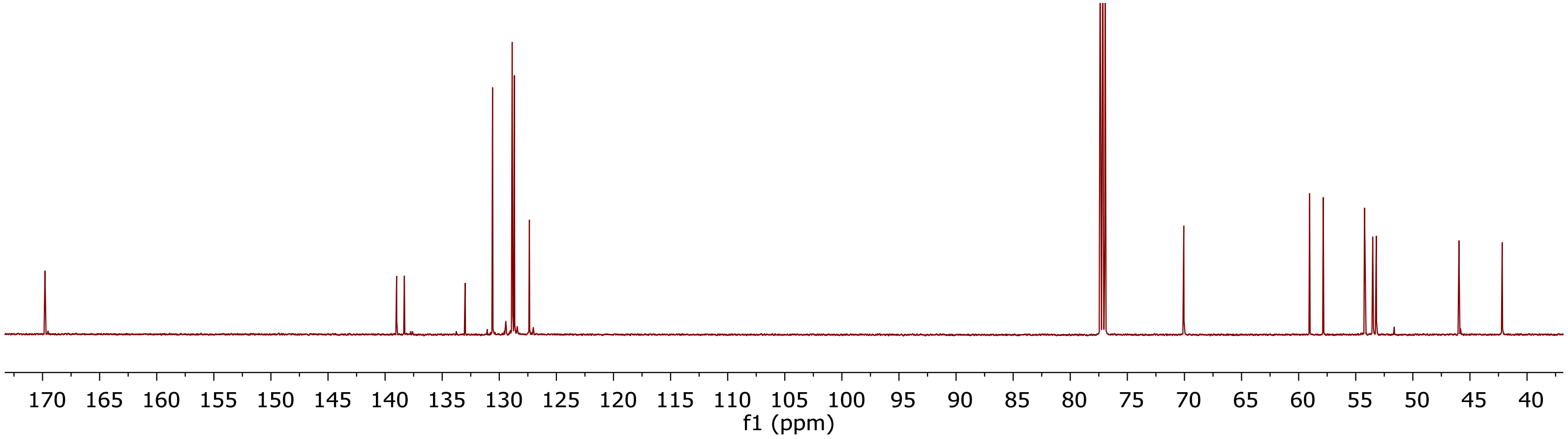

^{1}H and ^{13}C NMR Spectra for **S42**

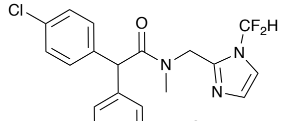

S42

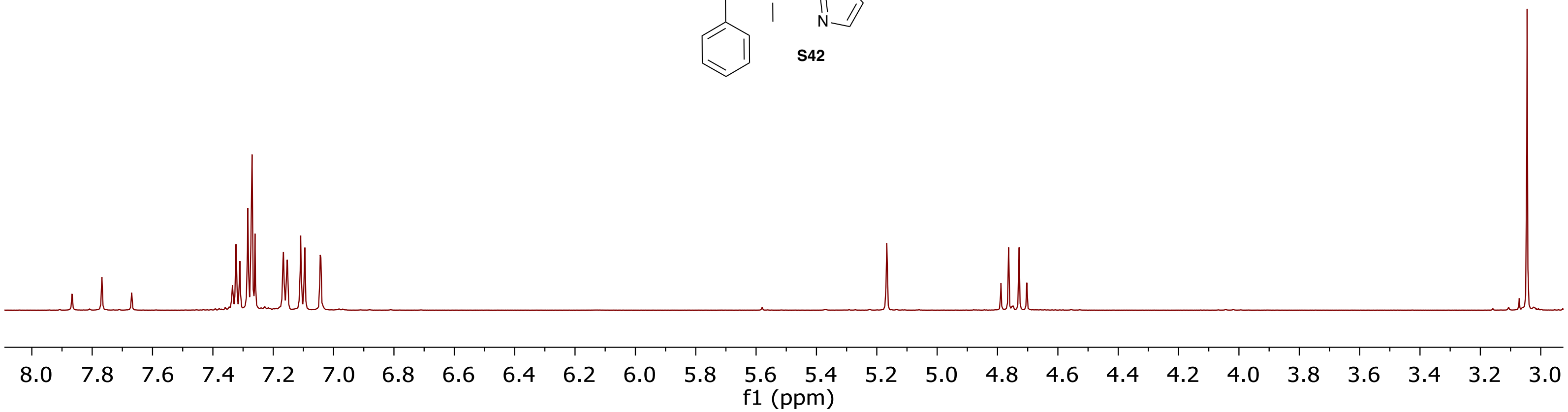

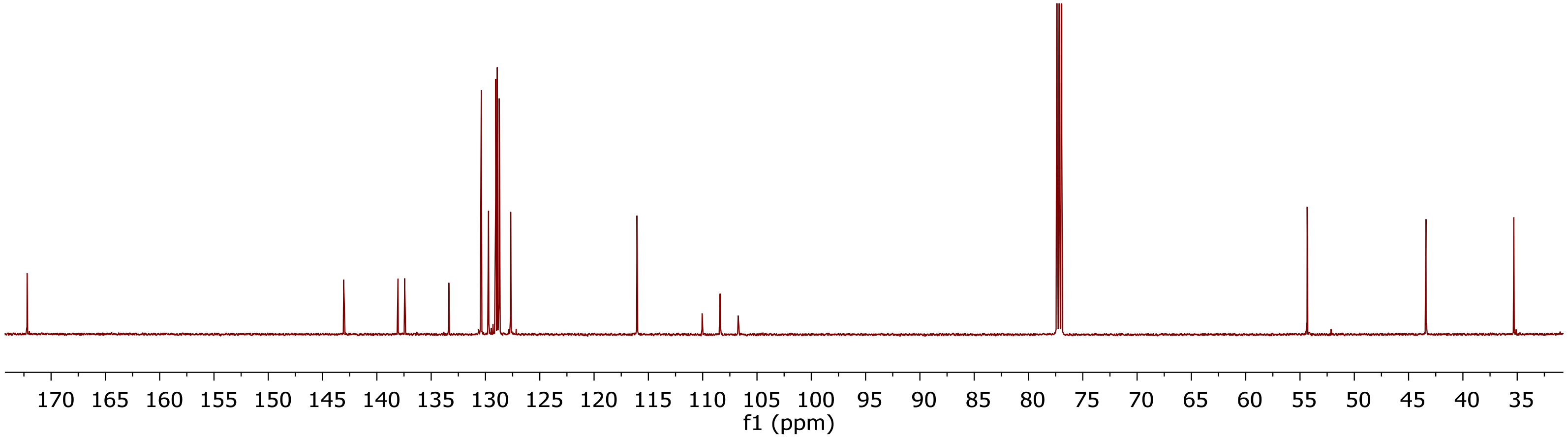

^{1}H and ^{13}C NMR Spectra for **S43**

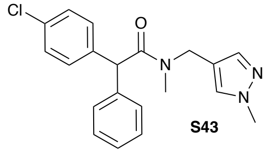

S43

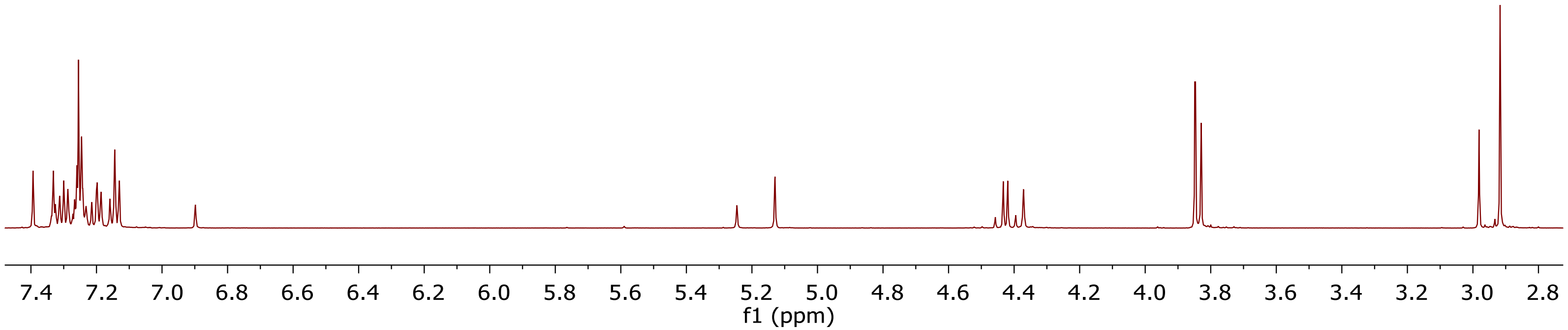

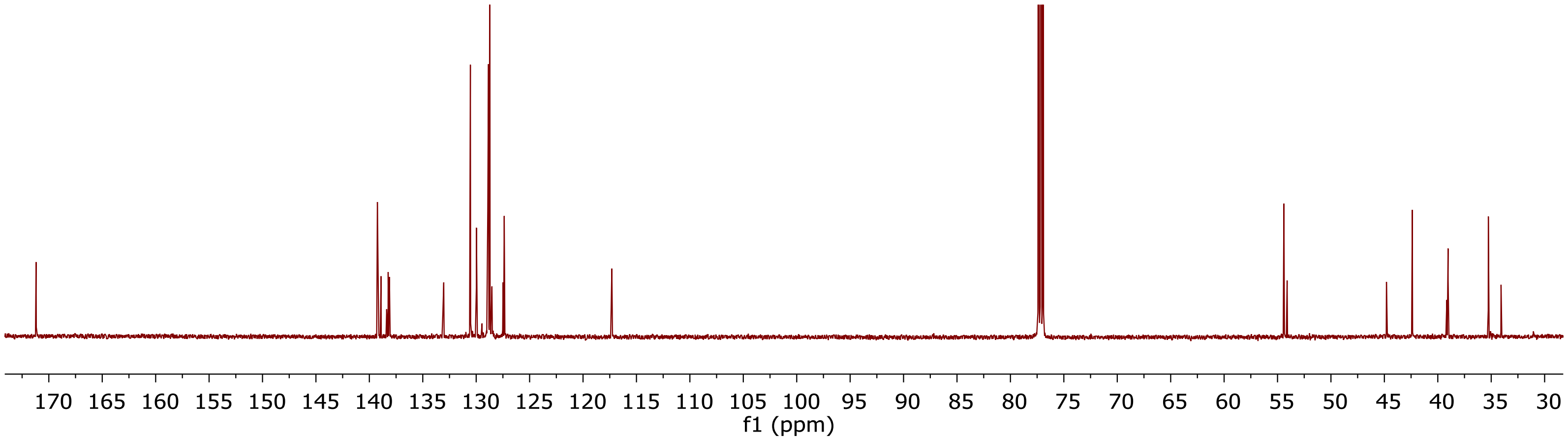

^{1}H and ^{13}C NMR Spectra for **S44**

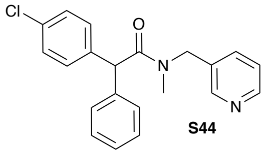

S44

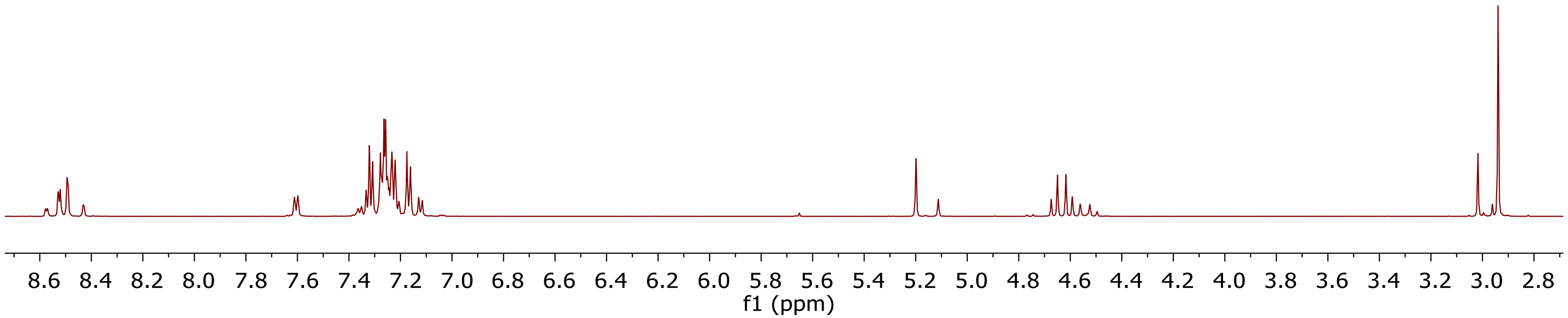

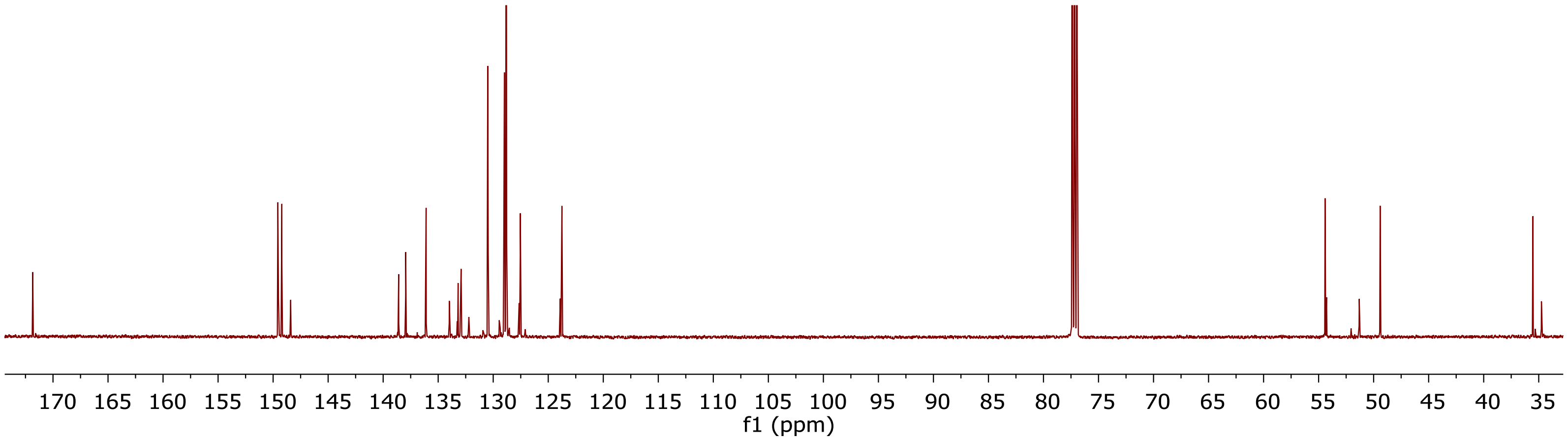

^1H and ^{13}C NMR Spectra for **S45**

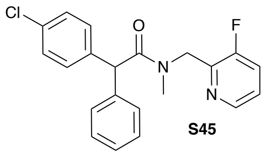

S45

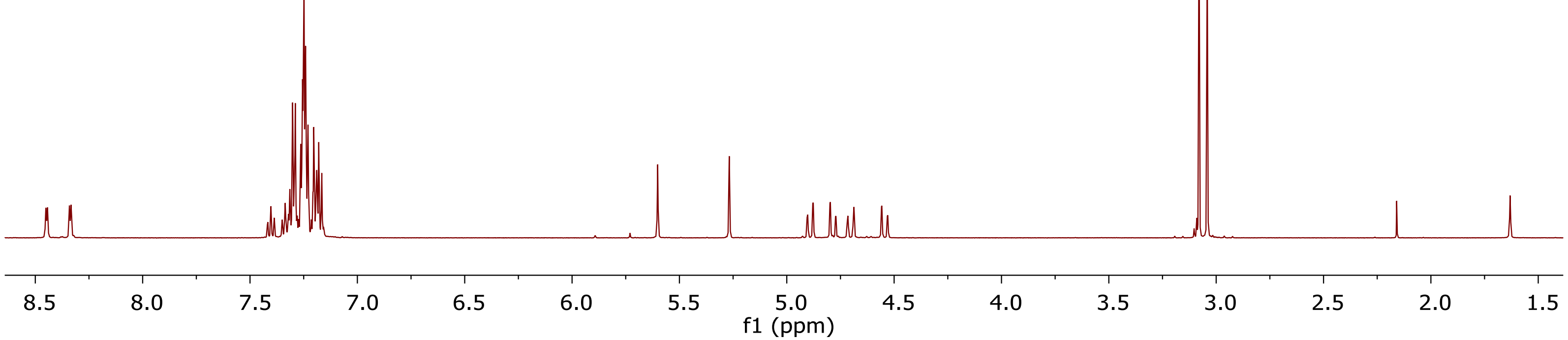

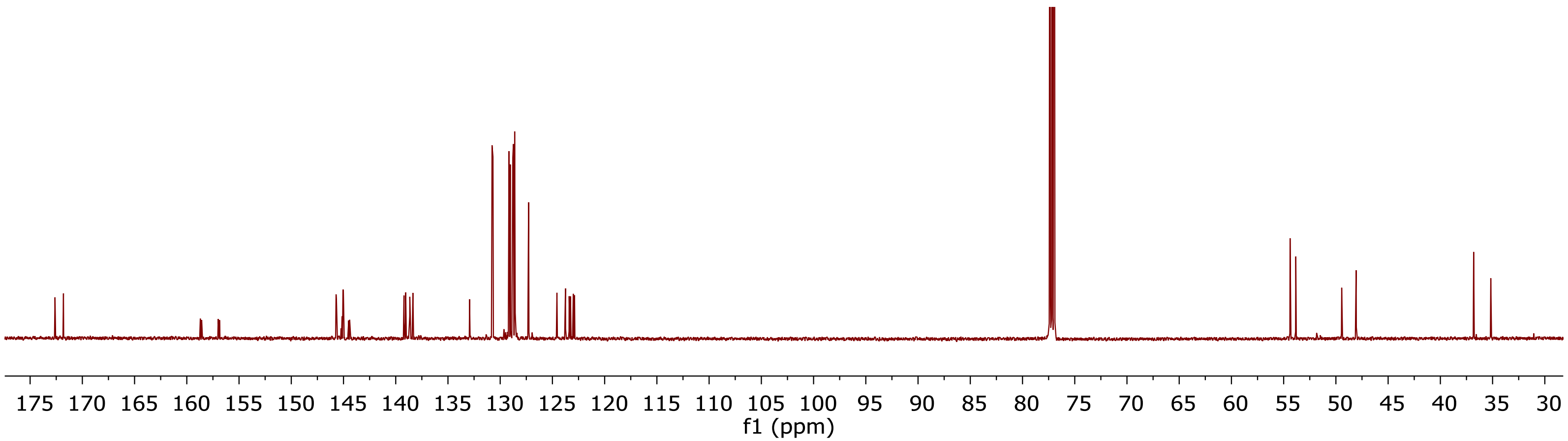

^{1}H and ^{13}C NMR Spectra for **S46**

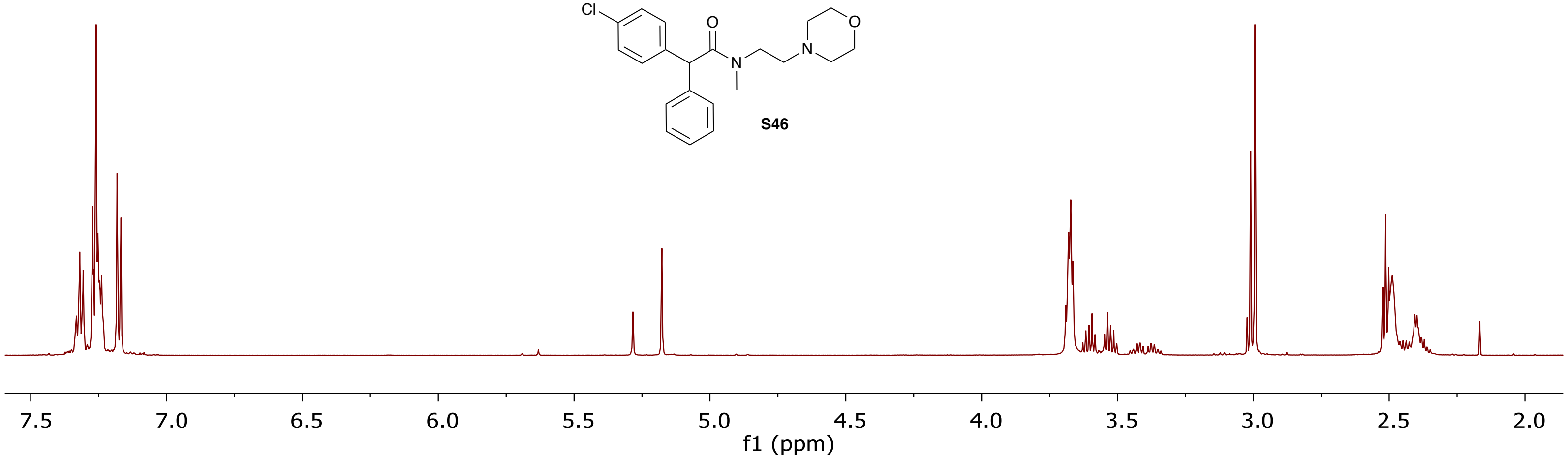

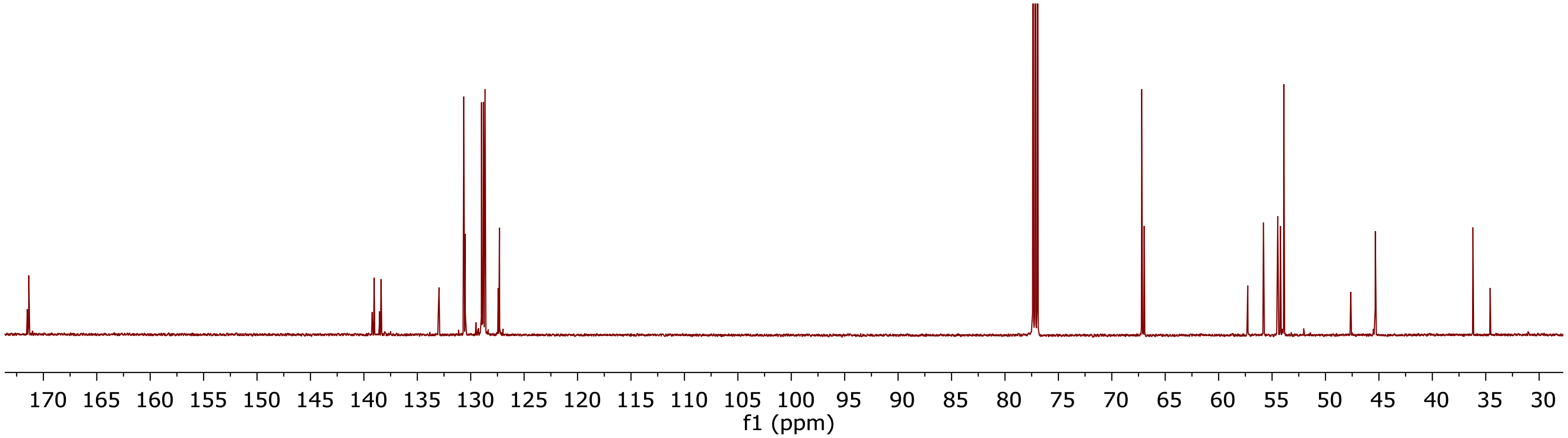

^{1}H and ^{13}C NMR Spectra for **S47**

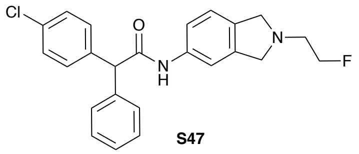

S47

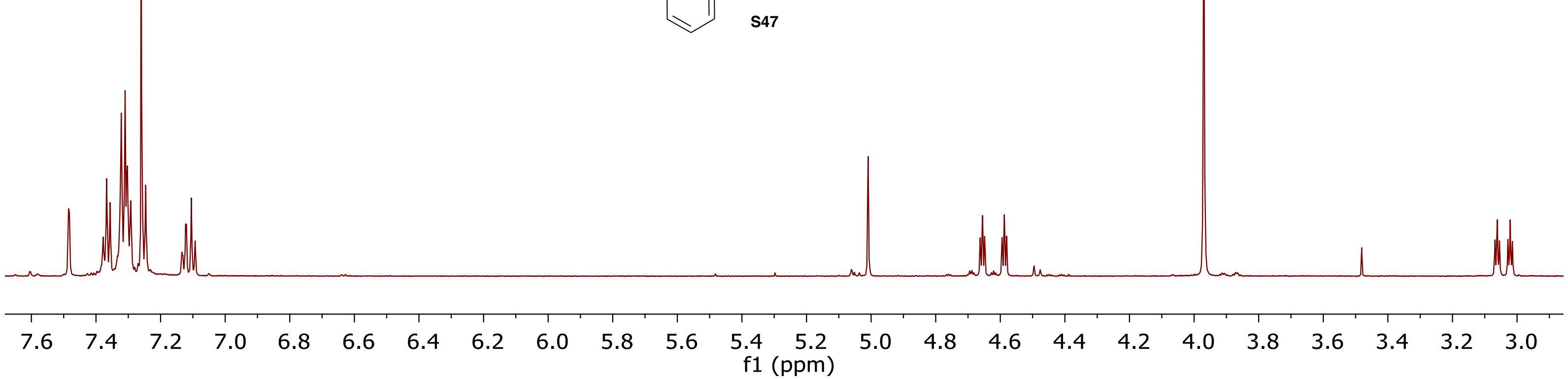

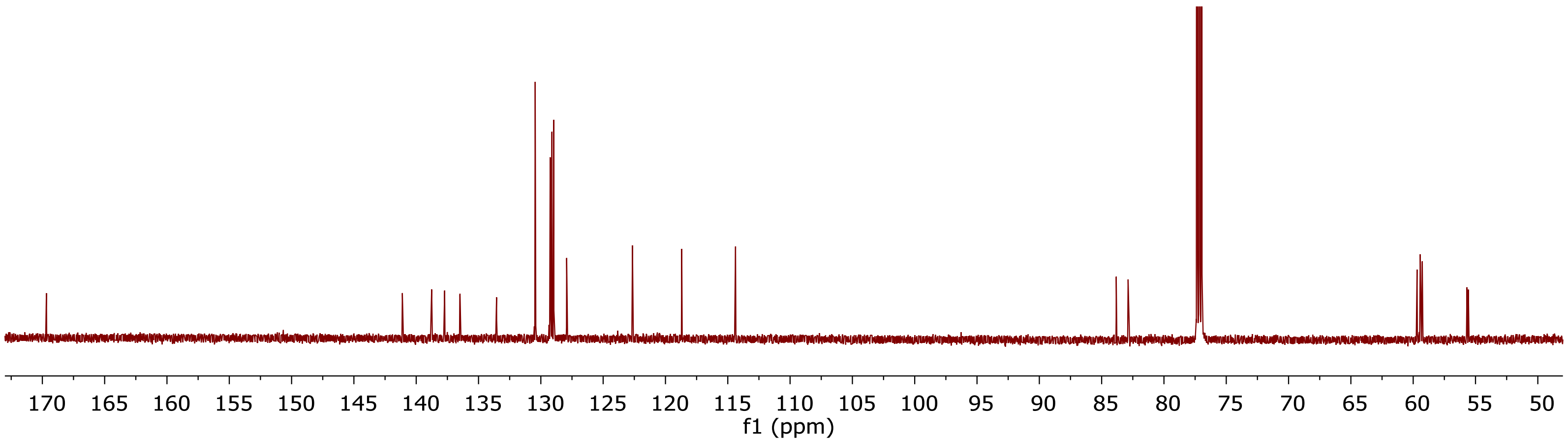

^{1}H and ^{13}C NMR Spectra for **S48**

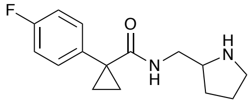

S48

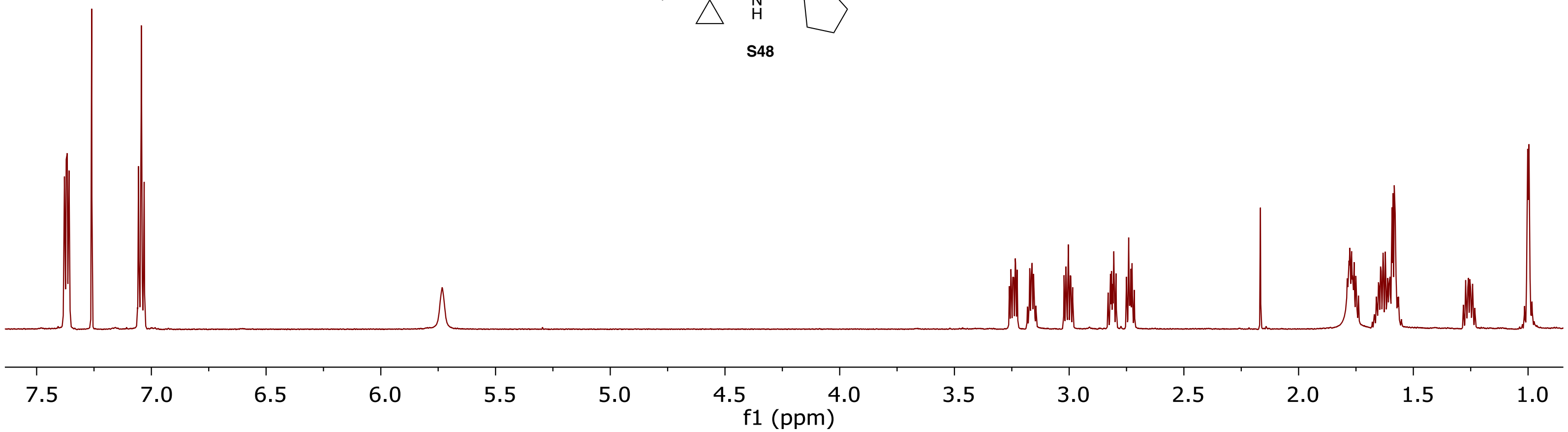

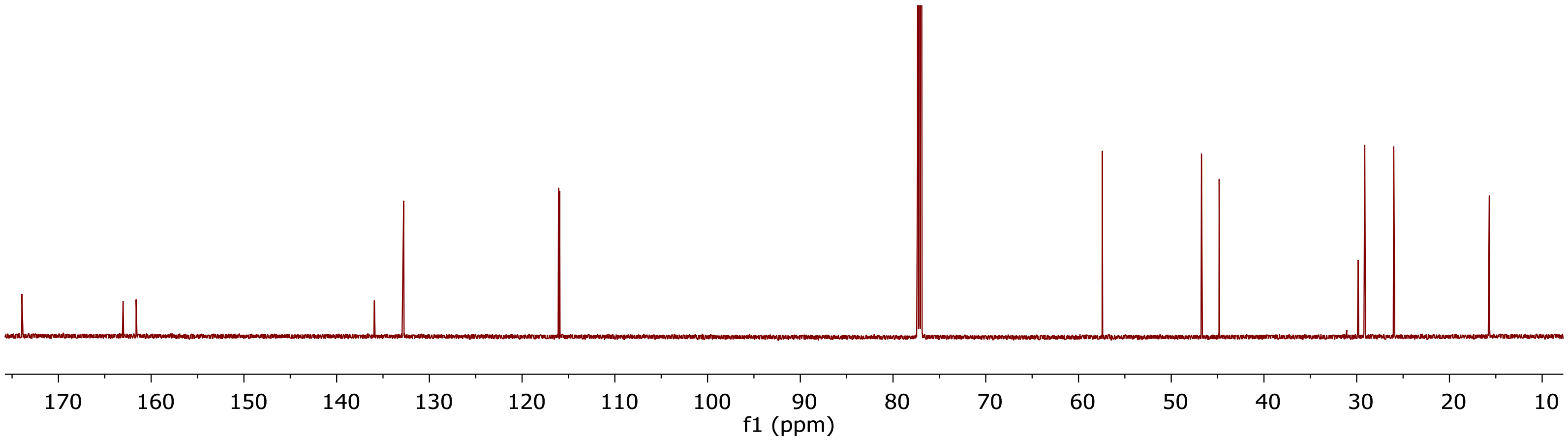

^{1}H and ^{13}C NMR Spectra for **S49**

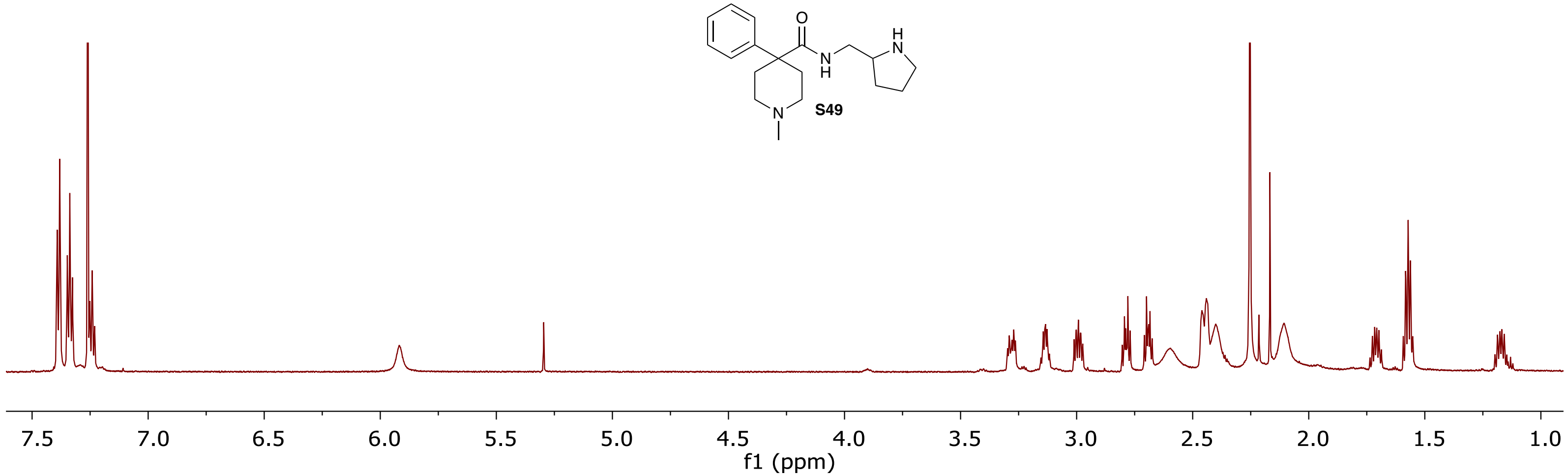

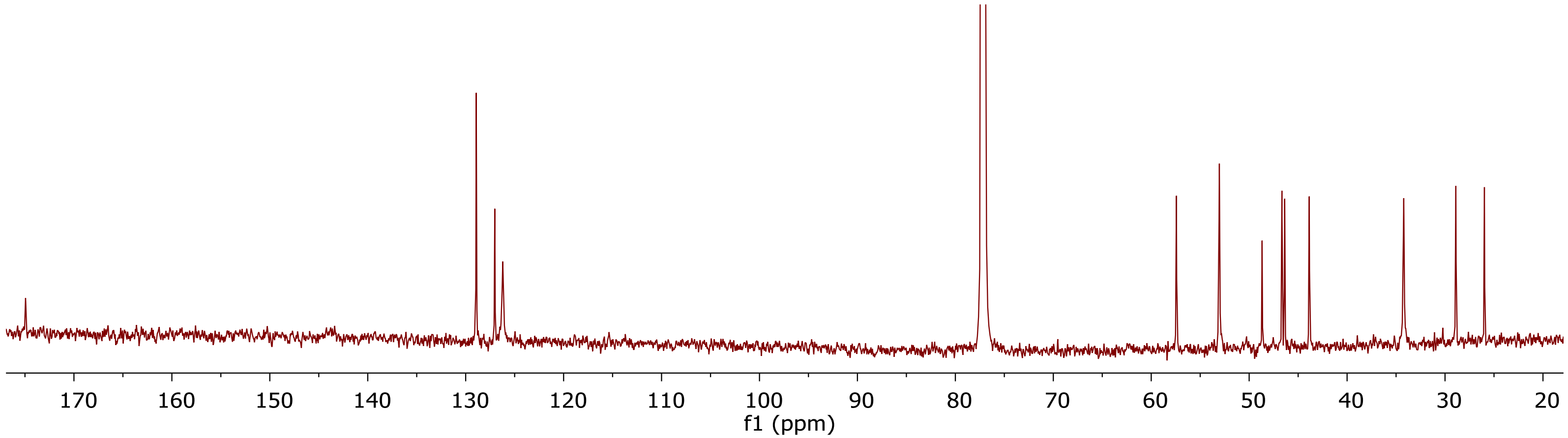

^{1}H and ^{13}C NMR Spectra for **S50**

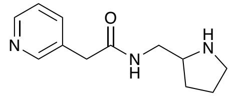

S50

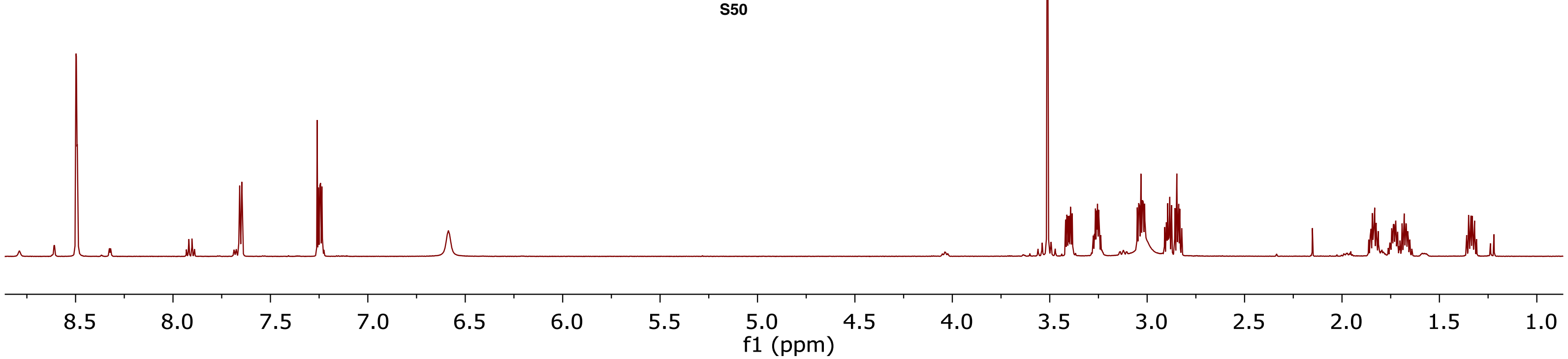

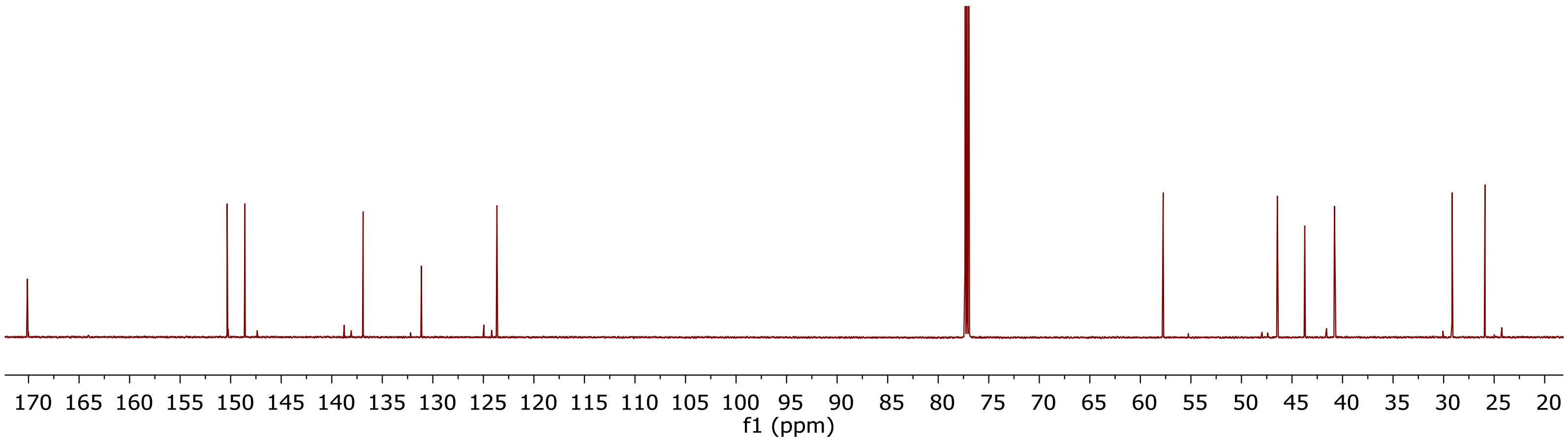

^{1}H and ^{13}C NMR Spectra for **S51**

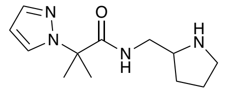

S51

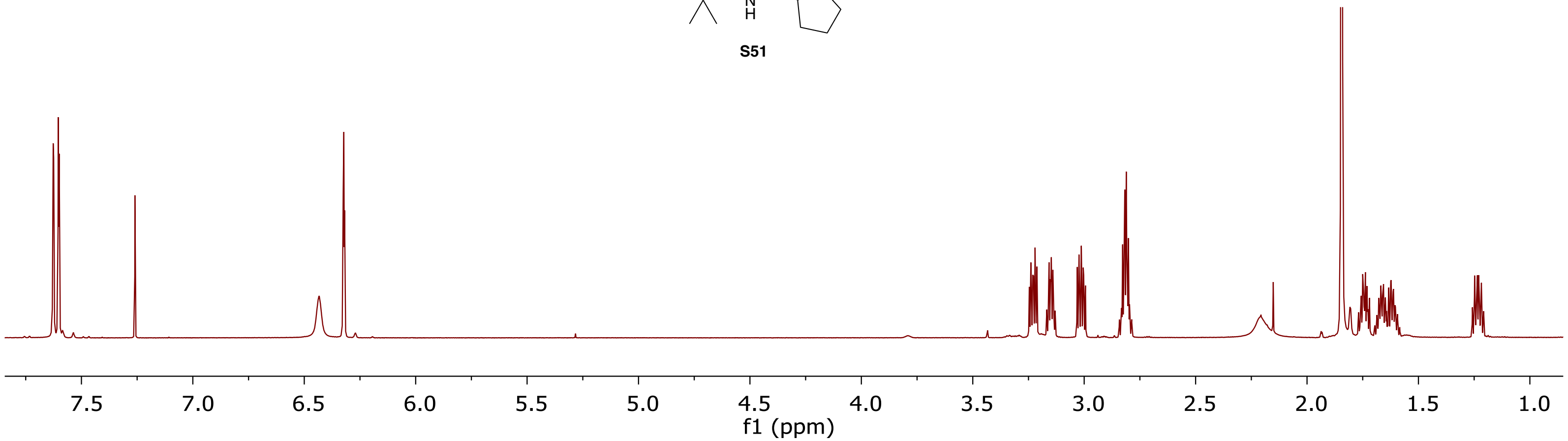

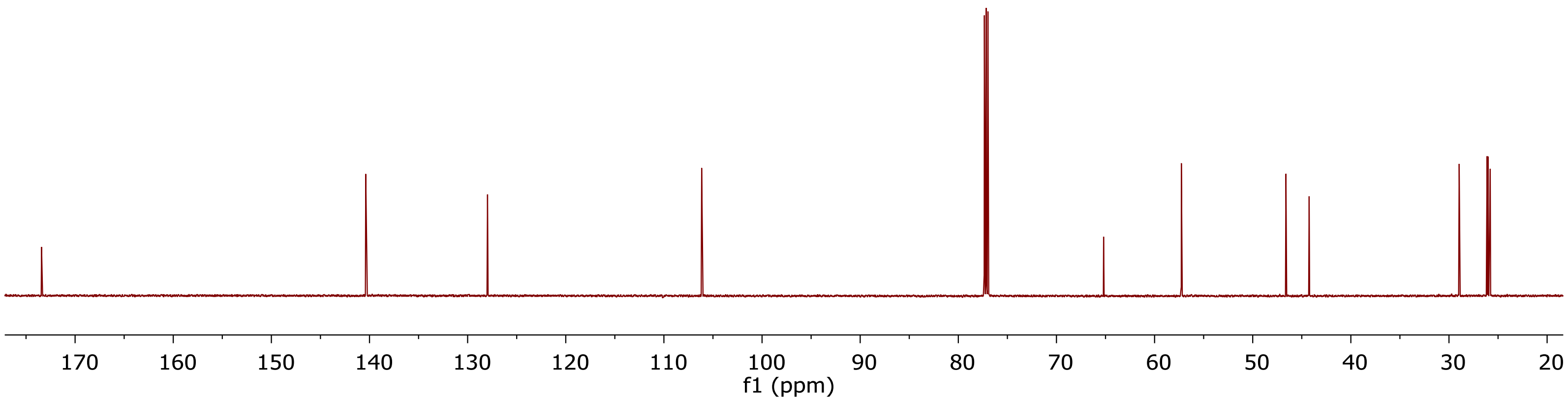

^{1}H and ^{13}C NMR Spectra for **S52**

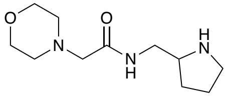

S52

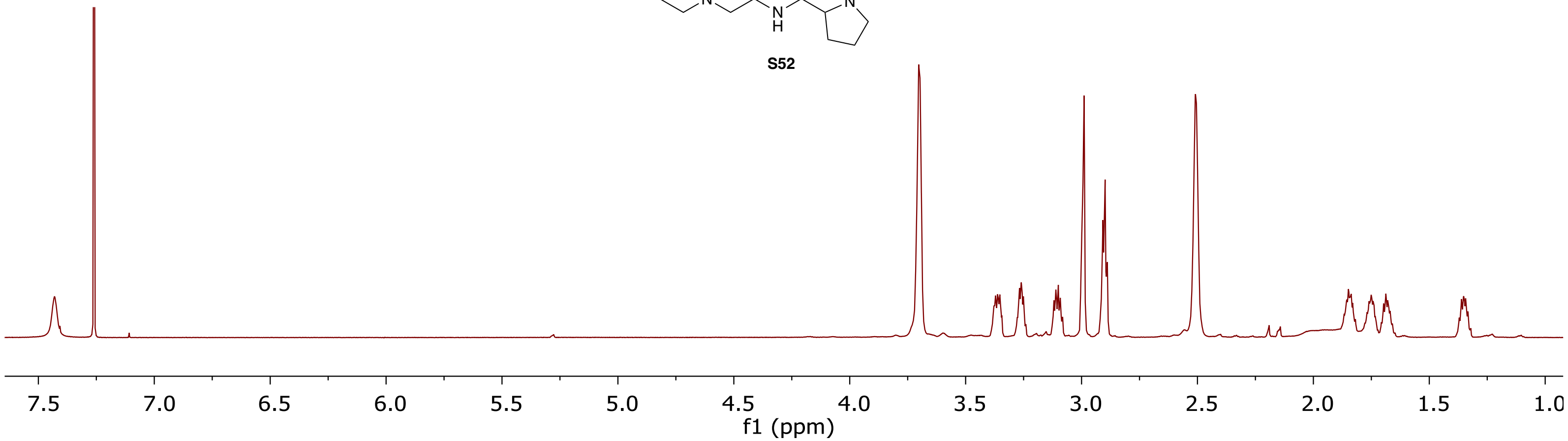

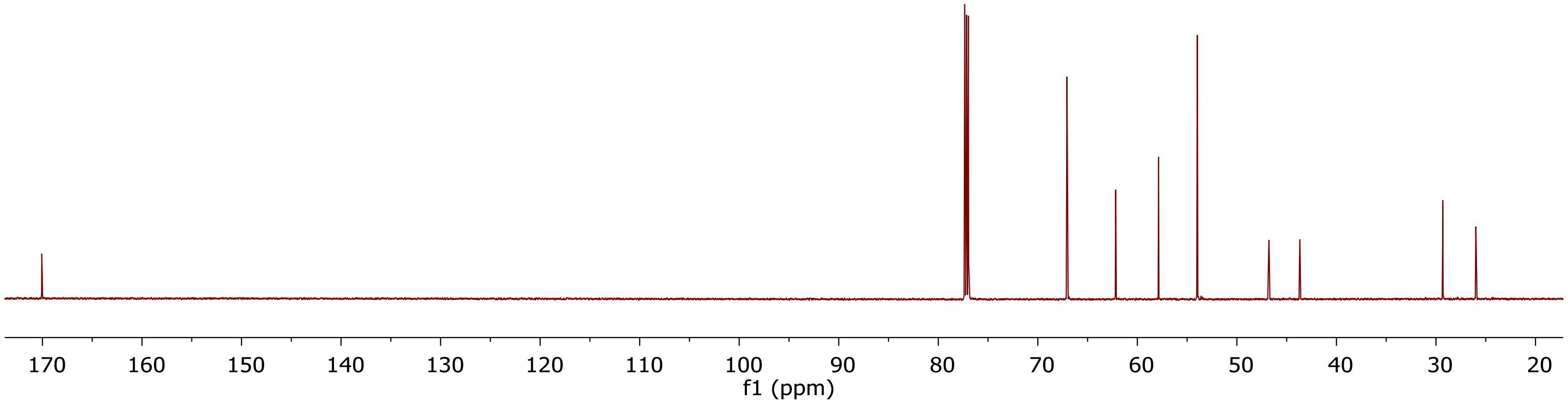

^{1}H and ^{13}C NMR Spectra for **S53**

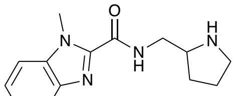

S53

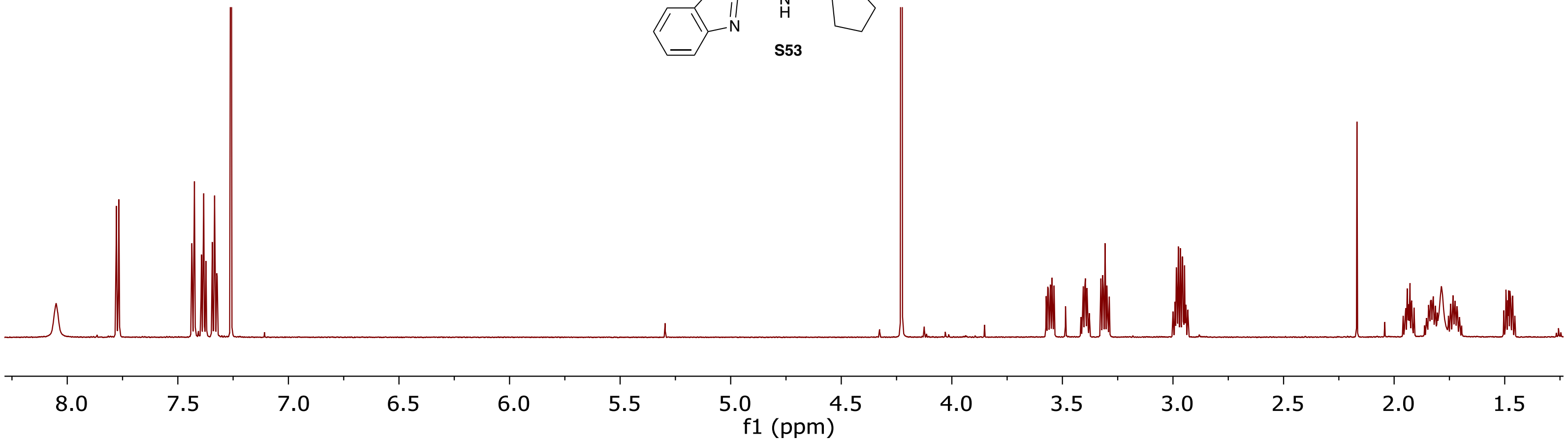

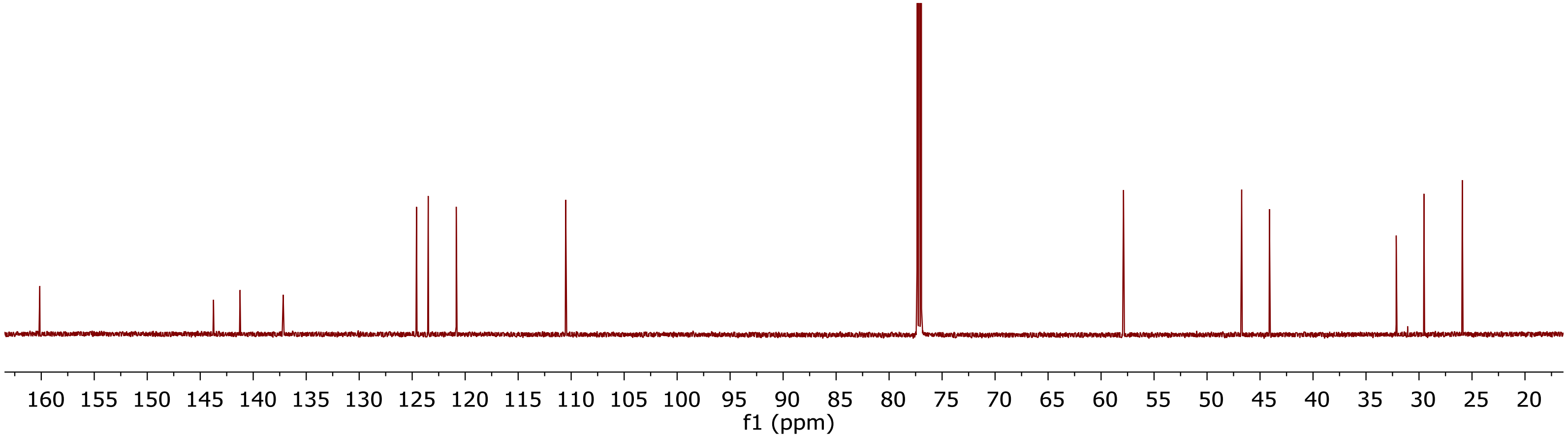

^{1}H and ^{13}C NMR Spectra for **S54**

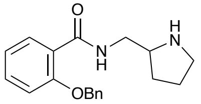

S54

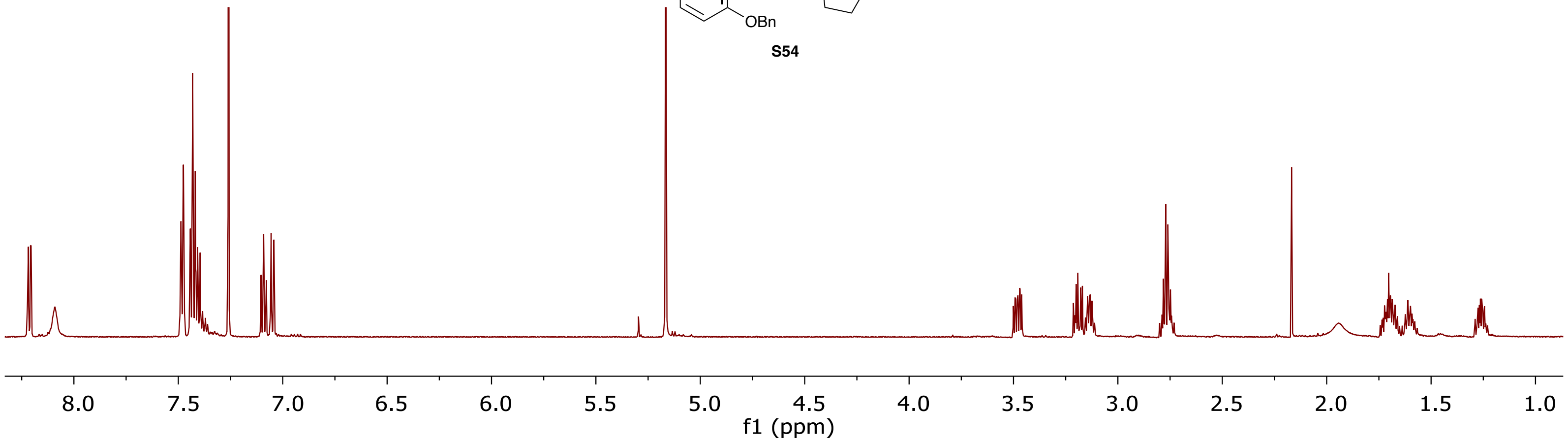

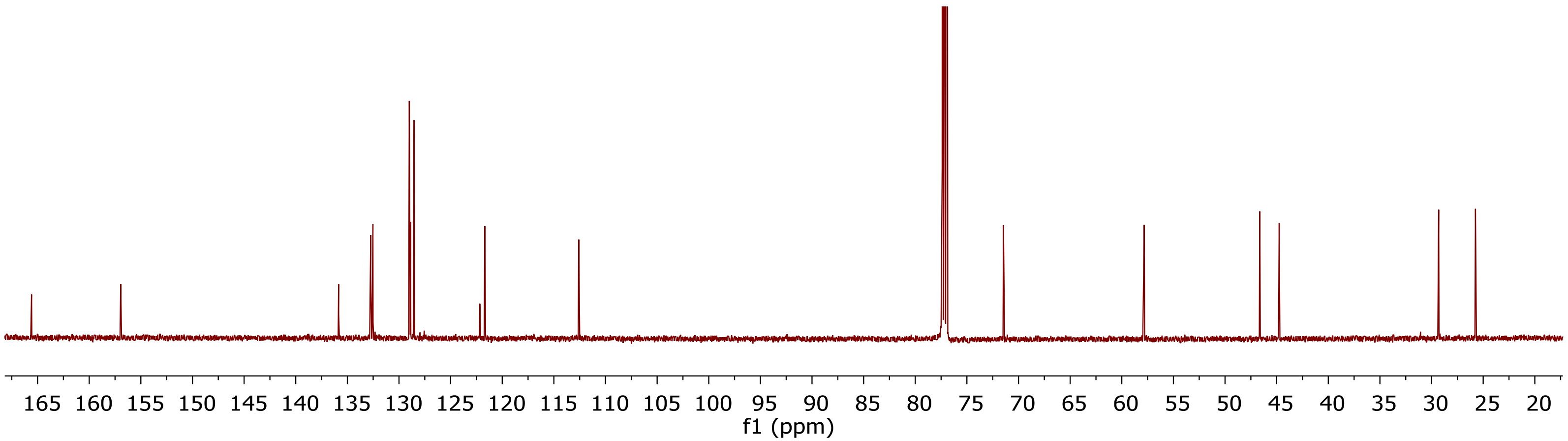

^{1}H and ^{13}C NMR Spectra for **S55**

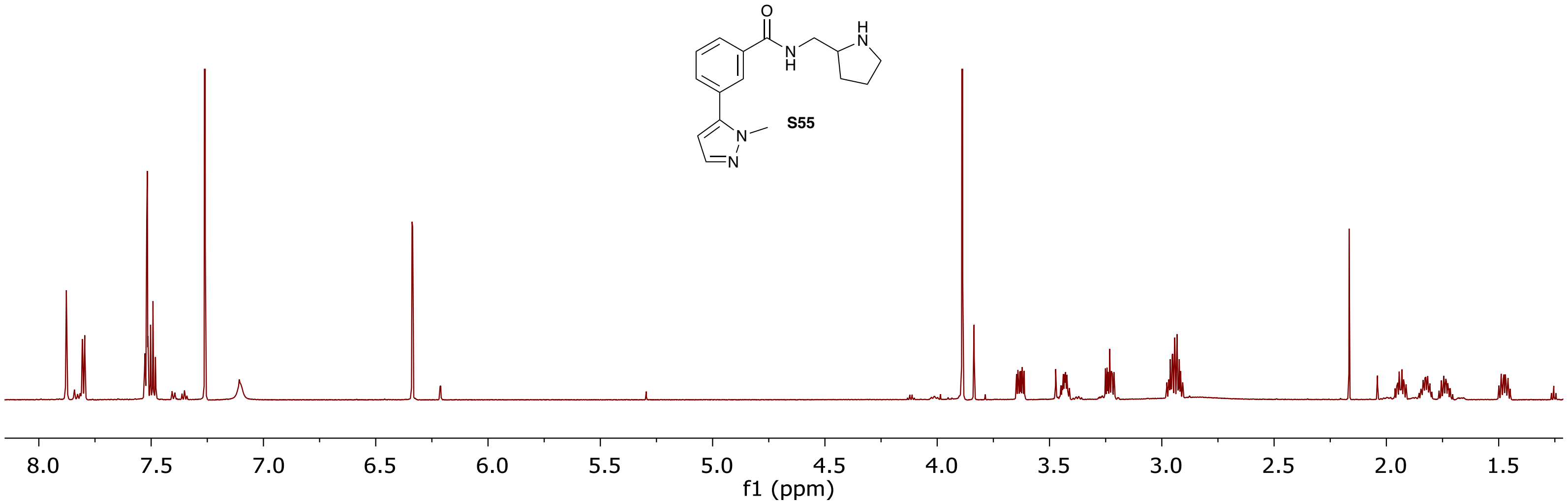

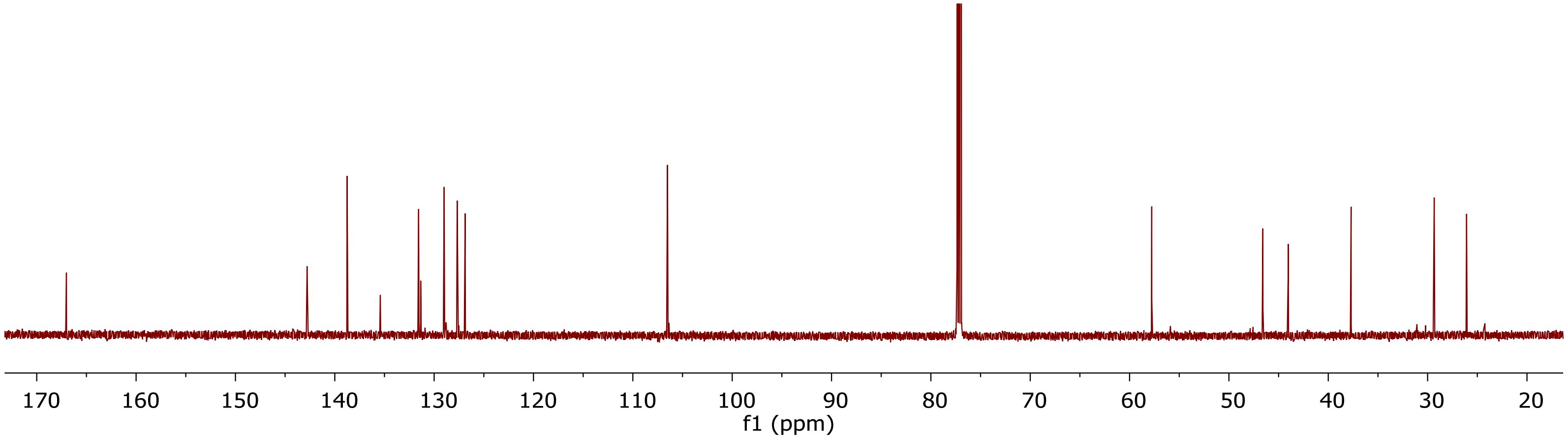

Supplement: MD-016-D4MD01004C-s001 [file MD-016-D4MD01004C-s001.pdf]
